# Novel EP2 Antagonist Attenuates Microgliosis and Memory Deficits in Pilocarpine-Induced Status Epilepticus Mice

**DOI:** 10.1021/acs.jmedchem.6c00172

**Published:** 2026-04-15

**Authors:** Thota Ganesh, Soheila Pourkhodadad, Paola Heman-Bozadas, Raymond Dingledine, Wenyi Wang, Varun Rawat, Nicholas H. Varvel, R. Jason Herr, Qin Jiang, Graham Johnson

**Affiliations:** † Department of Pharmacology and Chemical Biology, 1371 Emory University School of Medicine, 1510 Clifton Rd, Atlanta, Georgia 30322, United States; ‡ 17451 Curia Global Inc, 24 Corporate Circle, Albany, New York 12203, United States; § NuPharmAdvise LLC, Lakeside Dr, Sanbornton, New Hampshire 03269, United States

## Abstract

EP2 receptors promote neuroinflammation in several central nervous system diseases including status epilepticus (SE). Our laboratory has been optimizing selective small-molecule antagonists for EP2 receptors to determine their functional role in neuropathology and behavioral deficits in SE models, with a goal of developing an EP2 antagonist for clinical use. Through lead-optimization, we identified a novel brain- penetrant compound **BPN-37440**, which possesses excellent EP2 potency, selectivity against other prostanoid receptors, demonstrates anti-inflammatory actions in the BV2-hEP2 cellular model, and exhibits suitable in vitro ADME and in vivo PK properties. A brief exposure of **BPN-37440** after SE onset in mice attenuated microgliosis in the amygdala, cortex, and hippocampus CA3 region 4 days following recovery from SE. Moreover, memory deficits were prevented in mice at 1–1.5 months following SE. The results validate the notion that neuroinflammation promoted by EP2 exacerbates behavioral deficits, supporting further exploration of EP2 antagonists in the clinical setting.

## Graphical abstract

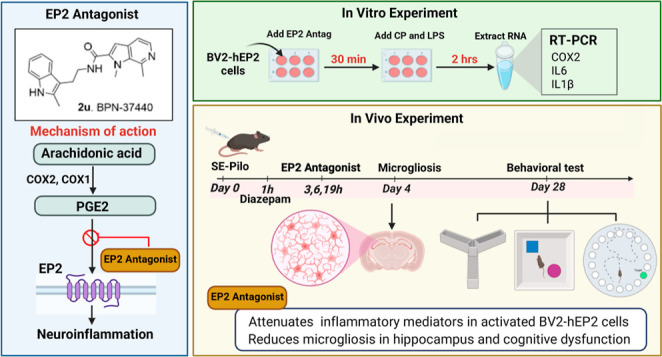

## Introduction

Acute brain injuries such as the seizures of status epilepticus (SE), traumatic brain injury and chronic neurological conditions including Alzheimer’s disease and Parkinson’s disease feature neuroinflammation with cognitive decline and are associated with profound loss of quality of life.^1,2^ Identifying novel biological targets that contribute to neuroinflammation and subsequent cognitive/memory deficits are essential for future therapeutic discovery. We and others have identified the activation of the prostaglandin-E2 receptor EP2 subtype as a major driver of the inflammatory reaction that ensues after brain injury, during chronic neurologic disease, and in response to systemic inflammatory insults.^3,4^ We have recently shown that pharmacological blockade of the EP2 receptor with a brain-penetrant EP2 antagonist reduces neuroinflammation and prevents long-term memory deficits in a mouse model of lipopolysaccharide (LPS)-induced sepsis-associated encephalopathy.^5^ Moreover, inhibition of EP2 also mitigates cognitive deficits in aged mice^6^ and in a brain injury model of SE.^7^ Our previously reported initial high-throughput screening (HTS) campaign yielded selective EP2 antagonists, but their selectivity against other prostanoid receptors and/or their ADME and PK properties were suboptimal.^7–9^ Further extensive structure activity relationship (SAR) exploration led to the identification of two novel lead compounds **BPN-37112** (**2p**) and **BPN-37440** (**2u**). These molecules display high EP2 antagonist potency and selectivity, acceptable ADME and PK properties, and blunt the effects of inflammation in a cellular model. Moreover, **BPN-37440** (**2u**) attenuates microgliosis in mice 4 days after pilocarpine-induced SE, and relieves memory deficits 28 days after SE.

## Results

### Synthesis and Structure Activity Relationship Studies

The recently developed lead EP2 antagonist **BPN-30343** (**1**, Figure 1) met most of the desired ADME and PK criteria required for development. It successfully passed dose-range finding (DRF) studies in Sprague–Dawley rats with essentially no changes in the clinical chemistry, hematology, behavior or histopathology after 7 days of daily dosing up to 1000 mg/kg^7^. However, when dogs were subjected to increasing dosages, **1** showed unexpected plateauing in plasma levels after the 60 mg/kg dose. The calculated No-Observed-Adverse-Effect Level (NOAEL)^10^ is 1000 mg/kg/day for rats and 60 mg/kg/day for dogs. The therapeutic index, based on ratio of NOAEL plasma AUC to the efficacious AUC in animals, was 35 for rat and 26 for dog.^7^ One possibility for the unexpected plateauing effect in dogs is due to differences in the stomach and intestinal pH of dogs (neutral-alkaline) compared to rats (acidic-neutral)^11,12^ that lead to differing degrees of compound ionization, which in turn can lead to differential absorption effects. Another contributing factor is the low- and pH dependent aqueous solubility of **1** that may also limit absorption (see Table 1). Another area of concern in terms of the clinical development of **1** was the observation that 93% of its metabolism proceeds through the single isoform CYP3A4 which has the potential to lead to clinical drug–drug interactions (DDI). To address these issues, we first designed and synthesized several derivatives in which the 2-methylindole fragment of **1** was replaced with various other heterocycles shown in Scheme 1. The syntheses of **1a-1ii** were achieved with advanced acid precursor **8** and commercially available heterocyclic amines **9** using HATU (Hexafluorophosphate Azabenzotriazole Tetramethyl Uronium)-mediated coupling reaction (Scheme 1). Unfortunately, this broad-based effort was unsuccessful in identifying EP2 antagonist compounds of improved activity and aqueous solubility (Table 1).

**Figure 1:**
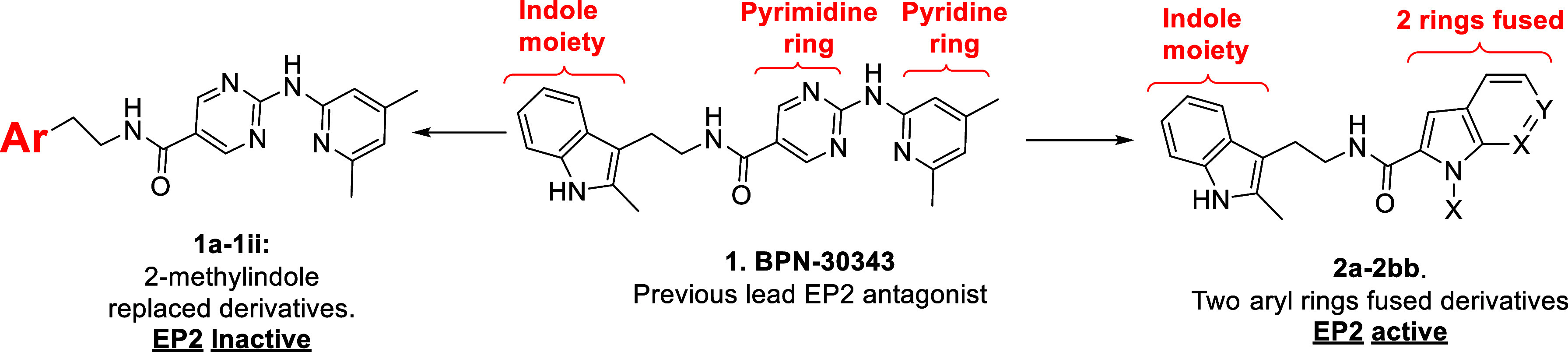
Novel compounds designed around the previous lead candidate molecule **1** (BPN-30343).

**Table 1:**
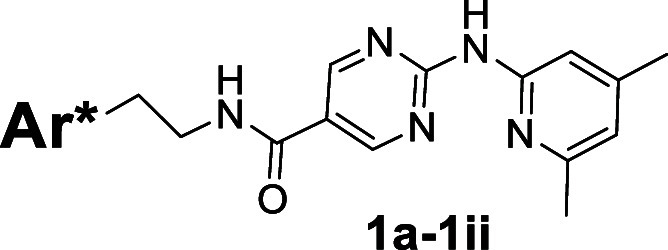

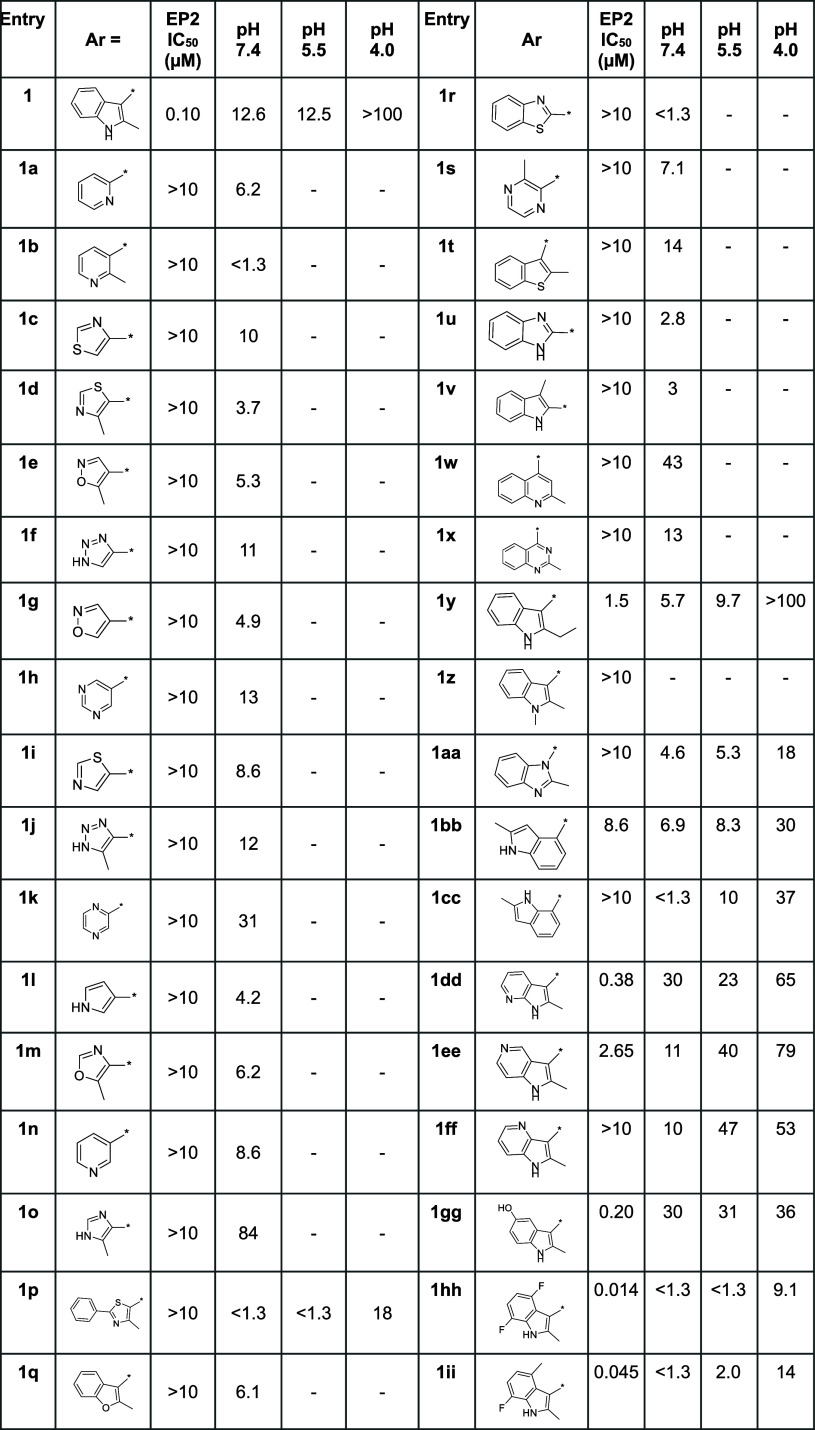
EP2 Potency and Aqueous Solubility of Left-Side 2-Methylindole Modified Analogs^a^

a IC_50_ was derived from a cAMP-driven TR-FRET assay and kinetic solubility is determined from UV-based method (see Experimental for details).

**Scheme 1:**
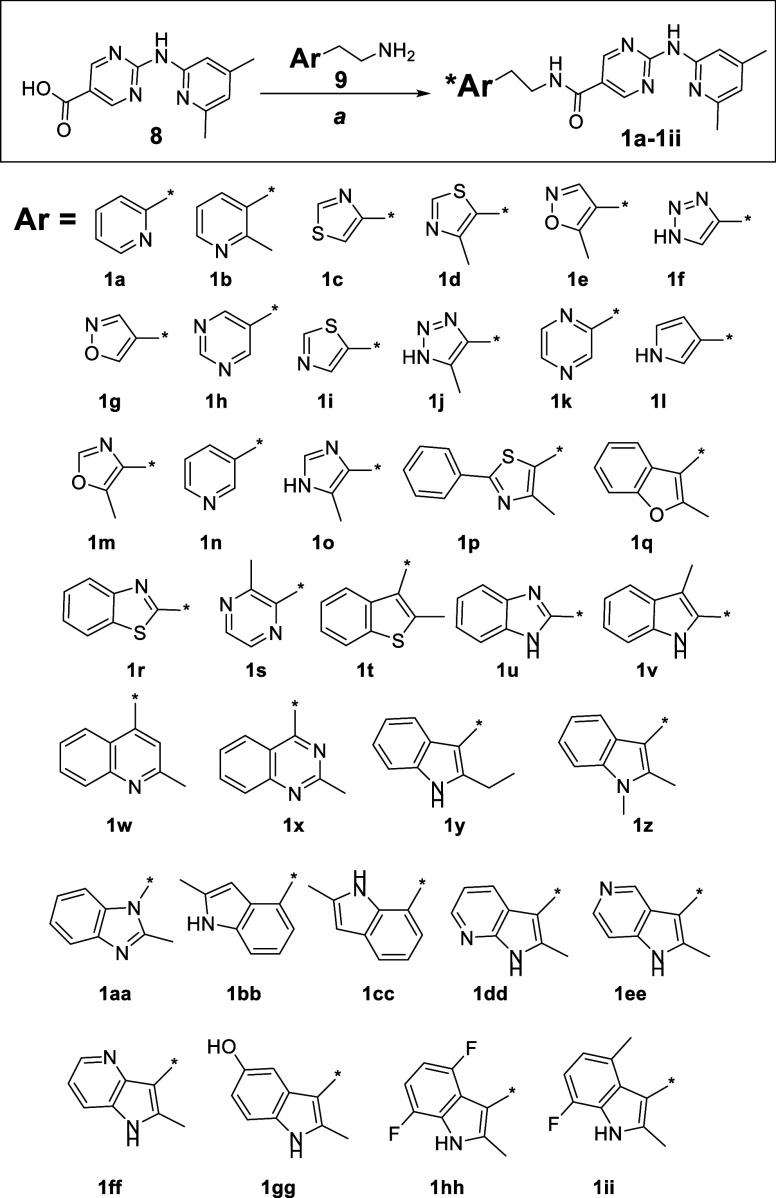
Reagents and Conditions: a. HATU, Et_3_N, DMF, rt, Yields (6–79%, Unoptimized)

We next turned our attention to developing analogs in which the left side 2-methylindole moiety was retained, but with modifications made to the right side pyridinyl-aminopyrimidine fragment, resulting in the straightforward synthesis of novel 1*H*-pyrrolo-[2,3]-pyridine-2-carboxamide derivatives **2a-2bb** (Figure 1). As depicted in Scheme 2, the starting material 2-(2-methyl-1H-indol-3-yl-ethan-1-amine (**3**) was coupled to either 1*H*-indole-2-carboxylic acid (**4**) or to 1*H*-pyrrolo­[2,3]­pyridine-2-carboxylic acids **(5–7)** in the presence of HATU^13^ to provide the final products **2a-2bb** in moderate to good yields.

**Scheme 2:**
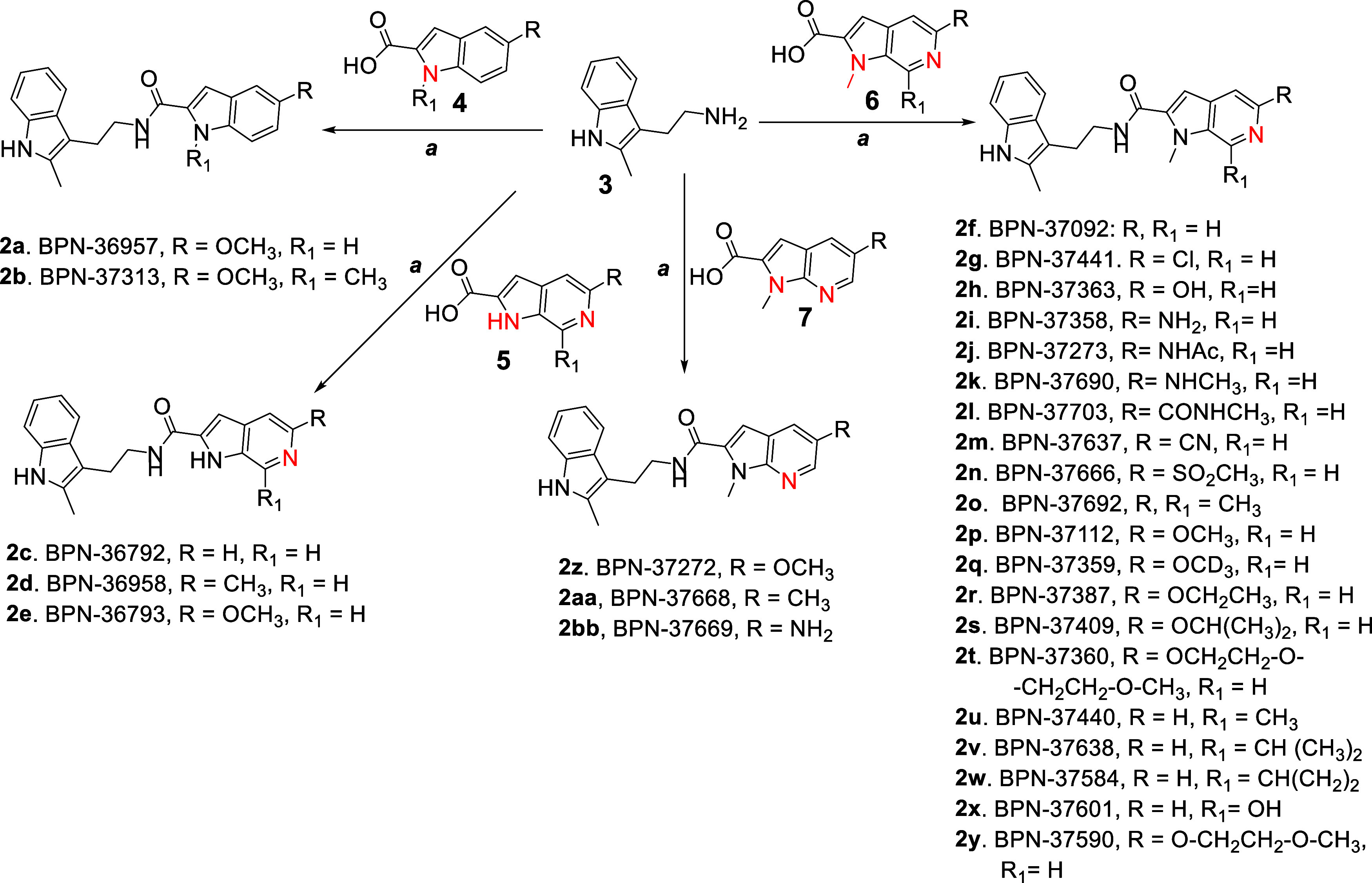
Reagents and Conditions: a. HATU, Et_3_N, DMF, rt. Yields (7–86%, Unoptimized)

### EP2 Potency and Kinetic Aqueous Solubility, SAR Studies

All synthesized compounds were tested for potency and selectivity using a cAMP-derived TR-FRET assay.^8^ C6-Glioma cells overexpressing human-EP2 receptors, human-EP4, human-DP1 or human-IP receptors were used for the assay. These cells upon activation with PGE2 (natural endogenous agonist for EP2 and EP4), BW245C (synthetic agonist for the DP1 receptor) and iloprost (synthetic agonist for the IP receptor) produce cyclic AMP.^14,15^ In parallel, we also tested these compounds for aqueous solubility (kinetic) at 3 different pH conditions (pH 7.4, 5.5, 4.0) to identify compounds with improved aqueous solubility that was less dependent on the pH of various biological compartments.

Unfortunately, it was found that all of the derivatives **1a**-**1ff** bearing various heterocycles to replace the left side unsubstituted 2-methylindole ring were ineffective at antagonizing EP2 (Table 1). Interestingly, the incorporation of substituents such as hydroxyl, fluorine, or methyl groups (see **1ff-ii**) onto the 2-methylindole moiety were found to restore high EP2 potency. These results reinforce the notion that the 2-methylindole moiety is a privileged structure that is essential for maintaining EP2 potency within the compound **1** ^16,17^ class. On the other hand, the aqueous solubility among analogs **1a-1ii** was inferior to the previous lead candidate **1** (Table 1).

Among the right-side modified compounds, a fused 1*H*-indole-3-yl-1*H*-indole-2-carboxamide derivative **2a** displayed comparable IC_50_ potency to **1**, and 1-methyl-1*H*-indole-3-yl-1*H*-indole-2-carboxamide **2b** displayed a 2-fold increase in potency (Table 2). Although initially promising, however, these two compounds displayed low aqueous solubility when compared to **1**. The pyrrolo-pyridine-carboxamide derivatives **2c**–**2e** showed 2-to-3-fold less potency, but 4-to-5-fold improved aqueous solubility than **1**, potentially due to the hydrogen bonding ability of the NH moiety in the right side 1*H*-pyrrolo­[2,3-*c*]-pyridine (Table 2). Alternatively, N-methylated 1*H*-pyrrolo­[2,3-*c*]-pyridine **2f** demonstrated 2-fold higher potency with a 5-fold improved aqueous solubility versus **1**, and so was selected for further modification. Inclusion of a chlorine onto the fused pyridine ring **(2g)** resulted in 10-fold higher potency, but significantly reduced aqueous solubility. Nevertheless, results from both **2f** and **2g** suggested that the N-methylation of the heterocyclic ring may serve as optimal for potency. Among other 1-methyl-1*H*-pyrrolo­[2,3-*b*]­pyridine derivatives, **2h, 2n** and **2t** were found to demonstrate good aqueous solubility (>60 μM), likely due to the presence of a polar functional group (OH, SO_2_Me, ethylene-glycol ether), but also exhibited significantly diminished EP2 potency. Compounds **2k, 2o, 2p, 2q** and **2u** retained high EP2 potency (IC_50_ < 50 nM) and with aqueous solubility (>50 μM) 3-to-5-fold higher than **1** (Table 2). While compound **2l** was both weakly active and poorly soluble, compound **2m** displayed equal potency to **1** and with moderate aqueous solubility (<50 μM). Likewise, compounds **2v**–**x** showed weak potency against EP2 while displaying superior aqueous solubility to **1**. Interestingly, extension of the alkoxy moiety to a methoxy–ethoxy-ethyl ether moiety (**2y**) afforded both higher EP2 potency and improved solubility (>45 μM). We also synthesized and tested an additional group of fused indole-1*H*-pyrrolo­[2,3-*b*]-pyridine-2-carboxamide derivatives **2z, 2aa** and **2bb**. Among these, methoxy derivative **2z** and methyl derivative **2aa** found to be potent analogs, but they did not show improved solubility, whereas amino-derivative **2bb** demonstrated equal potency, but with >5-fold increased aqueous solubility.

**Table 2:**
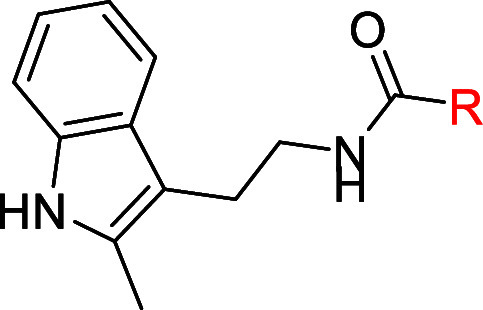

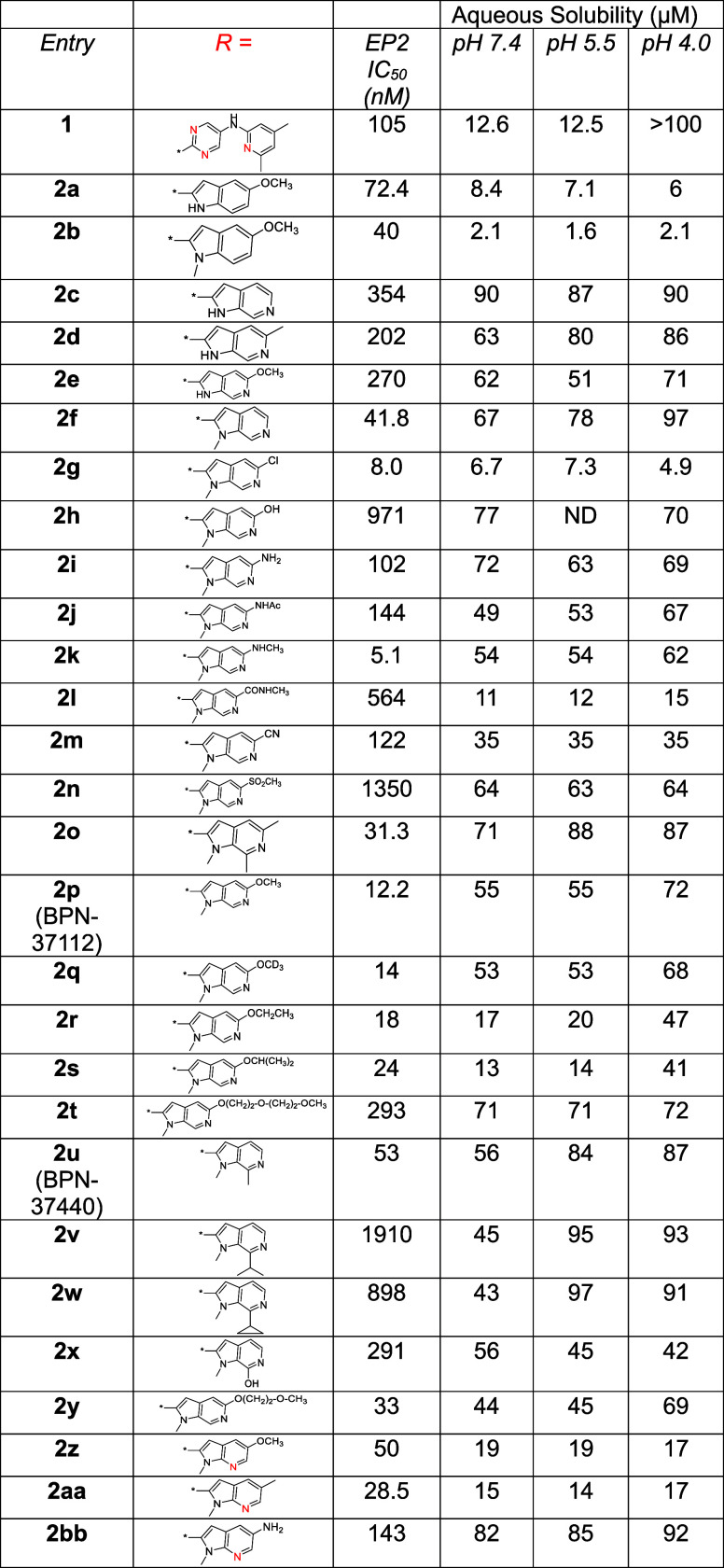
EP2 Potency, Aqueous Solubility Properties of Novel Compounds^a^

a IC_50_ was derived from a cAMP-driven TR-FRET assay (see details below).

Although compounds **2k** and **2o** displayed good EP2 potency and requisite aqueous solubility (Table 2), **2o** displayed a high efflux-ratio (18.1) in the membrane permeability (MDCK-MDR1) assay (Table 3). On the other hand, we decided not to progress compound **2k** due to the potential metabolic liability associated with an *N*-methyl amine moiety. Compound **2y** was not further evaluated as it synthesis was low yielding (overall yield, <2%), compared to the overall synthesis yields obtained for **2p** (50%) and **2u** (53%). For these reasons, **2k**, **2o** and **2y** were not selected for further characterization. Overall, we identified a small set of compounds including **2p, 2u** which met both our IC_50_ potency (<100 nM) and aqueous solubility (>50 μM) criteria and so were selected for further characterization.

**Table 3:** ADME Properties of Selected pyrrolo­[2,3-*c*] and [2,3-*b*] Pyridine-2-Carboxamide Compounds^a^

entry	MDCK-MDR1 permeability (× 10^–6^ cm/s)			hERG (IC_50_, μM)	CYP inhibition (IC_50_, μM)				plasma protein binding (%)				
	B-to-A	A-to-B	efflux ratio		2B6	2D6	3A4 (mid.)	3A4 (test.)	human	rat	mouse	dog	cyano
**1**	31.2	7.04	4.43	>10	>100	>100	78	8.5	98.5	98.5	98.6	99.6	98.2
**2c**	82.9	2.82	29.4	>10	16	0.31	0.87	0.45	97.1	96.6	97.3	95.3	99.3
**2d**	62.0	1.61	38.6	9.3	30	42	33	14	ND	ND	ND	ND	ND
**2e**	84.0	14.7	5.72	>10	25	6.4	10	1.3	99.5	99.7	99.8	99.5	99.5
**2f**	64.6	5.52	11.7	>10	2.1	1.1	0.1	0.07	96.1	96.8	99.5	94.2	94.5
**2i**	63	1.91	33	>10	50	46	11	11	94.6	96.2	98.2	94.1	90.9
**2j**	75	2.14	35	>10	24	11	1.5	1.2	93.1	97.9	96.6	92.0	92.9
**2o**	64	3.53	18.1	ND	ND	ND	ND	ND	98.3	98.0	96.8	95.4	95.6
**2p**	34.5	32.3	1.07	>10	>50	7.6	5.6	1.3	98.1	98.7	99.6	98.2	97.9
**2q**	34.9	28.2	1.24	>10	38	16	11	0.6	98.4	98.8	99.5	98.4	97.5
**2u**	28.0	8.36	3.36	>10	11	4.5	0.72	0.43	95.1	96.4	98.7	99.4	99.5
**2y**	50.5	18.7	2.7	3	>50	15	6.4	3.1	94.7	98.7	99.3	93.7	91.1
**2z**	51.0	42.6	1.2	>10	16	20	13	38	98.8	99.0	99.3	99.8	98.9

a ND = not determined.

### IC_50_ and Schild Potency and Selectivity of Leads BPN-37112 (**2p**) and BPN-37440 (**2u**)

From these SAR studies, two compounds **BPN-37112** and **BPN37440** emerged as promising leads in several ways. As shown in Figure 2, the IC_50_ potency for all compounds against EP2 function is determined using a single concentration of agonist PGE_2_ (10 nM) and varying concentrations of test compound between 0.01 and 10 μM. Compound **BPN-37112** exhibited IC_50_ 8.9 nM against EP2, but >10 μM against the DP1, EP4 and IP receptors confirming its high EP2 selectivity (>150–600-fold against other receptors, Supporting Information Figure 1). Similarly, compound **BPN-37440** exhibited an EP2 IC_50_ potency of 60 nM and >10 μM against other receptors indicating 175-fold selectivity (Figure 2A,B). To select our lead, we set forth a selectivity criteria at least 100-fold against other prostaglandin receptors. We synthesized three independent batches of **BPN-37440**, and as shown in Figure 2A, all three batches displayed EP2 potency with <2-fold variance (IC_50_ = 53–60 nM) confirming a consistent potency across all batches. Likewise, two independent batches of **BPN-37112** displayed potency within 2-fold variance [IC_50_ 8.9 (Figure 2A) vs 15.6 nM (not shown)]. In the same assay, the positive control (previous lead) **1** displayed IC_50_ average potency of 105 nM. Potential cytotoxicity was evaluated using the parent (C6-glioma) cell line using a cell viability assay. When compared to the positive control doxorubicin IC_50_ (50 nM), both compounds **BPN-37112** and **BPN-37440** demonstrated IC_50_ > 50 μM, which translates to a >900-fold in vitro therapeutic index (Figure 2C), further supporting these two compounds for advancement. Likewise, a methoxy-ethyl-ether compound **2y** (**BPN-37590**) which demonstrated IC_50_ = 33 nM against EP2 (Table 2), also showed >50 μM IC_50_ in a cytotoxicity assay (Figure 2C), indicating a general nontoxicity trend within the class.

**Figure 2:**
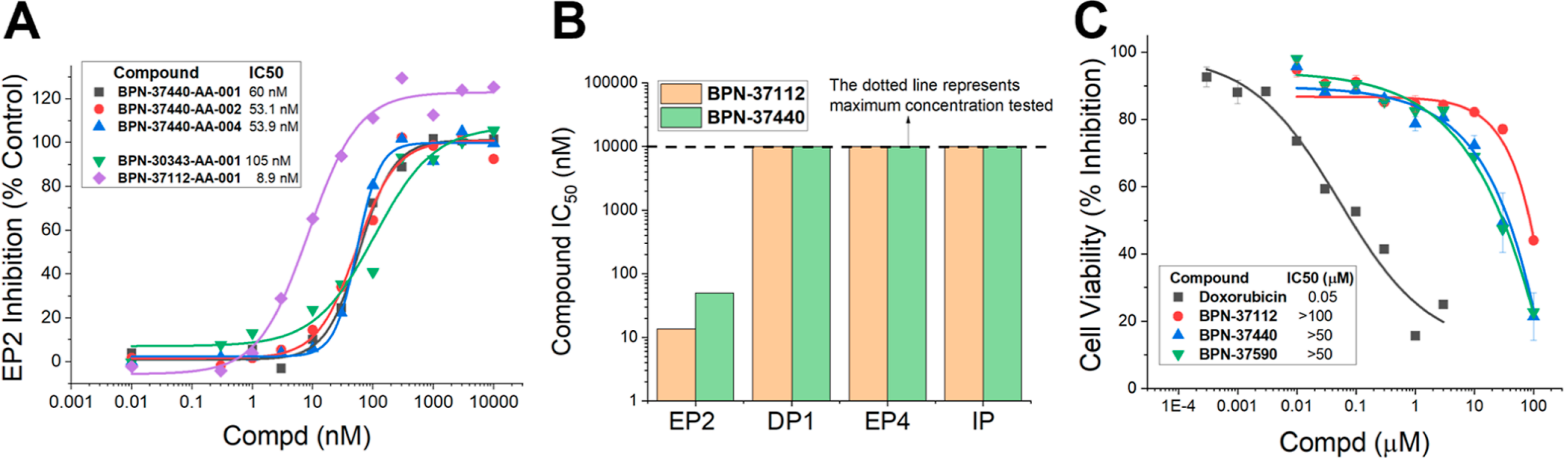
(A) IC_50_ Potency of compounds **BPN-37112 (2p**) and **BPN-37440** (**2u**) against EP2. (B) Selective IC_50_ potency to EP2 vs DP1, EP4 and IP receptors. (C) Cytotoxicity against C6-glioma cell line. C6-Glioma cell lines overexpressing EP2, DP1, EP4 and IP receptors are used with activation with their respective agonist. A 10 nM PGE2 for EP2 and EP4 activation, 1 nM BW245C to activate DP1 receptors, and 1 nM iloprost to activate IP receptors were used in the assay to induce cAMP production by these receptors with varying concentrations of the antagonist molecules. In each assay a positive control compound was used (see Supporting Information Figure 1). Parent C6-glioma cells are used for cell viability assay using positive control compound, doxorubicin.

Knowing that the IC_50_ potency would depend on various factors including agonist concentration and receptor density; we further evaluated the potency of these compounds and mode of inhibition by following a Schild assay protocol.^18,19^ In this assay, EP2 agonist PGE2 was tested for EC_50_ in the presence of increasing concentrations of the antagonist to determine the EC_50_ fold change. As shown in Supporting Information Figure 2 and Figure 3A,C, compounds **2p** and **2u** exhibited a concentration-dependent rightward shift of PGE2 EC_50_. A Schild regression analysis indicates a Schild K_B_ of 1.1 nM for **2p** (Supporting Information Figure 2) and 5.65 nM for **2u** (Figure 3). Both compounds displayed a Schild slope of 1, indicating these compounds are highly potent and competitive inhibitors. Schild K_B_ represents a concentration required to induce a 2-fold rightward shift of agonist EC_50_.

**Figure 3:**
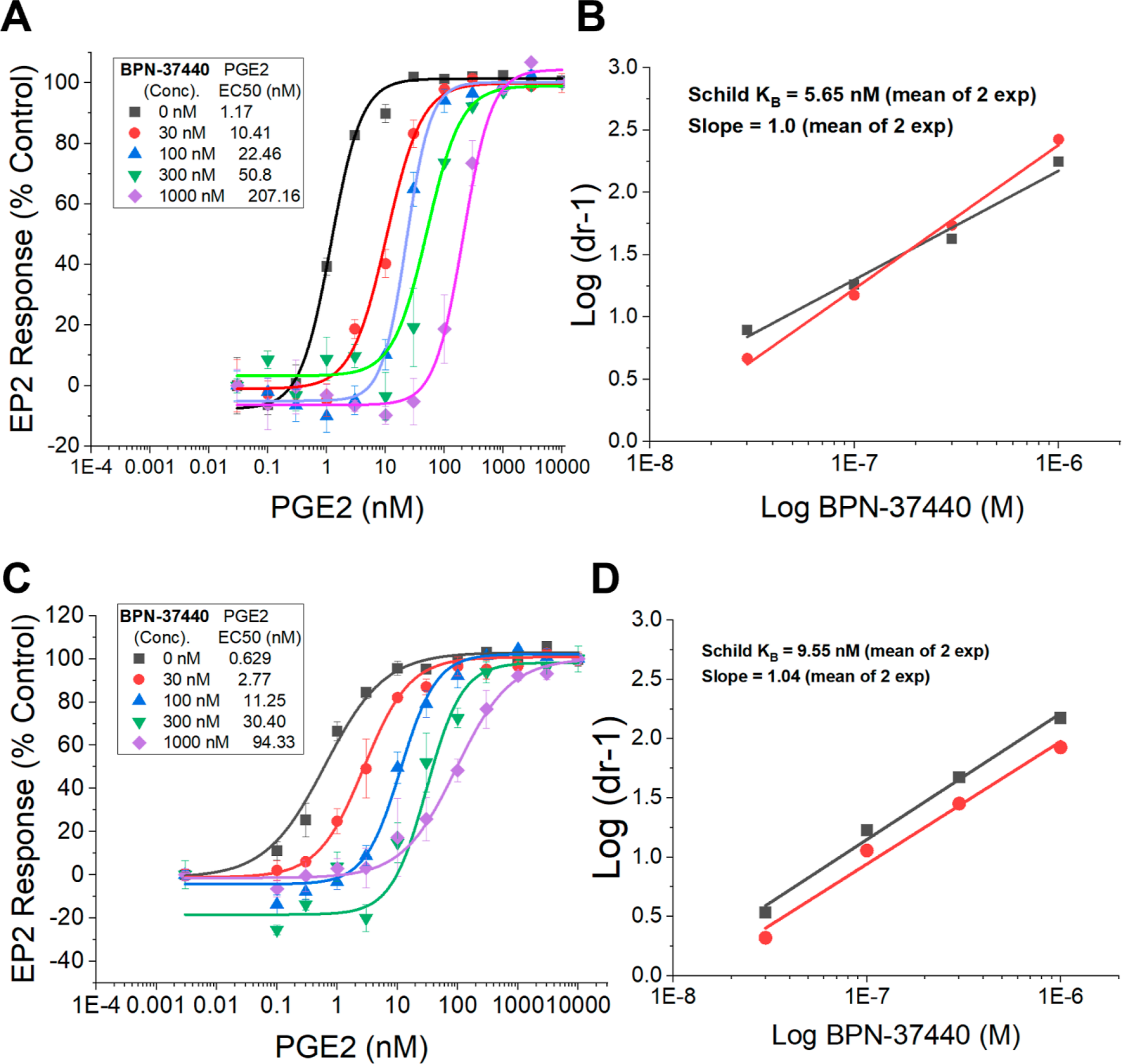
**BPN-37440** Potency by Schild assay. **BPN-37440** displayed a concentration-dependent rightward shift of PGE2 EC_50_. Schild analysis resulted in a K_B_ = 5.65 nM (for human-EP2 (A,B)) and K_B_ = 9.55 nM (for mouse-EP2, (C,D)) with a Schild slope of 1 in both the experiments. Schild plots with slope from 2 independent experiments are shown (B,D). Schild K_B_ = Concentration required to bring 2-fold rightward shift of agonist (PGE2) EC_50_. Schild slope of 1 indicates a competitive inhibition of the EP2 receptor.

The amino acid sequence homology between mouse and human EP2 receptor is 85%.^20^ While small differences exist at N-terminus of the mouse protein with 25 extra amino acids,^21^ the overall EP2 protein is similar in both human and mouse, and therefore we anticipated similar potency by compound **BPN-37440** and others in the class across human and mouse EP2. To validate this point, we tested **BPN-37440** in C6-glioma cells overexpressing mouse-EP2 using the Schild assay. As shown in Figure 3C,D, **BPN-37440** displayed 1.6-fold lower EP2 potency (Schild K_B_ = 9.55 nM) against mouse EP2, confirming dual human/mouse potency.

### ADME-PK Properties of Selected Best Compounds

To select the lead candidate(s) for advancement to preclinical evaluation, we selected several compounds with optimal EP2 potency and aqueous solubility properties and evaluated them for further in-depth ADME characteristics. High intrinsic membrane permeability and avoidance of P_gp_-efflux are key factors that determine if a small molecule can be absorbed and cross the biological membranes to reach the site of action that leads to efficacy.^22^ As shown in Table 3, several compounds in the class displayed excellent apical-to-basolateral (A-to-B) permeability 1.6–32.3 × 10^–6^ cm/s in the MDR1-MDCK assay, but several of them, including **2c–e, 2f, 2i–j**, **2o**, also served as substrates for P_gp_-mediated efflux, resulting in efflux ratios >5.7. Interestingly, compounds **2p, 2q**, **2u, 2y** and **2z** displayed not only high (A-to-B) apparent permeability (P_app_), but also with lower efflux (ER ratios < 3.4), suggesting that these compounds were suitable for advanced evaluation.

Cytochrome P450 (CYP) enzymes are responsible for much observed drug metabolism. Among several CYPs, CYP3A4 and CYP2D6 collectively accounts for >50% of CYP-mediated drug metabolism followed by CYP2B6 and thus are deemed clinically relevant.^23^ We subjected several EP2 active potent compounds for potential inhibitory actions on these key CYP isoforms, 2B6, 2D6 and 3A4. As shown in Table 3, none of the tested compounds in the class inhibited CYP2B6 (IC_50_ > 1 μM). Only **2c** displayed submicromolar IC_50_ against CYP2D6. However, compound **2f** displayed 70–100 nM IC_50_ against CYP3A4 in assays using either midazolam or testosterone as test probes, indicating a strong potential for drug–drug-interaction (DDI). Compound **BPN-37440** (**2u**) displayed a submicromolar inhibitory activity against CYP3A4 (again using both test probes), indicating that additional studies to address DDI liabilities are warranted for this compound before nominating it for clinical evaluation. Interestingly, **2e, 2p** and **2q** displayed low potency against CYP3A4 when midazolam was used as a substrate, but high potency when testosterone was used, potentially indicating that these compounds can influence the activity of CPY3A4, impacting the potential for DDI.^24^ It is worth pointing out that for a life-threatening condition or for an acute treatment regimen in patients (1 day), the risks with CYP inhibition can be mitigated with dose adjustment and therapeutic monitoring, and therefore CYP3A4 inhibition by **BPN-37440** was considered a lesser concern at this stage. Furthermore, **BPN-37440** showed 40, 19, 15, and 0.9 μM IC_50_ against other CYP isoforms, 1A2, 2C9, 2C19 and 2C8 respectively.

Plasma protein binding (PPB) of small molecules can have very important ramifications on drug efficacy as dictated by drug distribution, metabolism and elimination. To determine the concentration of free drug in plasma (fraction unbound), we tested several compounds for binding to plasma proteins from human, rat, mouse, dog, and cyno species. As shown in Table 3, all compounds bound to plasma proteins of all species with >95%, except **2i** and **2j**, which displayed <95% binding to plasma proteins from human, dog and cyno, perhaps due to the effects of the amino and acetamide moieties. While **2i** and **2j** demonstrate excellent free plasma protein fraction values, these compounds were found to be strong efflux-pump substrates in the MRD1-MDCK cell permeability assay (Table 3), preventing their selection for advancement. However, **BPN-37440** appeared as a suitable molecule due to its acceptable fractions unbound in human (4.9%), rat (3.6%) and mouse (1.3%) plasma. Therefore, it was ultimately decided to advance **BPN-37440** for in vivo PK/PD evaluation. The second-best molecule **BPN-37112** also displayed an acceptable free fraction in human (1.9%), rat (1.3%) and mouse (0.4%) fractions. It is anticipated that with low fraction unbound in mouse, **BPN-37112** may result in in vivo PK/PD outcomes that are more difficult to extrapolate versus results from studies of **BPN-37440**.^25^ It is important to emphasize that none of the compounds including **BPN-37440** inhibit *h*-ERG, suggesting the derivatives in the class are unlikely to have adverse effects on cardiac arrhythmias (Table 3).

### Pharmacokinetics of the Candidate Molecules **BPN-37112** and **BPN-37440**

Since efficacy is evaluated in mice, we determined the pharmacokinetics and brain exposure characteristics of both **BPN-37112** and **BPN-37440** in C57BL/6 mice. As shown in Figure 4, following intraperitoneal (ip) injection of 10 mg/kg, **BPN-37112** displayed rapid absorption with excellent exposure in plasma and brain tissues, with a mean (*n* = 3 mice) plasma half-life ∼0.9 h, AUC_inf_ = 7099 h × ng/mL in plasma and 2455 h × ng/mL in brain tissues. The brain-Kp (brain-to-plasma ratio) values determined are found in the range of 0.35–0.79 (Figure 4A,B), which is consistent with observed permeability data. Oral dosing of **BPN-37112** in SD-rats (10 mg/kg) indicated a plasma half-life of 0.92 h and with an oral bioavailability (F) of 45%, with a brain-Kp > 1 (Table 4). Likewise, **BPN-37440** was rapidly absorbed with excellent exposure in plasma and brain tissues, with a mean (*n* = 3 mice) plasma half-life ∼1.8 h, AUC_last_ = 4645 h × ng/mL in plasma and 1345 h × ng/mL in brain tissues. The brain-Kp value of 0.29–0.76 confirmed partition into the brain (Figure 4C,D and Table 4). It is worth indicating that the plasma exposure levels (with 10 mg/kg, ip) for these two compounds is about 10-fold higher than the Schild potency of the molecules (Figure 4). By oral gavage (po) dosing in SD-rats **BPN-37440** at 10 mg/kg displayed a plasma half-life of 1.78 h and an oral bioavailability of 88% (Table 4). These parameters suggest that these compounds are suitable for in vivo efficacy studies and additional preclinical evaluation.

**Figure 4:**
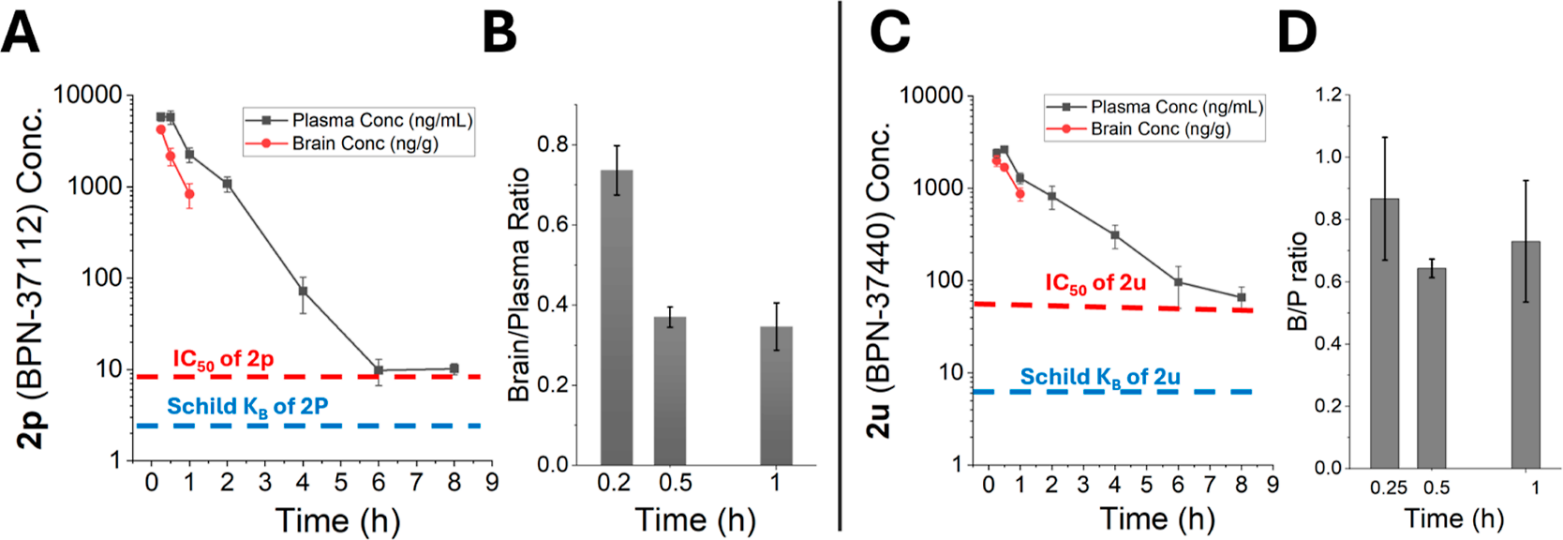
Plasma and brain pharmacokinetics of **BPN-37112** (**2p**) (A,B) and **BPN-37440** (**2u**) (C,D). 10 mg/kg dose via intraperitoneal (ip) injection was made and blood and brain samples investigated for plasma and brain concentrations by LC–MS/MS. C57BL6 mice (*n* = 3 mice) are used. A vehicle containing 10% DMSO, 50% PEG400 and 40% water was used to dissolve and inject the compound.

**Table 4:** Pharmacokinetic Parameters of BPN-37112 and BPN-37440^a^

compd	species	route	dose (mg/kg)	matrix	*T*_max_	*C*_max_ or (C_o/_ *C*_max)_	AUC_last_ (h × ng/mL)	AUC_inf_ (h × ng/mL)	*T*_1/2_(h)	MRT_last_(h)	CL (mL/min/kg)	*F* (%)	brain-Kp
**BPN-37112**	C57BL6-mice	ip	10	plasma	0.25	5830	7093	7099	0.87	1.01	-	-	
				brain	0.25	4223	2075	2455	-	-	-	-	0.35–0.79
	SD-rats	po	10	plasma	0.5	462	1101	1106	0.92	1.52		45	1.17–3.54
		iv	2	plasma	-	3135	490	494	0.37	0.20	67.42	-	-
**BPN-37440**	C57BL6-mice	ip	10	plasma	0.5	2624	4645	4794	1.79	1.77	-	-	
				brain	0.25	1985	1345		-	-	-	-	0.29–0.76
	SD-rats	po	10	plasma	0.5	1360	3786	3791	0.76	1.78		88	
		iv	2	plasma	-	1481	865	876	1.86	0.8	38.0	-	-

a *F* = Oral bioavailability; brain-Kp = brain-to-plasma ratio determined at various time points. ip = intraperitoneal, iv = intravenous, po = oral gavage. MRT = mean residence time.

### EP2 Antagonists **BPN-37112** and **BPN-37440** Dampen the Induction of Inflammatory Mediators in **BV2-hEP2** Microglia

We have recently demonstrated that EP2 activation is coupled to neuroinflammatory signaling in in vitro cellular models^16,17^ and in vivo models of acute and chronic brain injuries.^7,26^ To determine anti-inflammatory activity, we tested the lead molecules using a human-EP2 overexpressed BV2 microglial (**BV2-hEP2**) cellular model.^27^ As shown in Figure 5, when **BV2-hEP2** microglia are activated with LPS and a selective EP2 agonist (CP544326 ^28^), the inflammatory mediator cyclooxygenase-2 (COX-2) and cytokines IL-1β and IL6 were significantly induced compared to the controls [no treatment, LPS alone, or agonist (CP544326) alone]. Interestingly, the induction of these inflammatory markers was significantly attenuated by pretreatment with the novel EP2 antagonist **BPN-37440** (Figure 5A–C). To determine the IC_50_ activity against IL-1β, a percent inhibition was plotted against the **BPN-37440** concentration. From this analysis, **BPN-37440** demonstrated IC_50_ = 21 nM against IL-1β induction (Figure 5D) and 42 nM IC_50_ against COX-2 induction (data not shown), suggesting it is a highly potent anti-inflammatory molecule in this cellular model. Similarly, compound **BPN-37112** also displayed anti-inflammatory effects by suppressing IL-1β, IL-6 and COX-2 in a concentration-dependent manner (see Supporting Information Figure 3).

**Figure 5:**
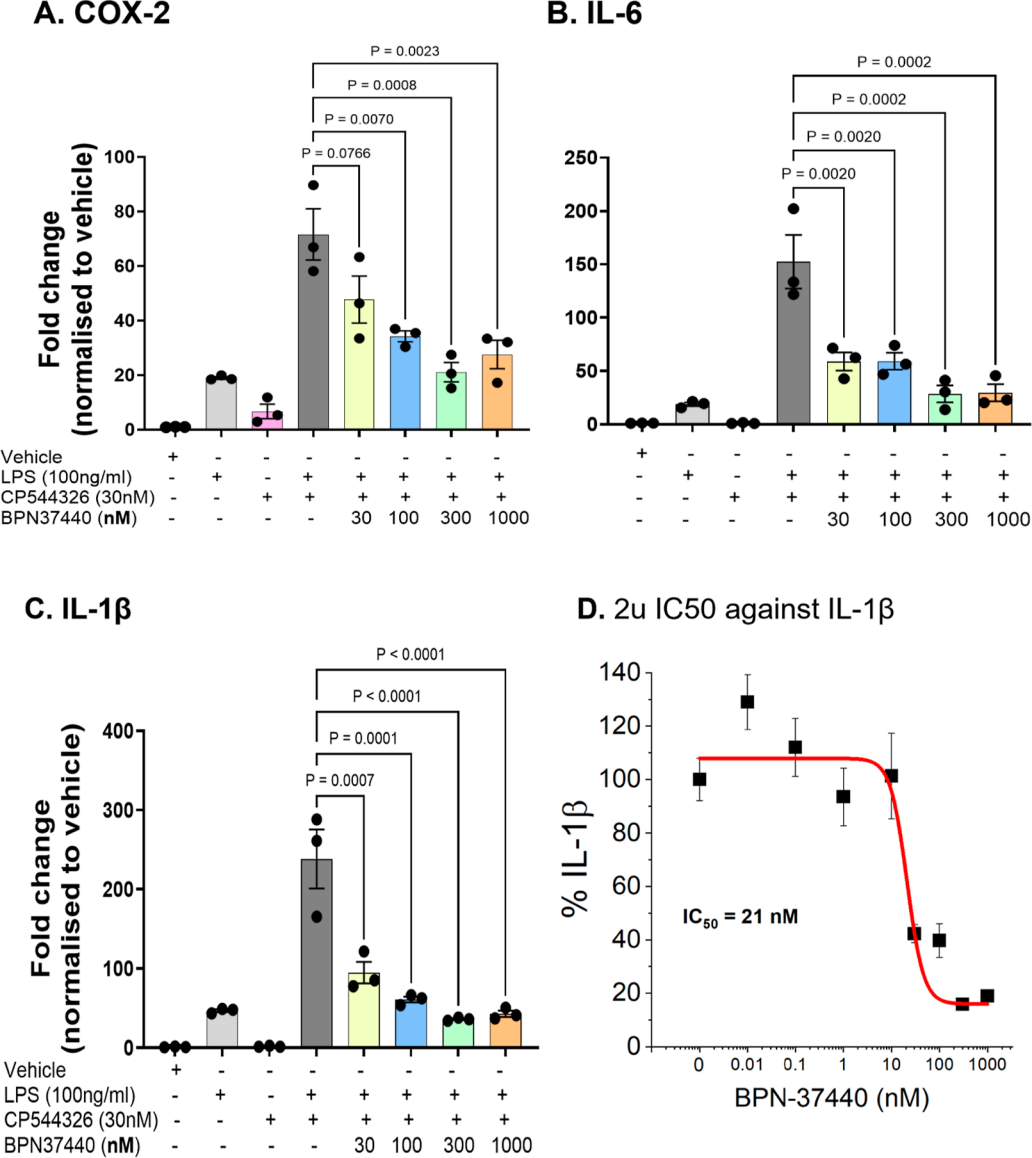
mRNA Gene expression of inflammatory mediators in BV2 microglia cell line overexpressed with human EP2 receptor (BV2-hEP2) with and without LPS, EP2 agonist (CP544326) and EP2 antagonist **BPN-37440**. Each dot on panel A–C represents a treated well from which duplicate RNA extraction was done to carry out qRT-PCR. One-way ANOVA with Dunnett posthoc test is used to analyze the data and the *P* values are shown in the graph. (D) Compound **BPN-37440** displayed IC_50_ = 21 nM against IL-1β expression in BV2-hEP2 cells (9 concentrations (*n* = 3 wells per concentration) are used to analyze the data.

### EP2 Antagonist **BPN-37440** Attenuates Microgliosis in a Mouse Model of Status Epilepticus

Status epilepticus (SE) (defined as a continuous seizure activity lasting >5 min) is associated with significant morbidity, mortality and acute brain inflammatory pathology, which subsequently results in cognitive and memory deficits extending long after SE.^29^ We have recently used the pilocarpine-induced SE mouse model to establish a proof-of-concept that EP2 antagonism following SE mitigates the ensuing inflammatory burst and behavioral deficits using lead candidate **1**.^7^ To validate the anti-inflammatory efficacy by novel lead candidate **BPN-37440**, we used the experimental design shown in Figure 6A. Mice were randomized to pilocarpine or saline first to create SE and non-SE control mice. Then, diazepam was injected to terminate the seizure in mice (control mice were also treated similarly to have matching controls). **BPN-37440** was injected (10 mg/kg) at 3, 6, and 19 h after SE onset. As shown in Figure 6B, all the mice lost weight on day 1 due to various chemical reagents including the diazepam they received, but from day 2 onward they recovered from this loss. Interestingly, the SE group that received only the vehicle continued to lose weight until day 2 and then slowly recovered over time to day 4, whereas SE mice treated with EP2 antagonist **BPN-37440** recovered faster than the vehicle group (Figure 6B). Analysis of microgliosis by monitoring IBA 1 positive area covered 4 days post-SE indicated that SE results in increased microgliosis compared to non-SE controls 4 days post SE. Treatment with EP2 antagonist **BPN-37440** significantly attenuated the microgliosis in amygdala, cortex and hippocampus CA3 region (Figure 6C–E) and showed a reducing trend in hippocampus CA1 region (Figure 6F). Representative fluorescence images showing IBA covered area in different brain regions from treatment groups are shown in Supporting Information Figure 4. This validates the previous findings^7^ that EP2 antagonism, irrespective of chemical structure, broadly dampens the brain inflammatory reaction.

**Figure 6:**
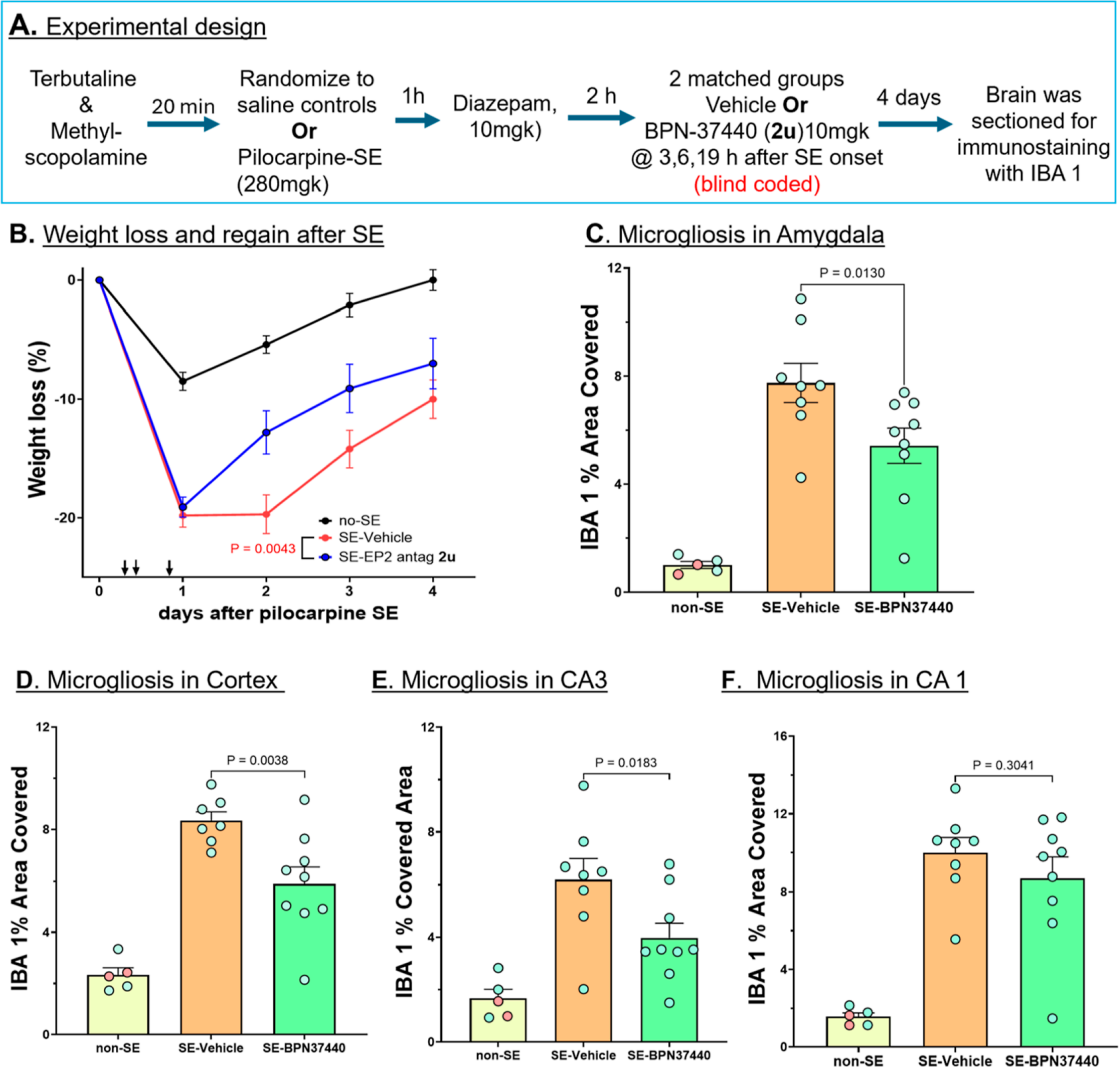
Antimicrogliosis effect of compound **BPN-37440** in pilocarpine-induced SE mouse model. (A) Experimental design. (B) Weight regain of the no-SE, pilocarpine SE-vehicle and pilocarpine SE-**BPN-37440** treated mice. (C–F) Microgliosis measured by immunohistochemistry analyzing the IBA 1 area covered in the amygdala (C), cortex (D) and hippocampus CA3 region (E) and hippocampus CA1 region (F). In the non-SE group both vehicle and drug treated animals are grouped and color coded. Mice (*n* = 5–9/group) are derived from one experimental batch (cohort). 1-way ANOVA with Sidak’s posthoc test is used to analyze the data. *P* values are shown on the figure panels.

To determine if **BPN-37440** displays a similar effect on neuroinflammatory mediator mRNA (cytokines, chemokines, COX-2, and mPGES1), we investigated hippocampi from non-SE mice and SE mice treated with either vehicle or **BPN-37440**, 4 days after SE. SE mice displayed higher mRNA levels of selected inflammatory mediators compared to non-SE mice (Supporting Information Figure 5, red-dotted line vs black bars, *P* = 0.0013, two-tailed paired-*t* test). The EP2 antagonist **BPN-37440** treatment caused a mild reduction of these induced inflammatory mRNAs (*P* = 0.033, two-tailed paired-*t* test) (Supporting Information Figure 5), reinforcing the notion that EP2 antagonism quenches SE-induced inflammatory induction in the brain.

### Cognitive Memory Function Tests in Pilocarpine-SE Model

SE in patients is associated with cognitive and psychiatric comorbidities which compromise the quality of life.^29–31^ The currently available anticonvulsant drugs do not mitigate the ensuing behavioral and memory deficits of SE. We asked whether short-term working memory and recognition memory are compromised in pilocarpine-induced SE mice, and if so, will they be rescued by a brief exposure of EP2 antagonist after SE onset. We evaluated a separate cohort of mice treated with vehicle or **BPN-37440**, as shown in Figure 7A, and examined their spatial working memory by using a Y-maze assay at day 28, retention memory using a novel object recognition test between days 29–34, and then finally spatial memory using a Barnes maze test between days 42–47 (Figure 7A).

**Figure 7:**
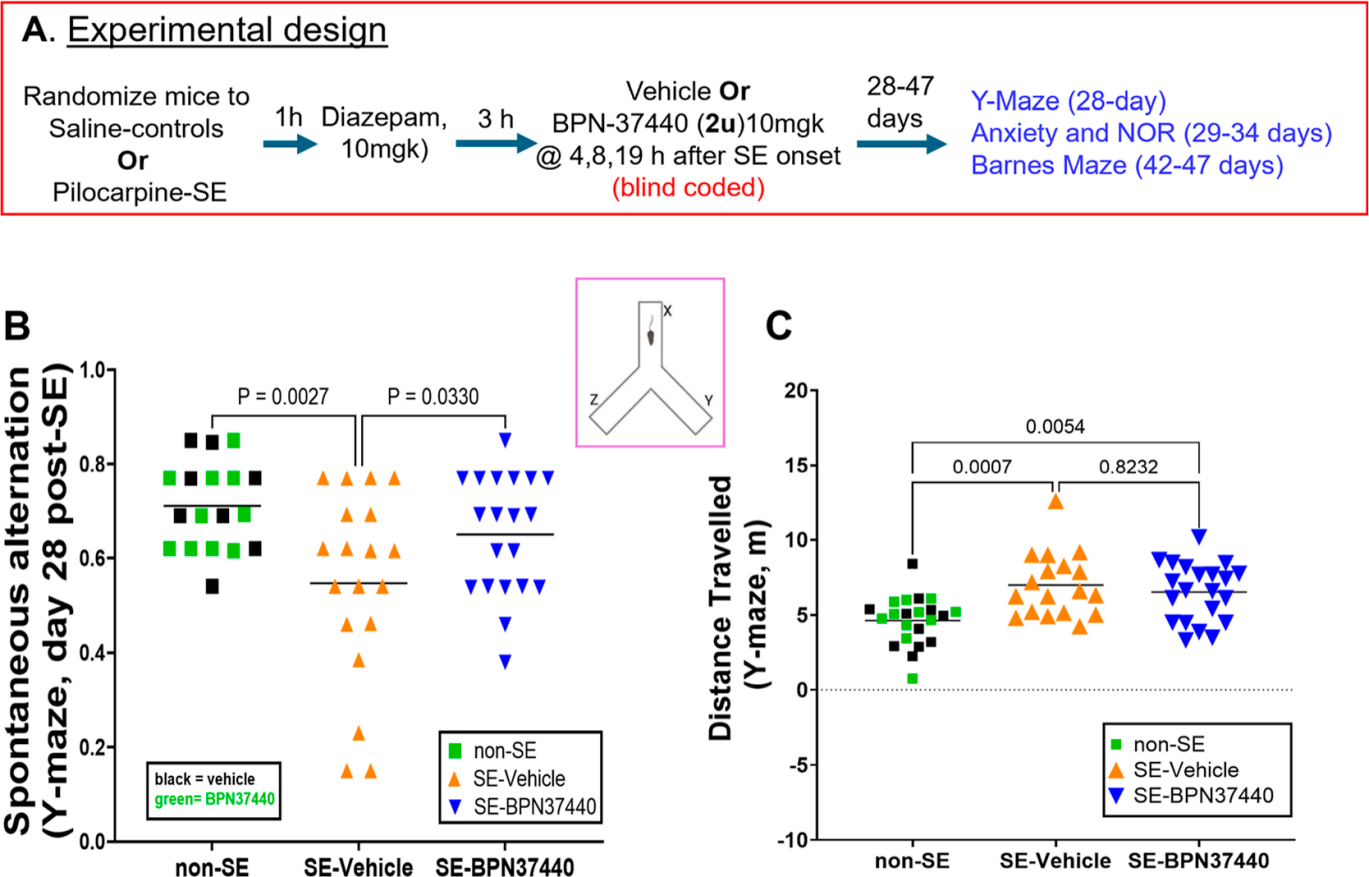
(A) Experimental design for cognitive function tests in non-SE and SE mice treated with EP2 antagonist. The mice used in each group are derived from four different batches of experimental mice to reach *n* = 18–20 per group. In each experiment, mice were randomized first to two groups (non-SE vs pilocarpine-SE), then pilocarpine-SE group was again randomized for vehicle or EP2 antagonist treatment (see Experimental for details). (B) Mice were allowed to explore freely on a Y-maze to determine the spontaneous alternation factor (see Methods). Each symbol is from a different mouse. Mice in non-SE group either treated with vehicle or **BPN-37440** are merged (*n* = 18) for analysis but color coded with black and green squares. (C) Distance traveled by mice w/o SE and w/o **BPN-37440** on the Y-maze is measured. Data between the groups (*n* = 18–20 mice/group) are analyzed by One-way ANOVA followed by Sidak’s multiple comparisons test.

### EP2 Antagonist **BPN-37440** Decreases Working Memory Deficit in Mouse Model of SE

The Y-maze test has been routinely used to assess mice exploratory behavior and short-term working memory in neurological disease models. This does not require any pretraining and involves mice exploring 3 equal-distance arms connected in a Y-shaped apparatus. Rodents typically have an innate tendency to visit the novel arm than the previously visited arm,^34^ and a successful alternation on all three arms (without repeats) reflects mouse spatial working memory. As shown in Figure 7B, non-SE mice showed mean alternation index of 0.71, which is close to a typically expected value (0.7–0.75) for naive (uninjured) mice. The pilocarpine-SE mice, on the other hand, displayed a decreased alternation factor to 0.54 (*P* = 0.0027), indicating a memory deficit (Figure 7B). Interestingly, mice treated with **BPN-37440** displayed an improved alternation factor 0.65 (*P* = 0.033), indicating an improvement in working memory. We and others have repeatedly found that pilocarpine-SE mice are hyperactive, and that their distance traveled is longer than non-SE mice, whether they are treated with either vehicle or drug. The candidate molecule **BPN-37440** did not show any impact on the distance traveled on Y-maze (Figure 7C), further indicating the memory improvement is not related to motor improvement.

### EP2 Antagonist **BPN-37440** Attenuates Retention Memory Deficit in the Pilocarpine Mouse Model of SE

The Novel object recognition (NOR) test provides an efficient assay for assessing recognition memory and cognitive function in rodents with relatively low-stress.^35^ This test is indicative of rodents’ intrinsic preference for exploring a novel object over a familiar object. Successful performance in the NOR test implies that the rodents remember previously encountered objects and therefore can discriminate against the novel object. This evaluation is a model widely used in studies of neurological disease therapies.

As shown in Figure 8A, the same cohort of mice (those which had been tested on the Y-maze) was subjected to an open field (OF) test to determine the impact of SE on anxiety behavior in mice, and to determine the effect of EP2 antagonism on any observed deviations in behavior. In this test, the mice with SE spent less time in center of the open field box compared with non-SE mice, but this effect was not statistically significant. Likewise, EP2 antagonist treated mice showed the reverse trend from the SE-vehicle group, spending less time in the center similar to the non-SE mice, but this impact was also not statistically significant (Supporting Information Figure 6). Overall, the OF test indicated that mice after SE do not have significantly increased anxiety behavior, and therefore no need for an EP2 antagonist to ameliorate it. Following the OF test, two identical objects were introduced to the mice in the same exploration box environment during two familiarization periods 24 h apart. As shown in Figure 8B,C, mice in all treatment groups (non-SE, SE-vehicle or SE-**BPN-37440**) did not show a preference to one of the identical objects. During the recognition test phase, the non-SE control mice spent more time exploring the novel object, whereas the SE-vehicle group showed no preference for the novel object (DI = 0.048, *n* = 19, *P* = 0.293). In contrast, the SE-antagonist group showed a preference for the novel object with a discrimination index of 0.277 (*n* = 20, *P* = < 0.0001, Figure 8D). One sample *t*-test was used for the analysis to determine whether or not the discrimination index among the treatment groups is different from zero, which is anticipated with no memory triggers are evident or when two familiar objects are used (Figure 8B,C versus D). These results suggest that a fleeting exposure of the EP2 antagonist after SE has an enduring beneficial impact on working and recognition memory deficits.

**Figure 8:**
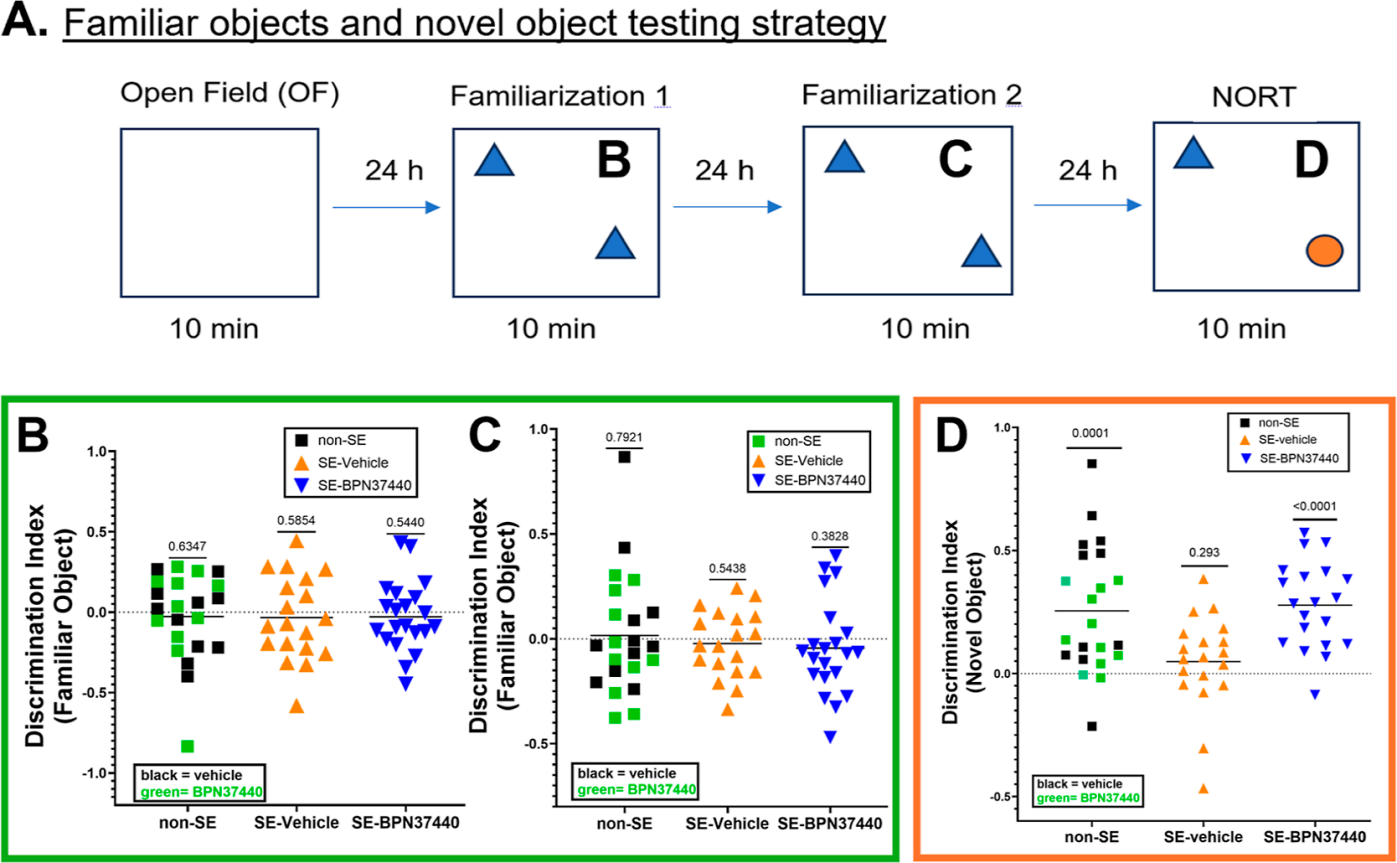
EP2 Antagonist treatment rescues recognition memory following SE in mice. (A) Testing strategy adopted to examine mice for exploring familiar objects and novel object for recognition. (B) Discrimination index against two identical objects for the first time. (C) Discrimination index against the same objects 24 h later (10 min exploration time for each mouse). (D) Discrimination index determined after replacing a familiar object with novel object. The SE mice could not discriminate against the novel object. (p. 0.293, one-sample *t*-test), whereas SE mice treated with **BPN-37440** improved the retention memory with discrimination-index (*P* < 0.0001, one-sample *t*-test), which is equal to the discrimination index exhibited by non-SE mice.

Following the NOR test, the mice were further tested for spatial retention memory deficits between days 42–47 post-SE using the Barnes Maze. The Barnes maze is used to assess spatial learning and memory in rodents and is associated with hippocampal dependent cognitive function.^36^ The rodents’ ability to locate an escape box using visual cues around a circular platform reflects their capacity to acquire, retain, and recall spatial information. As shown in Supporting Information Figure 7, the mice from non-SE control group, or the SE mice with vehicle or the EP2 antagonist treatment all learned to find the escape box using the visual cues in the room over 4 days of trial period (Supporting Information Figure 7A). On day 5, these mice were tested for their ability to remember the location of the escape box and to spend more time in the target quadrant. Non-SE mice (*n* = 19) spent an average of 45 s in the target quadrant during the 90 s exploration time. The SE mice treated with vehicle spent significantly less time (27.34 s, *P* = 0.0003, *n* = 15, one-way ANOVA with Sidak’s posthoc multiple comparison test) in the target quadrant. Interestingly the EP2 antagonist treated mice spent average of 33.93 s (*P* = 0.27, *n* = 18), although not statistically significant, is an improved trend in spatial memory (Supporting Information Figure 7B). Taken together, EP2 antagonism demonstrates a beneficial effect in restoring the working and retention memory following the SE in the pilocarpine-mouse model.

## Discussion

Generalized tonic-clonic seizures and status epilepticus in humans will have long lasting impacts. SE can lead to epilepsy (spontaneous recurring seizures) and can lead to memory and learning dysfunction.^37,38^ In a recent study, nearly 60% of 250 patients who experienced convulsive SE (>85 min) had a poor outcome in 3 months and were unable to work and live independently.^32^ Another study following refractory-SE patients (who are nonresponsive to anticonvulsant drugs) in ICUs also reported similar trends in behavioral deficits.^33^ Therefore, SE-derived cognitive comorbidities can be considered as a major unmet medical need for SE patients, in addition to epileptogenesis in a significant number of patients.^30,31^ Epilepsy patients also develop SE, therefore addressing these cognitive comorbidities with a therapeutic agent will be most valuable to treat these patients, and potentially for others with neurological diseases such as stroke, TBI, AD and PD.

The acute dosing paradigm for EP2 antagonists within 24 h should have a clinical advantage in SE patients such as better adherence compared to longer-duration regimens. Better adherence will translate to better therapeutic outcomes. This also could result in limited drug–drug interactions for the SE patient who is taking antiseizure drugs for days to weeks. Our dosing regimen, to deliver the EP2 antagonist drug with three injections in the first 24 h, is based on the pharmacokinetics of the molecule (Figure 4) and more prominently based on the high induction of neuroinflammatory markers including COX-2 within first 24 h following SE in rodents.^3,39^ Patients also manifest with high neuroinflammatory environment following SE. Therefore, a PET-study examining the time course of neuroinflammation in the ICU will guide the ultimate dosing regimen for human SE patents.

## Conclusion

Our overarching goal is to promote an EP2 antagonist that can mitigate cognitive deficits following SE. We and others have showed the EP2 receptor as a key driver of neuroinflammation and cognitive deficits, and that inhibition of EP2 with a selective antagonist decreases brain inflammation, neurodegeneration, and memory deficits in rodent mdoels.^4,7,39,40^ The novel EP2 antagonist **BPN-37440** reported in this work validates the previous findings and adds additional data indicating working and retention memory is restored following SE. While **BPN-37440** meets several ADME-criteria (Table 3) to evaluate the potential as a preclinical candidate molecule, additional data including dose-range finding (DRF) toxicology, activity against the liver and kidney transporters, and CYP-reaction phenotype assay are still needed to determine the feasibility of **BPN-37440** for later-stage preclinical assessment and eventually for investigation in a clinical setting.

## Experimental Section

### General Experimental Procedures

Chemicals and drugs: PGE_2_, BW245C, iloprost, and rolipram were purchased from Cayman Chemical. LPS (L2880), pilocarpine HCl from (P6503) terbutaline (T2528) and methylscopolamine (S8502) were also purchased form Sigma. Proton NMR spectra were recorded in solvent in DMSO-*d*_6_/CDCl_3_ on either a Bruker Avance 300 or 500 MHz Nuclear Magnetic Resonance spectrometer. NMR data are reported as chemical shift (δ values) [multiplicity, coupling constant (Hertz), integration] using tetramethylsilane as an internal reference. Multiplicity and qualifier abbreviations are as follows: s, singlet; d, doublet; t, triplet; q, quartet; m, multiplet; br, broad. Thin layer chromatography was performed on precoated, aluminum-backed plates (silica gel 60 F_254_, 0.25 mm thickness) and was visualized by UV lamp, PMA solution, and iodine. Column chromatography was performed with prepacked silica gel columns on Teledyne-ISCO automated medium-pressure chromatography system. Low-resolution mass spectroscopic analyses were recorded on Waters Acquity time-of-flight single quadrupole mass spectrometer using electrospray ionization (ESI) in either positive or negative mode. Purity of compounds is determined by Waters Acquity UPLC instrument. UPLC Conditions: Mobile phase A: water (0.1% trifluoroacetic acid); mobile phase B: acetonitrile (0.1% trifluoroacetic acid); column: BEH C18 1.7 μM, 2.1 × 75 mm; gradient: 5% B at 0 min, increased linearly to 100% by 6 min, 100% B for 2 min, then decreased to 5% B by 1 min; UV wavelength = 254 nm; flow rate = 0.5 mL/min. All compounds with >95% purity by UPLC were tested in cellular bioassays and in vitro ADME-PK assays. Compound BPN-37440 with >97% is tested in mouse model of SE.

### Synthesis of Intermediate Compound **8** (See Supporting Information Figure 8A)^16^

#### Step −1: Synthesis of Ethyl 2-((4,6-dimethylpyridin-2-yl)­amino)­pyrimidine-5-carboxylate (**12**)

A stirred solution of compound **10** (5.00 g, 29.9 mmol, 1 equiv), **11** (8.35 g, 44.8 mmol, 1.5 equiv), Cs_2_CO_3_ (19.5 g, 59.8 mmol, 2 equiv), and XantPhos (1.73 g, 2.99 mmol, 0.1 equiv) in 1,4-dioxane (120 mL) was purged with nitrogen for 10 min; and then was added Pd_2_(dba)_3_ (2.74 g, 2.99 mmol, 0.1 equiv) at rt under nitrogen. The reaction mixture was heated at 100 °C overnight. After this time, the reaction mixture was cooled to rt and diluted with CH_2_Cl_2_ (400 mL) and water (100 mL). The solid was removed by vacuum filtration. The layers were separated. The aqueous layer was back-extracted with CH_2_Cl_2_. The combined organics were dried over anhydrous Na_2_SO_4_, filtered, and concentrated under reduced pressure. The resulting residue was purified by flash column chromatography on silica gel eluting with 0–100% EtOAc/hexanes) to provide compound **12** (3.50 g, 43%) as a light-yellow solid: ESI MS *m*/*z*, 273 [M + H]^+^, which was used for next step.

#### Step-2: Synthesis of 2-((4,6-dimethylpyridin-2-yl)­amino)­pyrimidine-5-carboxylic acid (**8**)

To a stirred solution of **12** (3.08 g, 11.3 mmol, 1 equiv) in MeOH (60 mL), THF (60 mL), and water (20 mL) was added LiOH·H_2_O (1.42 g, 33.8 mmol, 3 equiv). The reaction mixture was heated at 60 °C overnight. After this time, the reaction mixture was cooled to rt. The resulting mixture was diluted with water (20 mL) and adjusted pH to ∼5 by addition of 2 N HCl aqueous solution. The solid formed was collected by vacuum filtration. The filter cake was rinsed with water (10 mL) and dried under high vacuum to provide compound **8** (2.05 g, 74%) as a light gray solid: ESI MS *m*/*z*, 245 [M + H]^+^, which was used as it is for next step.

##### Synthesis of **1a–1ii**

(Typical Procedure, Scheme 1) To a stirred solution of acid compound **8** (0.214 mmol, 1 equiv) and Et_3_N (1.07 mmol, 5 equiv) in DMF (4.5 mL) at rt was added amine compound **9** (0.321 mmol, 1.5 equiv), followed by the coupling agent HATU (0.428 mmol, 2 equiv). The reaction mixture was stirred at room temperature overnight. The resulting reaction mixture was quenched with sat. aq. NaHCO_3_ (50 mL). Then reaction mixture was extracted with EtOAc (3 x). The combined extracts were washed with 10% LiCl aq. solution (3 x), dried over anhydrous Na_2_SO_4_, filtered, and concentrated under reduced pressure. The resulting residue was purified by flash column chromatography on silica gel eluting with 0–10% MeOH/CH_2_Cl_2_ to afford final product as an off-white solid.

#### 2-((4,6-Dimethylpyridin-2-yl)­amino)-N-(2-(pyridin-2-yl)­ethyl)­pyrimidine-5-carboxamide (**1a**)

Yield (14 mg, 17%). ^1^H NMR (300 MHz, DMSO-*d*_6_): δ 10.06 (s, 1H), 8.87 (s, 2H), 8.63–8.51 (m, 2H), 7.89 (s, 1H), 7.75–7.70 (m, 1H), 7.31–7.21 (m, 2H), 6.77 (s, 1H), 3.67–3.59 (m, 2H), 3.02–2.98 (m, 2H), 2.36 (s, 3H), 2.29 (s, 3H); ESI MS *m*/*z*, 349 [M + H]^+^; UPLC purity: >99%.

#### 2-((4,6-Dimethylpyridin-2-yl)­amino)-N-(2-(2-methylpyridin-3-yl)­ethyl)­pyrimidine-5-carboxamide (**1b**)

Yield (7 mg, 9%). ^1^H NMR (500 MHz, DMSO-*d*_6_): δ 10.01 (s, 1H), 8.88 (s, 2H), 8.64 (t, *J* = 6.2 Hz, 1H), 8.29 (dd, *J* = 5.4, 2.1 Hz, 1H), 7.90 (s, 1H), 7.53 (dd, *J* = 7.7, 1.8 Hz, 1H), 7.15 (dd, *J* = 7.6, 5.0 Hz, 1H), 6.76 (s, 1H), 3.51–3.45 (m, 2H), 2.87–2.85 (m, 2H), 2.52 (s, 3H), 2.37 (s, 3H), 2.29 (s, 3H); ESI MS *m*/*z*, 363 [M + H]^+^; UPLC purity: 97.4%.

#### 2-((4,6-Dimethylpyridin-2-yl)­amino)-N-(2-(thiazol-4-yl)­ethyl)­pyrimidine-5-carboxamide (**1c**)

Yield (17 mg, 22%). ^1^H NMR (500 MHz, DMSO-*d*_6_): δ 10.01 (s, 1H), 9.05 (d, *J* = 1.9 Hz, 1H), 8.89 (s, 2H), 8.60 (t, *J* = 5.4 Hz, 1H), 7.90 (s, 1H), 7.44–7.43 (m, 1H), 6.77 (s, 1H), 3.62–3.56 (m, 2H), 3.05–3.01 (m, 2H), 2.37 (s, 3H), 2.29 (s, 3H); ESI MS *m*/*z*, 355 [M + H]^+^; UPLC purity: >99%.

#### 2-((4,6-Dimethylpyridin-2-yl)­amino)-N-(2-(4-methylthiazol-5-yl)­ethyl)­pyrimidine-5-carboxamide (**1d**)

Yield (83 mg, 79%). ^1^H NMR (300 MHz, DMSO-*d*_6_): δ 10.05 (s, 1H), 8.92 (s, 2H), 8.84–8.79 (m, 2H), 7.91 (s, 1H), 6.76 (s, 1H), 3.48–3.41 (m, 2H), 3.06–3.01 (m, 2H), 2.37 (s, 3H), 2.33 (s, 3H), 2.30 (s, 3H); ESI MS *m*/*z*, 369 [M + H]^+^; UPLC purity: >99%.

#### 2-((4,6-Dimethylpyridin-2-yl)­amino)-N-(2-(5-methylisoxazol-4-yl)­ethyl)­pyrimidine-5-carboxamide (**1e**)

Yield (50 mg, 66%). ^1^H NMR (500 MHz, DMSO-*d*_6_): δ 10.01 (s, 1H), 8.88 (s, 2H), 8.58 (t, *J* = 5.7 Hz, 1H), 8.40 (s, 1H), 7.91 (s, 1H), 6.76 (s, 1H), 3.44–3.38 (m, 2H), 2.63–2.60 (m, 2H), 2.36 (s, 3H), 2.32 (s, 3H), 2.29 (s, 3H); ESI MS *m*/*z*, 353 [M + H]^+^; UPLC purity: >99%.

#### N-(2-(1H-1,2,3-Triazol-4-yl)­ethyl)-2-((4,6-dimethylpyridin-2-yl)­amino)­pyrimidine-5-carboxamide (**1f**)

Yield (45 mg, 47%). ^1^H NMR (300 MHz, DMSO-*d*_6_): δ 10.04 (s, 1H), 8.88 (s, 2H), 8.64–8.60 (m, 1H), 7.90 (s, 1H), 7.63–7.62 (m, 1H), 6.76 (s, 1H), 3.56–3.49 (m, 2H), 2.94–2.89 (m, 2H), 2.36 (s, 3H), 2.29 (s, 3H); ESI MS *m*/*z*, 339 [M + H]^+^; UPLC purity: 98.1%.

#### 2-((4,6-Dimethylpyridin-2-yl)­amino)-N-(2-(isoxazol-4-yl)­ethyl)­pyrimidine-5-carboxamide (**1g**)

Yield (16 mg, 17%). ^1^H NMR (300 MHz, DMSO-*d*_6_): δ 10.01 (br s, 1H), 8.91 (s, 2H), 8.78 (s, 1H), 8.61 (t, *J* = 5.1 Hz, 1H), 8.55 (s, 1H), 7.88 (s, 1H), 6.79 (s, 1H), 3.50–3.43 (m, 2H), 2.73–2.68 (m, 2H), 2.37 (s, 3H), 2.28 (s, 3H); ESI MS *m*/*z*, 339 [M + H]^+^; UPLC purity: >99%.

#### 2-((4,6-Dimethylpyridin-2-yl)­amino)-N-(2-(pyrimidin-5-yl)­ethyl)­pyrimidine-5-carboxamide (**1h**)

Yield (38 mg, 50%). ^1^H NMR (500 MHz, DMSO-*d*_6_): δ 10.01 (s, 1H), 9.04 (s, 1H), 8.86 (s, 2H), 8.70 (s, 2H), 8.59 (t, *J* = 5.5 Hz, 1H), 7.88 (s, 1H), 6.78 (s, 1H), 3.59–3.53 (m, 2H), 2.90–2.86 (m, 2H), 2.37 (s, 3H), 2.30 (s, 3H); ESI MS *m*/*z*, 350 [M + H]^+^; UPLC purity: 95.1%.

#### 2-((4,6-Dimethylpyridin-2-yl)­amino)-N-(2-(thiazol-5-yl)­ethyl)­pyrimidine-5-carboxamide (**1i**)

Yield (39 mg, 79%). ^1^H NMR (500 MHz, DMSO-*d*_6_): δ 10.02 (s, 1H), 8.94 (d, *J* = 0.8 Hz, 1H), 8.89 (s, 2H), 8.66 (t, *J* = 5.6 Hz, 1H), 7.91 (s, 1H), 7.72 (d, *J* = 0.7 Hz, 1H), 6.77 (s, 1H), 3.54–3.49 (m, 2H), 3.15–3.12 (m, 2H), 2.36 (s, 3H), 2.29 (s, 3H); ESI MS *m*/*z*, 355 [M + H]^+^; UPLC purity: 98.6%.

#### 2-((4,6-Dimethylpyridin-2-yl)­amino)-N-(2-(5-methyl-1H-1,2,3-triazol-4-yl)­ethyl)­pyrimidine-5-carboxamide (**1j**)

Yield (60 mg, 80%). ^1^H NMR (500 MHz, DMSO-*d*_6_): δ 14.22 (s, 1H), 10.02 (s, 1H), 8.89 (s, 2H), 8.61 (t, *J* = 5.5 Hz, 1H), 7.90 (s, 1H), 6.77 (s, 1H), 3.51–3.44 (m, 2H), 2.85–2.82 (m, 2H), 2.37 (s, 3H), 2.30 (s, 3H), 2.19 (s, 3H); ESI MS *m*/*z*, 351 [M–H]^−^; UPLC purity: 98.2%.

#### 2-((4,6-Dimethylpyridin-2-yl)­amino)-N-(2-(pyrazin-2-yl)­ethyl)­pyrimidine-5-carboxamide (**1k**)

Yield (66 mg, 66%). ^1^H NMR (500 MHz, DMSO-*d*_6_): δ 10.00 (s, 1H), 8.86 (s, 2H), 8.62–8.57 (m, 3H), 8.50 (d, *J* = 2.3 Hz, 1H), 7.90 (s, 1H), 6.76 (s, 1H), 3.69–3.62 (m, 2H), 3.06–3.02 (m, 2H), 2.36 (s, 3H), 2.29 (s, 3H); ESI MS *m*/*z*, 350 [M + H]^+^; UPLC purity: >99%.

#### N-(2-(1H-Pyrrol-3-yl)­ethyl)-2-((4,6-dimethylpyridin-2-yl)­amino)­pyrimidine-5-carboxamide (**1l**)

Yield (76 mg, 79%). ^1^H NMR (300 MHz, DMSO-*d*_6_): δ 10.69 (br s, 1H), 9.16 (s, 2H), 8.82–8.78 (m, 1H), 7.97 (br s, 1H), 7.07 (s, 1H), 6.86–6.79 (m, 2H), 6.14–6.11 (m, 1H), 3.63–3.58 (m, 2H), 2.86–2.82 (m, 2H), 2.63 (s, 3H), 2.53 (s, 3H); ESI MS *m*/*z*, 337 [M + H]^+^; UPLC purity: >99%.

#### 2-((4,6-Dimethylpyridin-2-yl)­amino)-N-(2-(5-methyloxazol-4-yl)­ethyl)­pyrimidine-5-carboxamide (**1m**)

Yield (58 mg, 58%). ^1^H NMR (300 MHz, DMSO-*d*_6_): δ 10.04 (s, 1H), 8.88 (s, 2H), 8.62–8.58 (m, 1H), 8.13 (s, 1H), 7.91 (s, 1H), 6.76 (s, 1H), 3.47–3.40 (m, 2H), 2.68–2.64 (m, 2H), 2.36 (s, 3H), 2.29 (s, 3H), 2.21 (s, 3H); ESI MS *m*/*z*, 353 [M + H]^+^; UPLC purity: >99%.

#### 2-((4,6-Dimethylpyridin-2-yl)­amino)-N-(2-(pyridin-3-yl)­ethyl)­pyrimidine-5-carboxamide (**1n**)

Yield (46 mg, 47%). ^1^H NMR (300 MHz, DMSO-*d*_6_): δ 10.06 (s,1H), 8.87 (s, 2H), 8.66–8.62 (m, 1H), 8.47–8.41 (m, 2H), 7.91 (s, 1H), 7.69–7.65 (m, 1H), 7.34–7.30 (m, 1H), 6.76 (s, 1H), 3.55–3.48 (m, 2H), 2.89–2.84 (m, 2H), 2.36 (s, 3H), 2.29 (s, 3H); ESI MS *m*/*z*, 349 [M + H]^+^; UPLC purity: 97.9%.

#### 2-((4,6-Dimethylpyridin-2-yl)­amino)-N-(2-(5-methyl-1H-imidazole-4-yl)­ethyl)­pyrimidine-5-carboxamide (**1o**)

Yield (7 mg, 7%). ^1^H NMR (500 MHz, DMSO-*d*_6_): δ 9.99 (s, 1H), 8.88 (s, 2H), 8.59–8.56 (m, 1H), 7.90 (s, 1H), 7.71 (br s, 1H), 6.76 (s, 1H), 3.44–3.39 (m, 2H), 2.73–2.70 (m, 2H), 2.36 (s, 3H), 2.29 (s, 3H), 2.09 (s, 3H); ESI MS *m*/*z*, 352 [M + H]^+^; UPLC purity: 95.0%.

#### 2-((4,6-Dimethylpyridin-2-yl)­amino)-N-(2-(4-methyl-2-phenylthiazol-5-yl)­ethyl)­pyrimidine-5-carboxamide (**1p**)

Yield (64 mg, 44%). ^1^H NMR (500 MHz, DMSO-*d*_6_): δ 10.03 (s, 1H), 8.90 (s, 2H), 8.73–8.68 (m, 1H), 7.90 (s, 1H), 7.87–7.83 (m, 2H), 7.49–7.41 (m, 3H), 6.77 (s, 1H), 3.53–3.46 (m, 2H), 3.09–3.03 (m, 2H), 2.36 (s, 3H), 2.35 (s, 3H), 2.29 (s, 3H); ESI MS *m*/*z*, 445 [M + H]^+^; UPLC purity: 97.3%.

#### 2-((4,6-Dimethylpyridin-2-yl)­amino)-N-(2-(2-methylbenzofuran-3-yl)­ethyl)­pyrimidine-5-carboxamide (**1q**)

Yield (78 mg, 68%). ^1^H NMR (500 MHz, DMSO-*d*_6_): δ 10.00 (s, 1H), 8.87 (s, 2H), 8.65 (t, *J* = 5.7 Hz, 1H), 7.90 (s, 1H), 7.59–7.56 (m, 1H), 7.46–7.43 (m, 1H), 7.22–7.18 (m, 2H), 6.76 (s, 1H), 3.52–3.46 (m, 2H), 2.90–2.86 (m, 2H), 2.36 (s, 6H), 2.29 (s, 3H); ESI MS *m*/*z*, 402 [M + H]^+^; UPLC purity: 97.5%.

#### N-(2-(Benzo­[*d*]­thiazol-2-yl)­ethyl)-2-((4,6-dimethylpyridin-2-yl)­amino)­pyrimidine-5-carboxamide (**1r**)

Yield (72 mg, 63%). ^1^H NMR (500 MHz, DMSO-*d*_6_): δ 10.00 (s, 1H), 8.91 (s, 2H), 8.85–8.81 (m, 1H), 8.06 (d, *J* = 8.0 Hz, 1H), 7.95 (d, *J* = 8.0 Hz, 1H), 7.89 (s, 1H), 7.51–7.47 (m, 1H), 7.42–7.39 (m, 1H), 6.76 (s, 1H), 3.75–3.71 (m, 2H), 3.40–3.38 (m, 2H), 2.36 (s, 3H), 2.29 (s, 3H); ESI MS *m*/*z*, 405 [M + H]^+^; UPLC purity: 98.8%.

#### 2-((4,6-Dimethylpyridin-2-yl)­amino)-N-(2-(3-methylpyrazin-2-yl)­ethyl)­pyrimidine-5-carboxamide (**1s**)

Yield (77 mg, 27%). ^1^H NMR (500 MHz, DMSO-*d*_6_): δ 10.07 (br s, 1H), 8.88 (s, 1H), 8.86 (s, 2H), 8.64 (t, *J* = 5.6 Hz, 1H), 8.48 (s, 1H), 7.89 (s, 1H), 6.77 (s, 1H), 3.30–3.22 (m, 2H), 2.90–2.85 (m, 2H), 2.52 (s, 3H), 2.37 (s, 3H), 2.30 (s, 3H); ESI MS *m*/*z*, 364 [M + H]^+^; UPLC purity: 97.2%.

#### 2-((4,6-Dimethylpyridin-2-yl)­amino)-N-(2-(2-methylbenzo­[*b*]­thiophen-3-yl)­ethyl)­pyrimidine-5-carboxamide (**1t**)

Yield (78 mg, 68%). ^1^H NMR (500 MHz, DMSO-*d*_6_): δ 10.01 (s, 1H), 8.90 (s, 2H), 8.78 (t, *J* = 5.7 Hz, 1H), 7.91 (s, 1H), 7.86 (d, *J* = 8.0 Hz, 1H), 7.82 (d, *J* = 7.5 Hz, 1H), 7.37 (t, *J* = 7.5 Hz, 1H), 7.29 (t, *J* = 7.5 Hz, 1H), 6.77 (s, 1H), 3.48–3.41 (m, 2H), 3.07–3.03 (m, 2H), 2.49 (s, 3H), 2.37 (s, 3H), 2.30 (s, 3H); ESI MS *m*/*z*, 418 [M + H]^+^; UPLC purity: 95.9%.

#### N-(2-(1H-Benzo­[d]­imidazole-2-yl)­ethyl)-2-((4,6-dimethylpyridin-2-yl)­amino)­pyrimidine-5-carboxamide (**1u**)

Yield (12 mg, 14%). ^1^H NMR (500 MHz, DMSO-*d*_6_): δ 12.36 (br s, 1H), 10.00 (s, 1H), 8.89 (s, 2H), 8.71–8.68 (m, 1H), 7.89 (s, 1H), 7.49 (br s, 2H), 7.14–7.12 (m, 2H), 6.76 (s, 1H), 3.74–3.70 (m, 2H), 3.13–3.08 (m, 2H), 2.36 (s, 3H), 2.28 (s, 3H); ESI MS *m*/*z*, 388 [M + H]^+^; UPLC purity: 95.0%

#### 2-((4,6-Dimethylpyridin-2-yl)­amino)-N-(2-(3-methyl-1H-indol-2-yl)­ethyl)­pyrimidine-5-carboxamide (**1v**)

Yield (22 mg, 42%). ^1^H NMR (500 MHz, DMSO-*d*_6_): δ 10.74 (s, 1H), 10.00 (s, 1H), 8.89 (s, 2H), 8.63 (t, *J* = 5.7 Hz, 1H), 7.90 (s, 1H), 7.37 (d, *J* = 7.6 Hz, 1H), 7.25 (d, *J* = 8.2 Hz, 1H), 7.02–6.97 (m, 1H), 6.95–6.90 (m, 1H), 6.76 (s, 1H), 3.57–3.50 (m, 2H), 2.98–2.93 (m, 2H), 2.36 (s, 3H), 2.29 (s, 3H), 2.17 (s, 3H); ESI MS *m*/*z*, 399 [M–H]^−^; UPLC purity: 95.6%.

#### 2-((4,6-Dimethylpyridin-2-yl)­amino)-N-(2-(2-methylquinolin-4-yl)­ethyl)­pyrimidine-5-carboxamide (**1w**)

Yield (27 mg, 24%). ^1^H NMR (300 MHz, DMSO-*d*_6_): δ 10.06 (s, 1H), 8.97–8.92 (m, 3H), 8.25 (d, *J* = 8.1 Hz, 1H), 7.96–7.91 (m, 2H), 7.73–7.61 (m, 1H), 7.59–7.54 (m, 1H), 7.33 (s, 1H), 6.77 (s, 1H), 3.65–3.58 (m, 2H), 3.33–3.29 (m, 2H), 2.61 (s, 3H), 2.37 (s, 3H), 2.28 (s, 3H); ESI MS *m*/*z*, 413 [M + H]^+^; UPLC purity: 97.2%.

#### 2-((4,6-Dimethylpyridin-2-yl)­amino)-N-(2-(2-methylquinazolin-4-yl)­ethyl)­pyrimidine-5-carboxamide (**1x**)

Yield (29 mg, 13%). ^1^H NMR (500 MHz, DMSO-*d*_6_): δ 10.01 (s, 1H), 8.83 (s, 2H), 8.65 (t, *J* = 5.5 Hz, 1H), 8.30 (d, *J* = 8.6 Hz, 1H), 7.95–7.88 (m, 3H), 7.67–7.63 (m, 1H), 6.76 (s, 1H), 3.80–3.72 (m, 2H), 3.53–3.48 (m, 2H), 2.73 (s, 3H), 2.36 (s, 3H), 2.29 (s, 3H); ESI MS *m*/*z*, 414 [M + H]^+^; UPLC purity: 96.0%.

#### 2-((4,6-Dimethylpyridin-2-yl)­amino)-N-(2-(2-ethyl-1H-indol-3-yl)­ethyl)­pyrimidine-5-carboxamide (**1y**)

Yield (58 mg, 64%). ^1^H NMR (500 MHz, DMSO-*d*_6_): δ 10.72 (s, 1H), 10.00 (s, 1H), 8.90 (s, 2H), 8.67–8.62 (m, 1H), 7.90 (s, 1H), 7.49 (d, *J* = 8.0 Hz, 1H), 7.25 (d, *J* = 8.5 Hz, 1H), 7.01–6.91 (m, 2H), 6.77 (s, 1H), 3.45–3.38 (m, 2H), 2.93–2.87 (m, 2H), 2.70 (q, *J* = 7.5 Hz, 2H), 2.37 (s, 3H), 2.30 (s, 3H), 1.22 (t, *J* = 7.5 Hz, 3H); ESI MS *m*/*z*, 415 [M + H]^+^; UPLC purity: 95.5%.

#### N-(2-(1,2-Dimethyl-1H-indol-3-yl)­ethyl)-2-((4,6-dimethylpyridin-2-yl)­amino)­pyrimidine-5-carboxamide (**1z**)

Yield (67 mg, 50%). ^1^H NMR (500 MHz, DMSO-*d*_6_): δ 9.99 (s, 1H), 8.88 (s, 2H), 8.63–8.61 (m, 1H), 7.90 (s, 1H), 7.50 (d, *J* = 8.0 Hz, 1H), 7.34 (d, *J* = 8.0 Hz, 1H), 7.07–7.03 (m, 1H), 6.98–6.95 (m, 1H), 6.76 (s, 1H), 3.64 (s, 3H), 3.41–3.37 (m, 2H), 2.94–2.91 (m, 2H), 2.36 (s, 3H), 2.34 (s, 3H), 2.29 (s, 3H); ESI MS *m*/*z*, 415 [M + H]^+^; UPLC purity: >99%.

#### 2-((4,6-Dimethylpyridin-2-yl)­amino)-N-(2-(2-methyl-1H-benzo­[*d*]­imidazole-1-yl)­ethyl)­pyrimidine-5-carboxamide (**1aa**)

Yield (89 mg, 68%). ^1^H NMR (500 MHz, DMSO-*d*_6_): δ 10.02 (s, 1H), 8.80–8.78 (m, 3H), 7.87 (s, 1H), 7.51–7.49 (m, 2H), 7.14–7.11 (m, 2H), 6.76 (s, 1H), 4.40–4.32 (m, 2H), 3.64–3.60 (m, 2H), 2.50 (s, 3H), 2.36 (s, 3H), 2.28 (s, 3H); ESI MS *m*/*z*, 402 [M + H]^+^; UPLC purity: 98.4%.

#### 2-((4,6-Dimethylpyridin-2-yl)­amino)-N-(2-(2-methyl-1H-indol-4-yl)­ethyl)­pyrimidine-5-carboxamide (**1bb**)

Yield (30 mg, 75%). ^1^H NMR (500 MHz, DMSO-*d*_6_): δ 10.87 (s, 1H), 10.01 (s, 1H), 8.90 (s, 2H), 8.62 (t, *J* = 5.6 Hz, 1H), 7.91 (s, 1H), 7.12 (d, *J* = 8.0 Hz, 1H), 6.91 (t, *J* = 7.3 Hz, 1H), 6.79–6.76 (m, 2H), 6.25 (s, 1H), 3.56–3.52 (m, 2H), 3.03–3.00 (m, 2H), 2.38 (s, 3H), 2.37 (s, 3H), 2.30 (s, 3H); ESI MS *m*/*z*, 401 [M + H]^+^; UPLC purity: 95.3%.

#### 2-((4,6-Dimethylpyridin-2-yl)­amino)-N-(2-(2-methyl-1H-indol-7-yl)­ethyl)­pyrimidine-5-carboxamide (**1cc**)

Yield (14 mg, 9%). ^1^H NMR (500 MHz, DMSO-*d*_6_): δ 11.81 (s, 1H), 11.61 (s, 1H), 10.01 (s, 1H), 8.87 (s, 2H), 8.57 (t, *J* = 5.4 Hz, 1H), 7.90 (s, 1H), 7.80 (d, *J* = 7.8 Hz, 1H), 7.03–7.00 (m, 1H), 6.95 (d, *J* = 7.0 Hz, 1H), 6.76 (s, 1H), 3.62–3.58 (m, 2H), 3.13–3.10 (m, 2H), 2.66 (s, 3H), 2.36 (s, 3H), 2.29 (s, 3H); ESI MS *m*/*z*, 399 [M–H]^−^; UPLC purity: 95.4%.

#### 2-((4,6-Dimethylpyridin-2-yl)­amino)-N-(2-(2-methyl-1H-pyrrolo­[2,3-*b*]­pyridin-3-yl)­ethyl)­pyrimidine-5-carboxamide (**1dd**)

Yield (41 mg, 45%). ^1^H NMR (500 MHz, DMSO-*d*_6_): δ 11.28 (s, 1H), 10.20 (br s, 1H), 8.89 (s, 2H), 8.63 (t, *J* = 5.3 Hz, 1H), 8.07 (d, *J* = 4.1 Hz, 1H), 7.89–7.83 (m, 2H), 6.99–6.96 (m, 1H), 6.81 (s, 1H), 3.45–3.40 (m, 2H), 2.91–2.88 (m, 2H), 2.39 (s, 3H), 2.33 (s, 3H), 2.31 (s, 3H); ESI MS *m*/*z*, 402 [M + H]^+^; UPLC purity: 96.0%.

#### 2-((4,6-Dimethylpyridin-2-yl)­amino)-N-(2-(2-methyl-1H-pyrrolo­[3,2-*c*]­pyridin-3-yl)­ethyl)­pyrimidine-5-carboxamide (**1ee**)

Yield (15 mg, 11%). ^1^H NMR (500 MHz, DMSO-*d*_6_): δ 11.17 (s, 1H), 10.00 (s, 1H), 8.87 (s, 2H), 8.74 (s, 1H), 8.63 (t, *J* = 5.5 Hz, 1H), 8.06 (d, *J* = 6.0 Hz, 1H), 7.90 (s, 1H), 7.23–7.19 (m, 1H), 6.76 (s, 1H), 3.49–3.42 (m, 2H), 2.96–2.92 (m, 2H), 2.37 (s, 3H), 2.33 (s, 3H), 2.30 (s, 3H); ESI MS *m*/*z*, 400 [M–H]^−^; UPLC purity: 98.5%.

#### 2-((4,6-Dimethylpyridin-2-yl)­amino)-N-(2-(2-methyl-1H-pyrrolo­[3,2-*b*]­pyridin-3-yl)­ethyl)­pyrimidine-5-carboxamide (**1ff**)

Yield (16 mg, 19%). ^1^H NMR (500 MHz, CD_3_OD): δ 9.08 (s, 2H), 8.44–8.41 (m, 1H), 8.36–8.32 (m, 1H), 7.56–7.52 (m, 1H), 7.37 (br s, 1H), 7.14 (s, 1H), 3.70–3.65 (m, 2H), 3.20–3.16 (m, 2H), 2.68 (s, 3H), 2.61 (s, 3H), 2.51 (s, 3H); ESI MS *m*/*z*, 402 [M + H]^+^; UPLC purity: 96.3%.

#### 2-((4,6-Dimethylpyridin-2-yl)­amino)-N-(2-(5-hydroxy-2-methyl-1H-indol-3-yl)­ethyl)­pyrimidine-5-carboxamide (**1gg**)

Yield (24 mg, 14%). ^1^H NMR (500 MHz, DMSO-*d*_6_): δ 10.38 (s, 1H), 10.04 (s, 1H), 8.91 (s, 2H), 8.63 (t, *J* = 5.7 Hz, 1H), 8.47 (s, 1H), 7.90 (s, 1H), 7.01 (d, *J* = 8.5 Hz, 1H), 6.78 (d, *J* = 9.0 Hz, 2H), 6.49 (dd, *J* = 8.5, 2.3 Hz, 1H), 3.39–3.35 (m, 2H), 2.82–2.77 (m, 2H), 2.37 (s, 3H), 2.30 (s, 3H), 2.27 (s, 3H); ESI MS *m*/*z*, 417 [M + H]^+^; UPLC purity: 95.1%.

#### N-(2-(4,7-Difluoro-2-methyl-1H-indol-3-yl)­ethyl)-2-((4,6-dimethylpyridin-2-yl)­amino)­pyrimidine-5-carboxamide (**1hh**)

Yield (20 mg, 22%). ^1^H NMR (500 MHz, DMSO-*d*_6_): δ 11.41 (s, 1H), 9.93 (s, 1H), 8.80 (s, 2H), 8.52 (t, *J* = 5.7 Hz, 1H), 7.84 (s, 1H), 6.72–6.66 (m, 2H), 6.59–6.56 (m, 1H), 3.39–3.35 (m, 2H), 2.88–2.85 (m, 2H), 2.30 (s, 3H), 2.23 (s, 6H); ESI MS *m*/*z*, 437 [M + H]^+^; UPLC purity: 96.8%.

#### 2-((4,6-Dimethylpyridin-2-yl)­amino)-N-(2-(7-fluoro-2,4-dimethyl-1H-indol-3-yl)­ethyl)­pyrimidine-5-carboxamide (**1ii**)

Yield (11 mg, 6%). ^1^H NMR (500 MHz, DMSO-*d*_6_): δ 11.16 (s, 1H), 10.00 (s, 1H), 8.91 (s, 2H), 8.71–8.69 (m, 1H), 7.91 (s, 1H), 6.76 (s, 1H), 6.68–6.59 (m, 2H), 3.39–3.35 (m, 2H), 3.01–2.97 (m, 2H), 2.62 (s, 3H), 2.36 (s, 3H), 2.32 (s, 3H), 2.29 (s, 3H); ESI MS *m*/*z*, 433 [M + H]^+^;UPLC purity: 96.1%.

### Synthesis of Intermediate Compound **15** (See Supporting Information Figure 8B)

#### Step-1: Synthesis of Methyl 5-methoxy-1-methyl-1H-pyrrolo­[2,3-*c*]­pyridine-2-carboxylate (**14**)

To a stirred solution of compound **13** (1.00 g, 5.20 mmol, 1 equiv) in DMSO (25 mL) at rt was added KOH (874 mg, 15.6 mmol, 3 equiv), followed by MeI (1.62 g, 11.4 mmol, 2.2 equiv). The reaction mixture was stirred at rt for 2 h. After this time, the resulting reaction mixture was added water (200 mL). The solid formed was collected by vacuum filtration. The filter cake was rinsed with water (10 mL) and dried under high vacuum in a 50 °C oven to provide compound **14** as an off-white solid (784 mg, 57%): ESI MS *m*/*z*, 221 [M + H]^+^.

#### Step-2: Synthesis of 5-methoxy-1-methyl-1H-pyrrolo­[2,3-*c*]­pyridine-2-carboxylic acid (**15**)

To a stirred solution of compound **14** (360 mg, 1.64 mmol, 1 equiv) in MeOH (15 mL), THF (15 mL), and water (5 mL) at rt was added LiOH·H_2_O (206 mg, 4.91 mmol, 3 equiv). The reaction mixture was stirred at 40 °C for overnight. After this time, the resulting reaction mixture was cooled to rt and concentrated under reduced pressure. The resulting residue was dissolved in water (10 mL) and adjusted pH to ∼4 by addition of 2 N HCl aqueous solution. The solid formed was collected by vacuum filtration. The filter cake was dried under high vacuum in a 50 °C oven to provide compound **15** as an off-white solid (321 mg, 95%): ESI MS *m*/*z*, = 207 [M + H]^+^, which is used for next as it is.

### Synthesis of Intermediate Compound **19** (See Supporting Information Figure 8C)

#### Step-1: Synthesis of Ethyl 7-chloro-1-methyl-1H-pyrrolo­[2,3-*c*]­pyridine-2-carboxylate (**17**)

To a stirred solution of compound **16** (16.8 g, 74.6 mmol, 1 equiv) in DMF (200 mL) was added Cs_2_CO_3_ (36.5 g, 112 mmol, 1.5 equiv) followed by CH_3_I (13.7 g, 96.3 mmol, 1.3 equiv) at rt. The reaction mixture was stirred at rt for 3 h. After this time, the reaction mixture was cooled with an ice/water bath and diluted with water (500 mL). The precipitated solid was collected by vacuum filtration. The filter cake was rinsed with water (10 mL), dried under high vacuum at 50 °C oven to afford compound **17** (16.7 g, 94%) as a light-yellow solid: ESI MS *m*/*z*, 239 [M + H]^+^.

#### Step-2: Synthesis of ethyl 1,7-dimethyl-1H-pyrrolo­[2,3-c]­pyridine-2-carboxylate (**18**)

An oven-dried flask was charged with compound **17** (8.00 g, 33.5 mmol, 1 equiv) and dissolved in anhydrous THF (160 mL) at rt. The resulting solution was added NMP (33.2 g, 335 mmol, 10 equiv) followed by iron­(lll) acetylacetonate (591 mg, 1.68 mmol, 0.05 equiv) under nitrogen. The resulting dark-red solution was cooled to 0 °C and a solution of MeMgCl in THF (3 M, 12.3 mL, 36.9 mmol, 1.1 equiv) was added dropwise and stirred at 0 °C for 5 min. After this time, the reaction mixture was quenched with water (20 mL) at 0 °C. The resulting suspension was warmed to rt, diluted with ethyl acetate (600 mL), washed with water (200 mL) and brine (100 mL). The organics were dried over anhydrous Na_2_SO_4_, filtered, and concentrated under reduced pressure. The resulting residue was purified by flash column chromatography on silica gel eluting with 0–50% EtOAc/hexane) to provide compound **18** (7.00 g, 95%) as a yellow solid: ESI MS *m*/*z*, 219 [M + H]^+^.

#### Step-3: Synthesis of 1,7-dimethyl-1H-pyrrolo­[2,3-*c*]­pyridine-2-carboxylic acid (**19**)

To a stirred solution of compound **18** (7.00 g, 32.1 mmol, 1 equiv) in MeOH (14 mL), THF (14 mL), and water (4 mL) was added LiOH·H_2_O (4.04 g, 96.2 mmol, 3 equiv) at rt. The reaction mixture was then heated at 40 °C for 2 h. After this time, the reaction mixture was cooled to 0 °C and adjusted pH to ∼4 by addition of 6 N HCl aqueous solution. The solid formed was collected by vacuum filtration. The filter cake was dried under high vacuum in a 50 °C oven to provide compound **19** (5.78 g, 95%) as a white solid: ^1^H NMR (500 MHz, DMSO-*d*_6_): δ 14.76 (br s, 1H), 8.15 (d, *J* = 6.3 Hz, 1H), 7.97 (d, *J* = 6.3 Hz, 1H), 7.42 (s, 1H), 4.40 (s, 3H), 3.21 (s, 3H). ESI MS *m*/*z*, 191 [M + H]^+^.

### Synthesis of Intermediate Compound **24** (See Supporting Information Figure 8D)

#### Step-1: Synthesis of 2-(2-methoxyethoxy)-4-methyl-5-nitropyridine (**21**)

To a stirred mixture of compound **20** (1.00 g, 5.79 mmol, 1 equiv) and 2-methoxy ethanol (661 mg, 8.69 mmol, 1.5 equiv) was added NaH (60%, 348 mg, 8.70 mmol, 1.5 equiv) at rt under nitrogen. The reaction mixture was then heated at 80 °C for 2 h. After this time, the reaction mixture was cooled to rt and quenched with MeOH (1 mL). The resulting mixture was diluted with EtOAc, dried over anhydrous Na_2_SO_4_, filtered, and concentrated under reduced pressure. The resulting residue was purified by flash column chromatography on silica gel eluting with 0–100% EtOAc/hexanes) to provide compound **21** (507 mg, 41%) as an off-white solid: ESI MS *m*/*z*, 213 [M + H]^+^.

#### Step-2: Synthesis of ethyl 5-(2-methoxyethoxy)-1H-pyrrolo­[2,3-*c*]­pyridine-2-carboxylate (**22**)

To a stirred mixture of compound **21** (507 mg, 2.38 mmol, 1 equiv) and diethyl oxalate (1.50 g, 9.09 mmol, 4.3 equiv) was added DBU (836 mg, 5.49 mmol, 2.3 equiv) at rt under nitrogen. The reaction mixture was then stirred at rt overnight. After this time, the reaction mixture was concentrated to dryness. The resulting residue was dissolved in HOAc (22 mL) and Fe (266 mg, 4.76 mmol, 2 equiv) was added at rt under nitrogen. The reaction mixture was then heated at 80 °C for 5 h. After this time, the reaction mixture was cooled to rt and diluted with water (50 mL). The resulting mixture was extracted with EtOAc (100 mL). The extracts were dried over anhydrous Na_2_SO_4_, filtered, and concentrated under reduced pressure. The resulting residue was purified by flash column chromatography on silica gel eluting with 0–100% EtOAc/hexanes) to provide compound **22** (225 mg, 36%) as an off-white solid: ESI MS *m*/*z*, 265 [M + H]^+^.

#### Step-3: Synthesis of ethyl 5-(2-methoxyethoxy)-1-methyl-1H-pyrrolo­[2,3-*c*]­pyridine-2-carboxylate (**23**)

To a stirred solution of compound **22** (225 mg, 0.851 mmol, 1 equiv) and MeI (265 mg, 1.87 mmol, 2.2 equiv) in DMSO (3 mL) was added KOH (143 mg, 2.55 mmol, 3 equiv) at rt under nitrogen. The reaction mixture was stirred at rt for 20 min. After this time, the reaction mixture was diluted with water (50 mL). The solid formed was collected by vacuum filtration. The filter cake was washed with water and dried under high vacuum to provide compound **23** (191 mg, 81%) as a white solid: ESI MS *m*/*z*, 279 [M + H]^+^.

#### Step-4: Synthesis of 5-(2-methoxyethoxy)-1-methyl-1H-pyrrolo­[2,3-*c*]­pyridine-2-carboxylic acid (**24**)

To a stirred solution of compound **23** (191 mg, 0.686 mmol, 1 equiv) in MeOH (9 mL), THF (6 mL), and water (1 mL) was added LiOH·H_2_O (86 mg, 2.05 mmol, 3 equiv) at rt. The reaction mixture was stirred at rt for 2 h. After this time, the reaction mixture was diluted with water (20 mL) and concentrated to ∼20 mL remained. The resulting solution was adjusted pH to ∼5 by addition of 2 N HCl aqueous solution. The solid formed was collected by vacuum filtration. The filter cake was rinsed with water (10 mL) and dried under high vacuum to provide compound **24** (155 mg, 90%) as an off-white solid: ESI MS *m*/*z*, 251 [M + H]^+^.

### Synthesis of Intermediate Compound **27** (See Supporting Information Figure 8E)

#### Step-1: Synthesis of Methyl 5-methoxy-1-methyl-1H-pyrrolo­[2,3-*b*]­pyridine-2-carboxylate (**26**)

To a stirred solution of compound **25** (100 mg, 0.485 mmol, 1 equiv) and MeI (76 mg, 0.535 mmol, 1.1 equiv) in anhydrous DMF (5 mL) was added NaH (60%, 22 mg, 0.550 mmol, 1.1 equiv) at rt under nitrogen. The reaction mixture was stirred at rt overnight. After this time, the reaction mixture was quenched with water (20 mL) and extracted with EtOAc (80 mL). The extracts were dried over anhydrous Na_2_SO_4_, filtered, and concentrated under reduced pressure. The resulting residue was purified by flash column chromatography on silica gel eluting with 0–100% EtOAc/hexanes) to provide compound **26** (96 mg, 89%) as an orange solid: ESI MS *m*/*z*, 221 [M + H]^+^.

#### Step-2: Synthesis of 5-methoxy-1-methyl-1H-pyrrolo­[2,3-b]­pyridine-2-carboxylic acid (**27**)

To a stirred solution of compound **26** (95 mg, 0.432 mmol, 1 equiv) in MeOH (2 mL), THF (7 mL), and water (2 mL) was added LiOH·H_2_O (145 mg, 3.45 mmol, 8 equiv) at rt. The reaction mixture was stirred at rt for 2 h. After this time, the reaction mixture was diluted with water (20 mL) and concentrated to ∼20 mL remained. The resulting solution was adjusted pH to ∼5 by addition of 2 N HCl aqueous solution. The solid formed was collected by vacuum filtration. The filter cake was rinsed with water (10 mL) and dried under high vacuum to provide compound **27** (75 mg, 84%) as a pink solid: ESI MS *m*/*z*, 207 [M + H]^+^.

##### Synthesis of **2a–2bb**

(Typical procedure, Scheme 2): To a stirred solution of acid compounds, for example **15, 19, 24** or **27** (Supporting Information Figure 8) (Markush structures **4–7** in Scheme 2) (1.26 mmol, 1 equiv) and Et_3_N (3.81 mmol, 3 equiv) in DMF (10 mL) at rt was added amine **3** (Scheme 2) (1.89 mmol, 1.5 equiv), followed by HATU (1.89 mmol, 1.5 equiv). The reaction mixture was stirred at rt overnight. After this time, the resulting reaction mixture was quenched with sat. aq. NaHCO_3_ (50 mL). The resulting mixture was extracted with EtOAc (3 x). The combined extracts were washed with 10% LiCl aqueous solution (3 x), dried over anhydrous Na_2_SO_4_, filtered, and concentrated under reduced pressure. The resulting residue was purified by flash column chromatography on silica gel eluting with 50–80% EtOAc/CH_2_Cl_2_ to afford compound **2** (**a–bb**) as off-white solid.

#### 5-Methoxy-N-(2-(2-methyl-1H-indol-3-yl)­ethyl)-1H-indole-2-carboxamide (**2a**)

Yield (86 mg, 47%). ^1^H NMR (500 MHz, DMSO-*d*_6_): δ 11.38 (s, 1H), 10.70 (s, 1H), 8.51 (t, *J* = 7.5 Hz, 1H), 7.50 (d, *J* = 10.0 Hz, 1H), 7.31 (d, *J* = 15.0 Hz, 1H), 7.23 (d, *J* = 10.0 Hz, 1H), 7.07 (d, *J* = 5.0 Hz, 1H), 6.99–6.95 (m, 2H), 6.94–6.91 (m, 1H), 6.82 (dd, *J* = 10.0, 5.0 Hz, 1H), 3.76 (s, 3H), 3.46–3.40 (m, 2H), 2.92–2.88 (m, 2H), 2.31 (s, 3H). ESI MS *m*/*z*, 348 [M + H]^+^; UPLC purity: >99%.

#### 5-Methoxy-1-methyl-N-(2-(2-methyl-1H-indol-3-yl)­ethyl)-1H-indole-2-carboxamide (**2b**)

Yield (130 mg, 37%). ^1^H NMR (500 MHz, DMSO-*d*_6_): δ 10.70 (s, 1H), 8.51–8.48 (m, 1H), 7.49 (d, *J* = 10.0 Hz, 1H), 7.41 (d, *J* = 9.0 Hz, 1H), 7.22 (d, *J* = 8.0 Hz, 1H), 7.08 (d, *J* = 2.0 Hz, 1H), 6.99–6.93 (m, 2H), 6.91–6.89 (m, 2H), 3.95 (s, 3H), 3.77 (s, 3H), 3.42–3.38 (m, 2H), 2.93–2.87 (m, 2H), 2.00 (s, 3H). ESI MS, *m*/*z* = 362 [M + H]^+^; UPLC purity: 98.6%.

##### N-(2-(2-Methyl-1H-indol-3-yl)­ethyl)-1H-pyrrolo­[2,3-*c*]­pyridine-2-carboxamide (**2c**)

Yield (45 mg, 23%). ^1^H NMR (500 MHz, DMSO-*d*_6_): δ 12.09 (s, 1H), 10.71 (s, 1H), 8.83 (t, *J* = 5.9 Hz, 1H), 8.79 (s, 1H), 8.13 (d, *J* = 5.5 Hz, 1H), 7.62 (d, *J* = 5.5 Hz, 1H), 7.49 (d, *J* = 7.7 Hz, 1H), 7.22 (d, *J* = 7.7 Hz, 1H), 7.10 (s, 1H), 6.99–6.91 (m, 2H), 3.49–3.43 (m, 2H), 2.94–2.90 (m, 2H), 2.31 (s, 3H). ESI MS *m*/*z*, 319 [M + H]^+^; UPLC purity: 95.2%.

##### 5-Methyl-N-(2-(2-methyl-1H-indol-3-yl)­ethyl)-1H-pyrrolo­[2,3-*c*]­pyridine-2-carboxamide (**2d**)

Yield (56 mg, 30%). ^1^H NMR (500 MHz, DMSO-*d*_6_): δ 12.64 (s, 1H), 10.72 (s, 1H), 9.02 (t, *J* = 5.0 Hz, 1H), 8.80 (s, 1H), 7.79 (s, 1H), 7.48 (d, *J* = 10.0 Hz, 1H), 7.23 (d, *J* = 10.0 Hz, 1H), 7.19 (s, 1H), 6.99–6.89 (m, 2H), 3.50–3.44 (m, 2H), 2.94–2.90 (m, 2H), 2.60 (s, 3H), 2.31 (s, 3H). ESI MS *m*/*z*, 333 [M + H]^+^; UPLC purity: 95.5%.

##### 5-Methoxy-N-(2-(2-methyl-1H-indol-3-yl)­ethyl)-1H-pyrrolo­[2,3-*c*]­pyridine-2-carboxamide (**2e**)

Yield (44 mg, 20%). ^1^H NMR (500 MHz, DMSO-*d*_6_): δ 11.76 (s, 1H), 10.70 (s, 1H), 8.78 (t, *J* = 5.8 Hz, 1H), 8.40 (s, 1H), 7.47 (d, *J* = 7.6 Hz, 1H), 7.23 (d, *J* = 7.7 Hz, 1H), 6.99–6.91 (m, 4H), 3.84 (s, 3H), 3.46–3.40 (m, 2H), 2.93–2.89 (m, 2H), 2.31 (s, 3H). ESI MS *m*/*z*, 349 [M + H]^+^; UPLC purity: >99%.

##### 1-Methyl-N-(2-(2-methyl-1H-indol-3-yl)­ethyl)-1H-pyrrolo­[2,3-*c*]­pyridine-2-carboxamide (**2f**)

Yield (46 mg, 49%). ^1^H NMR (500 MHz, DMSO-*d*_6_): δ 10.72 (s, 1H), 9.16 (s, 1H), 8.95 (t, *J* = 5.8 Hz, 1H), 8.28–8.25 (m, 1H), 7.85 (d, *J* = 5.8 Hz, 1H), 7.48 (d, *J* = 7.7 Hz, 1H), 7.24–7.22 (m, 1H), 7.10 (s, 1H), 6.99–6.88 (m, 2H), 4.09 (s, 3H), 3.47–3.43 (m, 2H), 2.94–2.89 (m, 2H), 2.34 (s, 3H). ESI MS *m*/*z*, 333 [M + H]^+^; UPLC purity: 98.0%.

##### 5-Chloro-1-methyl-N-(2-(2-methyl-1H-indol-3-yl)­ethyl)-1H-pyrrolo­[2,3-*c*]­pyridine-2-carboxamide (**2g**)

Yield (105 mg, 37%). ^1^H NMR (500 MHz, DMSO-*d*_6_): δ 10.72 (s, 1H), 8.91–8.87 (m, 1H), 8.79 (s,1H), 7.73 (d, *J* = 1.0 Hz, 1H), 7.47 (d, *J* = 7.0 Hz, 1H), 7.22 (d, *J* = 5.0 Hz, 1H), 6.99–6.95 (m, 2H), 6.94–6.91 (m, 1H), 4.04 (s, 3H), 3.44–3.40 (m, 2H), 2.93–2.89 (m, 2H), 2.33 (s, 3H). ESI MS, *m*/*z*, 367 [M + H]^+^; UPLC purity: 98.9%.

##### 5-Hydroxy-1-methyl-N-(2-(2-methyl-1H-indol-3-yl)­ethyl)-1H-pyrrolo­[2,3-*c*]­pyridine-2-carboxamide (**2h**)

Yield (16 mg, 15%). ^1^H NMR (500 MHz, DMSO-*d*_6_): δ 10.72 (s, 1H), 9.98 (br s, 1H), 8.77 (t, *J* = 5.8 Hz, 1H), 8.38 (s, 1H), 7.47 (d, *J* = 7.7 Hz, 1H), 7.22 (d, *J* = 7.7 Hz, 1H), 6.99–6.91 (m, 2H), 6.72 (s, 1H), 6.34 (s, 1H), 3.93 (s, 3H), 3.42–3.38 (m, 2H), 2.91–2.88 (m, 2H), 2.33 (s, 3H). ESI MS *m*/*z*, 349 [M + H]^+^; UPLC purity: 96.3%.

##### 5-Amino-1-methyl-N-(2-(2-methyl-1H-indol-3-yl)­ethyl)-1H-pyrrolo­[2,3-*c*]­pyridine-2-carboxamide (**2i**)

Yield (30 mg, 26%). ^1^H NMR (500 MHz, DMSO-*d*_6_): δ 10.71 (s, 1H), 8.67 (t, *J* = 5.8 Hz, 1H), 8.39 (s, 1H), 7.47 (d, *J* = 7.6 Hz, 1H), 7.23 (d, *J* = 7.7 Hz, 1H), 6.99–6.91 (m, 2H), 6.65 (s, 1H), 6.56 (s, 1H), 5.56 (br s, 2H), 3.91 (s, 3H), 3.45–3.38 (m, 2H), 2.91–2.87 (m, 2H), 2.33 (s, 3H). ESI MS *m*/*z*, 348 [M + H]^+^; UPLC purity: >99%.

##### 5-Acetamido-1-methyl-N-(2-(2-methyl-1H-indol-3-yl)­ethyl)-1H-pyrrolo­[2,3-*c*]­pyridine-2-carboxamide (**2j**)

Yield (70 mg, 69%). ^1^H NMR (500 MHz, DMSO-*d*_6_): δ 10.71 (s, 1H), 10.29 (s, 1H), 8.79 (t, *J* = 5.8 Hz, 1H), 8.71 (s, 1H), 8.22 (s, 1H), 7.48 (d, *J* = 7.6 Hz, 1H), 7.23 (d, *J* = 7.7 Hz, 1H), 6.99–6.91 (m, 2H), 6.92 (s, 1H), 4.02 (s, 3H), 3.44–3.88 (m, 2H), 2.93–2.88 (m, 2H), 2.34 (s, 3H), 2.09 (s, 3H). ESI MS *m*/*z*, 390 [M + H]^+^; UPLC purity: >99%.

##### 1-Methyl-N-(2-(2-methyl-1H-indol-3-yl)­ethyl)-5-(methylamino)-1H-pyrrolo­[2,3-*c*]­pyridine-2-carboxamide (**2k**)

Yield (11 mg, 7%). ^1^H NMR (500 MHz, DMSO-*d*_6_): δ 10.70 (s, 1H), 8.64 (s, 1H), 8.43 (s, 1H), 7.48 (d, *J* = 8.0 Hz, 1H), 7.22 (d, *J* = 8.0 Hz, 1H), 6.97–6.92 (m, 2H), 6.67 (s, 1H), 6.40 (d, *J* = 1.0 Hz, 1H), 5.70 (s, 1H), 3.92 (s, 3H), 3.41–3.37 (m, 2H), 2.91–2.87 (m, 2H), 2.74 (d, *J* = 5.0 Hz, 3H), 2.07 (s, 3H). ESI MS *m*/*z*, 362 [M + H]^+;^ UPLC purity: 98.9%.

##### N^5^,1-Dimethyl-N^2^-(2-(2-methyl-1H-indol-3-yl)­ethyl)-1H-pyrrolo­[2,3-*c*]­pyridine-2,5-dicarboxamide (**2l**)

Yield (20 mg, 10%). ^1^H NMR (500 MHz, DMSO-*d*_6_): δ 10.72 (s, 1H), 8.96 (s, 1H), 8.88 (m, 1H), 8.66–8.63 (m, 1H), 8.35 (d, *J* = 1.0 Hz, 1H), 7.48 (d, *J* = 8.0 Hz, 1H), 7.22 (d, *J* = 8.0 Hz, 1H), 7.12 (s, 1H), 6.99–6.91 (m, 2H), 4.11 (s, 3H), 3.45–3.41 (m, 2H), 2.93–2.89 (m, 2H), 2.84 (d, *J* = 5.0 Hz, 3H), 2.34 (s, 3H). ESI MS *m*/*z*, 390 [M + H]^+;^ UPLC purity: 97.2%.

##### 5-Cyano-1-methyl-N-(2-(2-methyl-1H-indol-3-yl)­ethyl)-1H-pyrrolo­[2,3-*c*]­pyridine-2-carboxamide (**2m**)

Yield (71 mg, 58%). ^1^H NMR (500 MHz, DMSO-*d*_6_): δ 10.70 (s, 1H), 9.12 (s, 1H), 8.96 (t, *J* = 5.8 Hz, 1H), 8.40 (s, 1H), 7.47 (d, *J* = 7.8 Hz, 1H), 7.22 (d, *J* = 7.8 Hz, 1H), 7.15 (s, 1H), 6.99–6.91 (m, 2H), 4.11 (s, 3H), 3.46–3.42 (m, 2H), 2.93–2.88 (m, 2H), 2.33 (s, 3H). ESI MS *m*/*z*, 358 [M + H]^+^; UPLC purity: 98.6%.

##### 1-Methyl-N-(2-(2-methyl-1H-indol-3-yl)­ethyl)-5-(methylsulfonyl)-1H-pyrrolo­[2,3-*c*]­pyridine-2-carboxamide (**2n**)

Yield (70 mg, 75%). ^1^H NMR (500 MHz, DMSO-*d*_6_): δ 10.72 (s, 1H), 9.13 (s, 1H), 8.98 (t, *J* = 5.8 Hz, 1H), 8.38 (s, 1H), 7.48 (d, *J* = 7.7 Hz, 1H), 7.24–7.22 (m, 2H), 6.99–6.91 (m, 2H), 4.14 (s, 3H), 3.46–3.42 (m, 2H), 3.23 (s, 3H), 2.94–2.90 (m, 2H), 2.30 (s, 3H). ESI MS *m*/*z*, 411 [M + H]^+^; UPLC purity: 97.8%.

##### 1,5,7-Trimethyl-N-(2-(2-methyl-1H-indol-3-yl)­ethyl)-1H-pyrrolo­[2,3-*c*]­pyridine-2-carboxamide (**2o**)

Yield (42 mg, 48%). ^1^H NMR (500 MHz, DMSO-*d*_6_): δ 10.71 (s, 1H), 8.73 (t, *J* = 5.8 Hz, 1H), 7.47 (d, *J* = 7.7 Hz, 1H), 7.24–7.22 (m, 2H), 6.99–6.91 (m, 2H), 6.76 (s, 1H), 4.17 (s, 3H), 3.43–3.39 (m, 2H), 2.91–2.89 (m, 5H), 2.44 (s, 3H), 2.33 (s, 3H). ESI MS *m*/*z*, 361 [M + H]^+^; UPLC purity: 99.0%.

##### 5-Methoxy-1-methyl-N-(2-(2-methyl-1H-indol-3-yl)­ethyl)-1H-pyrrolo­[2,3-*c*]­pyridine-2-carboxamide (**2p, BPN-37112**)

Yield (416 mg, 91%): ^1^H NMR (500 MHz, DMSO-*d*_6_): δ 10.71 (s, 1H), 8.77 (t, *J* = 5.3 Hz, 1H), 8.56 (s, 1H), 7.48 (d, *J* = 7.6 Hz, 1H), 7.23 (d, *J* = 7.7 Hz, 1H), 6.99–6.92 (m, 3H), 6.82 (s, 1H), 3.98 (s, 3H), 3.85 (s, 3H), 3.45–3.40 (m, 2H), 2.95–2.87 (m, 2H), 2.33 (s, 3H). ESI MS *m*/*z*, 363 [M + H]^+^; UPLC purity: >99%.

##### 5-(Methoxy-*d*_3_)-1-methyl-N-(2-(2-methyl-1H-indol-3-yl)­ethyl)-1H-pyrrolo­[2,3-*c*]­pyridine-2-carboxamide (**2q**)

Yield (31 mg, 37%). ^1^H NMR (500 MHz, DMSO-*d*_6_): δ 10.71 (s, 1H), 8.77 (t, *J* = 5.8 Hz, 1H), 8.56 (s, 1H), 7.48 (d, *J* = 7.6 Hz, 1H), 7.23 (d, *J* = 7.8 Hz, 1H), 6.99–6.91 (m, 3H), 6.82 (s, 1H), 3.98 (s, 3H), 3.43–3.39 (m, 2H), 2.91–2.89 (m, 2H), 2.33 (s, 3H). ESI MS *m*/*z*, 366 [M + H]^+^; UPLC purity: >99%.

##### 5-Ethoxy-1-methyl-N-(2-(2-methyl-1H-indol-3-yl)­ethyl)-1H-pyrrolo­[2,3-*c*]­pyridine-2-carboxamide (**2r**)

Yield (78 mg, 86%). ^1^H NMR (500 MHz, DMSO-*d*_6_): δ 10.71 (s, 1H), 8.76 (t, *J* = 5.7 Hz, 1H), 8.55 (s, 1H), 7.48 (d, *J* = 7.6 Hz, 1H), 7.23 (d, *J* = 7.8 Hz, 1H), 6.99–6.90 (m, 3H), 6.82 (s, 1H), 4.27 (q, *J* = 7.0 Hz, 2H), 3.98 (s, 3H), 3.43–3.39 (m, 2H), 2.92–2.89 (m, 2H), 2.33 (s, 3H), 1.32 (t, *J* = 7.0 Hz, 3H). ESI MS *m*/*z*, 377 [M + H]^+^; UPLC purity: >99%.

##### 5-Isopropoxy-1-methyl-N-(2-(2-methyl-1H-indol-3-yl)­ethyl)-1H-pyrrolo­[2,3-*c*]­pyridine-2-carboxamide (**2s**)

Yield (9 mg, 75%). ^1^H NMR (500 MHz, DMSO-*d*_6_): δ 10.71 (s, 1H), 8.76 (t, *J* = 5.7 Hz, 1H), 8.54 (s, 1H), 7.48 (d, *J* = 7.6 Hz, 1H), 7.23 (d, *J* = 7.8 Hz, 1H), 6.99–6.91 (m, 2H), 6.85 (s, 1H), 6.80 (s, 1H), 5.20–5.15 (m, 1H), 3.98 (s, 3H), 3.43–3.39 (m, 2H), 2.92–2.89 (m, 2H), 2.33 (s, 3H), 1.27 (d, *J* = 6.2 Hz, 6H). ESI MS *m*/*z*, 391 [M + H]^+^; UPLC purity: >99%.

##### 5-(2-(2-Methoxyethoxy)­ethoxy)-1-methyl-N-(2-(2-methyl-1H-indol-3-yl)­ethyl)-1H-pyrrolo­[2,3-*c*]­pyridine-2-carboxamide (**2t**)

Yield (18 mg, 27%). ^1^H NMR (500 MHz, DMSO-*d*_6_): δ 10.71 (s, 1H), 8.77 (t, *J* = 5.8 Hz, 1H), 8.55 (s, 1H), 7.48 (d, *J* = 7.6 Hz, 1H), 7.23 (d, *J* = 7.8 Hz, 1H), 6.99–6.91 (m, 3H), 6.82 (s, 1H), 4.35–4.33 (m, 2H), 3.98 (s, 3H), 3.75–3.72 (m, 2H), 3.59–3.57 (m, 2H), 3.47–3.38 (m, 4H), 3.25 (s, 3H), 2.92–2.89 (m, 2H), 2.33 (s, 3H). ESI MS *m*/*z*, 451 [M + H]^+^; UPLC purity: >99%.

##### 1,7-Dimethyl-N-(2-(2-methyl-1H-indol-3-yl)­ethyl)-1H-pyrrolo­[2,3-*c*]­pyridine-2-carboxamide (**2u**, **BPN-37440**)

Yield (14 mg, 63%). ^1^H NMR (500 MHz, DMSO-*d*_6_): δ 10.71­(s, 1H), 8.77–8.73 (m, 1H), 7.96 (d, *J* = 5.0 Hz, 1H), 7.46 (d, *J* = 5.0 Hz, 1H), 7.40 (d, *J* = 7.0 Hz, 1H), 7.22 (d, *J* = 5.0 Hz, 1H), 6.99–6.91 (m, 2H), 6.89 (s, 1H), 4.20 (s, 3H), 3.44–3.40 (m, 2H), 2.93 (s, 3H), 2.91–2.87 (m, 2H), 2.33 (s, 3H). ESI MS, *m*/*z*, 347 [M + H]^+^; UPLC purity: 98.4%.

##### 7-Isopropyl-1-methyl-N-(2-(2-methyl-1H-indol-3-yl)­ethyl)-1H-pyrrolo­[2,3-*c*]­pyridine-2-carboxamide (**2v**)

Yield (60 mg, 36%). ^1^H NMR (500 MHz, DMSO-*d*_6_): δ 10.72 (s, 1H), 8.76 (t, *J* = 5.8 Hz, 1H), 8.10 (d, *J* = 5.3 Hz, 1H), 7.48 (d, *J* = 7.6 Hz, 1H), 7.40 (d, *J* = 5.3 Hz, 1H), 7.23 (d, *J* = 7.8 Hz, 1H), 7.00–6.89 (m, 2H), 6.84 (s, 1H), 4.15 (s, 3H), 3.94–3.85 (m, 1H), 3.45–3.41 (m, 2H), 2.93–2.89 (m, 2H), 2.34 (s, 3H), 1.33 (d, *J* = 6.6 Hz, 6H). ESI MS *m*/*z*, 375 [M + H]^+^; UPLC purity: >99%.

##### 7-Cyclopropyl-1-methyl-N-(2-(2-methyl-1H-indol-3-yl)­ethyl)-1H-pyrrolo­[2,3-*c*]­pyridine-2-carboxamide (**2w**)

Yield (30 mg, 50%). ^1^H NMR (500 MHz, DMSO-*d*_6_): δ 10.72 (s, 1H), 8.77 (t, *J* = 5.8 Hz, 1H), 7.97 (d, *J* = 5.3 Hz, 1H), 7.49 (d, *J* = 7.7 Hz, 1H), 7.35 (d, *J* = 5.4 Hz, 1H), 7.23 (d, *J* = 7.8 Hz, 1H), 7.00–6.91 (m, 2H), 6.85 (s, 1H), 4.32 (s, 3H), 3.45–3.41 (m, 2H), 2.94–2.90 (m, 2H), 2.81–2.74 (m, 1H), 2.34 (s, 3H), 1.16–1.12 (m, 2H), 1.02–0.97 (m, 2H). ESI MS *m*/*z*, 373 [M + H]^+^: UPLC purity: >99%.

##### 7-Hydroxy-1-methyl-N-(2-(2-methyl-1H-indol-3-yl)­ethyl)-1H-pyrrolo­[2,3-*c*]­pyridine-2-carboxamide (**2x**)

Yield (10 mg, 13%). ^1^H NMR (500 MHz, DMSO-*d*_6_): δ 11.00–10.95 (m, 1H), 10.70 (s, 1H), 8.58 (t, *J* = 5.8 Hz, 1H), 7.47 (d, *J* = 7.6 Hz, 1H), 7.23 (d, *J* = 7.8 Hz, 1H), 7.00–6.84 (m, 3H), 6.75 (s, 1H), 6.42 (d, *J* = 6.9 Hz, 1H), 4.29 (s, 3H), 3.40–3.36 (m, 2H), 2.90–2.86 (m, 2H), 2.33 (s, 3H). ESI MS *m*/*z*, 349 [M + H]^+^; UPLC purity: >99%.

##### 5-(2-Methoxyethoxy)-1-methyl-N-(2-(2-methyl-1H-indol-3-yl)­ethyl)-1H-pyrrolo­[2,3-*c*]­pyridine-2-carboxamide (**2y**)

Yield (88 mg, 19%). ^1^H NMR (500 MHz, DMSO-*d*_6_): δ 10.71 (s, 1H), 8.77 (d, *J* = 6.0 Hz, 1H), 8.54 (s, 1H), 7.47 (d, *J* = 8.0 Hz, 1H), 7.23 (s, 1H), 6.99–6.91 (m, 3H), 6.82 (s, 1H), 4.35–4.33 (m, 2H), 3.97 (s, 3H), 3.65 (d, *J* = 5.0 Hz, 2H), 3.43–3.39 (m, 2H), 3.30 (s, 3H), 2.90 (m, 2H), 2.36 (s, 3H). ESI MS *m*/*z*, 407 [M + H]^+;^ UPLC purity: 97.7%.

##### 5-Methoxy-1-methyl-N-(2-(2-methyl-1H-indol-3-yl)­ethyl)-1H-pyrrolo­[2,3-*b*]­pyridine-2-carboxamide (**2z**)

Yield (30 mg, 23%). ^1^H NMR (500 MHz, DMSO-*d*_6_): δ 10.71 (s, 1H), 8.66 (t, *J* = 5.8 Hz, 1H), 8.14 (d, *J* = 2.75 Hz, 1H), 7.65 (d, *J* = 2.75 Hz, 1H), 7.49 (d, *J* = 7.7 Hz, 1H), 7.23 (d, *J* = 7.7 Hz, 1H), 6.99–6.92 (m, 3H), 3.99 (s, 3H), 3.84 (s, 3H), 3.45–3.39 (m, 2H), 2.93–2.89 (m, 2H), 2.34 (s, 3H). ESI MS *m*/*z*, 363 [M + H]^+^; UPLC purity: >99%.

##### 1,5-Dimethyl-N-(2-(2-methyl-1H-indol-3-yl)­ethyl)-1H-pyrrolo­[2,3-*b*]­pyridine-2-carboxamide (**2aa**)

Yield (54 mg, 23%). ^1^H NMR (500 MHz, DMSO-*d*_6_): δ 10.70 (s, 1H), 8.66 (m, 1H), 8.23 (d, *J* = 2.0 Hz, 1H), 7.87–7.86 (m, 1H), 7.48 (d, *J* = 8.0 Hz, 1H), 7.23 (d, *J* = 8.0 Hz, 1H), 6.99–6.91 (m, 3H), 3.99 (s, 3H), 3.43–3.39 (m, 2H), 2.92–2.88 (m, 2H), 2.39 (s, 3H), 2.33 (s, 3H). ESI MS *m*/*z*, 347 [M + H]^+;^ UPLC purity: 99.0%.

##### 5-Amino-1-methyl-N-(2-(2-methyl-1H-indol-3-yl)­ethyl)-1H-pyrrolo­[2,3-*b*]­pyridine-2-carboxamide (**2bb**)

Yield (78 mg, 69%). ^1^H NMR (500 MHz, DMSO-*d*_6_): δ 10.70 (s, 1H), 8.51 (m, 1H), 7.90 (d, *J* = 3.0 Hz, 1H), 7.48 (d, *J* = 8.0 Hz, 1H), 7.23 (d, *J* = 8.0 Hz, 1H), 7.12 (d, *J* = 3.0 Hz, 1H), 6.99–6.91 (m, 2H), 6.74 (s, 1H), 4.84 (s, 2H), 3.94 (s, 3H), 3.41–3.37 (m, 2H), 2.91–2.87 (m, 2H), 2.07 (s, 3H). ESI MS *m*/*z*, 348 [M + H]^+;^ UPLC purity: 98.8%.

### Cell Culture

Recently, we have created rat C6 glioma (C6G) cells stably expressing human EP2, EP4, DP1, or IP receptors in the laboratory^8,9,41^ and grown in Dulbecco’s Modified Eagle Medium (DMEM) (Invitrogen) supplemented with 10% (v/v) fetal bovine serum (FBS) (Invitrogen), 100 U/ml penicillin, 100 μg/mL streptomycin (Invitrogen), and 0.5 μg/mL G418 (Invitrogen).

### Cell-Based cAMP Assay

We described the details of the assay.^8,9^ Briefly, the assay is based on generation of a strong FRET signal upon the interaction of two molecules, an anti-cAMP antibody coupled to a FRET donor (Cryptate) and cAMP coupled to a FRET acceptor (d2). Endogenous cAMP produced by cells (up on activation of the prostanoid receptors) competes with labeled cAMP for binding to the cAMP antibody and thus reduces the FRET signal (Cisbio assays). Cells stably expressing human EP2, EP4, DP1, or IP receptors were seeded into 384-well plates in 40 μL complete medium (4000 cells/well) and grown overnight. The medium was carefully withdrawn and 10 μL Hanks’ Buffered Salt Solution (HBSS) (Hyclone) containing 20 μM rolipram was added into the wells to block phosphodiesterases. The cells were incubated at room temperature for 0.5–1 h and then treated with vehicle or test compound for 30 min before the addition of increasing concentrations of appropriate agonist: PGE_2_ for EP2 and EP4, BW245C for DP1, iloprost for IP (for Schild KB assay) or a fixed concentration of appropriate agonist (1–10 nM, see Supporting Information Figure 1) for IC50 assay. The cells were incubated at room temperature for 40 min, then lysed in 5 μL lysis buffer containing the FRET acceptor cAMP-d2 and 1 min later another 5 μL lysis buffer with anti-cAMP-Cryptate was added. After 1–2 h incubation at room temperature, the FRET signal was measured by an Envision 2103 Multilabel Plate Reader (PerkinElmer Life Sciences) with a laser excitation at 337 nm and dual emissions at 665 and 590 nm for d2 and Cryptate (50 μs delay), respectively. The FRET signal was expressed as F665/F590 × 10^4^.

### Anti-Inflammatory Assay

Stable BV2-hEP2 microglia cells were created in the lab^27^ and were grown overnight on 6 well plates at 400,000 cells per well in culture media. The cells were exposed to test compound **BPN-37112** (300, 1000 nM) or **BPN-37440** (0–1000 nM, see Figure 5D) for 30 min, and then the EP2 selective agonist CP544326 (30 nM) and LPS (100 ng/mL) for 2 h. All compounds were dissolved in DMSO and diluted in media just prior to cell treatment. Following incubation, media was removed from the wells, and the cells were subjected to RNA extraction and purification using Zymo Research Quick-RNA miniprep kit according to the manufacturer’s protocol (Genesee Scientific). First-strand cDNA synthesis, qRT-PCR and analysis was performed using the primers (Supporting Information Table 1). Glyceraldehyde-3-phosphate dehydrogenase (GAPDH) and β-actin were used as internal controls for relative quantification to determine whether EP2 activation modulates expression of inflammatory mediators in BV2-hEP2 microglia (Figure 5 and Supporting Information Figure 3). qRT-PCR gene expression data are presented as the mean fold change of each gene of interest in the compound treated groups compared to vehicle.

### Animal Experiments

All the mouse experimental procedures were performed in accordance with the National Institutes of Health Guide for the Care and Use of Laboratory Animals. The mouse efficacy protocol (IACUC protocol no. 201700775) was reviewed and approved by the Institutional Animal Care and Use Committee (IACUC) of Emory University. The experiments reported here are in accordance with the ARRIVE guidelines.

### Pilocarpine-Induced Status Epilepticus in Mice

We have described the methods for this in detail recently.^7,42^ Briefly, C57BL/6 male mice (8–12 weeks old, from Charles River) were batch-randomized to receive saline (10 mL/kg) or pilocarpine (freshly prepared 280 mg/kg) in saline by intraperitoneal (ip) route. All mice first received terbutaline (4 mg/kg ip) and methylscopolamine (4 mg/kg, ip) to alleviate respiratory and cardiovascular effects of pilocarpine, then 30 min later received saline or pilocarpine. Pilocarpine-treated mice experienced SE for 1 h, after which all mice (including saline-treated) were administered diazepam (10 mg/kg, ip) to interrupt SE. During this period, mice were scored for seizure severity every 5 min 44 ^40^ Immediately after receiving diazepam, mice were matched into two groups to approximately equalize the number of stage 4 seizures experienced during SE, then a coin flip determined whether a group would receive BPN-37440 ip at 3–4, 6–8 and 19 h after SE onset, or its vehicle (10% DMSO, 50% PEG400, 40% H2O, by volume), dosing at 10 mg/kg and 10 mL/kg. The experimenter was blinded as to whether each group received drug or vehicle and was not unblinded until the data had been analyzed. Mice were group housed in a warm (28 °C) and humid environment for 2–3 days, after which they were separated into individual cages. Weight and overall health as judged by a modified Irwin test^40^ were monitored daily. The starting dose of BPN-37440 for the efficacy experiments (10 mgk) was based on inflammatory induction in this model^3^ and also the pharmacokinetics data that the brain concentration should be in a therapeutic range.

### Immunohistochemical Analysis

Four days after SE, mice were anesthetized deeply with isoflurane, perfused through the heart with ice-cold PBS solution, and their brains rapidly removed from the cranium. The brain was immediately bisected through the midline, and the left hemisphere was fixed in 4% (w/v) paraformaldehyde for 24 h at 4 °C. After fixation, the left hemisphere was cryoprotected in 30% (w/v) sucrose in PBS solution. The brains were then frozen in 2-methylbutane chilled with dry ice and sectioned coronally at 35 μm using a freezing/sliding microtome.

### Microgliosis

Fluorescent images of the amygdala, dorsal hippocampus and neocortex overlying the hippocampus were acquired from IBA 1-stained brain sections with a Carl Zeiss Axio Observer A1 epifluorescence microscope equipped with an AxioCam Mrc5 camera. A threshold level for IBA 1 marker was determined to detect the maximum difference between saline control and pilocarpine-treated SE mice subject to vehicle. The camera settings for IBA 1-stained sections were −0.11 (brightness), 9.24 (contrast), and 750 ms (exposure time) with the “Moments” threshold setting used in NIH ImageJ Fiji software. The same camera settings and threshold levels were employed for all images stained. Percentage area occupied by IBA 1 indicates the area above the threshold level. The percentage areas of three sections in each brain region were averaged to provide a single value for each animal.

### Analysis of Inflammatory Mediators by Quantitative Real-Time PCR

Hippocampus tissue samples were collected and homogenized by sonication in lysis buffer supplied with the Quick-RNA MiniPrep Kit (Zymo Research). The lysates were briefly centrifuged to remove debris, and the supernatant was processed for total RNA isolation according to the manufacturer’s protocol. RNA samples were quantified using an Epoch Microplate Spectrophotometer (BioTek) and further converted to cDNA using a qScript cDNA SuperMix (Quanta Biosciences). Quantitative real-time PCR (qRT-PCR) was carried out on a CFX96 Touch Real-Time PCR Detection System (Bio-Rad) using iQ SYBR Green Supermix (Quanta Biosciences). Each reaction was performed in technical duplicates to ensure reproducibility. For data analysis, cycle threshold (Ct) values of target genes were normalized to the geometric mean of three housekeeping genes (β-actin, GAPDH, and HPRT1). Relative gene expression was calculated using the 2̂–^ΔΔCt^ method, with control samples serving as the calibrator group. ΔΔCt values were used for statistical analysis comparing non-SE vs SE-vehicle group; SE-vehicle vs SE-BPN-37440 group. Primer sequences for all genes are listed in Supporting Information Table 1.

### Y-Maze Test

We have recently published a detailed procedure for Y-maze performance test.^42^ We used the same protocol.

### Open Field Test

We have also published detailed procedure for open field test^42^ and we used the same protocol.

### Novel Object Recognitions Test

We have also published detailed procedures for NOR test recently.^42^ The protocol is slightly revised. Briefly, NORT test was performed on days 29–34 post-SE. Four distinctly different and immovable objects of similar height and firmness were used to evaluate their discriminatory ability. The experiment consisted of four phases: habituation, training 1, training 2, and testing. In the habituation phase, each mouse was placed in the empty arena and allowed to freely explore for 10 min 24 h later, the training phase followed during which each animal was exposed to two identical objects for 10 min, and the exploration time spent with the familiar objects was recorded. Another 24 h later, the same objects were used to explore 10 min and recorded to determine if they discriminate between familiar objects. After an interval of 24 h, the animals were tested again for 10 min, with one familiar object and one novel object (Figure 8A). Exploration time in both tests was defined as the duration during which the animal’s nose was within 2 cm of the object or when it made physical contact with the object. The discrimination index (DI) was then calculated to quantify the animal’s recognition ability using the following equation: DI = (time exploring novel object – time exploring familiar object)/(time exploring novel object + time exploring familiar object).

### Barnes Maze Test

The Barnes maze test was conducted to assess spatial learning and memory over a five-day period. The maze consisted of an elevated circular platform with 20 evenly spaced holes around the perimeter, one of which was connected to a hidden escape box. Visual cues were positioned outside the maze around the testing room and remained constant throughout the experiment. An overhead **light** was used as mild aversive stimuli to motivate exploration. During the training phase on Days 1 through 4, each mouse completed three trials per day. At the beginning of each 3 min trial, the overhead light was turned on, and the mouse was released in the center of the platform to freely explore until it located the target hole and entered the escape box. If a mouse failed to enter the escape box within 3 min, it was gently guided into the box by the experimenter and allowed to remain there undisturbed for 1 min. The escape location remained fixed for each mouse across all training sessions. Between trials, the platform was cleaned with 70% ethanol to eliminate olfactory cues. The latency to locate the escape box was measured using the Any-Maze automated tracking software. On Day 5, a single 90 s probe trial was performed to evaluate memory retention. For this trial, the escape box was removed, and mice were allowed to explore the maze freely. Time spent in the target quadrant corresponding to the former escape location was quantified as measures of spatial memory.

### Aqueous Solubility

Test compound was prepared as a 10 mM stock solution in DMSO. Aqueous suspensions of test compound and controls at 100 μM were prepared in assay buffer with 1% DMSO. The composition of phosphate buffered saline (PBS) is 0.01 M Phosphate Buffer, 0.0027 M Potassium Chloride, and 0.137 M Sodium chloride pH 7.4. Phosphate Buffers at pH 5.5 and 4.0 were prepared from the phosphate buffer pH 7.4 by slowly adding 12 N HCl until the desired pH was obtained. The suspensions were agitated at 200 rpm for 1 h at 25 °C. The suspensions were transferred to a 0.45 μm hydrophilic PVDF 96-well filter plate mounted on a fresh 2 mL 96-well plate and were filtered by centrifugation at 3000 rpm for 1 min 150 μL of filtrates were transferred to 96-well UV plate for absorbance measurement. In order to determine the optimal wavelength for detection (λ_max_), the absorbance spectrum of the DMSO stock solution for each compound was recorded over a broad range of wavelengths (260–450 nm) using a UV/vis plate reader (Molecular Devices Spectramax i3). To prepare calibration curves, a 1:1 serial dilution was performed on each compound starting at 100 μM to generate calibration solutions with concentrations ranging from 1.25 μM to 100 μM. The UV absorbance of calibrants was measured at λ_max_ for each compound. Concentration of each compound in the assay solution was calculated using the corresponding calibration curve. All assay measurements were performed in duplicate.

### In Vitro ADME Assays and In Vivo PK Experiments

The detailed methods for in vitro determination of MDR1-MDCK membrane permeability, h-ERG channel inhibition, CYP inhibition, plasma protein binding and in vivo-pharmacokinetics were described recently.^17^ These assays are done at CRO laboratories (Curia, USA or Sai Life Sciences, India) using the standard methods in drug discovery projects.

## Supplementary Material





## Notes

Supporting Information contains in vitro cellular potency, representative images of microgliosis, primers used for qRT-PCR, Open field and Barnes maze test results. Supporting Information also contains novel intermediate compounds synthesis and certificate of analysis with NMR, Mass and UPLC spectra for novel compounds, all the above as one document (PDF)
Molecular formula strings file is included as csv file (CSV)
