# Supplementary material for: Novel EP2 Antagonist Attenuates Microgliosis and Memory Deficits in Pilocarpine-Induced Status Epilepticus Mice
Source: J Med Chem. 2026 Apr 15;69(9):11024–43. doi: 10.1021/acs.jmedchem.6c00172 (PMC13181794; doi:10.1021/acs.jmedchem.6c00172)
Supplement: Supplementary file 1 [file jm6c00172_si_001.pdf]

Supporting Information

Novel EP2 antagonist attenuates microgliosis and memory deficits in pilocarpine-induced status epilepticus mice

Thota Ganesh[1, *], Soheila Pourkhodadad[1], Paola Heman-Bozadas[1], Raymond Dingledine[1], Wenyi Wang[1], Varun Rawat[1], Nicholas H. Varvel[1], R. Jason Herr[2], Qin Jiang[2], Graham Johnson[3]

[1]Department of Pharmacology and Chemical Biology, Emory University School of Medicine, 1510 Clifton Rd, Atlanta, GA 30322. [2]Curia Global Inc. 24 Corporate Circle, Albany, NY 12203. [3]NuPharmAdvise LLC, Lakeside Dr, Sanbornton, NH 03269.

Corresponding Author
Thota Ganesh, PhD
Department of Pharmacology and Chemical Biology
Emory University School of Medicine
1510 Clifton Rd, Suite 5019
Atlanta, GA 30322
tganesh@emory.edu
Phone +1-404-727-7393

Table of contents:

1. SI Table 1. Primers used on the in vitro and in vivo qPCR …………………………..S2
2. SI Figure 1. In vitro IC50 potency of lead the compound………………………………S2
3. SI Figure 2. Invitro Schild Potency of the lead compound……………………………..S3
4. SI Figure 3. In vitro anti-inflammatory potency of the lead compound………………..S3
5. SI Figure 4. Representative images of microgliosis……………………………………S4
6. SI Figure 5. Regulation of neuroinflammatory mediators in the hippocampus………S5
7. SI Figure 6. Open field exploration of mice treated with lead compound……………S6
8. SI Figure 7. Memory deficits by Barnes maze with lead compound…………………..S6
9. SI Figure 8. Synthesis schemes for starting materials…………………………………..S7
10. SI Figure 9. NMR, HPLC and Mass spectral images of the key compounds…….S7-254

***SI* Table 1**. Primers used for gene expression analysis by qRT-PCR

**Genes**	**Forward Primer (sequence 5'-3')**	**Reverse Primer (sequence 5'-3')**
β-actin	AAGGCCAACCGTGAAAAGAT	GTGGTACGACCAGAGGCATAC
GAPDH	TGTCCGTCGTGGATCTGAC	CCTGCTTCACCACCTTCTTG
HPRT1	GGAGCGGTAGCACCTCCT	CTGGTTCATCATCGCTAATCAC
COX2	CTCCACCGCCACCACTAC	TGGATTGGAACAGCAAGGAT
mPGES-1	ATCAAGATGTACGCGGTGGC	GAGGAAATGTATCCAGGCGA
IL-1β	TGAGCACCTTCTTTTCCTTCA	TTGTCTAATGGGAACGTCACAC
IL-6	TCTAATTCATATCTTCAACCAAGAGG	TGGTCCTTAGCCACTCCTTC
TNF-α	TCTTCTGTCTACTGAACTTCGG	AAGATGATCTGAGTGTGAGGG
CCL2	CATCCACGTGTTGGCTCA	GCTGCTGGTGATCCTCTTGTA
CCL3	TGCCCTTGCTGTTCTTCTCT	GTGGAATCTTCCGGCTGTAG
CCL4	CATGAAGCTCTGCGTGTCTG	GGAGGGTCAGAGCCCATT

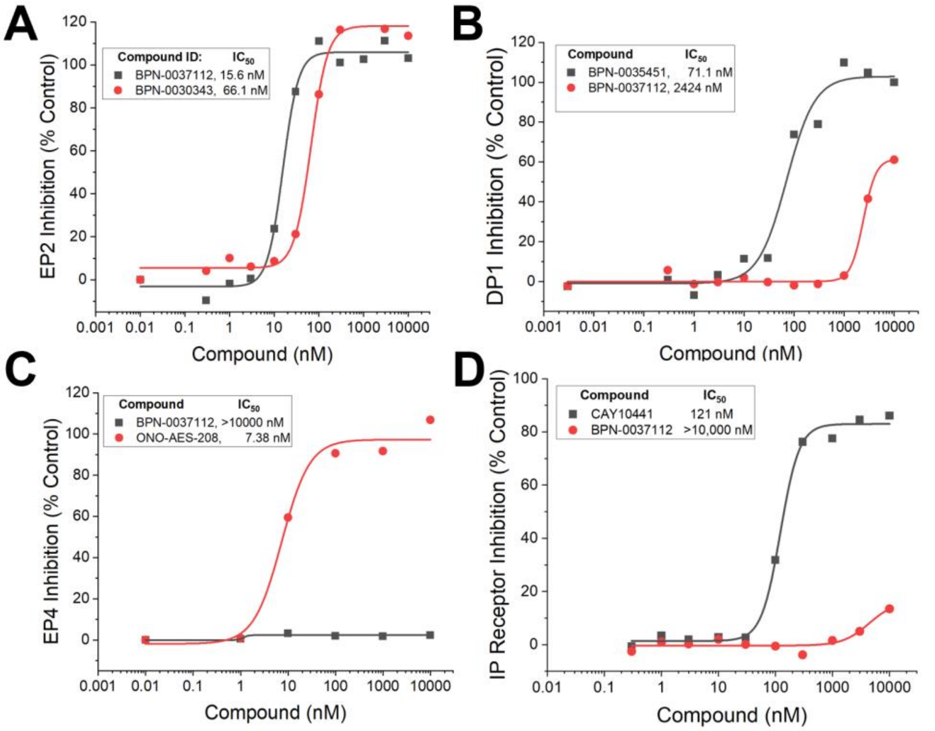

SI Fig 1: Inhibitory potency of **BPN-37112** (**2p**) against EP2 (**A**), DP1 (**B**), EP4 (**C**), IP (**D**) receptors measured in C6-glioma cells overexpressed with either of these receptors with activation with their respective agonists. 10 nM PGE2 is used for EP2 and EP4 activation (**A & C**), 1 nM BW245C was used to activate DP1 receptors (**B**), and 1 nM iloprost is used to activate IP receptors (**D**). The data indicates the compound is potent and selective EP2 antagonist. The IC_{50} data is plotted alongside the positive control used in each assay.

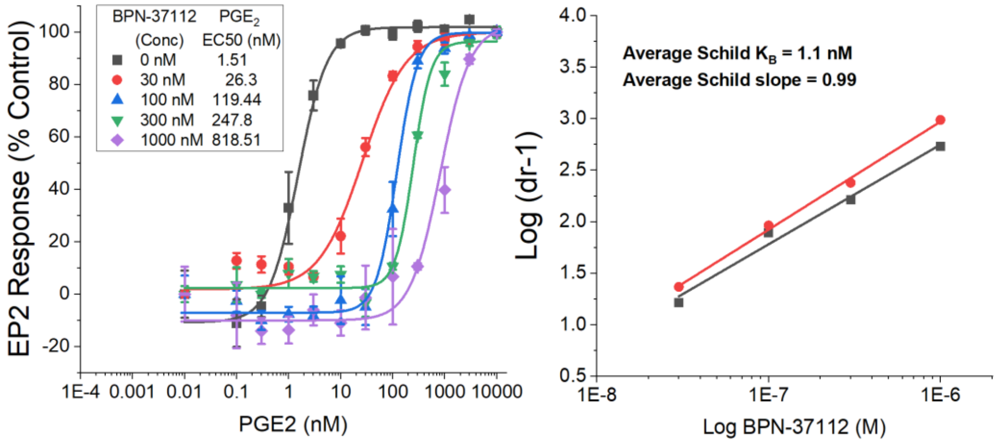

SI Figure 2. Schild regression analysis of **BPN-37112** (**2p**) indicates it is a competitive binding inhibitor with Schild K_B = 1.1 nM and Schild slope of 0.99. Schild plots with slope from 2 independent experiments are shown to get an average value.

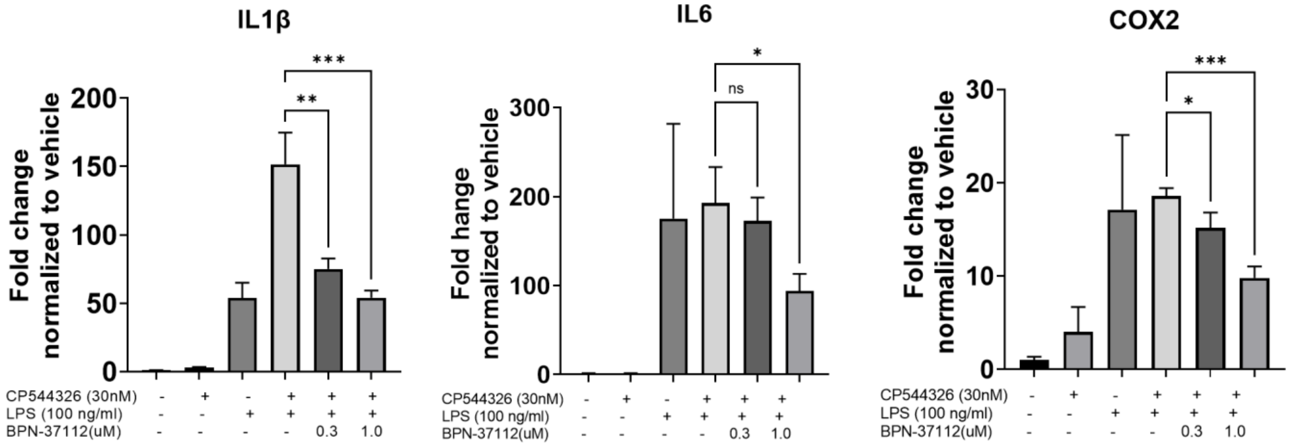

SI Fig 3: mRNA Gene expression of inflammatory mediators in BV2 microglia cell line overexpressed with human EP2 receptor (BV2-hEP2) with and without LPS, EP2 agonist (CP544326) and EP2 antagonist **BPN 37112**. * p = <0.05, ** P < 0.01, *** P < 0.001 (One-way ANOVA with Dunnett post-hoc test)

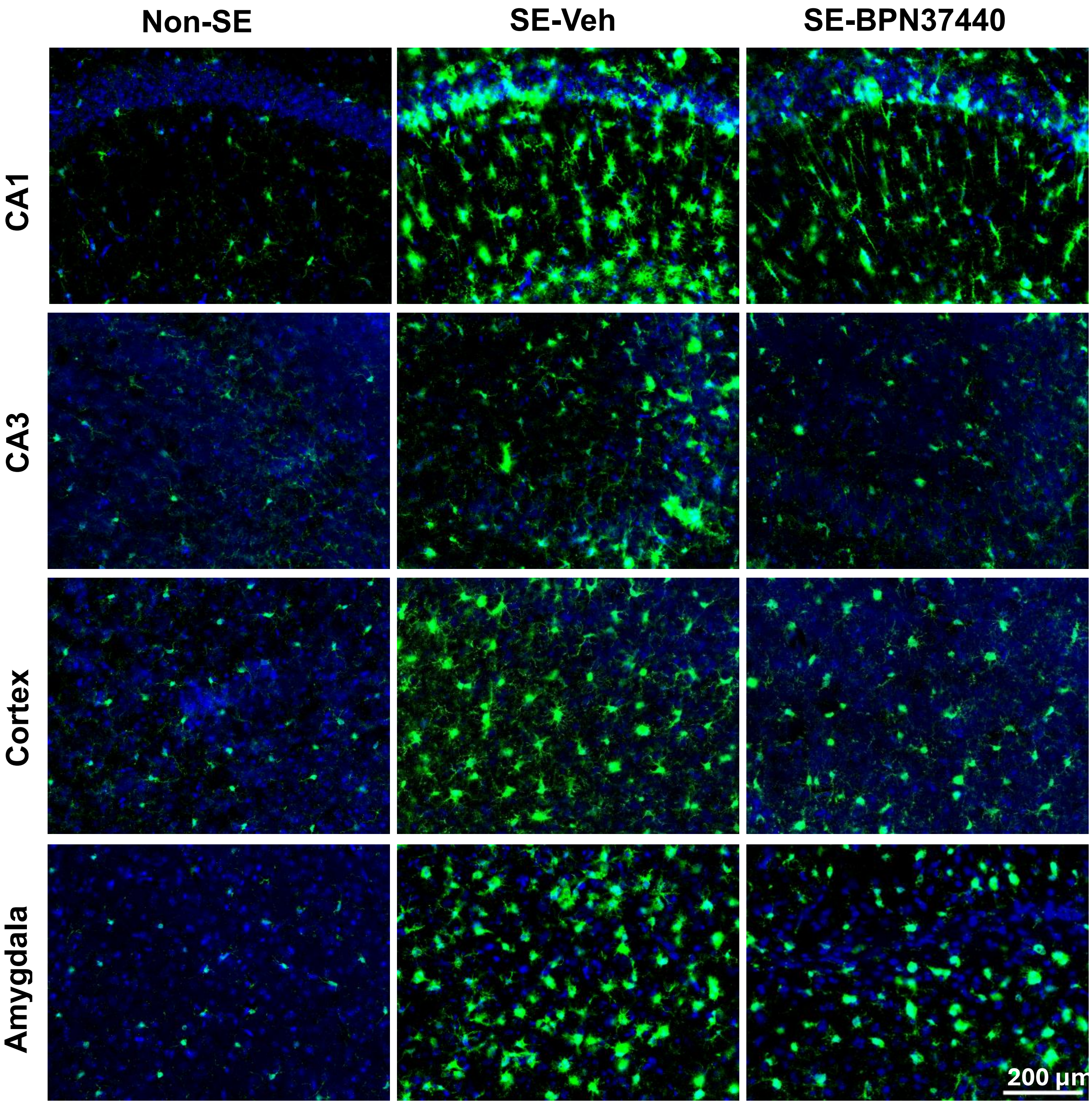

SI Fig 4: Representative fluorescence images showing Iba1 (green) and nuclear staining DAPI (blue) in different areas of the brain (CA1, CA3, Cortex and Amygdala). Four days after SE, microgliosis is noticeable in SE mice group compared to non-SE group. Administration of compound **BPN 37740** 10 mg/kg, reduced microgliosis compared with the vehicle-treated group.

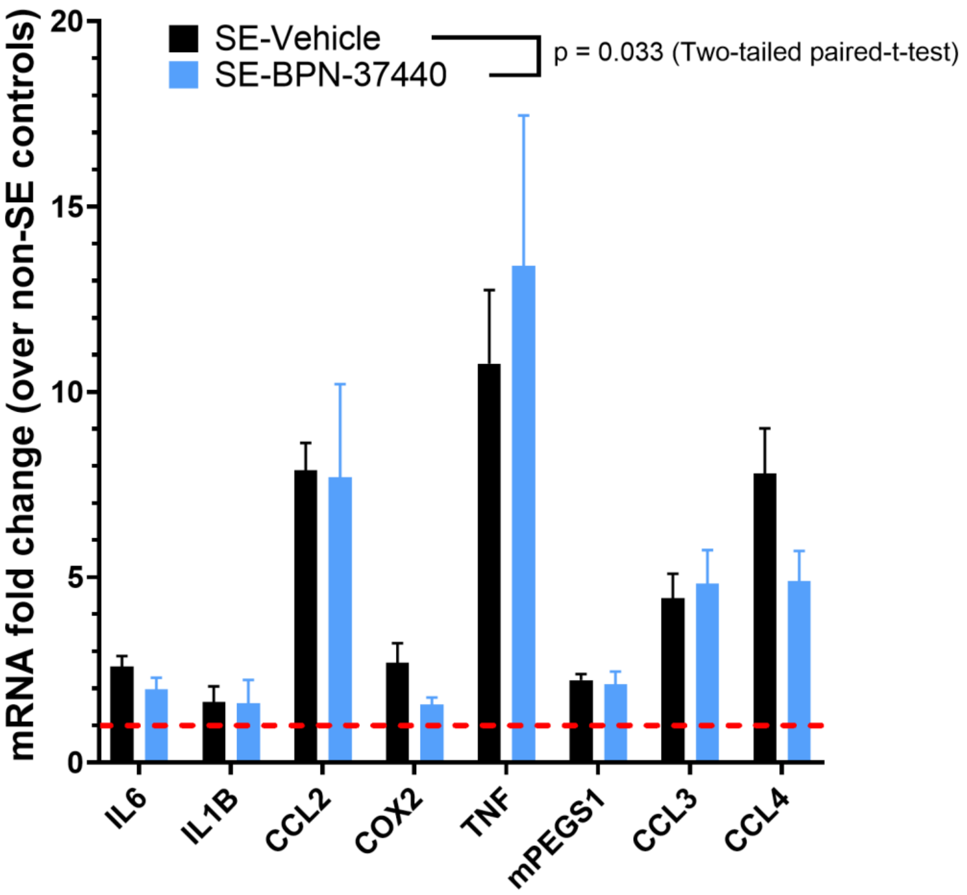

SI Fig 5. BPN-37440 causes a mild reduction of brain inflammation after SE. mRNA levels of 8 (cytokines, chemokines, COX-2, mPGS1) markers in mouse hippocampi were measured by quantitative real-time PCR. All tested cytokines and chemokines were substantially induced 4 days after pilocarpine-induced SE, as shown by fold mRNA induction, calculated as the mean mRNA (with SEM) level in SE mice treated with the vehicle relative to that in control mice without SE (n = 5-8, P = 0.0013, red-dotted line vs. black bars, Two-tailed paired t-test). Treatment with BPN-37440 reduced SE-induced mRNA induction (n = 8–9, P = 0.033 (Two-tailed, paired t-test).

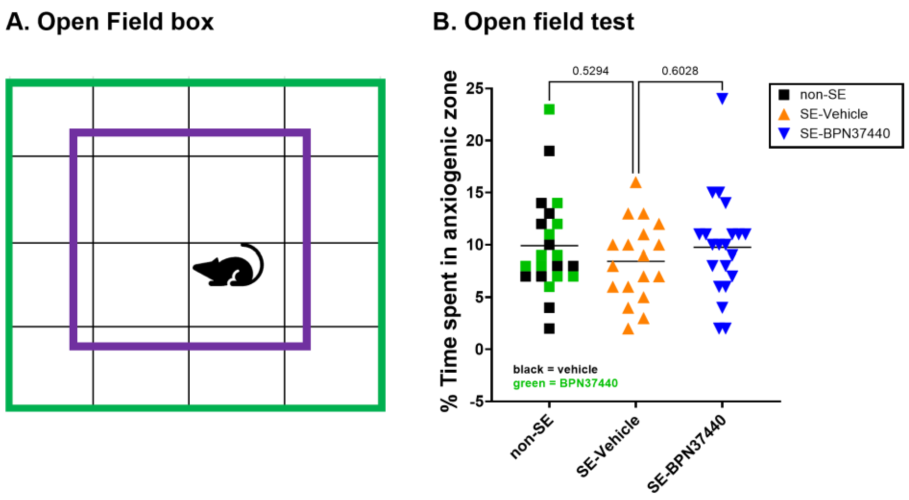

SI Fig 6. **A**. Exploration box environment used for anxiolytic behavior tests and then for familiar and novel object recognition tests. Area inside the middle of the box is 37% of the total area. **B**. SE animals with vehicle treatment showed a trend towards less time spent in the center of the box compared with the other two groups. <u>Less time in the center indicates higher anxiety in the mouse</u>. SE animals showed a trend towards increasing anxiety in mice, and **BPN-37440** treatment showed a reversing trend on anxiety of the mice but both cases data did not meet the statistical significance suggesting no impact on anxiety behavior of mice after SE and by the EP2 antagonist. One-way ANOVA with Sidak's post-hoc multiple comparisons test was used to analyze the data.

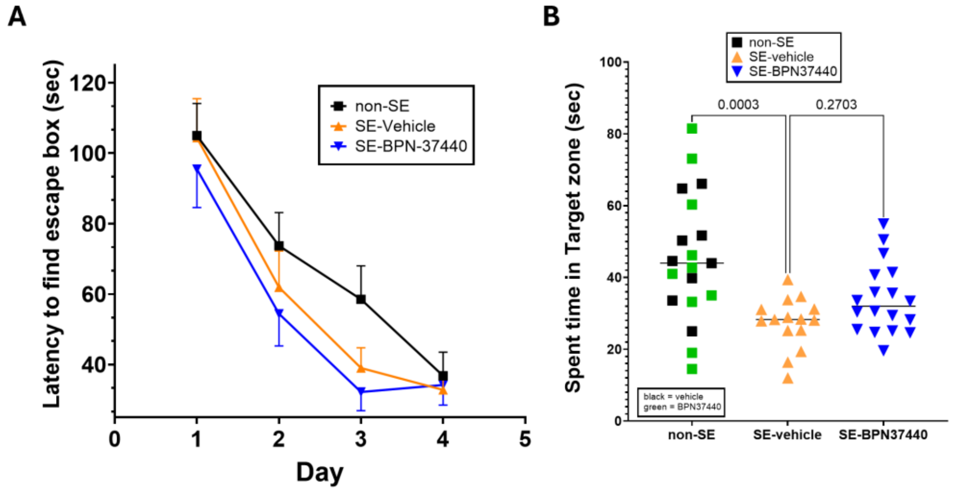

SI Fig 7. EP2 antagonist treatment showed a trend towards improving recognition memory in Barnes maze test. **A**. Mice were trained for 4 days to find the escape box (Left panel). Each mouse underwent 3 trials per day, with each trial lasting 3 minutes. P = 0.04, Two-way ANOVA, Tukey's multiple comparisons (post-hoc) test is used. **B**. The Probe trial test (right) consists of 90 seconds**.** Mice with retention memory are expected to spend more time in target quadrant. SE mice showed memory deficit in the probe trial test (P = 0.003), and the EP2 antagonist **BPN-37440** shows a reverse trend, which did not reach statistical significance (One-way ANOVA with Sidak's multiple comparisons test).

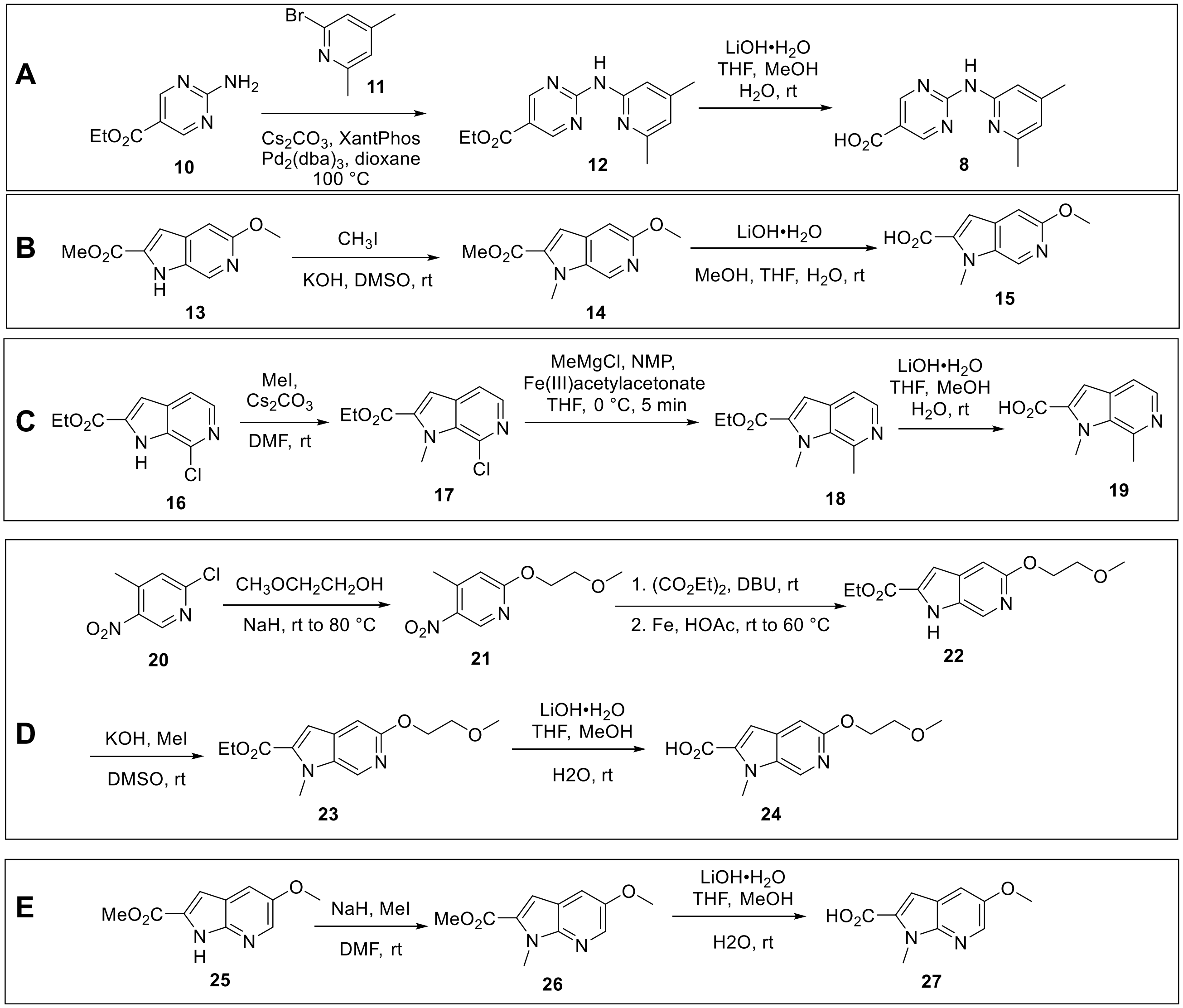

SI Fig 8: Synthesis of intermediate **8 (A), 15 (B), 19 (C), 24 (D)** and **27 (E)**. These intermediates were used for the synthesis of various novel EP2 antagonists: Reagents and conditions are shown in the arrows. The details are presented in the experimental section of the manuscript.

SI Figure 9. Certificates of analysis, NMR, Mass and HPLC spectral images of the key compounds (**1a-1ii** and **2a-2bb**)

(see next pages)

Revision: 01

CERTIFICATE OF ANALYSIS

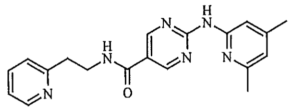

Compound Name: BPN-0035301-AA-001 1a
ALB Number: ALB-229816
Batch: 1
Lot Number: ALK-C-1-2
Molecular Formula: $C_{19}H_{20}N_6O$
Molecular Weight: 348.40
Last Solvent: Methylene Chloride, Methanol

TEST	RESULT/REFERENCE
Appearance	Off-white Solid
NMR Spectrum	^{1}H, 300 MHz, Dimethyl Sulfoxide-d_6, Consistent - Attached
Mass Spectrum	ESI, *m/z* 349 $[M + H]^+$, Attached
UPLC	>99% (area %), ACQUITY UPLC BEH C18 (2.1 *75) mm, 1.7 micron Column, UV 254 nm Detection, Attached

Revision History

Revision	Date	Description
00	June 2, 2022	Initial Issue
01	June 15, 2022	The structure was amended.

[signature]
Approved By

6-15-2022
Date

For Research Purposes Only. Not Intended for Food or Drug Use.

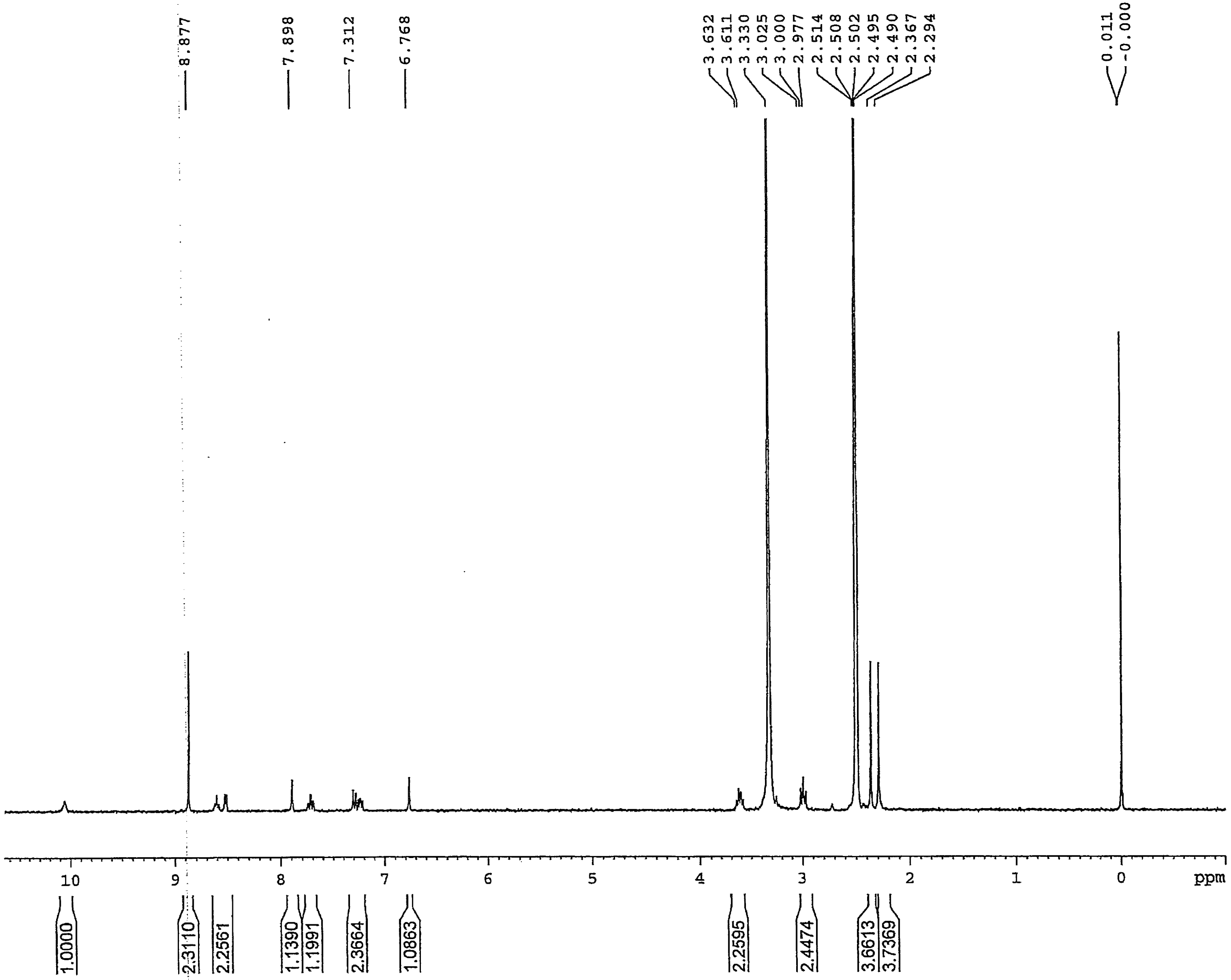

Name Marwah Albaker

Date 27 May 2022

NB # ALK-B-1-2

NAME	ALK-C-1-2
EXPNO	20
PROCNO	1
Date_	20220527
Time	9.17
INSTRUM	spect
PROBHD	5 mm QNP 1H/15
PULPROG	zg30
TD	65536
SOLVENT	DMSO
NS	32
DS	2
SWH	5995.204 Hz
FIDRES	0.091480 Hz
AQ	5.4657526 sec
RG	1149.4
DW	83.400 usec
DE	6.00 usec
TE	300.0 K
D1	1.00000000 sec
TD0	1

======== CHANNEL f1 ========

NUC1	1H
P1	12.88 usec
PL1	1.00 dB
PL1W	9.77678490 W
SFO1	300.1319509 MHz
SI	32768
SF	300.1300006 MHz
WDW	EM
SSB	0
LB	0.30 Hz
GB	0
PC	1.00

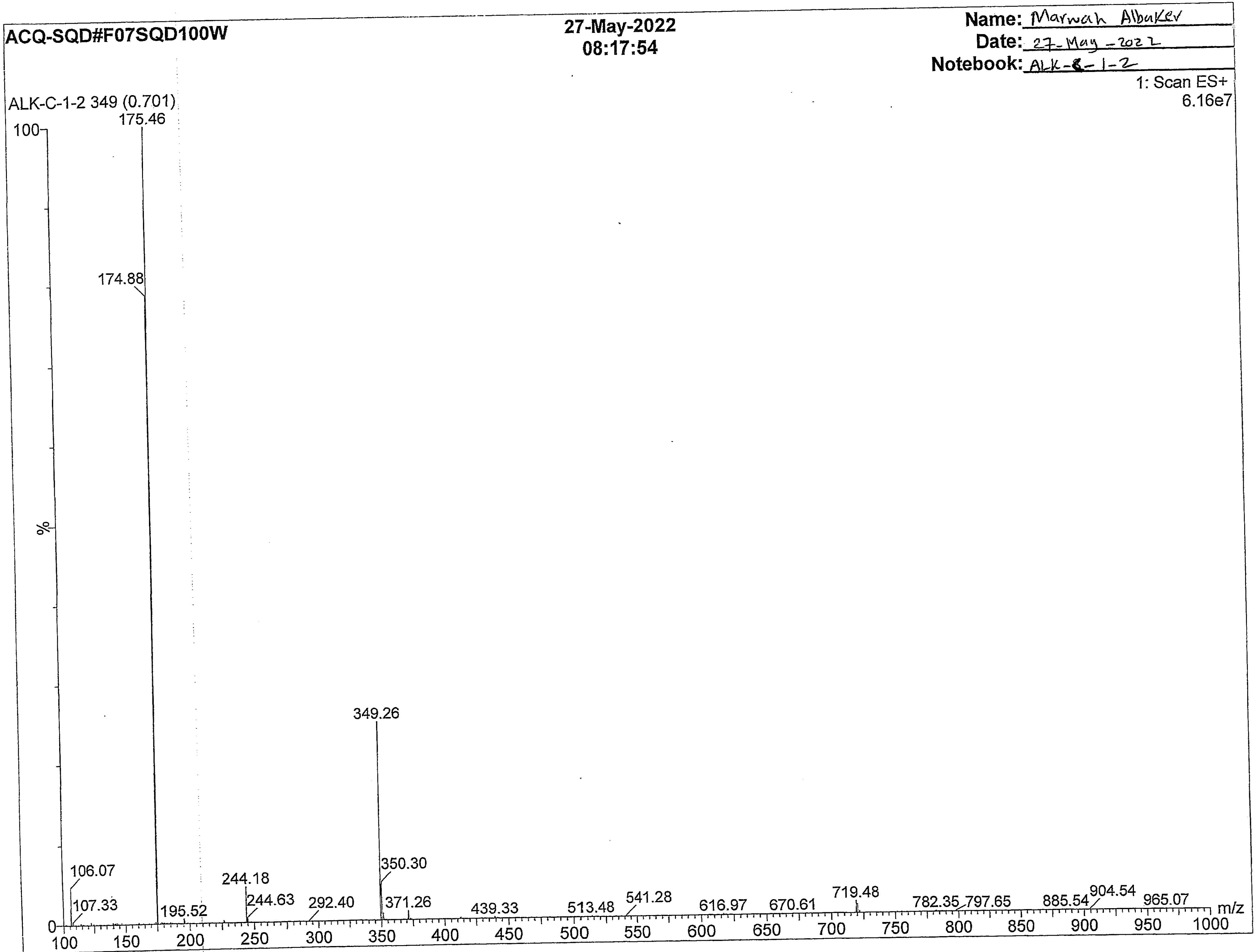
ACQ-SQD#F07SQD100W
27-May-2022
08:17:54
Name: Marwah Albaker
Date: 27-May-2022
Notebook: ALK-8-1-2
1: Scan ES+
6.16e7
ALK-C-1-2 349 (0.701)
175.46
174.88
349.26
350.30
106.07
107.33
195.52
244.18
244.63
292.40
371.26
439.33
513.48
541.28
616.97
670.61
719.48
782.35
797.65
885.54
904.54
965.07
100
%
0
m/z
100
150
200
250
300
350
400
450
500
550
600
650
700
750
800
850
900
950
1000

SAMPLE INFORMATION

Sample Name: ALK-C-1-2
Injection Volume: 3.00 ul
Run Time: 9.0 Minutes
Date Acquired: 5/31/2022 9:23:35 AM EDT
Date Processed: 5/31/2022 9:35:37 AM EDT
Sample Set Name: Template
Acq. Method Set: HSS T3_PDA_75mm_polar 408
Processing Method: BEH_C18_PDA
Channel Name: 254nm

Method Notes:
Acquity UPLC HSS T3 1.8u (2.1x75mm)
Flow Rate : 0.5 mL/min
Solvent A : 0.1% TFA in Waters
Solvent B : 0.1% TFA in Acetonitrile
Solvent Gradient Program:

Time (min)	%A	% B
0:00	95	5
1:00	95	5
3:30	70	30
6:00	0	100
8:00	0	100
9:00	95	5

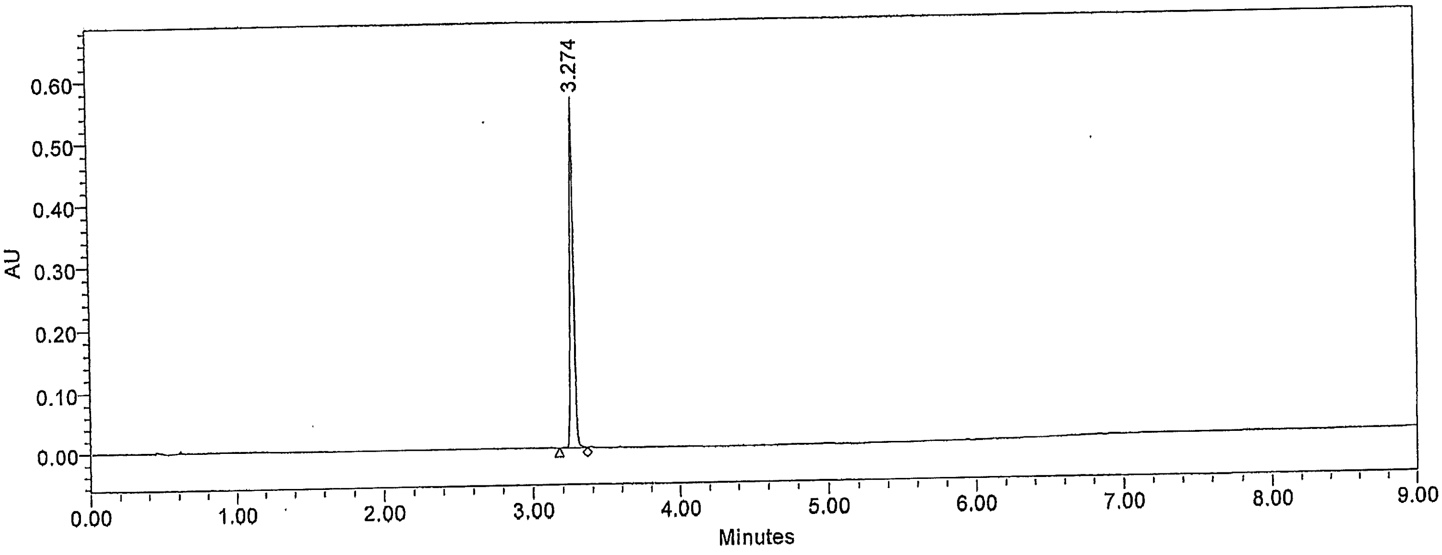

Peak Results

	RT	Area	Int Type	Width (sec)	% Area
1	3.274	946285	BV	11.249	100.00

Name: Marwah Albaker

Date: 31 May 2022

NB #: ALK-C-1-2

CERTIFICATE OF ANALYSIS

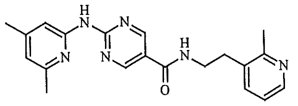

Compound Name: BPN-0035318-AA-001 1b
ALB Number: ALB-229925
Batch: 1
Lot Number: ARN-E-1-1
Molecular Formula: $C_{20}H_{22}N_6O$
Molecular Weight: 362.43
Last Solvent: Methylene Chloride, Methanol

TEST	RESULT/REFERENCE
Appearance	White Solid
NMR Spectrum	^{1}H, 500 MHz, Dimethyl Sulfoxide-d_6, Consistent - Attached
Mass Spectrum	ESI, m/z 363 $[M + H]^+$, Attached
UPLC	97.4% (area %), ACQUITY UPLC BEH C18 (2.1 *75) mm, 1.7 micron Column, UV 254 nm Detection, Attached

Approved By: Maria Maychack

Date: 6-8-2022

For Research Purposes Only. Not Intended for Food or Drug Use.

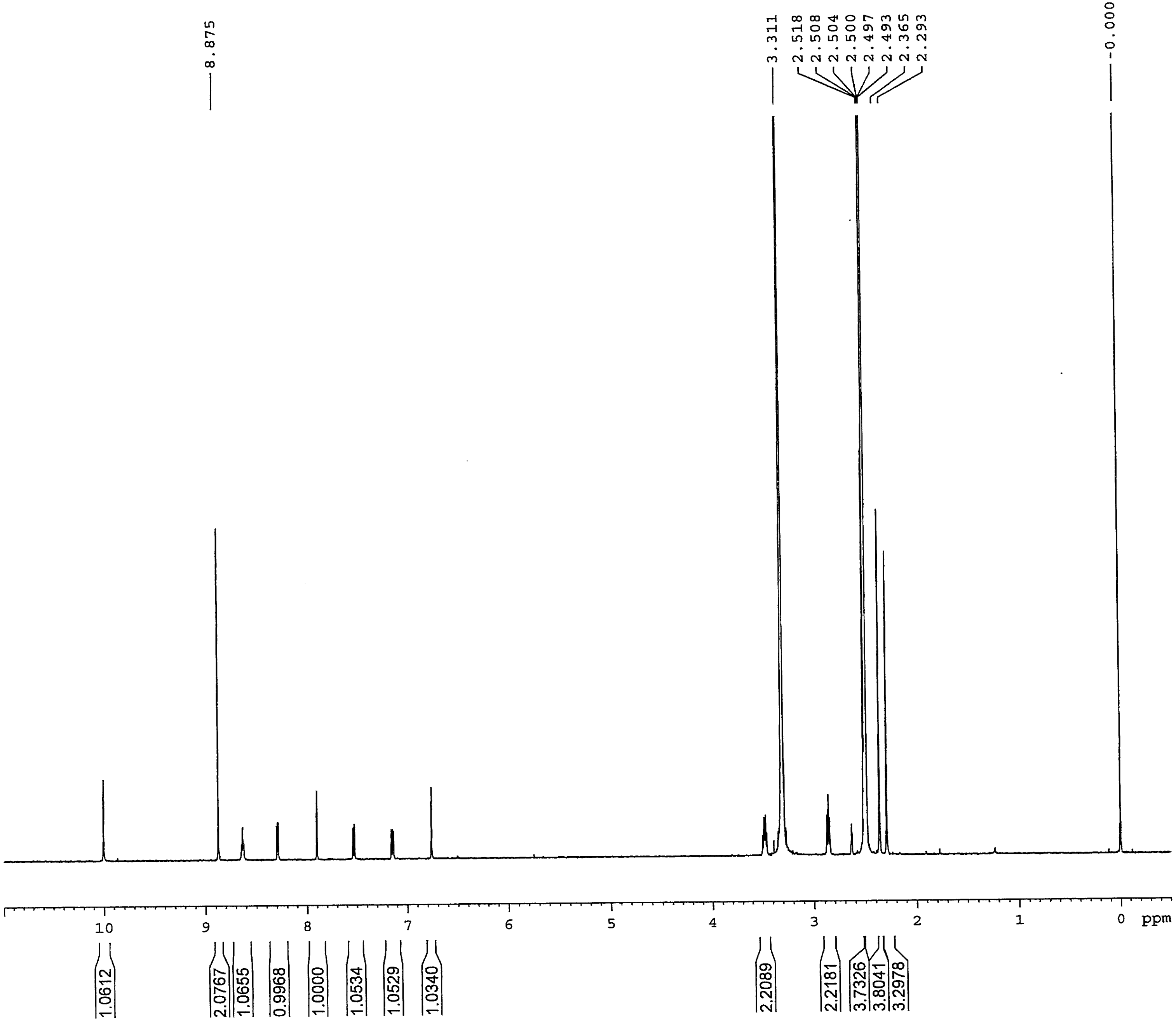

Name Corey Arnett-Butscher
Date 6/6/2022
NB # ARN-E-1-1

NAME	ARN-E-1-1	
EXPNO	10	
PROCNO	1	
Date_	20220602	
Time	11.37	h
INSTRUM	Avance Neo	
PROBHD	Z167419_0029 (	
PULPROG	zg30	
TD	65536	
SOLVENT	DMSO	
NS	32	
DS	2	
SWH	10000.000	Hz
FIDRES	0.305176	Hz
AQ	3.2768500	sec
RG	101	
DW	50.000	usec
DE	11.14	usec
TE	300.0	K
D1	1.00000000	sec
TD0	1	
SFO1	500.1330883	MHz
NUC1	1H	
P0	2.67	usec
P1	8.00	usec
SI	65536	
SF	500.1300041	MHz
WDW	EM	
SSB	0	
LB	0.30	Hz
GB	0	
PC	1.00	

Openlynx Report
Vial:1:35
Date:02-Jun-2022
Name: CorryArnett-Stryker

ID:
Time:13:20:30
Date: 6/6/2022

File:ARN-E-1-1
Notebook: ARN-E-1-1

Printed: Thu Jun 02 13:22:48 2022

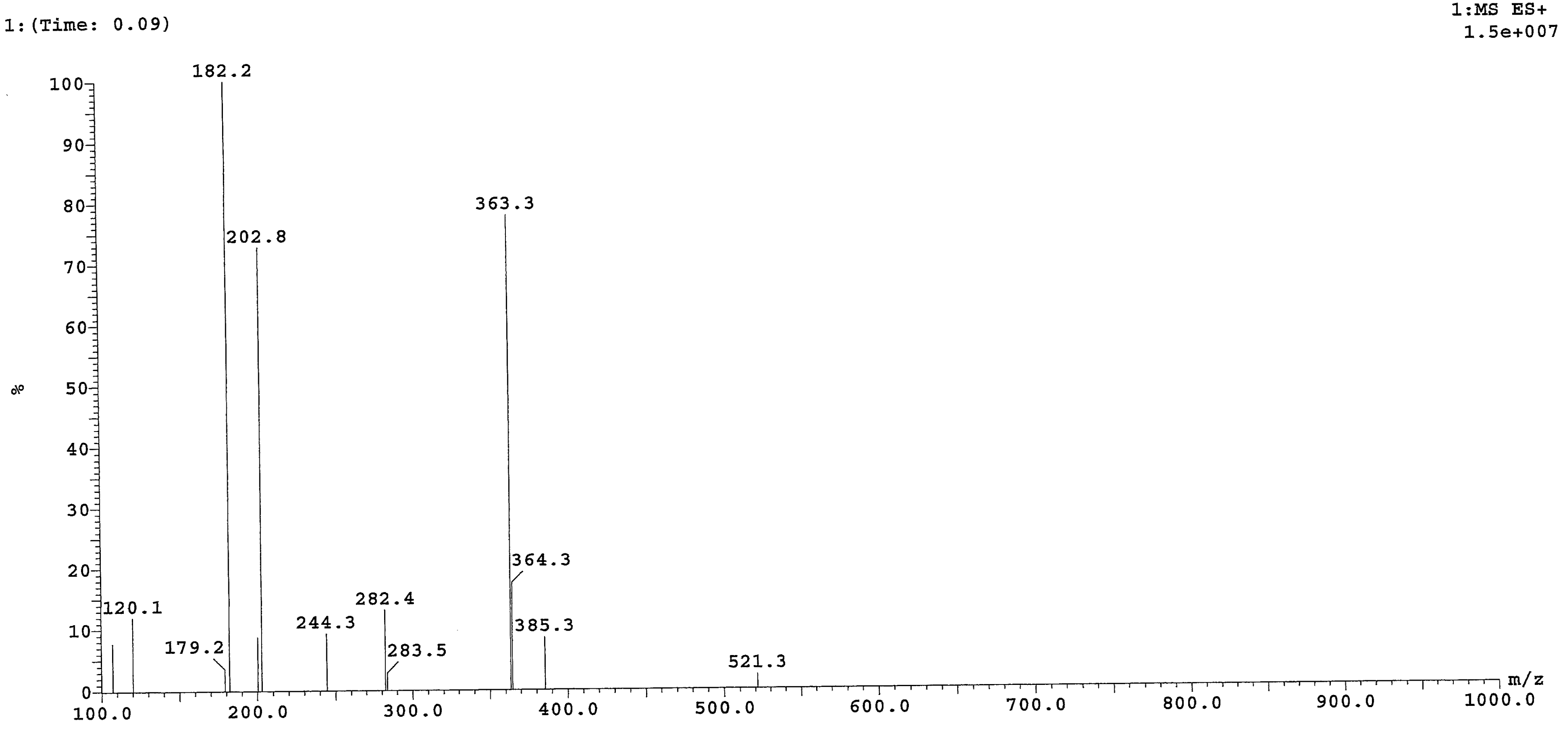

SAMPLE INFORMATION

Sample Name:	ARN-E-1-1
Injection Volume:	3.00 ul
Run Time:	9.0 Minutes
Date Acquired:	6/3/2022 8:32:15 AM EDT
Date Processed:	6/3/2022 9:01:40 AM EDT
Sample Set Name:	Template
Acq. Method Set:	BEH_C18_PDA_75mm 408
Processing Method:	BEH_C18_PDA_CAB
Channel Name:	254nm

Method Notes:
Acquity UPLC BEH C18 1.7u (2.1x75mm)
Flow Rate : 0.5 mL/min
Solvent A : 0.1% TFA in Waters
Solvent B : 0.1% TFA in Acetonitrile
Solvent Gradient Program:

Time (min)	%A	% B
0:00	95	5
6:00	0	100
8:00	0	100
9:00	95	5

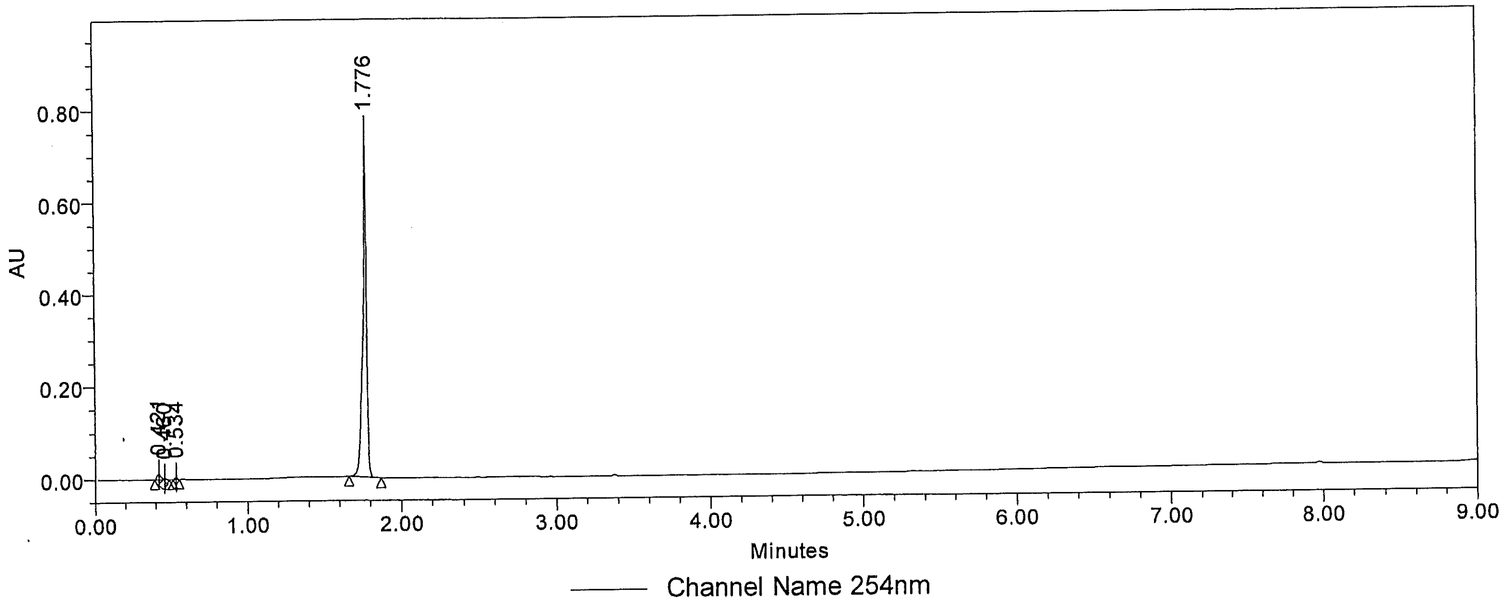

Peak Results

	RT	Area	Int Type	Width (sec)	% Area
1	0.421	20978	bV	3.250	1.70
2	0.460	4226	VB	2.350	0.34
3	0.534	5974	Bb	2.300	0.48
4	1.776	1204174	bb	12.401	97.48

Name: CoreyArnett-Butscher

Date: 6/6/2022

NB #: ARN-E-1-1

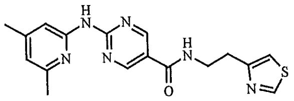

CERTIFICATE OF ANALYSIS

Compound Name: BPN-0035319-AA-001 1c
ALB Number: ALB-229926
Batch: 1
Lot Number: ARN-E-2-1
Molecular Formula: $C_{17}H_{18}N_6OS$
Molecular Weight: 354.43
Last Solvent: Methylene Chloride, Methanol

TEST	RESULT/REFERENCE
Appearance	Off-white Solid
NMR Spectrum	^{1}H, 500 MHz, Dimethyl Sulfoxide-d_6, Consistent - Attached
Mass Spectrum	ESI, m/z 355 [M + H]$^+$, Attached
UPLC	>99% (area %), ACQUITY UPLC BEH C18 (2.1 *75) mm, 1.7 micron Column, UV 254 nm Detection, Attached

Approved By

6-8-2022
Date

For Research Purposes Only. Not Intended for Food or Drug Use.

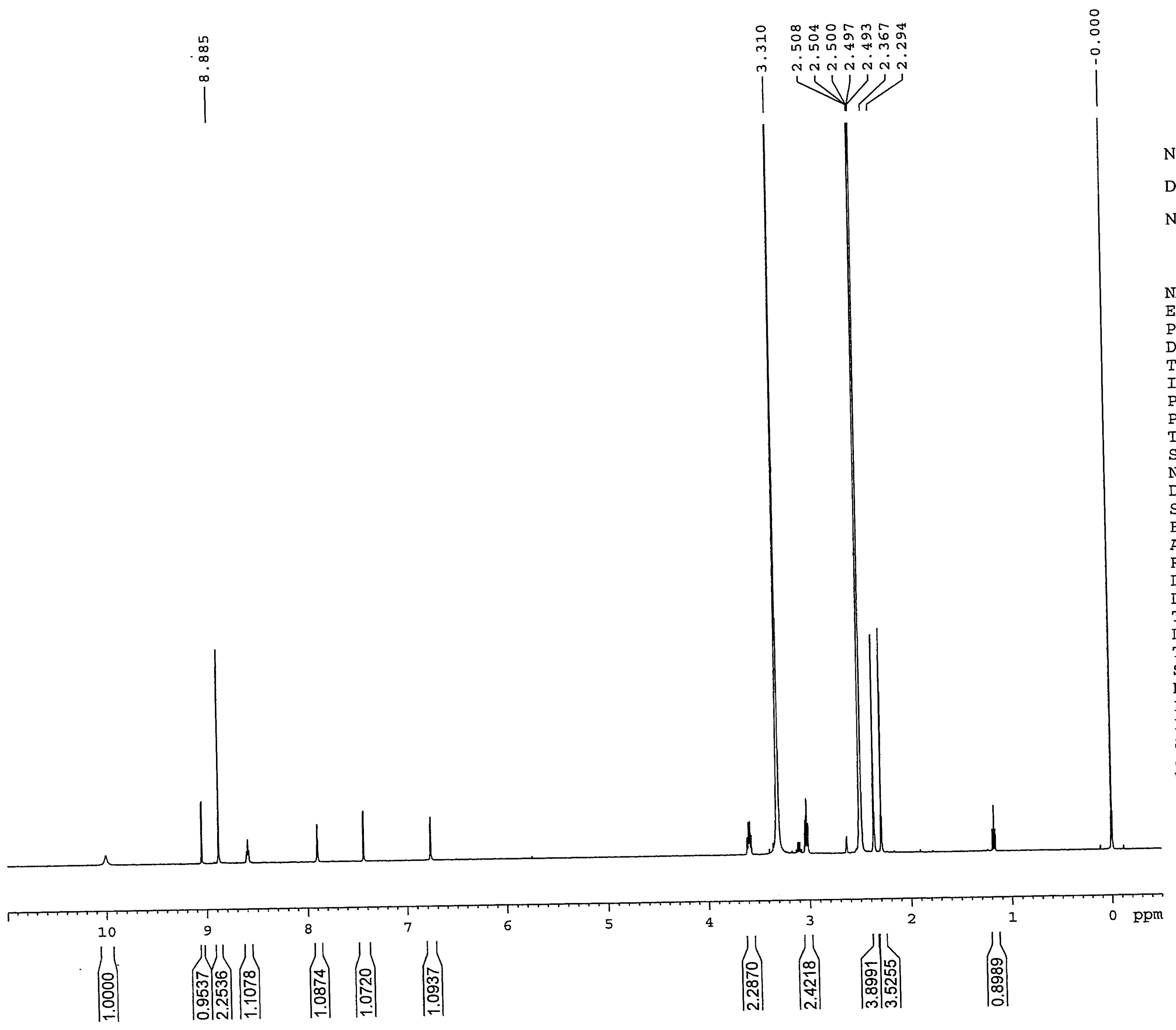

Name Corey Arnett-Butscher

Date 6/6/2022

NB # ARN-E-2-1

NAME	ARN-E-2-1
EXPNO	10
PROCNO	1
Date_	20220602
Time	11.43 h
INSTRUM	Avance Neo
PROBHD	Z167419_0029 (
PULPROG	zg30
TD	65536
SOLVENT	DMSO
NS	32
DS	2
SWH	10000.000 Hz
FIDRES	0.305176 Hz
AQ	3.2768500 sec
RG	101
DW	50.000 usec
DE	11.14 usec
TE	300.0 K
D1	1.00000000 sec
TD0	1
SFO1	500.1330883 MHz
NUC1	1H
P0	2.67 usec
P1	8.00 usec
SI	65536
SF	500.1300041 MHz
WDW	EM
SSB	0
LB	0.30 Hz
GB	0
PC	1.00

Openlynx Report
Vial:1:36
Date:02-Jun-2022
Name: Cory Arnett-Butscher

ID:
Time:13:22:51
Date: 6/6/2022

File:ARN-E-2-1
Notebook: ARN-E-2-1

Printed: Thu Jun 02 13:24:50 2022

1:(Time: 0.09)

1:MS ES+
2.3e+007

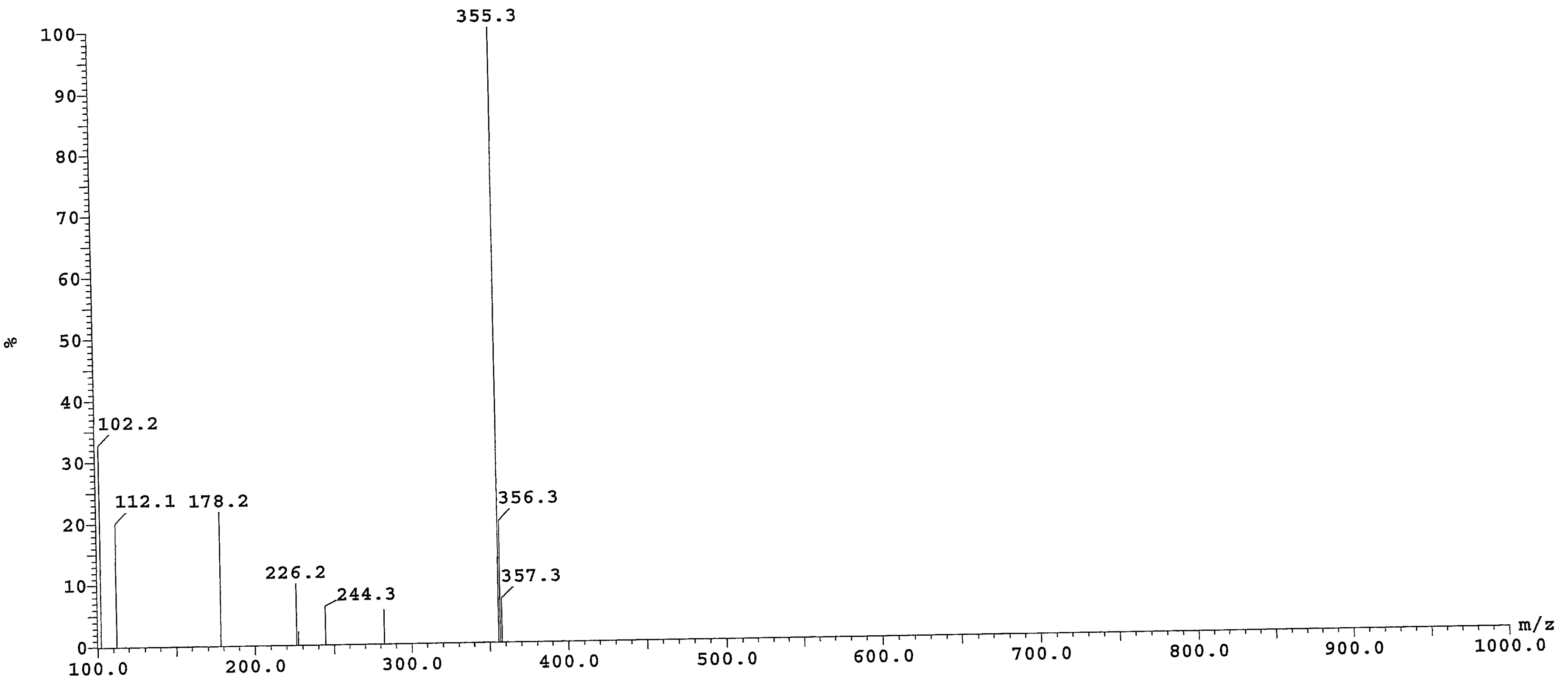

SAMPLE INFORMATION

Sample Name:	ARN-E-2-1
Injection Volume:	3.00 ul
Run Time:	9.0 Minutes
Date Acquired:	6/2/2022 11:52:38 AM EDT
Date Processed:	6/2/2022 1:05:11 PM EDT
Sample Set Name:	Template
Acq. Method Set:	BEH_C18_PDA_75mm 408
Processing Method:	BEH_C18_PDA_CAB
Channel Name:	254nm

Method Notes:
Acquity UPLC BEH C18 1.7u (2.1x75mm)
Flow Rate : 0.5 mL/min
Solvent A : 0.1% TFA in Waters
Solvent B : 0.1% TFA in Acetonitrile
Solvent Gradient Program:

Time (min)	%A	% B
0:00	95	5
6:00	0	100
8:00	0	100
9:00	95	5

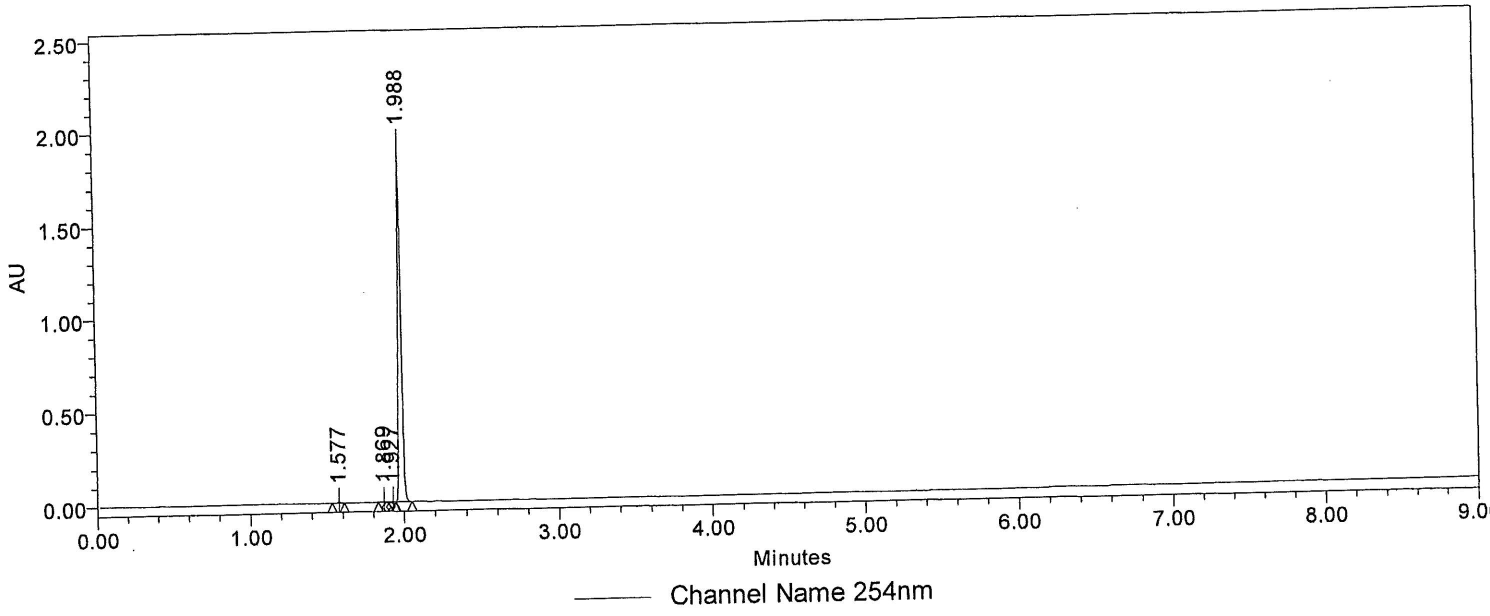

Peak Results

	RT	Area	Int Type	Width (sec)	% Area
1	1.577	6031	bb	4.750	0.22
2	1.869	5498	bV	3.350	0.20
3	1.927	3890	Vb	1.650	0.14
4	1.988	2674991	bb	6.200	99.43

Name: Corey Arnett-Butscher

Date: 6/6/2022

NB #: ARN-E-2-1

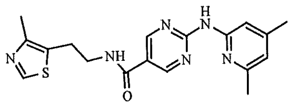

<u>**CERTIFICATE OF ANALYSIS**</u>

Compound Name: BPN-0035320-AA-001 1d
ALB Number: ALB-229927
Batch: 1
Lot Number: ALK-C-2-1
Molecular Formula: $C_{18}H_{20}N_6OS$
Molecular Weight: 368.46
Last Solvent: Ethyl Acetate

TEST	RESULT/REFERENCE
Appearance	Off-white Solid
NMR Spectrum	^{1}H, 300 MHz, Dimethyl Sulfoxide-d_6, Consistent - Attached
Mass Spectrum	ESI, m/z 369 [M + H]$^+$, Attached
UPLC	>99% (area %), ACQUITY UPLC BEH C18 (2.1 *75) mm, 1.7 micron Column, UV 254 nm Detection, Attached

[signature]
Approved By

6-8-2022
Date

For Research Purposes Only. Not Intended for Food or Drug Use.

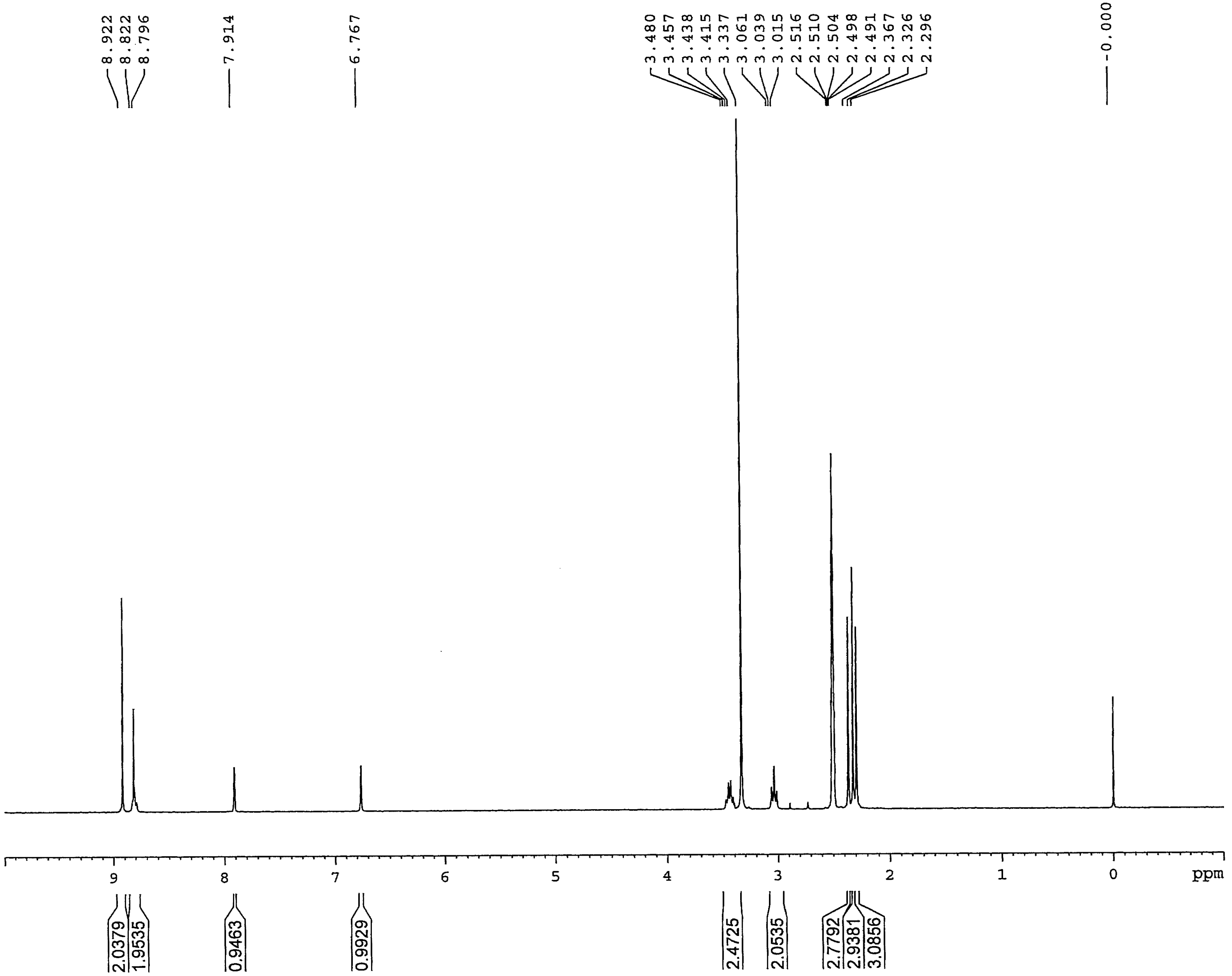

Name Marwah Albaker

Date 2-June-2022

NB # ALK_C-2-1

NAME	ALK-C-2-1
EXPNO	10
PROCNO	1
Date_	20220601
Time	13.46
INSTRUM	spect
PROBHD	5 mm QNP 1H/15
PULPROG	zg30
TD	65536
SOLVENT	DMSO
NS	16
DS	2
SWH	5995.204 Hz
FIDRES	0.091480 Hz
AQ	5.4657526 sec
RG	1149.4
DW	83.400 usec
DE	6.00 usec
TE	300.0 K
D1	1.00000000 sec
TD0	1

======== CHANNEL f1 ========

NUC1	1H
P1	12.88 usec
PL1	1.00 dB
PL1W	9.77678490 W
SFO1	300.1319509 MHz
SI	32768
SF	300.1300001 MHz
WDW	EM
SSB	0
LB	0.30 Hz
GB	0
PC	1.00

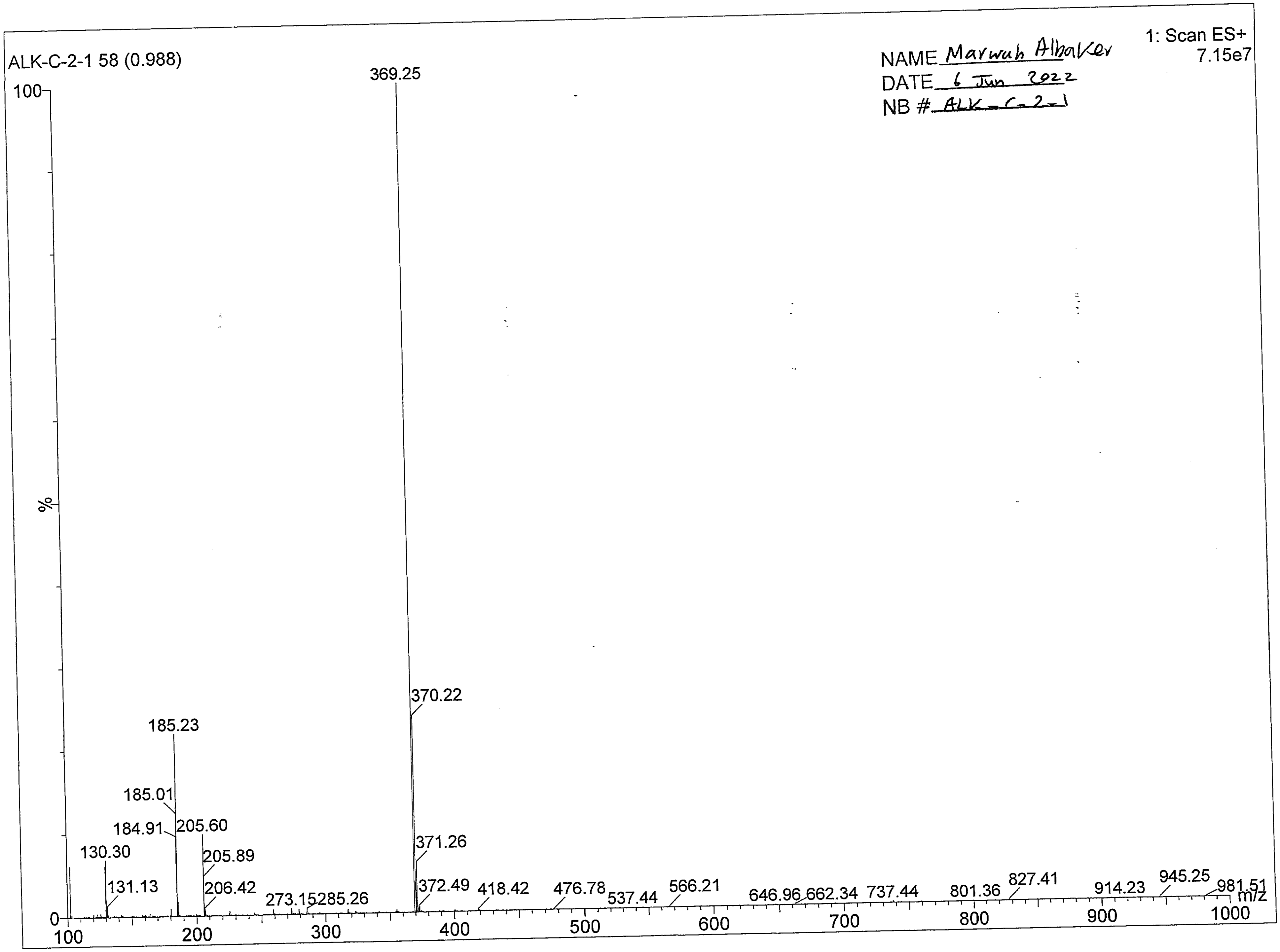
ALK-C-2-1 58 (0.988)
1: Scan ES+
7.15e7
NAME Marwah Albaker
DATE 6 Jun 2022
NB # ALK-C-2-1
100
%
0
369.25
370.22
371.26
372.49
185.23
185.01
184.91
205.60
205.89
206.42
130.30
131.13
273.15
285.26
418.42
476.78
537.44
566.21
646.96
662.34
737.44
801.36
827.41
914.23
945.25
981.51
m/z
100
200
300
400
500
600
700
800
900
1000

SAMPLE INFORMATION

Sample Name: ALK-C-2-1
Injection Volume: 3.00 ul
Run Time: 9.0 Minutes
Date Acquired: 6/1/2022 1:40:14 PM EDT
Date Processed: 6/1/2022 1:52:16 PM EDT
Sample Set Name: Template
Acq. Method Set: HSS T3_PDA_75mm_polar 408
Processing Method: BEH_C18_PDA
Channel Name: 254nm

Method Notes:
Acquity UPLC HSS T3 1.8u (2.1x75mm)
Flow Rate : 0.5 mL/min
Solvent A : 0.1% TFA in Waters
Solvent B : 0.1% TFA in Acetonitrile
Solvent Gradient Program:

Time (min)	%A	% B
0:00	95	5
1:00	95	5
3:30	70	30
6:00	0	100
8:00	0	100
9:00	95	5

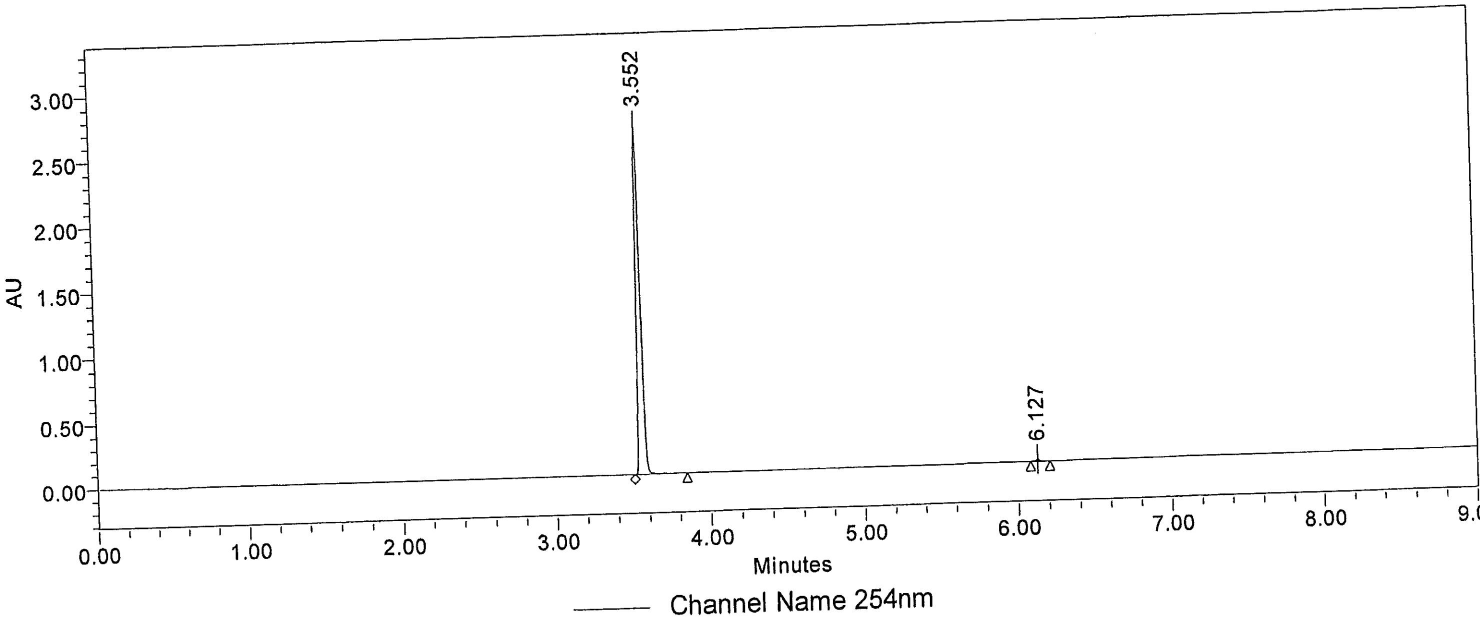

Peak Results

	RT	Area	Int Type	Width (sec)	% Area
1	3.552	4974119	VB	20.349	99.46
2	6.127	27197	BB	7.452	0.54

Name: Marwah Albaker

Date: 2-June-2022

NB #: ALK-C-2-1

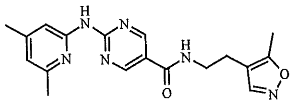

CERTIFICATE OF ANALYSIS

Compound Name: BPN-0035345-AA-001 1e
ALB Number: ALB-230038
Batch: 1
Lot Number: ARN-E-6-2
Molecular Formula: $C_{18}H_{20}N_6O_2$
Molecular Weight: 352.39
Last Solvent: Water, Ethyl Acetate

TEST	RESULT/REFERENCE
Appearance	Off-white Solid
NMR Spectrum	^{1}H, 500 MHz, Dimethyl Sulfoxide-d_6, Consistent - Attached
Mass Spectrum	ESI, *m/z* 353 [M + H]$^+$, Attached
UPLC	>99% (area %), ACQUITY UPLC BEH C18 (2.1 *75) mm, 1.7 micron Column, UV 254 nm Detection, Attached

Approved By

6-15-2022
Date

For Research Purposes Only. Not Intended for Food or Drug Use.

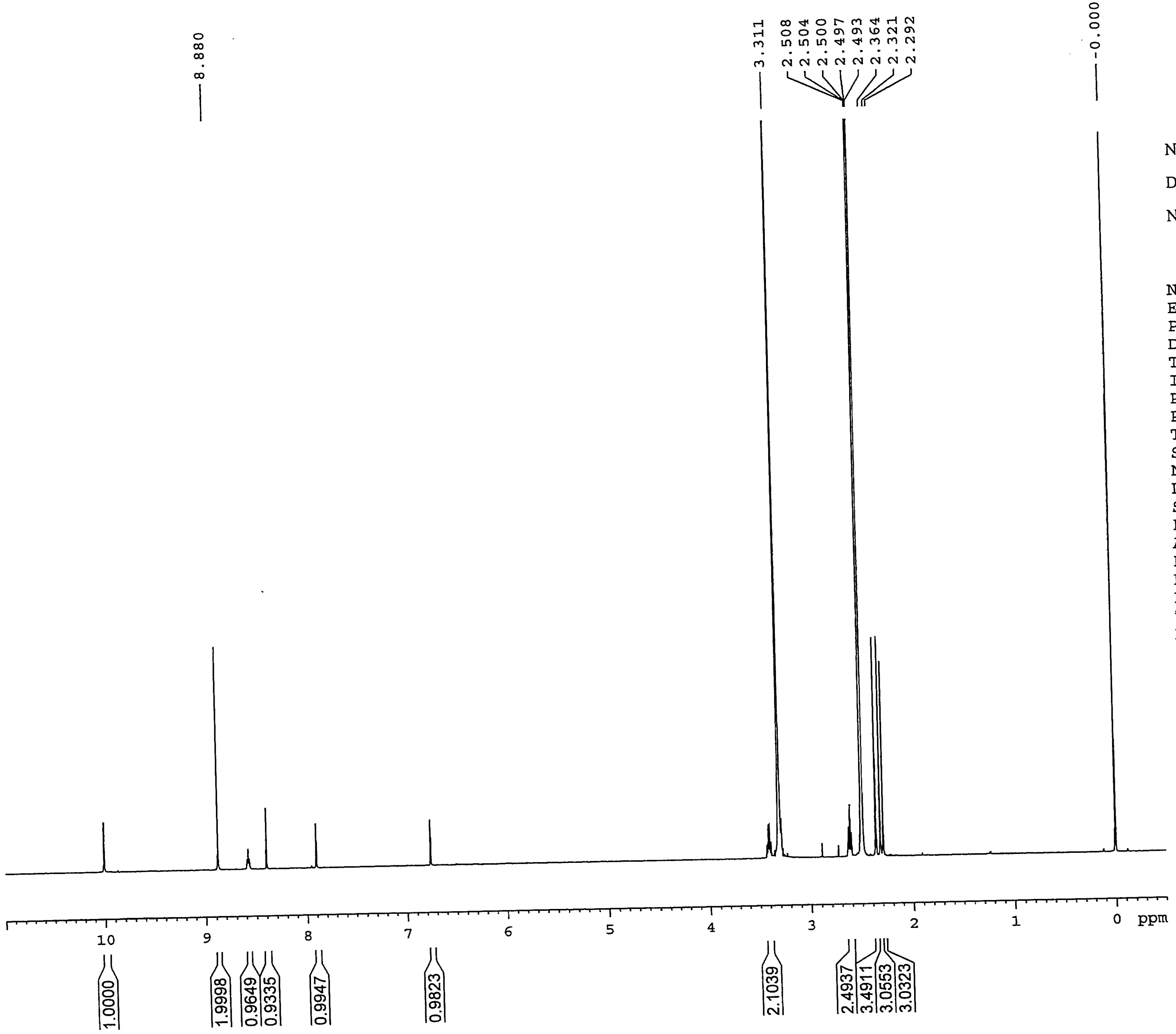

Name Cory Arnett-Butscher

Date 6/8/2022

NB # ARN-E6-2

NAME	ARN-E-6-2
EXPNO	10
PROCNO	1
Date_	20220608
Time	9.49 h
INSTRUM	Avance Neo
PROBHD	Z167419_0029 (
PULPROG	zg30
TD	65536
SOLVENT	DMSO
NS	32
DS	2
SWH	10000.000 Hz
FIDRES	0.305176 Hz
AQ	3.2768500 sec
RG	101
DW	50.000 usec
DE	11.14 usec
TE	300.0 K
D1	1.00000000 sec
TD0	1
SFO1	500.1330883 MHz
NUC1	1H
P0	2.67 usec
P1	8.00 usec
SI	65536
SF	500.1300040 MHz
WDW	EM
SSB	0
LB	0.30 Hz
GB	0
PC	1.00

Openlynx Report
Vial:1:3
Date:08-Jun-2022
Name: Corey Arnett-[illegible]

ID:
Time:10:47:20
Date: 6/8/2022

File:ARN-E-6-2
Notebook: ARN-E-6-2

Printed: Wed Jun 08 10:49:33 2022

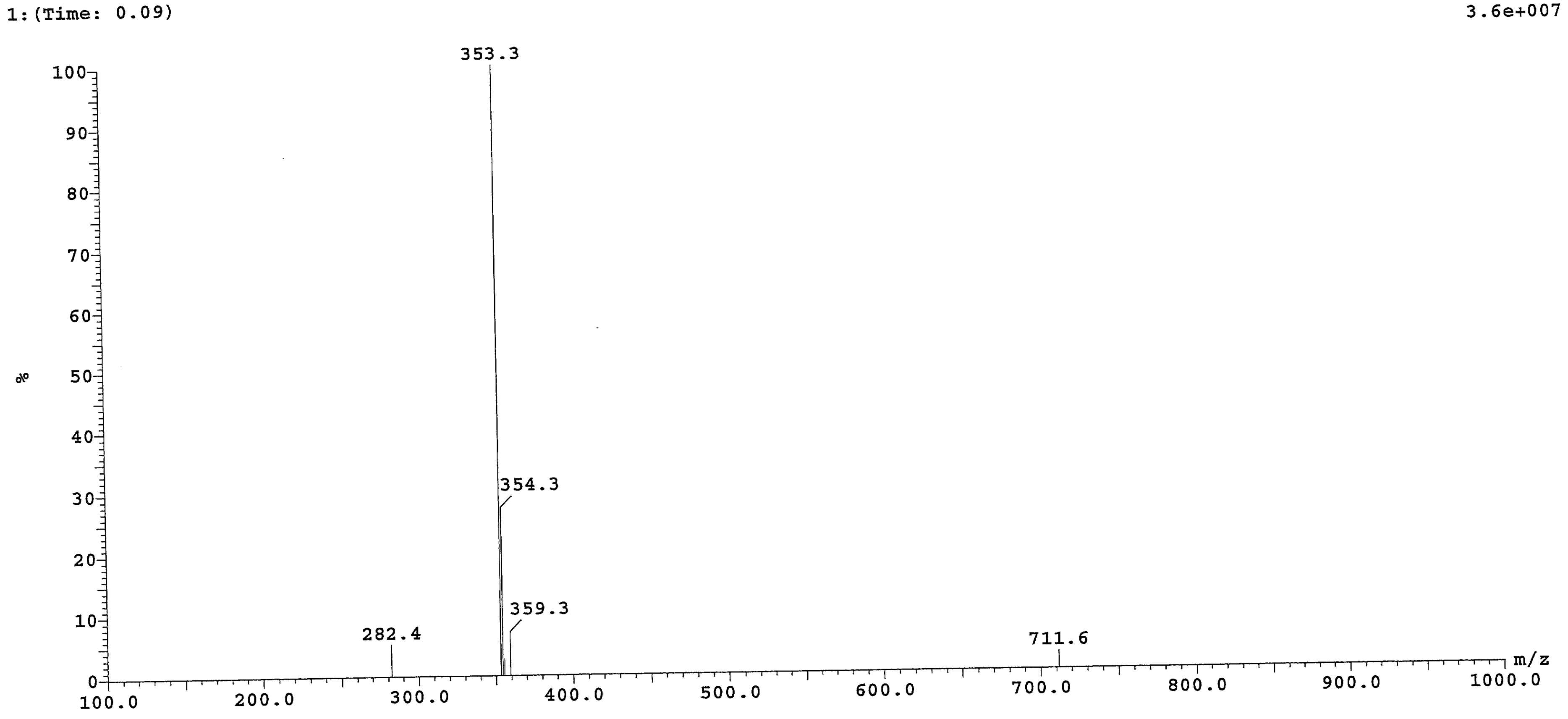

SAMPLE INFORMATION

Sample Name:	ARN-E-6-2
Injection Volume:	3.00 ul
Run Time:	9.0 Minutes
Date Acquired:	6/8/2022 10:01:14 AM EDT
Date Processed:	6/8/2022 10:43:47 AM EDT
Sample Set Name:	Template
Acq. Method Set:	BEH_C18_PDA_75mm 408
Processing Method:	BEH_C18_PDA_CAB
Channel Name:	254nm

Method Notes:
Acquity UPLC BEH C18 1.7u (2.1x75mm)
Flow Rate : 0.5 mL/min
Solvent A : 0.1% TFA in Waters
Solvent B : 0.1% TFA in Acetonitrile
Solvent Gradient Program:

Time (min)	%A	% B
0:00	95	5
6:00	0	100
8:00	0	100
9:00	95	5

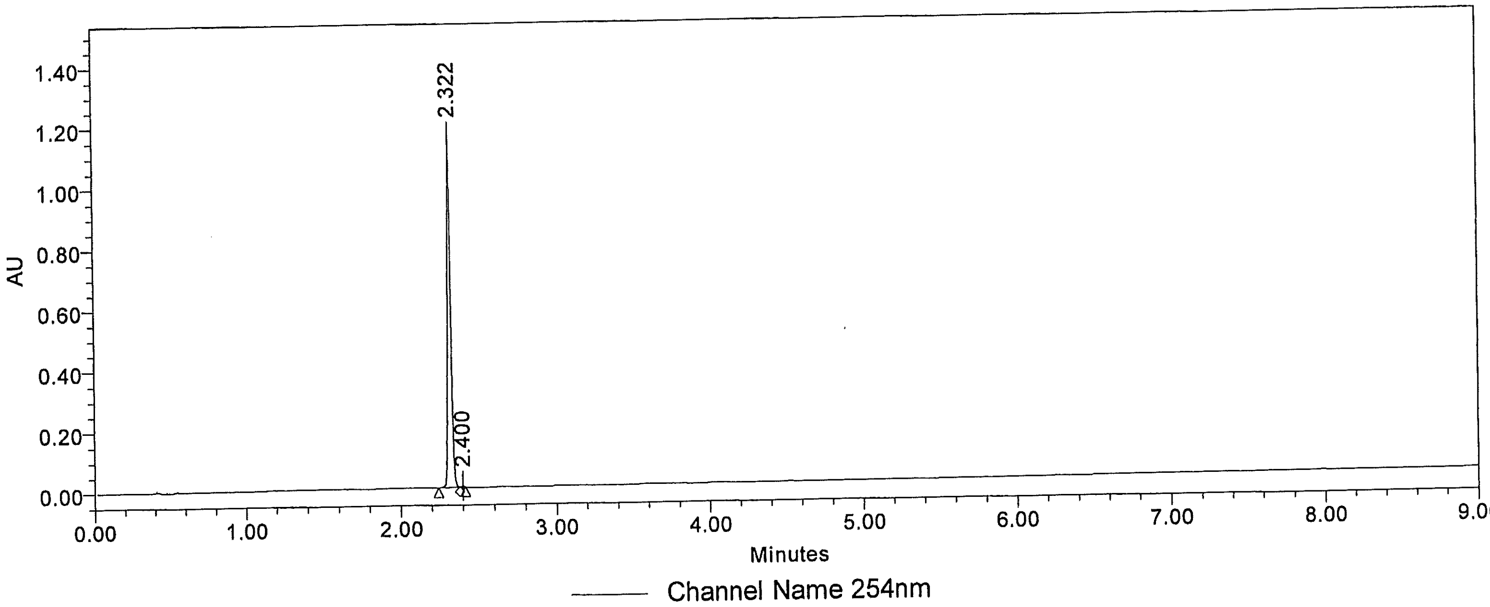

Peak Results

	RT	Area	Int Type	Width (sec)	% Area
1	2.322	1687311	bV	8.149	99.72
2	2.400	4693	Vb	2.150	0.28

Name: Cory Arnett-Butscher

Date: 6/8/2022

NB #: ARN-E-6-2

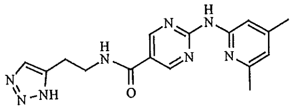

CERTIFICATE OF ANALYSIS

Compound Name: BPN-0035322-AA-001 1f
ALB Number: ALB-229929
Batch: 1
Lot Number: ALK-C-5-2
Molecular Formula: $C_{16}H_{18}N_8O$
Molecular Weight: 338.37
Last Solvent: Methylene Chloride, Methanol

TEST	RESULT/REFERENCE
Appearance	Off-white Solid
NMR Spectrum	^{1}H, 300 MHz, Dimethyl Sulfoxide-d_6, Consistent - Attached
Mass Spectrum	ESI, *m/z* 339 $[M + H]^+$, Attached
UPLC	98.1% (area %), ACQUITY UPLC BEH C18 (2.1 *75) mm, 1.7 micron Column, UV 254 nm Detection, Attached

[signature]
Approved By

6-8-2022
Date

For Research Purposes Only. Not Intended for Food or Drug Use.

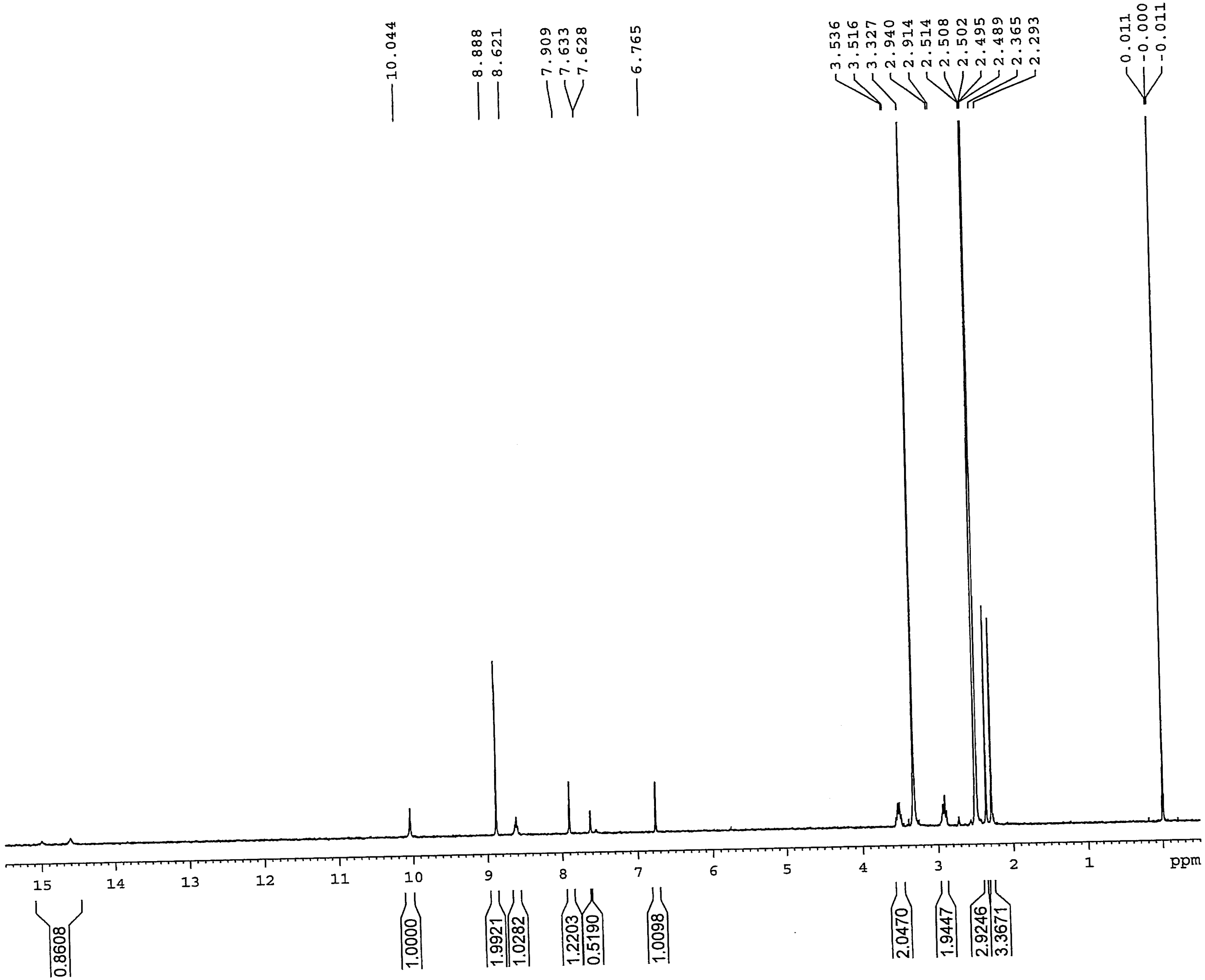

Name Marwah Albuker

Date 7 Jun 2022

NB # ALK-C-5-2

NAME	ALK-C-5-2
EXPNO	30
PROCNO	1
Date_	20220606
Time	8.47
INSTRUM	spect
PROBHD	5 mm QNP 1H/15
PULPROG	zg30
TD	65536
SOLVENT	DMSO
NS	32
DS	2
SWH	5995.204 Hz
FIDRES	0.091480 Hz
AQ	5.4657526 sec
RG	1149.4
DW	83.400 usec
DE	6.00 usec
TE	300.0 K
D1	1.00000000 sec
TD0	1

======== CHANNEL f1 ========

NUC1	1H
P1	12.88 usec
PL1	1.00 dB
PL1W	9.77678490 W
SFO1	300.1319509 MHz
SI	32768
SF	300.1300007 MHz
WDW	EM
SSB	0
LB	0.30 Hz
GB	0
PC	1.00

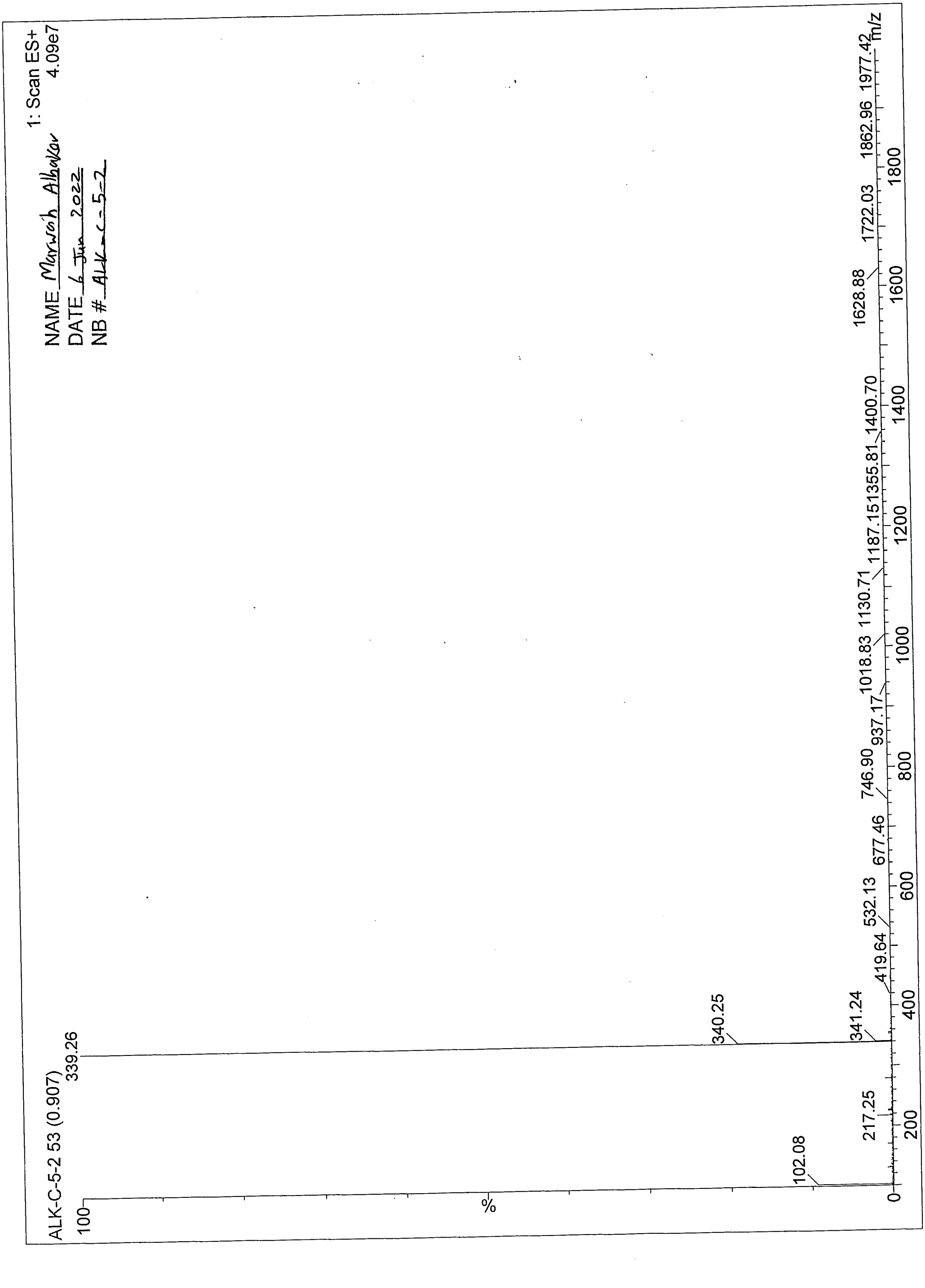

ALK-C-5-2 53 (0.907)
1: Scan ES+
4.09e7
NAME
DATE
NB #
100
%
0
339.26
340.25
341.24
102.08
217.25
419.64
532.13
677.46
746.90
937.17
1018.83
1130.71
1187.15
1355.81
1400.70
1628.88
1722.03
1862.96
1977.42
m/z
200
400
600
800
1000
1200
1400
1600
1800

SAMPLE INFORMATION

Sample Name:	ALK-C-5-2
Injection Volume:	3.00 ul
Run Time:	9.0 Minutes
Date Acquired:	6/3/2022 2:28:59 PM EDT
Date Processed:	6/3/2022 2:42:41 PM EDT
Sample Set Name:	Template
Acq. Method Set:	HSS T3_PDA_75mm_polar 408
Processing Method:	BEH_C18_PDA
Channel Name:	254nm

Method Notes:
Acquity UPLC HSS T3 1.8u (2.1x75mm)
Flow Rate : 0.5 mL/min
Solvent A : 0.1% TFA in Waters
Solvent B : 0.1% TFA in Acetonitrile
Solvent Gradient Program:

Time (min)	%A	% B
0:00	95	5
1:00	95	5
3:30	70	30
6:00	0	100
8:00	0	100
9:00	95	5

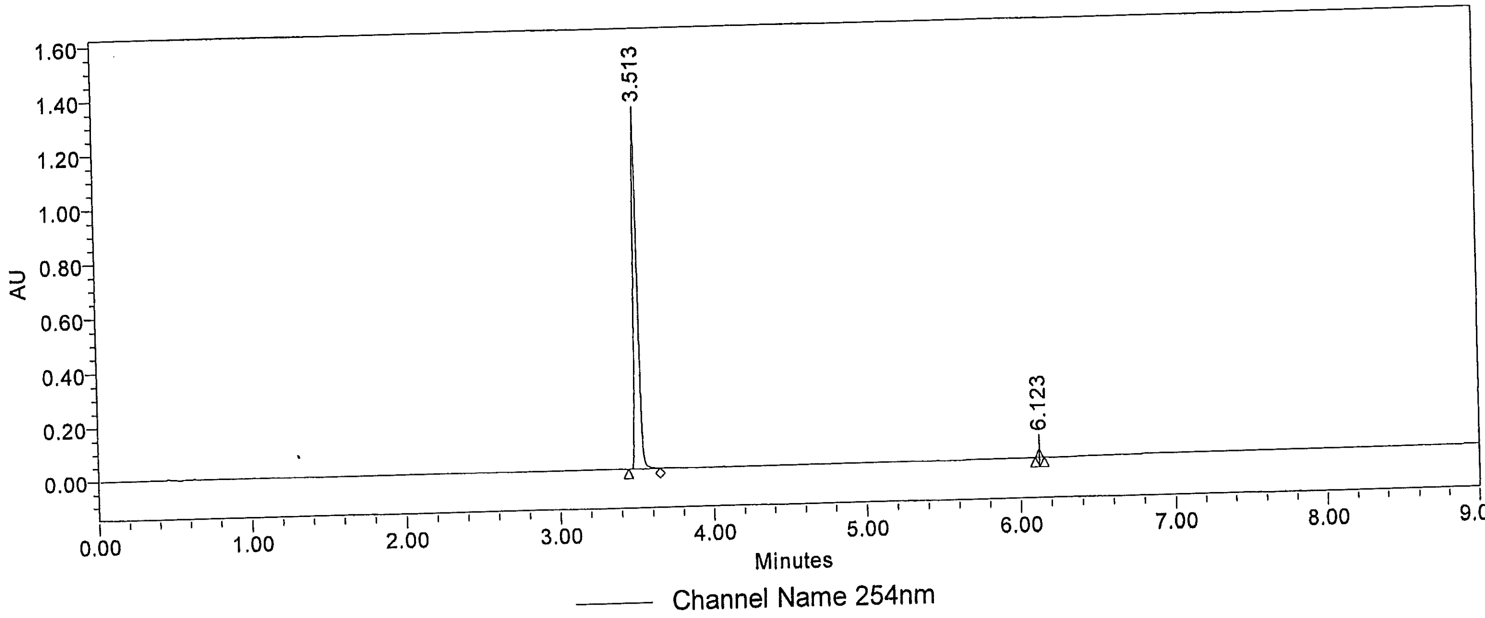

Peak Results

	RT	Area	Int Type	Width (sec)	% Area
1	3.513	2461152	BV	12.349	98.13
2	6.123	46969	bb	3.551	1.87

Name: Marwah Albaker

Date: 3 Jun 2022

NB #: ALK-C-5-2

CERTIFICATE OF ANALYSIS

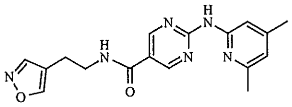

Compound Name: BPN-0035323-AA-001 1g
ALB Number: ALB-229930
Batch: 1
Lot Number: ALK-C-6-2
Molecular Formula: $C_{17}H_{18}N_6O_2$
Molecular Weight: 338.36
Last Solvent: Methylene Chloride, Methanol

TEST	RESULT/REFERENCE
Appearance	Off-white Solid
NMR Spectrum	^1H, 300 MHz, Dimethyl Sulfoxide-d_6, Attached
Mass Spectrum	ESI, *m/z* 339 $[M + H]^+$, Attached
UPLC	>99% (area %), ACQUITY UPLC BEH C18 (2.1 *75) mm, 1.7 micron Column, UV 254 nm Detection, Attached

Marian Maychack
Approved By

6-8-2022
Date

For Research Purposes Only. Not Intended for Food or Drug Use.

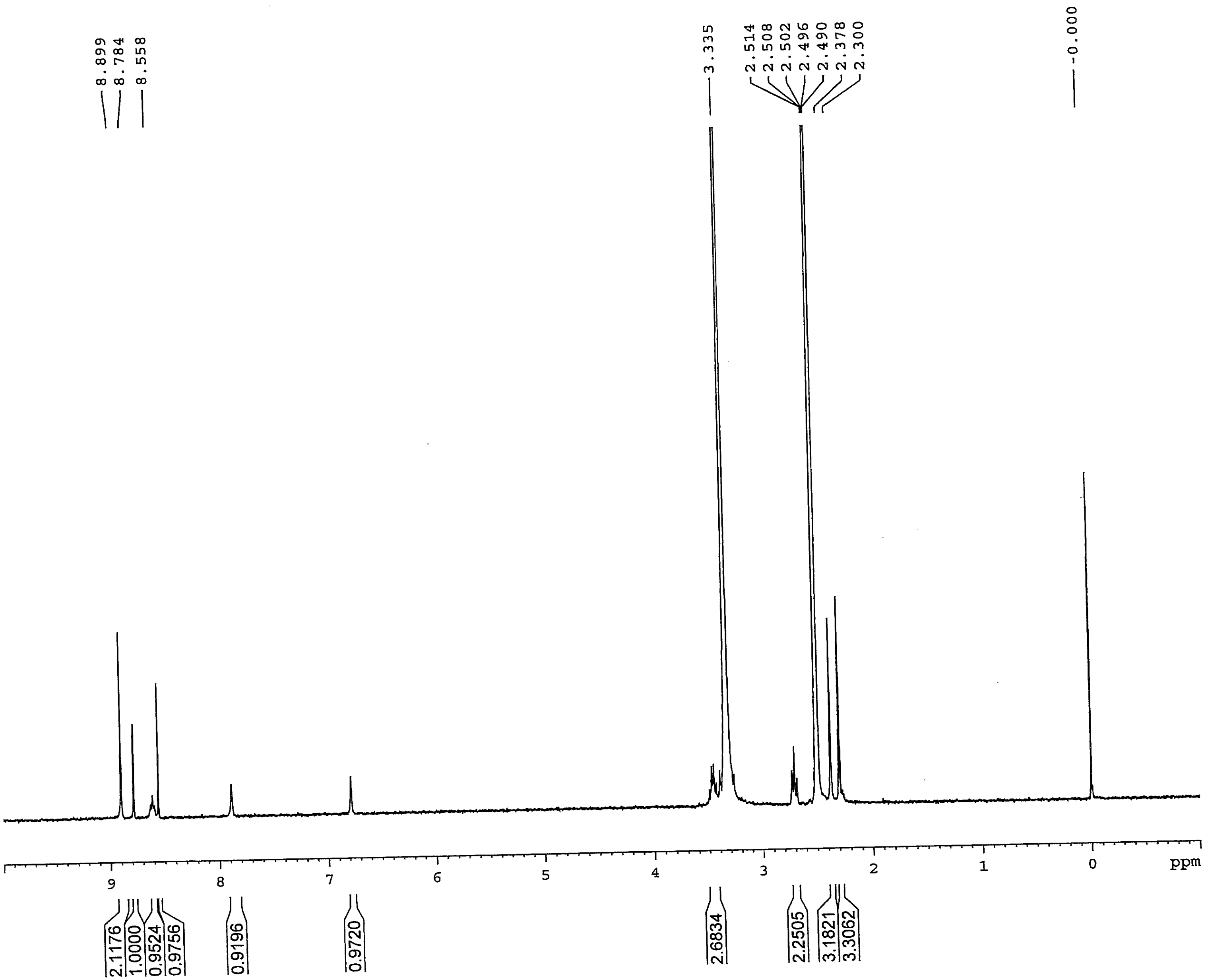

Name Marwah Albaker
Date 6 Jun 2022
NB # ALK-C-6-2

NAME	ALK-C-6-2
EXPNO	10
PROCNO	1
Date_	20220606
Time	8.53
INSTRUM	spect
PROBHD	5 mm QNP 1H/15
PULPROG	zg30
TD	65536
SOLVENT	DMSO
NS	32
DS	2
SWH	5995.204 Hz
FIDRES	0.091480 Hz
AQ	5.4657526 sec
RG	812.7
DW	83.400 usec
DE	6.00 usec
TE	300.0 K
D1	1.00000000 sec
TD0	1

======== CHANNEL f1 ========

NUC1	1H
P1	12.88 usec
PL1	1.00 dB
PL1W	9.77678490 W
SFO1	300.1319509 MHz
SI	32768
SF	300.1300005 MHz
WDW	EM
SSB	0
LB	0.30 Hz
GB	0
PC	1.00

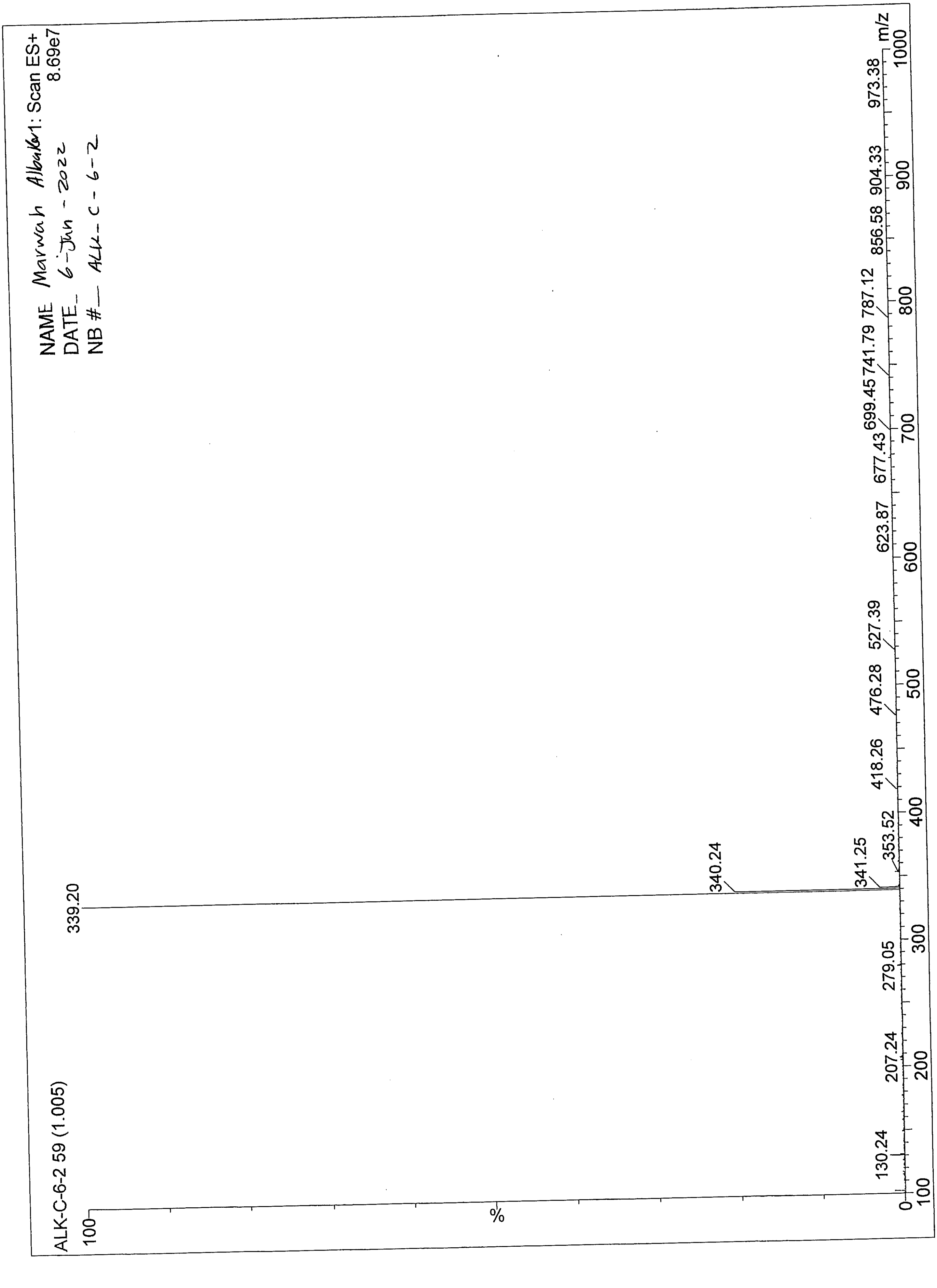
ALK-C-6-2 59 (1.005)
1: Scan ES+
8.69e7
NAME Marwah Albakri
DATE_ 6-Jun-2022
NB #_ ALK-C-6-2
100
%
0
339.20
340.24
341.25
353.52
130.24
207.24
279.05
418.26
476.28
527.39
623.87
677.43
699.45
741.79
787.12
856.58
904.33
973.38
100
200
300
400
500
600
700
800
900
1000
m/z

SAMPLE INFORMATION

Sample Name:	ALK-C-6-2
Injection Volume:	3.00 ul
Run Time:	9.0 Minutes
Date Acquired:	6/6/2022 9:43:10 AM EDT
Date Processed:	6/6/2022 9:55:12 AM EDT
Sample Set Name:	Template
Acq. Method Set:	HSS T3_PDA_75mm_polar 408
Processing Method:	BEH_C18_PDA
Channel Name:	254nm

Method Notes:
Acquity UPLC HSS T3 1.8u (2.1x75mm)
Flow Rate : 0.5 mL/min
Solvent A : 0.1% TFA in Waters
Solvent B : 0.1% TFA in Acetonitrile
Solvent Gradient Program:

Time (min)	%A	% B
0:00	95	5
1:00	95	5
3:30	70	30
6:00	0	100
8:00	0	100
9:00	95	5

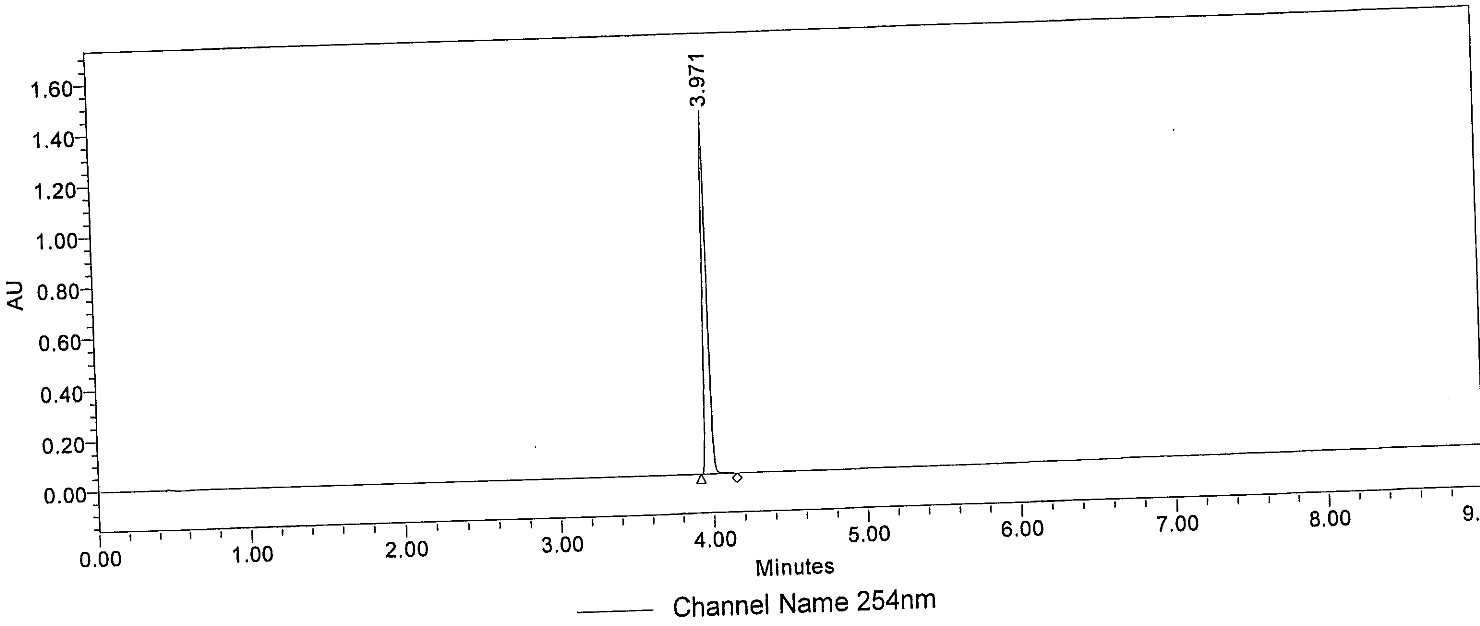

Peak Results

	RT	Area	Int Type	Width (sec)	% Area
1	3.971	2753053	BV	13.902	100.00

Name: Marwah Albaker

Date: 6-Jun-2022

NB #: ALK-C-6-2

CERTIFICATE OF ANALYSIS

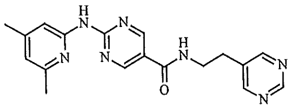

Compound Name: BPN-0035324-AA-001 1h
ALB Number: ALB-229931
Batch: 1
Lot Number: ARN-E-3-2
Molecular Formula: $C_{18}H_{19}N_7O$
Molecular Weight: 349.39
Last Solvent: Methylene Chloride

TEST	RESULT/REFERENCE
Appearance	Off-white Solid
NMR Spectrum	^1H, 500 MHz, Dimethyl Sulfoxide-d_6, Consistent - Attached
Mass Spectrum	ESI, *m/z* 350 $[M + H]^+$, Attached
UPLC	95.1% (area %), ACQUITY UPLC BEH C18 (2.1 *75) mm, 1.7 micron Column, UV 254 nm Detection, Attached

Maura Maychack
Approved By

6-8-2022
Date

For Research Purposes Only. Not Intended for Food or Drug Use.

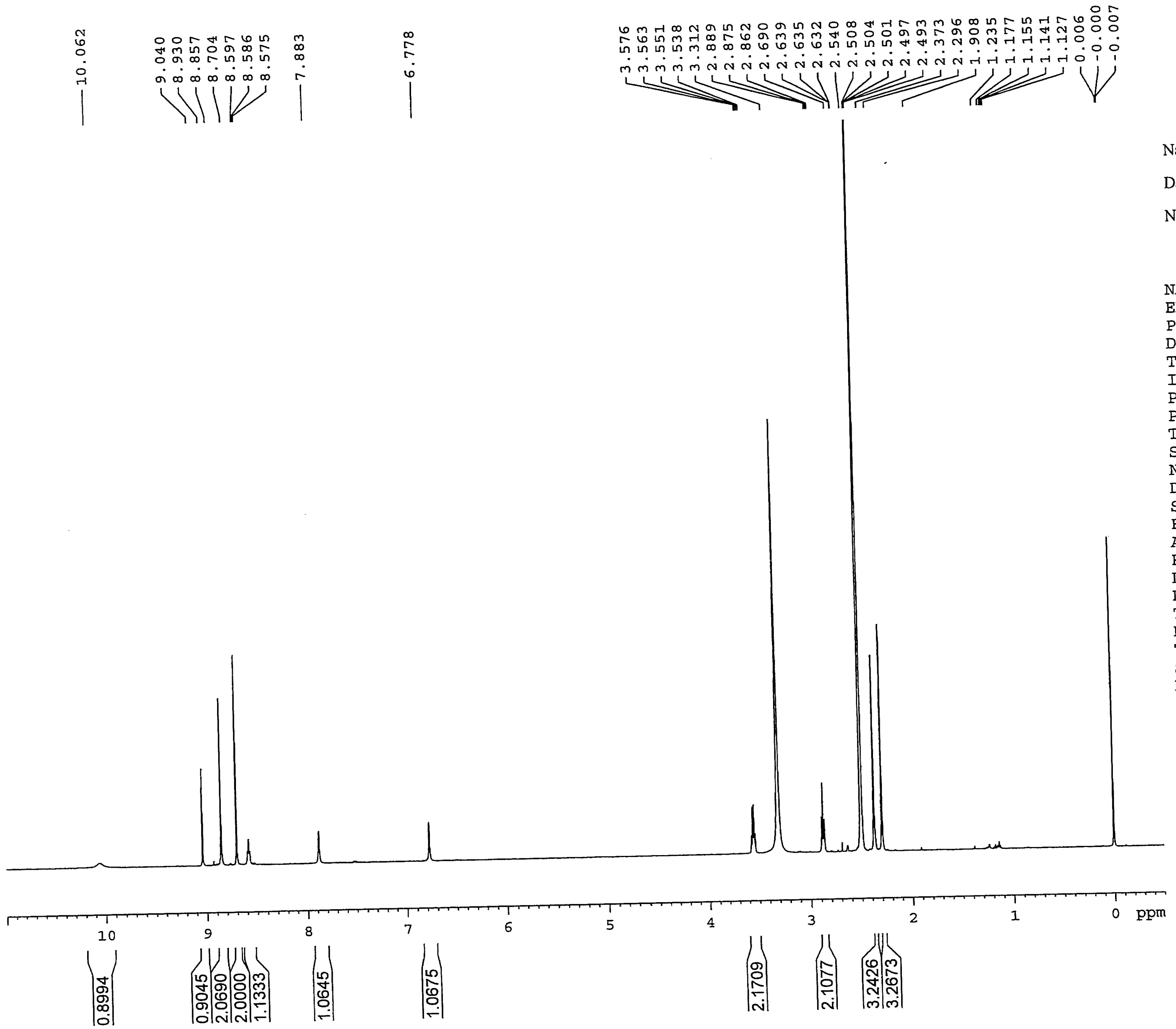

Name Corey Arnett-Butscher

Date 6/6/2022

NB # ARN-E-3-2

NAME	ARN-E-3-2
EXPNO	10
PROCNO	1
Date_	20220606
Time	8.09 h
INSTRUM	Avance Neo
PROBHD	Z167419_0029 (
PULPROG	zg30
TD	65536
SOLVENT	DMSO
NS	32
DS	2
SWH	10000.000 Hz
FIDRES	0.305176 Hz
AQ	3.2768500 sec
RG	101
DW	50.000 usec
DE	11.14 usec
TE	300.0 K
D1	1.00000000 sec
TD0	1
SFO1	500.1330883 MHz
NUC1	1H
P0	2.67 usec
P1	8.00 usec
SI	65536
SF	500.1300040 MHz
WDW	EM
SSB	0
LB	0.30 Hz
GB	0
PC	1.00

Openlynx Report
Vial:1:16
Date:06-Jun-2022
Name: Corey Arnett-Butscher

ID:
Time:11:00:00
Date: 6/6/2022

File:ARN-E-3-2
Notebook: ARN-E-3-2

Printed: Mon Jun 06 11:02:23 2022

1:(Time: 0.09)

1:MS ES+
1.4e+007

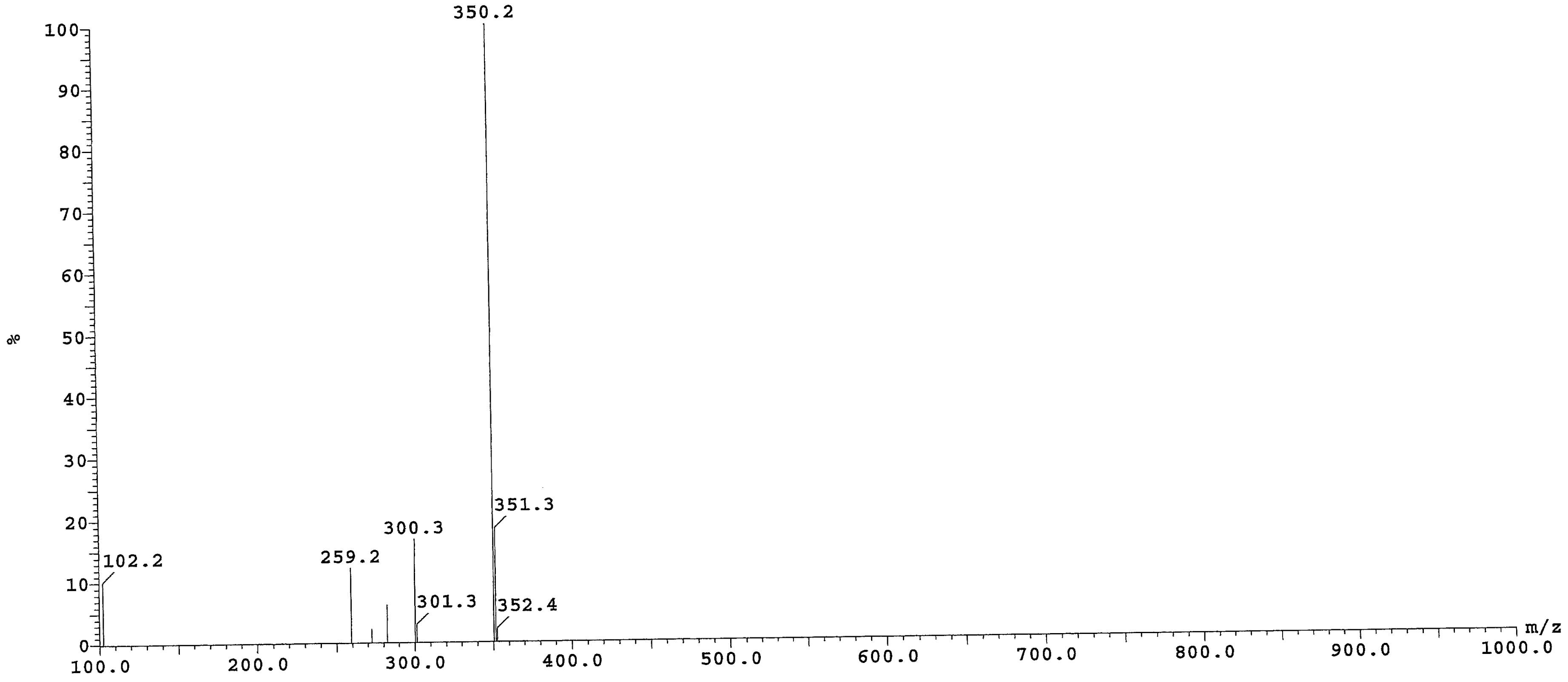

SAMPLE INFORMATION

Sample Name:	ARN-E-3-2
Injection Volume:	3.00 ul
Run Time:	9.0 Minutes
Date Acquired:	6/6/2022 9:56:11 AM EDT
Date Processed:	6/6/2022 10:57:22 AM EDT
Sample Set Name:	Template
Acq. Method Set:	BEH_C18_PDA_75mm 408
Processing Method:	BEH_C18_PDA_CAB
Channel Name:	254nm

Method Notes:
Acquity UPLC BEH C18 1.7u (2.1x75mm)
Flow Rate : 0.5 mL/min
Solvent A : 0.1% TFA in Waters
Solvent B : 0.1% TFA in Acetonitrile
Solvent Gradient Program:

Time (min)	%A	% B
0:00	95	5
6:00	0	100
8:00	0	100
9:00	95	5

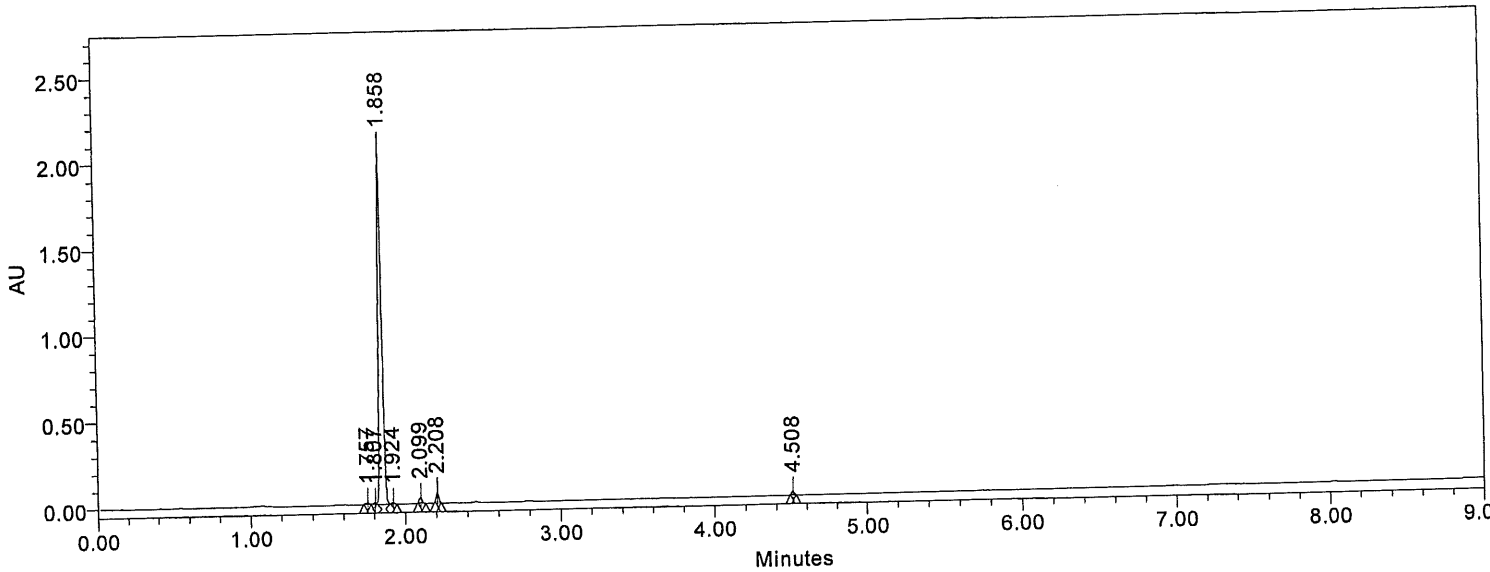

Peak Results

	RT	Area	Int Type	Width (sec)	% Area
1	1.757	11597	bB	2.750	0.35
2	1.807	12170	BV	2.350	0.36
3	1.858	3189528	VV	5.200	95.14
4	1.924	13813	Vb	2.500	0.41
5	2.099	37226	bb	3.000	1.11
6	2.208	65192	bb	2.300	1.94
7	4.508	23001	bb	2.250	0.69

Name: Corey Arnett-Butscher

Date: 6/6/2022

NB #: ARN-E-3-2

CERTIFICATE OF ANALYSIS

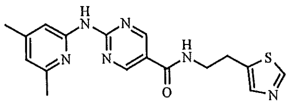

Compound Name: BPN-0035325-AA-001 1i
ALB Number: ALB-229932
Batch: 1
Lot Number: ARN-E-4-1
Molecular Formula: $C_{17}H_{18}N_6OS$
Molecular Weight: 354.43
Last Solvent: Ethyl Acetate

TEST	RESULT/REFERENCE
Appearance	Off-white Solid
NMR Spectrum	^{1}H, 500 MHz, Dimethyl Sulfoxide-d_6, Consistent - Attached
Mass Spectrum	ESI, *m/z* 355 $[M + H]^+$, Attached
UPLC	98.6% (area %), ACQUITY UPLC BEH C18 (2.1 *75) mm, 1.7 micron Column, UV 254 nm Detection, Attached

Maria Mayxhach

Approved By

6-8-2022

Date

For Research Purposes Only. Not Intended for Food or Drug Use.

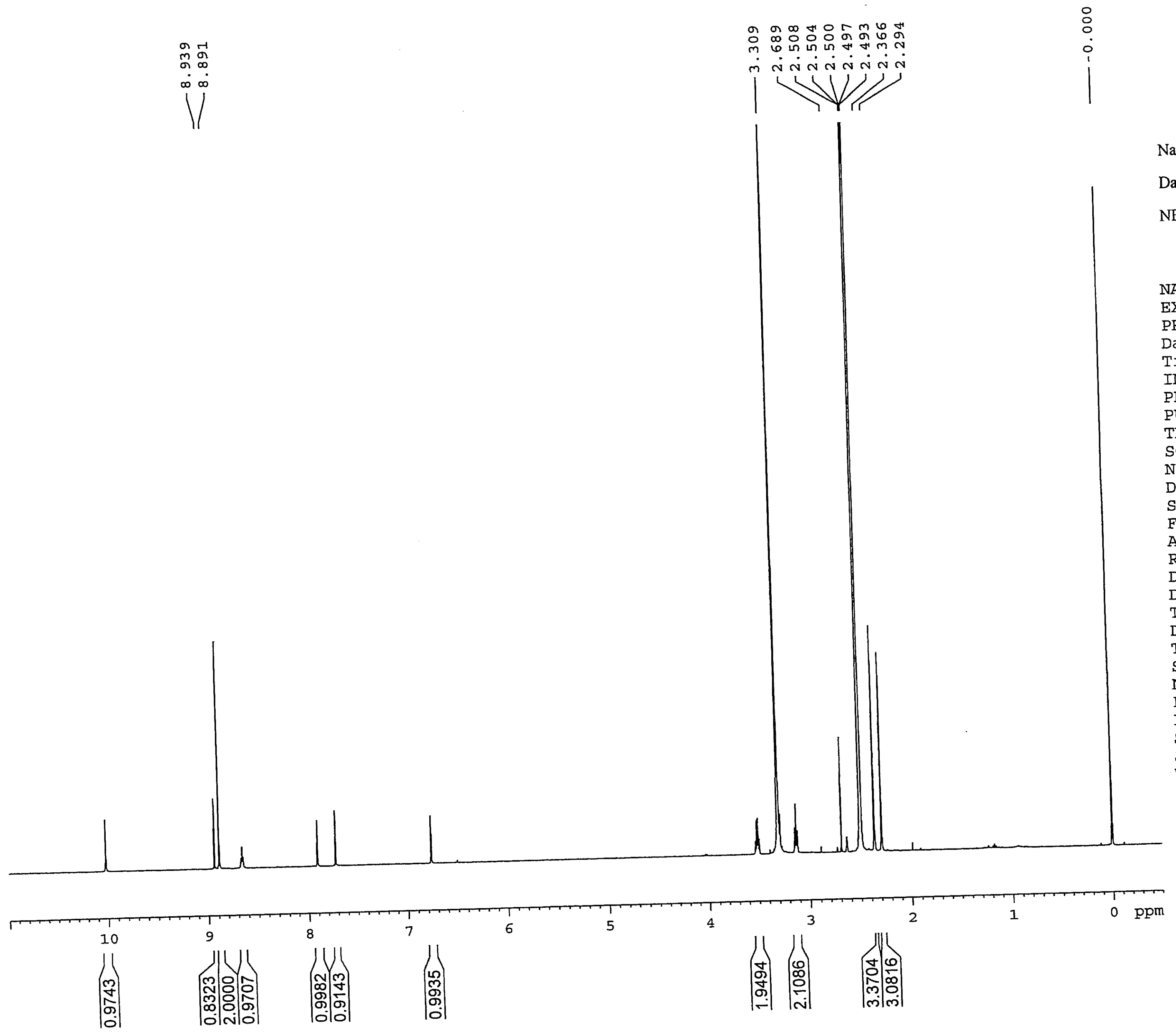

Name Corey Arnett-Butscher
Date 6/6/2022
NB # ARN-E-4-1

NAME	ARN-E-4-1
EXPNO	10
PROCNO	1
Date_	20220606
Time	8.16 h
INSTRUM	Avance Neo
PROBHD	Z167419_0029 (
PULPROG	zg30
TD	65536
SOLVENT	DMSO
NS	32
DS	2
SWH	10000.000 Hz
FIDRES	0.305176 Hz
AQ	3.2768500 sec
RG	101
DW	50.000 usec
DE	11.14 usec
TE	300.0 K
D1	1.00000000 sec
TD0	1
SFO1	500.1330883 MHz
NUC1	1H
P0	2.67 usec
P1	8.00 usec
SI	65536
SF	500.1300040 MHz
WDW	EM
SSB	0
LB	0.30 Hz
GB	0
PC	1.00

Openlynx Report
Vial:1:32
Date:03-Jun-2022
Name: Corey Arnett-Butscher

ID:
Time:11:01:46
Date: 6/6/2022

File:ARN-E-4-1
Notebook: ARN-E-4-1

Printed: Fri Jun 03 11:04:05 2022

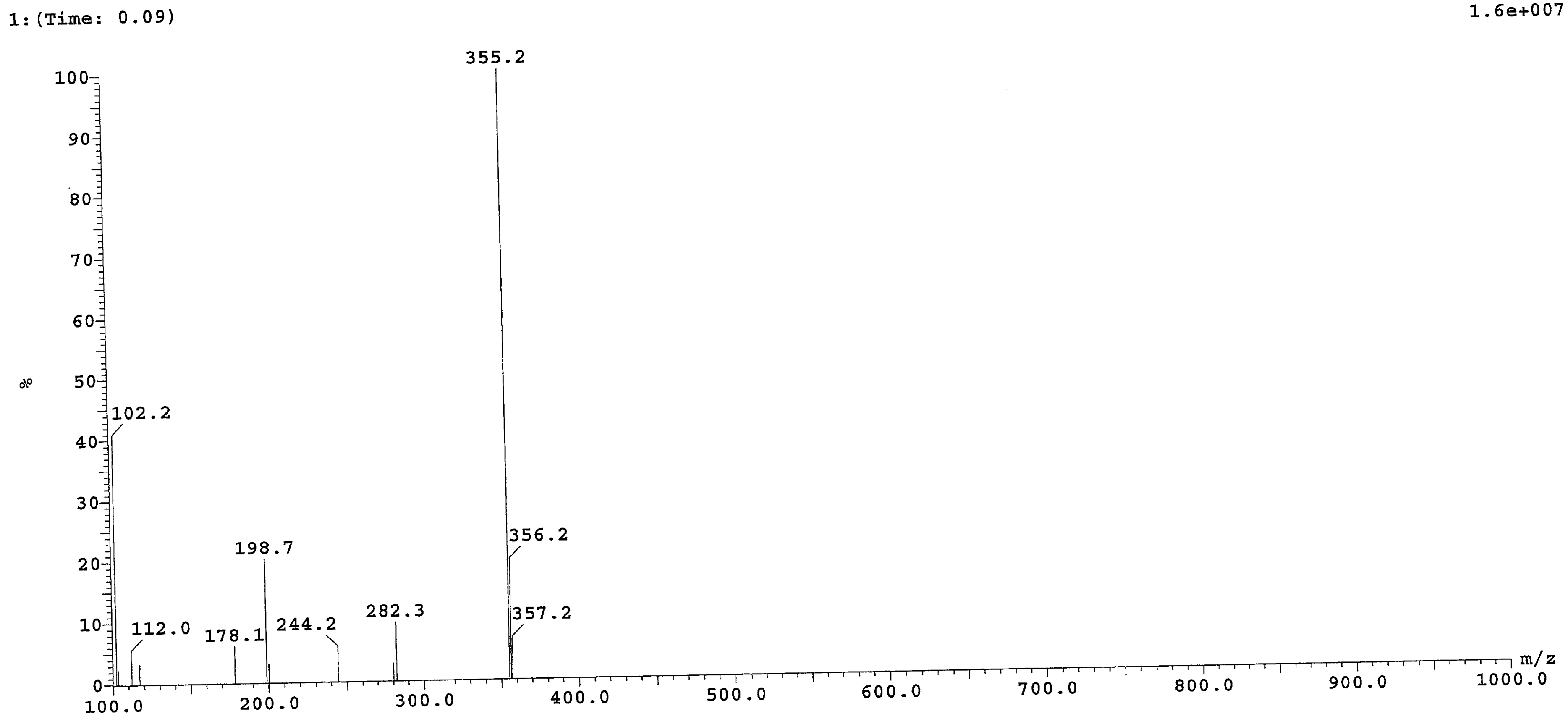

SAMPLE INFORMATION

Sample Name:	ARN-E-4-1
Injection Volume:	3.00 ul
Run Time:	9.0 Minutes
Date Acquired:	6/3/2022 10:48:48 AM EDT
Date Processed:	6/3/2022 11:15:28 AM EDT
Sample Set Name:	Template
Acq. Method Set:	BEH_C18_PDA_75mm 408
Processing Method:	BEH_C18_PDA_CAB
Channel Name:	254nm

Method Notes:
Acquity UPLC BEH C18 1.7u (2.1x75mm)
Flow Rate : 0.5 mL/min
Solvent A : 0.1% TFA in Waters
Solvent B : 0.1% TFA in Acetonitrile
Solvent Gradient Program:

Time (min)	%A	% B
0:00	95	5
6:00	0	100
8:00	0	100
9:00	95	5

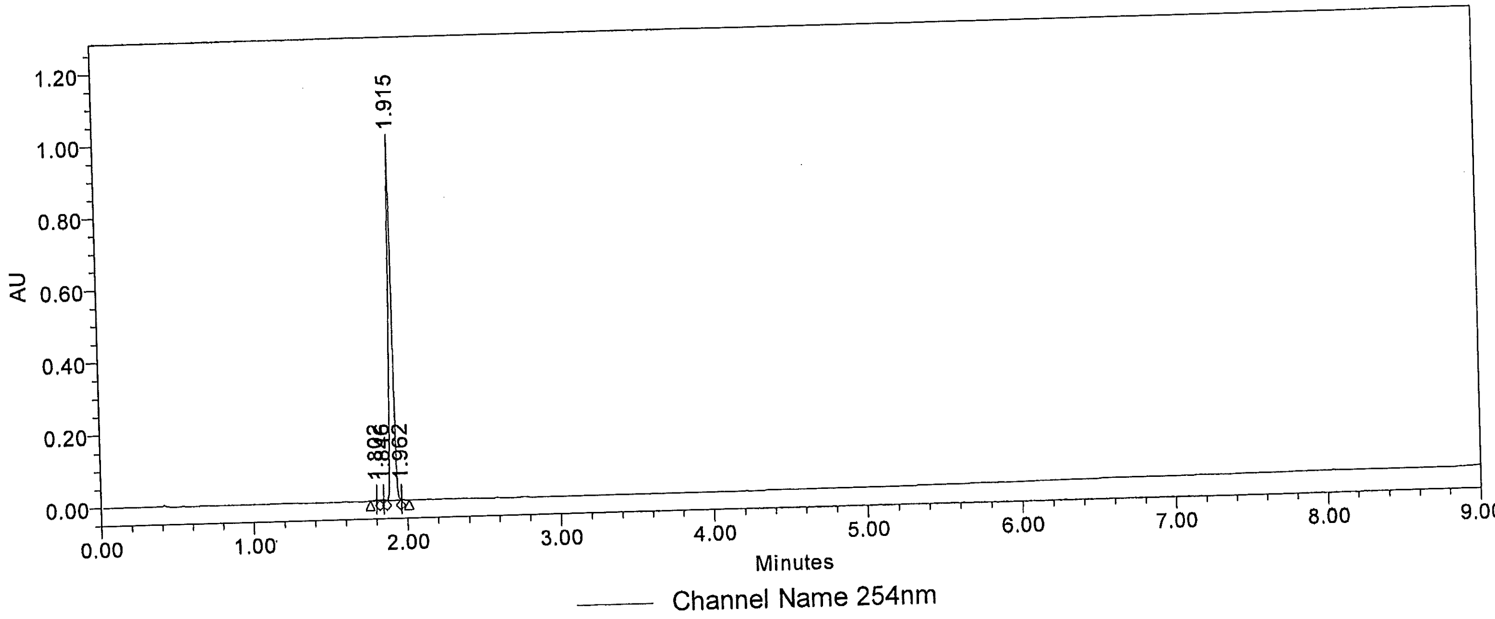

Peak Results

	RT	Area	Int Type	Width (sec)	% Area
1	1.802	7822	bV	3.750	0.52
2	1.846	7594	VV	2.700	0.50
3	1.915	1496001	VV	5.450	98.63
4	1.962	5424	VB	3.050	0.36

Name: Corey Arndt-Betcher

Date: 6/6/2022

NB #: ARN-E-4-1

CERTIFICATE OF ANALYSIS

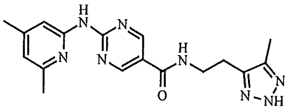

Compound Name: BPN-0035344-AA-001 1j
ALB Number: ALB-230037
Batch: 1
Lot Number: ARN-E-5-2
Molecular Formula: $C_{17}H_{20}N_8O$
Molecular Weight: 352.39
Last Solvent: Methylene Chloride

TEST	RESULT/REFERENCE
Appearance	Off-white Solid
NMR Spectrum	^1H, 500 MHz, Dimethyl Sulfoxide-d_6, Consistent - Attached
Mass Spectrum	ESI, *m/z* 351 $[M-H]^-$, Attached
UPLC	98.2% (area %), ACQUITY UPLC BEH C18 (2.1 *75) mm, 1.7 micron Column, UV 254 nm Detection, Attached

Maria Mayrhach
Approved By

6-15-2022
Date

For Research Purposes Only. Not Intended for Food or Drug Use.

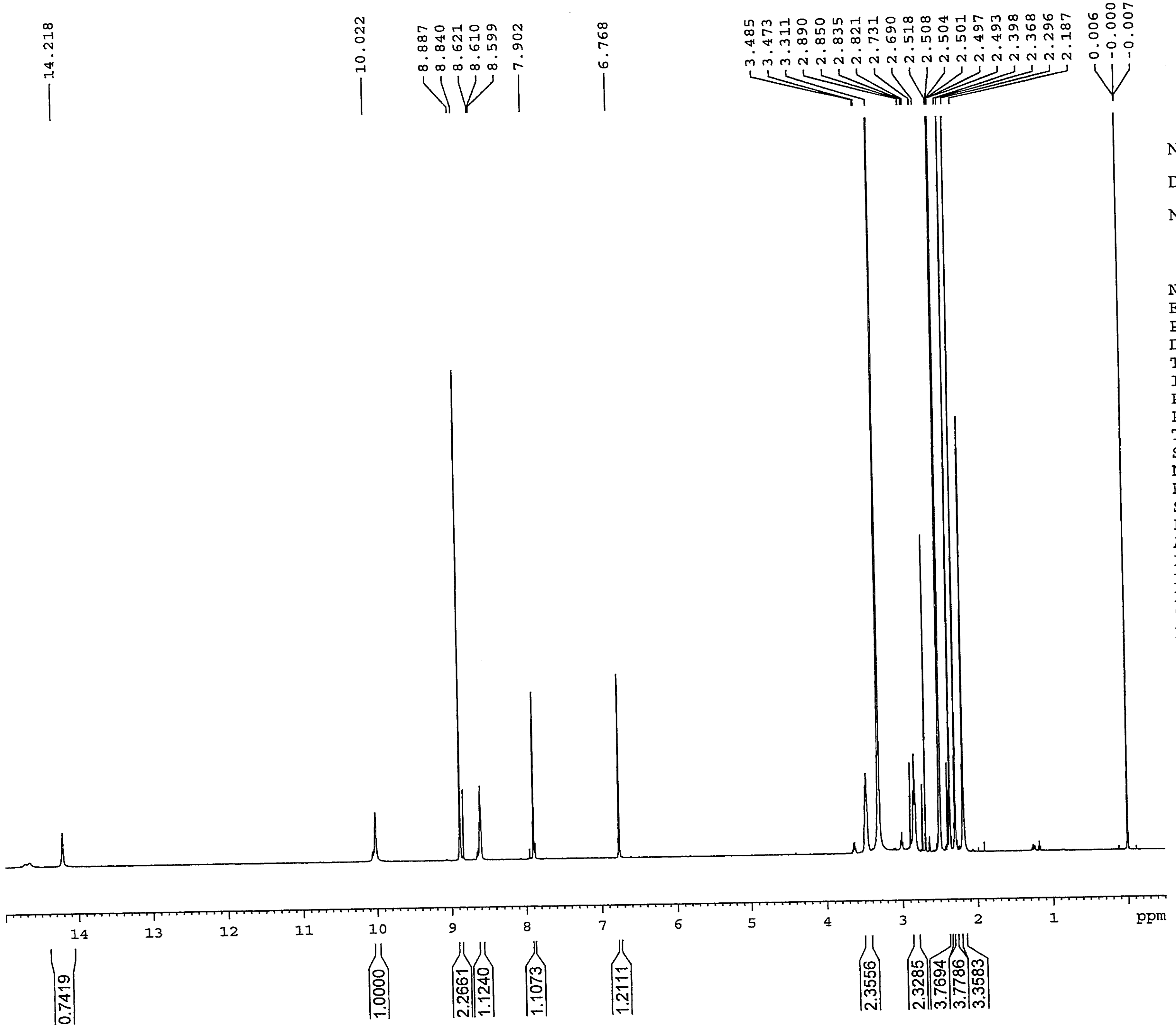

NAME	ARN-E-5-2
EXPNO	10
PROCNO	1
Date_	20220608
Time_	9.14 h
INSTRUM	Avance Neo
PROBHD	Z167419_0029 (
PULPROG	zg30
TD	65536
SOLVENT	DMSO
NS	32
DS	2
SWH	10000.000 Hz
FIDRES	0.305176 Hz
AQ	3.2768500 sec
RG	101
DW	50.000 usec
DE	11.14 usec
TE	300.0 K
D1	1.00000000 sec
TD0	1
SFO1	500.1330883 MHz
NUC1	1H
P0	2.67 usec
P1	8.00 usec
SI	65536
SF	500.1300040 MHz
WDW	EM
SSB	0
LB	0.30 Hz
GB	0
PC	1.00

Openlynx Report
Vial:2:6
Date:08-Jun-2022

ID:
Time:10:01:32

File:ARN-E-5-2

Name: Cory Arnett-Butscher

Date: 6/8/2022

Notebook: ARN-E-5-2

Printed: Wed Jun 08 10:03:31 2022

3:(Time: 0.09) Combine (1:4)

2:MS ES-
5.9e+005

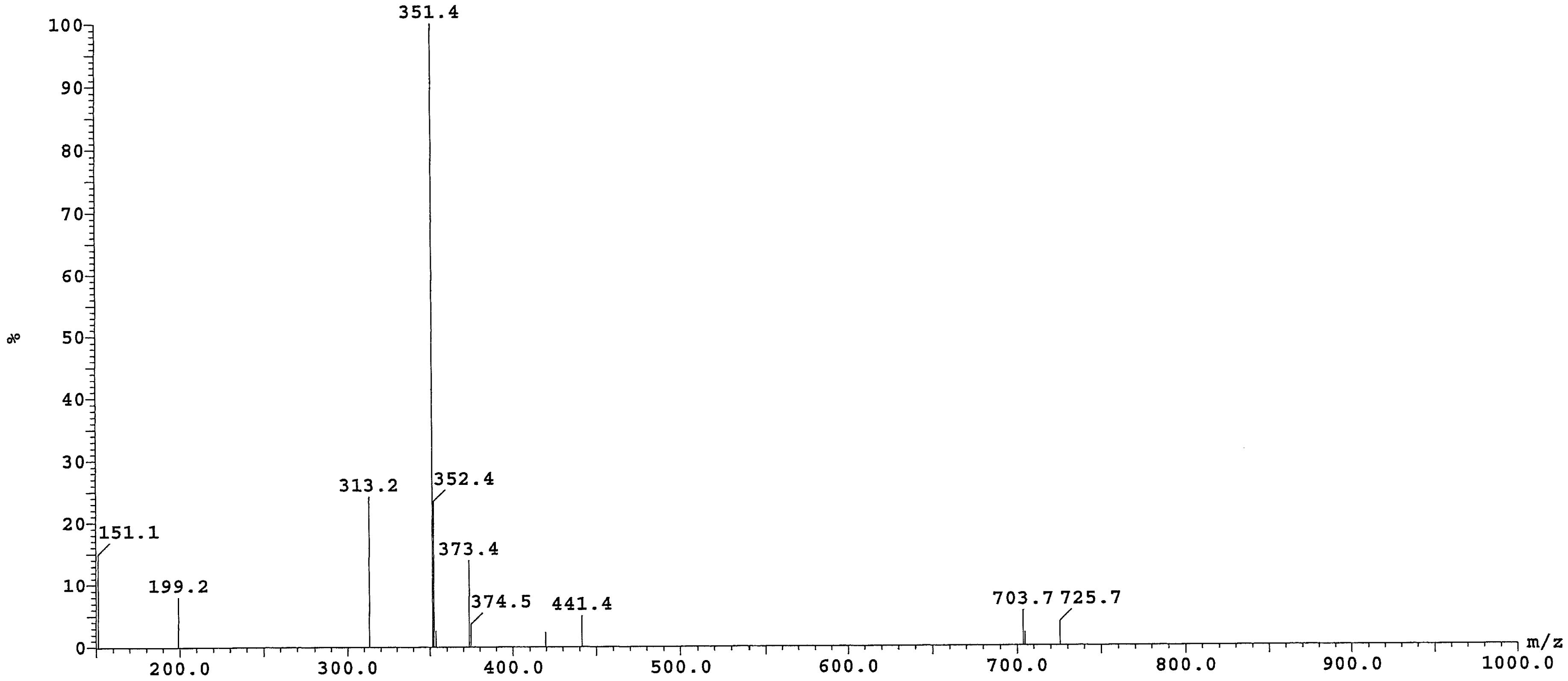

SAMPLE INFORMATION

Sample Name:	ARN-E-5-2
Injection Volume:	3.00 ul
Run Time:	9.0 Minutes
Date Acquired:	6/7/2022 7:34:21 AM EDT
Date Processed:	6/8/2022 9:52:12 AM EDT
Sample Set Name:	Template
Acq. Method Set:	BEH_C18_PDA_75mm 408
Processing Method:	BEH_C18_PDA_CAB
Channel Name:	254nm

Method Notes:
Acquity UPLC BEH C18 1.7u (2.1x75mm)
Flow Rate : 0.5 mL/min
Solvent A : 0.1% TFA in Waters
Solvent B : 0.1% TFA in Acetonitrile
Solvent Gradient Program:

Time (min)	%A	% B
0:00	95	5
6:00	0	100
8:00	0	100
9:00	95	5

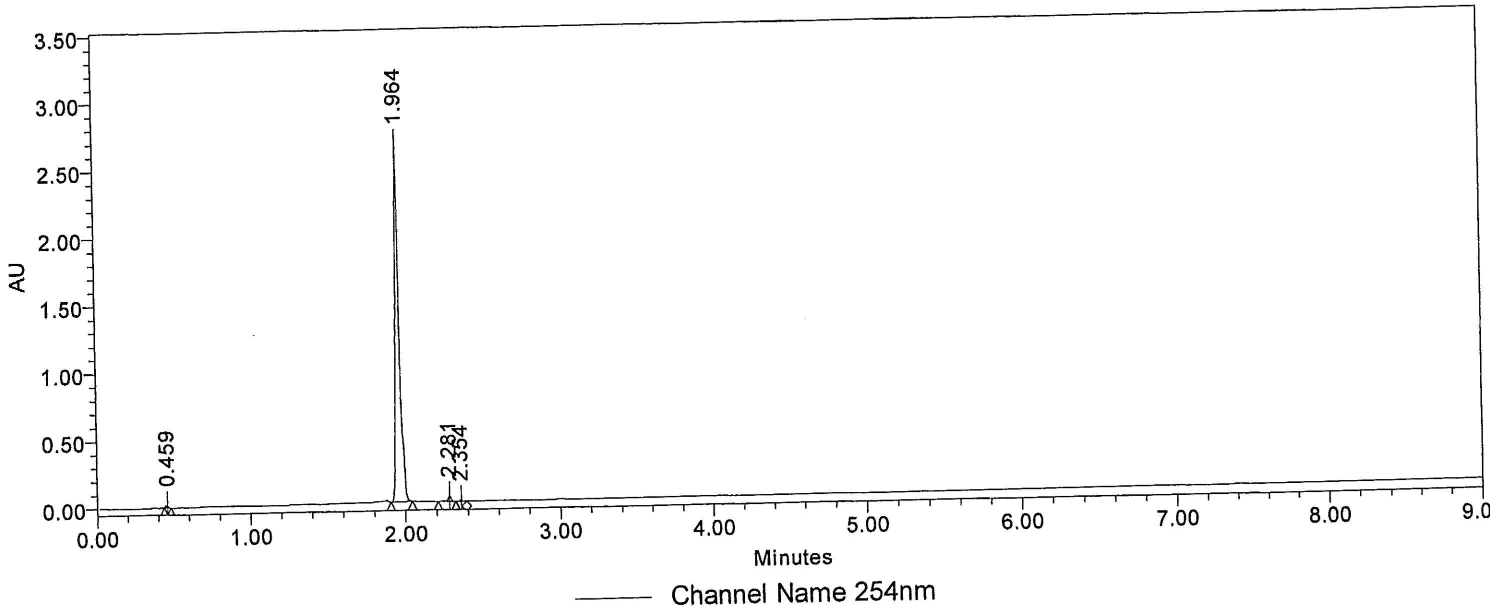

Peak Results

	RT	Area	Int Type	Width (sec)	% Area
1	0.459	20879	bb	2.550	0.43
2	1.964	4822746	bb	8.200	98.27
3	2.281	53716	BB	6.750	1.09
4	2.354	10554	BV	4.200	0.22

Name: Corey Arnett-Butscher

Date: 6/8/2022

NB #: ARN-E-5-2

CERTIFICATE OF ANALYSIS

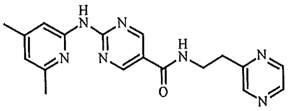

Compound Name: BPN-0035347-AA-001 1k
ALB Number: ALB-230040
Batch: 1
Lot Number: ARN-E-9-1
Molecular Formula: $C_{18}H_{19}N_7O$
Molecular Weight: 349.39
Last Solvent: Water, Ethyl Acetate

TEST	RESULT/REFERENCE
Appearance	Off-white Solid
NMR Spectrum	^{1}H, 500 MHz, Dimethyl Sulfoxide-d_6, Consistent - Attached
Mass Spectrum	ESI, m/z 350 $[M + H]^+$, Attached
UPLC	>99% (area %), ACQUITY UPLC BEH C18 (2.1 *75) mm, 1.7 micron Column, UV 254 nm Detection, Attached

[signature]

Approved By

6-15-2022

Date

For Research Purposes Only. Not Intended for Food or Drug Use.

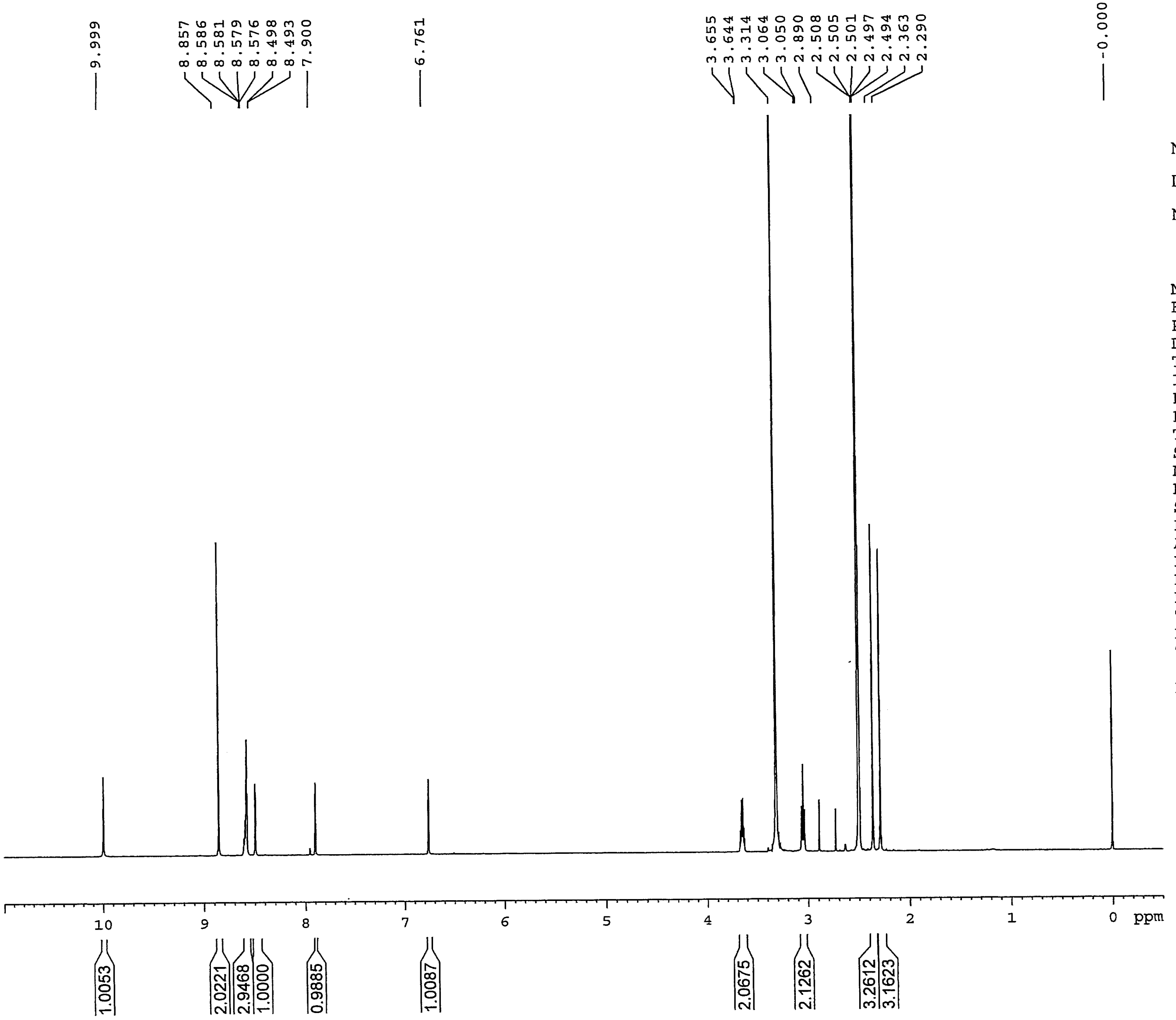

Name Cory Arnett-Butscher

Date 6/9/2022

NB # ARN-E-9-1

NAME	ARN-E-9-1	
EXPNO	10	
PROCNO	1	
Date_	20220609	
Time	7.38	h
INSTRUM	Avance Neo	
PROBHD	Z167419_0029 (	
PULPROG	zg30	
TD	65536	
SOLVENT	DMSO	
NS	32	
DS	2	
SWH	10000.000	Hz
FIDRES	0.305176	Hz
AQ	3.2768500	sec
RG	101	
DW	50.000	usec
DE	11.14	usec
TE	300.0	K
D1	1.00000000	sec
TD0	1	
SFO1	500.1330883	MHz
NUC1	1H	
P0	2.67	usec
P1	8.00	usec
SI	65536	
SF	500.1300038	MHz
WDW	EM	
SSB	0	
LB	0.30	Hz
GB	0	
PC	1.00	

Openlynx Report
Vial:1:19
Date:09-Jun-2022
Name: CoryArnett-Butscher

ID:
Time:09:31:05
Date: 6/9/2022

File:ARN-E-9-1
Notebook: ARN-E-9-1

Printed: Thu Jun 09 09:33:17 2022

1:(Time: 0.09)

1:MS ES+
3.2e+007

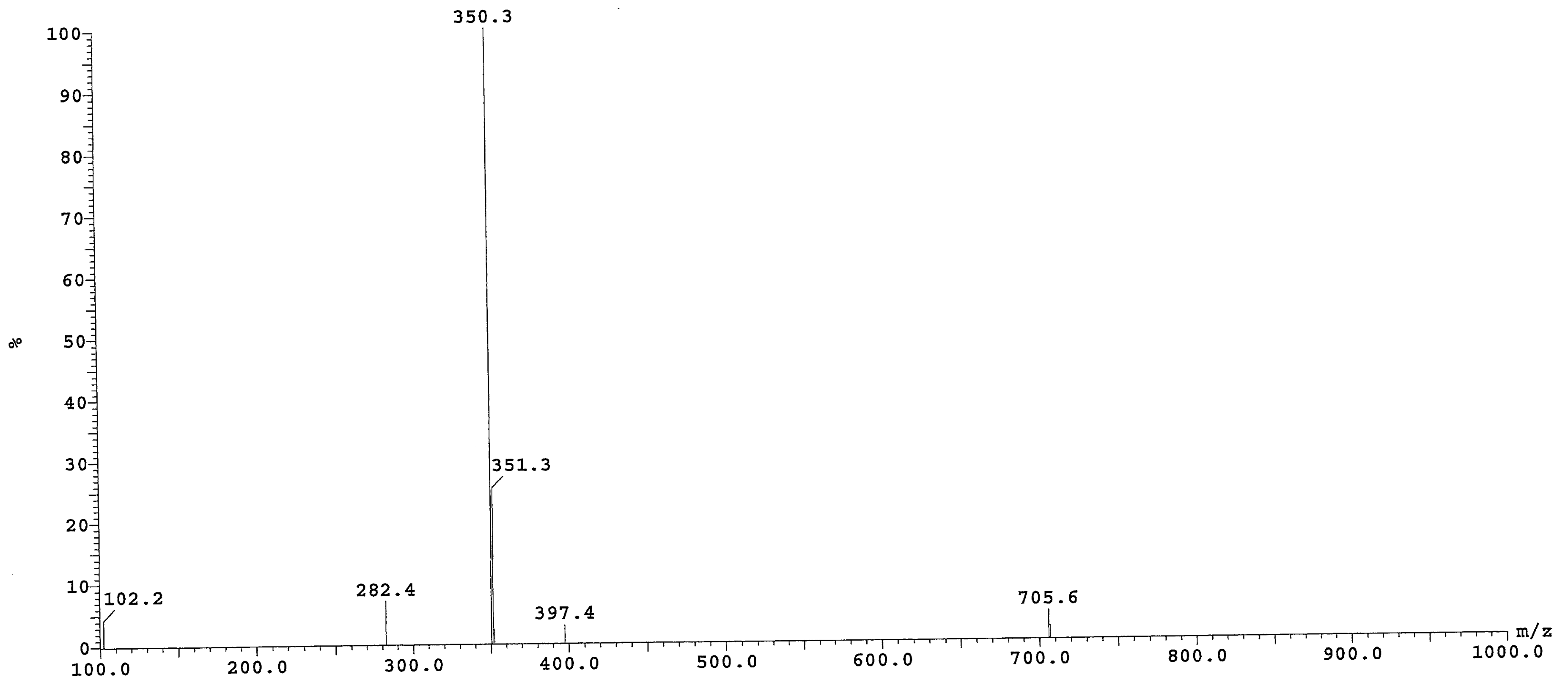

SAMPLE INFORMATION

Sample Name:	ARN-E-9-1
Injection Volume:	3.00 ul
Run Time:	9.0 Minutes
Date Acquired:	6/9/2022 8:32:52 AM EDT
Date Processed:	6/9/2022 9:13:46 AM EDT
Sample Set Name:	Template
Acq. Method Set:	BEH_C18_PDA_75mm 408
Processing Method:	BEH_C18_PDA_CAB
Channel Name:	254nm

Method Notes:
Acquity UPLC BEH C18 1.7u (2.1x75mm)
Flow Rate : 0.5 mL/min
Solvent A : 0.1% TFA in Waters
Solvent B : 0.1% TFA in Acetonitrile
Solvent Gradient Program:

Time (min)	%A	% B
0:00	95	5
6:00	0	100
8:00	0	100
9:00	95	5

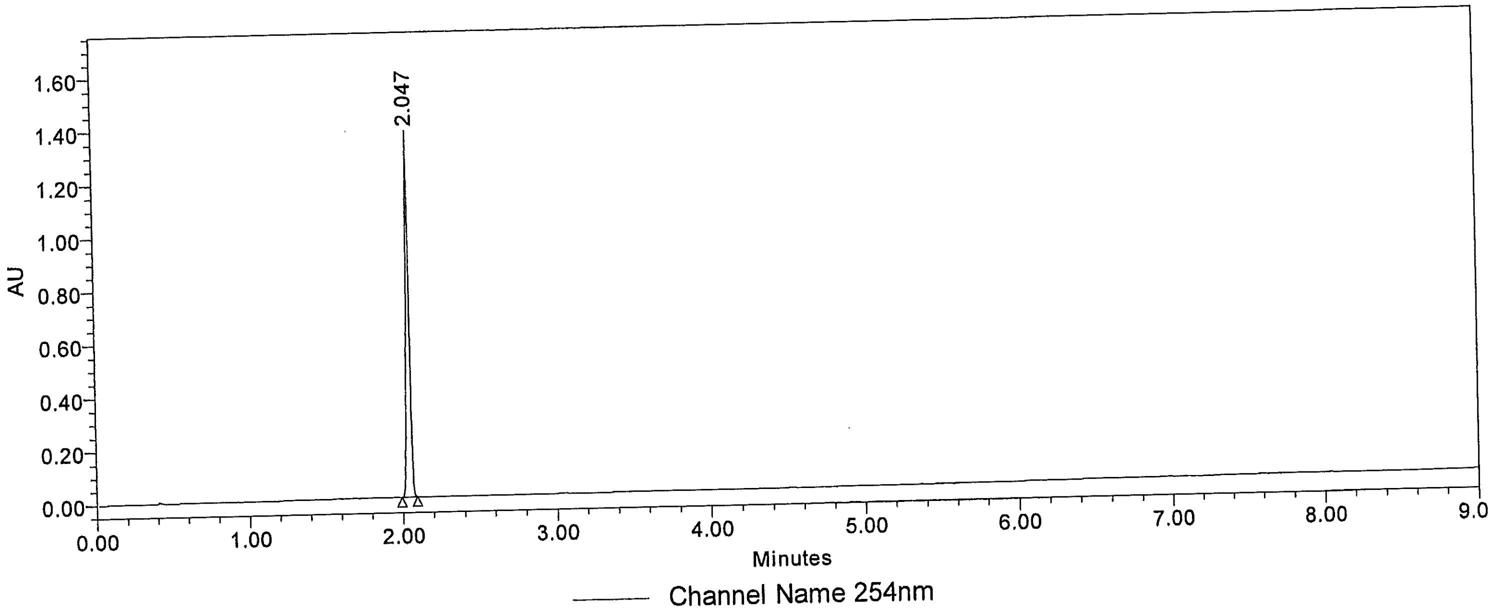

Peak Results

	RT	Area	Int Type	Width (sec)	% Area
1	2.047	2012254	bb	6.150	100.00

Name: Cory Arne H-Bitscher

Date: 6/9/2022

NB #: ARN-E-9-1

CERTIFICATE OF ANALYSIS

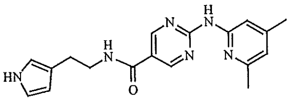

Compound Name: BPN-0035348-AA-001 11
ALB Number: ALB-230045
Batch: 1
Lot Number: ALK-C-9-2
Molecular Formula: $C_{18}H_{20}N_6O$
Molecular Weight: 336.39
Last Solvent: Methylene Chloride, Methanol

TEST	RESULT/REFERENCE
Appearance	Off-white Solid
NMR Spectrum	^{1}H, 300 MHz, Dimethyl Sulfoxide-d_6, Consistent - Attached
Mass Spectrum	ESI, *m/z* 337 $[M + H]^+$, Attached
UPLC	>99% (area %), ACQUITY UPLC BEH C18 (2.1 *75) mm, 1.7 micron Column, UV 254 nm Detection, Attached

[signature]

Approved By

6-15-2022

Date

For Research Purposes Only. Not Intended for Food or Drug Use.

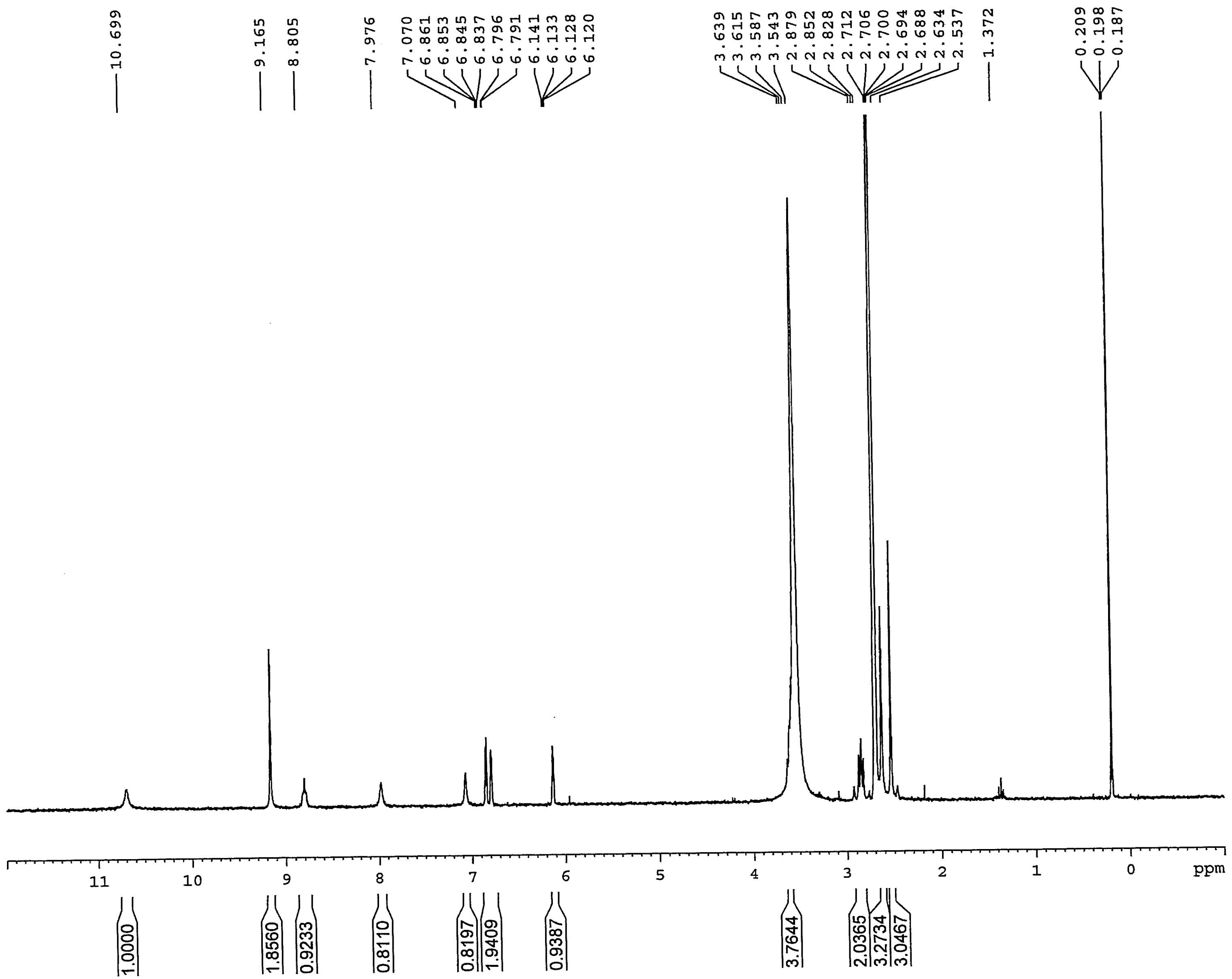

Name Marwah Albaker
Date 13 Jun 2022
NB # ALK-C-9-2

NAME	ALK-C-9-2
EXPNO	20
PROCNO	1
Date_	20220610
Time	11.22
INSTRUM	spect
PROBHD	5 mm QNP 1H/15
PULPROG	zg30
TD	65536
SOLVENT	DMF
NS	32
DS	2
SWH	5995.204 Hz
FIDRES	0.091480 Hz
AQ	5.4657526 sec
RG	1149.4
DW	83.400 usec
DE	6.00 usec
TE	300.0 K
D1	1.00000000 sec
TD0	1

======== CHANNEL f1 ========

NUC1	1H
P1	12.88 usec
PL1	1.00 dB
PL1W	9.77678490 W
SFO1	300.1319509 MHz
SI	32768
SF	300.1300672 MHz
WDW	EM
SSB	0
LB	0.30 Hz
GB	0
PC	1.00

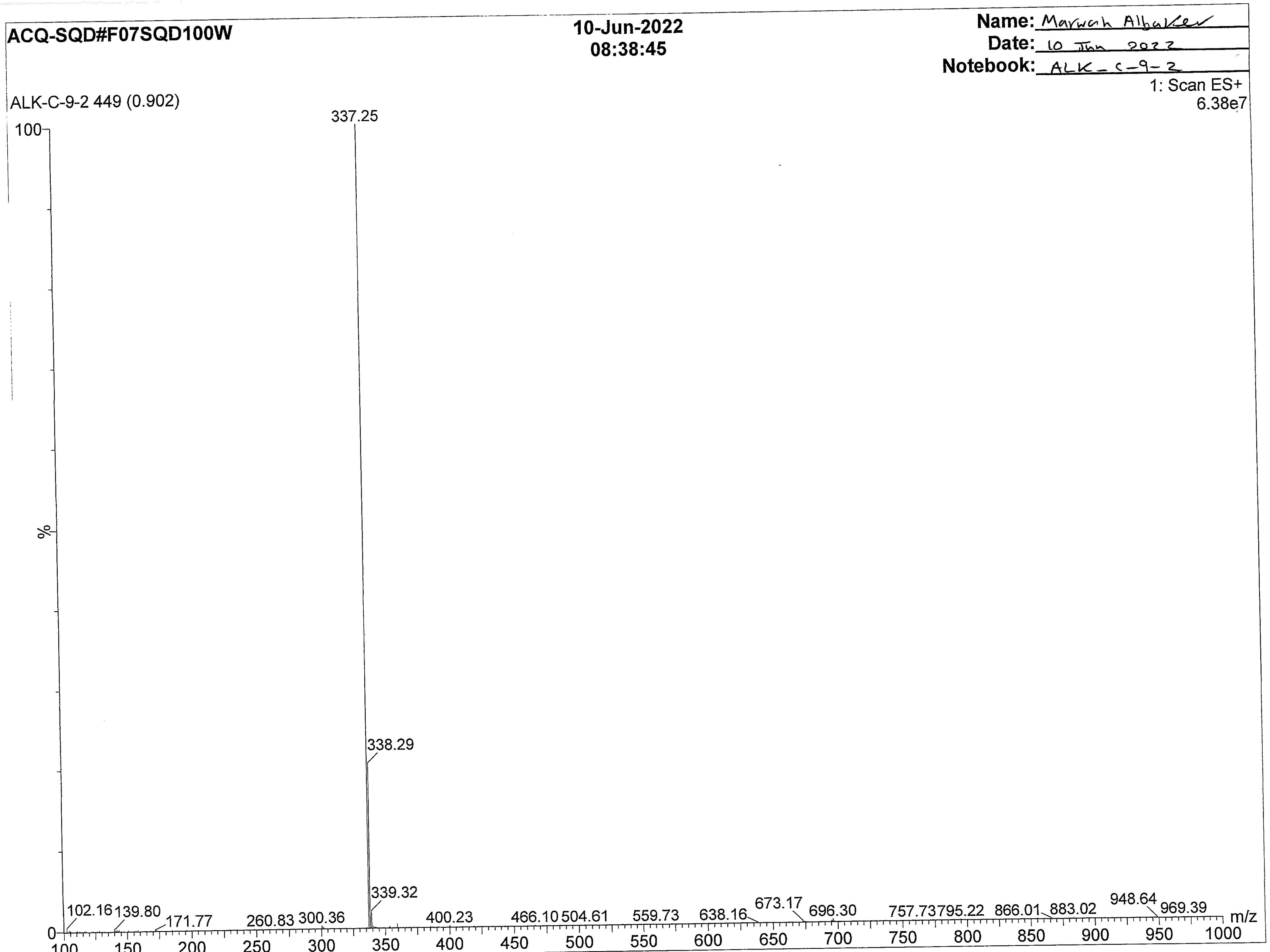

ACQ-SQD#F07SQD100W
10-Jun-2022
08:38:45
Name: Marwah Albaker
Date: 10 Jun 2022
Notebook: ALK_C-9-2
1: Scan ES+
6.38e7
ALK-C-9-2 449 (0.902)
100
%
0
337.25
338.29
339.32
102.16
139.80
171.77
260.83
300.36
400.23
466.10
504.61
559.73
638.16
673.17
696.30
757.73
795.22
866.01
883.02
948.64
969.39
m/z
100
150
200
250
300
350
400
450
500
550
600
650
700
750
800
850
900
950
1000

SAMPLE INFORMATION

Sample Name:	ALK-C-9-2
Injection Volume:	3.00 ul
Run Time:	9.0 Minutes
Date Acquired:	6/10/2022 8:51:44 AM EDT
Date Processed:	6/10/2022 9:03:45 AM EDT
Sample Set Name:	Template
Acq. Method Set:	HSS T3_PDA_75mm_polar 408
Processing Method:	BEH_C18_PDA
Channel Name:	254nm

Method Notes:
Acquity UPLC BEH C18 1.7u (2.1x75mm)
Flow Rate : 0.5 mL/min
Solvent A : 0.1% TFA in Waters
Solvent B : 0.1% TFA in Acetonitrile
Solvent Gradient Program:

Time (min)	%A	% B
0:00	95	5
6:00	0	100
8:00	0	100
9:00	95	5

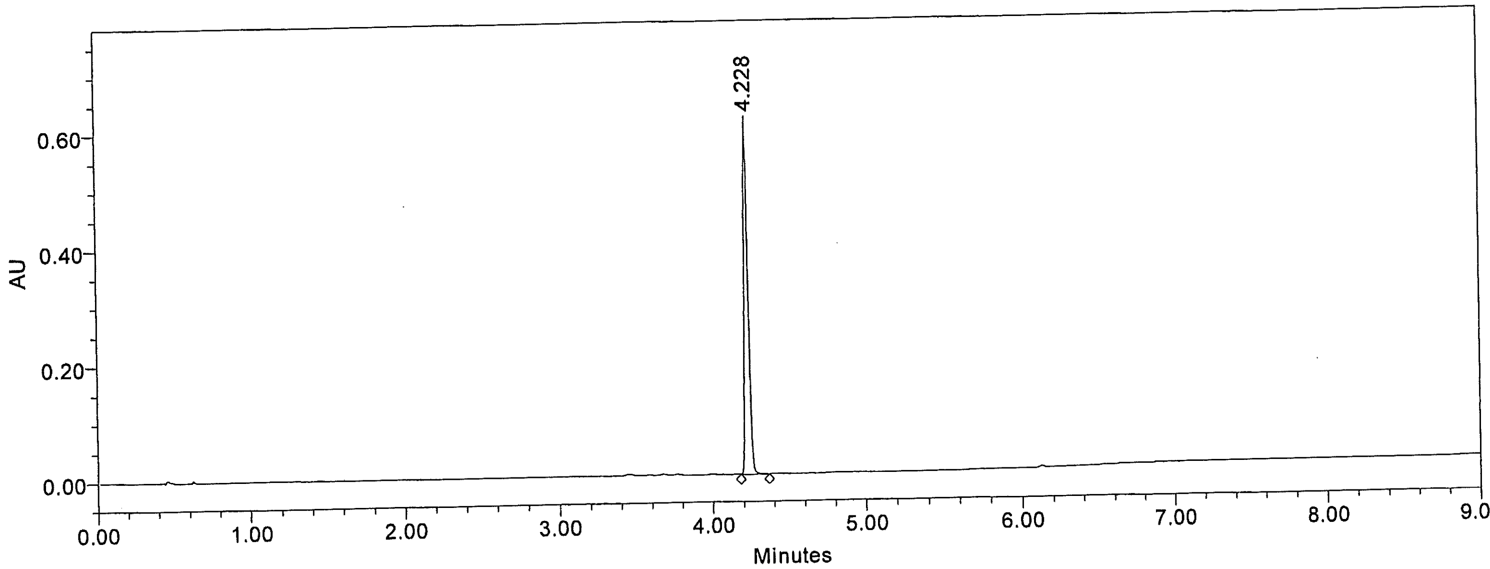

Channel Name 254nm

Peak Results

	RT	Area	Int Type	Width (sec)	% Area
1	4.228	1142640	VV	11.052	100.00

Name: Marwah Albaker

Date: 10 Jun 2022

NB #: ALK-C-9-2

CERTIFICATE OF ANALYSIS

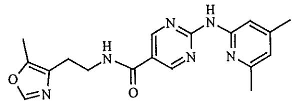

Compound Name: BPN-0035349-AA-001 1m
ALB Number: ALB-230044
Batch: 1
Lot Number: ALK-C-10-1
Molecular Formula: $C_{18}H_{20}N_6O_2$
Molecular Weight: 352.39
Last Solvent: Ethyl Acetate

TEST	RESULT/REFERENCE
Appearance	Off-white Solid
NMR Spectrum	^{1}H, 300 MHz, Dimethyl Sulfoxide-d_6, Consistent - Attached
Mass Spectrum	ESI, *m/z* 353 $[M + H]^+$, Attached
UPLC	>99% (area %), ACQUITY UPLC BEH C18 (2.1 *75) mm, 1.7 micron Column, UV 254 nm Detection, Attached

[signature]
Approved By

6-15-2022
Date

For Research Purposes Only. Not Intended for Food or Drug Use.

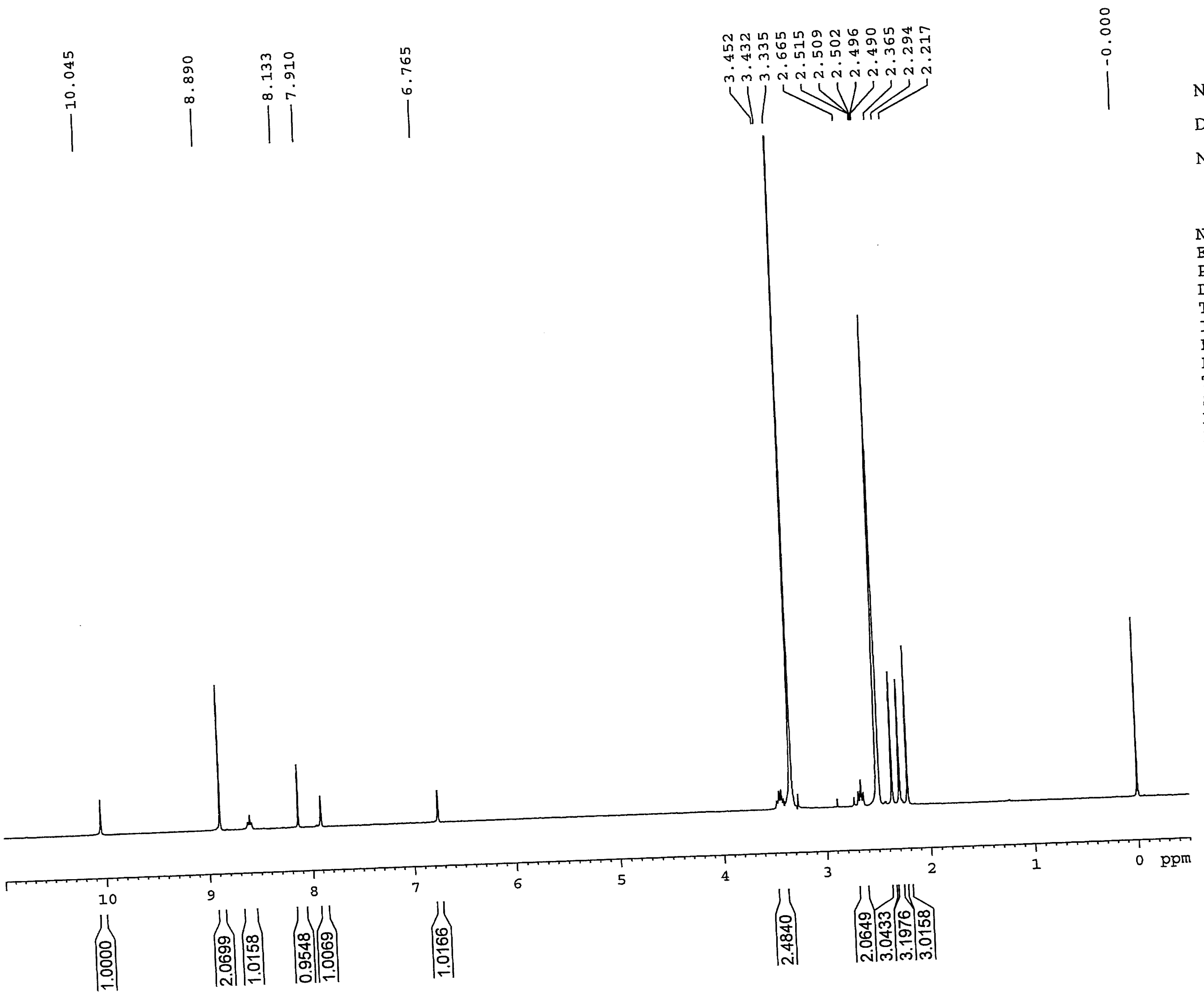

Name Marwah Albaker
Date 10 Jun 2022
NB # ALK_C-10-1

NAME	ALK-C-10-1
EXPNO	10
PROCNO	1
Date_	20220610
Time	10.55
INSTRUM	spect
PROBHD	5 mm QNP 1H/15
PULPROG	zg30
TD	65536
SOLVENT	DMSO
NS	32
DS	2
SWH	5995.204 Hz
FIDRES	0.091480 Hz
AQ	5.4657526 sec
RG	812.7
DW	83.400 usec
DE	6.00 usec
TE	300.0 K
D1	1.00000000 sec
TD0	1

======== CHANNEL f1 ========

NUC1	1H
P1	12.88 usec
PL1	1.00 dB
PL1W	9.77678490 W
SFO1	300.1319509 MHz
SI	32768
SF	300.1300004 MHz
WDW	EM
SSB	0
LB	0.30 Hz
GB	0
PC	1.00

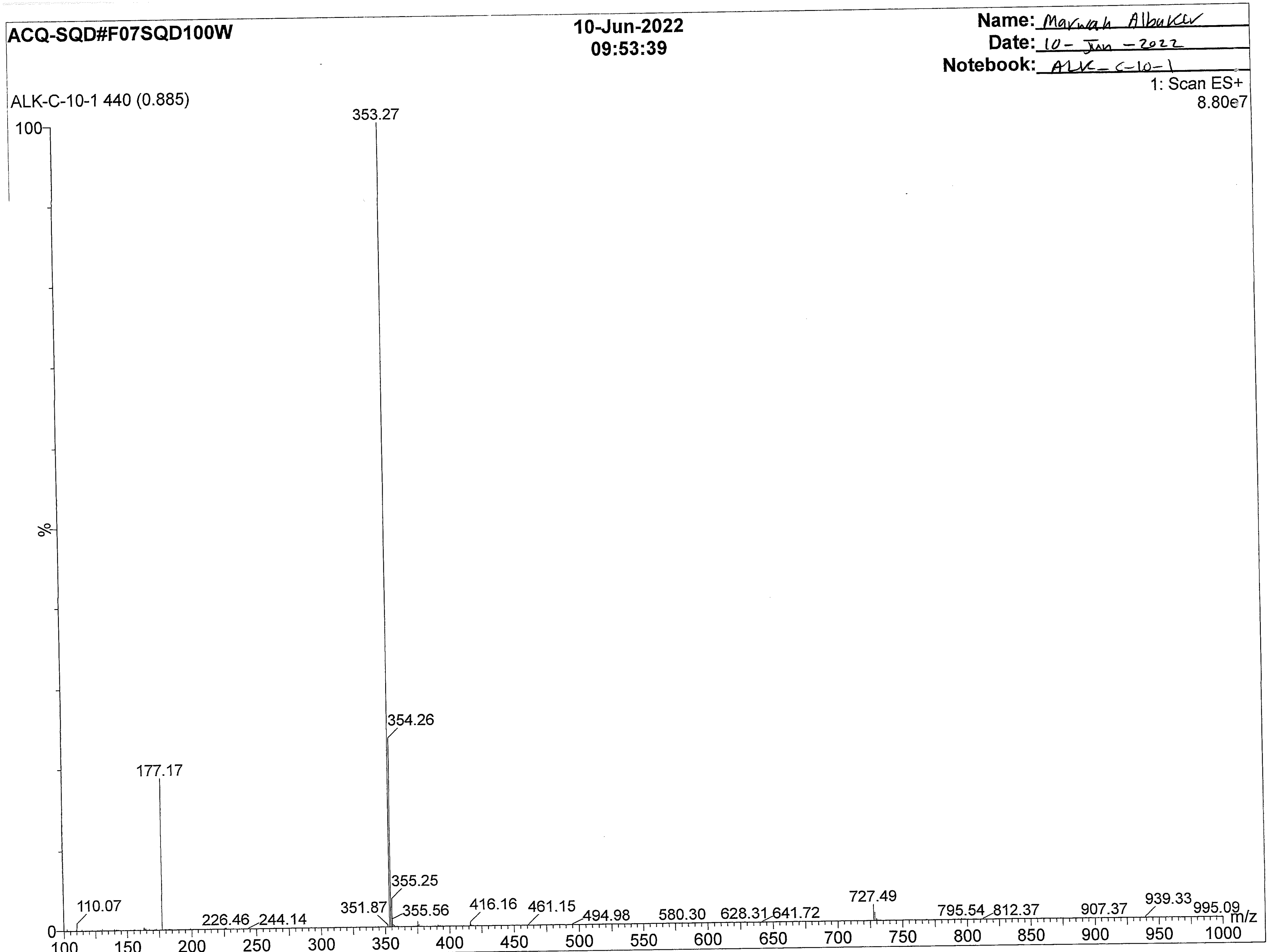
ACQ-SQD#F07SQD100W
10-Jun-2022
09:53:39
Name: Marwah Albaker
Date: 10-Jun-2022
Notebook: ALK-C-10-1
1: Scan ES+
8.80e7
ALK-C-10-1 440 (0.885)
100
%
0
353.27
354.26
177.17
355.25
110.07
226.46
244.14
351.87
355.56
416.16
461.15
494.98
580.30
628.31
641.72
727.49
795.54
812.37
907.37
939.33
995.09
m/z
100
150
200
250
300
350
400
450
500
550
600
650
700
750
800
850
900
950
1000

SAMPLE INFORMATION

Sample Name:	ALK-C-10-1
Injection Volume:	3.00 ul
Run Time:	9.0 Minutes
Date Acquired:	6/10/2022 10:02:43 AM EDT
Date Processed:	6/10/2022 10:14:45 AM EDT
Sample Set Name:	Template
Acq. Method Set:	HSS T3_PDA_75mm_polar 408
Processing Method:	BEH_C18_PDA
Channel Name:	254nm

Method Notes:
Acquity UPLC BEH C18 1.7u (2.1x75mm)
Flow Rate : 0.5 mL/min
Solvent A : 0.1% TFA in Waters
Solvent B : 0.1% TFA in Acetonitrile
Solvent Gradient Program:

Time (min)	%A	% B
0:00	95	5
6:00	0	100
8:00	0	100
9:00	95	5

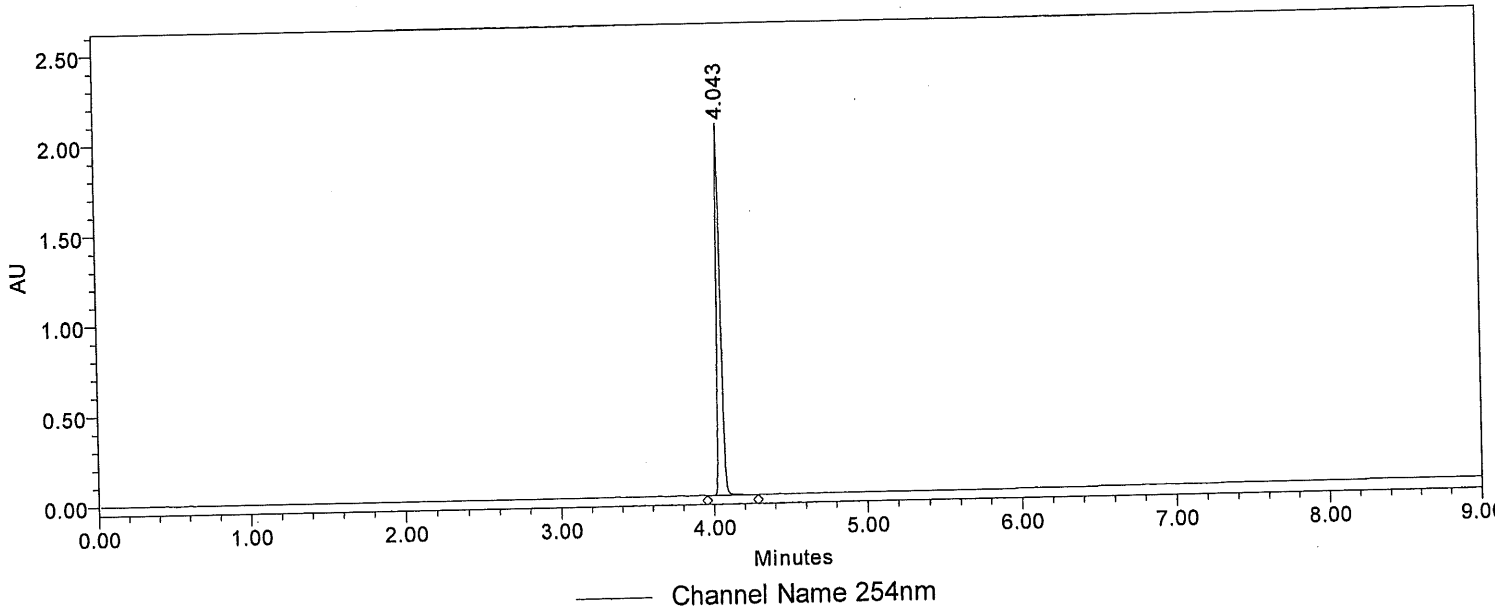

Peak Results

	RT	Area	Int Type	Width (sec)	% Area
1	4.043	3675992	VV	19.954	100.00

Name: Marwah Albuker

Date: 10-Jun 2022

NB #: ALK-C-10-1

CERTIFICATE OF ANALYSIS

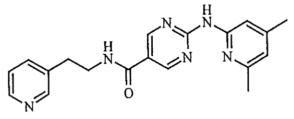

Compound Name: BPN-0035350-AA-001 1n
ALB Number: ALB-230049
Batch: 1
Lot Number: ALK-C-12-1
Molecular Formula: $C_{19}H_{20}N_6O$
Molecular Weight: 348.40
Last Solvent: Ethyl Acetate

TEST	RESULT/REFERENCE
Appearance	Off-white Solid
NMR Spectrum	^{1}H, 300 MHz, Dimethyl Sulfoxide-d_6, Consistent - Attached
Mass Spectrum	ESI, *m/z* 349 $[M + H]^+$, Attached
UPLC	97.9% (area %), ACQUITY UPLC BEH C18 (2.1 *75) mm, 1.7 micron Column, UV 254 nm Detection, Attached

Approved By

Date 6-15-2022

For Research Purposes Only. Not Intended for Food or Drug Use.

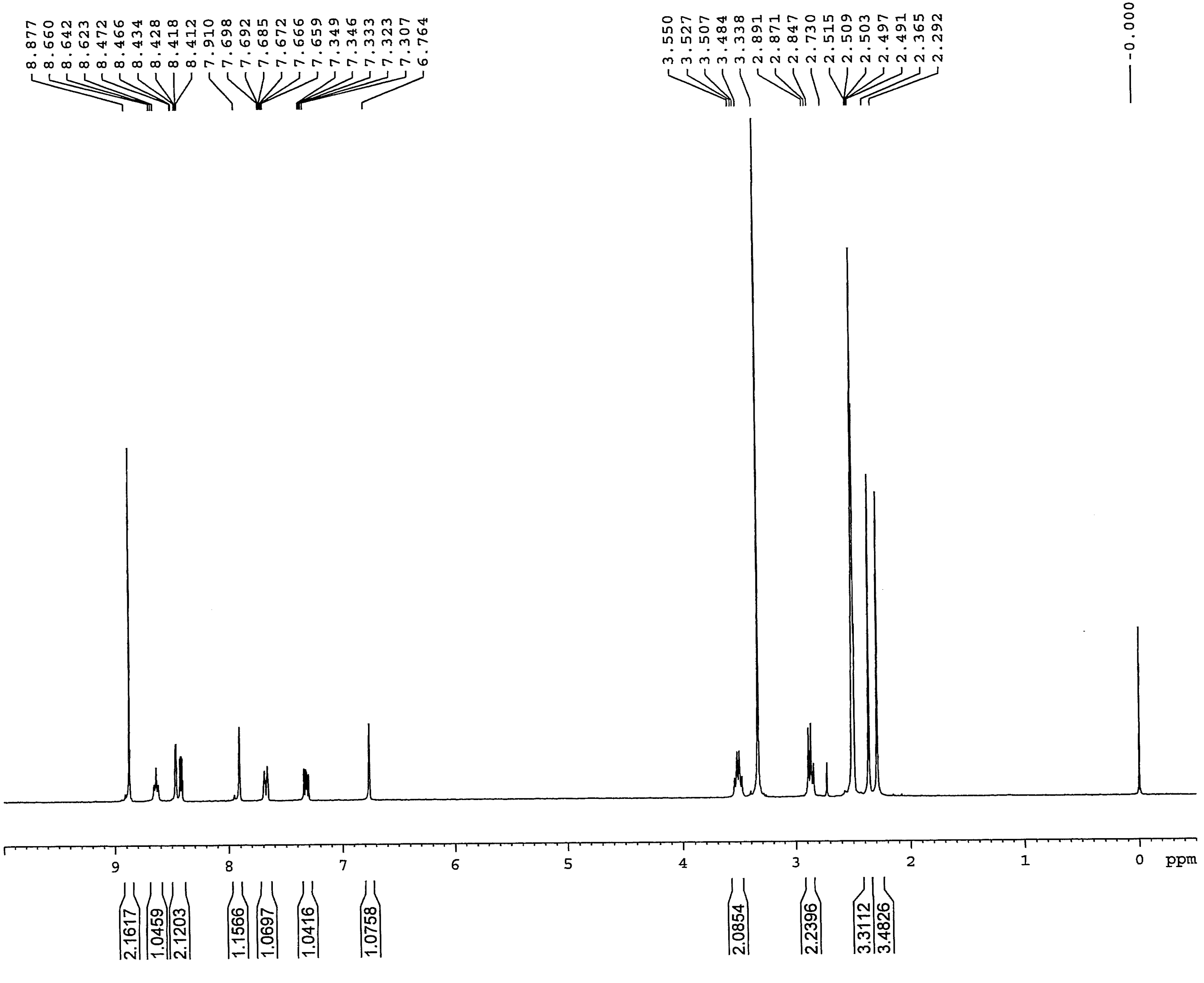

Name Marwah Albaker

Date 13 Jun 2022

NB # ALK_C_12_1

NAME	ALK-C-12-1
EXPNO	10
PROCNO	1
Date_	20220610
Time	13.51
INSTRUM	spect
PROBHD	5 mm QNP 1H/15
PULPROG	zg30
TD	65536
SOLVENT	DMSO
NS	32
DS	2
SWH	5995.204 Hz
FIDRES	0.091480 Hz
AQ	5.4657526 sec
RG	812.7
DW	83.400 usec
DE	6.00 usec
TE	300.0 K
D1	1.00000000 sec
TD0	1

======== CHANNEL f1 ========

NUC1	1H
P1	12.88 usec
PL1	1.00 dB
PL1W	9.77678490 W
SFO1	300.1319509 MHz
SI	32768
SF	300.1300001 MHz
WDW	EM
SSB	0
LB	0.30 Hz
GB	0
PC	1.00

ACQ-SQD#F07SQD100W **10-Jun-2022 11:22:15**

Name: Marwah Alhaker
Date: 10 Jun 2022
Notebook: ALK-C-12-1

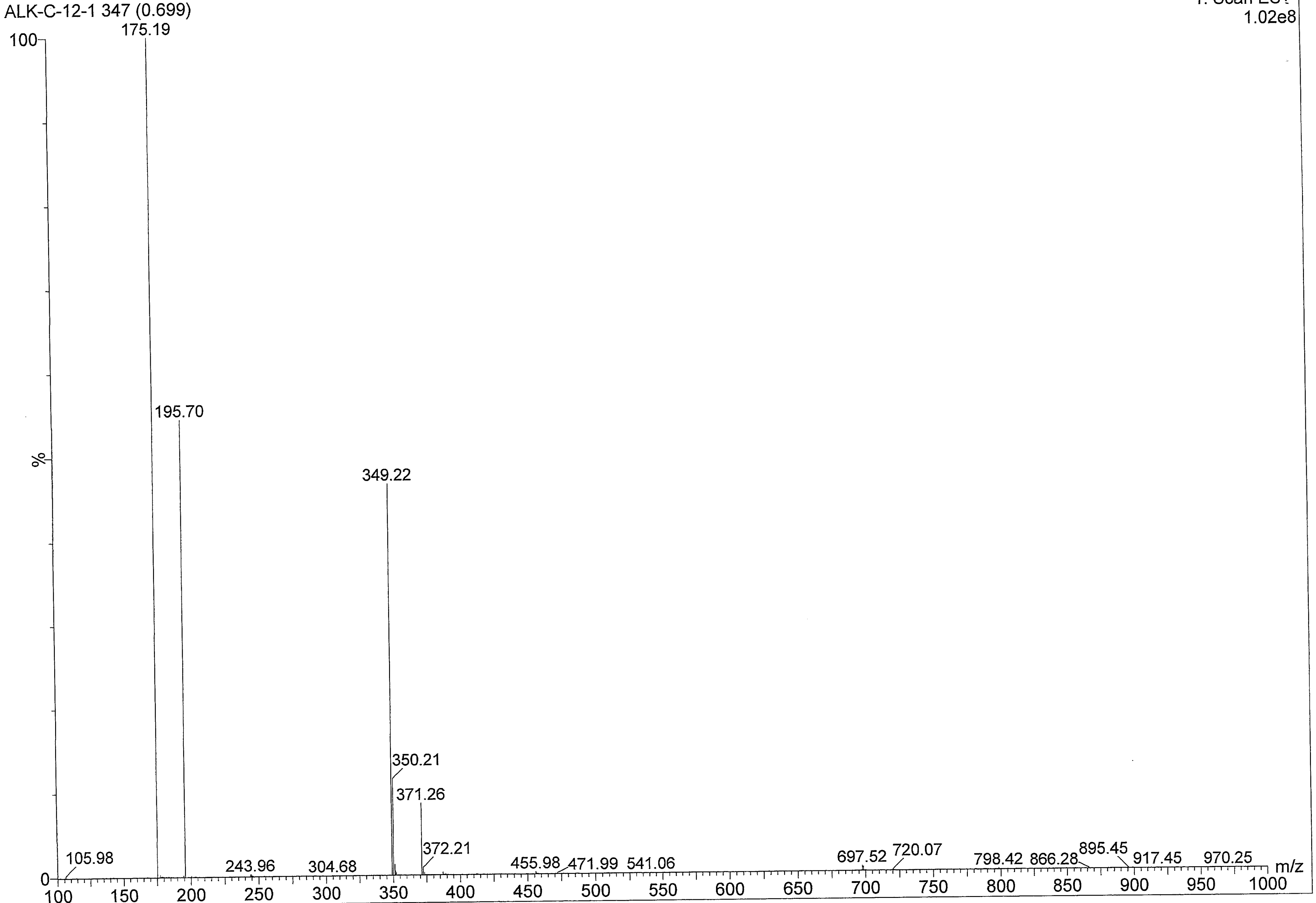

SAMPLE INFORMATION

Sample Name:	ALK-C12-1
Injection Volume:	3.00 ul
Run Time:	9.0 Minutes
Date Acquired:	6/10/2022 12:06:08 PM EDT
Date Processed:	6/10/2022 1:22:54 PM EDT
Sample Set Name:	Template
Acq. Method Set:	HSS T3_PDA_75mm_polar 408
Processing Method:	BEH_C18_PDA
Channel Name:	254nm

Method Notes:
Acquity UPLC HSS T3 1.8u (2.1x75mm)
Flow Rate : 0.5 mL/min
Solvent A : 0.1% TFA in Waters
Solvent B : 0.1% TFA in Acetonitrile
Solvent Gradient Program:

Time (min)	%A	% B
0:00	95	5
1:00	95	5
3:30	70	30
6:00	0	100
8:00	0	100
9:00	95	5

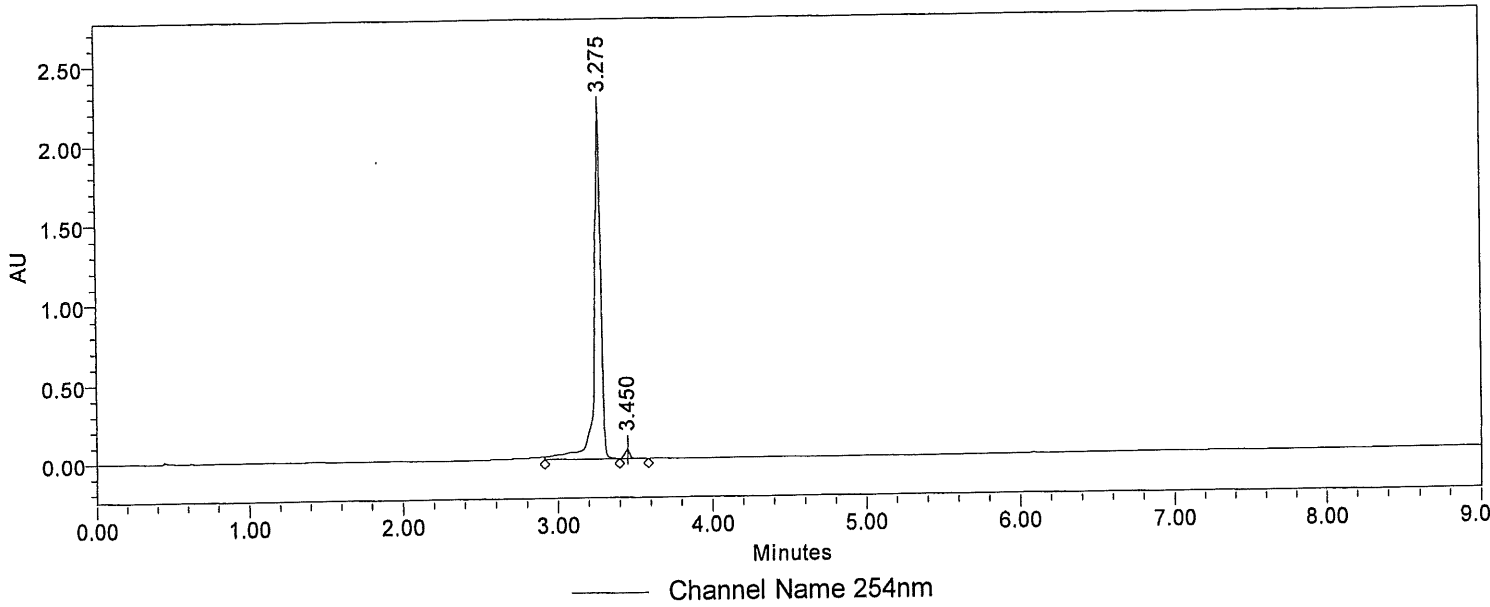

Peak Results

	RT	Area	Int Type	Width (sec)	% Area
1	3.275	6422392	VV	29.048	97.99
2	3.450	131409	VV	11.199	2.01

Name: Marwah Albaker

Date: 10 Jun 2022

NB #: ALK-C-12-1

Printed 1:23:20 PM US/Eastern 6/10/2022 Page: 1 of 1

CERTIFICATE OF ANALYSIS

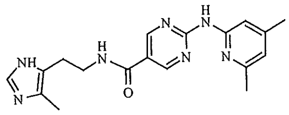

Compound Name: BPN-0035917-AA-001 10
ALB Number: ALB-230101
Batch: 1
Lot Number: ALK-C-11-4
Molecular Formula: $C_{18}H_{21}N_7O$
Molecular Weight: 351.41
Last Solvent: Methylene Chloride, Methanol

TEST	RESULT/REFERENCE
Appearance	Off-white Solid
NMR Spectrum	^{1}H, 500 MHz, Dimethyl Sulfoxide-d_6, Consistent - Attached
Mass Spectrum	ESI, *m/z* 352 $[M + H]^+$, Attached
UPLC	95.0% (area %), ACQUITY UPLC BEH C18 (2.1 *75) mm, 1.7 micron Column, UV 254 nm Detection, Attached

[signature]
Approved By

6-22-2022
Date

For Research Purposes Only. Not Intended for Food or Drug Use.

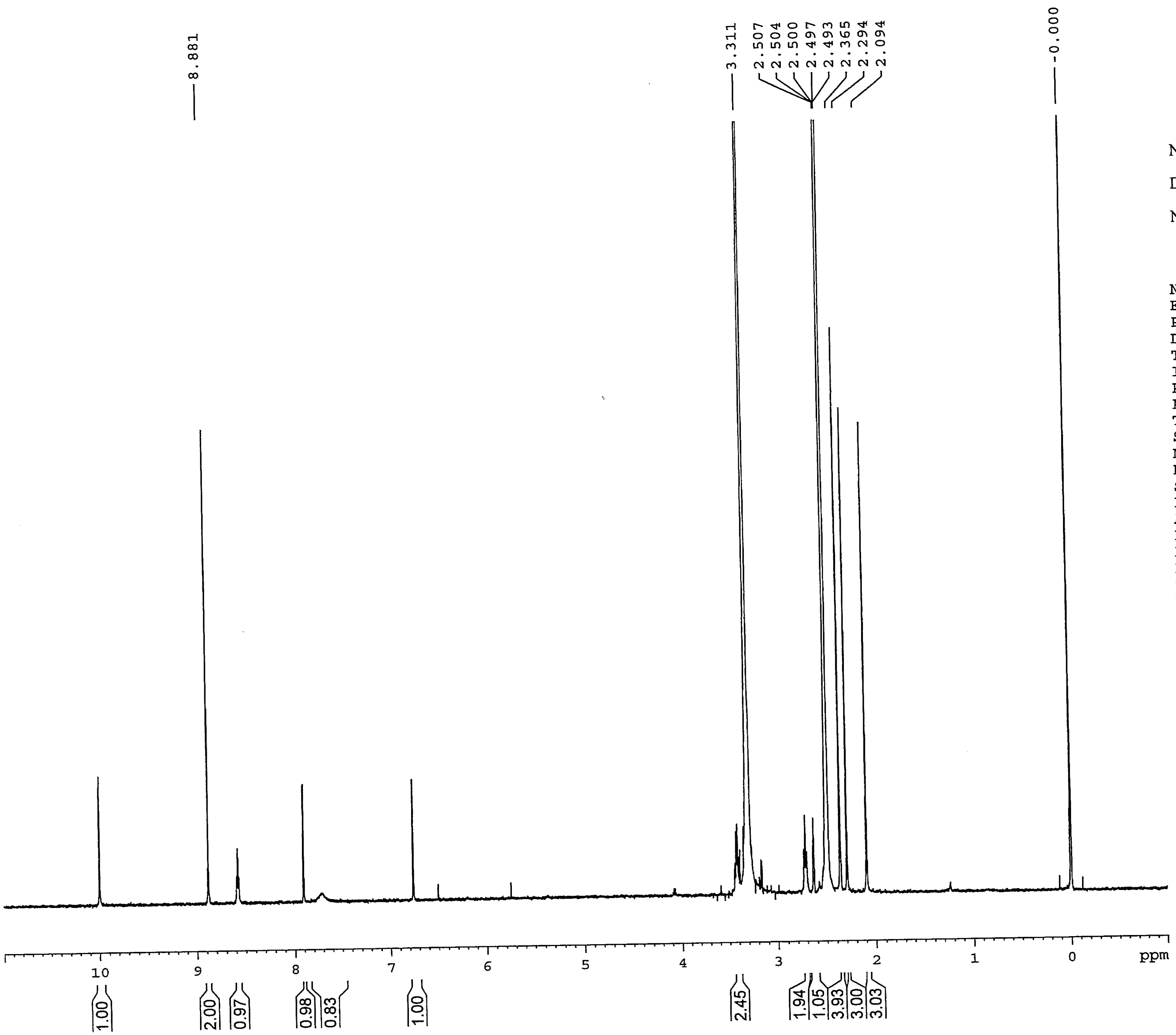

Name Marwah Albaker

Date 20 Jun 2022

NB # ALK-C-11-4

NAME	ALK-C-11-4
EXPNO	10
PROCNO	1
Date_	20220615
Time_	15.38 h
INSTRUM	Avance Neo
PROBHD	Z167419_0029 (
PULPROG	zg30
TD	65536
SOLVENT	DMSO
NS	64
DS	2
SWH	10000.000 Hz
FIDRES	0.305176 Hz
AQ	3.2768500 sec
RG	101
DW	50.000 usec
DE	11.14 usec
TE	300.0 K
D1	1.00000000 sec
TD0	1
SFO1	500.1330883 MHz
NUC1	1H
P0	2.67 usec
P1	8.00 usec
SI	65536
SF	500.1300042 MHz
WDW	EM
SSB	0
LB	0.30 Hz
GB	0
PC	1.00

ACQ-SQD#F07SQD100W

15-Jun-2022
14:34:47

Name: Marwan Albuker
Date: 15 Jun 2022
Notebook: ALK-C-11-4

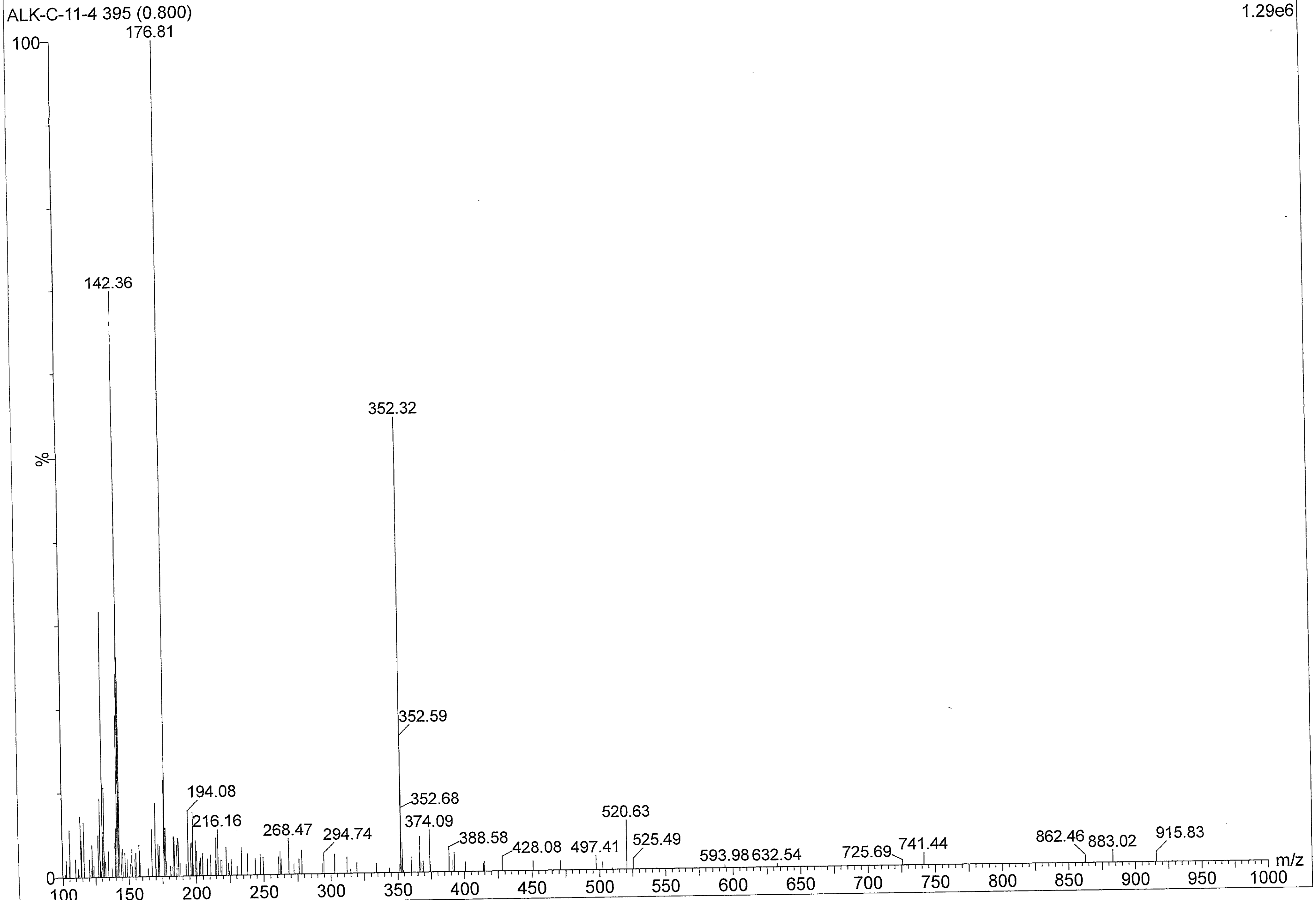

SAMPLE INFORMATION

Sample Name: ALK-C-11-4
Injection Volume: 3.00 ul
Run Time: 9.0 Minutes
Date Acquired: 6/15/2022 3:03:40 PM EDT
Date Processed: 6/15/2022 3:33:10 PM EDT
Sample Set Name: Template
Acq. Method Set: HSS T3_PDA_75mm_polar 408
Processing Method: BEH_C18_PDA
Channel Name: 254nm

Method Notes:
Acquity UPLC BEH C18 1.7u (2.1x75mm)
Flow Rate : 0.5 mL/min
Solvent A : 0.1% TFA in Waters
Solvent B : 0.1% TFA in Acetonitrile
Solvent Gradient Program:

Time (min)	%A	% B
0:00	95	5
6:00	0	100
8:00	0	100
9:00	95	5

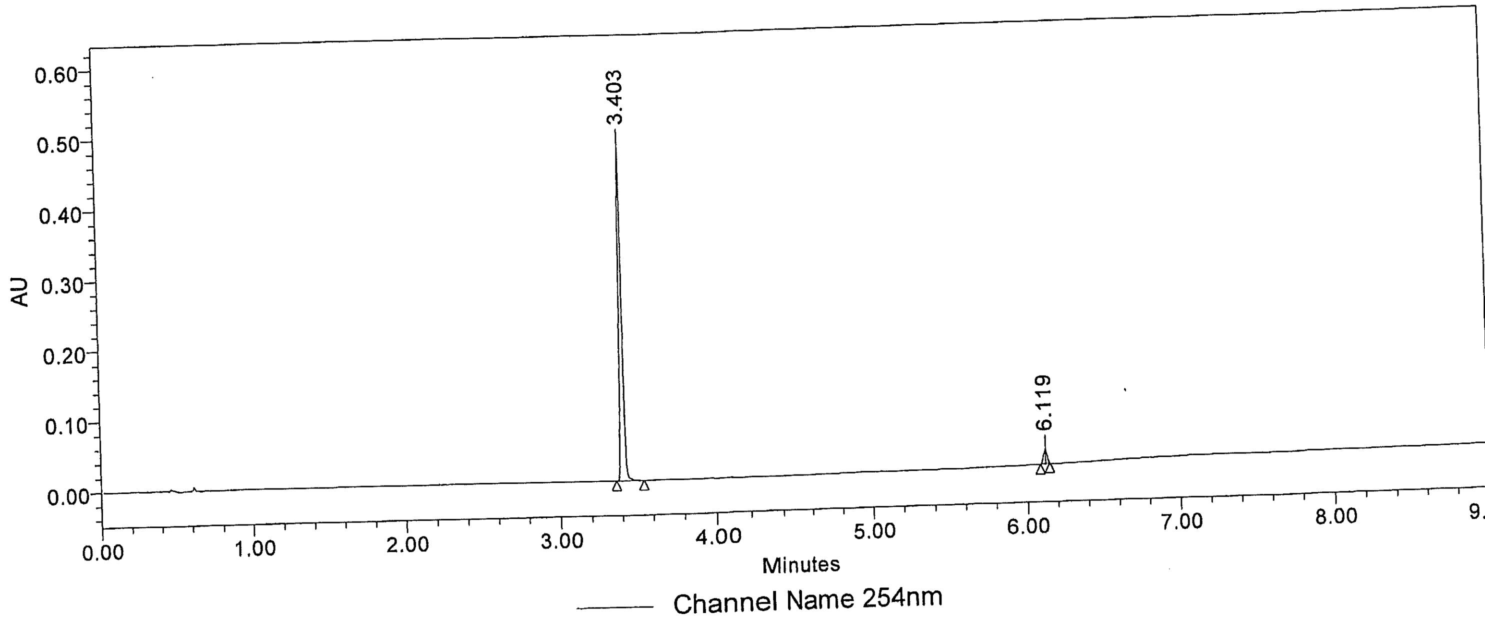

Peak Results

	RT	Area	Int Type	Width (sec)	% Area
1	3.403	623324	BB	10.649	95.08
2	6.119	32247	bb	3.601	4.92

Name: Marwah Albaker

Date: 15 Jun 2022

NB #: ALK - C - 11 - 4

CERTIFICATE OF ANALYSIS

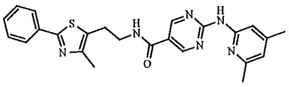

Compound Name: BPN-0036211-AA-001 1p
ALB Number: ALB-231431
Batch: 1
Lot Number: ARN-E-90-4
Molecular Formula: $C_{24}H_{24}N_6OS$
Molecular Weight: 444.55
Last Solvent: Methanol

TEST	RESULT/REFERENCE
Appearance	Off-white Solid
NMR Spectrum	^{1}H, 500 MHz, Dimethyl Sulfoxide-d_6, Consistent - Attached
Mass Spectrum	ESI, *m/z* 445 [M + H]$^+$, Attached
UPLC	97.3% (area %), ACQUITY UPLC BEH C18 (2.1 *75) mm, 1.7 micron Column, UV 254 nm Detection, Attached

Approved By

9-28-2022
Date

For Research Purposes Only. Not Intended for Food or Drug Use.

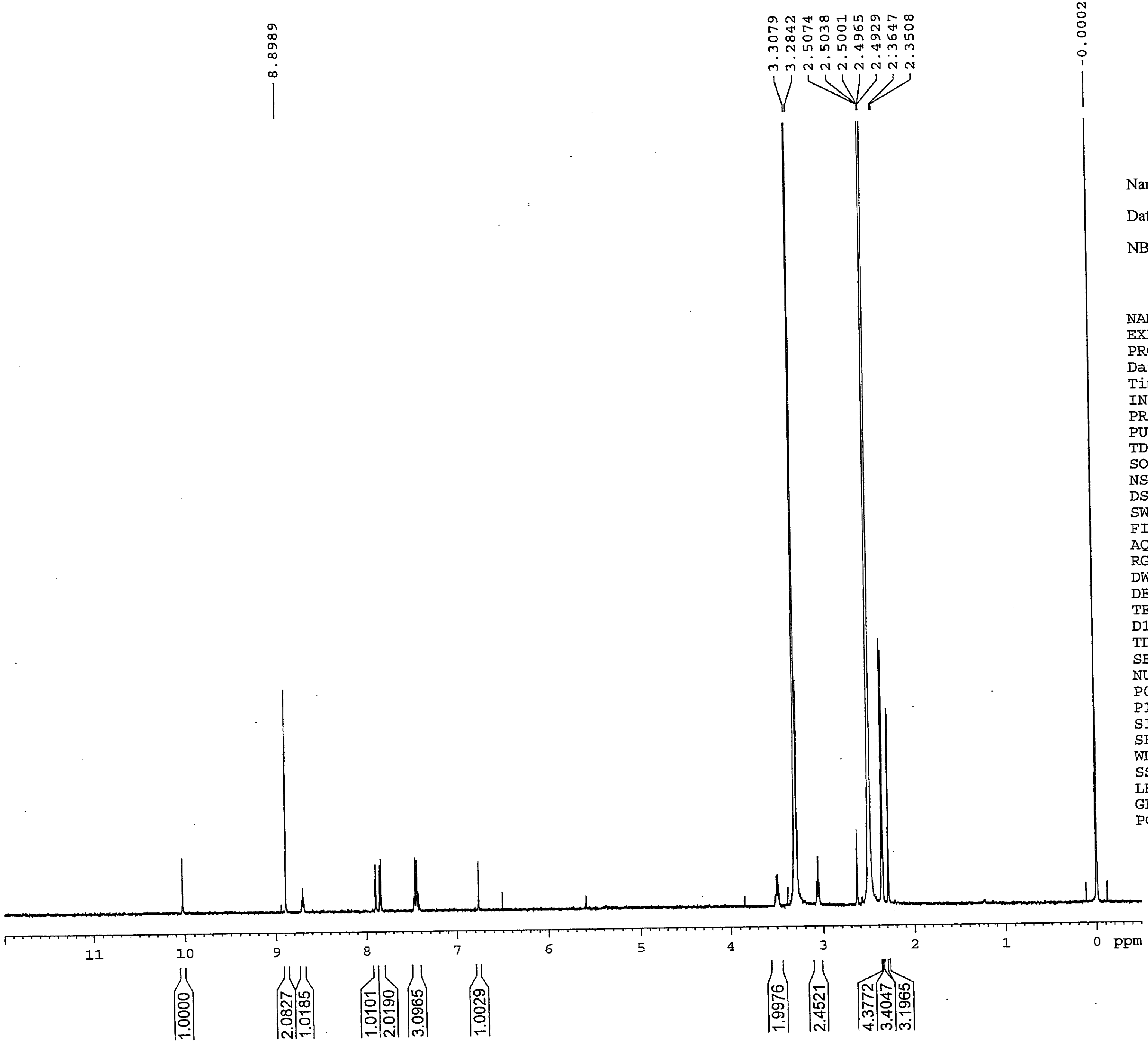

Name Cory Arnett-Betscher

Date 9/26/2022

NB # ARN-E-90-4

NAME	ARN-E-90-4
EXPNO	10
PROCNO	1
Date_	20220926
Time	10.14 h
INSTRUM	Avance Neo
PROBHD	Z167419_0029 (
PULPROG	zg30
TD	65536
SOLVENT	DMSO
NS	32
DS	2
SWH	10000.000 Hz
FIDRES	0.305176 Hz
AQ	3.2768500 sec
RG	101
DW	50.000 usec
DE	11.14 usec
TE	300.0 K
D1	1.00000000 sec
TD0	1
SFO1	500.1330883 MHz
NUC1	1H
P0	2.67 usec
P1	8.00 usec
SI	65536
SF	500.1300041 MHz
WDW	EM
SSB	0
LB	0.30 Hz
GB	0
PC	1.00

Openlynx Report
Vial:1:8
Date:23-Sep-2022
Name: Corey Arnett-Setscher

ID:
Time:08:28:35
Date: 9/26/2022

File:ARN-E-90-4
Notebook: ARN-E-90-4

Page 1

Printed: Fri Sep 23 08:30:54 2022

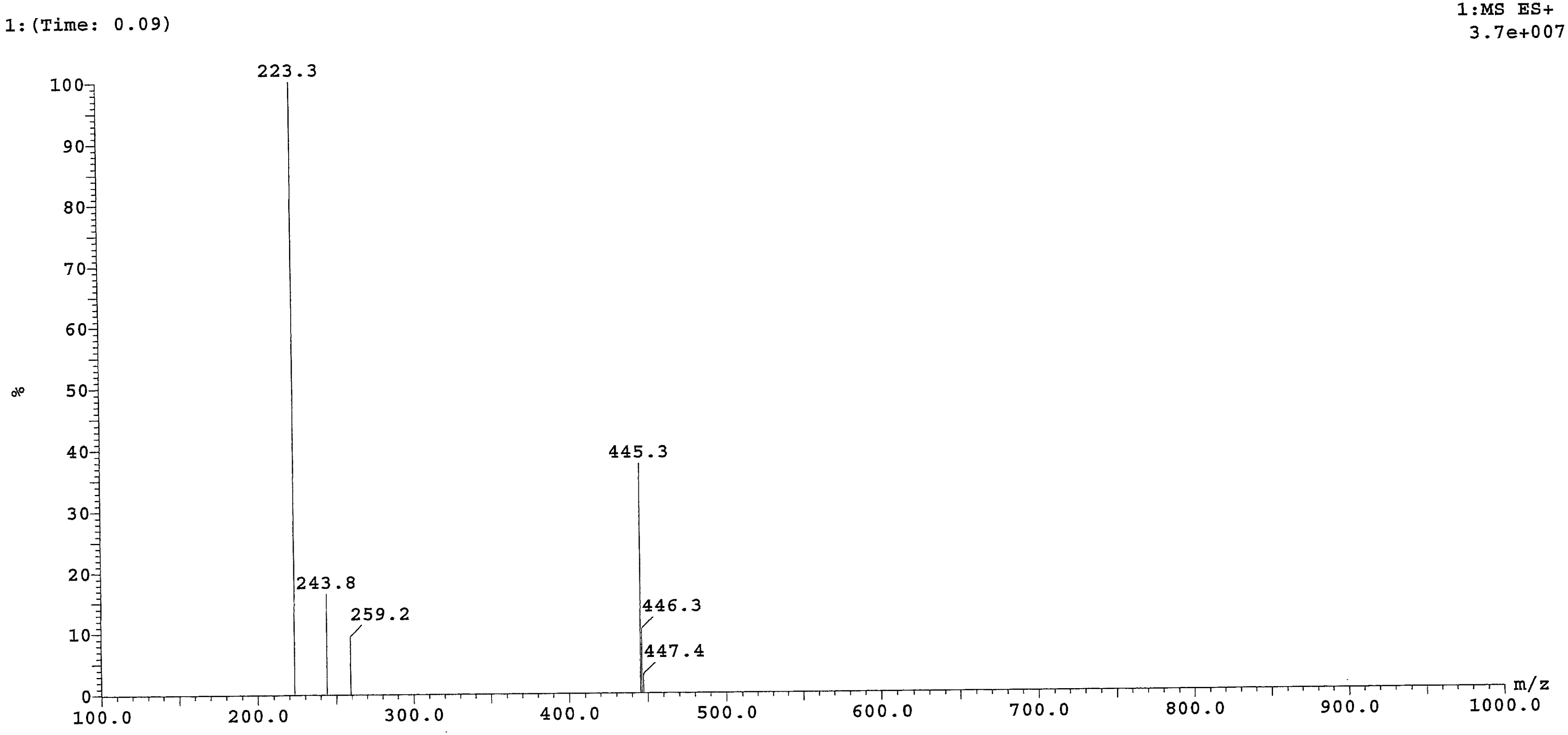

SAMPLE INFORMATION

Sample Name:	ARN-E-90-4
Injection Volume:	3.00 ul
Run Time:	9.0 Minutes
Date Acquired:	9/23/2022 7:52:58 AM EDT
Date Processed:	9/23/2022 8:26:08 AM EDT
Sample Set Name:	Template
Acq. Method Set:	BEH_C18_PDA_75mm 408
Processing Method:	BEH_C18_PDA_CAB
Channel Name:	254nm

Method Notes:
Acquity UPLC BEH C18 1.7u (2.1x75mm)
Flow Rate : 0.5 mL/min
Solvent A : 0.1% TFA in Waters
Solvent B : 0.1% TFA in Acetonitrile
Solvent Gradient Program:

Time (min)	%A	% B
0:00	95	5
6:00	0	100
8:00	0	100
9:00	95	5

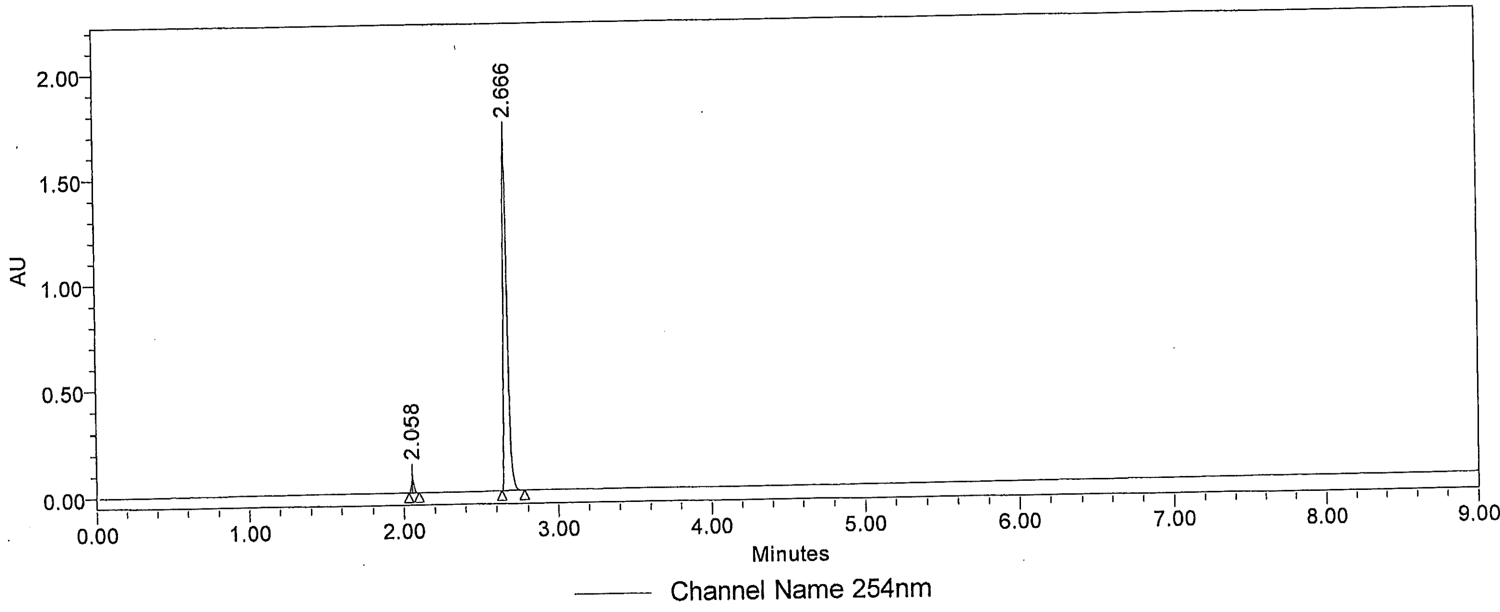

Peak Results

	RT	Area	Int Type	Width (sec)	% Area
1	2.058	80869	bb	4.000	2.65
2	2.666	2966180	bb	8.699	97.35

Name: Cory Arnett-Butscher

Date: 9/26/2022

NB #: ARN-E-90-4

CERTIFICATE OF ANALYSIS

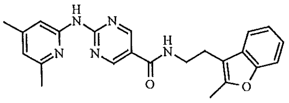

Compound Name: BPN-0035346-AA-001 1q
ALB Number: ALB-230039
Batch: 1
Lot Number: ARN-E-7-1
Molecular Formula: $C_{23}H_{23}N_5O_2$
Molecular Weight: 401.46
Last Solvent: Water, Ethyl Acetate

TEST	RESULT/REFERENCE
Appearance	White Solid
NMR Spectrum	^{1}H, 500 MHz, Dimethyl Sulfoxide-d_6, Consistent - Attached
Mass Spectrum	ESI, *m/z* 402 [M + H]$^+$, Attached
UPLC	97.5% (area %), ACQUITY UPLC BEH C18 (2.1 *75) mm, 1.7 micron Column, UV 254 nm Detection, Attached

Approved By

6-15-2022
Date

For Research Purposes Only. Not Intended for Food or Drug Use.

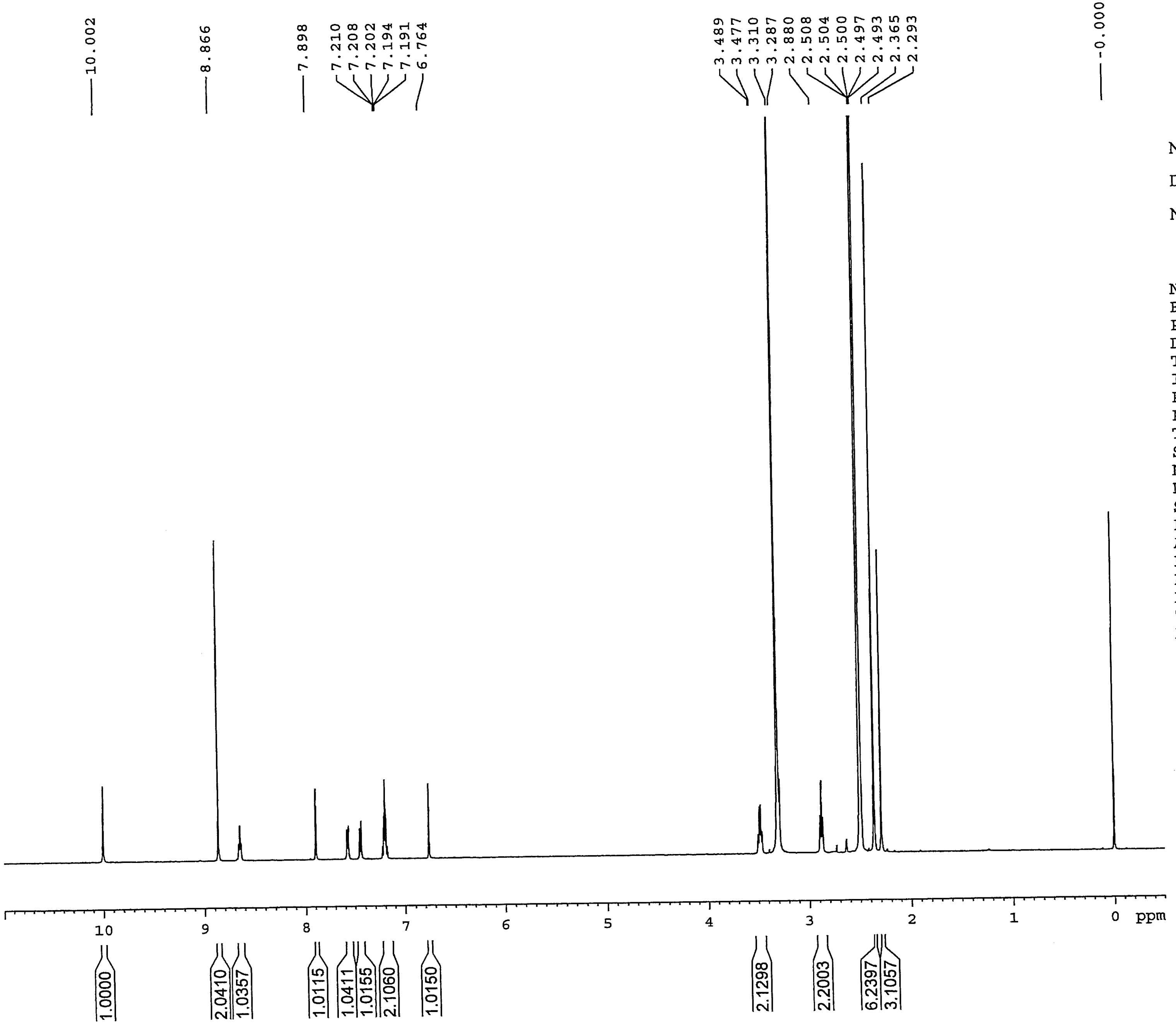

Name Corey Arnett-Butscher

Date 6/8/2022

NB # ARN-E-7-1

NAME	ARN-E-7-1
EXPNO	10
PROCNO	1
Date_	20220608
Time	9.20 h
INSTRUM	Avance Neo
PROBHD	Z167419_0029 (
PULPROG	zg30
TD	65536
SOLVENT	DMSO
NS	32
DS	2
SWH	10000.000 Hz
FIDRES	0.305176 Hz
AQ	3.2768500 sec
RG	101
DW	50.000 usec
DE	11.14 usec
TE	300.0 K
D1	1.00000000 sec
TD0	1
SFO1	500.1330883 MHz
NUC1	1H
P0	2.67 usec
P1	8.00 usec
SI	65536
SF	500.1300041 MHz
WDW	EM
SSB	0
LB	0.30 Hz
GB	0
PC	1.00

Openlynx Report
Vial:1:4
Date:08-Jun-2022
Name: Corey Arnett-Butscher

ID:
Time:10:49:36
Date: 6/8/2022

Page 1
File:ARN-E-7-1
Notebook: ARN-E-7-1

Printed: Wed Jun 08 10:51:32 2022

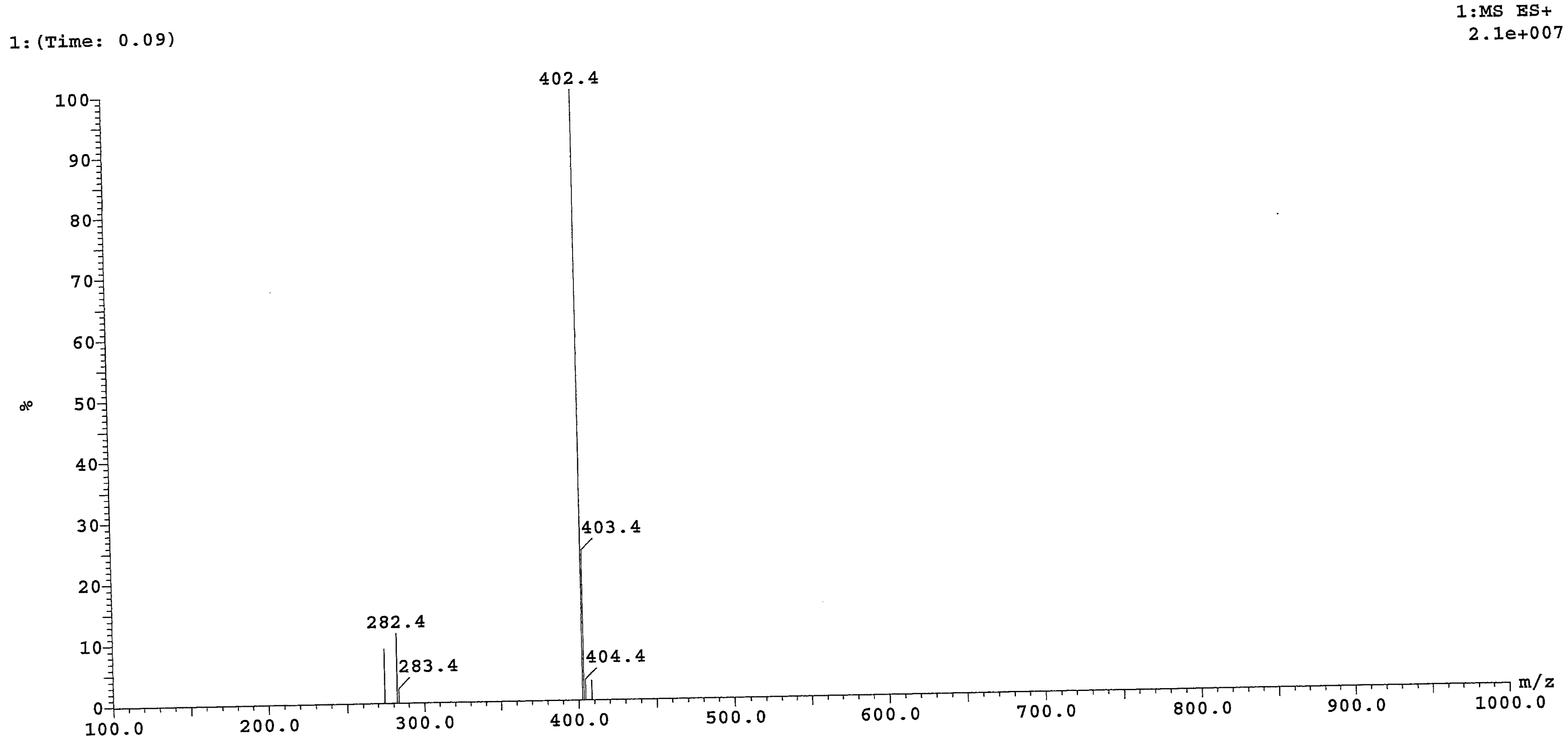

SAMPLE INFORMATION

Sample Name:	ARN-E-7-1
Injection Volume:	3.00 ul
Run Time:	9.0 Minutes
Date Acquired:	6/8/2022 10:13:49 AM EDT
Date Processed:	6/8/2022 10:44:17 AM EDT
Sample Set Name:	Template
Acq. Method Set:	BEH_C18_PDA_75mm 408
Processing Method:	BEH_C18_PDA_CAB
Channel Name:	254nm

Method Notes:
Acquity UPLC BEH C18 1.7u (2.1x75mm)
Flow Rate : 0.5 mL/min
Solvent A : 0.1% TFA in Waters
Solvent B : 0.1% TFA in Acetonitrile
Solvent Gradient Program:

Time (min)	%A	% B
0:00	95	5
6:00	0	100
8:00	0	100
9:00	95	5

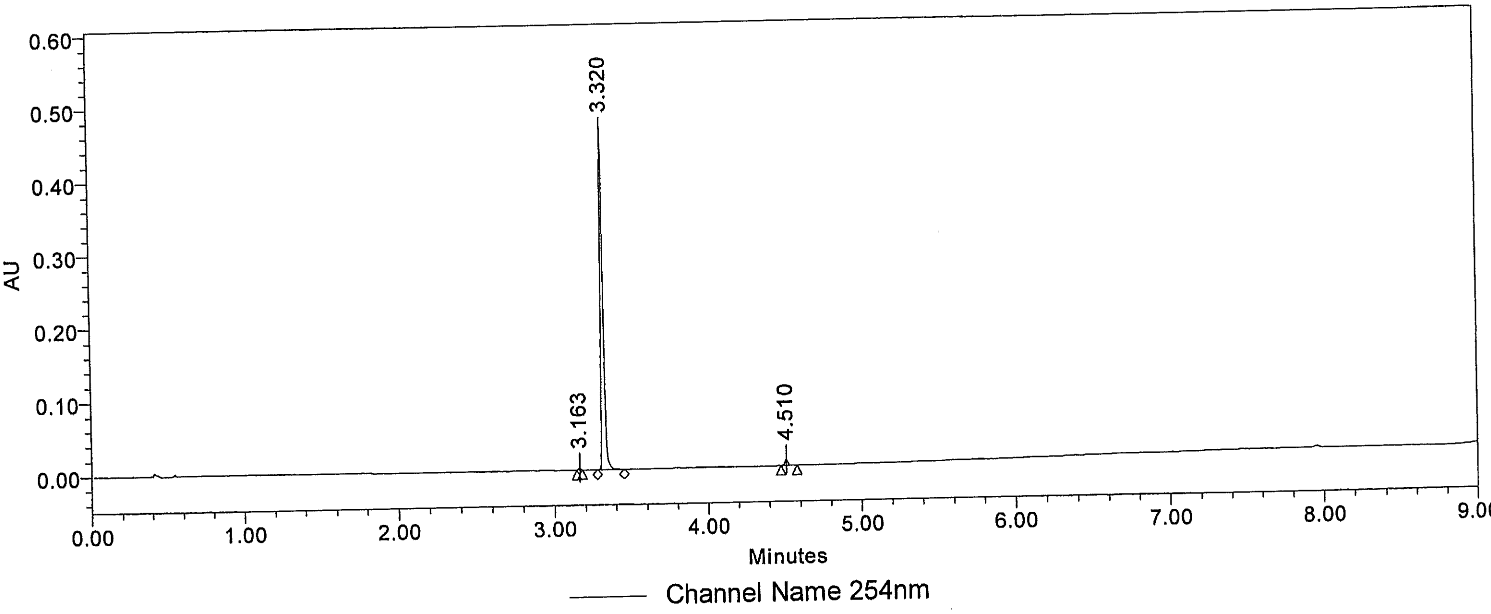

Peak Results

	RT	Area	Int Type	Width (sec)	% Area
1	3.163	2886	bb	2.100	0.50
2	3.320	560329	VV	10.549	97.52
3	4.510	11393	BB	6.251	1.98

Name: Corey Arnett-Butscher

Date: 6/8/2022

NB #: ARN-E-7-1

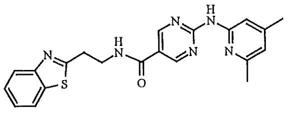

CERTIFICATE OF ANALYSIS

Compound Name: BPN-0035321-AA-001 1r
ALB Number: ALB-229928
Batch: 1
Lot Number: ALK-C-4-2
Molecular Formula: $C_{21}H_{20}N_6OS$
Molecular Weight: 404.49
Last Solvent: Ethyl Acetate

TEST	RESULT/REFERENCE
Appearance	Off-white Solid
NMR Spectrum	^{1}H, 500 MHz, Dimethyl Sulfoxide-d_6, Consistent - Attached
Mass Spectrum	ESI, m/z 405 $[M + H]^+$, Attached
UPLC	98.8% (area %), ACQUITY UPLC BEH C18 (2.1 *75) mm, 1.7 micron Column, UV 254 nm Detection, Attached

[illegible]
Approved By

6-8-2022
Date

For Research Purposes Only. Not Intended for Food or Drug Use.

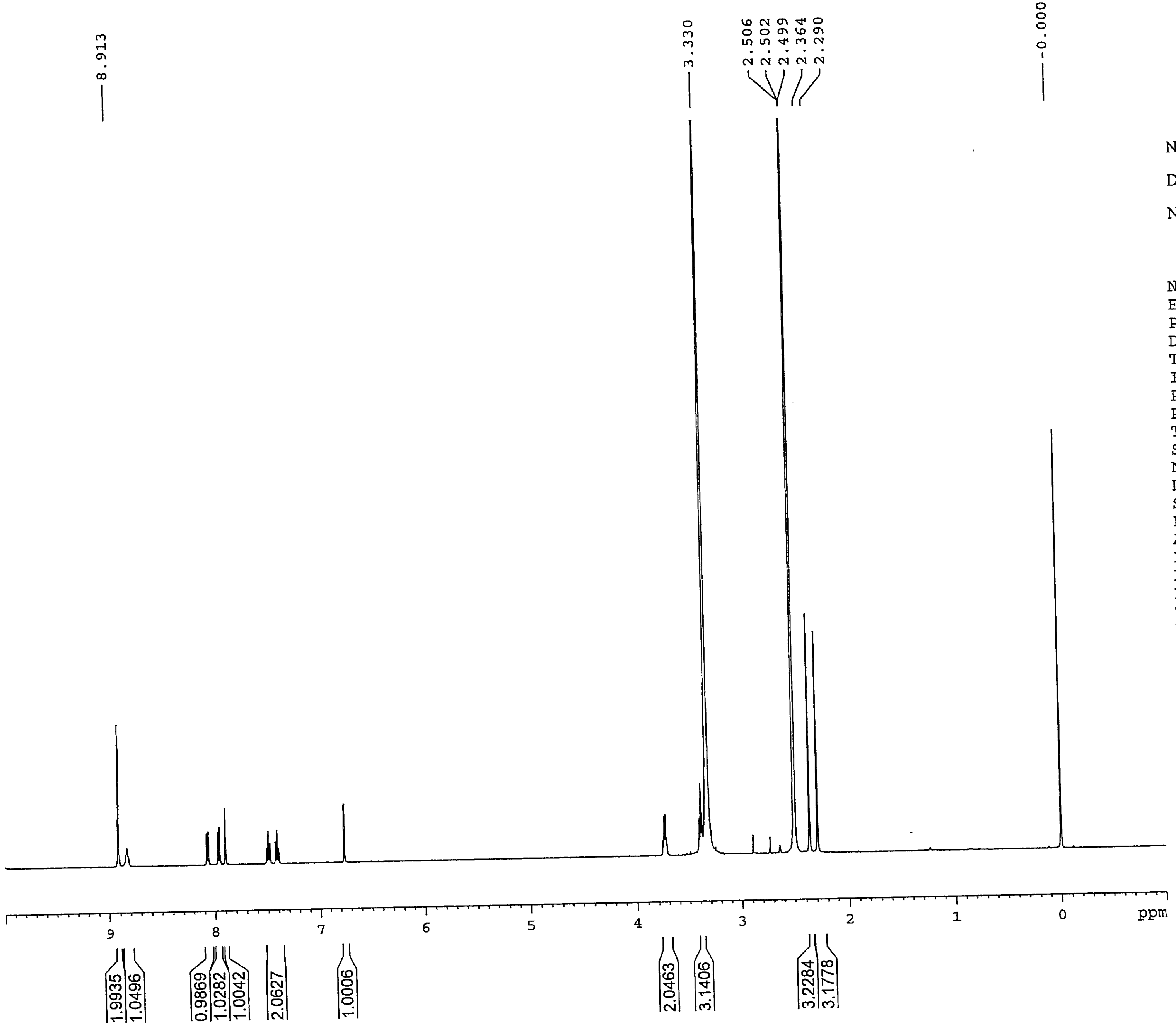

Name Marwah Albaker

Date 3-Jun 2022

NB # ALK-C-4-2

NAME	ALK-C-4-2
EXPNO	10
PROCNO	1
Date_	20220602
Time	9.22 h
INSTRUM	Avance Neo
PROBHD	Z167419_0029 (
PULPROG	zg30
TD	65536
SOLVENT	DMSO
NS	16
DS	2
SWH	10000.000 Hz
FIDRES	0.305176 Hz
AQ	3.2768500 sec
RG	101
DW	50.000 usec
DE	11.14 usec
TE	300.0 K
D1	1.00000000 sec
TD0	1
SFO1	500.1330883 MHz
NUC1	1H
P0	2.67 usec
P1	8.00 usec
SI	65536
SF	500.1300031 MHz
WDW	EM
SSB	0
LB	0.30 Hz
GB	0
PC	1.00

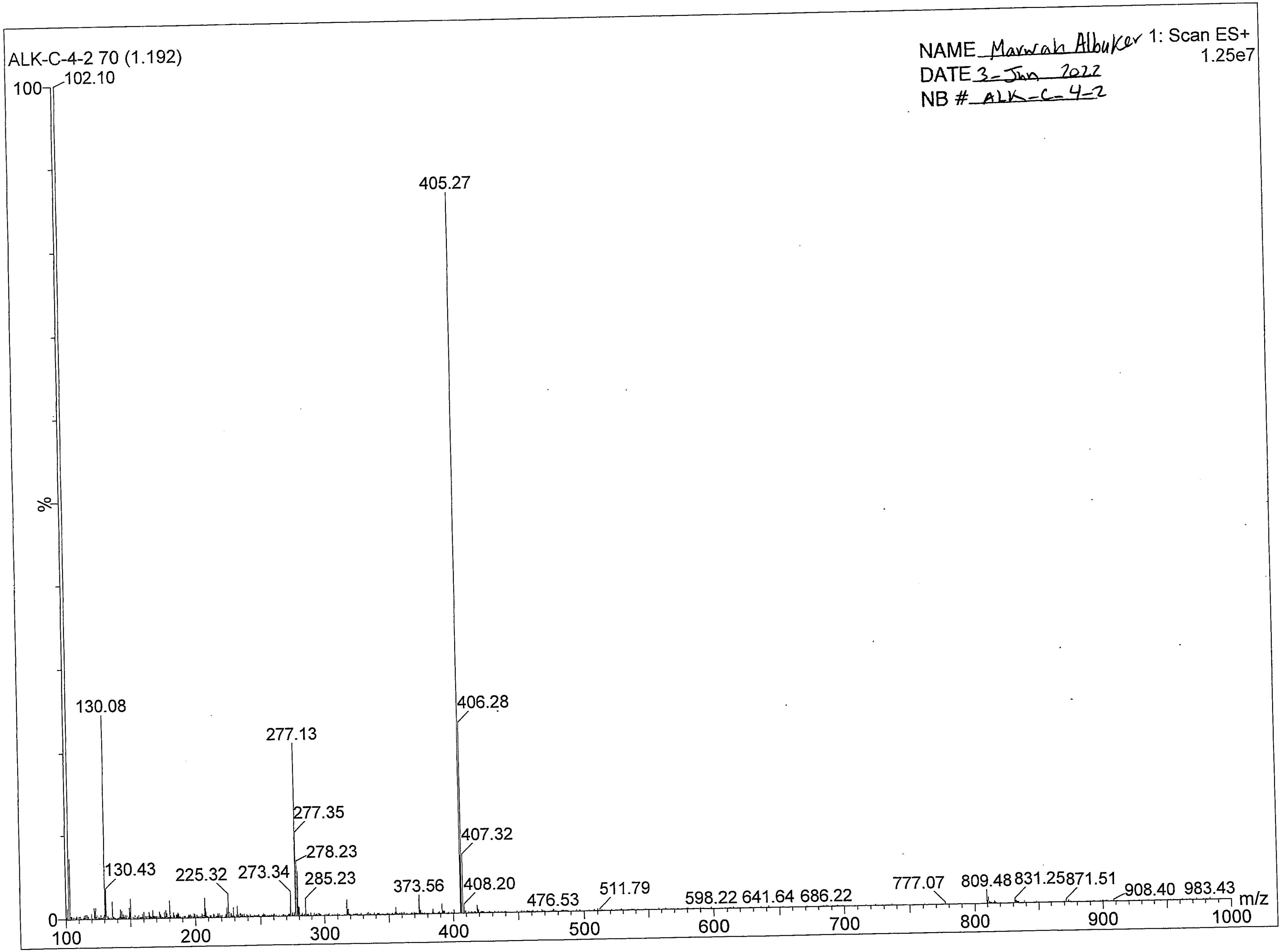
ALK-C-4-2 70 (1.192)
1: Scan ES+
1.25e7
NAME Marwah Albuker
DATE 3-Jun 2022
NB # ALK-C-4-2
100
%
0
102.10
130.08
130.43
225.32
273.34
277.13
277.35
278.23
285.23
373.56
405.27
406.28
407.32
408.20
476.53
511.79
598.22
641.64
686.22
777.07
809.48
831.25
871.51
908.40
983.43
100
200
300
400
500
600
700
800
900
1000
m/z

SAMPLE INFORMATION

Sample Name:	ALK-C-4-2
Injection Volume:	3.00 ul
Run Time:	9.0 Minutes
Date Acquired:	6/1/2022 3:51:27 PM EDT
Date Processed:	6/1/2022 4:06:25 PM EDT
Sample Set Name:	Template
Acq. Method Set:	HSS T3_PDA_75mm_polar 408
Processing Method:	BEH_C18_PDA
Channel Name:	254nm

Method Notes:
Acquity UPLC BEH C18 1.7u (2.1x75mm)
Flow Rate : 0.5 mL/min
Solvent A : 0.1% TFA in Waters
Solvent B : 0.1% TFA in Acetonitrile
Solvent Gradient Program:

Time (min)	%A	% B
0:00	95	5
6:00	0	100
8:00	0	100
9:00	95	5

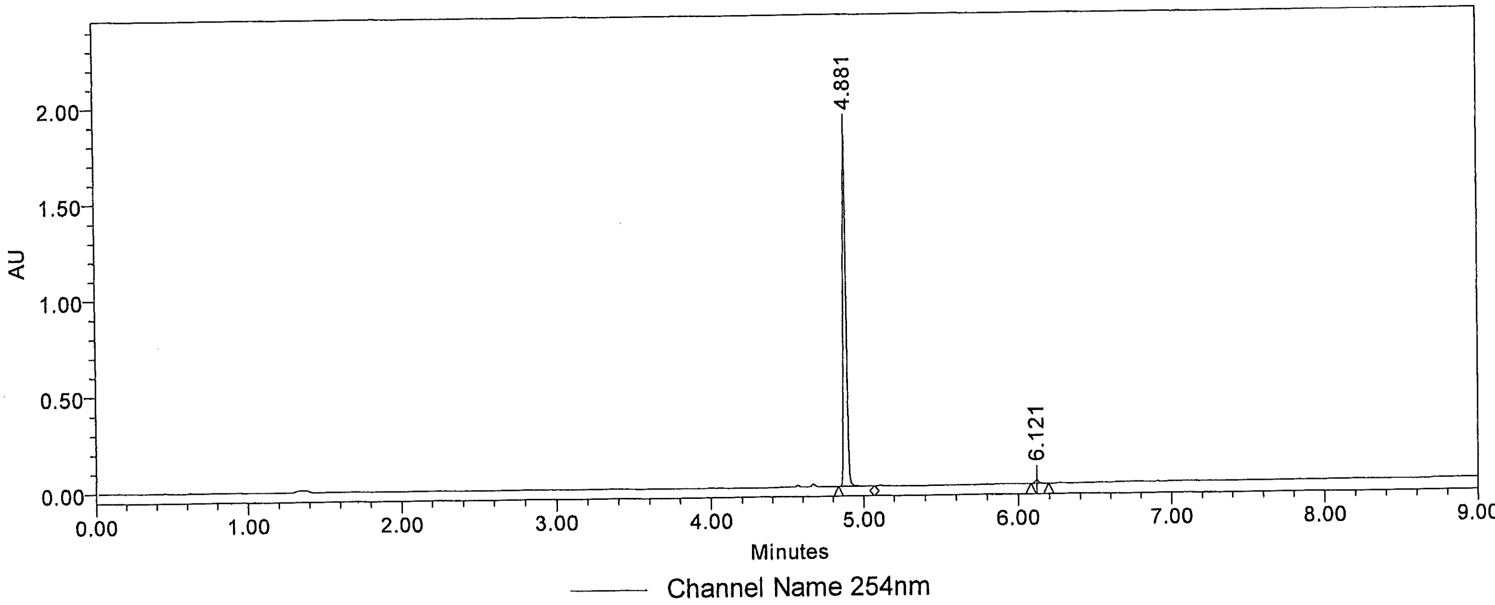

Peak Results

	RT	Area	Int Type	Width (sec)	% Area
1	4.881	2505996	BV	14.053	98.85
2	6.121	29027	BB	6.901	1.15

Name: Marwah Albaker

Date: 3-June 2022

NB #: ALK-C-4-2

CERTIFICATE OF ANALYSIS

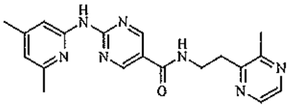

Compound Name: BPN-0035921-AA-001 1s
ALB Number: ALB-230105
Batch: 1
Lot Number: ARN-E-21-2
Molecular Formula: $C_{19}H_{21}N_7O$
Molecular Weight: 363.42
Last Solvent: Methylene Chloride

TEST	RESULT/REFERENCE
Appearance	Off-white Solid
NMR Spectrum	^{1}H, 500 MHz, Dimethyl Sulfoxide-d_6, Consistent - Attached
Mass Spectrum	ESI, *m/z* 364 $[M + H]^+$, Attached
UPLC	97.2% (area %), ACQUITY UPLC BEH C18 (2.1 *75) mm, 1.7 micron Column, UV 254 nm Detection, Attached

Approved By

Date 6-22-2022

For Research Purposes Only. Not Intended for Food or Drug Use.

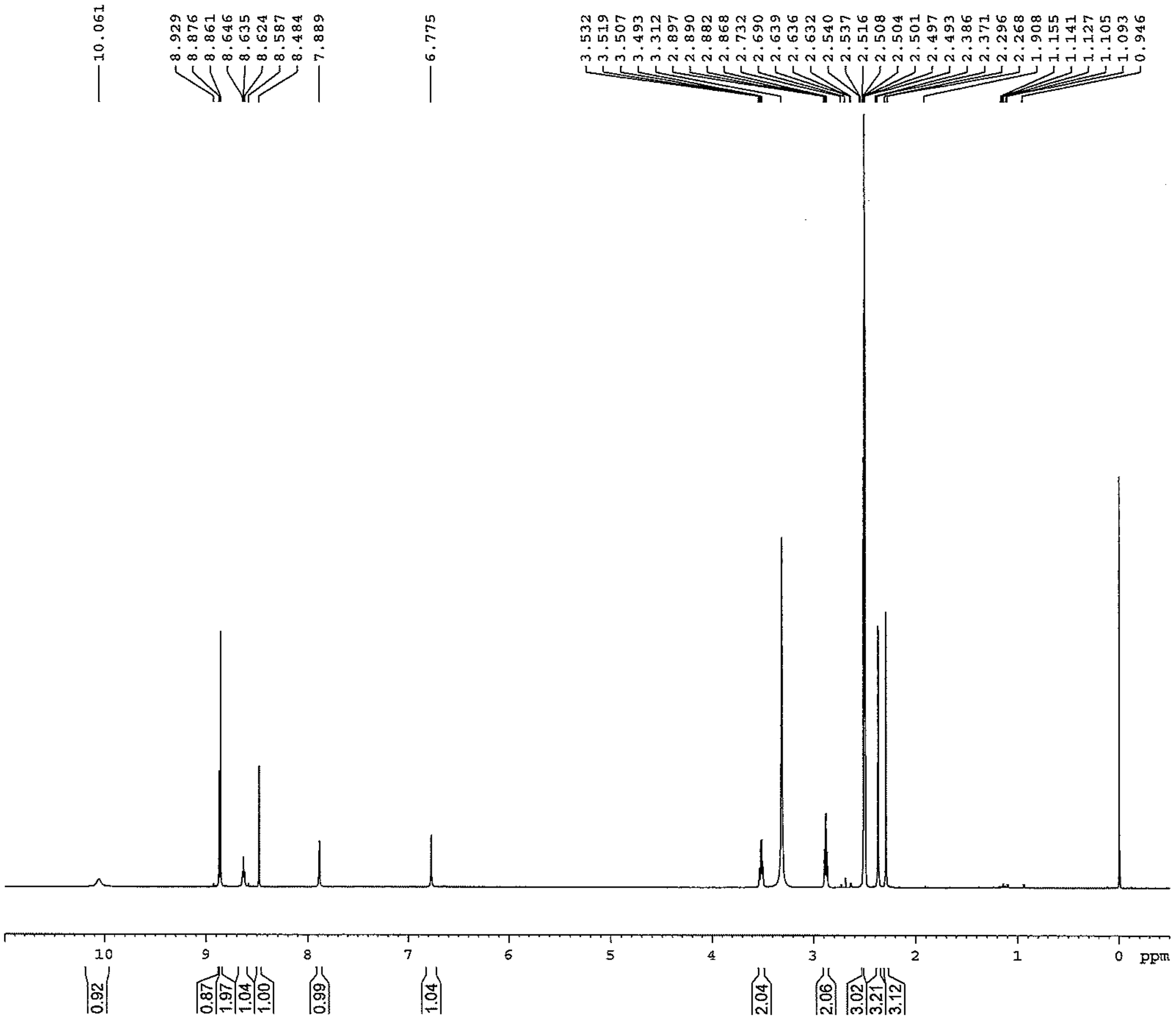

Name Corey Arnett-Butscher

Date 6/20/2021

NB # ARN-E-21-2

NAME	ARN-E-21-2
EXPNO	10
PROCNO	1
Date_	20220620
Time	10.07 h
INSTRUM	Avance Neo
PROBHD	Z167419_0029 (
PULPROG	zg30
TD	65536
SOLVENT	DMSO
NS	32
DS	2
SWH	10000.000 Hz
FIDRES	0.305176 Hz
AQ	3.2768500 sec
RG	101
DW	50.000 usec
DE	11.14 usec
TE	300.0 K
D1	1.00000000 sec
TD0	1
SFO1	500.1330883 MHz
NUC1	1H
P0	2.67 usec
P1	8.00 usec
SI	65536
SF	500.1300038 MHz
WDW	EM
SSB	0
LB	0.30 Hz
GB	0
PC	1.00

Openlynx Report Page 1

Vial:1:20
Date:17-Jun-2022

ID:
Time:10:10:15

File:ARN-E-21-2

Name: Corey Arnett-Betscher

Date: 6/20/2022

Notebook: ARN-E-21-2

Printed: Fri Jun 17 10:12:33 2022

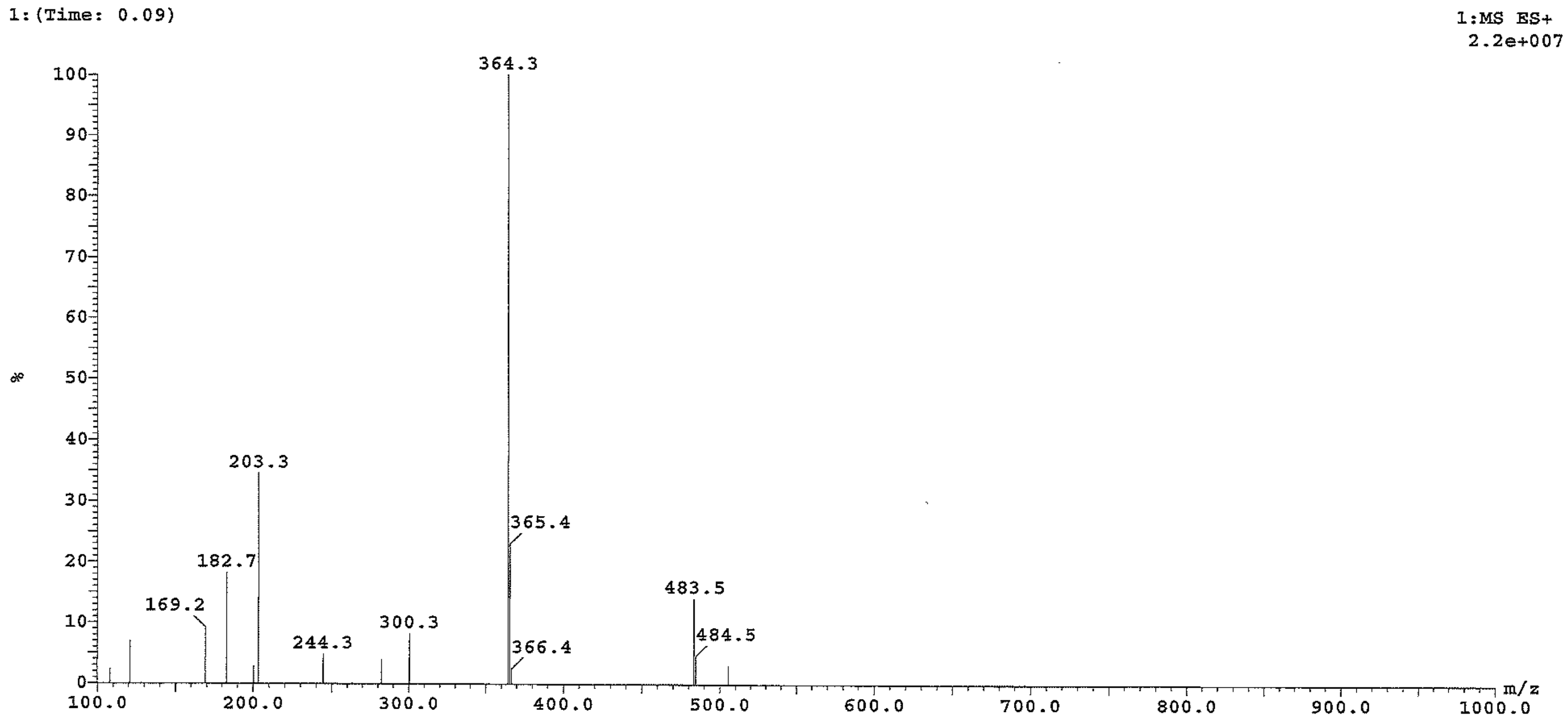

SAMPLE INFORMATION

Sample Name: ARN-E-21-2
Injection Volume: 3.00 ul
Run Time: 9.0 Minutes
Date Acquired: 6/17/2022 9:38:37 AM EDT
Date Processed: 6/17/2022 10:07:37 AM EDT
Sample Set Name: Template
Acq. Method Set: BEH_C18_PDA_75mm 408
Processing Method: BEH_C18_PDA_CAB
Channel Name: 254nm

Method Notes:
Acquity UPLC BEH C18 1.7u (2.1x75mm)
Flow Rate : 0.5 mL/min
Solvent A : 0.1% TFA in Waters
Solvent B : 0.1% TFA in Acetonitrile
Solvent Gradient Program:

Time (min)	%A	% B
0:00	95	5
6:00	0	100
8:00	0	100
9:00	95	5

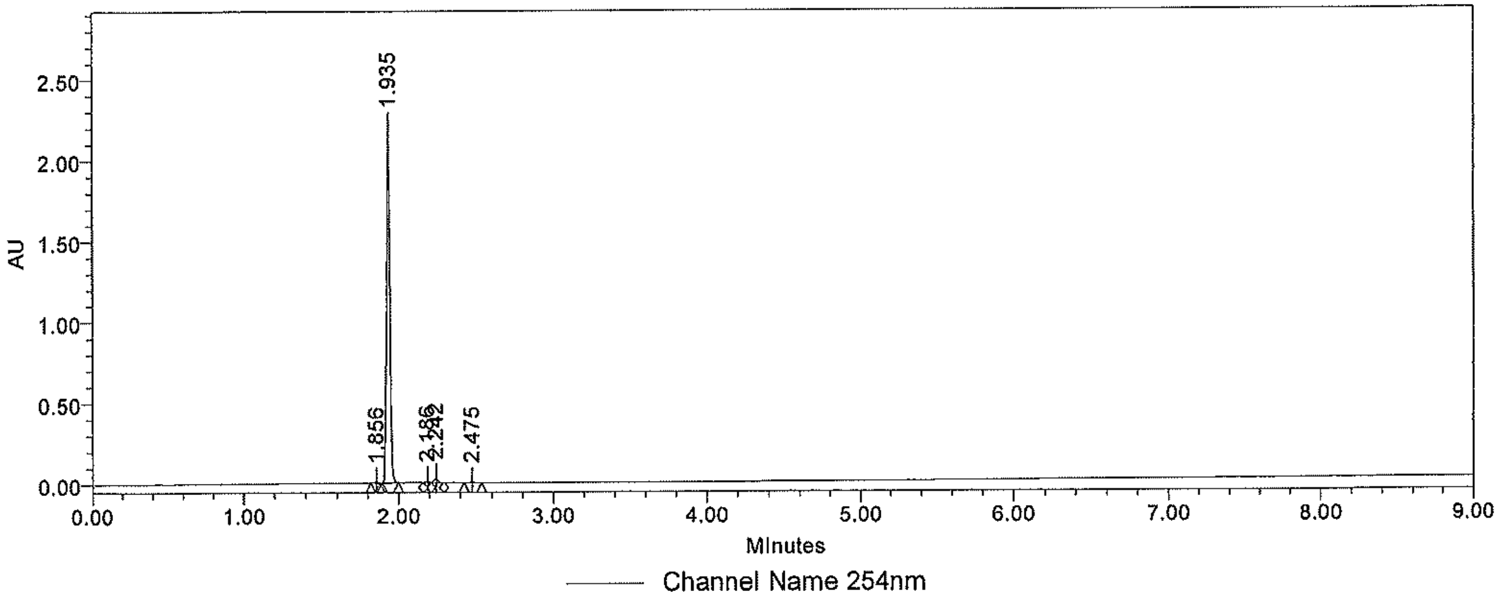

Peak Results

	RT	Area	Int Type	Width (sec)	% Area
1	1.856	10974	bb	3.450	0.34
2	1.935	3176153	bb	6.250	97.29
3	2.186	19305	VV	3.100	0.59
4	2.242	47149	VV	4.900	1.44
5	2.475	11012	BB	6.750	0.34

Name: Cory Arnett-Butscher

Date: 6/20/2022

NB #: ARN-E-21-2

CERTIFICATE OF ANALYSIS

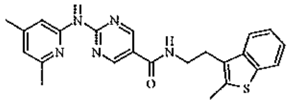

Compound Name: BPN-0035916-AA-001 1t
ALB Number: ALB-230106
Batch: 1
Lot Number: ARN-E-13-1
Molecular Formula: $C_{23}H_{23}N_5OS$
Molecular Weight: 417.53
Last Solvent: Ethyl Acetate, Water

TEST	RESULT/REFERENCE
Appearance	White Solid
NMR Spectrum	^{1}H, 500 MHz, Dimethyl Sulfoxide-d_6, Consistent - Attached
Mass Spectrum	ESI, m/z 418 $[M + H]^+$, Attached
UPLC	95.9% (area %), ACQUITY UPLC BEH C18 (2.1 *75) mm, 1.7 micron Column, UV 254 nm Detection, Attached

Maria Maychack

Approved By

6-22-2022

Date

For Research Purposes Only. Not Intended for Food or Drug Use.

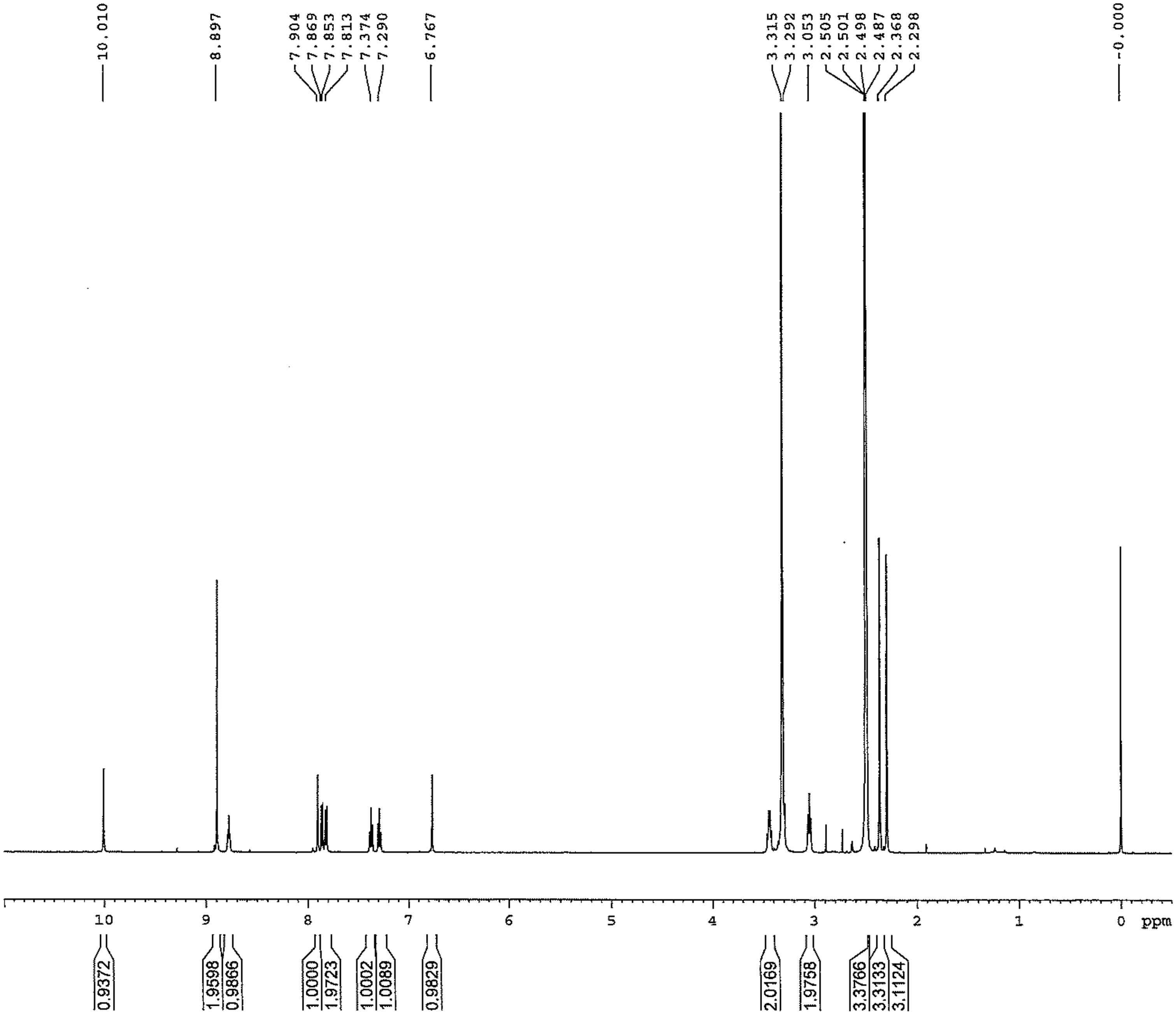

Name Cory Arnett-Butscher

Date 6/17/2022

NB # ARN-E-13-1

NAME	ARN-E-13-1	
EXPNO	10	
PROCNO	1	
Date_	20220615	
Time	7.48	h
INSTRUM	Avance Neo	
PROBHD	Z167419_0029 (	
PULPROG	zg30	
TD	65536	
SOLVENT	DMSO	
NS	32	
DS	2	
SWH	10000.000	Hz
FIDRES	0.305176	Hz
AQ	3.2768500	sec
RG	101	
DW	50.000	usec
DE	11.14	usec
TE	300.0	K
D1	1.00000000	sec
TD0	1	
SFO1	500.1330883	MHz
NUC1	1H	
P0	2.67	usec
P1	8.00	usec
SI	65536	
SF	500.1300037	MHz
WDW	EM	
SSB	0	
LB	0.30	Hz
GB	0	
PC	1.00	

Openlynx Report
Vial:1:28
Date:16-Jun-2022
Name: Cory Arnett-Butscher

ID:
Time:10:37:40
Date: 6/7/2022

File:ARN-E-13-1
Notebook: ARN-E-13-1

Printed: Thu Jun 16 10:56:48 2022

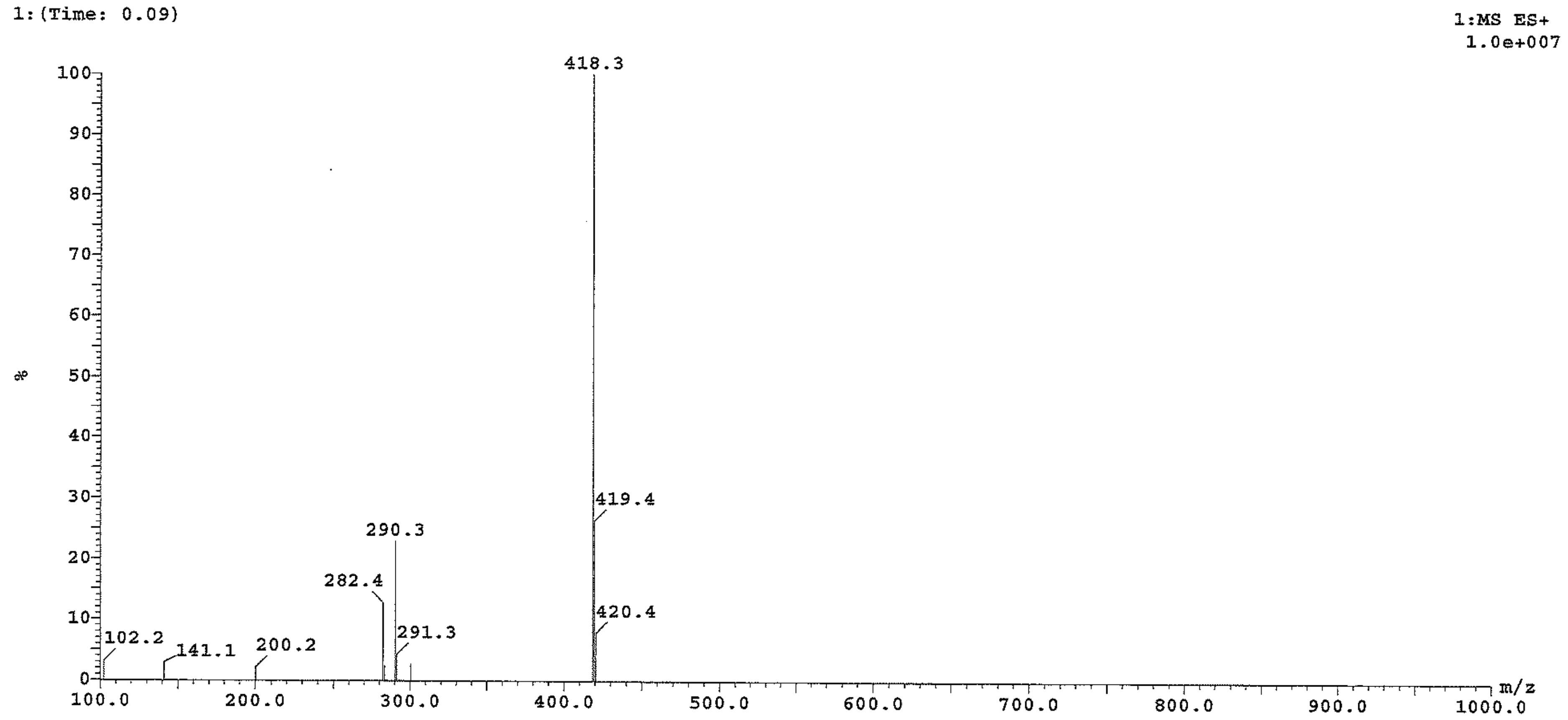

SAMPLE INFORMATION

Sample Name: ARN-E-13-1
Injection Volume: 3.00 ul
Run Time: 9.0 Minutes
Date Acquired: 6/16/2022 9:57:20 AM EDT
Date Processed: 6/16/2022 10:29:36 AM EDT
Sample Set Name: Template
Acq. Method Set: BEH_C18_PDA_75mm 408
Processing Method: BEH_C18_PDA_CAB
Channel Name: 254nm

Method Notes:
Acquity UPLC BEH C18 1.7u (2.1x75mm)
Flow Rate : 0.5 mL/min
Solvent A : 0.1% TFA in Waters
Solvent B : 0.1% TFA in Acetonitrile
Solvent Gradient Program:

Time (min)	%A	% B
0:00	95	5
6:00	0	100
8:00	0	100
9:00	95	5

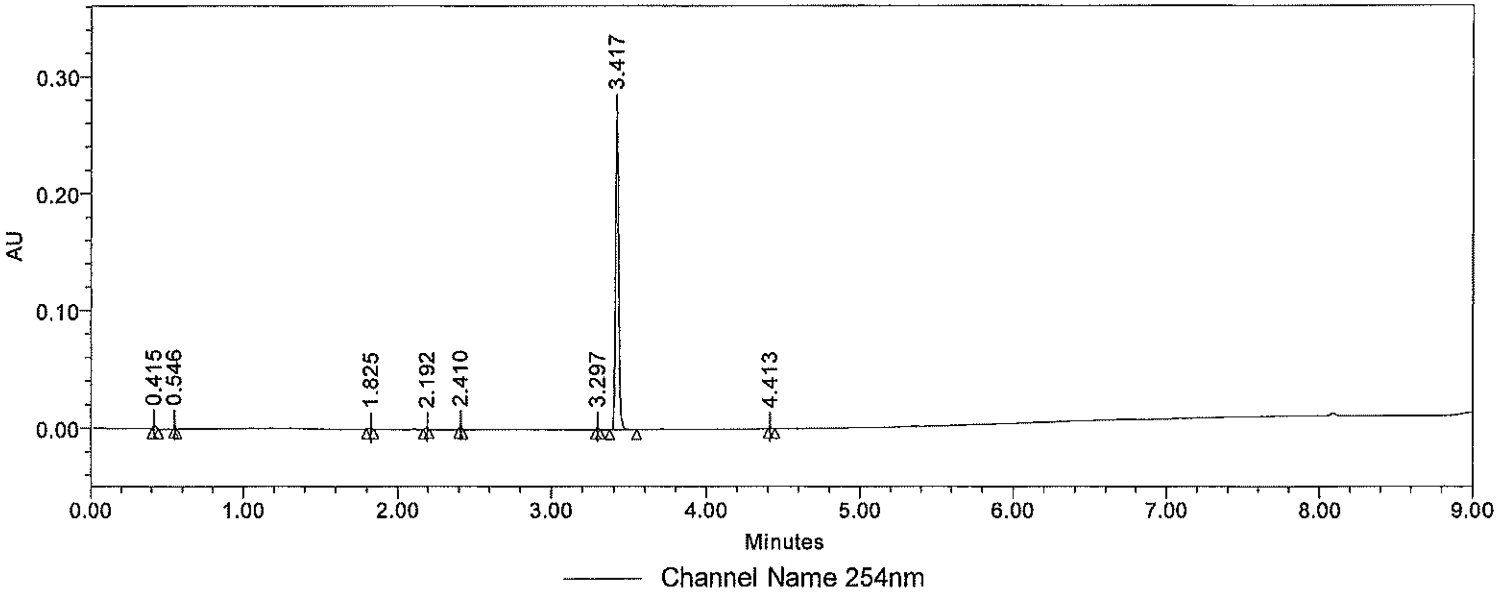

Peak Results

	RT	Area	Int Type	Width (sec)	% Area
1	0.415	4352	bb	2.400	1.12
2	0.546	1769	bb	1.300	0.45
3	1.825	1375	bb	2.800	0.35
4	2.192	1863	bb	2.150	0.48
5	2.410	3062	bb	1.950	0.79
6	3.297	2050	bb	1.750	0.53
7	3.417	373557	BB	10.399	95.98
8	4.413	1155	bb	2.601	0.30

Name: Corey Arnett-Butscher
Date: 6/17/2022
NB #: ARN-E-13-1

CERTIFICATE OF ANALYSIS

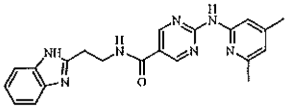

Compound Name: BPN-0035918-AA-001 1u
ALB Number: ALB-230102
Batch: 1
Lot Number: ALK-C-13-2
Molecular Formula: $C_{21}H_{21}N_7O$
Molecular Weight: 387.44
Last Solvent: Methylene Chloride, Methanol

TEST	RESULT/REFERENCE
Appearance	Off-white Solid
NMR Spectrum	^1H, 500 MHz, Dimethyl Sulfoxide-d_6, Consistent - Attached
Mass Spectrum	ESI, m/z 388 $[M + H]^+$, Attached
UPLC	95.0% (area %), ACQUITY UPLC BEH C18 (2.1 *75) mm, 1.7 micron Column, UV 254 nm Detection, Attached

[signature]
Approved By

6-22-2022
Date

For Research Purposes Only. Not Intended for Food or Drug Use.

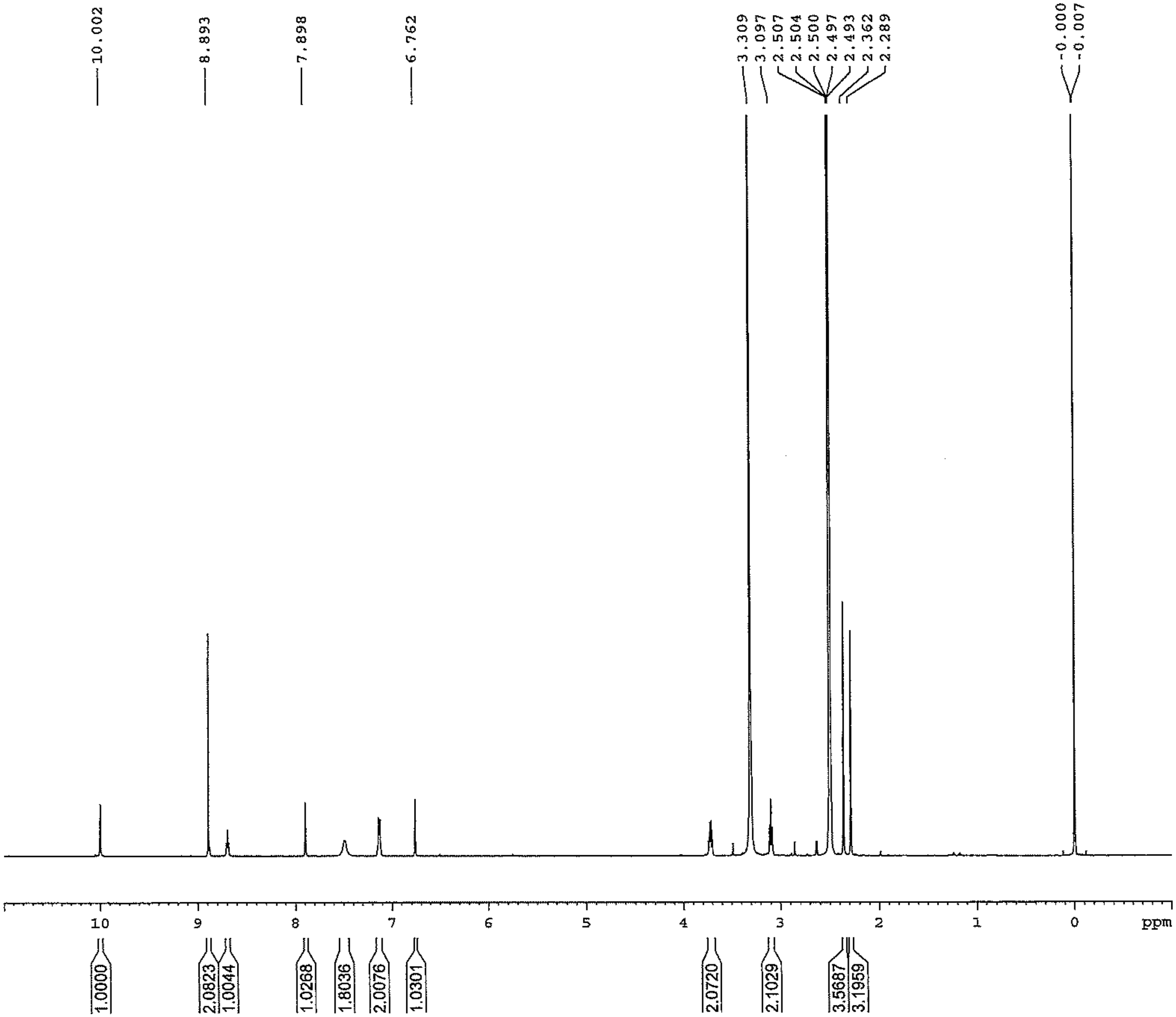

Name Marwah Albaker

Date 14 - Jun 2022

NB # ALK-C-13-2

NAME	ALK-C-13-2
EXPNO	10
PROCNO	1
Date_	20220615
Time	15.46 h
INSTRUM	Avance Neo
PROBHD	Z167419_0029 (
PULPROG	zg30
TD	65536
SOLVENT	DMSO
NS	64
DS	2
SWH	10000.000 Hz
FIDRES	0.305176 Hz
AQ	3.2768500 sec
RG	101
DW	50.000 usec
DE	11.14 usec
TE	300.0 K
D1	1.00000000 sec
TD0	1
SFO1	500.1330883 MHz
NUC1	1H
P0	2.67 usec
P1	8.00 usec
SI	65536
SF	500.1300041 MHz
WDW	EM
SSB	0
LB	0.30 Hz
GB	0
PC	1.00

ACQ-SQD#F07SQD100W

15-Jun-2022
13:56:42

Name: Marwah Albaker
Date: 15-Jun-2022
Notebook: ALK-C-13-2

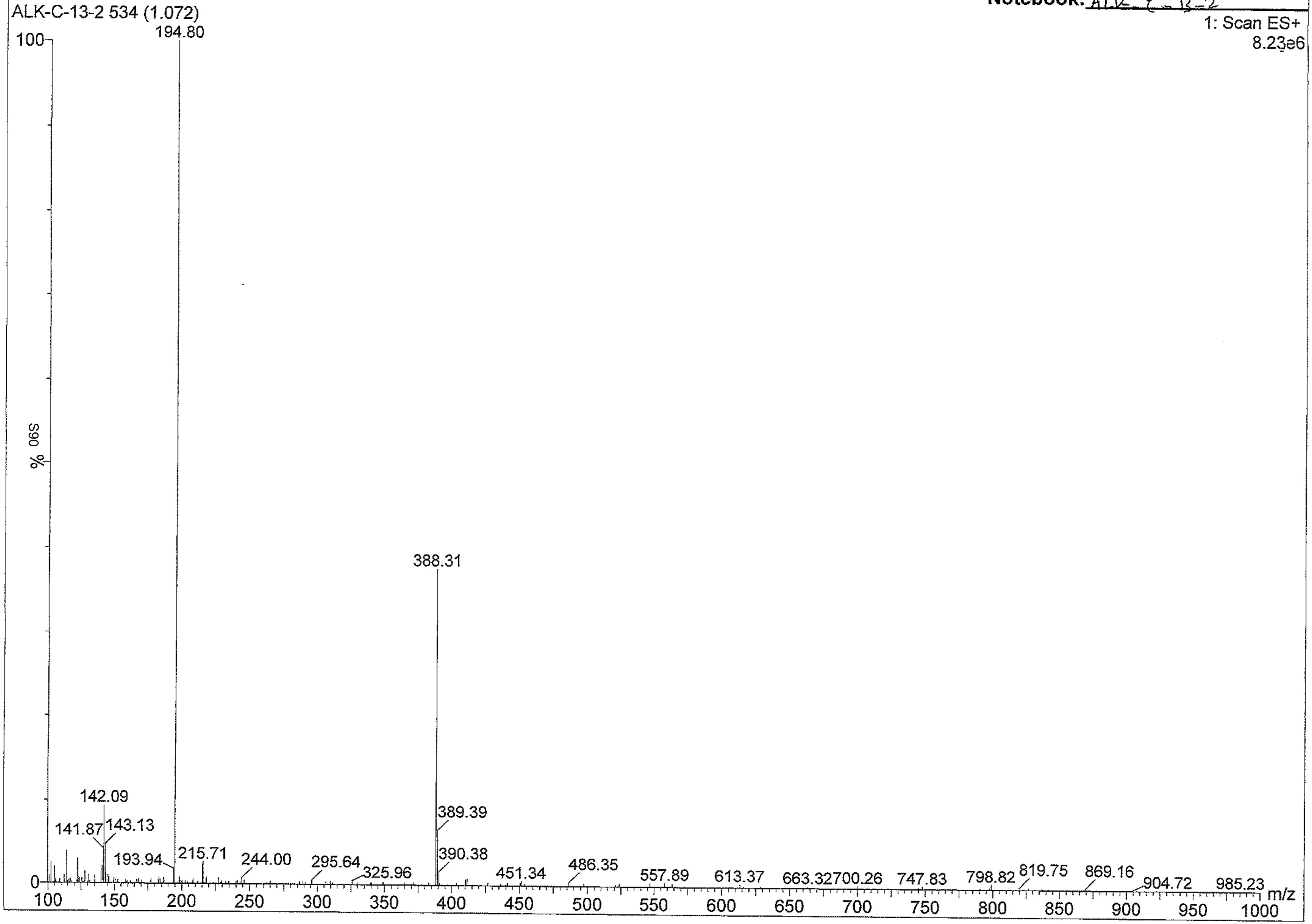

SAMPLE INFORMATION

Sample Name:	ALK-C-13-2
Injection Volume:	3.00 ul
Run Time:	9.0 Minutes
Date Acquired:	6/15/2022 2:05:15 PM EDT
Date Processed:	6/15/2022 2:19:19 PM EDT
Sample Set Name:	Template
Acq. Method Set:	HSS T3_PDA_75mm_polar 408
Processing Method:	BEH_C18_PDA
Channel Name:	254nm

Method Notes:
Acquity UPLC HSS T3 1.8u (2.1x75mm)
Flow Rate : 0.5 mL/min
Solvent A : 0.1% TFA in Waters
Solvent B : 0.1% TFA in Acetonitrile
Solvent Gradient Program:

Time (min)	%A	% B
0:00	95	5
1:00	95	5
3:30	70	30
6:00	0	100
8:00	0	100
9:00	95	5

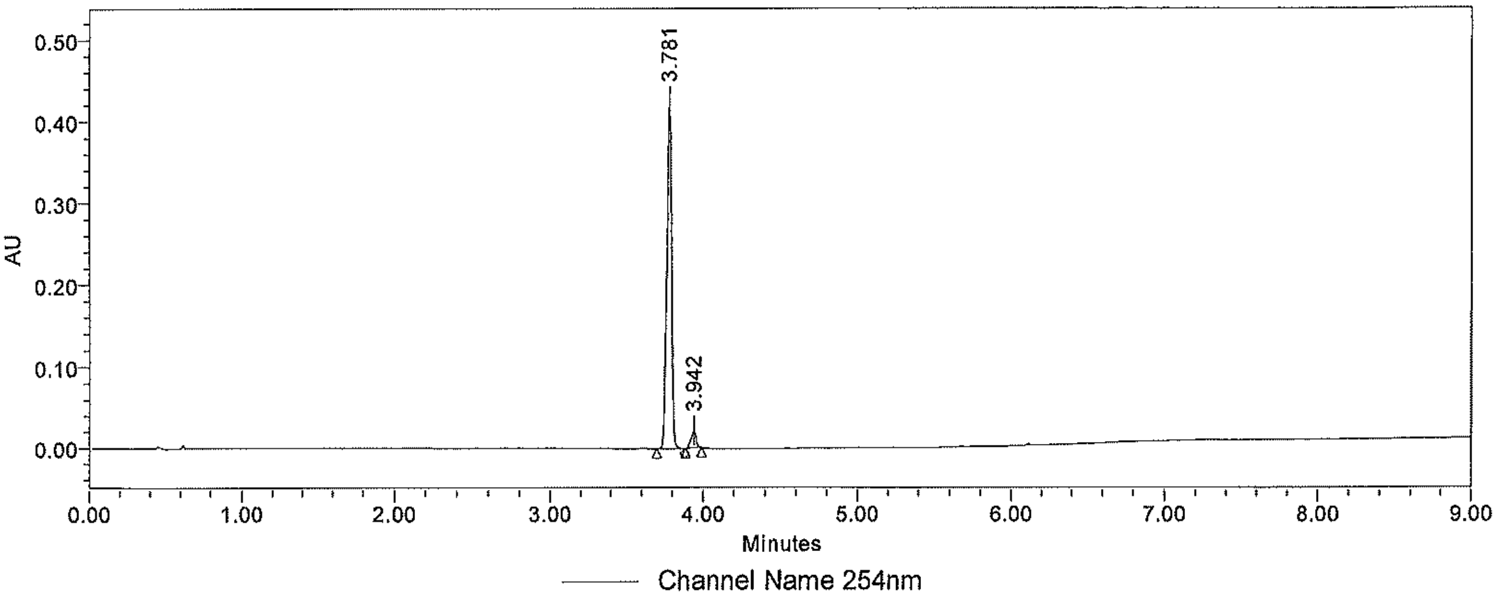

Peak Results

	RT	Area	Int Type	Width (sec)	% Area
1	3.781	905757	BV	11.099	95.02
2	3.942	47503	bb	6.300	4.98

Name: Marwah Albaker

Date: 15 Jun 2022

NB #: ALK-C-13-2

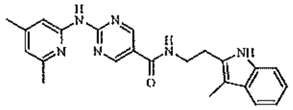

CERTIFICATE OF ANALYSIS

Compound Name: BPN-0035919-AA-001 1v
ALB Number: ALB-230103
Batch: 1
Lot Number: ARN-E-15-2
Molecular Formula: $C_{23}H_{24}N_6O$
Molecular Weight: 400.48
Last Solvent: Methylene Chloride

TEST	RESULT/REFERENCE
Appearance	Light Yellow Solid
NMR Spectrum	^{1}H, 500 MHz, Dimethyl Sulfoxide-d_6, Consistent - Attached
Mass Spectrum	ESI, *m/z* 399 [M – H]$^-$, Attached
UPLC	95.6% (area %), ACQUITY UPLC BEH C18 (2.1 *75) mm, 1.7 micron Column, UV 254 nm Detection, Attached

Approved By: [signature]

Date: 6-22-2022

For Research Purposes Only. Not Intended for Food or Drug Use.

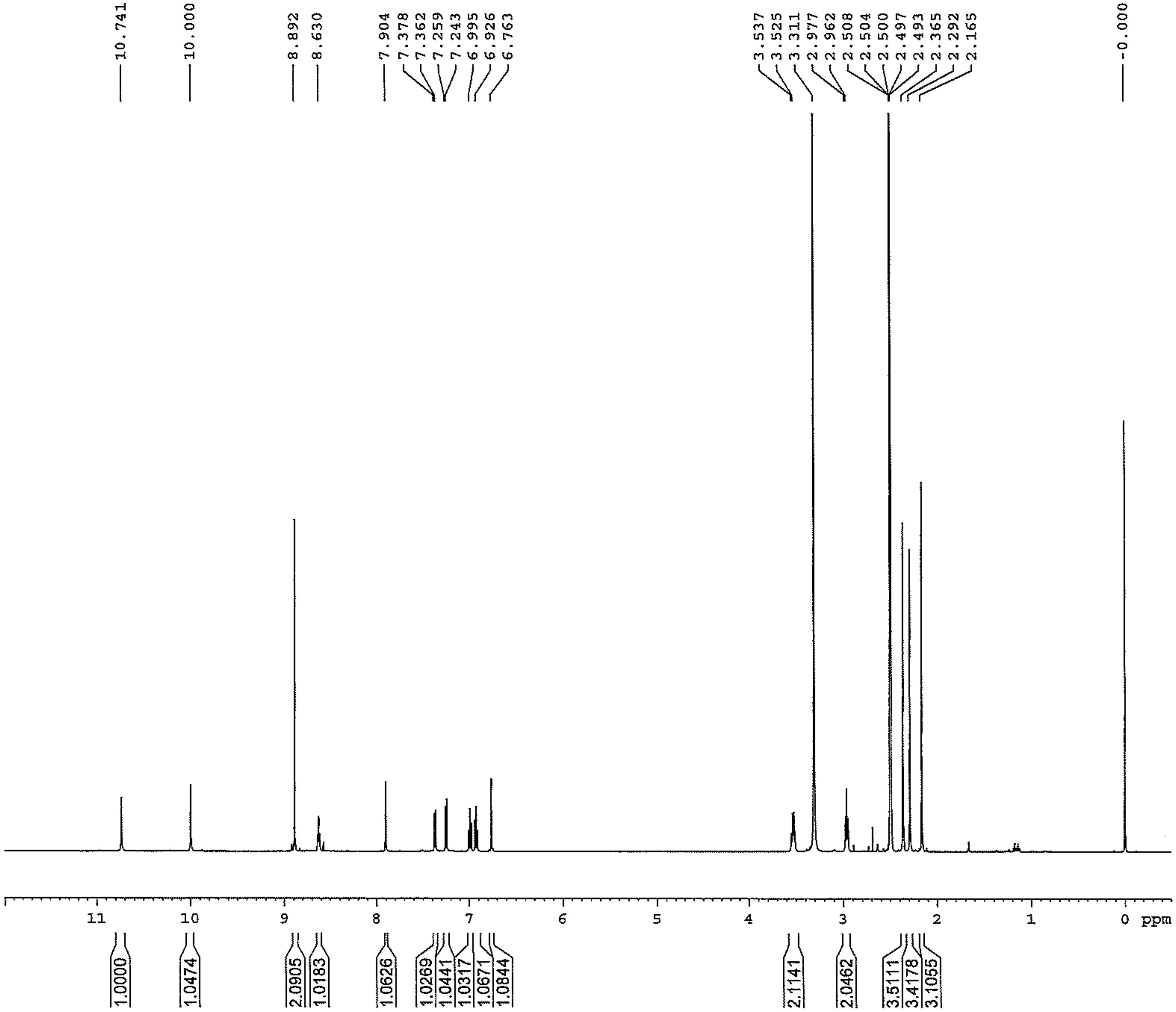

Name Cory Arnott-Butscher

Date 6/17/2022

NB # ARN-E-15-2

NAME	ARN-E-15-2
EXPNO	10
PROCNO	1
Date_	20220617
Time	8.22 h
INSTRUM	Avance Neo
PROBHD	Z167419_0029 (
PULPROG	zg30
TD	65536
SOLVENT	DMSO
NS	32
DS	2
SWH	10000.000 Hz
FIDRES	0.305176 Hz
AQ	3.2768500 sec
RG	101
DW	50.000 usec
DE	11.14 usec
TE	300.0 K
D1	1.00000000 sec
TD0	1
SFO1	500.1330883 MHz
NUC1	1H
P0	2.67 usec
P1	8.00 usec
SI	65536
SF	500.1300040 MHz
WDW	EM
SSB	0
LB	0.30 Hz
GB	0
PC	1.00

Openlynx Report
Vial:1:31
Date:16-Jun-2022

ID:
Time:13:16:22

File:ARN-E-15-2

Name: Corey Arnett-Butscher

Date: 6/17/2022

Notebook: ARN-E-15-2

Printed: Thu Jun 16 13:18:12 2022

3:(Time: 0.09) Combine (1:4)

2:MS ES-
1.8e+006

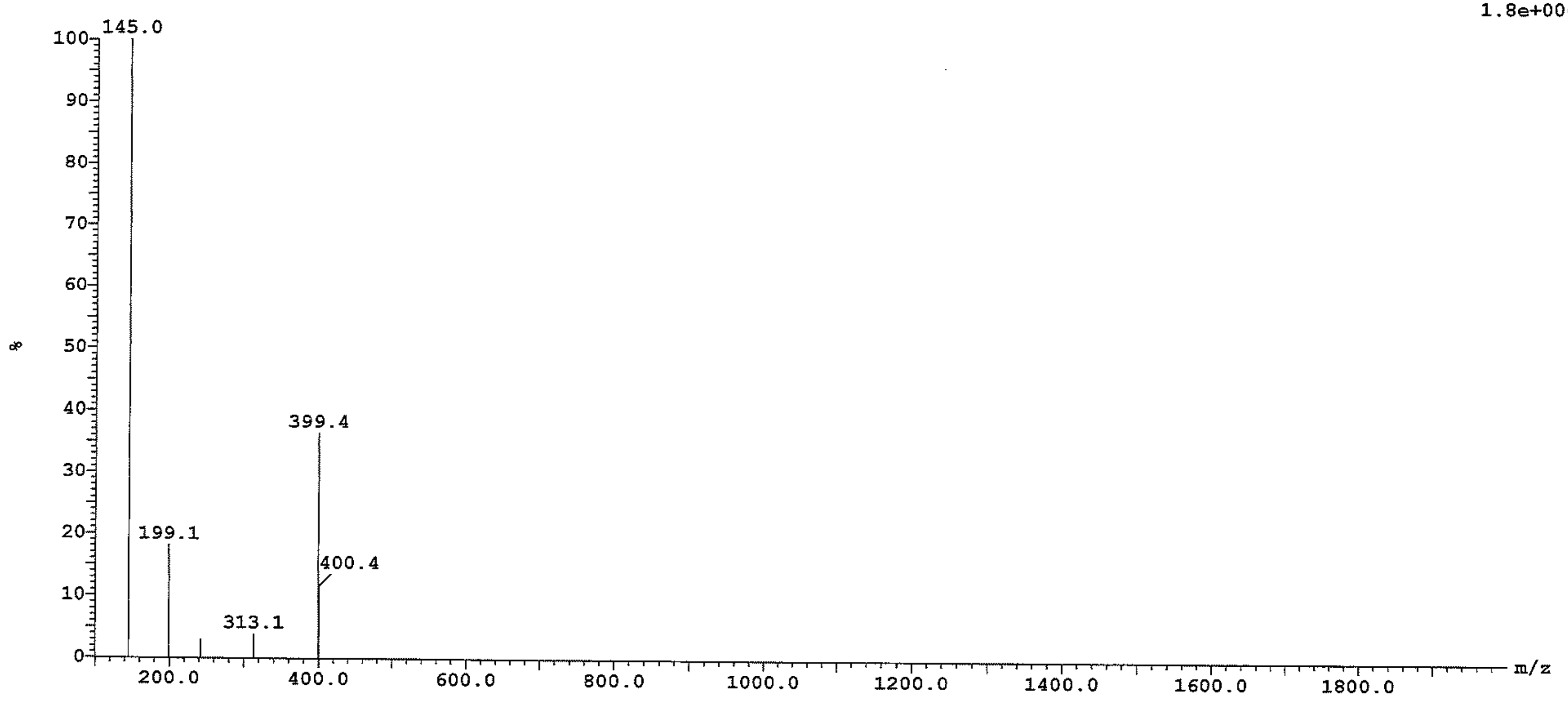

SAMPLE INFORMATION

Sample Name:	ARN-E-15-2
Injection Volume:	3.00 ul
Run Time:	9.0 Minutes
Date Acquired:	6/16/2022 10:41:44 AM EDT
Date Processed:	6/16/2022 1:11:38 PM EDT
Sample Set Name:	Template
Acq. Method Set:	BEH_C18_PDA_75mm 408
Processing Method:	BEH_C18_PDA_CAB
Channel Name:	254nm

Method Notes:
Acquity UPLC BEH C18 1.7u (2.1x75mm)
Flow Rate : 0.5 mL/min
Solvent A : 0.1% TFA in Waters
Solvent B : 0.1% TFA in Acetonitrile
Solvent Gradient Program:

Time (min)	%A	% B
0:00	95	5
6:00	0	100
8:00	0	100
9:00	95	5

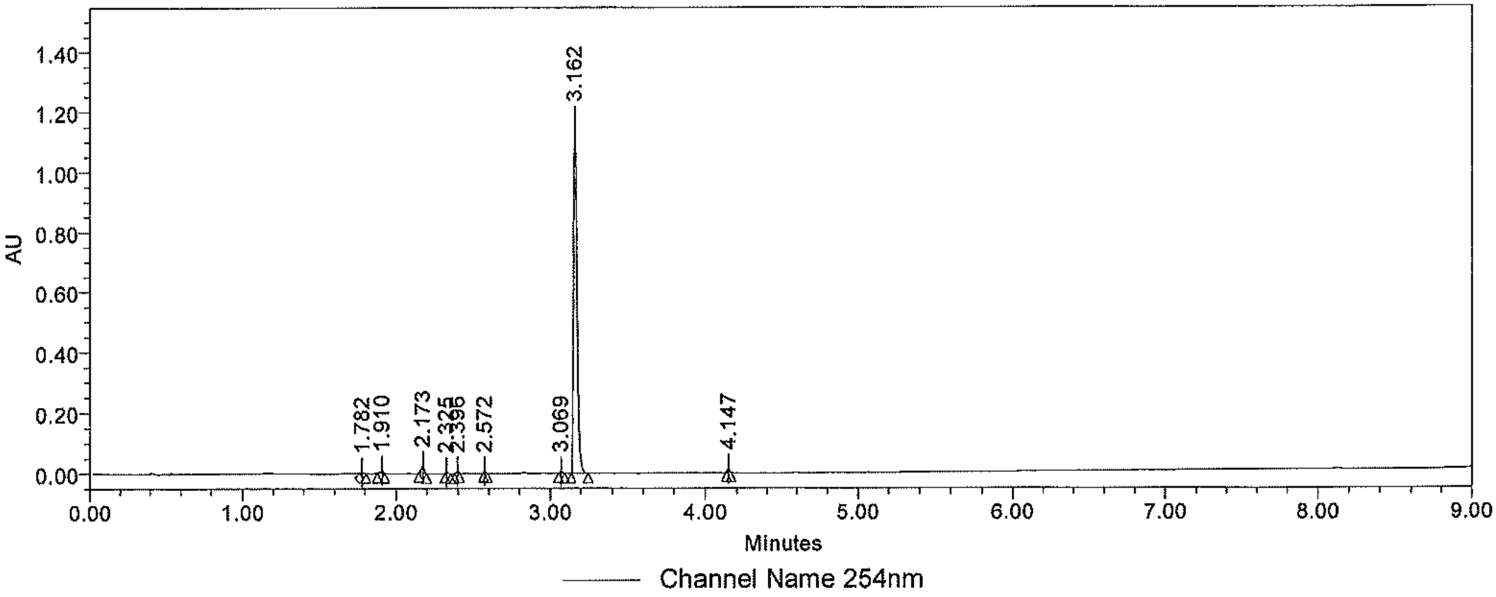

Peak Results

	RT	Area	Int Type	Width (sec)	% Area
1	1.782	6151	Vb	2.650	0.32
2	1.910	12690	bb	2.900	0.67
3	2.173	32042	bb	2.900	1.69
4	2.325	4529	bb	2.150	0.24
5	2.396	6552	bb	1.950	0.34
6	2.572	3871	bb	1.550	0.20
7	3.069	7057	bb	2.300	0.37
8	3.162	1816953	bb	6.600	95.62
9	4.147	10368	bb	1.800	0.55

Name: Cory Arnett-Butscher

Date: 6/17/2022

NB #: ARN-E-15-2

CERTIFICATE OF ANALYSIS

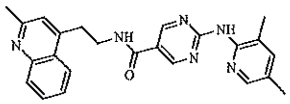

Compound Name: BPN-0035939-AA-001 1w
ALB Number: ALB-230207
Batch: 1
Lot Number: QUA-B-9-1
Molecular Formula: $C_{24}H_{24}N_6O$
Molecular Weight: 412.49
Last Solvent: Methanol, Methylene Chloride

TEST	RESULT/REFERENCE
Appearance	Gray Solid
NMR Spectrum	^{1}H, 300 MHz, Dimethyl Sulfoxide-d_6, Consistent - Attached
Mass Spectrum	ESI, *m/z* 413 $[M + H]^+$, Attached
HPLC Analysis	97.2% (area %), ACQUITY UPLC BEH C18 Column, UV 254 nm Detection, Attached

Approved By: [signature]

Date: 6-29-2022

For Research Purposes Only. Not Intended for Food or Drug Use.

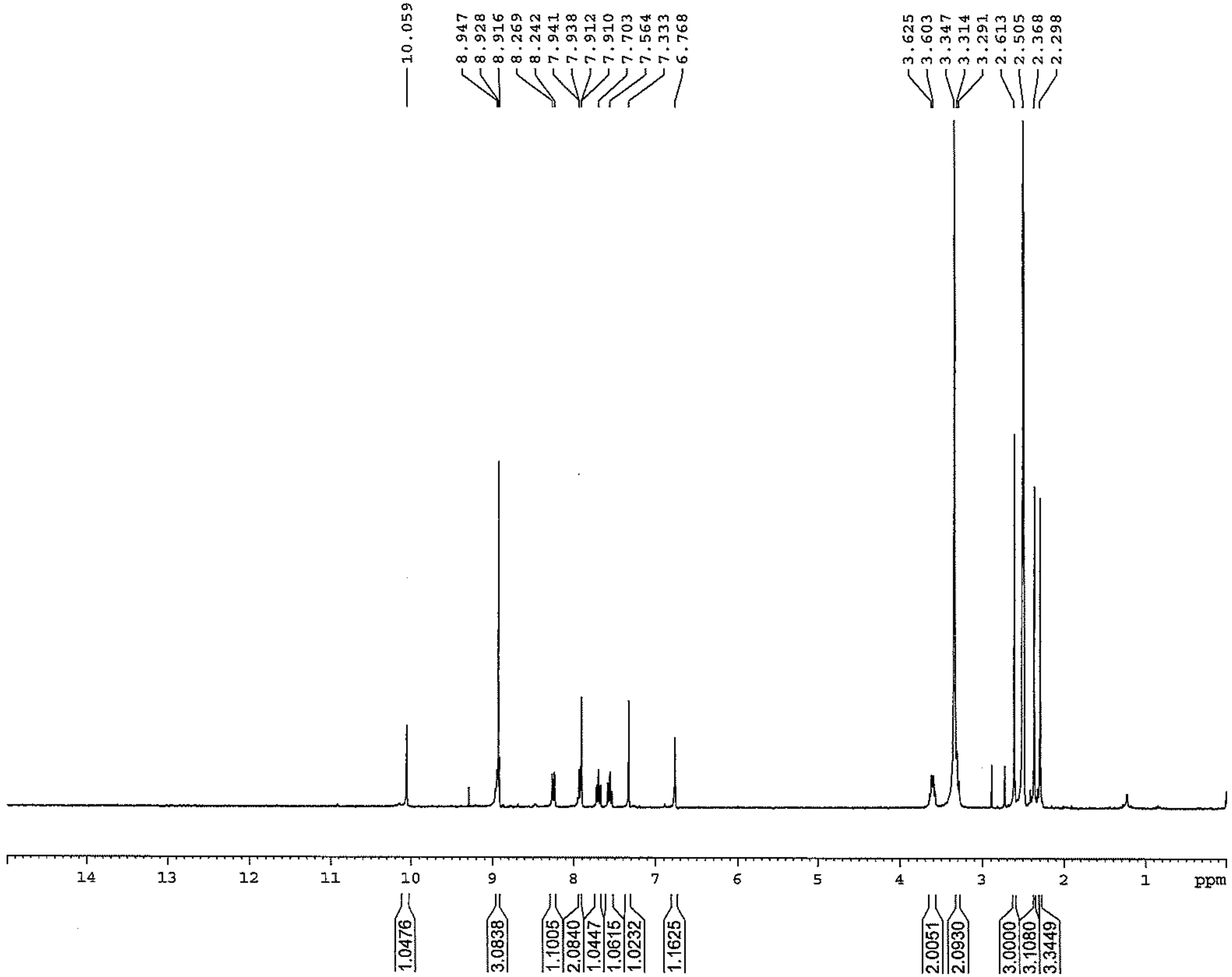

Name Tasdique Quadery
Date 06·23·22
NB # QUA-B-9-1

NAME	QUA-B-9-1	
EXPNO	10	
PROCNO	1	
Date_	20220623	
Time	11.33	
INSTRUM	spect	
PROBHD	5 mm QNP 1H/15	
PULPROG	zg30	
TD	65536	
SOLVENT	DMSO	
NS	16	
DS	2	
SWH	5995.204	Hz
FIDRES	0.091480	Hz
AQ	5.4657526	sec
RG	812.7	
DW	83.400	usec
DE	6.00	usec
TE	300.0	K
D1	1.00000000	sec
TD0	1	
======== CHANNEL f1 ========		
NUC1	1H	
P1	12.88	usec
PL1	1.00	dB
PL1W	9.77678490	W
SFO1	300.1319509	MHz
SI	32768	
SF	300.1299996	MHz
WDW	EM	
SSB	0	
LB	0.30	Hz
GB	0	
PC	1.00	

Sample Name:	QUA-B-9-1	Name: Tasdique Quodery
Date Acquired:	6/23/2022 12:01:27 PM EDT	Date: 06.23.22
Acq. Method Set:	C18_0_100mz150 1000 ESIpos_neg	Notebook: QUA-B-9-1

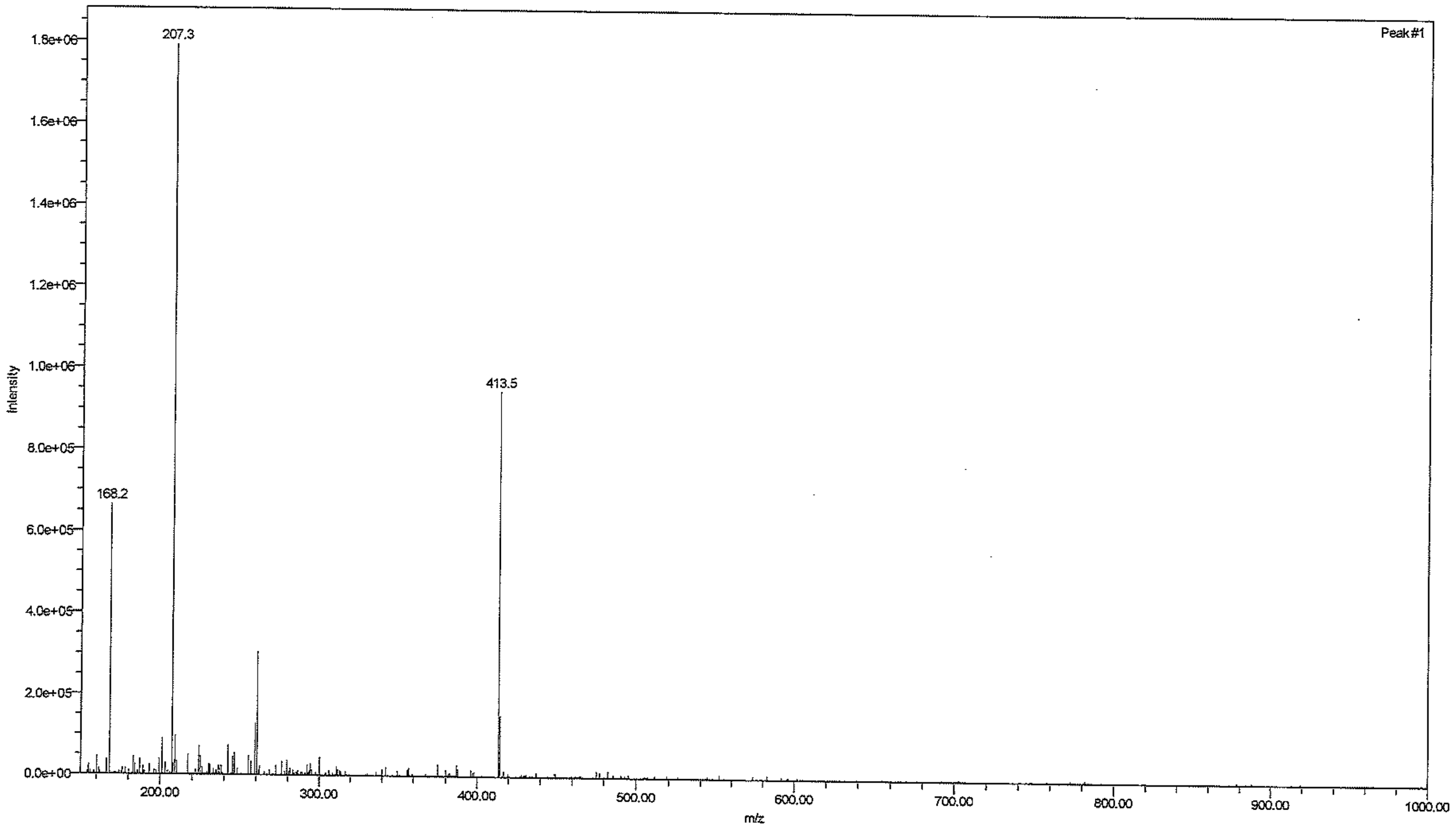

SAMPLE INFORMATION

Sample Name:	QUA-B-9-1
Injection Volume:	3.00 ul
Run Time:	9.0 Minutes
Date Acquired:	6/23/2022 12:17:15 PM EDT
Date Processed:	6/23/2022 12:29:17 PM EDT
Sample Set Name:	Template
Acq. Method Set:	BEH_C18_PDA_75mm 408
Processing Method:	BEH_C18_PDA
Channel Name:	254nm

Method Notes:
Acqulty UPLC BEH C18 1.7u (2.1x75mm)
Flow Rate : 0.5 mL/min
Solvent A : 0.1% TFA in Waters
Solvent B : 0.1% TFA in Acetonitrile
Solvent Gradient Program:

Time (min)	%A	% B
0:00	95	5
6:00	0	100
8:00	0	100
9:00	95	5

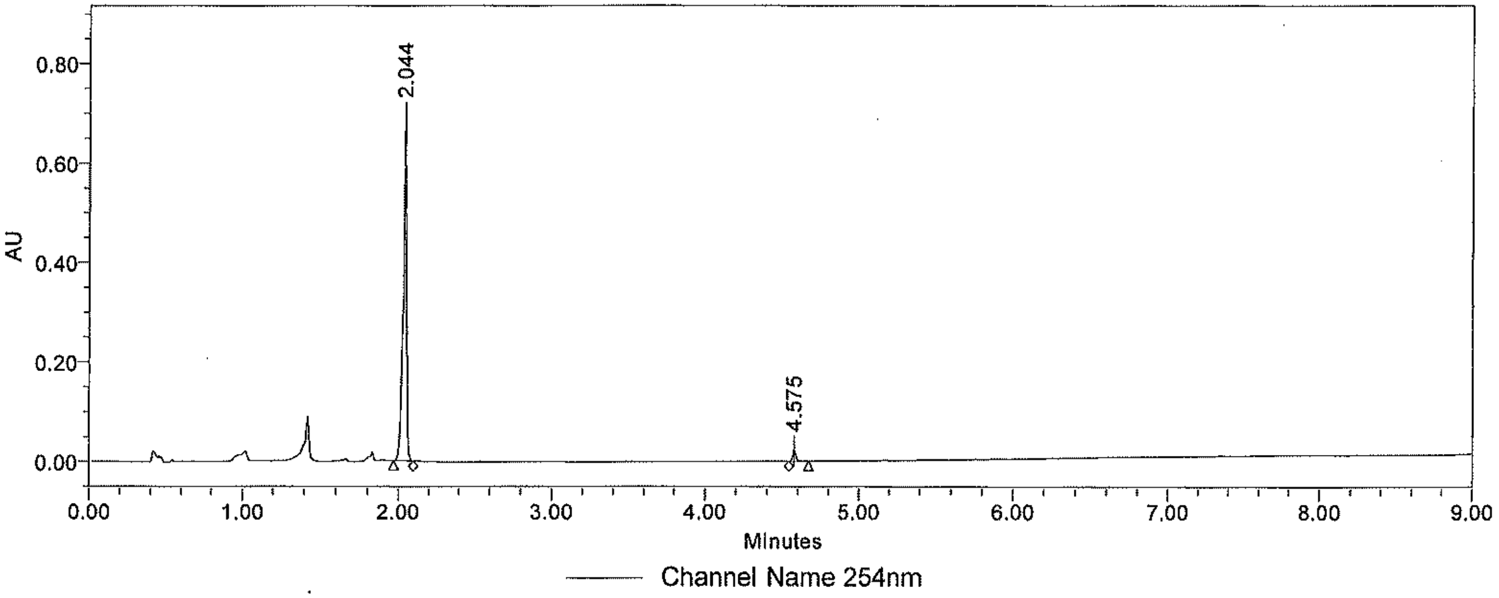

Peak Results

	RT	Area	Int Type	Width (sec)	% Area
1	2.044	1041343	BV	7.700	97.21
2	4.575	29878	VB	7.602	2.79

Name: Tasdique Quadery

Date: 06.23.22

NB #: QUA-B-9-1

CERTIFICATE OF ANALYSIS

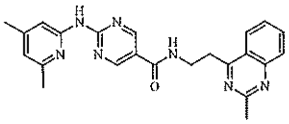

Compound Name: BPN-0035969-AA-001 1x
ALB Number: ALB-230410
Batch: 1
Lot Number: ARN-E-28-4
Molecular Formula: $C_{23}H_{23}N_7O$
Molecular Weight: 413.48
Last Solvent: Chloroform, Methylene Chloride, Methanol

TEST	RESULT/REFERENCE
Appearance	Light Yellow Solid
NMR Spectrum	^1H, 500 MHz, Dimethyl Sulfoxide-d_6, Consistent - Attached
Mass Spectrum	ESI, *m/z* 414 $[M + H]^+$, Attached
UPLC	96.0% (area %), ACQUITY UPLC BEH C18 (2.1 *75) mm, 1.7 micron Column, UV 254 nm Detection, Attached

[signature]
Approved By

7-13-2022
Date

For Research Purposes Only. Not Intended for Food or Drug Use.

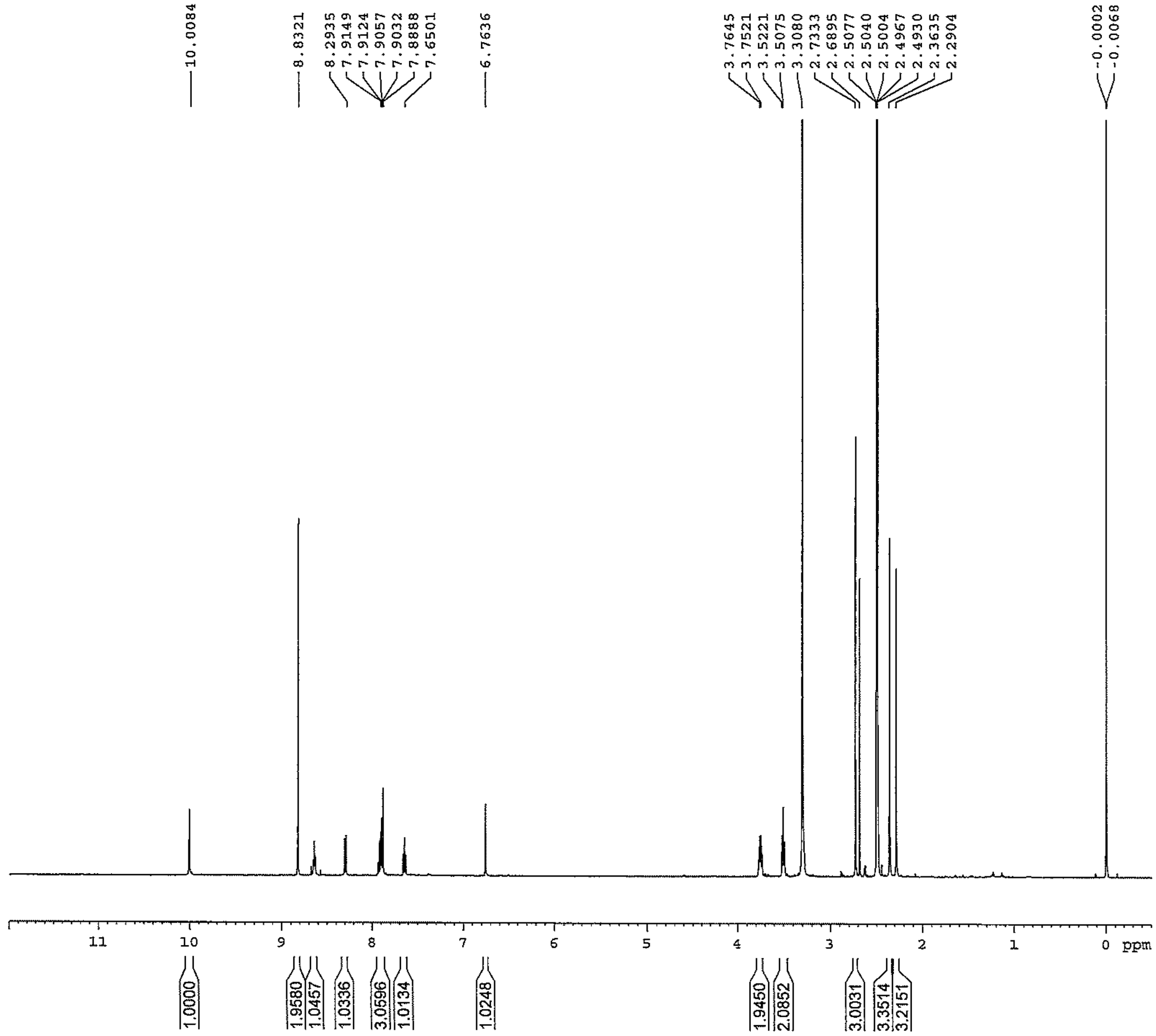

Name Corey Arnett-Butscher

Date 7/11/2022

NB # ARN-E-78-4

NAME	ARN-E-28-4
EXPNO	10
PROCNO	1
Date_	20220711
Time	9.52 h
INSTRUM	Avance Neo
PROBHD	Z167419_0029 (
PULPROG	zg30
TD	65536
SOLVENT	DMSO
NS	32
DS	2
SWH	10000.000 Hz
FIDRES	0.305176 Hz
AQ	3.2768500 sec
RG	101
DW	50.000 usec
DE	11.14 usec
TE	300.0 K
D1	1.00000000 sec
TD0	1
SFO1	500.1330883 MHz
NUC1	1H
P0	2.67 usec
P1	8.00 usec
SI	65536
SF	500.1300041 MHz
WDW	EM
SSB	0
LB	0.30 Hz
GB	0
PC	1.00

Openlynx Report Page 1

Vial:1:34

Date:11-Jul-2022

ID:

Time:13:30:09

File:ARN-E-28-4

Name: Corey Arnett-Butscher

Date: 7/11/2022

Notebook: ARN-E-28-4

Printed: Mon Jul 11 13:32:21 2022

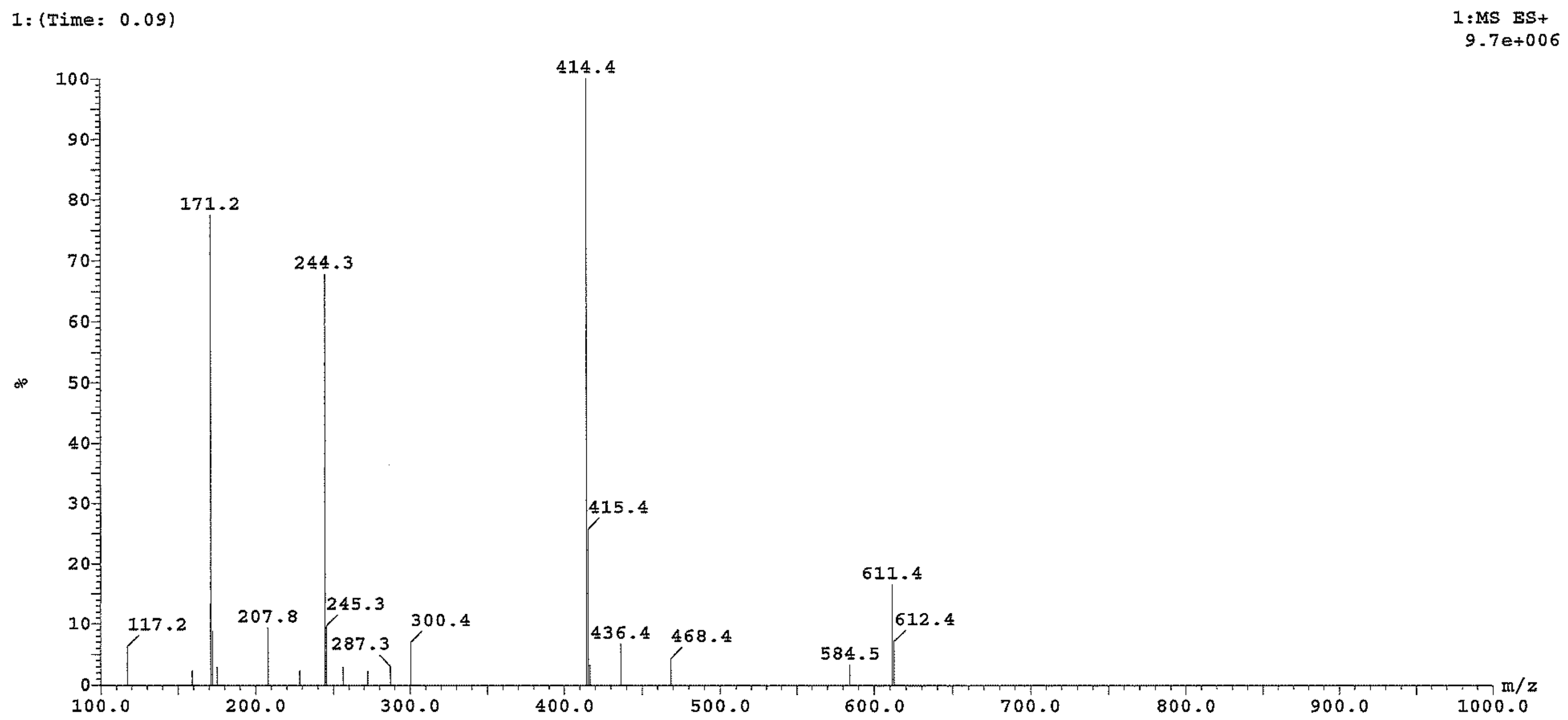

SAMPLE INFORMATION

Sample Name: ARN-E-28-4
Injection Volume: 3.00 ul
Run Time: 9.0 Minutes
Date Acquired: 7/11/2022 3:55:07 PM EDT
Date Processed: 7/11/2022 4:08:18 PM EDT
Sample Set Name: Template
Acq. Method Set: BEH_C18_PDA_75mm
Processing Method: BEH_C18_PDA_CAB
Channel Name: 254nm

Method Notes:
Acquity UPLC BEH C18 1.7u (2.1x75mm)
Flow Rate : 0.5 mL/min
Solvent A : 0.1% TFA in Waters
Solvent B : 0.1% TFA in Acetonitrile
Solvent Gradient Program:

Time (min)	%A	% B
0:00	95	5
6:00	0	100
8:00	0	100
9:00	95	5

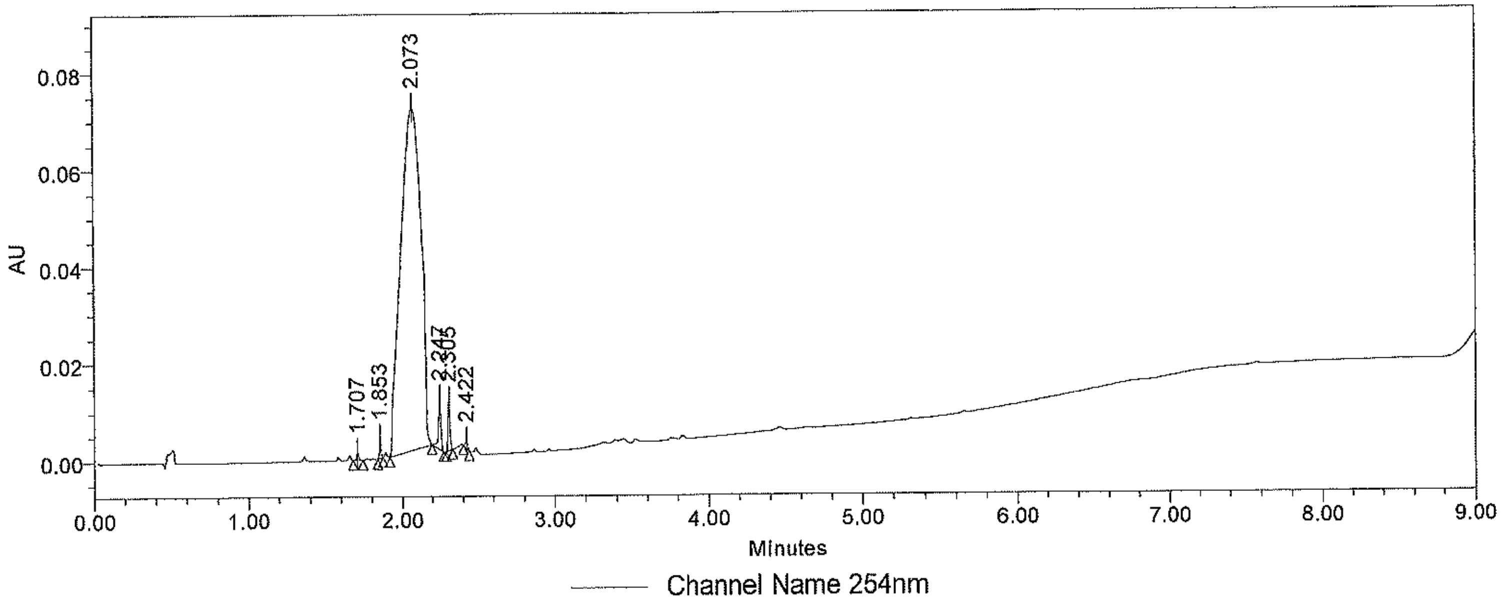

Peak Results

	RT	Area	Int Type	Width (sec)	% Area
1	1.707	1452	BB	3.750	0.21
2	1.853	3309	BB	2.150	0.49
3	2.073	651200	BB	16.699	96.06
4	2.247	11601	BB	4.550	1.71
5	2.305	8694	BB	2.650	1.28
6	2.422	1621	BB	2.400	0.24

Name: Cody Arnett-Betscher

Date: 7/11/2022

NB #: ARN-E-28-4

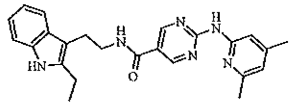

CERTIFICATE OF ANALYSIS

Compound Name: BPN-0036036-AA-001 1y
ALB Number: ALB-230690
Batch: 1
Lot Number: ARN-E-48-2
Molecular Formula: $C_{24}H_{26}N_6O$
Molecular Weight: 414.50
Last Solvent: Methylene Chloride

TEST	RESULT/REFERENCE
Appearance	Off-white Solid
NMR Spectrum	^1H, 500 MHz, Dimethyl Sulfoxide-d_6, Consistent - Attached
Mass Spectrum	ESI, *m/z* 415 $[M + H]^+$, Attached
UPLC	95.5% (area %), ACQUITY UPLC BEH C18 (2.1 *75) mm, 1.7 micron Column, UV 254 nm Detection, Attached

Approved By

8-3-2022
Date

For Research Purposes Only. Not Intended for Food or Drug Use

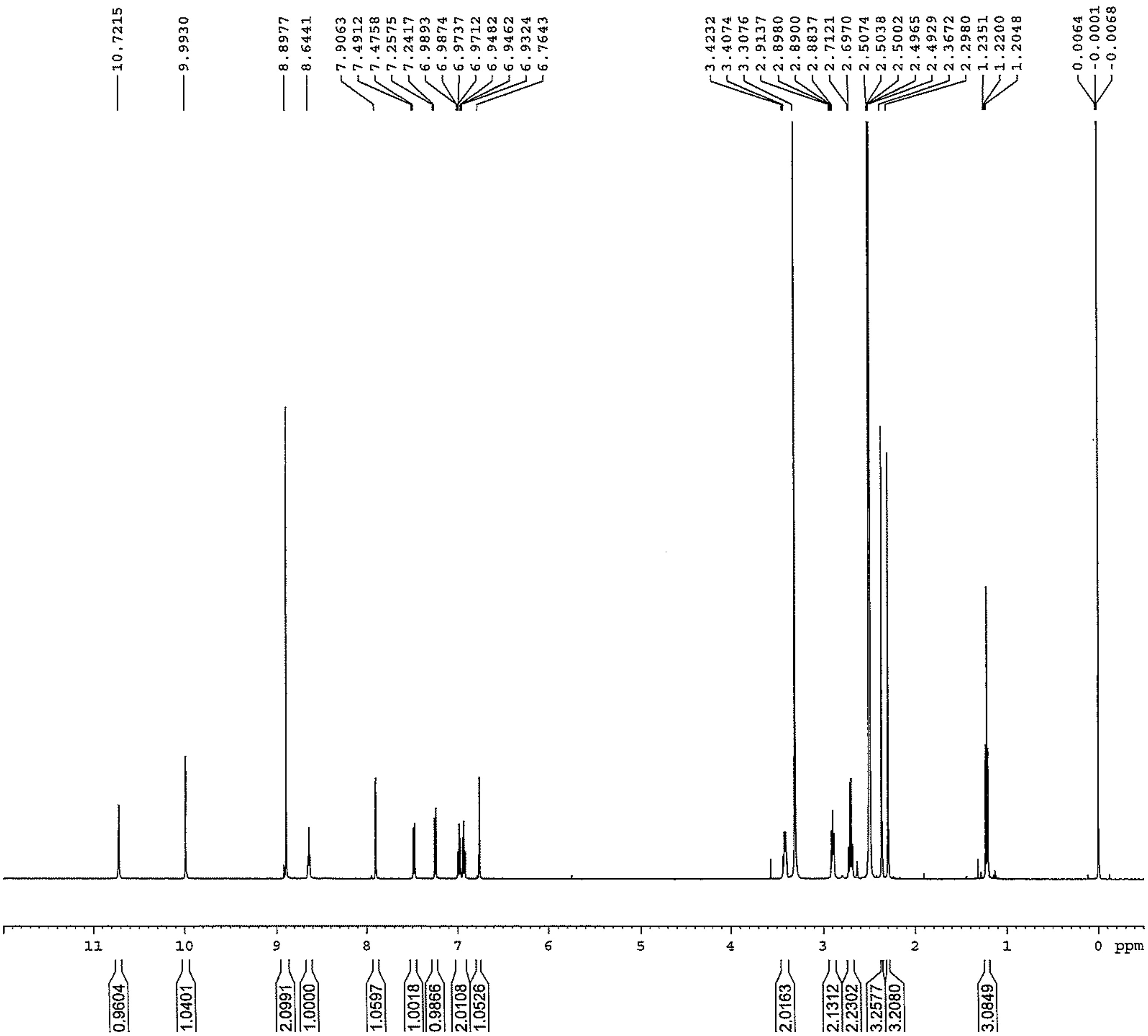

Name Corey Arnett-Butscher
Date 7/27/2022
NB # ARN-E-48-2

NAME	ARN-E-48-2
EXPNO	10
PROCNO	1
Date_	20220727
Time	13.20 h
INSTRUM	Avance Neo
PROBHD	Z167419_0029 (
PULPROG	zg30
TD	65536
SOLVENT	DMSO
NS	32
DS	2
SWH	10000.000 Hz
FIDRES	0.305176 Hz
AQ	3.2768500 sec
RG	101
DW	50.000 usec
DE	11.14 usec
TE	300.0 K
D1	1.00000000 sec
TD0	1
SFO1	500.1330883 MHz
NUC1	1H
P0	2.67 usec
P1	8.00 usec
SI	65536
SF	500.1300042 MHz
WDW	EM
SSB	0
LB	0.30 Hz
GB	0
PC	1.00

Openlynx Report
Vial:1:18
Date:27-Jul-2022

ID:
Time:10:09:37

File:ARN-E-48-2

Page 1

Name: Cory Arnett-Butscher

Date: 7/27/2022

Notebook: ARN-E-48-2

Printed: Wed Jul 27 10:11:33 2022

1: (Time: 0.09)

1:MS ES+
2.5e+007

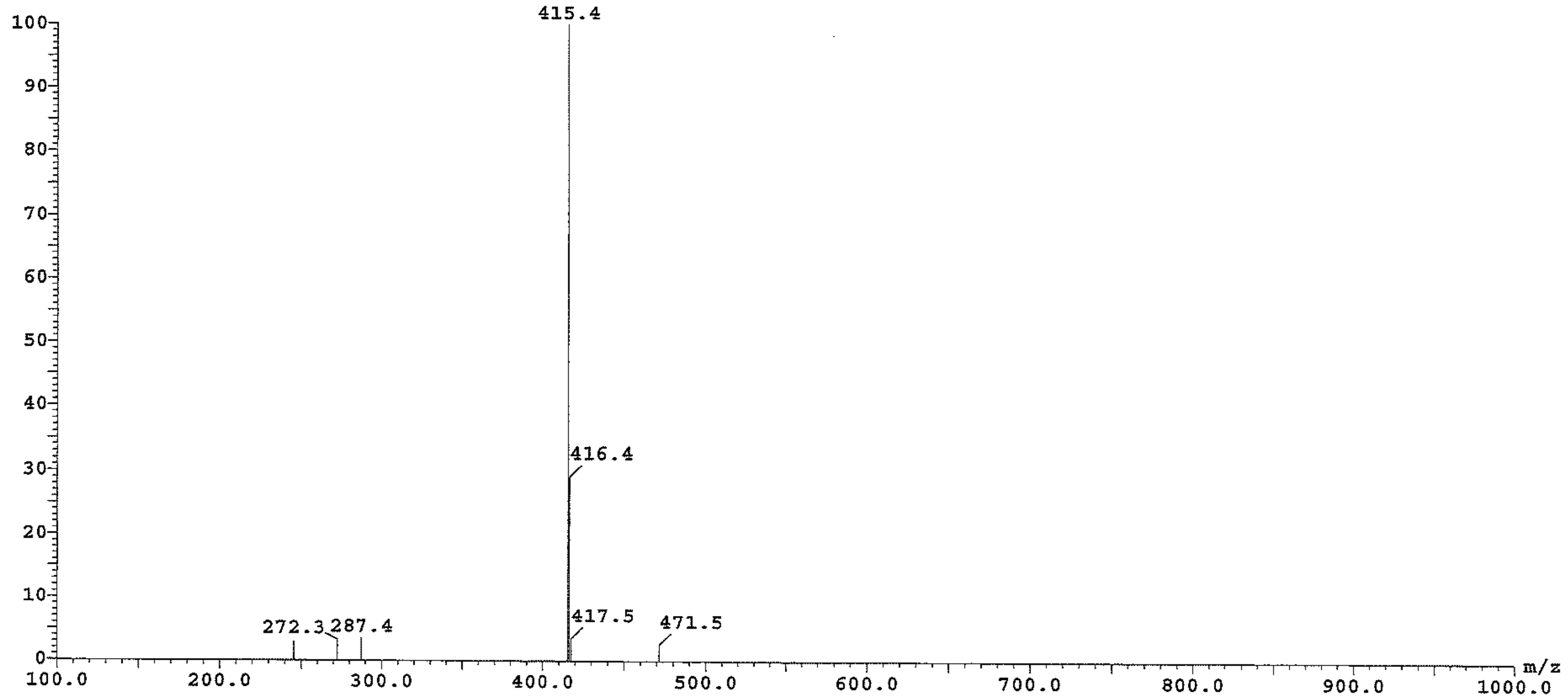

SAMPLE INFORMATION

Sample Name: ARN-E-48-2
Injection Volume: 3.00 ul
Run Time: 9.0 Minutes
Date Acquired: 7/27/2022 9:08:41 AM EDT
Date Processed: 7/27/2022 9:40:18 AM EDT
Sample Set Name: Template
Acq. Method Set: BEH_C18_PDA_75mm 408
Processing Method: BEH_C18_PDA_CAB
Channel Name: 254nm

Method Notes:
Acquity UPLC BEH C18 1.7u (2.1x75mm)
Flow Rate : 0.5 mL/min
Solvent A : 0.1% TFA in Waters
Solvent B : 0.1% TFA in Acetonitrile
Solvent Gradient Program:

Time (min)	%A	% B
0:00	95	5
6:00	0	100
8:00	0	100
9:00	95	5

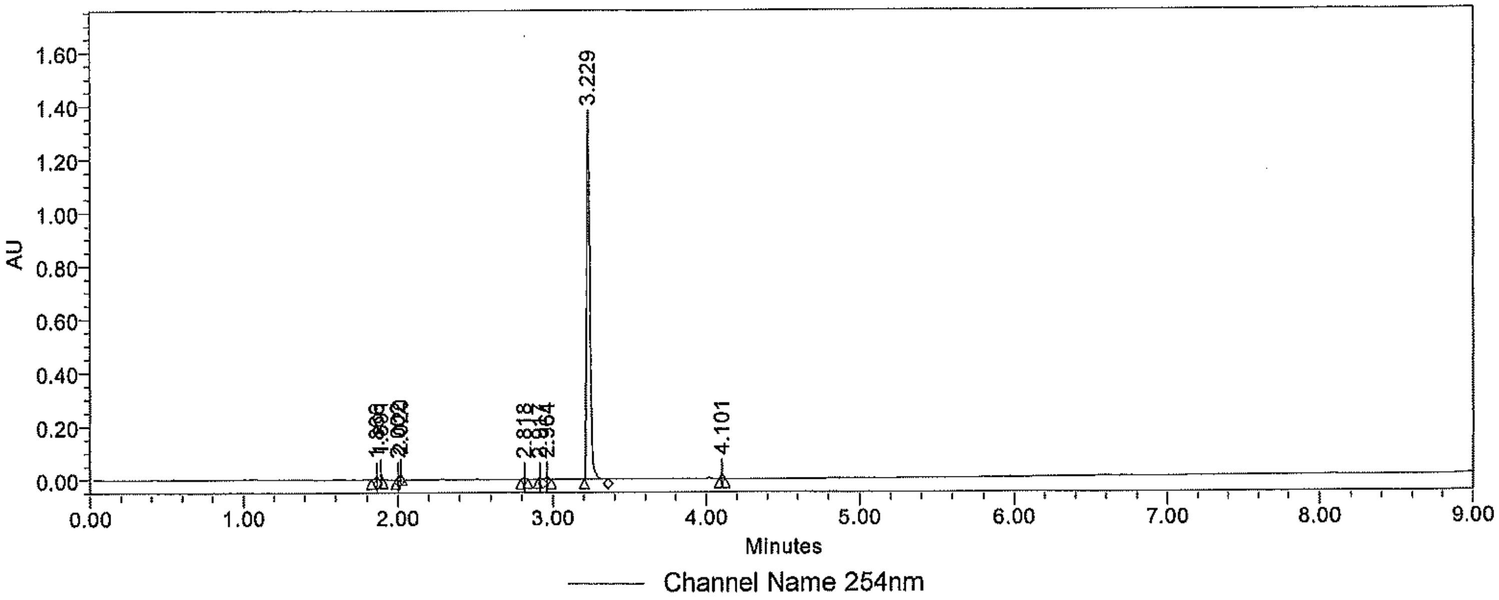

Peak Results

	RT	Area	Int Type	Width (sec)	% Area
1	1.866	8697	bV	2.650	0.42
2	1.891	20086	Vb	2.000	0.98
3	2.002	4289	bV	1.300	0.21
4	2.020	7377	Vb	1.100	0.36
5	2.818	9137	bb	2.700	0.45
6	2.917	6186	bV	2.050	0.30
7	2.964	14275	Vb	3.100	0.70
8	3.229	1959767	bV	9.199	95.53
9	4.101	21664	bb	2.401	1.06

Name: Cory Arnett-Butscher

Date: 7/27/2022

NB #: ARN-E-48-2

CERTIFICATE OF ANALYSIS

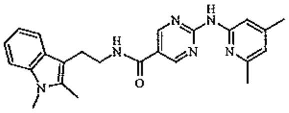

Compound Name: BPN-0036038-AA-001 1z
ALB Number: ALB-230688
Batch: 1
Lot Number: ALK-C-28-1
Molecular Formula: $C_{24}H_{26}N_6O$
Molecular Weight: 414.50
Last Solvent: Methylene Chloride, Methanol

**TEST**	**RESULT/REFERENCE**
Appearance	Off-white Solid
NMR Spectrum	^{1}H, 500 MHz, Dimethyl Sulfoxide-d_6, Consistent - Attached
Mass Spectrum	ESI, *m/z* 415 $[M + H]^+$, Attached
UPLC	>99% (area %), ACQUITY UPLC BEH C18 (2.1 *75) mm, 1.7 micron Column, UV 254 nm Detection, Attached

Approved By: [signature]

Date: 8-3-2022

For Research Purposes Only. Not Intended for Food or Drug Use.

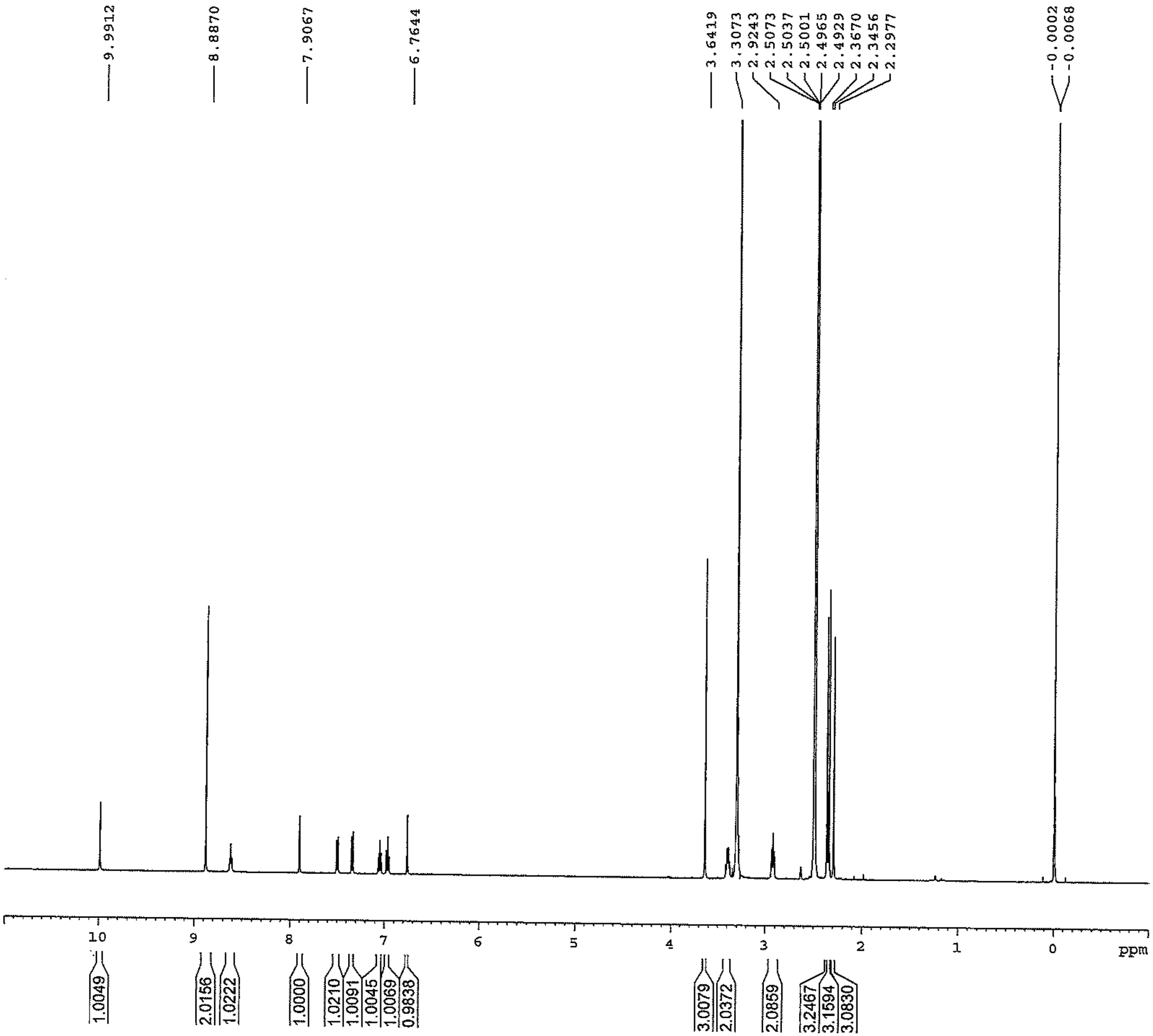

Name Marwah Albaker
Date 28-Jul-2022
NB # ALK-C-28-1

NAME	ALK-C-28-1
EXPNO	10
PROCNO	1
Date_	20220729
Time	11.33 h
INSTRUM	Avance Neo
PROBHD	Z167419_0029 (
PULPROG	zg30
TD	65536
SOLVENT	DMSO
NS	64
DS	2
SWH	10000.000 Hz
FIDRES	0.305176 Hz
AQ	3.2768500 sec
RG	101
DW	50.000 usec
DE	11.14 usec
TE	300.0 K
D1	1.00000000 sec
TD0	1
SFO1	500.1330883 MHz
NUC1	1H
P0	2.67 usec
P1	8.00 usec
SI	65536
SF	500.1300043 MHz
WDW	EM
SSB	0
LB	0.30 Hz
GB	0
PC	1.00

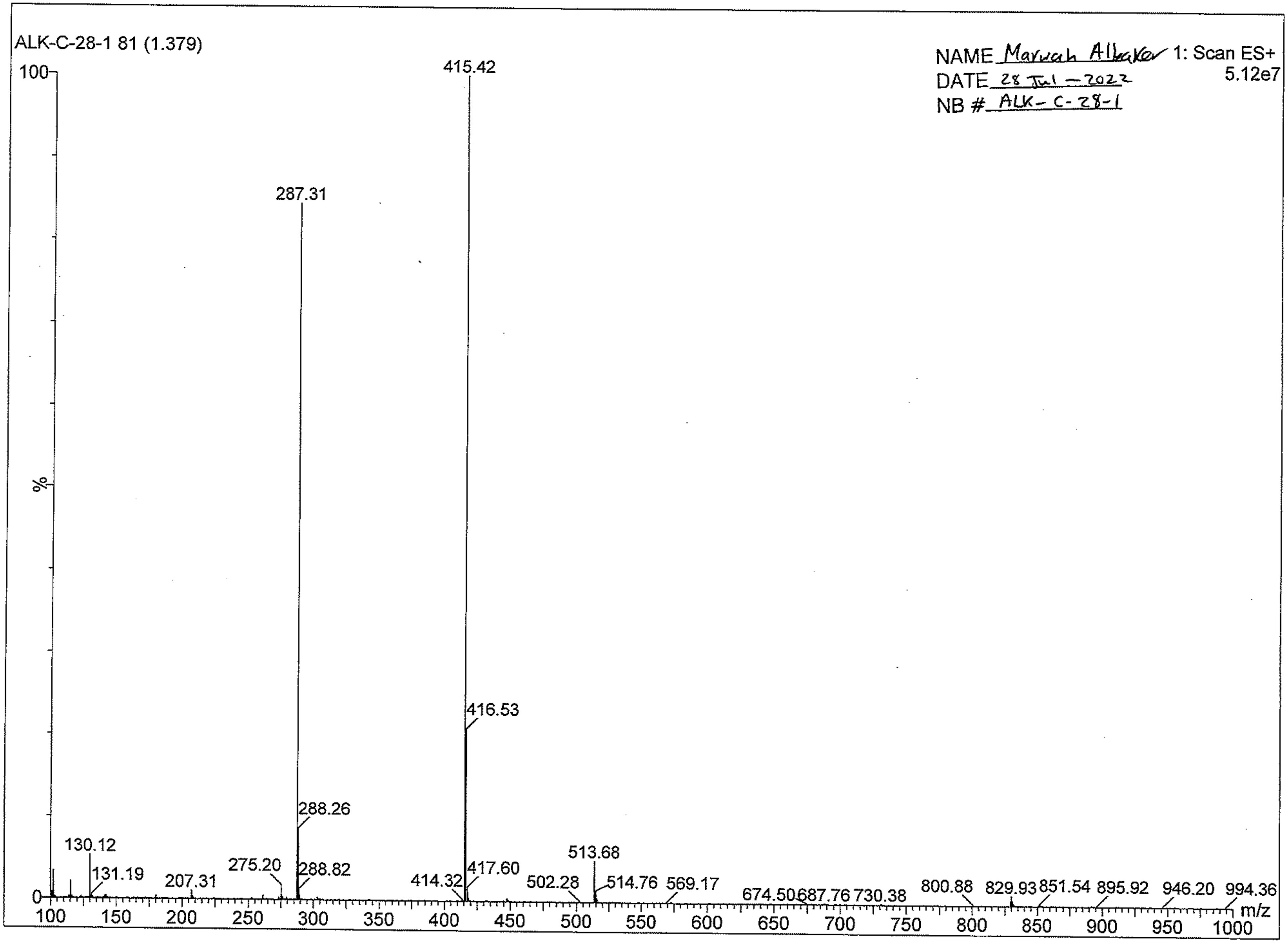

ALK-C-28-1 81 (1.379)
NAME Marwah Albaker
DATE 28 Jul - 2022
NB # ALK-C-28-1
1: Scan ES+
5.12e7
100
%
0
415.42
287.31
416.53
288.26
130.12
131.19
207.31
275.20
288.82
414.32
417.60
502.28
513.68
514.76
569.17
674.50
687.76
730.38
800.88
829.93
851.54
895.92
946.20
994.36
100
150
200
250
300
350
400
450
500
550
600
650
700
750
800
850
900
950
1000
m/z

SAMPLE INFORMATION

Sample Name:	ALK-C-28-1
Injection Volume:	2.00 ul
Run Time:	9.0 Minutes
Date Acquired:	7/28/2022 2:25:52 PM EDT
Date Processed:	7/28/2022 2:37:53 PM EDT
Sample Set Name:	Template
Acq. Method Set:	BEH_C18_PDA_75mm 408
Processing Method:	BEH_C18_PDA
Channel Name:	254nm

Method Notes:
Acquity UPLC BEH C18 1.7u (2.1x75mm)
Flow Rate : 0.5 mL/min
Solvent A : 0.1% TFA in Waters
Solvent B : 0.1% TFA in Acetonitrile
Solvent Gradient Program:

Time (min)	%A	% B
0:00	95	5
6:00	0	100
8:00	0	100
9:00	95	5

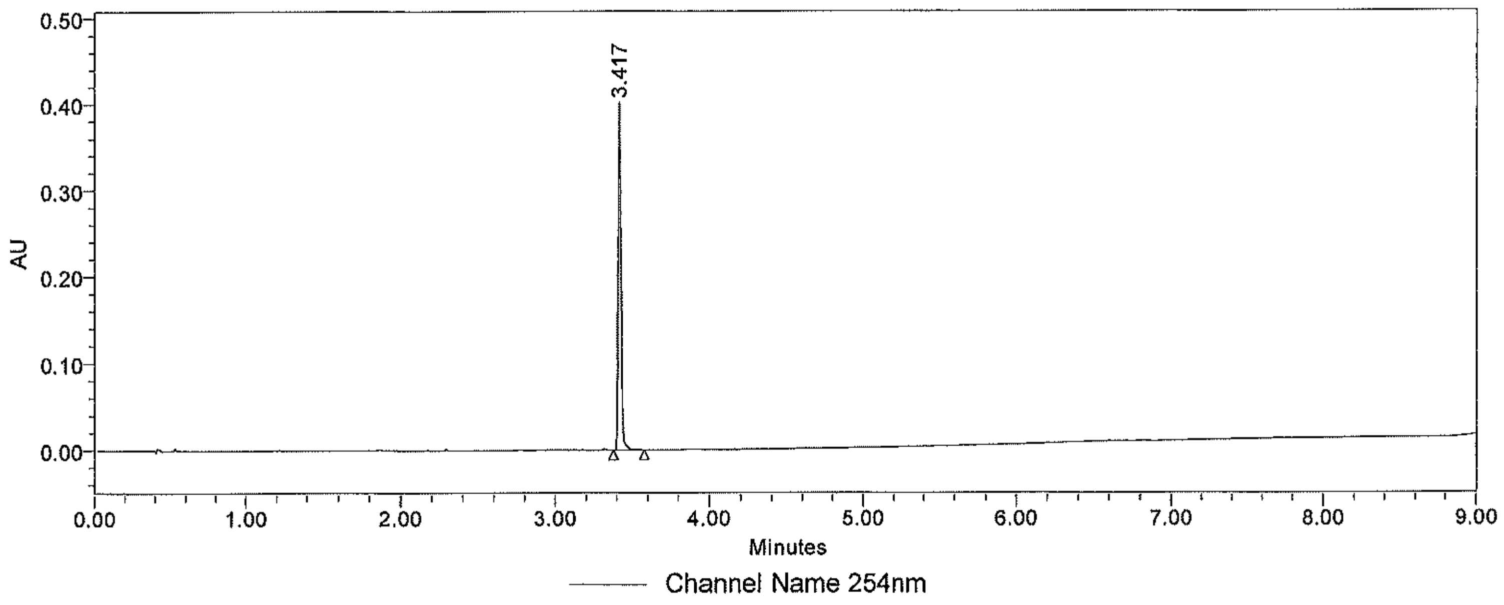

Peak Results

	RT	Area	Int Type	Width (sec)	% Area
1	3.417	510619	BB	11.949	100.00

Name: Marwah Albaker

Date: 28-Jul-2022

NB #: ALK-C-28-1

CERTIFICATE OF ANALYSIS

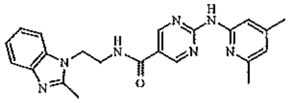

Compound Name: BPN-0036047-AA-001 1aa
ALB Number: ALB-230777
Batch: 1
Lot Number: ALK-C-32-1
Molecular Formula: $C_{22}H_{23}N_7O$
Molecular Weight: 401.46
Last Solvent: Ethyl Acetate

TEST	RESULT/REFERENCE
Appearance	Off-white Solid
NMR Spectrum	^{1}H, 500 MHz, Dimethyl Sulfoxide-d_6, Consistent - Attached
Mass Spectrum	ESI, *m/z* 402 [M + H]$^+$, Attached
UPLC	98.4% (area %), ACQUITY UPLC BEH C18 (2.1 *75) mm, 1.7 micron Column, UV 254 nm Detection, Attached

[signature]
Approved By

8-10-2022
Date

For Research Purposes Only. Not Intended for Food or Drug Use.

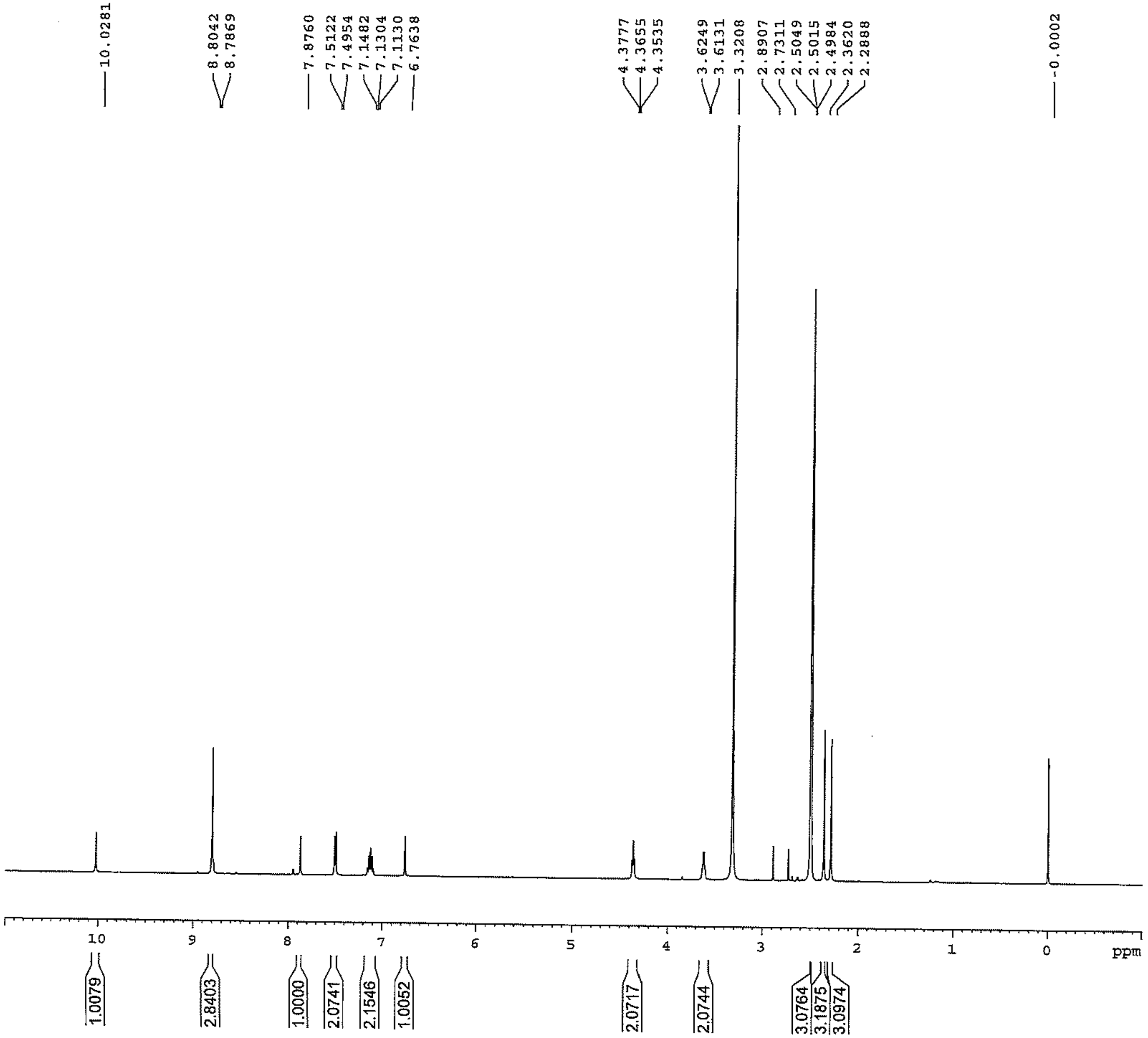

Name Marwah Albaker
Date 8-Aug-2022
NB # ALK C-32-1

NAME	ALK-C-32-1
EXPNO	10
PROCNO	1
Date_	20220808
Time	8.53 h
INSTRUM	Avance Neo
PROBHD	Z167419_0029 (
PULPROG	zg30
TD	65536
SOLVENT	DMSO
NS	32
DS	2
SWH	10000.000 Hz
FIDRES	0.305176 Hz
AQ	3.2768500 sec
RG	101
DW	50.000 usec
DE	11.14 usec
TE	300.0 K
D1	1.00000000 sec
TD0	1
SFO1	500.1330883 MHz
NUC1	1H
P0	2.67 usec
P1	8.00 usec
SI	65536
SF	500.1300036 MHz
WDW	EM
SSB	0
LB	0.30 Hz
GB	0
PC	1.00

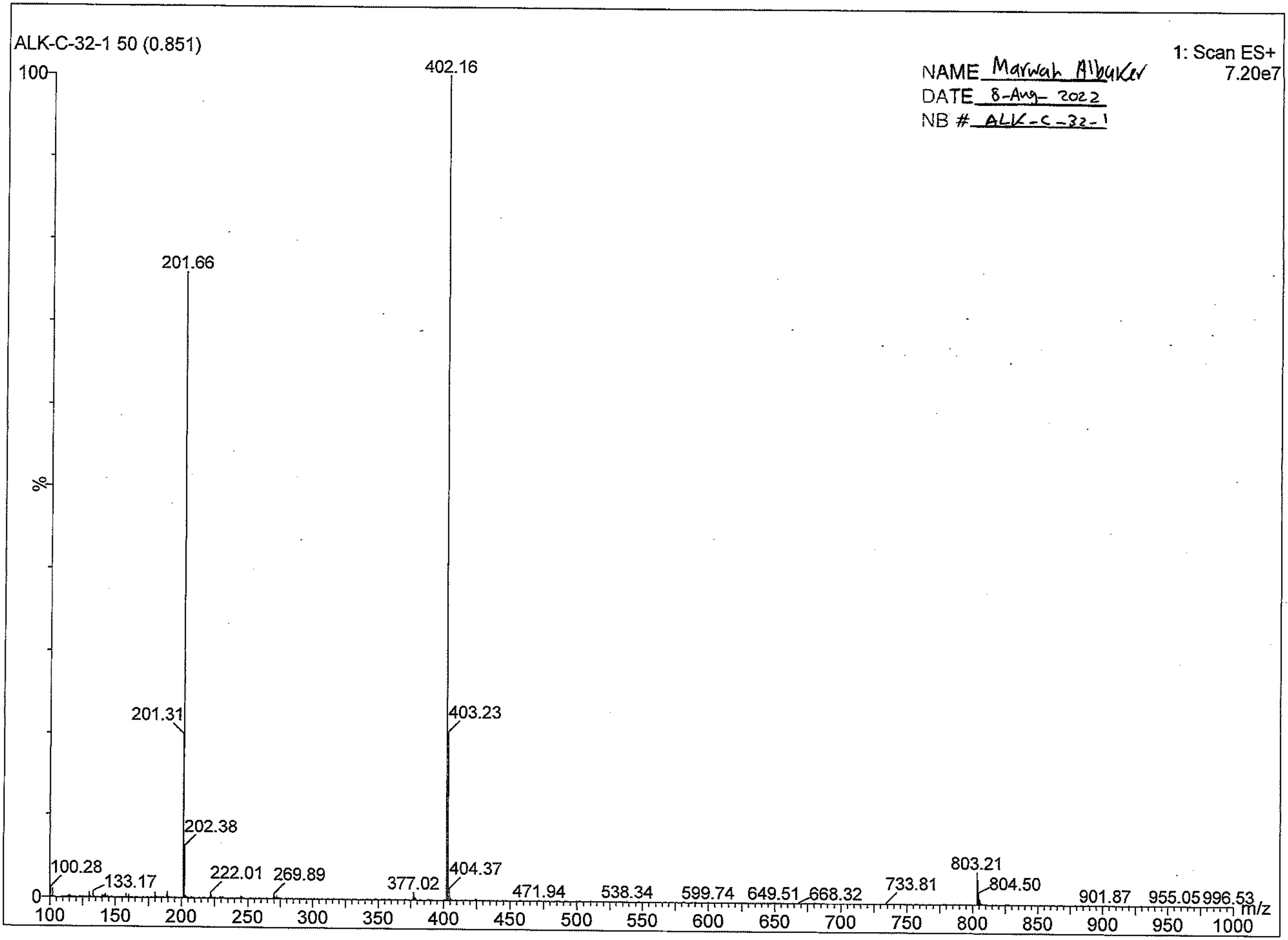
ALK-C-32-1 50 (0.851)
1: Scan ES+
7.20e7
NAME Marwah Albuker
DATE 8-Aug- 2022
NB # ALK-C-32-1
100
%
0
402.16
201.66
201.31
403.23
202.38
100.28
133.17
222.01
269.89
377.02
404.37
471.94
538.34
599.74
649.51
668.32
733.81
803.21
804.50
901.87
955.05
996.53
100
150
200
250
300
350
400
450
500
550
600
650
700
750
800
850
900
950
1000
m/z

SAMPLE INFORMATION

Sample Name:	ALK-C-32-1
Injection Volume:	2.00 ul
Run Time:	9.0 Minutes
Date Acquired:	8/5/2022 2:32:25 PM EDT
Date Processed:	8/5/2022 2:57:38 PM EDT
Sample Set Name:	Template
Acq. Method Set:	BEH_C18_PDA_75mm 408
Processing Method:	BEH_C18_PDA
Channel Name:	254nm

Method Notes:
Acquity UPLC BEH C18 1.7u (2.1x75mm)
Flow Rate : 0.5 mL/min
Solvent A : 0.1% TFA in Waters
Solvent B : 0.1% TFA in Acetonitrile
Solvent Gradient Program:

Time (min)	%A	% B
0:00	95	5
6:00	0	100
8:00	0	100
9:00	95	5

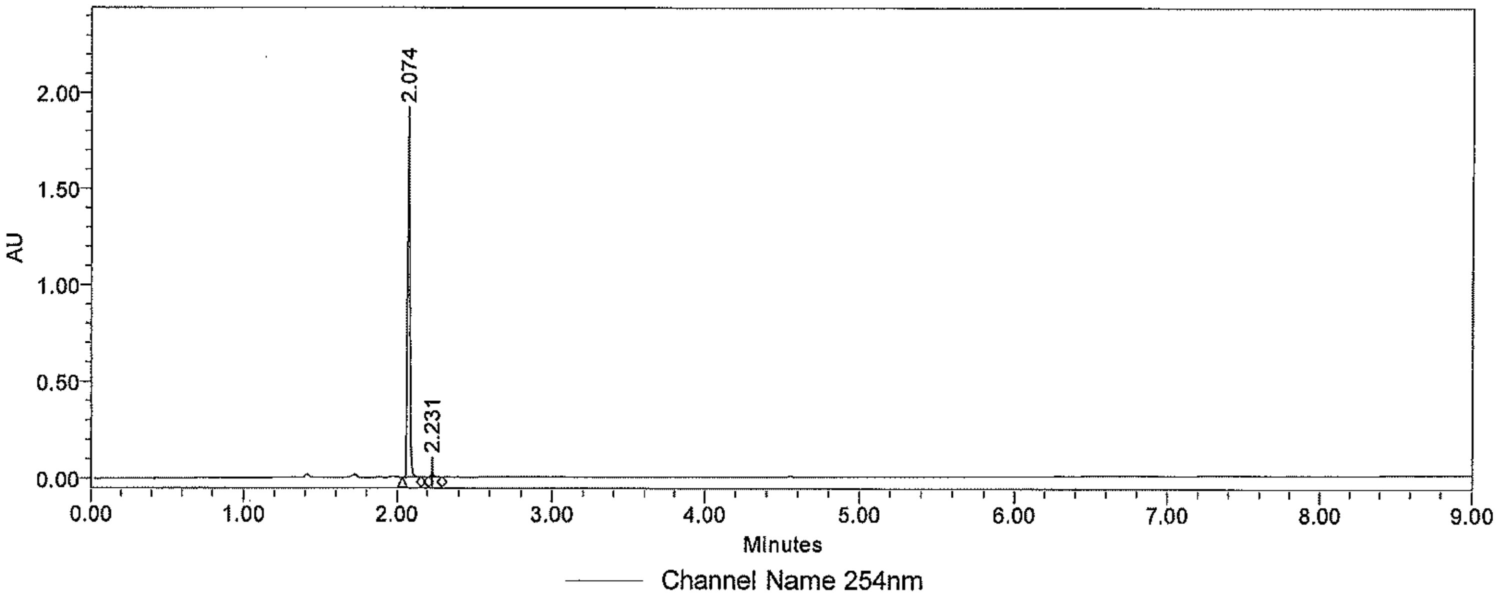

Peak Results

	RT	Area	Int Type	Width (sec)	% Area
1	2.074	2020654	BV	7.349	98.48
2	2.231	31171	VV	5.200	1.52

Name: Marwah Albakker

Date: 5-Aug-2022

NB #: ALK-C-32-1

CERTIFICATE OF ANALYSIS

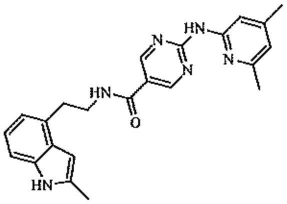

Compound Name: BPN-0036190-AA-001 1bb
ALB Number: ALB-231323
Batch: 1
Lot Number: QUA-B-55-1
Molecular Formula: $C_{23}H_{24}N_6O$
Molecular Weight: 400.48
Last Solvent: Water

TEST	RESULT/REFERENCE
Appearance	Light Brown Solid
NMR Spectrum	^{1}H, 500 MHz, Dimethyl Sulfoxide-d_6, Consistent - Attached
Mass Spectrum	ESI, *m/z* 401 [M + H]$^+$, Attached
UPLC	95.3% (area %), ACQUITY UPLC BEH C18 (2.1 *75) mm, 1.7 micron Column, UV 254 nm Detection, Attached

Approved By: [signature]

Date: 9-21-2022

For Research Purposes Only. Not Intended for Food or Drug Use.

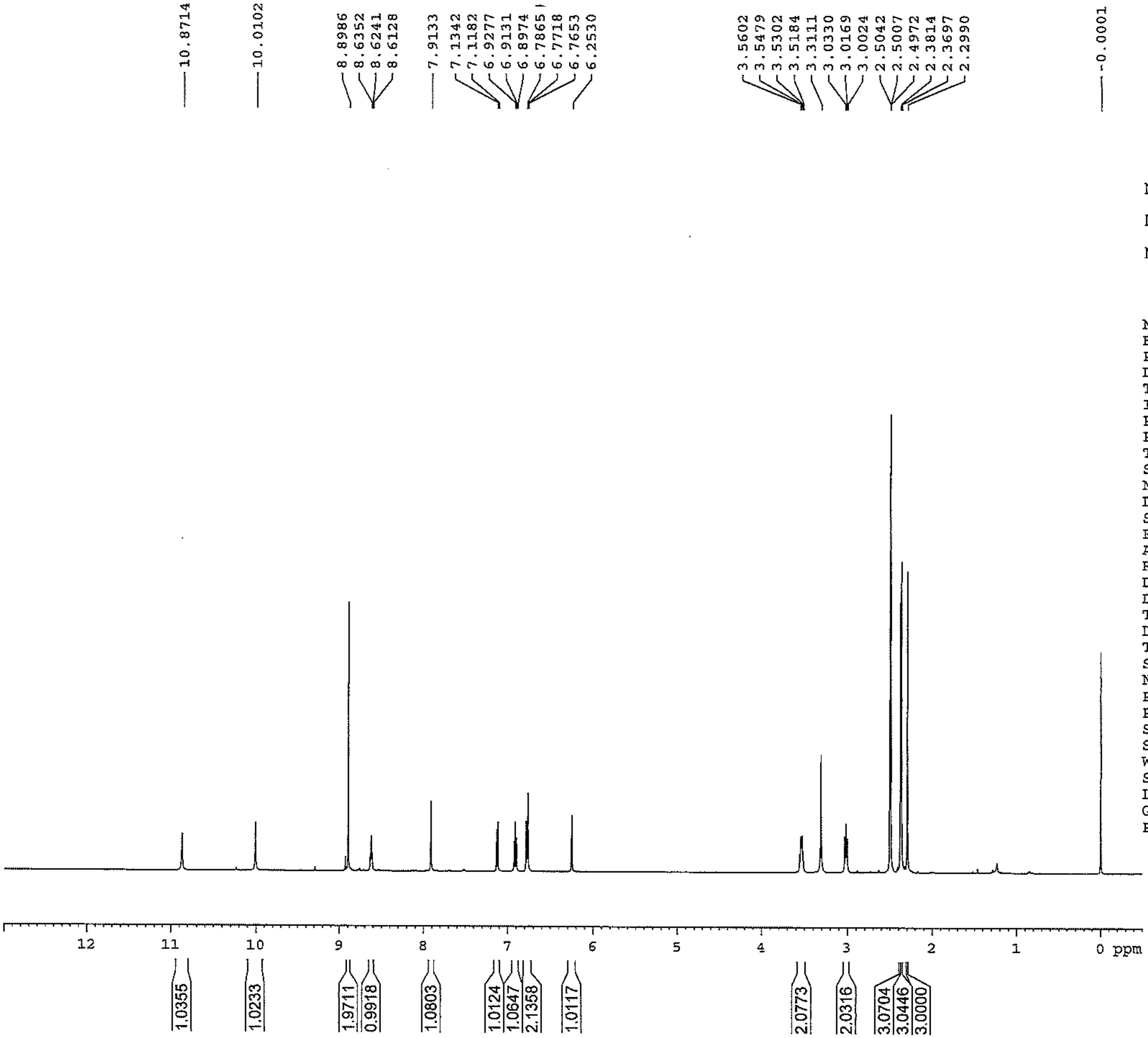

Name Tasdique Quadery

Date 09.09.22

NB # QUA-B-55-1

NAME	QUA-B-55-1
EXPNO	10
PROCNO	1
Date_	20220909
Time	9.59 h
INSTRUM	Avance Neo
PROBHD	Z167419_0029 (
PULPROG	zg30
TD	65536
SOLVENT	DMSO
NS	16
DS	2
SWH	10000.000 Hz
FIDRES	0.305176 Hz
AQ	3.2768500 sec
RG	101
DW	50.000 usec
DE	11.14 usec
TE	300.0 K
D1	1.00000000 sec
TD0	1
SFO1	500.1330883 MHz
NUC1	1H
P0	2.67 usec
P1	8.00 usec
SI	65536
SF	500.1300039 MHz
WDW	EM
SSB	0
LB	0.30 Hz
GB	0
PC	1.00

Openlynx Report Page 1

Vial:1:11
Date:09-Sep-2022

ID:
Time:09:42:09

File:QUA-B-55-1

Name: Tasdique Quadery Date: 09.09.22 Notebook: QUA-B-55-1

Printed: Fri Sep 09 09:43:58 2022

1:(Time: 0.09) Combine (2:5)

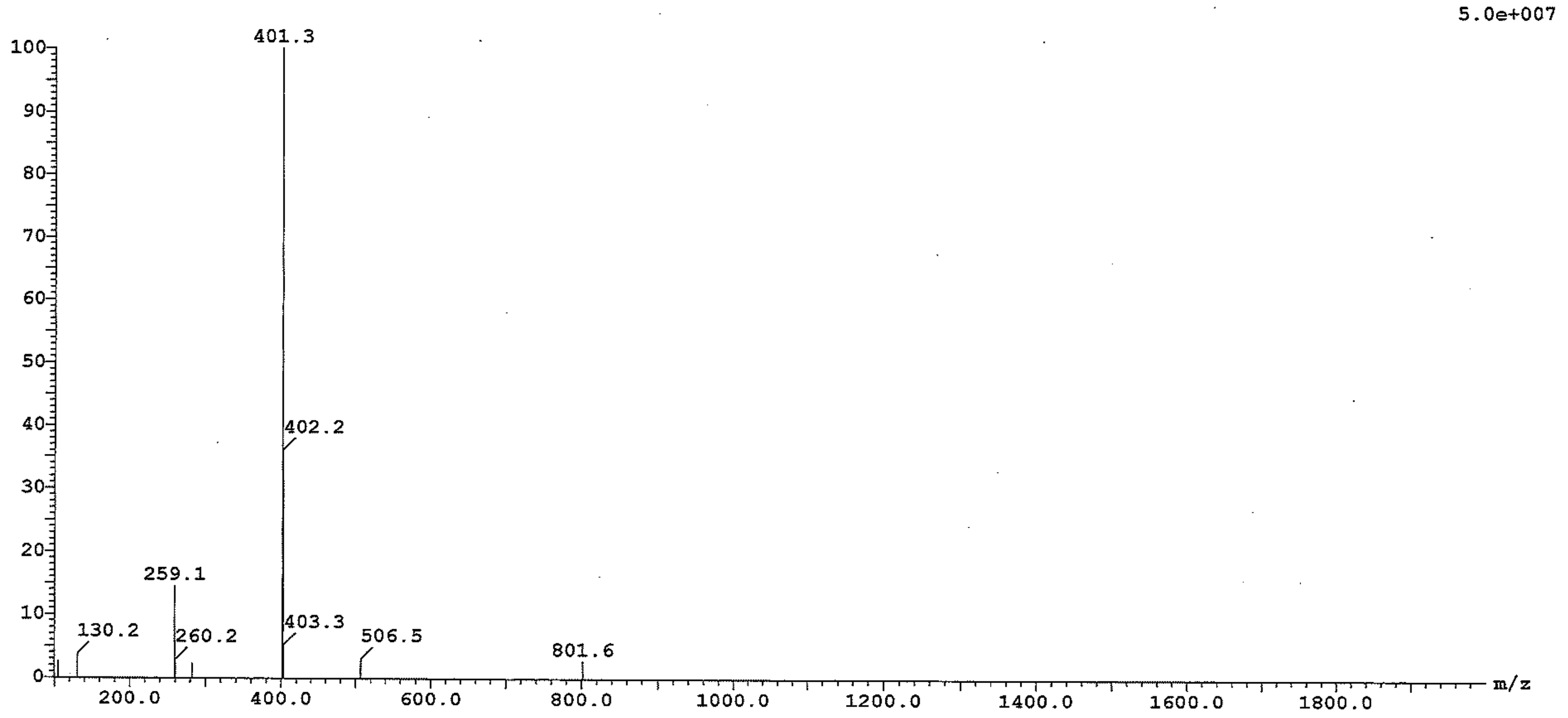

SAMPLE INFORMATION

Sample Name: QUA-B-55-1
Injection Volume: 3.00 ul
Run Time: 9.0 Minutes
Date Acquired: 9/9/2022 9:46:19 AM EDT
Date Processed: 9/9/2022 9:58:20 AM EDT
Sample Set Name: Template
Acq. Method Set: BEH_C18_PDA_75mm 408
Processing Method: BEH_C18_PDA
Channel Name: 254nm

Method Notes:
Acquity UPLC BEH C18 1.7u (2.1x75mm)
Flow Rate : 0.5 mL/min
Solvent A : 0.1% TFA in Waters
Solvent B : 0.1% TFA in Acetonitrile
Solvent Gradient Program:

Time (min)	%A	% B
0:00	95	5
6:00	0	100
8:00	0	100
9:00	95	5

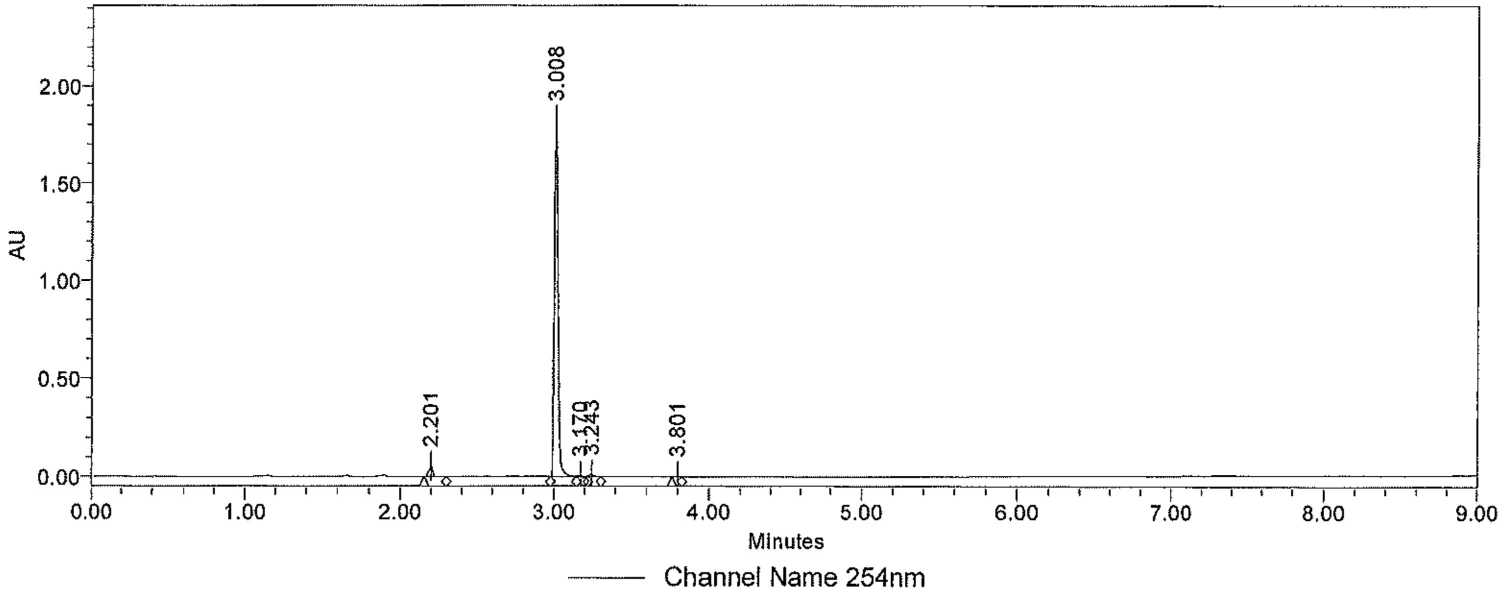

Peak Results

	RT	Area	Int Type	Width (sec)	% Area
1	2.201	99323	BV	8.849	3.14
2	3.008	3014112	VV	10.099	95.39
3	3.170	12869	VV	3.150	0.41
4	3.243	26733	VV	4.950	0.85
5	3.801	6632	BV	3.750	0.21

Name: Tasdique Quadery

Date: 09.09.22

NB #: QUA-B-55-1

CERTIFICATE OF ANALYSIS

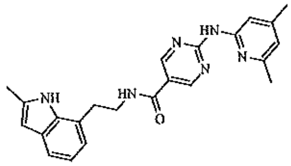

Compound Name: BPN-0036194-AA-001 1cc
ALB Number: ALB-231329
Batch: 1
Lot Number: QUA-B-59-3
Molecular Formula: $C_{23}H_{24}N_6O$
Molecular Weight: 400.48
Last Solvent: Methylene Chloride

TEST	RESULT/REFERENCE
Appearance	Pink Solid
NMR Spectrum	^{1}H, 500 MHz, Dimethyl Sulfoxide-d_6, Consistent - Attached
Mass Spectrum	ESI, *m/z* 399 [M – H]$^-$, Attached
UPLC	95.4% (area %), ACQUITY UPLC BEH C18 (2.1 *75) mm, 1.7 micron Column, UV 254 nm Detection, Attached

Approved By: [signature]

Date: 9-21-2022

For Research Purposes Only. Not Intended for Food or Drug Use.

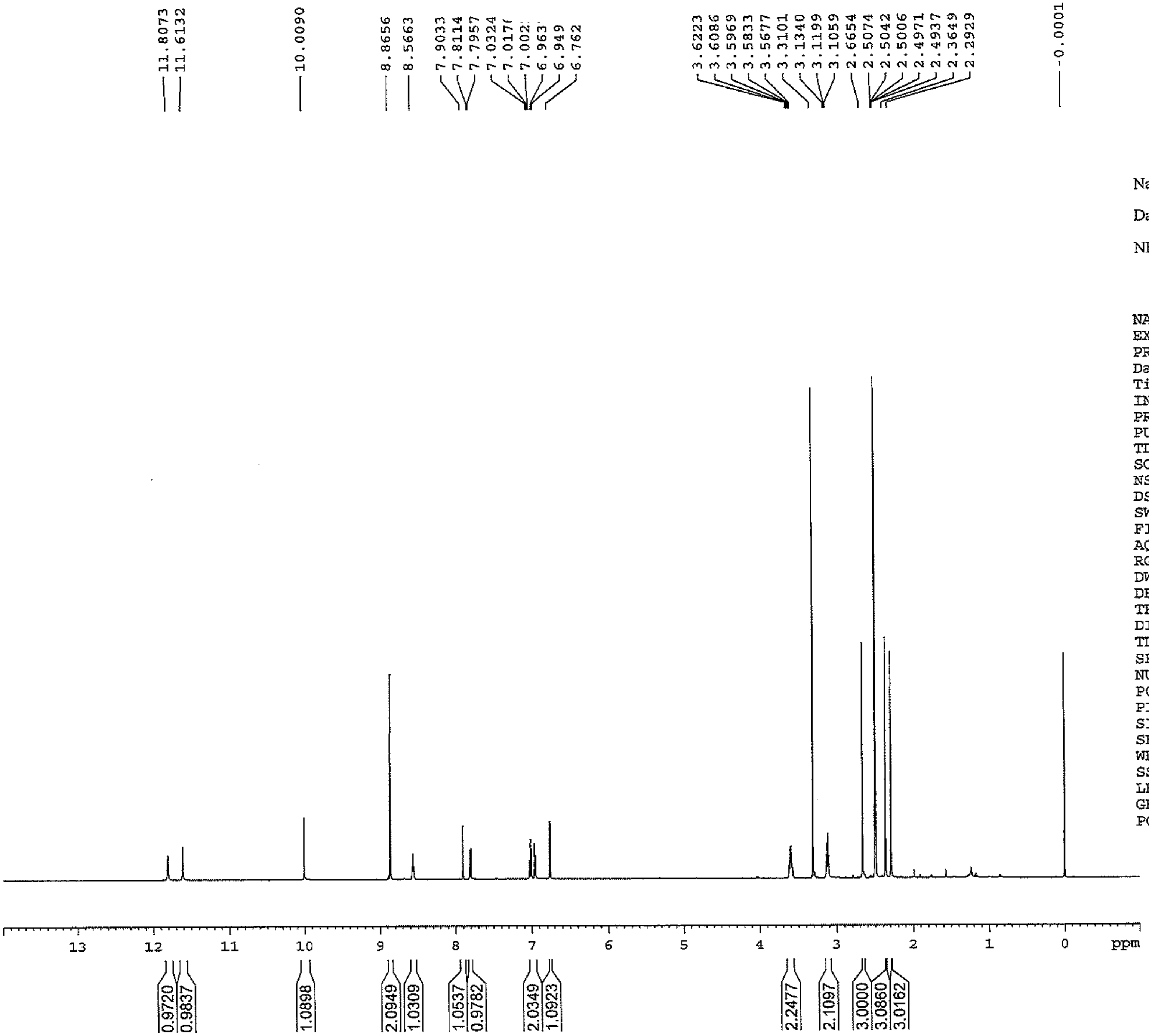

Name Tasdique Quadery

Date 09.19.22

NB # QUA-B-59-3

NAME	QUA-B-59-3
EXPNO	10
PROCNO	1
Date_	20220919
Time	16.06 h
INSTRUM	Avance Neo
PROBHD	Z167419_0029 (
PULPROG	zg30
TD	65536
SOLVENT	DMSO
NS	16
DS	2
SWH	10000.000 Hz
FIDRES	0.305176 Hz
AQ	3.2768500 sec
RG	101
DW	50.000 usec
DE	11.14 usec
TE	300.0 K
D1	1.00000000 sec
TD0	1
SFO1	500.1330883 MHz
NUC1	1H
P0	2.67 usec
P1	8.00 usec
SI	65536
SF	500.1300039 MHz
WDW	EM
SSB	0
LB	0.30 Hz
GB	0
PC	1.00

Openlynx Report Page 2

Vial:1:38 ID: File:QUA-B-59-3

Date:19-Sep-2022 Time:15:25:38

Name: Tasdique Quadery Date: 09.19.22 Notebook: QUA-B-59-3

Printed: Mon Sep 19 15:27:20 2022

3:(Time: 0.09) Combine (1:4) 2:MS ES- 3.3e+005

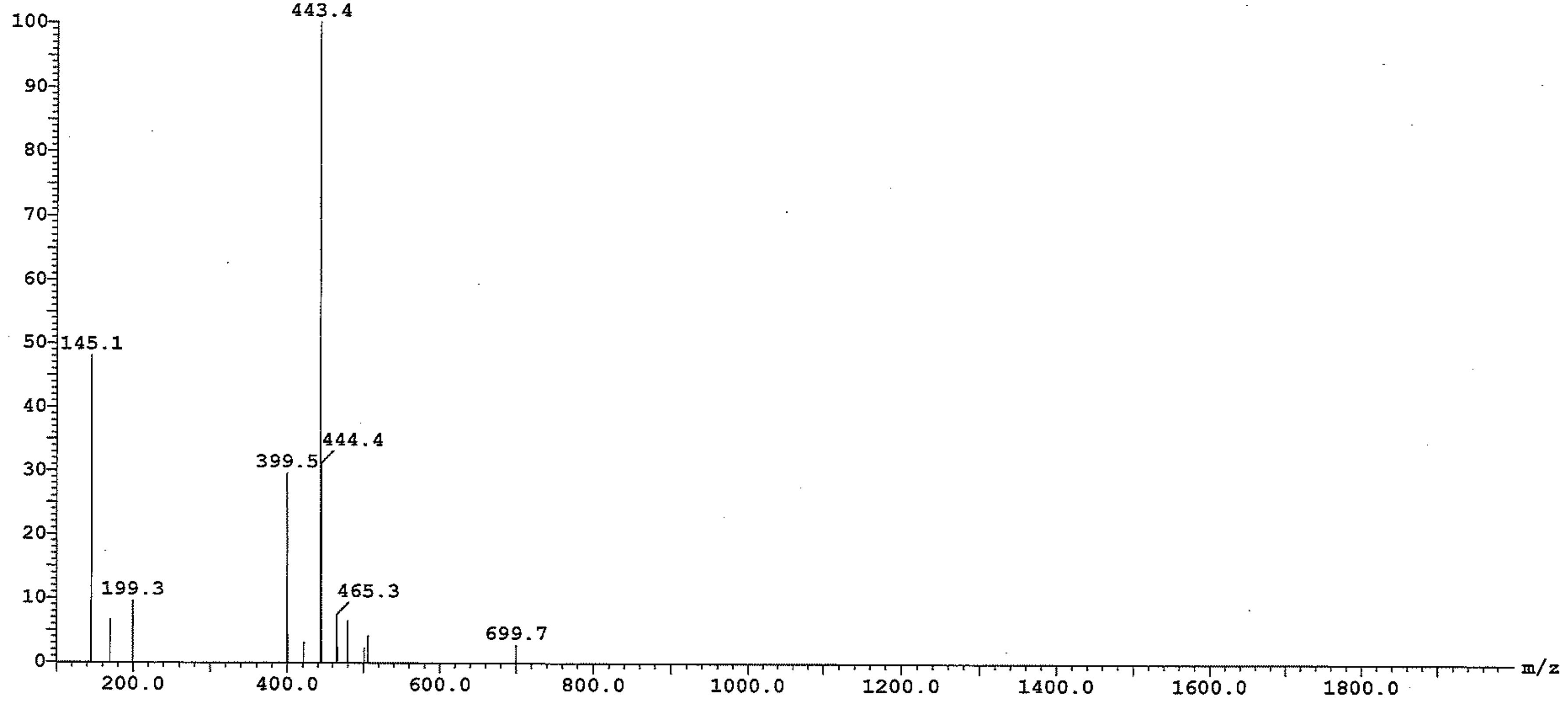

SAMPLE INFORMATION

Sample Name:	QUA-B-59-3
Injection Volume:	3.00 ul
Run Time:	9.0 Minutes
Date Acquired:	9/19/2022 3:33:58 PM EDT
Date Processed:	9/19/2022 3:53:52 PM EDT
Sample Set Name:	Template
Acq. Method Set:	BEH_C18_PDA_75mm 408
Processing Method:	BEH_C18_PDA
Channel Name:	254nm

Method Notes:
Acquity UPLC BEH C18 1.7u (2.1x75mm)
Flow Rate : 0.5 mL/min
Solvent A : 0.1% TFA in Waters
Solvent B : 0.1% TFA in Acetonitrile
Solvent Gradient Program:

Time (min)	%A	% B
0:00	95	5
6:00	0	100
8:00	0	100
9:00	95	5

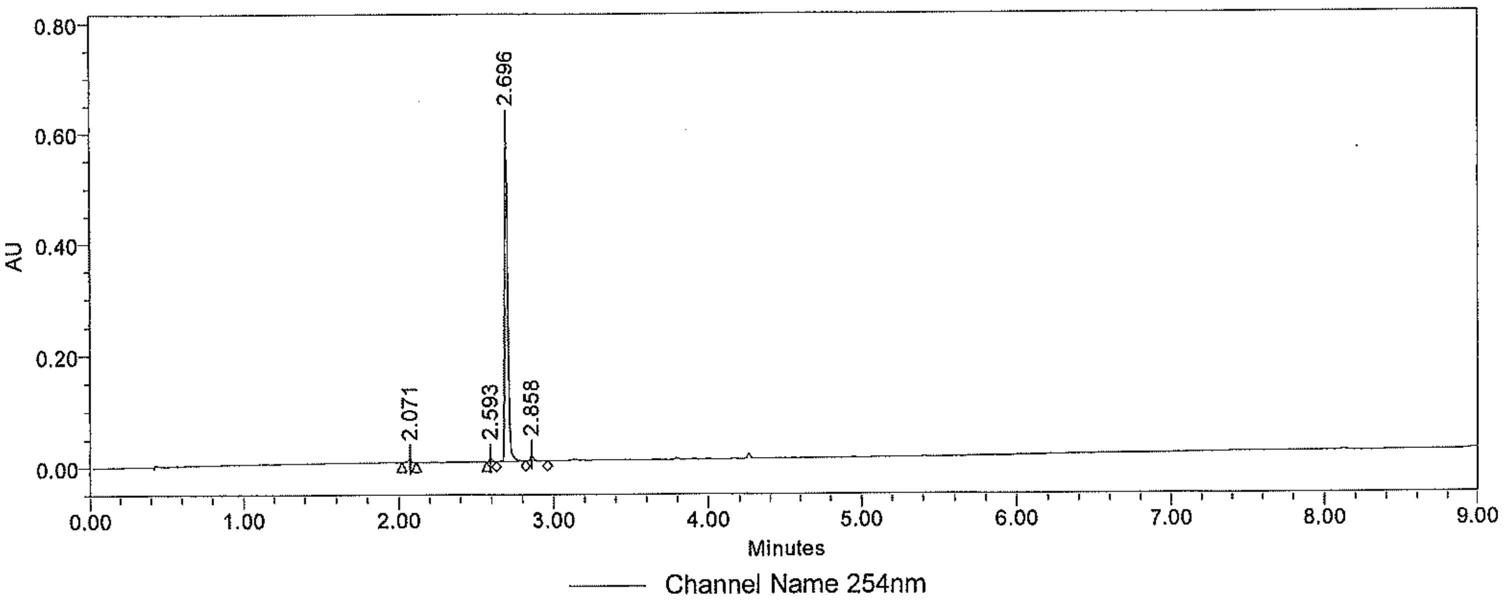

Peak Results

	RT	Area	Int Type	Width (sec)	% Area
1	2.071	9501	BB	5.550	1.09
2	2.593	7660	BV	3.700	0.88
3	2.696	828298	VV	11.449	95.43
4	2.858	22502	VV	8.449	2.59

Name: Tasdique Quadery

Date: 09.19.22

NB #: QUA-B-59-3

CERTIFICATE OF ANALYSIS

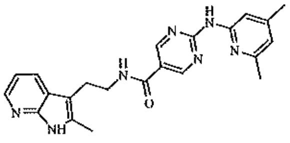

Compound Name: BPN-0036340-AA-001 1dd
ALB Number: ALB-232151
Batch: 1
Lot Number: QUA-B-96-4
Molecular Formula: $C_{22}H_{23}N_7O$
Molecular Weight: 401.46
Last Solvent: Water

TEST	RESULT/REFERENCE
Appearance	White Solid
NMR Spectrum	^{1}H, 500 MHz, Dimethyl Sulfoxide-d_6, Consistent - Attached
Mass Spectrum	ESI, *m/z* 402 $[M + H]^+$, Attached
UPLC	96.0% (area %), ACQUITY UPLC BEH C18 (2.1 *75) mm, 1.7 micron Column, UV 254 nm Detection, Attached

Maria Maychack
Approved By

11-16-2022
Date

For Research Purposes Only. Not Intended for Food or Drug Use.

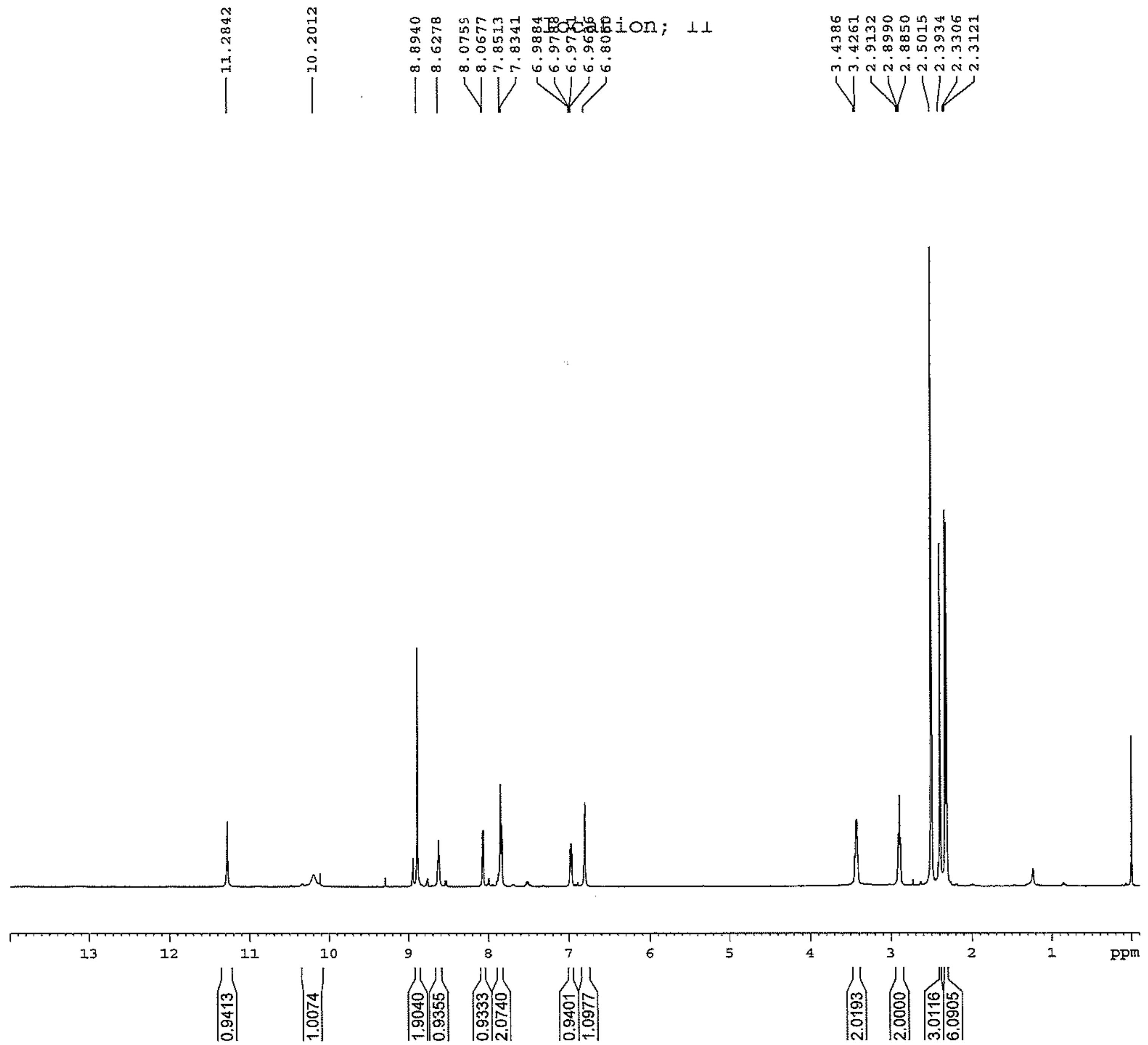

Name Tasdique Quadery

Date 11·14·22

NB # QUA-B-96-4

Current Data Parameters
NAME QUA-B-96-4
EXPNO 10
PROCNO 1

F2 - Acquisition Parameters
Date_ 20221114
Time_ 10.04 h
INSTRUM Avance Neo
PROBHD Z167419_0029 (
PULPROG zg30
TD 65536
SOLVENT DMSO
NS 16
DS 2
SWH 10000.000 Hz
FIDRES 0.305176 Hz
AQ 3.2767999 sec
RG 101
DW 50.000 usec
DE 11.14 usec
TE 300.0 K
D1 1.00000000 sec
TD0 1
SFO1 500.1330883 MHz
NUC1 1H
P0 2.67 usec
P1 8.00 usec
PLW1 24.22400093 W

F2 - Processing parameters
SI 65536
SF 500.1300035 MHz
WDW EM
SSB 0
LB 0.30 Hz
GB 0
PC 1.00

Openlynx Report
Vial:1:29
Date:11-Nov-2022
Name: Tasdique Quadery

ID:
Time:14:18:46
Date: 11·11·22

File:QUA-B-96-4
Notebook: QUA-B-96-4

Printed: Fri Nov 11 14:20:36 2022

1:(Time: 0.09) Combine (2:5)

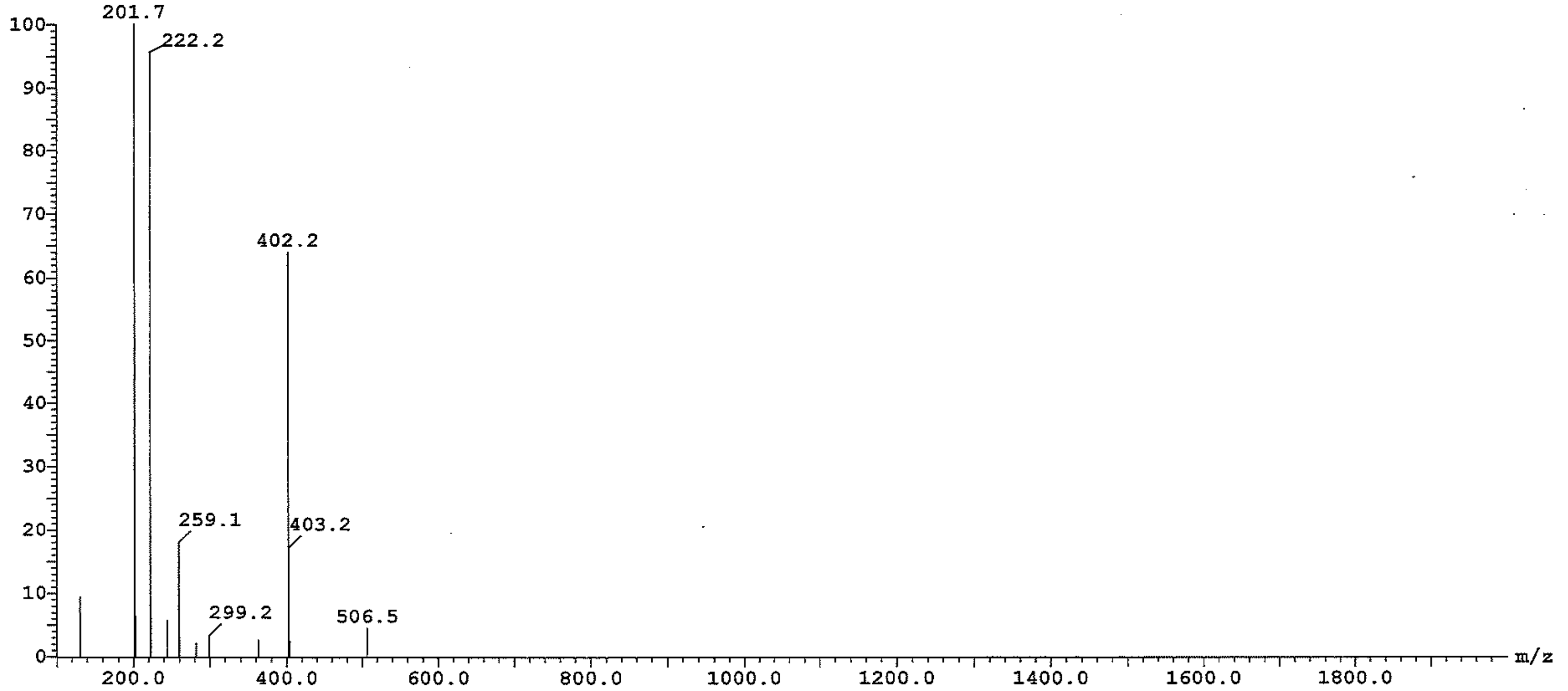

SAMPLE INFORMATION

Sample Name:	QUA-B-96-4
Injection Volume:	3.00 ul
Run Time:	9.0 Minutes
Date Acquired:	11/11/2022 2:01:07 PM EST
Date Processed:	11/11/2022 2:14:32 PM EST
Sample Set Name:	Template
Acq. Method Set:	BEH_C18_PDA_75mm
Processing Method:	BEH_C18_PDA
Channel Name:	254nm

Method Notes:
Acquity UPLC BEH C18 1.7u (2.1x75mm)
Flow Rate : 0.5 mL/min
Solvent A : 0.1% TFA in Waters
Solvent B : 0.1% TFA in Acetonitrile
Solvent Gradient Program:

Time (min)	%A	% B
0:00	95	5
6:00	0	100
8:00	0	100
9:00	95	5

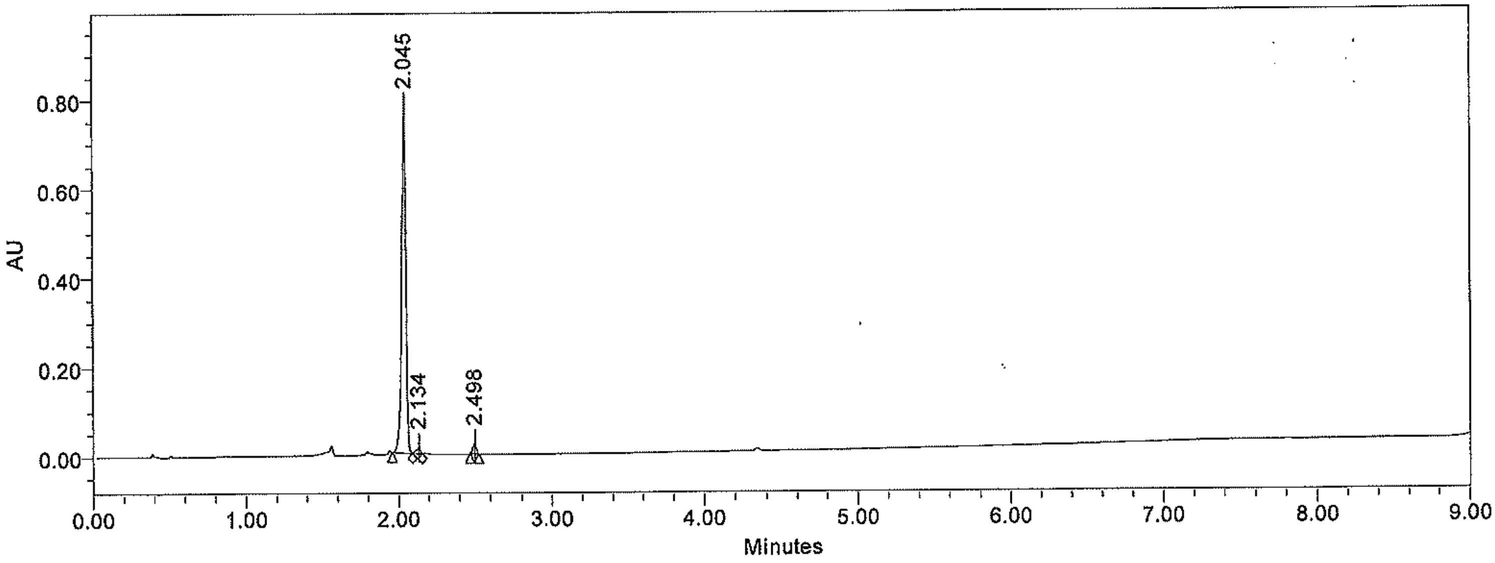

Peak Results

	RT	Area	Int Type	Width (sec)	% Area
1	2.045	1490192	BV	7.950	96.07
2	2.134	26286	VV	3.850	1.69
3	2.498	34649	bb	3.450	2.23

Name: Tasdique Quadery

Date: 11.11.22

NB #: QUA-B-96-4

CERTIFICATE OF ANALYSIS

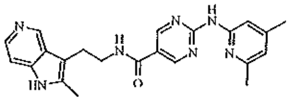

Compound Name: BPN-0036393-AA-001 1ee
ALB Number: ALB-232454
Batch: 1
Lot Number: ARN-E-154-3
Molecular Formula: $C_{22}H_{23}N_7O$
Molecular Weight: 401.46
Last Solvent: Water, Methylene Chloride, Ethyl Acetate

TEST	RESULT/REFERENCE
Appearance	Light Yellow Solid
NMR Spectrum	^{1}H, 500 MHz, Dimethyl Sulfoxide-d_6, Consistent - Attached
Mass Spectrum	ESI, *m/z* 400 $[M-H]^-$, Attached
UPLC	98.5% (area %), ACQUITY UPLC BEH C18 (2.1 *75) mm, 1.7 micron Column, UV 254 nm Detection, Attached

[illegible]

Approved By

12-7-2022

Date

For Research Purposes Only. Not Intended for Food or Drug Use.

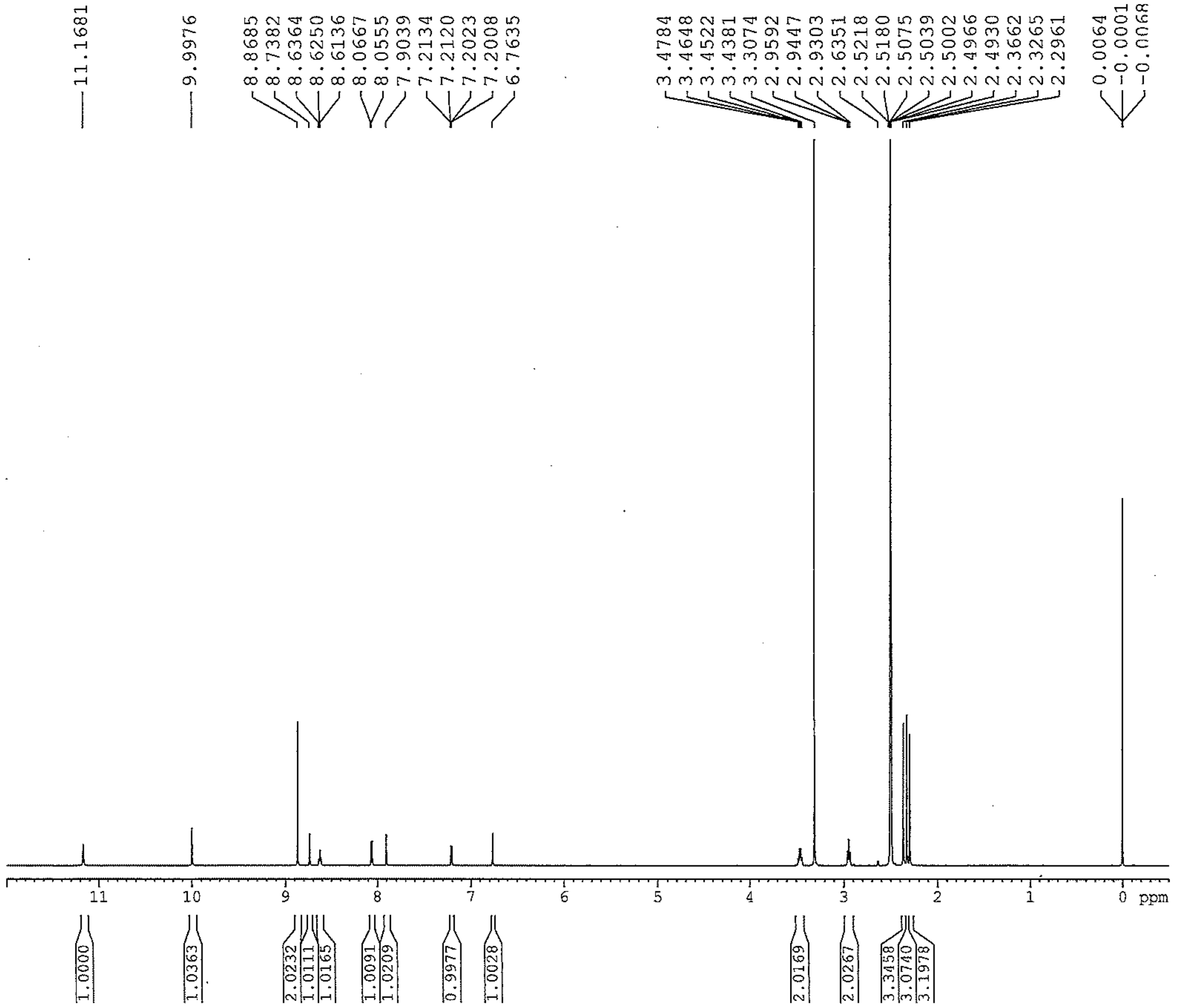

Name Corey Arnott-Butscher

Date 12/5/2022

NB # ARN-E-154-3

Current Data Parameters
NAME ARN-E-154-3
EXPNO 10
PROCNO 1

F2 - Acquisition Parameters
Date_ 20221202
Time 13.53 h
INSTRUM Avance Neo
PROBHD Z167419_0029 (
PULPROG zg30
TD 65536
SOLVENT DMSO
NS 32
DS 2
SWH 10000.000 Hz
FIDRES 0.305176 Hz
AQ 3.2767999 sec
RG 101
DW 50.000 usec
DE 11.14 usec
TE 300.0 K
D1 1.00000000 sec
TD0 1
SFO1 500.1330883 MHz
NUC1 1H
P0 2.67 usec
P1 8.00 usec
PLW1 24.22400093 W

F2 - Processing parameters
SI 65536
SF 500.1300041 MHz
WDW EM
SSB 0
LB 0.30 Hz
GB 0
PC 1.00

Openlynx Report

Page 2

Vial:2:5
Date:02-Dec-2022
ID:
Time:14:57:57
File:ARN-E-154-3

Name: Corey Arnett-Butscher
Date: 12/5/2022
Notebook: ARN-E-154-3

Printed: Fri Dec 02 15:00:11 2022

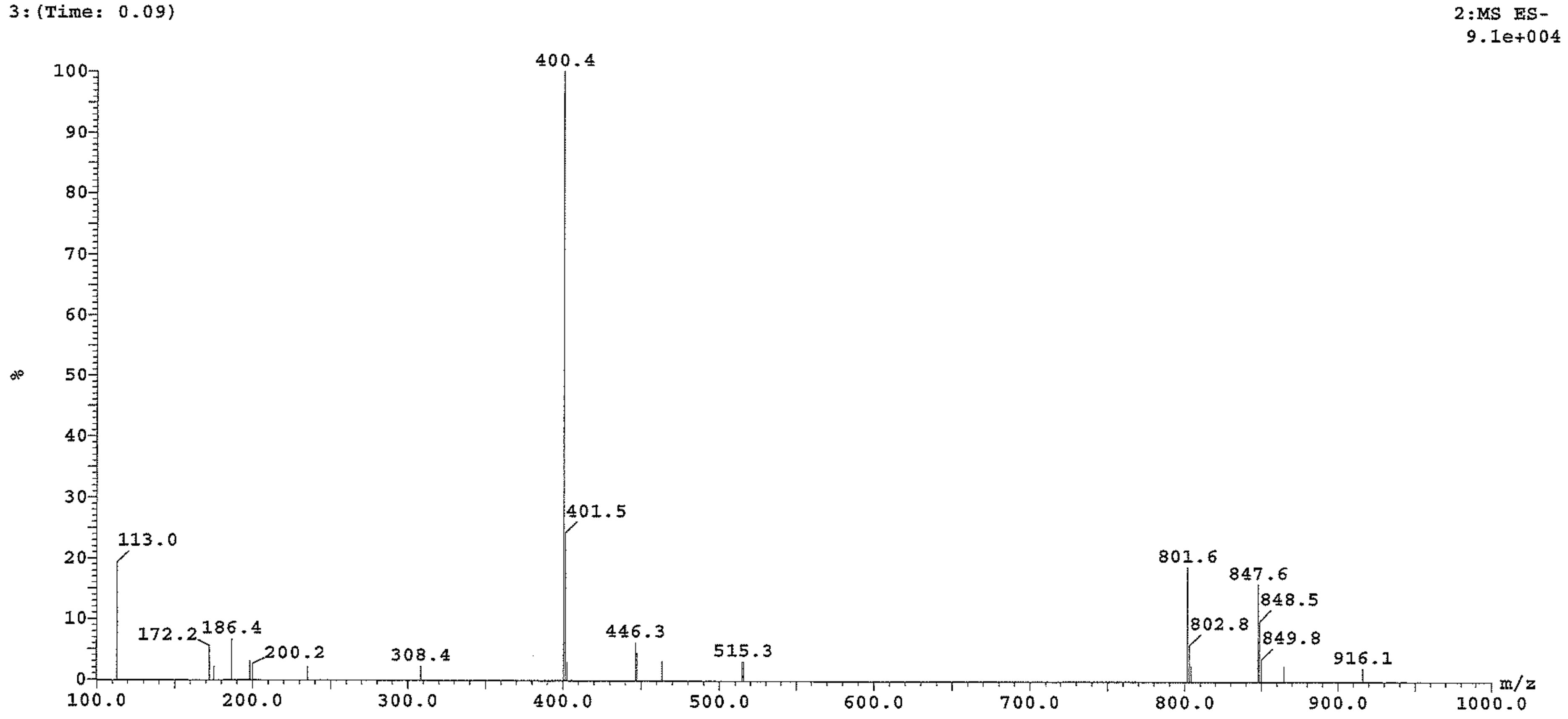

SAMPLE INFORMATION

Sample Name:	ARN-E-154-3
Injection Volume:	3.00 ul
Run Time:	9.0 Minutes
Date Acquired:	12/2/2022 3:18:21 PM EST
Date Processed:	12/5/2022 7:53:17 AM EST
Sample Set Name:	Template
Acq. Method Set:	BEH_C18_PDA_75mm 408
Processing Method:	BEH_C18_PDA_CAB
Channel Name:	254nm

Method Notes:
Acquity UPLC BEH C18 1.7u (2.1x75mm)
Flow Rate : 0.5 mL/min
Solvent A : 0.1% TFA in Waters
Solvent B : 0.1% TFA in Acetonitrile
Solvent Gradient Program:

Time (min)	%A	% B
0:00	95	5
6:00	0	100
8:00	0	100
9:00	95	5

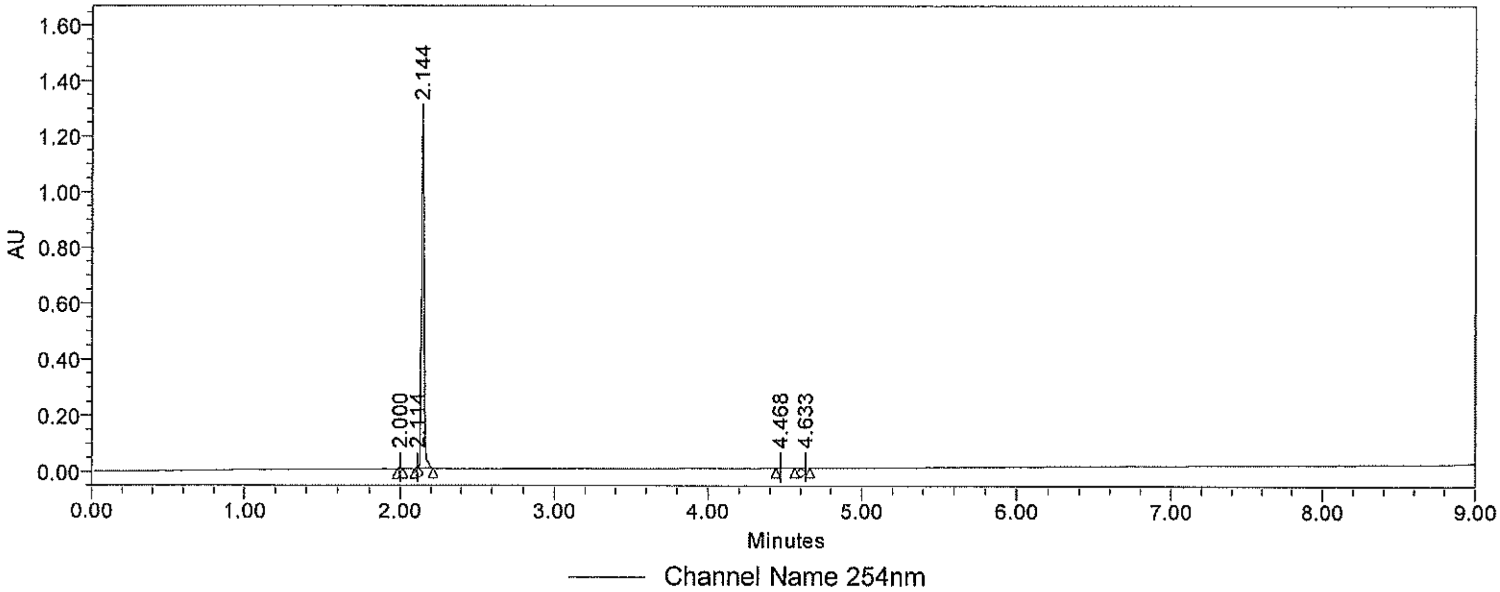

Peak Results

	RT	Area	Int Type	Width (sec)	% Area
1	2.000	5973	bb	2.250	0.40
2	2.114	4343	bV	1.550	0.29
3	2.144	1456423	Vb	5.700	98.59
4	4.468	5821	bB	7.402	0.39
5	4.633	4673	Vb	3.451	0.32

Name: CorcyArnett-Betscher

Date: 12/5/2022

NB #: ARN-E-154-3

CERTIFICATE OF ANALYSIS

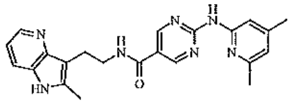

Compound Name: BPN-0036437-AA-001 1ff
ALB Number: ALB-232650
Batch: 1
Lot Number: ARN-E-167-3
Molecular Formula: $C_{22}H_{23}N_7O$
Molecular Weight: 401.46
Last Solvent: Water, Acetonitrile

TEST	RESULT/REFERENCE
Appearance	Off-white Solid
NMR Spectrum	^{1}H, 500 MHz, Methanol-d_4, Consistent - Attached
Mass Spectrum	ESI, *m/z* 402 $[M + H]^+$, Attached
UPLC	96.3% (area %), ACQUITY UPLC BEH C18 (2.1 *75) mm, 1.7 micron Column, UV 254 nm Detection, Attached

Maria Maychack
Approved By

1-4-2023
Date

For Research Purposes Only. Not Intended for Food or Drug Use.

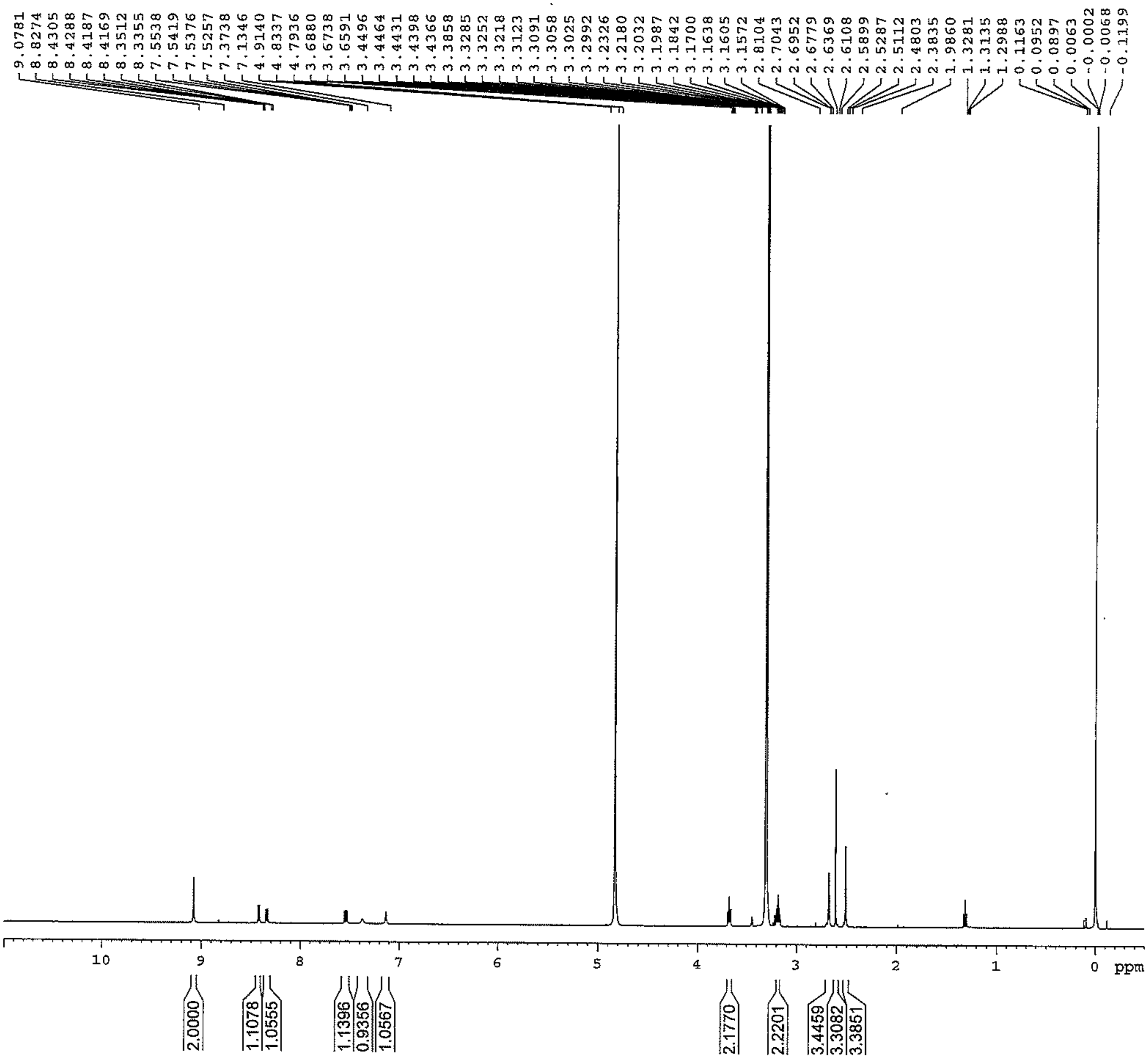

Name Cory Arnett-Butscher

Date 12/16/2022

NB # ARN-E-167-3

NAME	ARN-E-167-3
EXPNO	20
PROCNO	1
Date_	20221216
Time	9.01 h
INSTRUM	Avance Neo
PROBHD	Z167419_0029 (
PULPROG	zg30
TD	65536
SOLVENT	MeOD
NS	32
DS	2
SWH	10000.000 Hz
FIDRES	0.305176 Hz
AQ	3.2768500 sec
RG	101
DW	50.000 usec
DE	11.14 usec
TE	300.0 K
D1	1.00000000 sec
TD0	1
SFO1	500.1330883 MHz
NUC1	1H
P0	2.67 usec
P1	8.00 usec
SI	65536
SF	500.1300117 MHz
WDW	EM
SSB	0
LB	0.30 Hz
GB	0
PC	1.00

Openlynx Report

Page 1

Vial:1:7
Date:16-Dec-2022

ID:
Time:08:39:19

File:ARN-E-167-3

Name: Corey Arndt-Butscher

Date: 12/16/2022

Notebook: ARN-E-167-3

Printed: Fri Dec 16 08:41:34 2022

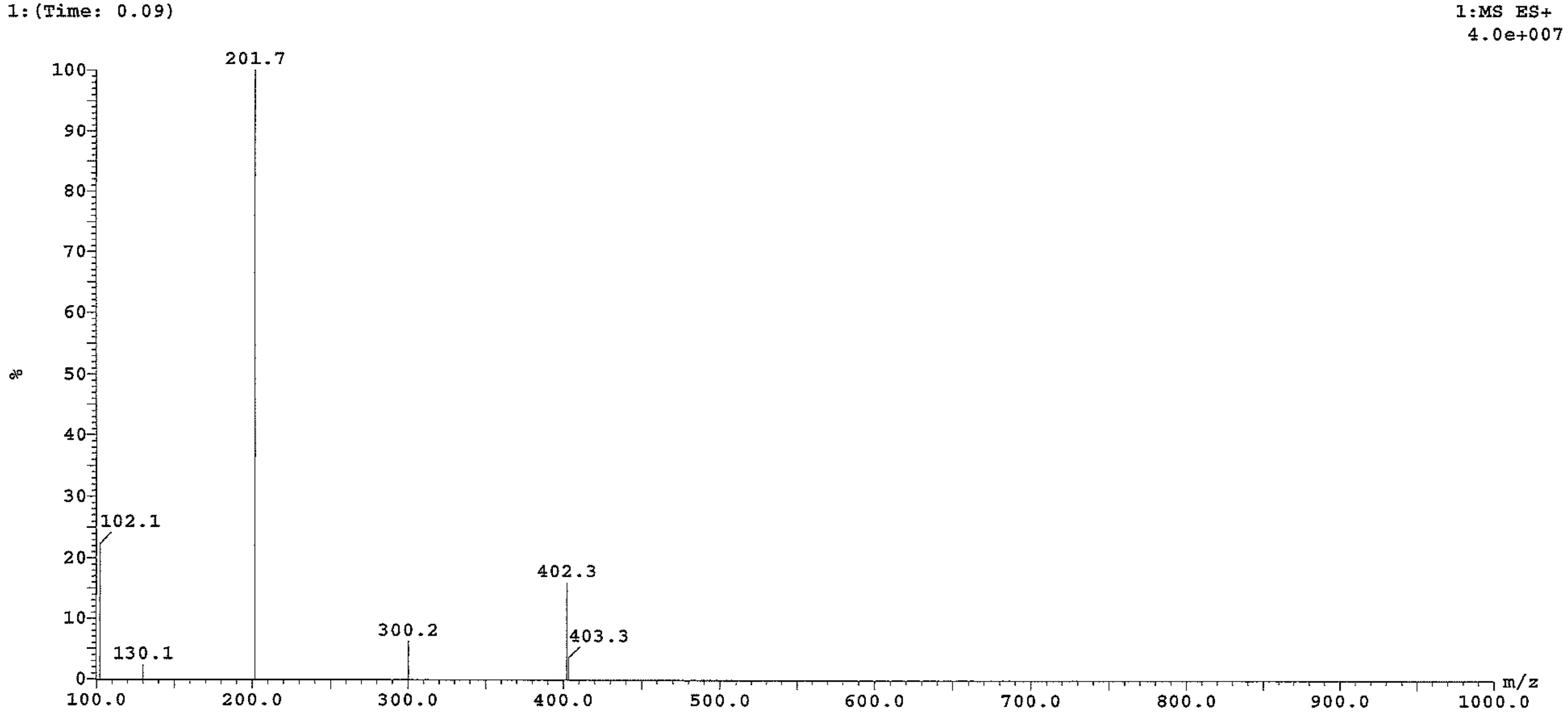

SAMPLE INFORMATION

Sample Name:	ARN-E-167-3
Injection Volume:	3.00 ul
Run Time:	9.0 Minutes
Date Acquired:	12/16/2022 8:17:16 AM EST
Date Processed:	12/16/2022 8:30:27 AM EST
Sample Set Name:	Template
Acq. Method Set:	BEH_C18_PDA_75mm 408
Processing Method:	BEH_C18_PDA_CAB
Channel Name:	254nm

Method Notes:
Acquity UPLC BEH C18 1.7u (2.1x75mm)
Flow Rate : 0.5 mL/min
Solvent A : 0.1% TFA in Waters
Solvent B : 0.1% TFA in Acetonitrile
Solvent Gradient Program:

Time (min)	%A	% B
0:00	95	5
6:00	0	100
8:00	0	100
9:00	95	5

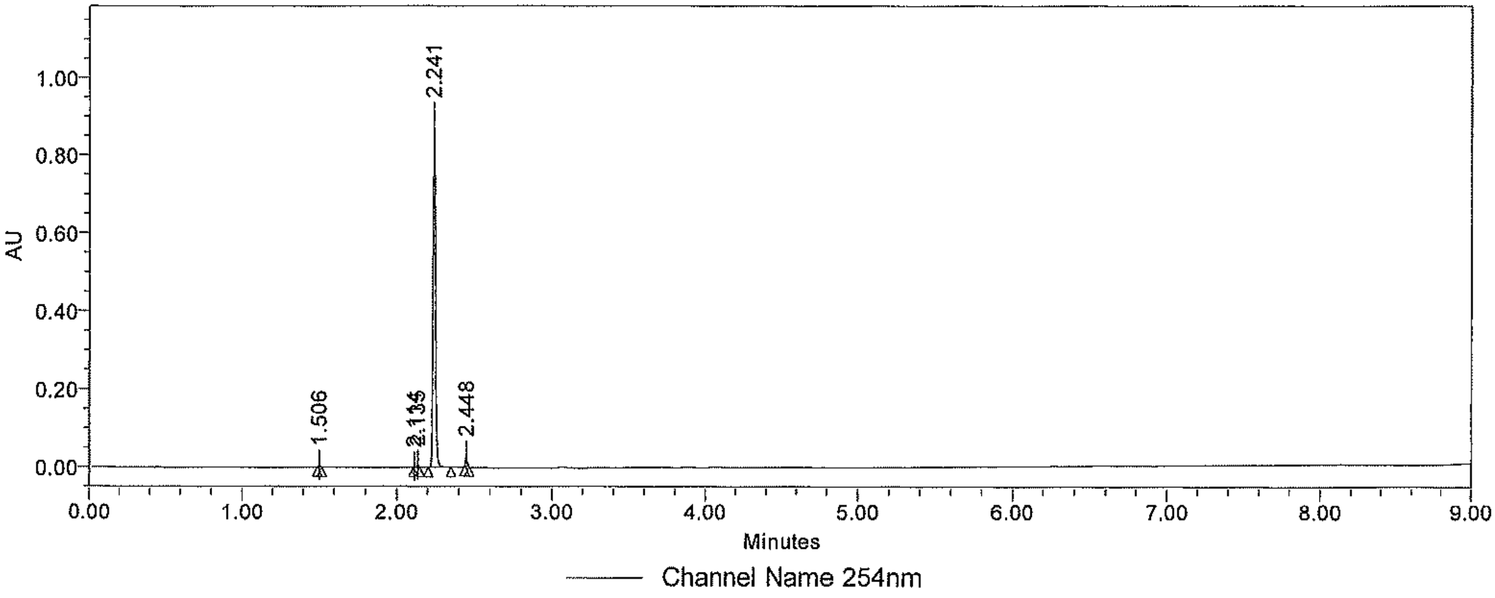

Peak Results

	RT	Area	Int Type	Width (sec)	% Area
1	1.506	4887	bb	1.650	0.51
2	2.114	1312	bV	1.050	0.14
3	2.135	4589	Vb	1.500	0.47
4	2.241	932174	bb	8.649	96.39
5	2.448	24107	bb	1.750	2.49

Name: Corey Arnett-Butscher

Date: 12/16/2022

NB #: ARN-E-167-3

CERTIFICATE OF ANALYSIS

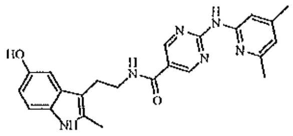

Compound Name: BPN-0036618-AA-001 1gg
ALB Number: ALB-233560
Batch: 1
Lot Number: QUA-B-152-1
Molecular Formula: $C_{23}H_{24}N_6O_2$
Molecular Weight: 416.48
Last Solvent: Acetonitrile, Water

TEST	RESULT/REFERENCE
Appearance	Off-white Solid
NMR Spectrum	^1H, 500 MHz, Dimethyl Sulfoxide-d_6, Consistent - Attached
Mass Spectrum	ESI, *m/z* 417 $[M + H]^+$, Attached
UPLC	95.1% (area %), ACQUITY UPLC BEH C18 (2.1 *75) mm, 1.7 micron Column, UV 254 nm Detection, Attached

Approved By: [illegible signature]

Date: 3-1-2023

For Research Purposes Only. Not Intended for Food or Drug Use.

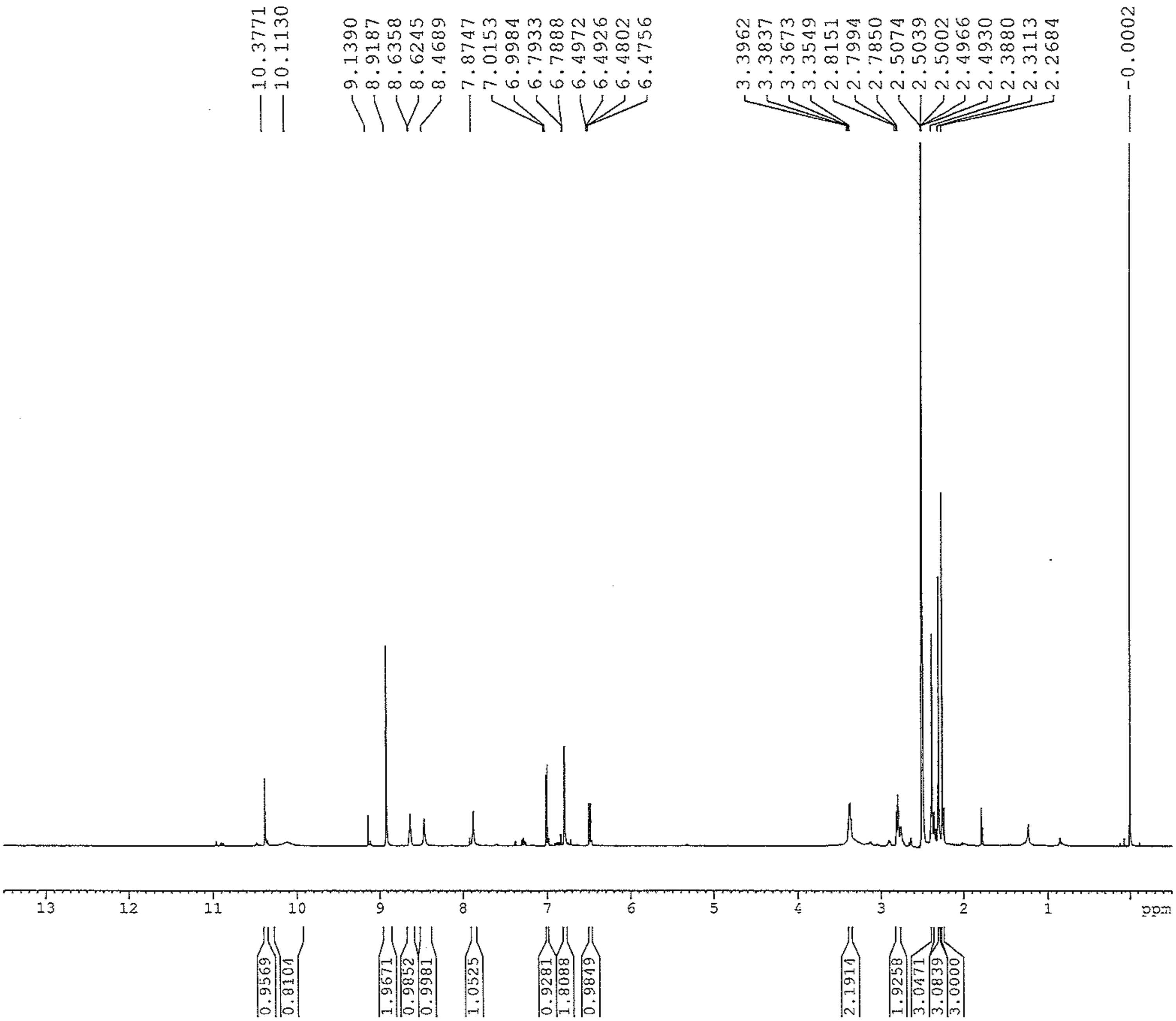

Name Tasdique Quadery
Date 02·24·23
NB # QUA-B-152-1

```
Current Data Parameters
NAME          QUA-B-152-1
EXPNO                  30
PROCNO                  1

F2 - Acquisition Parameters
Date_            20230224
Time                10.02 h
INSTRUM         Avance Neo
PROBHD    Z167419_0029 (
PULPROG              zg30
TD                  65536
SOLVENT              DMSO
NS                     16
DS                      2
SWH             10000.000 Hz
FIDRES           0.305176 Hz
AQ              3.2767999 sec
RG                    101
DW                 50.000 usec
DE                  11.14 usec
TE                  300.0 K
D1             1.00000000 sec
TD0                     1
SFO1          500.1330883 MHz
NUC1                   1H
P0                   2.67 usec
P1                   8.00 usec
PLW1          24.22400093 W

F2 - Processing parameters
SI                  65536
SF            500.1300041 MHz
WDW                    EM
SSB                     0
LB                   0.30 Hz
GB                      0
PC                   1.00
```

Openlynx Report
Vial:1:17
Date:20-Feb-2023
Name: Tasdique Quaderz

ID:
Time:09:22:48
Date: 02·20·23

File:QUA-B-152-1
Notebook: QUA-B-152-1

Printed: Mon Feb 20 09:24:58 2023

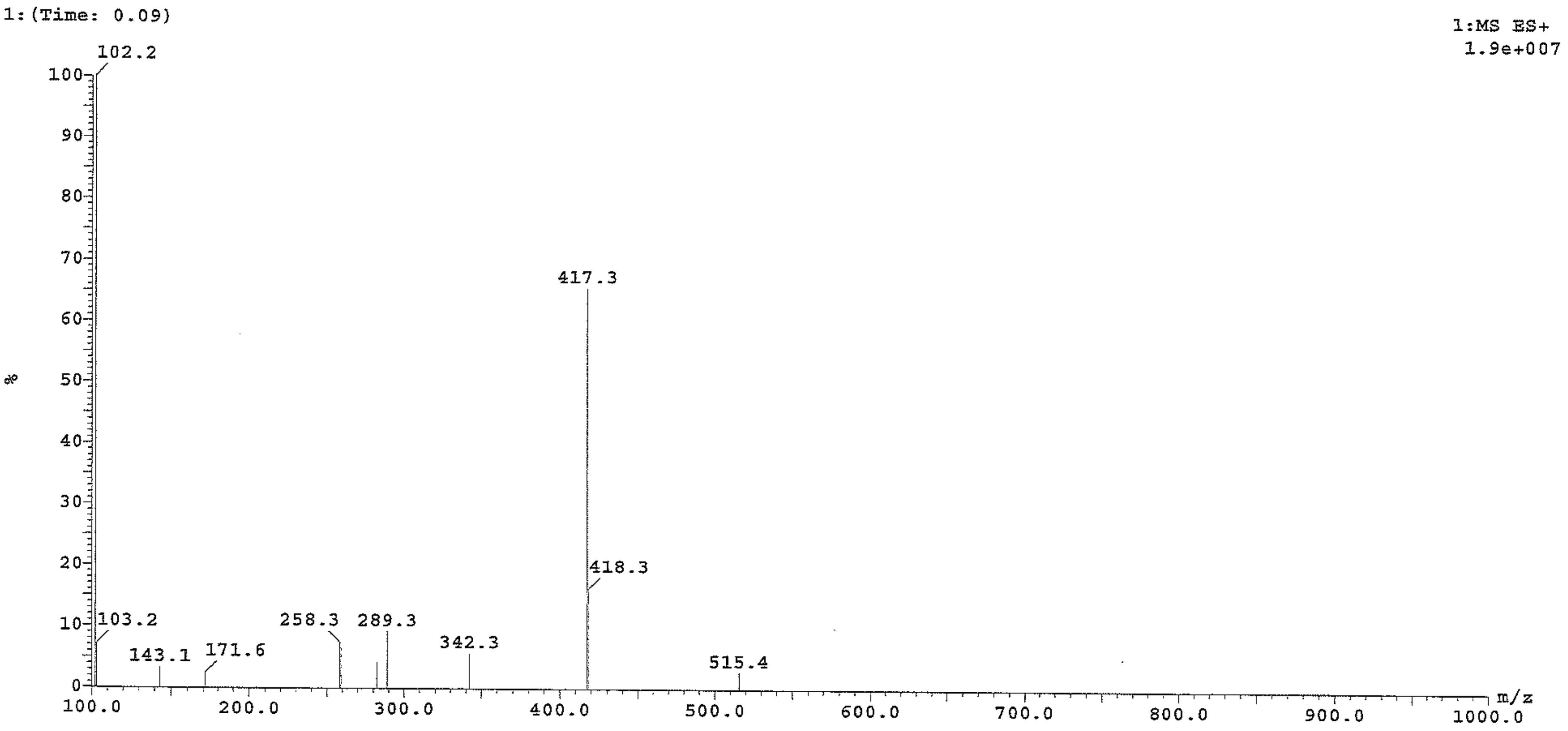

SAMPLE INFORMATION

Sample Name:	QUA-B-152-1
Injection Volume:	3.00 ul
Run Time:	9.0 Minutes
Date Acquired:	2/16/2023 4:03:24 PM EST
Date Processed:	2/16/2023 4:21:50 PM EST
Sample Set Name:	Template
Acq. Method Set:	BEH_C18_PDA_75mm
Processing Method:	BEH_C18_PDA
Channel Name:	254nm

Method Notes:
Acquity UPLC BEH C18 1.7u (2.1x75mm)
Flow Rate : 0.5 mL/min
Solvent A : 0.1% TFA in Waters
Solvent B : 0.1% TFA in Acetonitrile
Solvent Gradient Program:

Time (min)	%A	% B
0:00	95	5
6:00	0	100
8:00	0	100
9:00	95	5

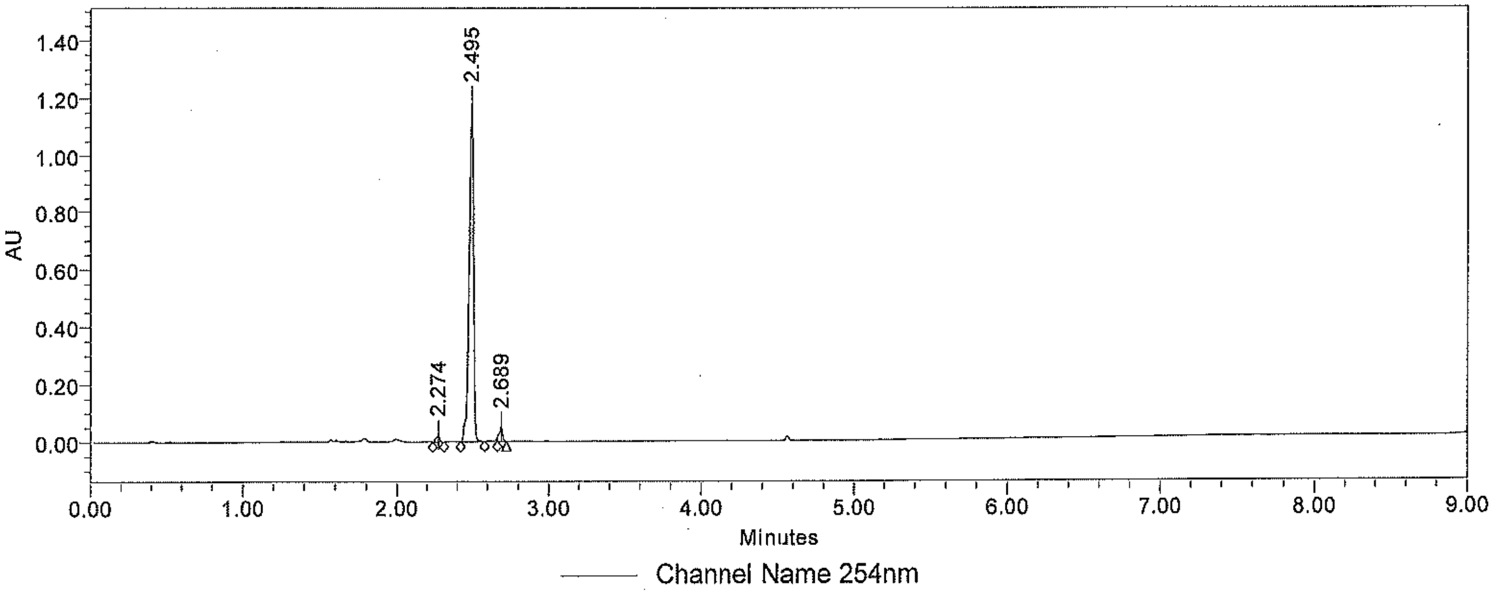

Peak Results

	RT	Area	Int Type	Width (sec)	% Area
1	2.274	43124	VV	4.350	1.69
2	2.495	2434909	VV	9.399	95.19
3	2.689	80015	vb	3.550	3.13

Name: Tasdique Quadery

Date: 02.16.23

NB #: QUA-B-152-1

CERTIFICATE OF ANALYSIS

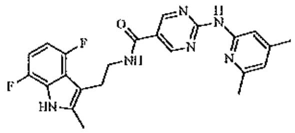

Compound Name: BPN-0036943-AA-001 1hh
ALB Number: ALB-234948
Batch: 1
Lot Number: CRA-A-16-2
Molecular Formula: $C_{23}H_{22}F_2N_6O$
Molecular Weight: 436.46
Last Solvent: Water, Acetonitrile

TEST	RESULT/REFERENCE
Appearance	White Solid
NMR Spectrum	^{1}H, 500 MHz, Dimethyl Sulfoxide-d_6, Consistent - Attached
Mass Spectrum	ESI, m/z 437 $[M + H]^+$, Attached
HPLC Analysis	96.8% (area %), ACQUITY UPLC BEH C18 Column, UV 254 nm Detection, Attached

Approved By: [signature]

Date: 5-31-2023

For Research Purposes Only. Not Intended for Food or Drug Use.

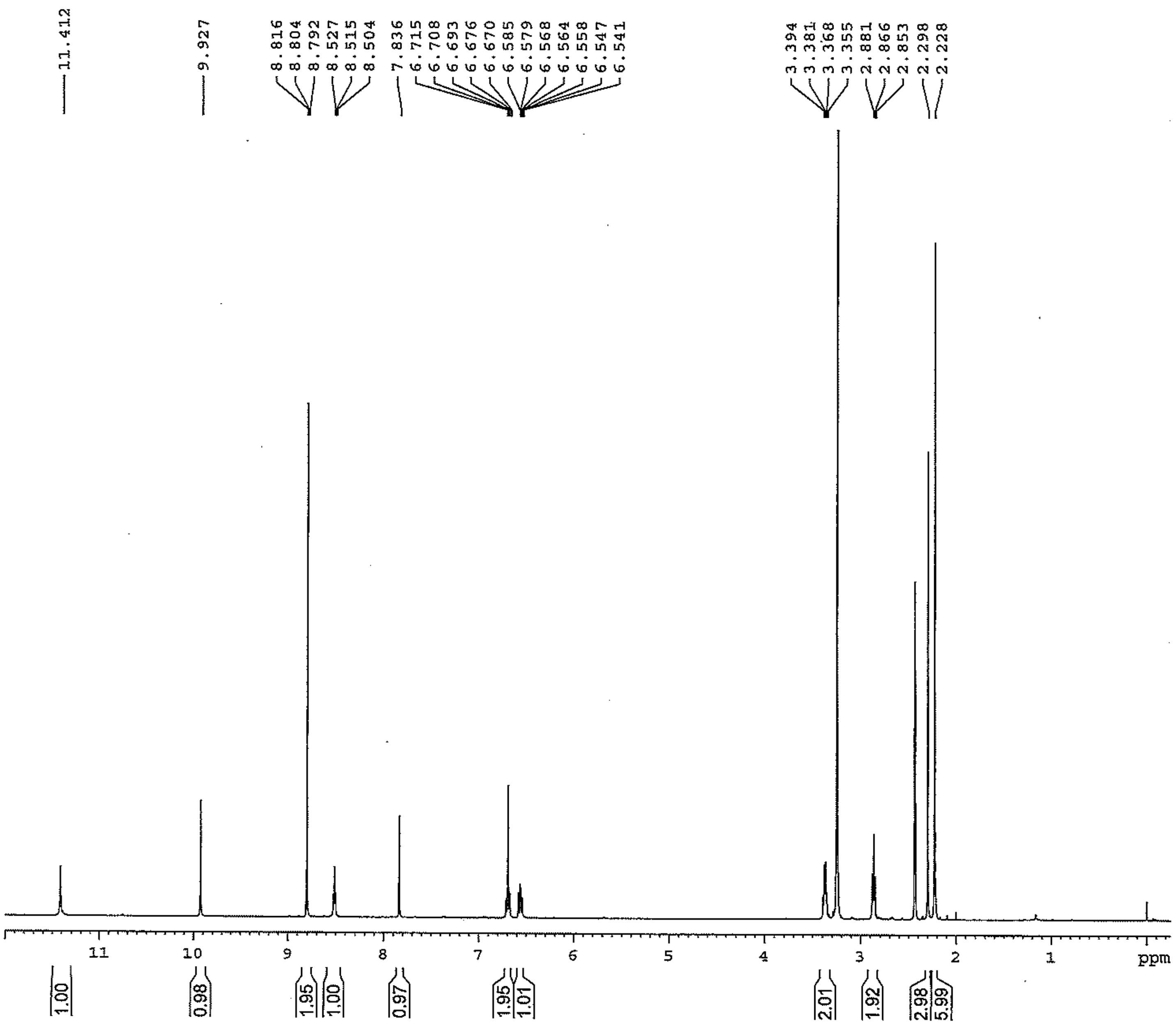

Name Ethan Osman

Date 26 May 2023

NB # CRA-A-16-2

NAME	CRA-A-16-2
EXPNO	10
PROCNO	1
Date_	20230526
Time	9.59 h
INSTRUM	Avance Neo
PROBHD	Z167419_0029 (
PULPROG	zg30
TD	65536
SOLVENT	DMSO
NS	32
DS	2
SWH	10000.000 Hz
FIDRES	0.305176 Hz
AQ	3.2768500 sec
RG	101
DW	50.000 usec
DE	11.14 usec
TE	300.0 K
D1	1.00000000 sec
TD0	1
SFO1	500.1330883 MHz
NUC1	1H
P0	2.67 usec
P1	8.00 usec
SI	65536
SF	500.1300376 MHz
WDW	EM
SSB	0
LB	0.30 Hz
GB	0
PC	1.00

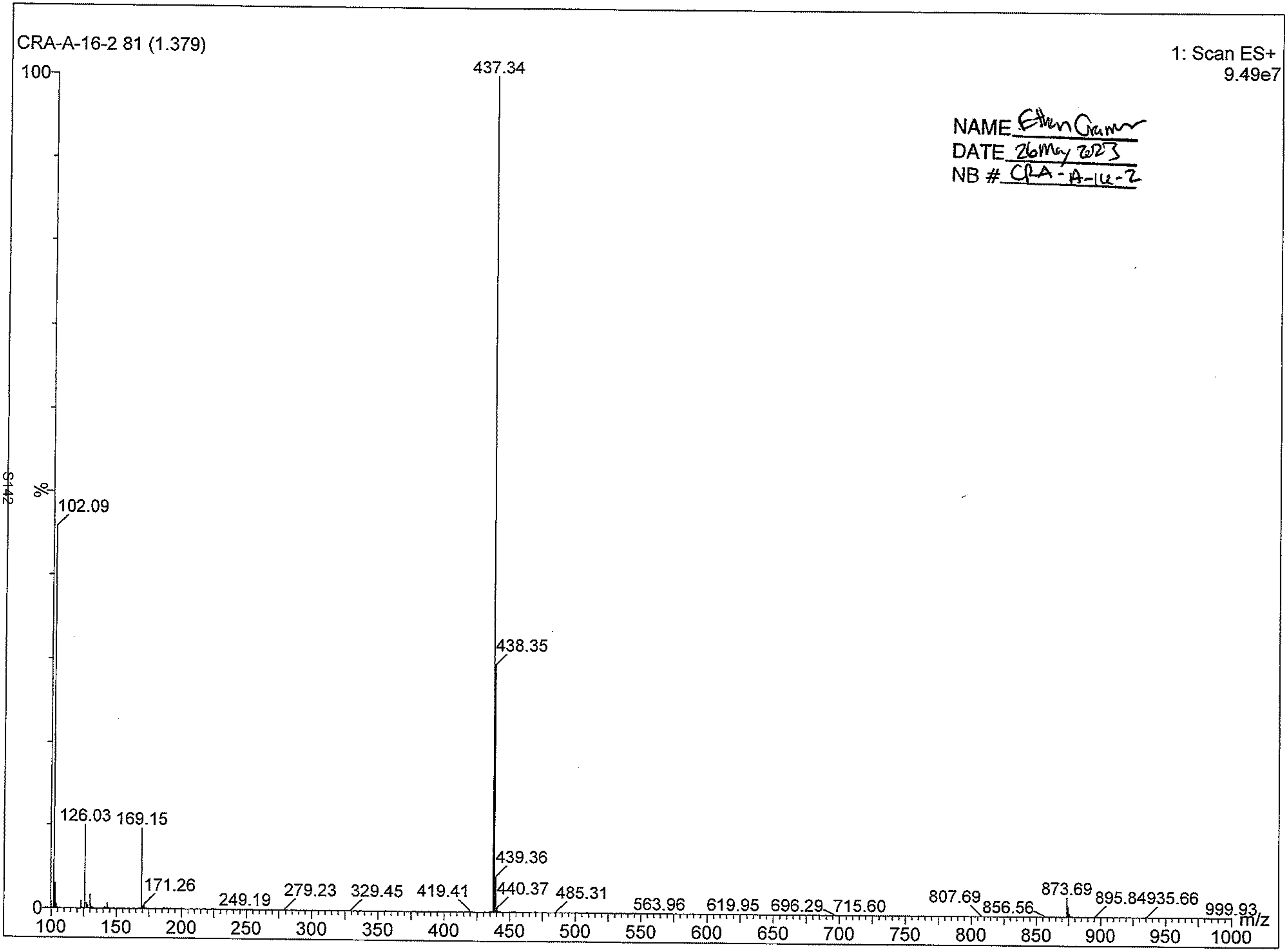

CRA-A-16-2 81 (1.379)
1: Scan ES+
9.49e7
NAME Ethan Cramer
DATE 26 May 2023
NB # CRA-A-16-2
100
%
0
437.34
102.09
438.35
126.03
169.15
171.26
249.19
279.23
329.45
419.41
439.36
440.37
485.31
563.96
619.95
696.29
715.60
807.69
856.56
873.69
895.84
935.66
999.93
m/z
100
150
200
250
300
350
400
450
500
550
600
650
700
750
800
850
900
950
1000

SAMPLE INFORMATION

Sample Name: CRA-A-16-2
Injection Volume: 7.00 ul
Run Time: 9.0 Minutes
Date Acquired: 5/26/2023 10:09:34 AM EDT
Date Processed: 5/26/2023 10:21:35 AM EDT
Sample Set Name: Template
Acq. Method Set: BEH_C18_PDA_75mm
Processing Method: BEH_C18_PDA
Channel Name: 254nm

Method Notes:
Acquity UPLC BEH C18 1.7u (2.1x75mm)
Flow Rate : 0.5 mL/min
Solvent A : 0.1% TFA in Waters
Solvent B : 0.1% TFA in Acetonitrile
Solvent Gradient Program:

Time (min)	%A	% B
0:00	95	5
6:00	0	100
8:00	0	100
9:00	95	5

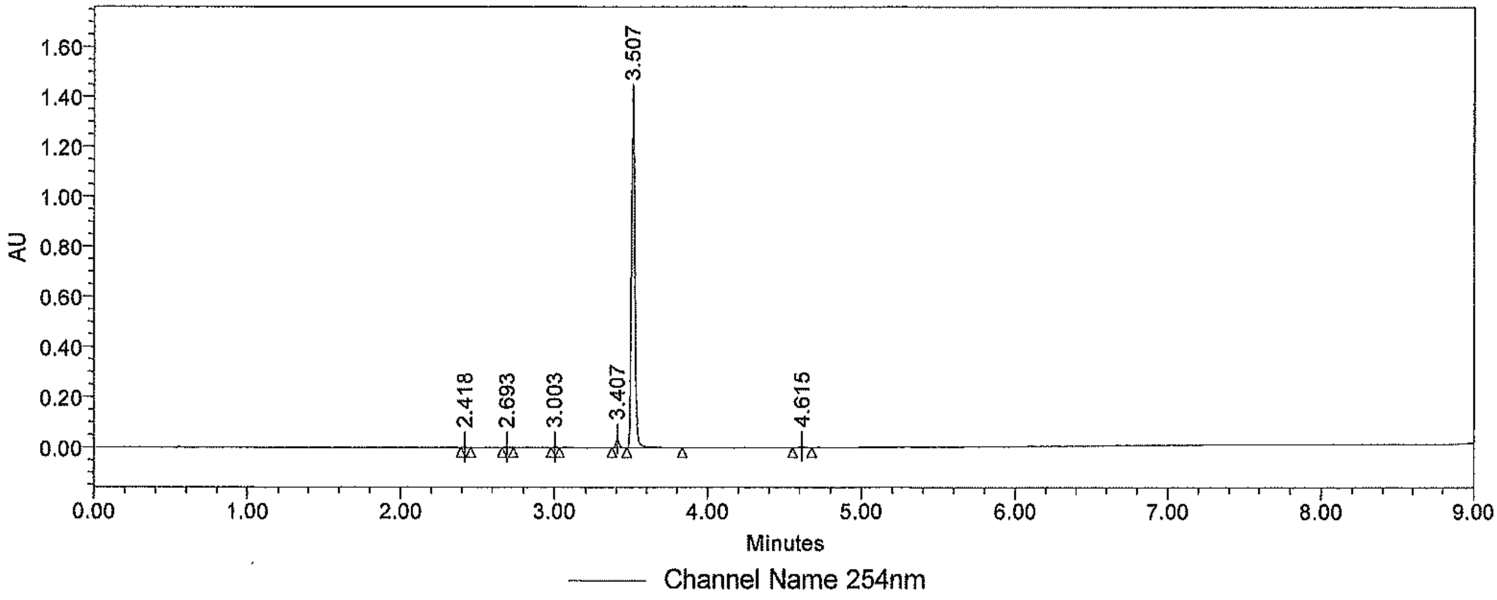

Peak Results

	RT	Area	Int Type	Width (sec)	% Area
1	2.418	3571	BB	3.750	0.15
2	2.693	3195	BB	4.250	0.13
3	3.003	3868	BB	3.250	0.16
4	3.407	53238	BB	5.800	2.24
5	3.507	2306322	BB	21.948	96.84
6	4.615	11469	BB	7.502	0.48

Name: Ethan Cramer

Date: 26 May 2023

NB #: CRA-A-16-2

CERTIFICATE OF ANALYSIS

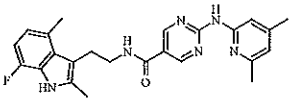

Compound Name: BPN-0036975-AA-001 **1ii**
ALB Number: ALB-235238
Batch: 1
Lot Number: ALK-C-202-4
Molecular Formula: $C_{24}H_{25}FN_6O$
Molecular Weight: 432.49
Last Solvent: Water, Acetonitrile

TEST	RESULT/REFERENCE
Appearance	Off-white Solid
NMR Spectrum	^{1}H, 500 MHz, Dimethyl Sulfoxide-d_6, Consistent - Attached
Mass Spectrum	ESI, m/z 433 $[M + H]^+$, Attached
UPLC	96.1% (area %), ACQUITY UPLC BEH C18 (2.1 *75) mm, 1.7 micron Column, UV 254 nm Detection, Attached

Approved By: Mauro Maychack (signature)

Date: 6-14-2023

For Research Purposes Only. Not Intended for Food or Drug Use.

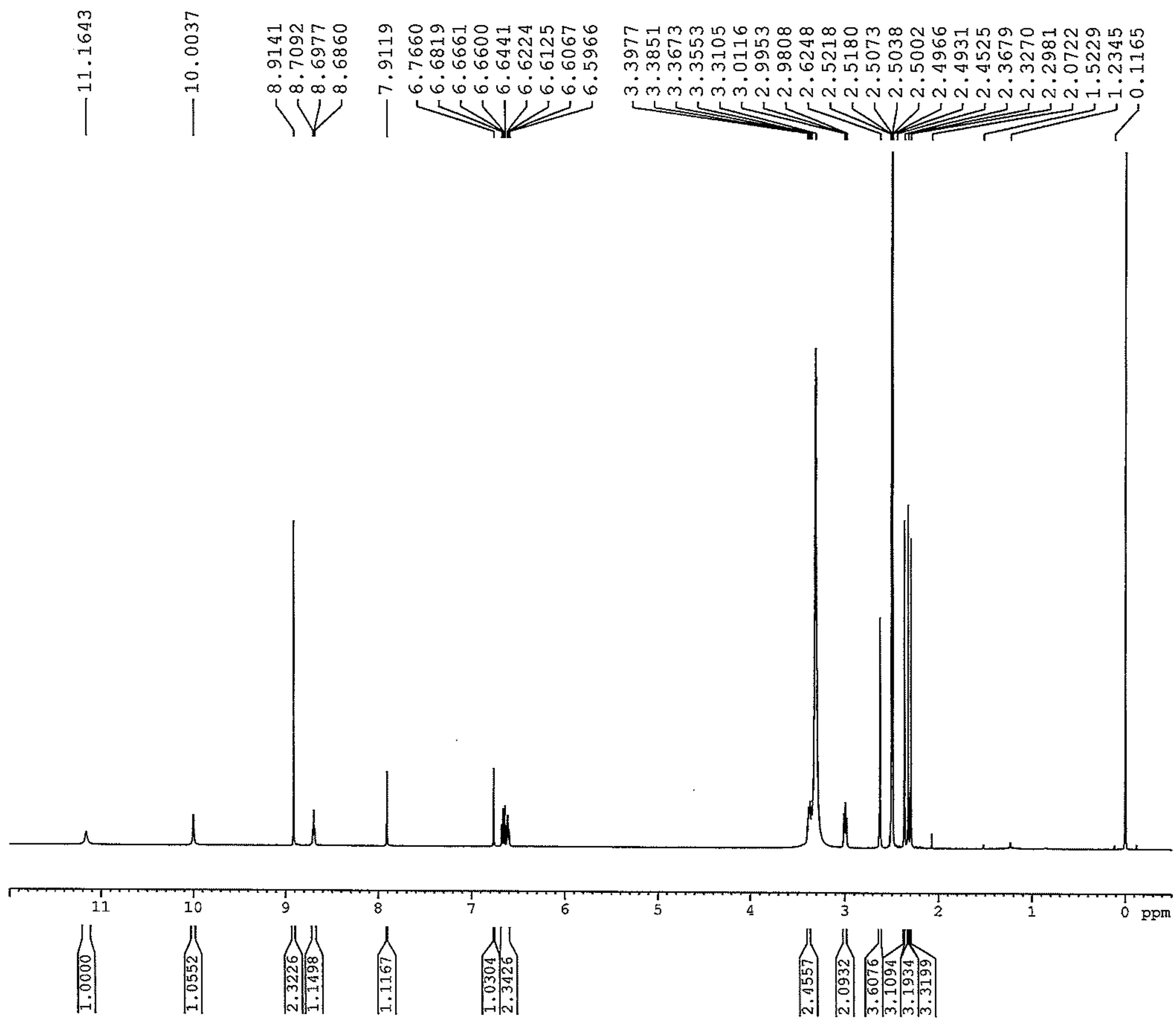

Name Marwah Albaker

Date 6-Jun-2023

NB # ALK-C-202-4

```
Current Data Parameters
NAME          ALK-C-202-4
EXPNO                  10
PROCNO                  1

F2 - Acquisition Parameters
Date_            20230606
Time                 8.18 h
INSTRUM        Avance Neo
PROBHD   Z167419_0029 (
PULPROG              zg30
TD                  65536
SOLVENT              DMSO
NS                     64
DS                      2
SWH             10000.000 Hz
FIDRES           0.305176 Hz
AQ              3.2767999 sec
RG                    101
DW                 50.000 usec
DE                  11.14 usec
TE                  300.0 K
D1             1.00000000 sec
TD0                     1
SFO1          500.1330883 MHz
NUC1                   1H
P0                   2.67 usec
P1                   8.00 usec
PLW1          24.22400093 W

F2 - Processing parameters
SI                  65536
SF            500.1300041 MHz
WDW                    EM
SSB                     0
LB                   0.30 Hz
GB                      0
PC                   1.00
```

ACQ-SQD#F07SQD100W

05-Jun-2023
13:13:02

Name: Marwah Albaker
Date: 5-Jun-2023
Notebook: ALK-C-202-4

ALK-C-202-4 641 (1.305)

1: Scan ES+
2.21e7

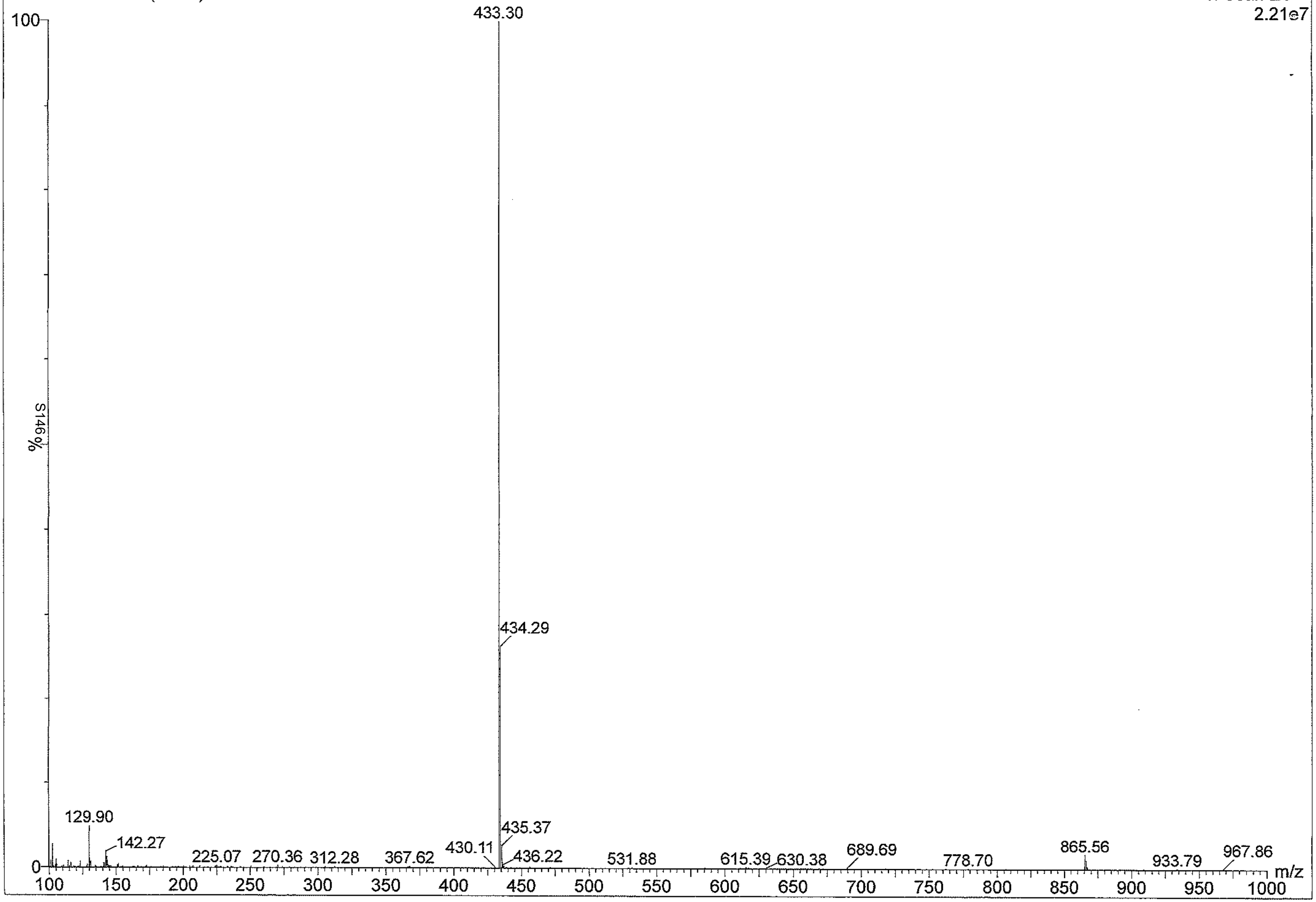

SAMPLE INFORMATION

Sample Name: ALK-C-202-4
Injection Volume: 3.00 ul
Run Time: 9.0 Minutes
Date Acquired: 6/5/2023 1:24:29 PM EDT
Date Processed: 6/5/2023 1:49:48 PM EDT
Sample Set Name: Template
Acq. Method Set: BEH_C18_PDA_75mm
Processing Method: BEH_C18_PDA
Channel Name: 254nm

Method Notes:
Acquity UPLC BEH C18 1.7u (2.1x75mm)
Flow Rate : 0.5 mL/min
Solvent A : 0.1% TFA in Waters
Solvent B : 0.1% TFA in Acetonitrile
Solvent Gradient Program:

Time (min)	%A	% B
0:00	95	5
6:00	0	100
8:00	0	100
9:00	95	5

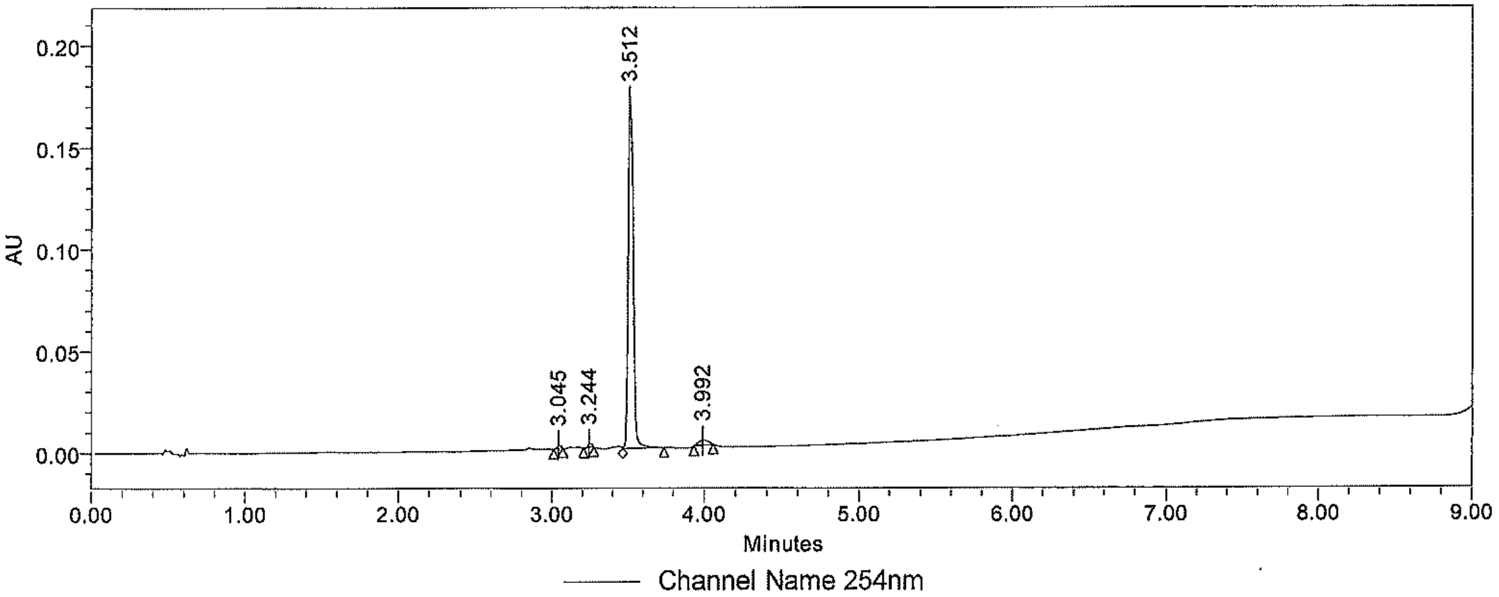

Peak Results

	RT	Area	Int Type	Width (sec)	% Area
1	3.045	2547	bb	3.600	0.61
2	3.244	3019	bb	4.050	0.73
3	3.512	398973	VB	16.449	96.13
4	3.992	10502	bb	7.300	2.53

Name: Marwah Albaker

Date: 6-Jun-2023

NB #: ALK-C-202-4

CERTIFICATE OF ANALYSIS

Compound Name: BPN-0036957-AA-001
ALB Number: ALB-235130
Batch: 1
Lot Number: ALK-C-200-3
Molecular Formula: $C_{21}H_{21}N_3O_2$
Molecular Weight: 347.41
Last Solvent: Water, Acetonitrile

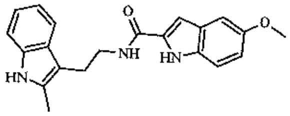

2a. BPN- 36957

TEST	RESULT/REFERENCE
Appearance	Off-white Solid
NMR Spectrum	^{1}H, 500 MHz, Dimethyl Sulfoxide-d_6, Consistent - Attached
Mass Spectrum	ESI, *m/z* 348 $[M + H]^+$, Attached
UPLC	>99% (area %), ACQUITY UPLC BEH C18 (2.1 *75) mm, 1.7 micron Column, UV 254 nm Detection, Attached

Approved By

6-7-2023
Date

For Research Purposes Only. Not Intended for Food or Drug Use.

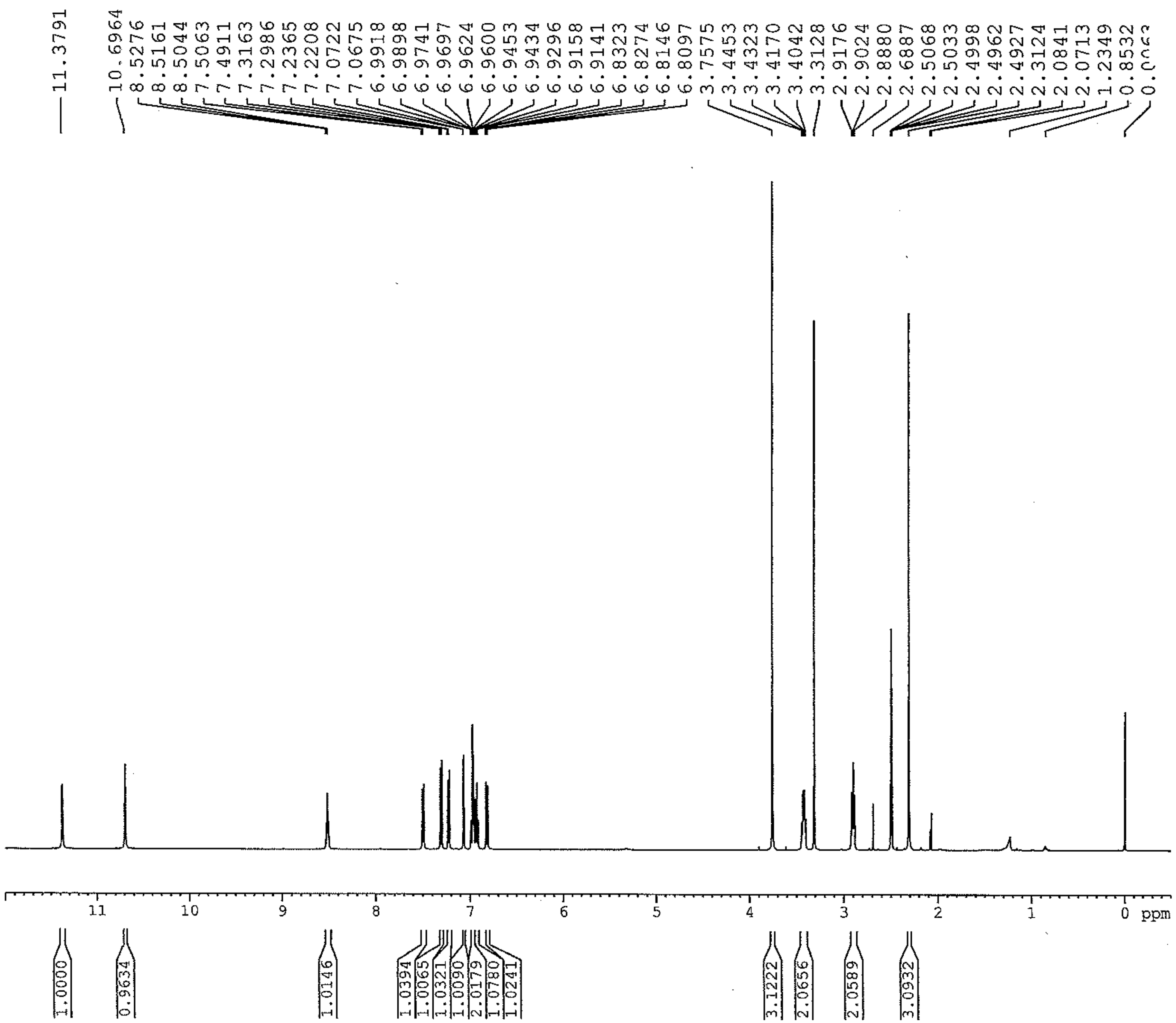

Name Marwah Albaker
Date 31-May-2023
NB # ALK-C-200-3

Current Data Parameters
NAME ALK-C-200-3
EXPNO 10
PROCNO 1

F2 - Acquisition Parameters
Date_ 20230531
Time 8.08 h
INSTRUM Avance Neo
PROBHD Z167419_0029 (
PULPROG zg30
TD 65536
SOLVENT DMSO
NS 16
DS 2
SWH 10000.000 Hz
FIDRES 0.305176 Hz
AQ 3.2767999 sec
RG 101
DW 50.000 usec
DE 11.14 usec
TE 300.0 K
D1 1.00000000 sec
TD0 1
SFO1 500.1330883 MHz
NUC1 1H
P0 2.67 usec
P1 8.00 usec
PLW1 24.22400093 W

F2 - Processing parameters
SI 65536
SF 500.1300043 MHz
WDW EM
SSB 0
LB 0.30 Hz
GB 0
PC 1.00

ACQ-SQD#F07SQD100W

30-May-2023
09:36:25

Name: Marwah Albaker
Date: 30-May-2023
Notebook: ALK-C-200-3

ALK-C-200-3 817 (1.663)

1: Scan ES+
5.65e6

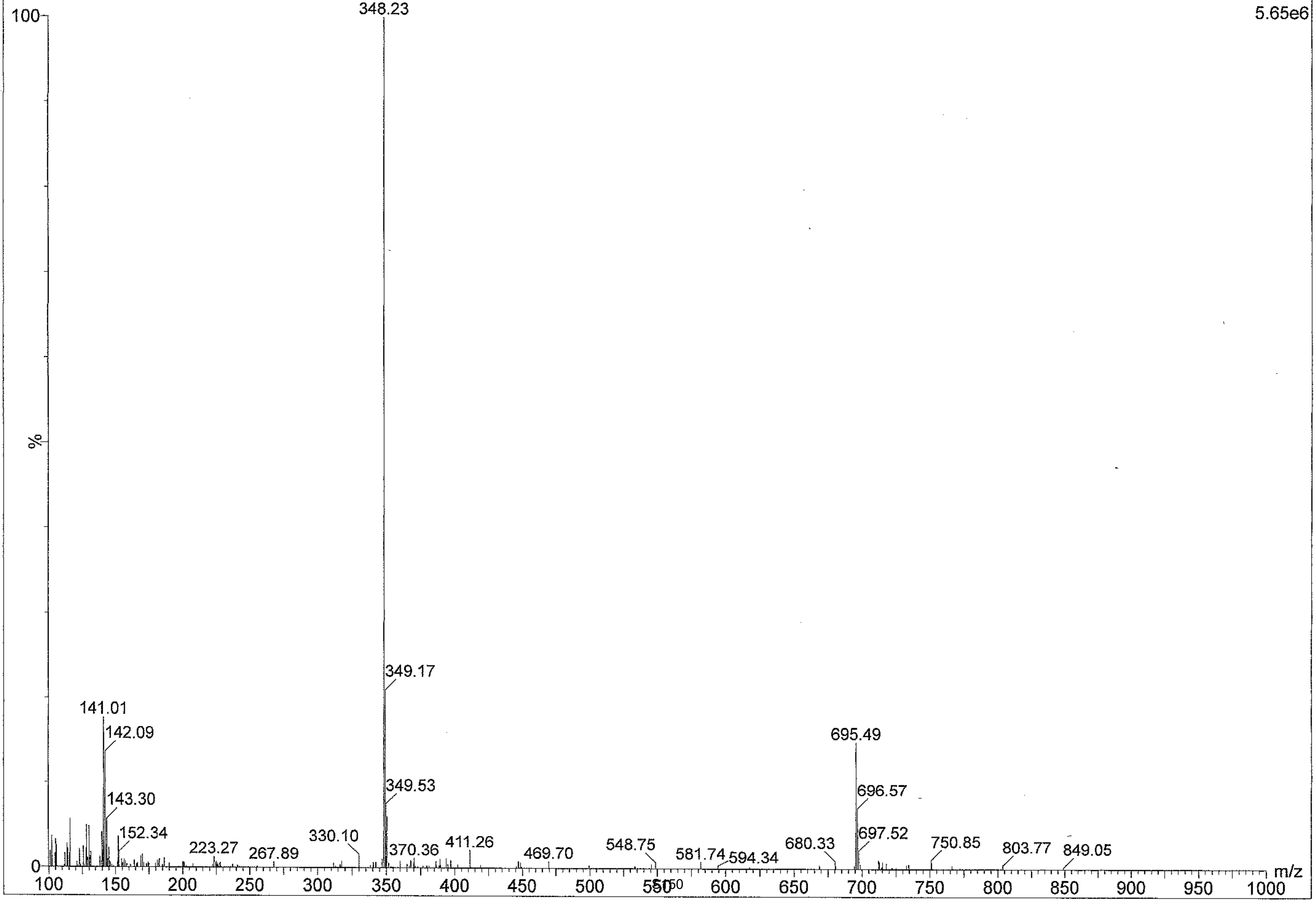

SAMPLE INFORMATION

Sample Name: ALK-C-200-3
Injection Volume: 3.00 ul
Run Time: 9.0 Minutes
Date Acquired: 5/30/2023 9:56:07 AM EDT
Date Processed: 5/30/2023 10:08:10 AM EDT
Sample Set Name: Template
Acq. Method Set: BEH_C18_PDA_75mm
Processing Method: BEH_C18_PDA
Channel Name: 254nm

Method Notes:
Acquity UPLC BEH C18 1.7u (2.1x75mm)
Flow Rate : 0.5 mL/min
Solvent A : 0.1% TFA in Waters
Solvent B : 0.1% TFA in Acetonitrile
Solvent Gradient Program:

Time (min)	%A	% B
0:00	95	5
6:00	0	100
8:00	0	100
9:00	95	5

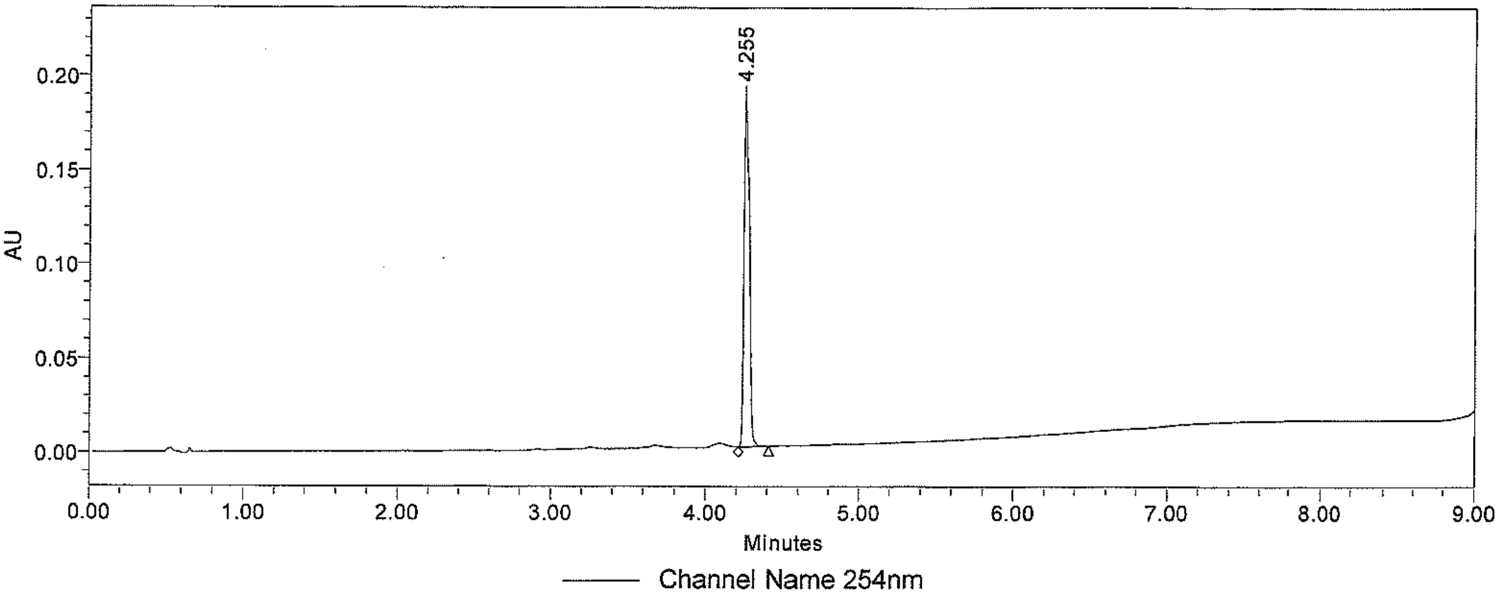

Peak Results

	RT	Area	Int Type	Width (sec)	% Area
1	4.255	454432	VB	11.953	100.00

Name: Marwah Albaker

Date: 30-May-2023

NB #: ALK-C-200-3

CERTIFICATE OF ANALYSIS

Compound Name: BPN-0037313-AA-001
ALB Number: ALB-236858
Batch: 1
Lot Number: ALK-D-77-3
Molecular Formula: $C_{22}H_{23}N_3O_2$
Molecular Weight: 361.44
Last Solvent: Water, Acetonitrile

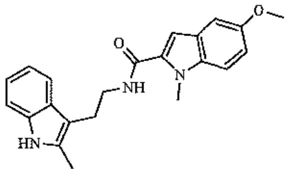

2b. BPN- 37313

TEST	RESULT/REFERENCE
Appearance	Off-white Solid
NMR Spectrum	^{1}H, 500 MHz, Dimethyl Sulfoxide-d_6, Consistent - Attached
Mass Spectrum	ESI, *m/z* 362 [M + H]$^+$, Attached
UPLC	98.6% (area %), ACQUITY UPLC BEH C18 (2.1 *75) mm, 1.7 micron Column, UV 254 nm Detection, Attached

Approved By: [signature]

Date: 9-27-2023

For Research Purposes Only. Not Intended for Food or Drug Use.

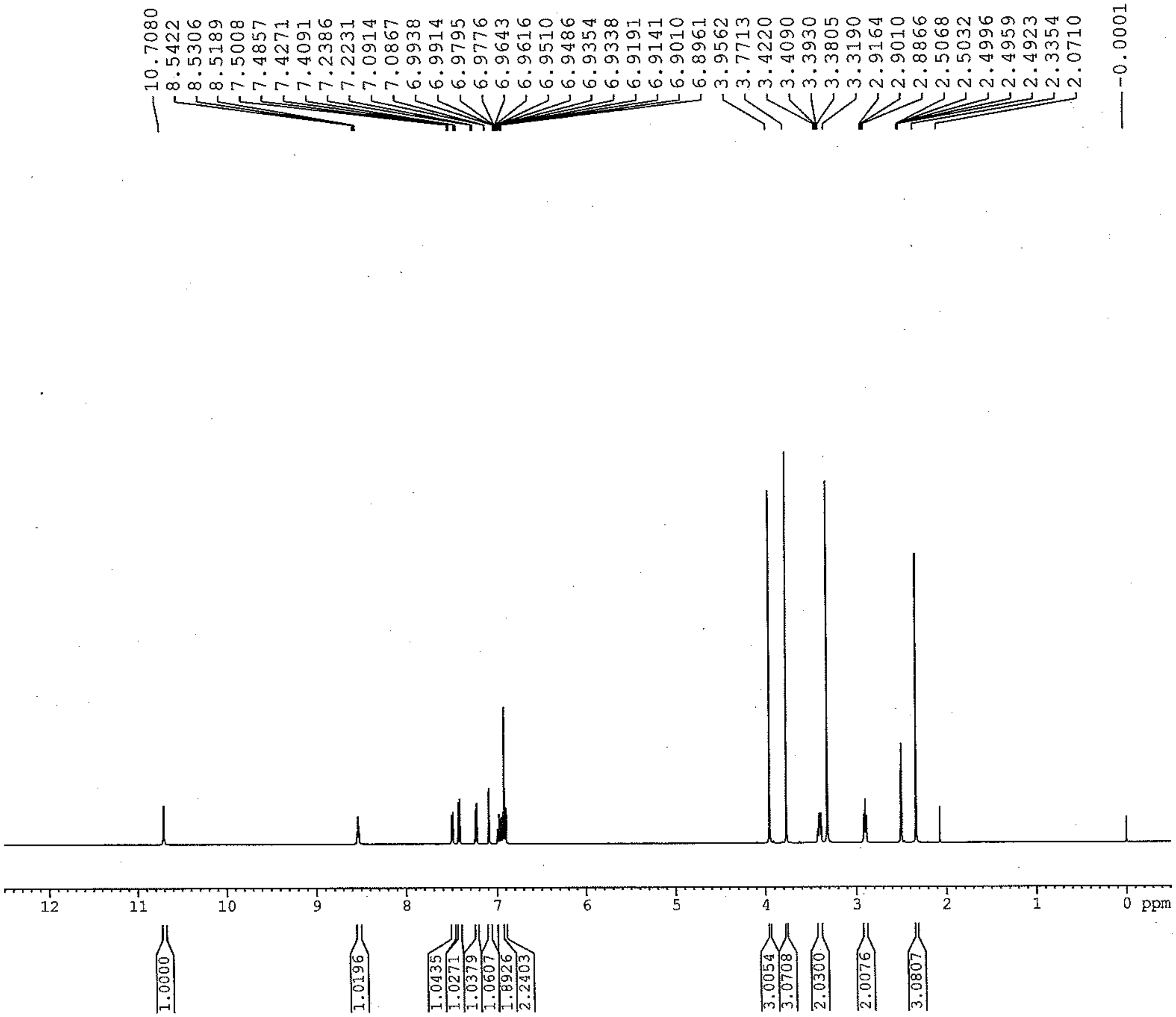

Name Marwah Albaker
Date 21-Sep-2023
NB # ALK-D-77-3

Current Data Parameters
NAME ALK-D-77-3
EXPNO 20
PROCNO 1

F2 - Acquisition Parameters
Date_ 20230921
Time 10.03 h
INSTRUM Avance Neo
PROBHD Z167419_0029 (
PULPROG zg30
TD 65536
SOLVENT DMSO
NS 16
DS 2
SWH 10000.000 Hz
FIDRES 0.305176 Hz
AQ 3.2767999 sec
RG 101
DW 50.000 usec
DE 11.14 usec
TE 300.0 K
D1 1.00000000 sec
TD0 1
SFO1 500.1330883 MHz
NUC1 1H
P0 2.67 usec
P1 8.00 usec
PLW1 24.22400093 W

F2 - Processing parameters
SI 65536
SF 500.1300045 MHz
WDW EM
SSB 0
LB 0.30 Hz
GB 0
PC 1.00

ACQ-SQD#F07SQD100W

20-Sep-2023
11:37:33

Name: Marwah Albaker
Date: 20-Sep-2023
Notebook: ALK-D-77-3

ALK-D-77-3 906 (1.842)

1: Scan ES+
2.75e6

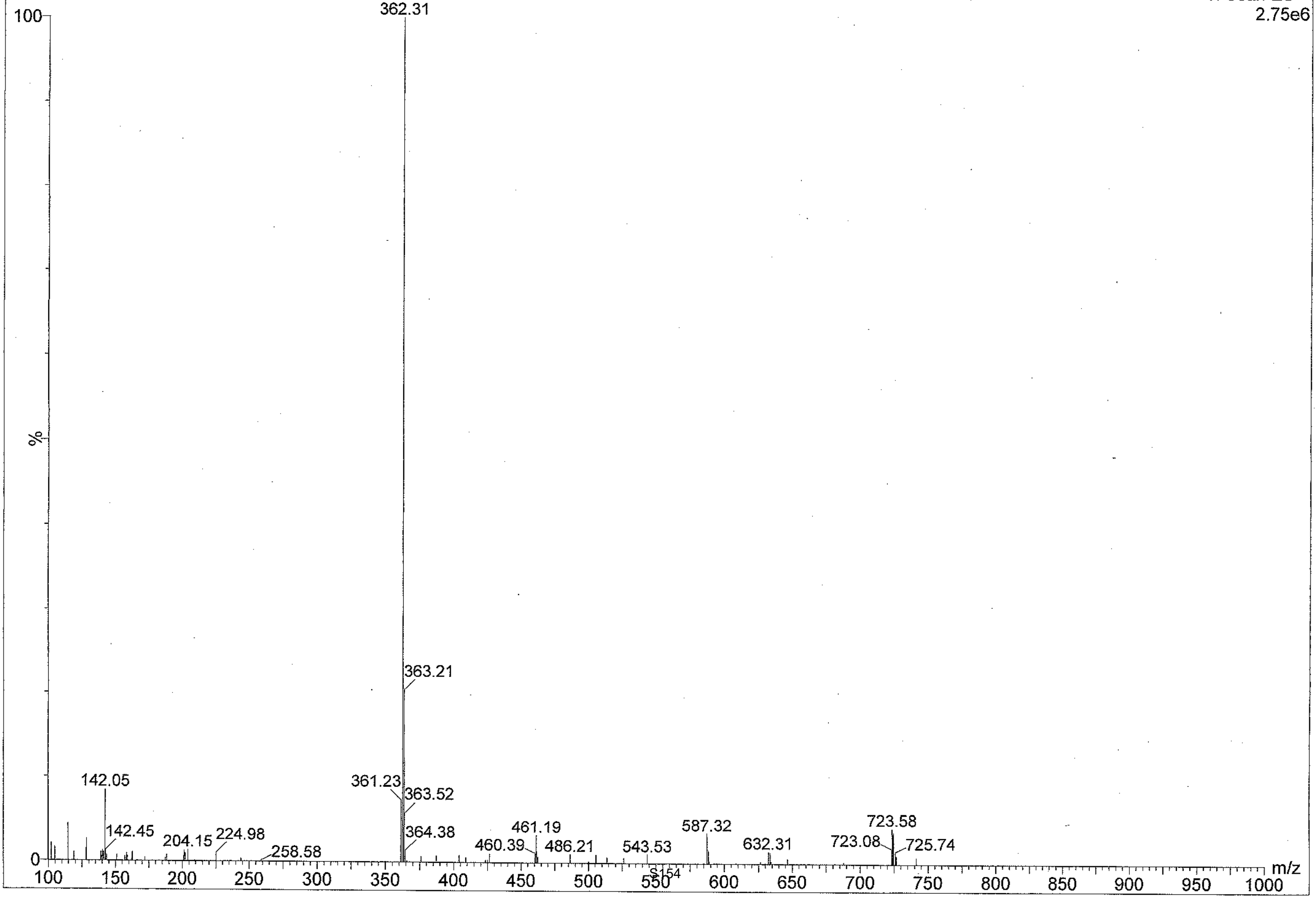

SAMPLE INFORMATION

Sample Name:	ALK-D-77-3
Injection Volume:	3.00 ul
Run Time:	9.0 Minutes
Date Acquired:	9/20/2023 10:48:26 AM EDT
Date Processed:	9/20/2023 11:30:40 AM EDT
Sample Set Name:	Template
Acq. Method Set:	BEH_C18_PDA_75mm
Processing Method:	BEH_C18_PDA
Channel Name:	254nm

Method Notes:
Acquity UPLC BEH C18 1.7u (2.1x75mm)
Flow Rate : 0.5 mL/min
Solvent A : 0.1% TFA in Waters
Solvent B : 0.1% TFA in Acetonitrile
Solvent Gradient Program:

Time (min)	%A	% B
0:00	95	5
6:00	0	100
8:00	0	100
9:00	95	5

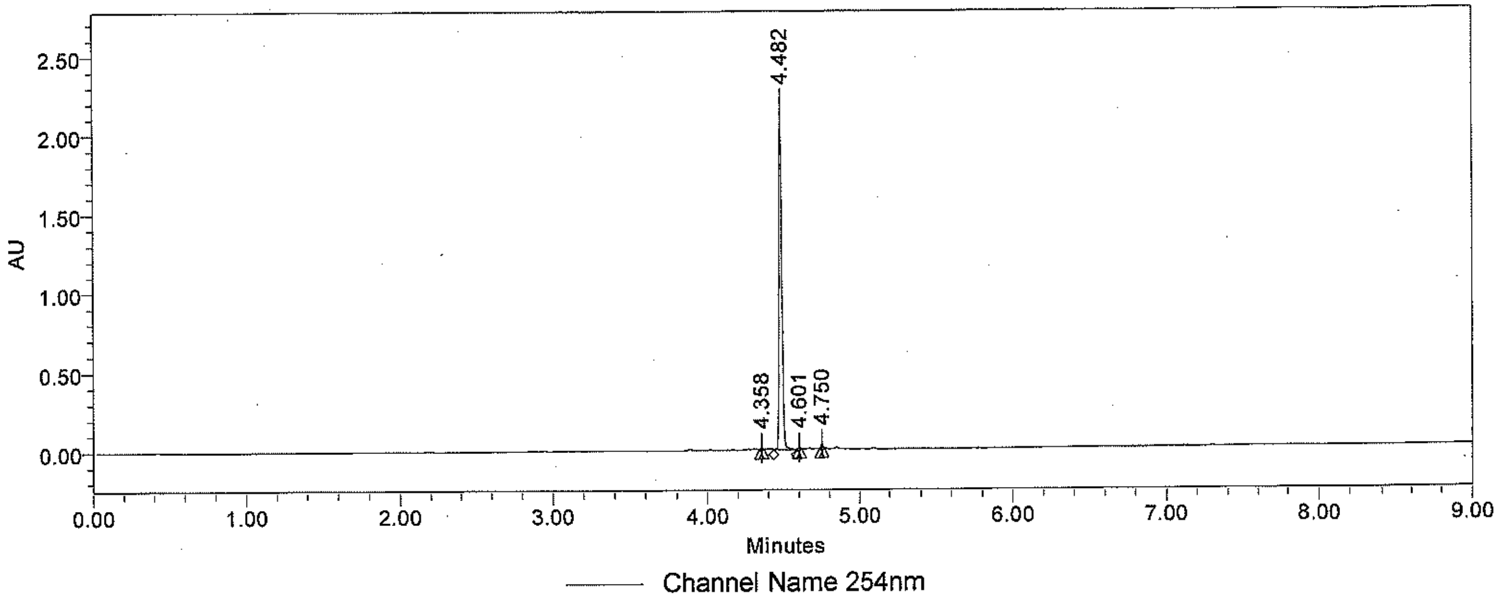

Peak Results

	RT	Area	Int Type	Width (sec)	% Area
1	4.358	8168	bb	1.800	0.28
2	4.482	2835857	VV	8.702	98.66
3	4.601	8036	bb	1.750	0.28
4	4.750	22246	bb	1.800	0.77

Name: Marwah Albaker

Date: 20-Sep-2023

NB #: ALK-D-77-3

CERTIFICATE OF ANALYSIS

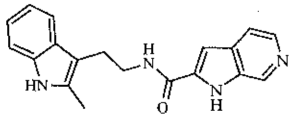

2c. BPN- 36792

Compound Name: BPN-0036792-AA-001
ALB Number: ALB-234256
Batch: I
Lot Number: QUA-B-168-1
Molecular Formula: $C_{19}H_{18}N_4O$
Molecular Weight: 318.37
Last Solvent: Acetonitrile, Water

TEST	RESULT/REFERENCE
Appearance	Yellow Solid
NMR Spectrum	^{1}H, 500 MHz, Dimethyl Sulfoxide-d_6, Consistent - Attached
Mass Spectrum	ESI, m/z 319 $[M + H]^+$, Attached
UPLC	95.2% (area %), ACQUITY UPLC BEH C18 (2.1 *75) mm, 1.7 micron Column, UV 254 nm Detection, Attached

[signature]

Approved By

4-19-2023

Date

For Research Purposes Only. Not Intended for Food or Drug Use.

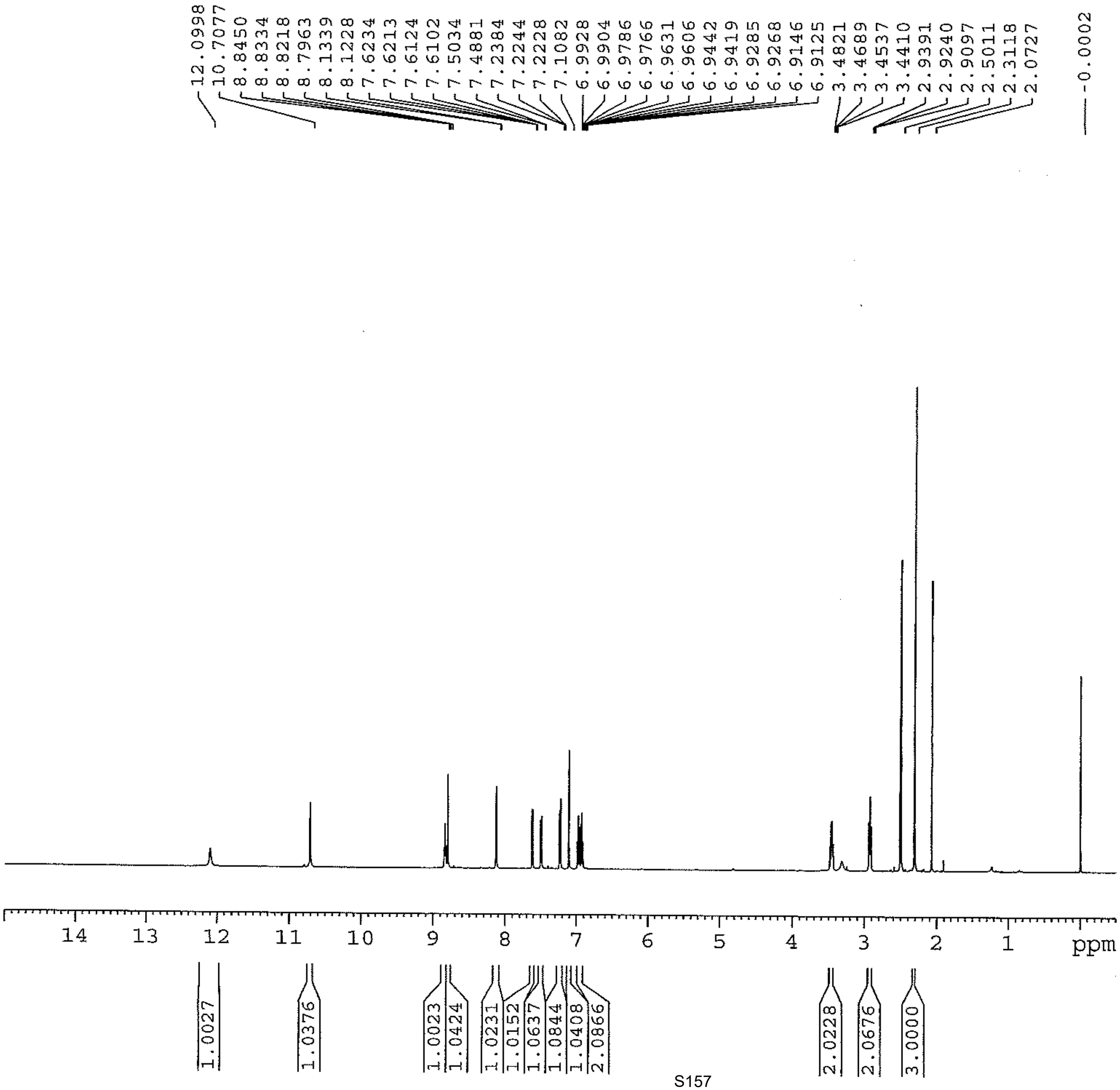

Name Tasdique Quadery
Date 04.14.23
NB # QUA-B-168-1

Current Data Parameters
NAME QUA-B-168-1
EXPNO 30
PROCNO 1

F2 - Acquisition Parameters
Date_ 20230414
Time 10.34 h
INSTRUM Avance Neo
PROBHD Z167419_0029 (
PULPROG zg30
TD 65536
SOLVENT DMSO
NS 32
DS 2
SWH 10000.000 Hz
FIDRES 0.305176 Hz
AQ 3.2767999 sec
RG 101
DW 50.000 usec
DE 11.14 usec
TE 300.0 K
D1 1.00000000 sec
TD0 1
SFO1 500.1330883 MHz
NUC1 1H
P0 2.67 usec
P1 8.00 usec
PLW1 24.22400093 W

F2 - Processing parameters
SI 65536
SF 500.1300038 MHz
WDW EM
SSB 0
LB 0.30 Hz
GB 0
PC 1.00

Openlynx Report

Vial:1:26
Date:11-Apr-2023

ID:
Time:14:18:23

File:QUA-B-168-1

Name: Tasdique Quadery

Date: 04.11.23

Notebook: QUA-B-168-1

Printed: Tue Apr 11 14:19:52 2023

1:(Time: 0.09) Combine (2:5)

1:MS ES+
4.6e+007

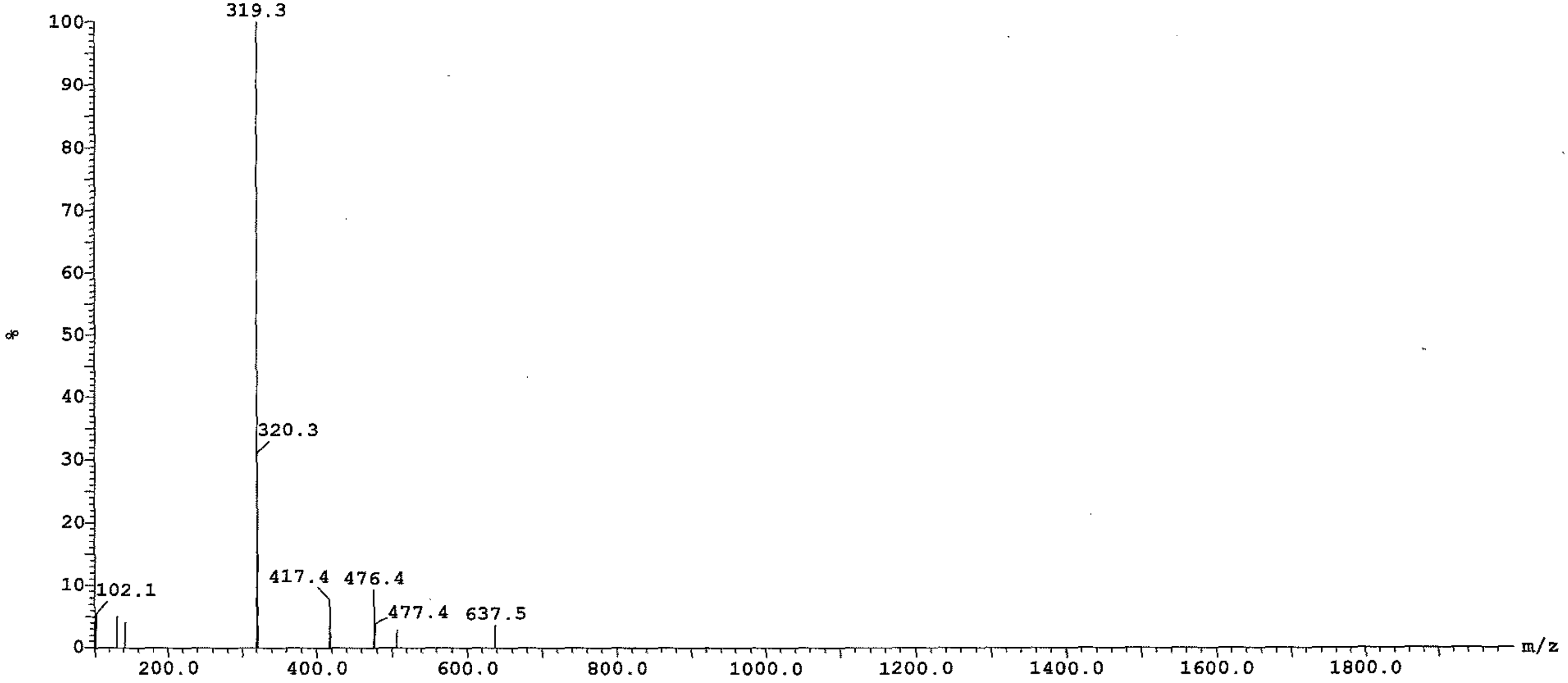

SAMPLE INFORMATION

Sample Name: QUA-B-168-1
Injection Volume: 3.00 ul
Run Time: 9.0 Minutes
Date Acquired: 4/11/2023 2:17:53 PM EDT
Date Processed: 4/11/2023 2:30:56 PM EDT
Sample Set Name: Template
Acq. Method Set: BEH_C18_PDA_75mm
Processing Method: BEH_C18_PDA
Channel Name: 254nm

Method Notes:
Acquity UPLC BEH C18 1.7u (2.1x75mm)
Flow Rate : 0.5 mL/min
Solvent A : 0.1% TFA in Waters
Solvent B : 0.1% TFA in Acetonitrile
Solvent Gradient Program:

Time (min)	%A	% B
0:00	95	5
6:00	0	100
8:00	0	100
9:00	95	5

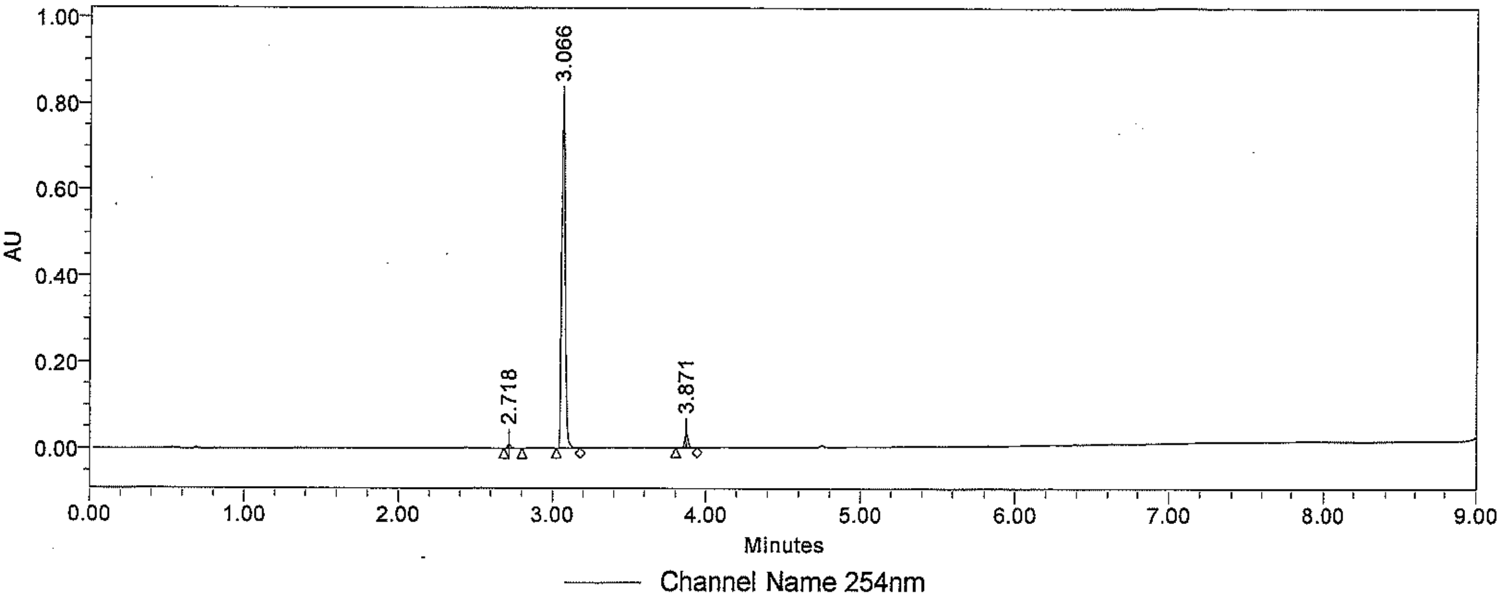

Peak Results

	RT	Area	Int Type	Width (sec)	% Area
1	2.718	15117	BB	7.099	1.13
2	3.066	1273488	BV	9.149	95.20
3	3.871	49144	BV	8.299	3.67

Name: Tasdique Quadery

Date: 04.11.23

NB #: QUA-B-168-1

CERTIFICATE OF ANALYSIS

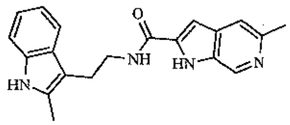

2d.BPN- 36958

Compound Name: BPN-0036958-AA-001
ALB Number: ALB-235132
Batch: 1
Lot Number: ALK-C-201-2
Molecular Formula: $C_{20}H_{20}N_4O$
Molecular Weight: 332.40
Last Solvent: Water, Acetonitrile

TEST	RESULT/REFERENCE
Appearance	Yellow Solid
NMR Spectrum	^{1}H, 500 MHz, Dimethyl Sulfoxide-d_6, Consistent - Attached
Mass Spectrum	ESI, *m/z* 333 $[M + H]^+$, Attached
UPLC	95.5% (area %), ACQUITY UPLC BEH C18 (2.1 *75) mm, 1.7 micron Column, UV 254 nm Detection, Attached

MariaDMaychack

Approved By

6-7-2023

Date

For Research Purposes Only. Not Intended for Food or Drug Use.

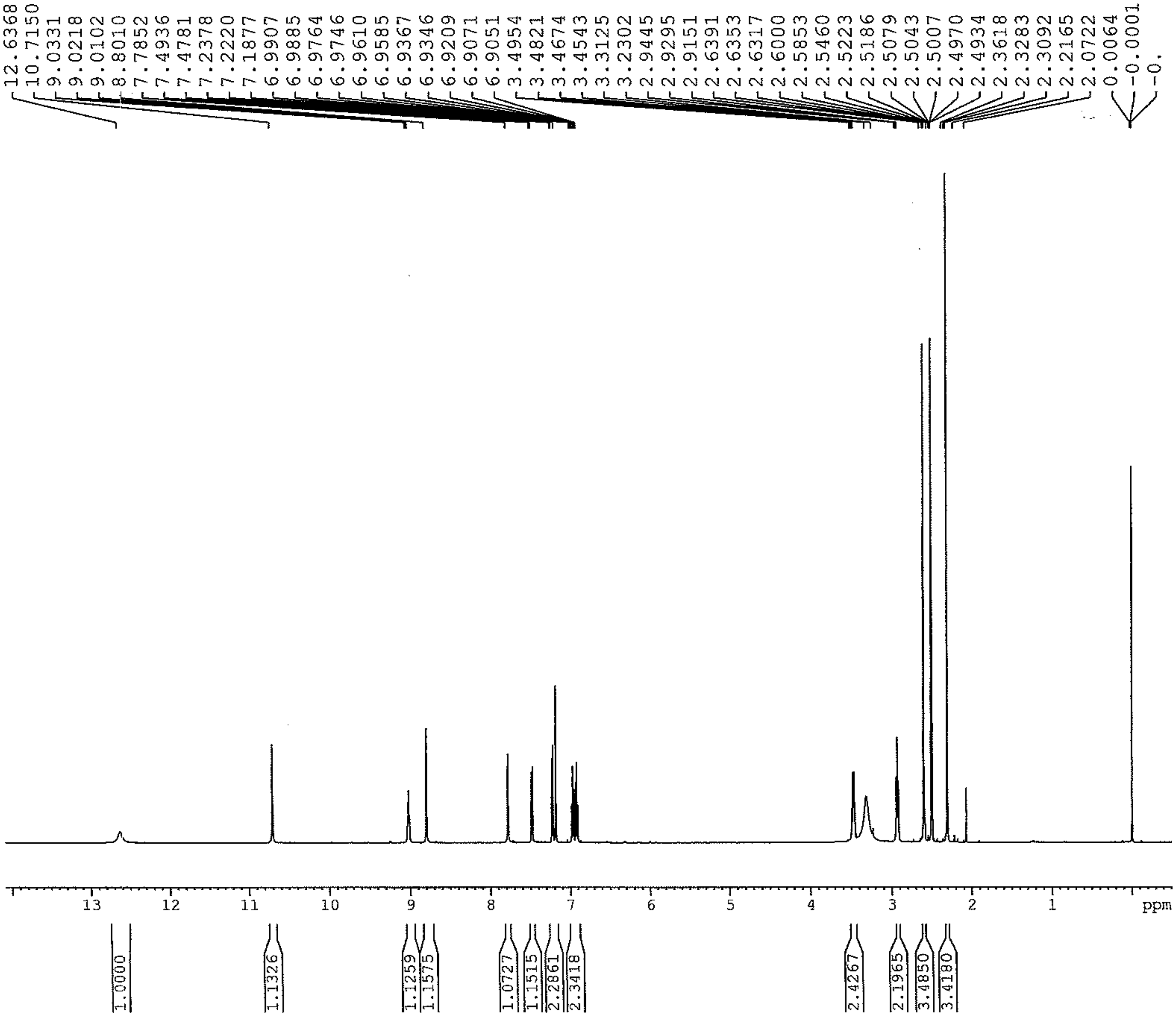

Name Marwah Albaker

Date 31-May-2023

NB # ALK-C-201-2

```
Current Data Parameters
NAME          ALK-C-201-2
EXPNO                  10
PROCNO                  1

F2 - Acquisition Parameters
Date_           20230531
Time                8.13 h
INSTRUM        Avance Neo
PROBHD   Z167419_0029 (
PULPROG              zg30
TD                  65536
SOLVENT              DMSO
NS                     16
DS                      2
SWH            10000.000 Hz
FIDRES          0.305176 Hz
AQ             3.2767999 sec
RG                    101
DW                 50.000 usec
DE                  11.14 usec
TE                  300.0 K
D1            1.00000000 sec
TD0                     1
SFO1         500.1330883 MHz
NUC1                   1H
P0                   2.67 usec
P1                   8.00 usec
PLW1          24.22400093 W

F2 - Processing parameters
SI                  65536
SF           500.1300039 MHz
WDW                    EM
SSB                     0
LB                   0.30 Hz
GB                      0
PC                   1.00
```

ACQ-SQD#F07SQD100W

26-May-2023
08:57:46

Name: Marwan Albaker
Date: 26-May-2023
Notebook: ALK-C-201-2

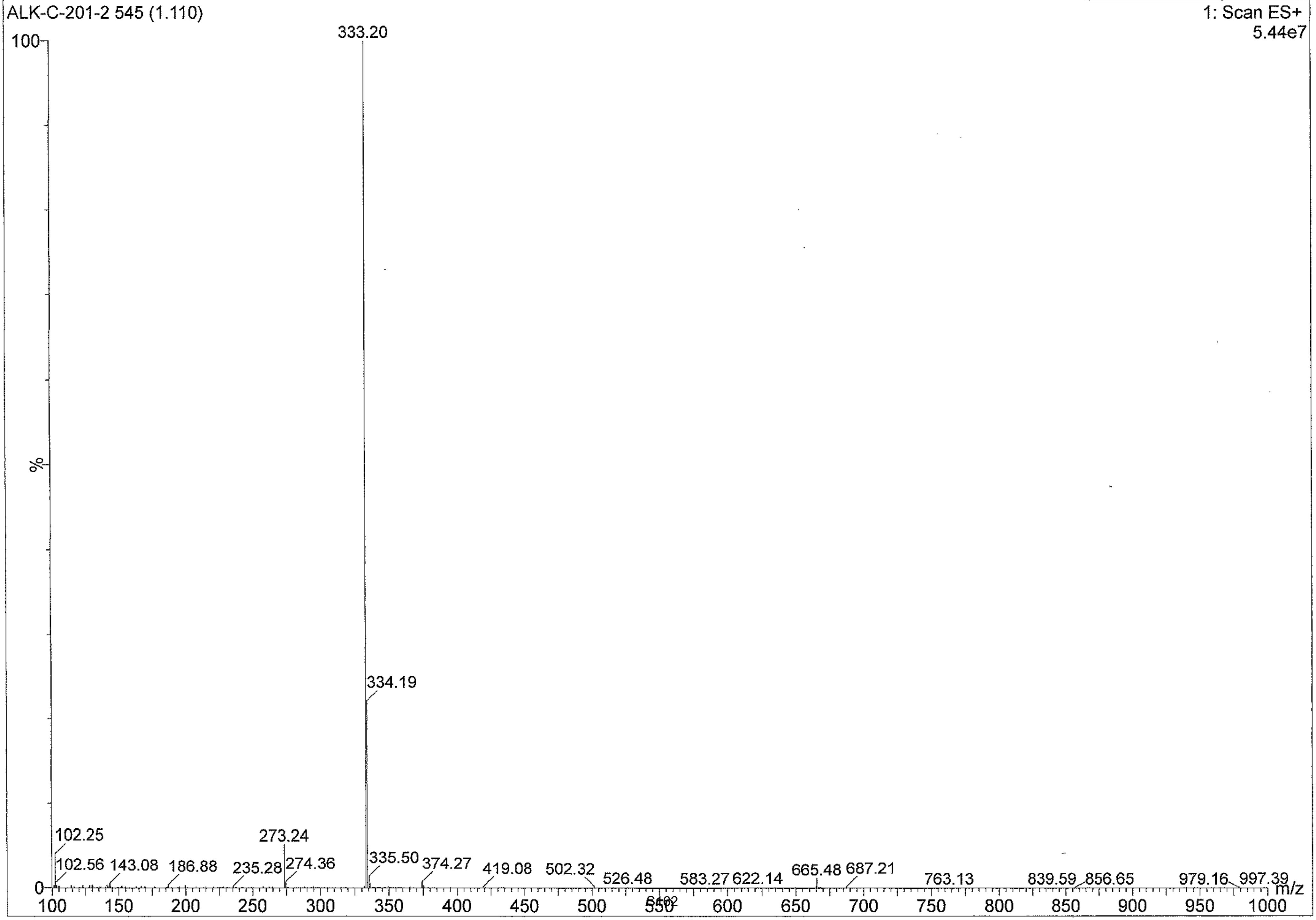

SAMPLE INFORMATION

Sample Name:	ALK-C-201-2
Injection Volume:	3.00 ul
Run Time:	9.0 Minutes
Date Acquired:	5/26/2023 9:44:28 AM EDT
Date Processed:	5/26/2023 9:58:34 AM EDT
Sample Set Name:	Template
Acq. Method Set:	BEH_C18_PDA_75mm
Processing Method:	BEH_C18_PDA
Channel Name:	254nm

Method Notes:
Acquity UPLC BEH C18 1.7u (2.1x75mm)
Flow Rate : 0.5 mL/min
Solvent A : 0.1% TFA in Waters
Solvent B : 0.1% TFA in Acetonitrile
Solvent Gradient Program:

Time (min)	%A	% B
0:00	95	5
6:00	0	100
8:00	0	100
9:00	95	5

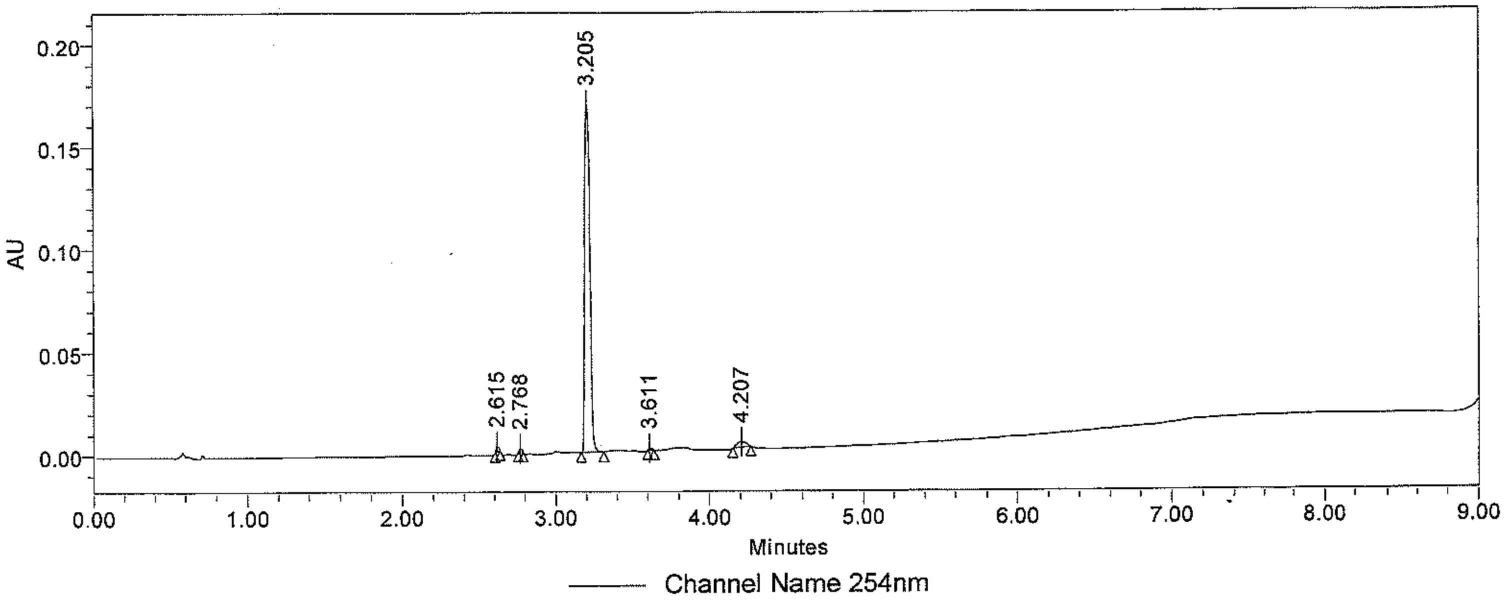

Peak Results

	RT	Area	Int Type	Width (sec)	% Area
1	2.615	3213	bb	2.000	0.80
2	2.768	2348	bb	2.000	0.58
3	3.205	383602	BB	8.849	95.55
4	3.611	1471	bb	2.450	0.37
5	4.207	10828	bb	6.901	2.70

Name: Marwah Albaker

Date: 26-May-2023

NB #: ALK-C-201-2

CERTIFICATE OF ANALYSIS

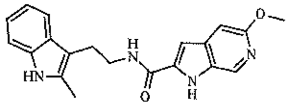

2e.BPN- 36793

Compound Name: BPN-0036793-AA-001
ALB Number: ALB-234258
Batch: 1
Lot Number: QUA-B-170-2
Molecular Formula: $C_{20}H_{20}N_4O_2$
Molecular Weight: 348.40
Last Solvent: Acetonitrile, Water

TEST	RESULT/REFERENCE
Appearance	Off-white Solid
NMR Spectrum	^{1}H, 500 MHz, Dimethyl Sulfoxide-d_6, Consistent - Attached
Mass Spectrum	ESI, *m/z* 349 $[M + H]^+$, Attached
UPLC	>99% (area %), ACQUITY UPLC BEH C18 (2.1 *75) mm, 1.7 micron Column, UV 254 nm Detection, Attached

Approved By

4-19-2023
Date

For Research Purposes Only. Not Intended for Food or Drug Use.

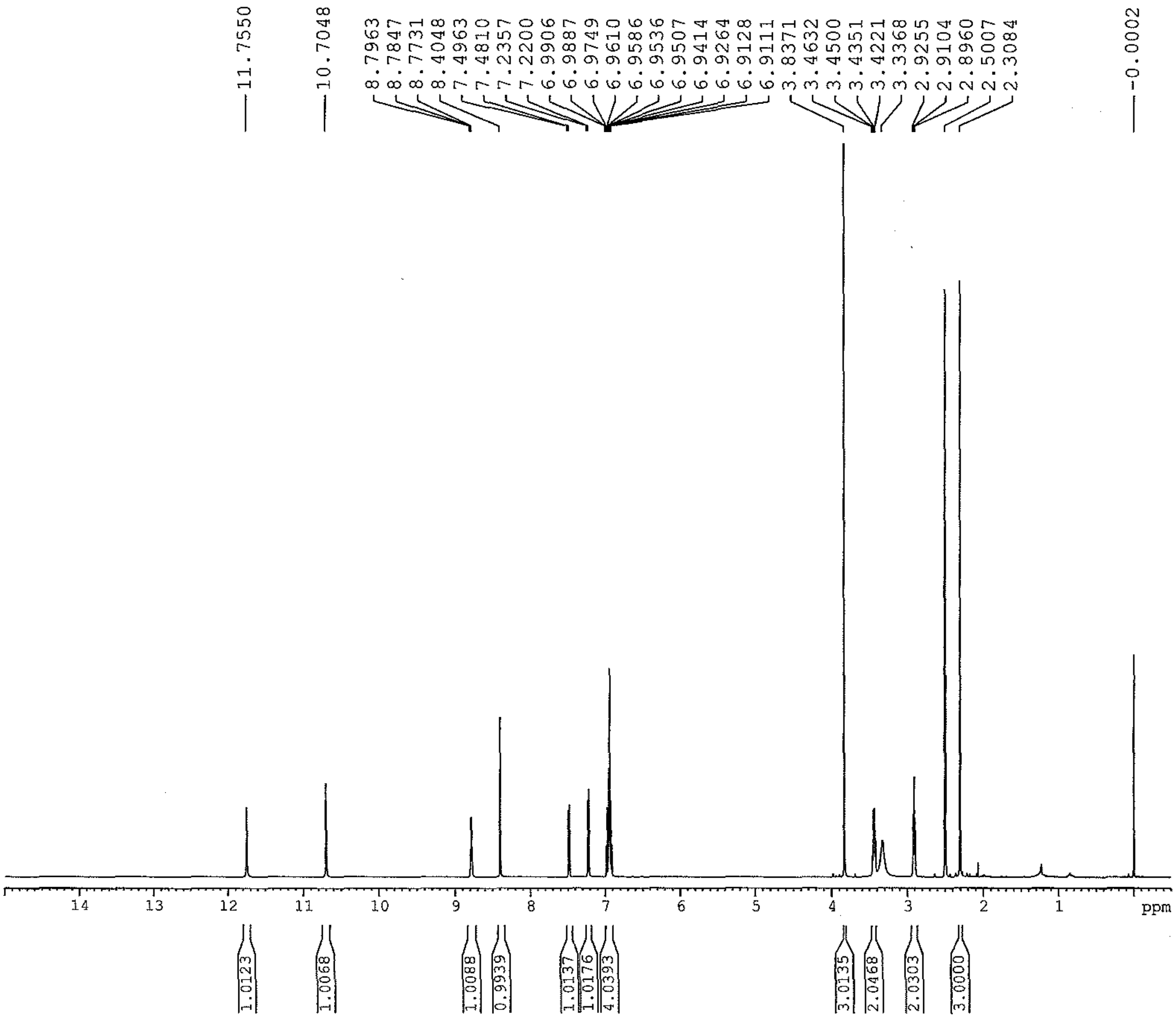

Name Tasdique Quadery

Date 04.14.23

NB # QUA-B-170-2

```
Current Data Parameters
NAME          QUA-B-170-2
EXPNO                  10
PROCNO                  1

F2 - Acquisition Parameters
Date_            20230414
Time                12.50 h
INSTRUM         Avance Neo
PROBHD   Z167419_0029 (
PULPROG              zg30
TD                  65536
SOLVENT              DMSO
NS                     32
DS                      2
SWH             10000.000 Hz
FIDRES           0.305176 Hz
AQ              3.2767999 sec
RG                    101
DW                 50.000 usec
DE                  11.14 usec
TE                  300.0 K
D1             1.00000000 sec
TD0                     1
SFO1          500.1330883 MHz
NUC1                   1H
P0                   2.67 usec
P1                   8.00 usec
PLW1          24.22400093 W

F2 - Processing parameters
SI                  65536
SF            500.1300039 MHz
WDW                    EM
SSB                     0
LB                   0.30 Hz
GB                      0
PC                   1.00
```

Openlynx Report

Vial:2:8
Date:13-Apr-2023

ID:
Time:12:10:37

File:QUA-B-170-2

Name: Tasdique Quadery

Date: 04-13-23

Notebook: QUA-B-170-2

Printed: Thu Apr 13 12:12:52 2023

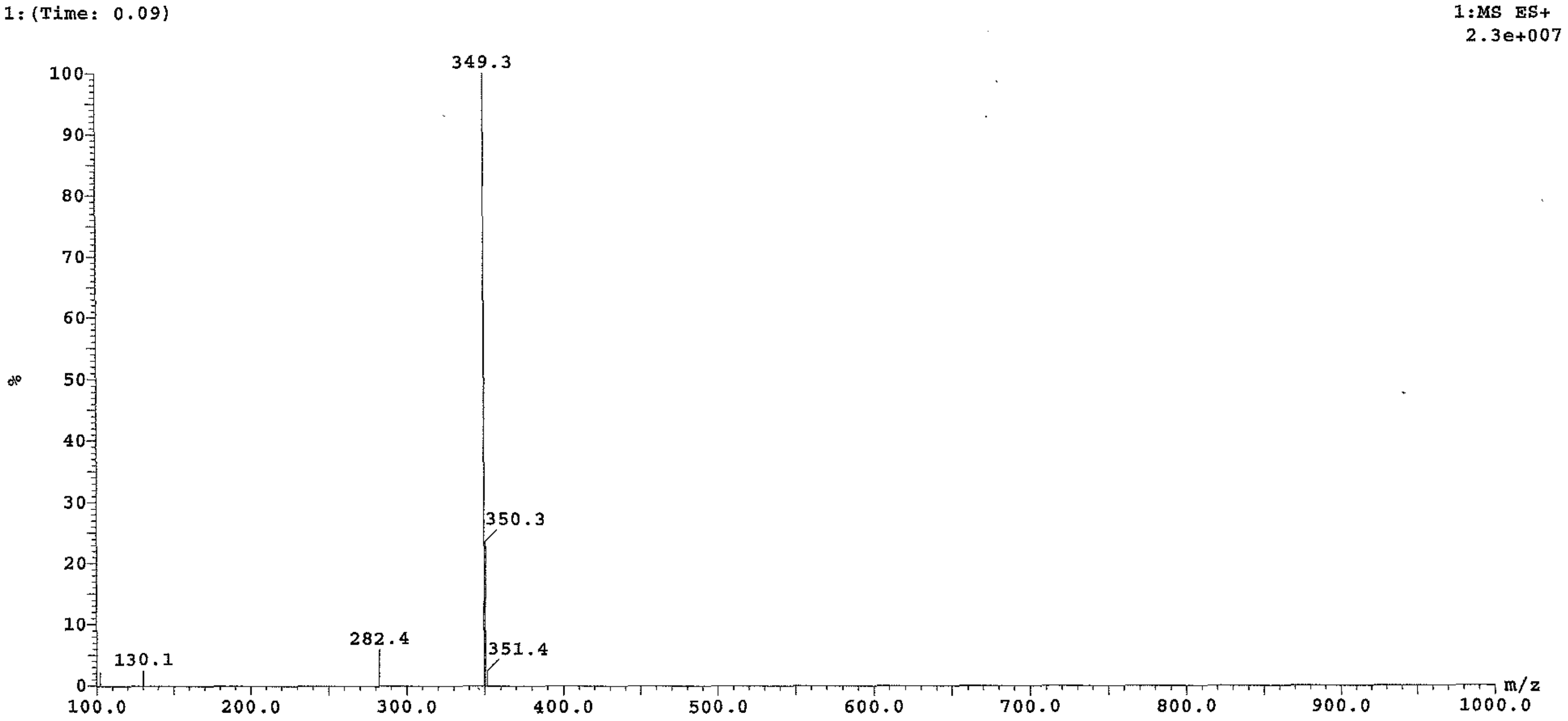

SAMPLE INFORMATION

Sample Name:	QUA-B-170-2
Injection Volume:	3.00 ul
Run Time:	9.0 Minutes
Date Acquired:	4/13/2023 11:06:54 AM EDT
Date Processed:	4/13/2023 11:18:56 AM EDT
Sample Set Name:	Template
Acq. Method Set:	BEH_C18_PDA_75mm
Processing Method:	BEH_C18_PDA
Channel Name:	254nm

Method Notes:
Acquity UPLC BEH C18 1.7u (2.1x75mm)
Flow Rate : 0.5 mL/min
Solvent A : 0.1% TFA in Waters
Solvent B : 0.1% TFA in Acetonitrile
Solvent Gradient Program:

Time (min)	%A	% B
0:00	95	5
6:00	0	100
8:00	0	100
9:00	95	5

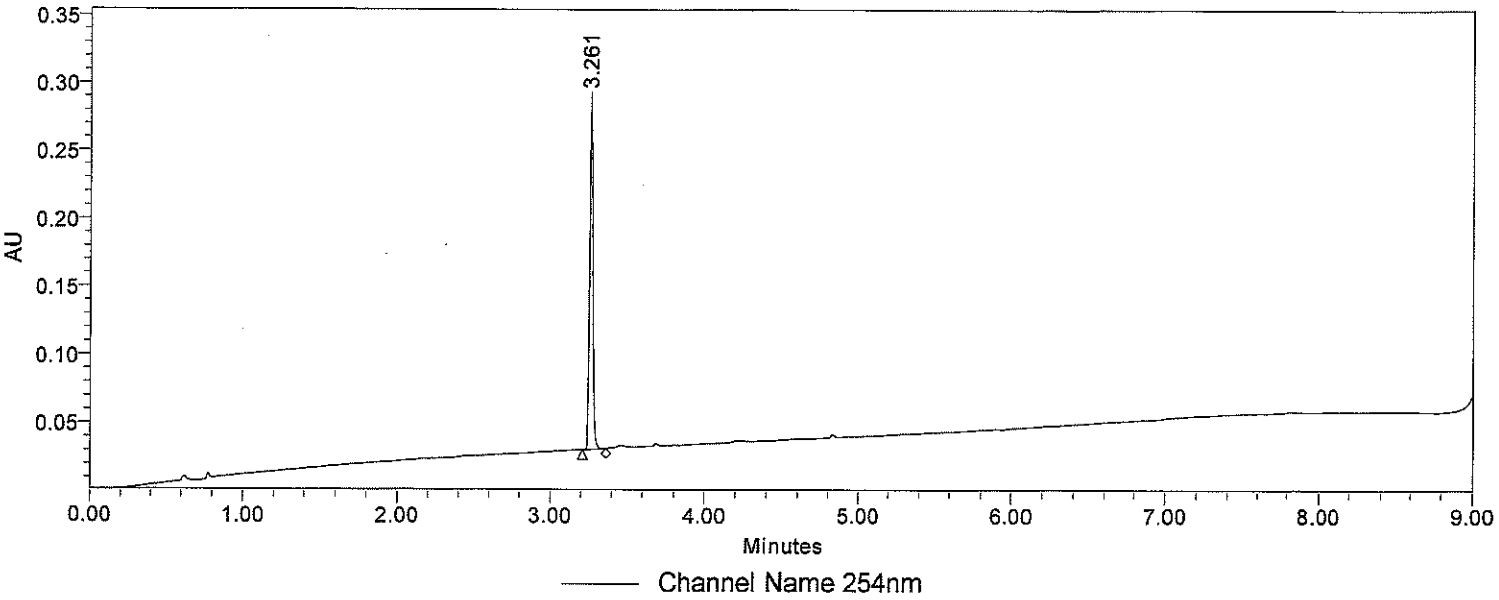

Peak Results

	RT	Area	Int Type	Width (sec)	% Area
1	3.261	369839	BV	9.149	100.00

Name: Tasdique Quodery

Date: 04.13.23

NB #: QUA-B-170-2

CERTIFICATE OF ANALYSIS

Compound Name: BPN-0037092-AA-001
ALB Number: ALB-235888
Batch: 1
Lot Number: ALK-D-24-2
Molecular Formula: $C_{20}H_{20}N_4O$
Molecular Weight: 332.40
Last Solvent: Water, Acetonitrile

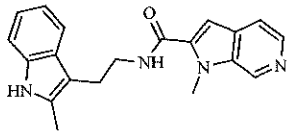

2f.BPN- 37092

TEST	RESULT/REFERENCE
Appearance	Off-white Solid
NMR Spectrum	^{1}H, 500 MHz, Dimethyl Sulfoxide-d_6, Consistent - Attached
Mass Spectrum	ESI, m/z 333 $[M + H]^+$, Attached
UPLC	98.0% (area %), ACQUITY UPLC BEH C18 (2.1 *75) mm, 1.7 micron Column, UV 254 nm Detection, Attached

Maria Mayshack
Approved By

7-26-2023
Date

For Research Purposes Only. Not Intended for Food or Drug Use.

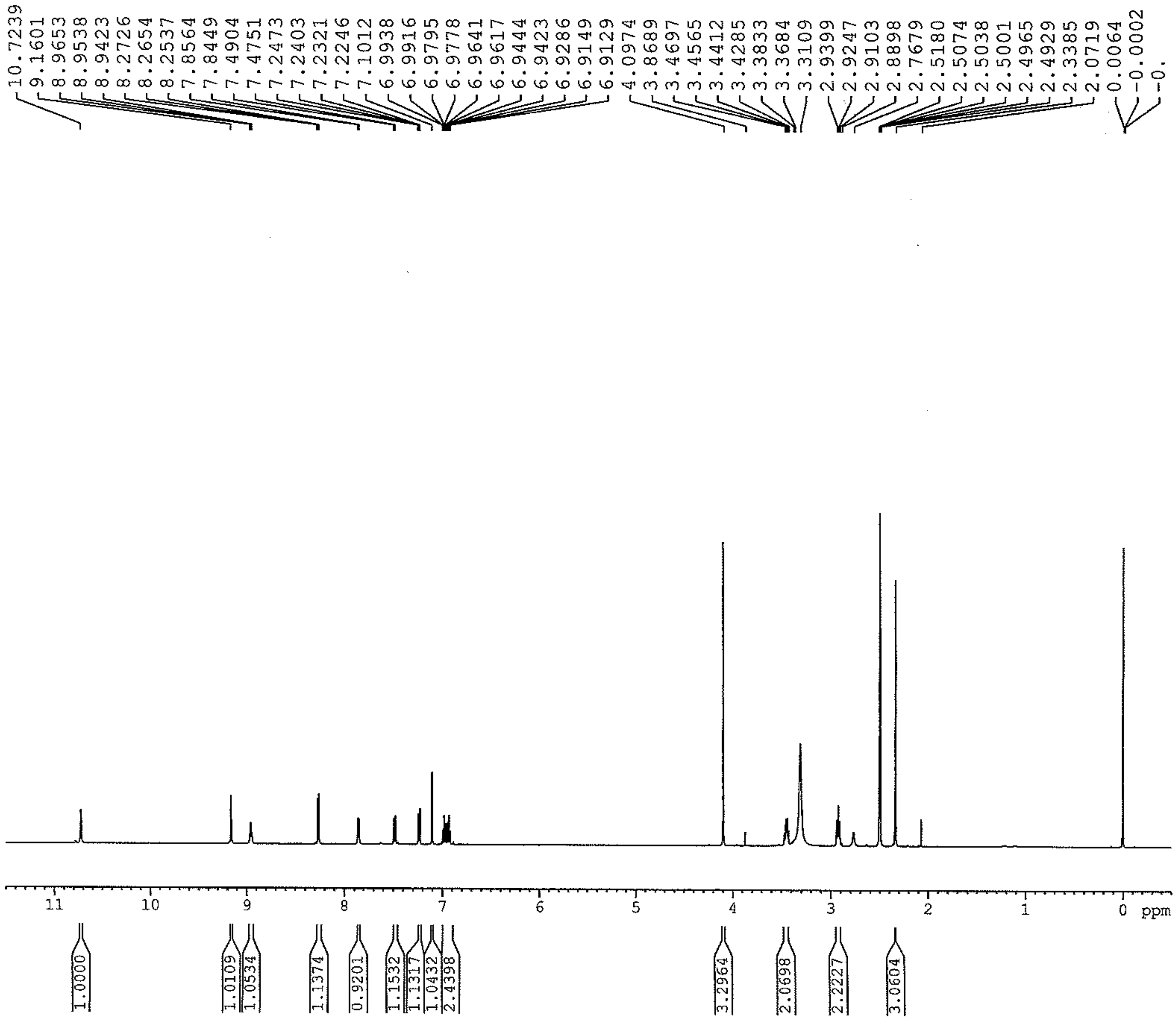

Name Marwah Albaker
Date 20-Jul-2023
NB # ALK-D-24-2

Current Data Parameters
NAME ALK-D-24-2
EXPNO 20
PROCNO 1

F2 - Acquisition Parameters
Date_ 20230720
Time 7.41 h
INSTRUM Avance Neo
PROBHD Z167419_0029 (
PULPROG zg30
TD 65536
SOLVENT DMSO
NS 64
DS 2
SWH 10000.000 Hz
FIDRES 0.305176 Hz
AQ 3.2767999 sec
RG 101
DW 50.000 usec
DE 11.14 usec
TE 300.0 K
D1 1.00000000 sec
TD0 1
SFO1 500.1330883 MHz
NUC1 1H
P0 2.67 usec
P1 8.00 usec
PLW1 24.22400093 W

F2 - Processing parameters
SI 65536
SF 500.1300042 MHz
WDW EM
SSB 0
LB 0.30 Hz
GB 0
PC 1.00

ACQ-SQD#F07SQD100W

18-Jul-2023
11:34:01

Name: Marwah Albakker
Date: 24-Jul-2023
Notebook: ALK-D-24-2

ALK-D-24-2 555 (1.123)

1: Scan ES+
4.94e7

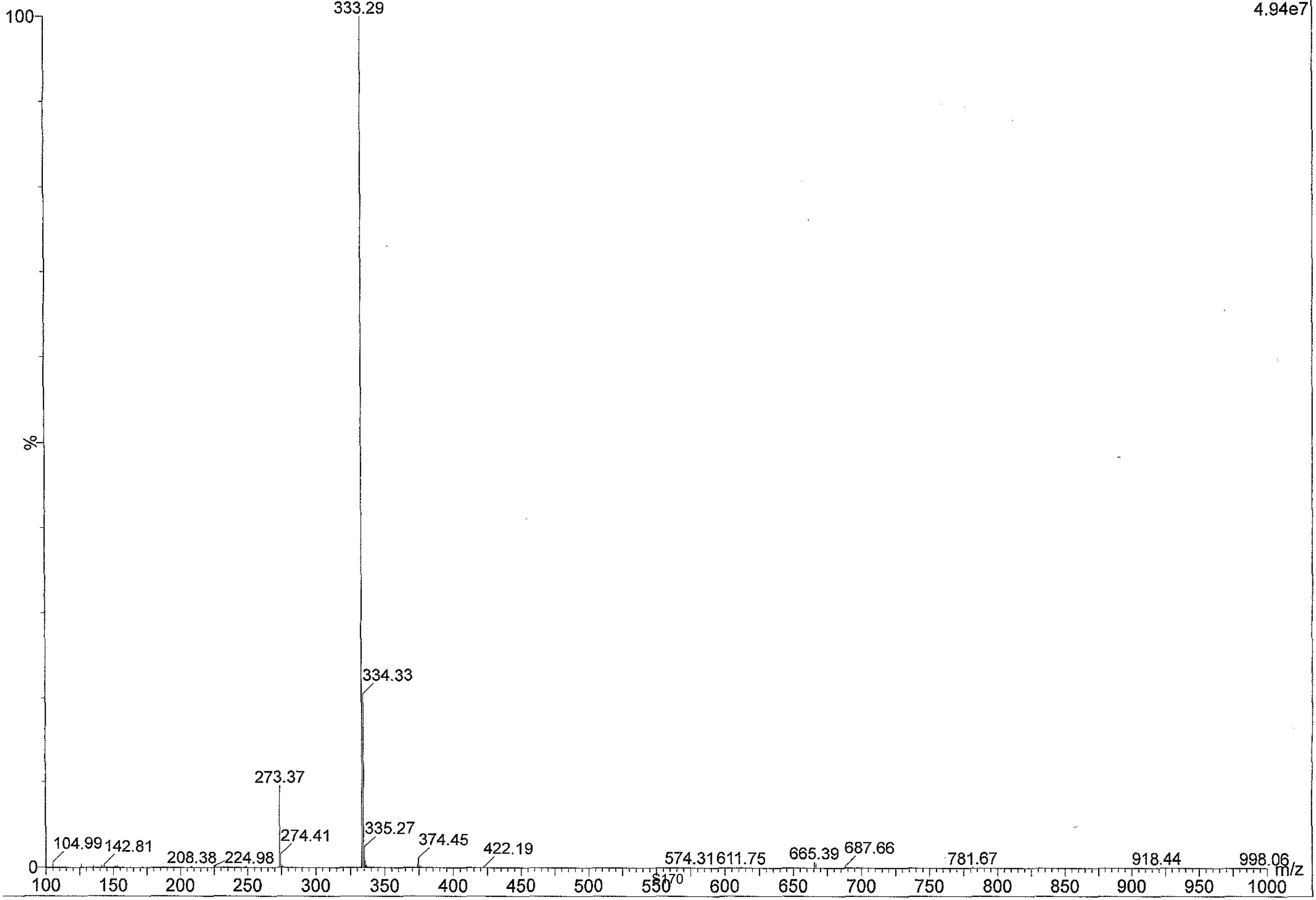

SAMPLE INFORMATION

Sample Name:	ALK-D-24-2
Injection Volume:	3.00 ul
Run Time:	9.0 Minutes
Date Acquired:	7/18/2023 12:19:19 PM EDT
Date Processed:	7/18/2023 12:45:23 PM EDT
Sample Set Name:	Template
Acq. Method Set:	BEH_C18_PDA_75mm
Processing Method:	BEH_C18_PDA
Channel Name:	254nm

Method Notes:
Acquity UPLC BEH C18 1.7u (2.1x75mm)
Flow Rate : 0.5 mL/min
Solvent A : 0.1% TFA in Waters
Solvent B : 0.1% TFA in Acetonitrile
Solvent Gradient Program:

Time (min)	%A	% B
0:00	95	5
6:00	0	100
8:00	0	100
9:00	95	5

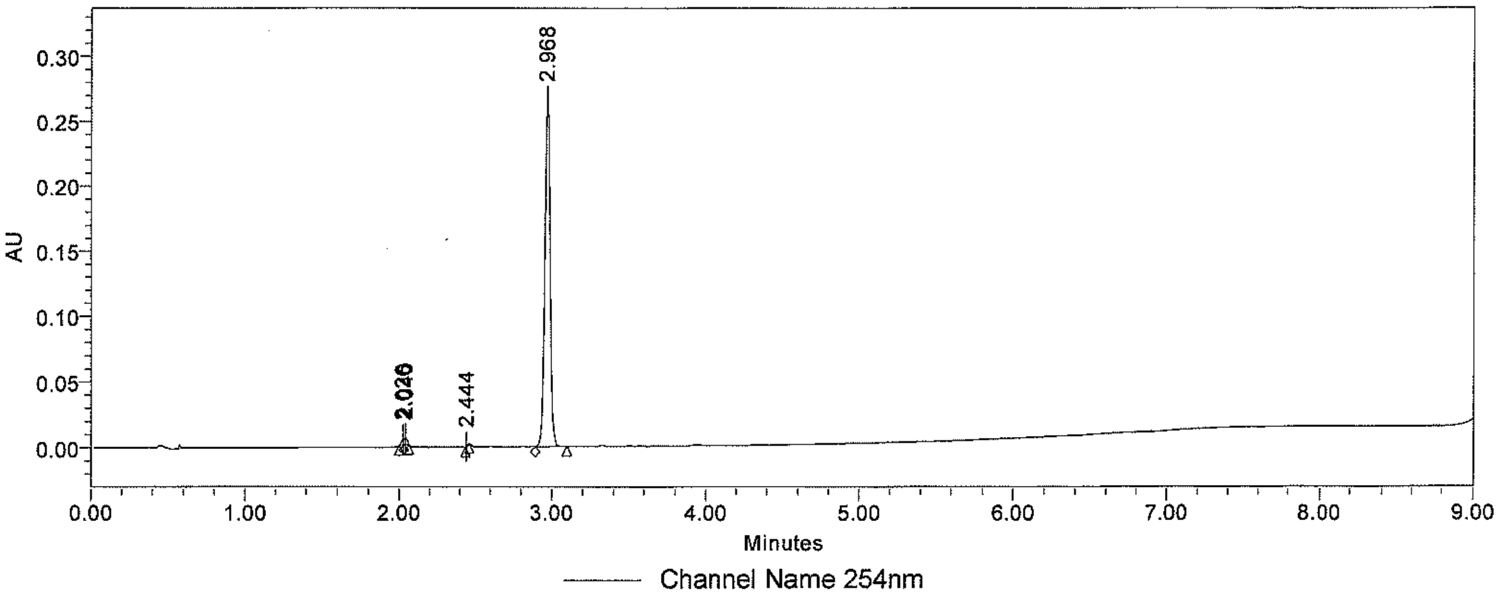

Peak Results

	RT	Area	Int Type	Width (sec)	% Area
1	2.026	4673	bV	1.800	0.74
2	2.040	7482	Vb	2.000	1.18
3	2.444	248	bb	1.450	0.04
4	2.968	620510	VB	12.149	98.04

Name: Marwah Albaker

Date: 18-Jul-2023

NB #: ALK-D-24-2

CERTIFICATE OF ANALYSIS

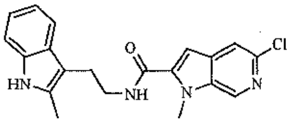

2g. BPN- 37441

Compound Name: BPN-0037441-AA-001
ALB Number: ALB-237283
Batch: 1
Lot Number: ALK-D-99-2
Molecular Formula: $C_{20}H_{19}ClN_4O$
Molecular Weight: 366.84
Last Solvent: Water, Acetonitrile

TEST	RESULT/REFERENCE
Appearance	Off-white Solid
NMR Spectrum	^{1}H, 500 MHz, Dimethyl Sulfoxide-d_6, Consistent - Attached
Mass Spectrum	ESI, m/z 367 $[M + H]^+$, Attached
UPLC	98.9% (area %), ACQUITY UPLC BEH C18 (2.1 *75) mm, 1.7 micron Column, UV 254 nm Detection, Attached

Maura Maychack
Approved By

10-25-2023
Date

For Research Purposes Only. Not Intended for Food or Drug Use.

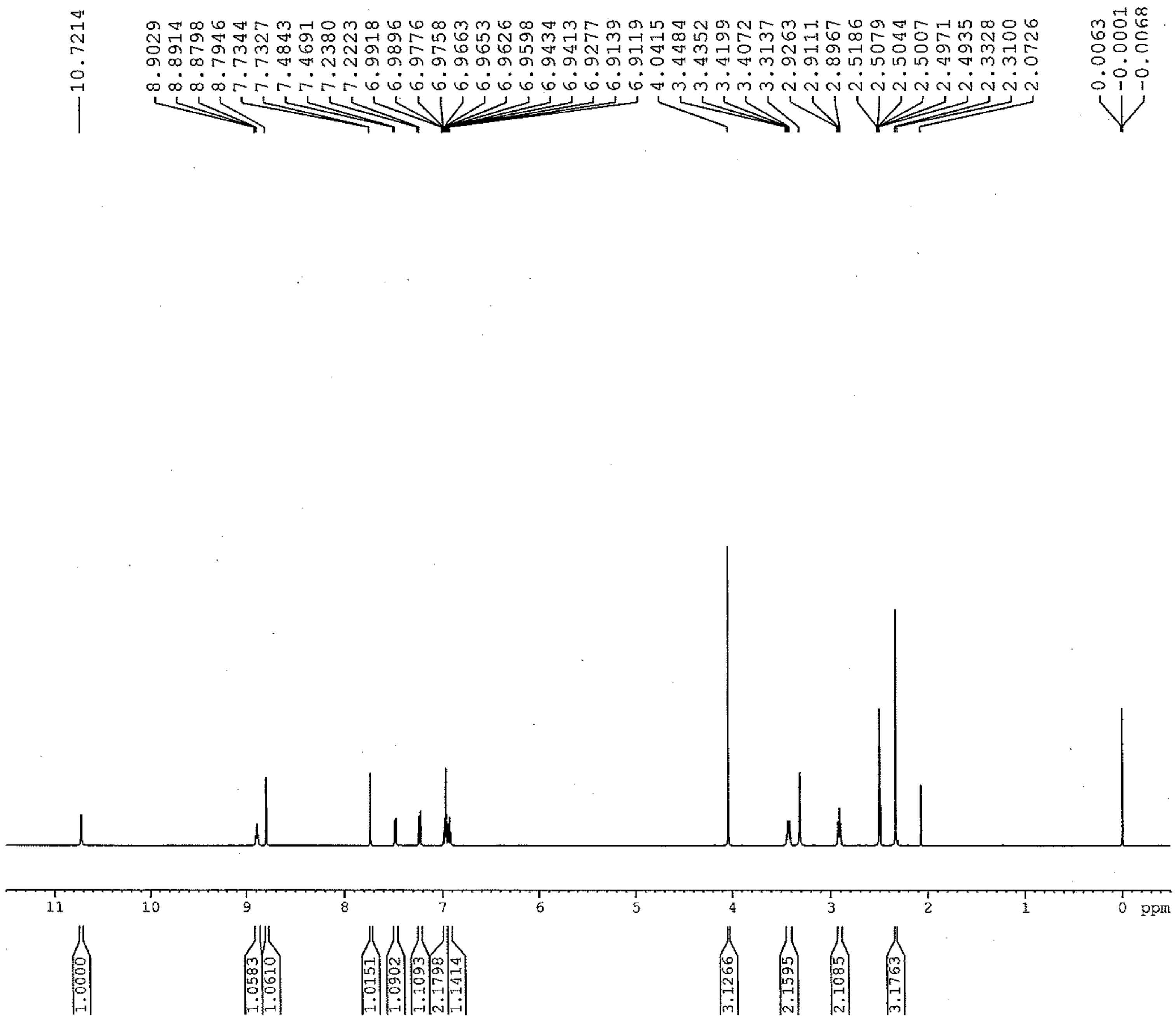

Name Marwah Albaker
Date 19-oct-2023
NB # ALK-D-99-2

Current Data Parameters
NAME ALK-D-99-2
EXPNO 10
PROCNO 1

F2 - Acquisition Parameters
Date_ 20231019
Time 8.23 h
INSTRUM Avance Neo
PROBHD Z167419_0029 (
PULPROG zg30
TD 65536
SOLVENT DMSO
NS 64
DS 2
SWH 10000.000 Hz
FIDRES 0.305176 Hz
AQ 3.2767999 sec
RG 101
DW 50.000 usec
DE 11.14 usec
TE 300.0 K
D1 1.00000000 sec
TD0 1
SFO1 500.1330883 MHz
NUC1 1H
P0 2.67 usec
P1 8.00 usec
PLW1 24.22400093 W

F2 - Processing parameters
SI 65536
SF 500.1300039 MHz
WDW EM
SSB 0
LB 0.30 Hz
GB 0
PC 1.00

ACQ-SQD#F07SQD100W

18-Oct-2023
14:02:24

Name: Marwah Albaker
Date: 19-oct-2023
Notebook: ALK-D-99-2

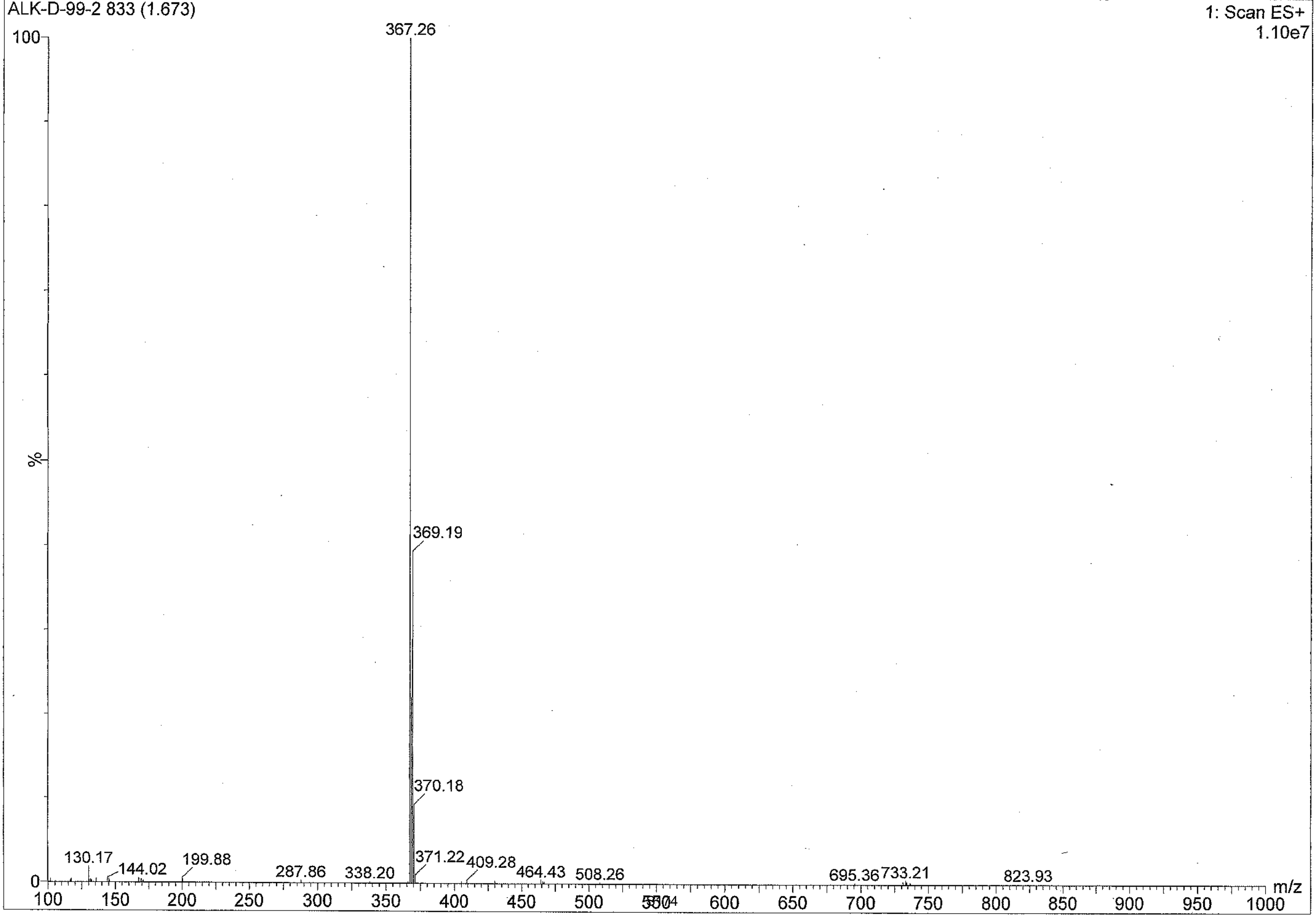

SAMPLE INFORMATION

Sample Name: ALK-D-99-2
Injection Volume: 1.50 ul
Run Time: 9.0 Minutes
Date Acquired: 10/18/2023 2:35:12 PM EDT
Date Processed: 10/19/2023 8:10:57 AM EDT
Sample Set Name: Template
Acq. Method Set: BEH_C18_PDA_75mm
Processing Method: BEH_C18_PDA
Channel Name: 254nm

Method Notes:
Acquity UPLC BEH C18 1.7u (2.1x75mm)
Flow Rate : 0.5 mL/min
Solvent A : 0.1% TFA in Waters
Solvent B : 0.1% TFA in Acetonitrile
Solvent Gradient Program:

Time (min)	%A	% B
0:00	95	5
6:00	0	100
8:00	0	100
9:00	95	5

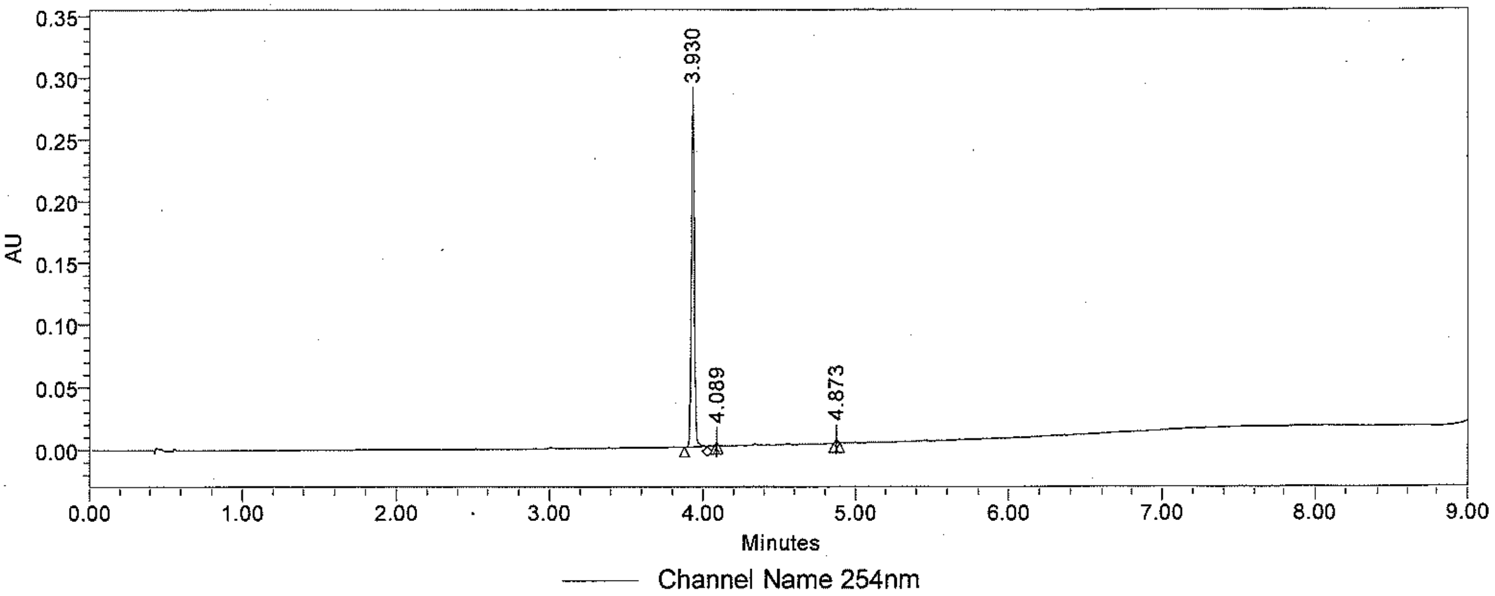

Peak Results

	RT	Area	Int Type	Width (sec)	% Area
1	3.930	384120	BV	8.900	98.95
2	4.089	1122	bb	1.150	0.29
3	4.873	2935	bb	2.351	0.76

Name: Marwah Albaker

Date: 19-Oct-2023

NB #: ALK-D-99-2

CERTIFICATE OF ANALYSIS

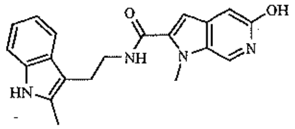

2h.BPN- 37363

Compound Name: BPN-0037363-AA-001
ALB Number: ALB-236970
Batch: 1
Lot Number: JIA-AS-28
Molecular Formula: $C_{20}H_{20}N_4O_2$
Molecular Weight: 348.40
Last Solvent: Water, Acetonitrile

TEST	RESULT/REFERENCE
Appearance	Light Yellow Solid
NMR Spectrum	^{1}H, 500 MHz, Dimethyl Sulfoxide-d_6, Consistent - Attached
Mass Spectrum	ESI, m/z 349 $[M + H]^+$, Attached
UPLC	96.3% (area %), ACQUITY UPLC BEH C18 (2.1 *75) mm, 1.7 micron Column, UV 254 nm Detection, Attached

Approved By

Date 10-4-2023

For Research Purposes Only. Not Intended for Food or Drug Use.

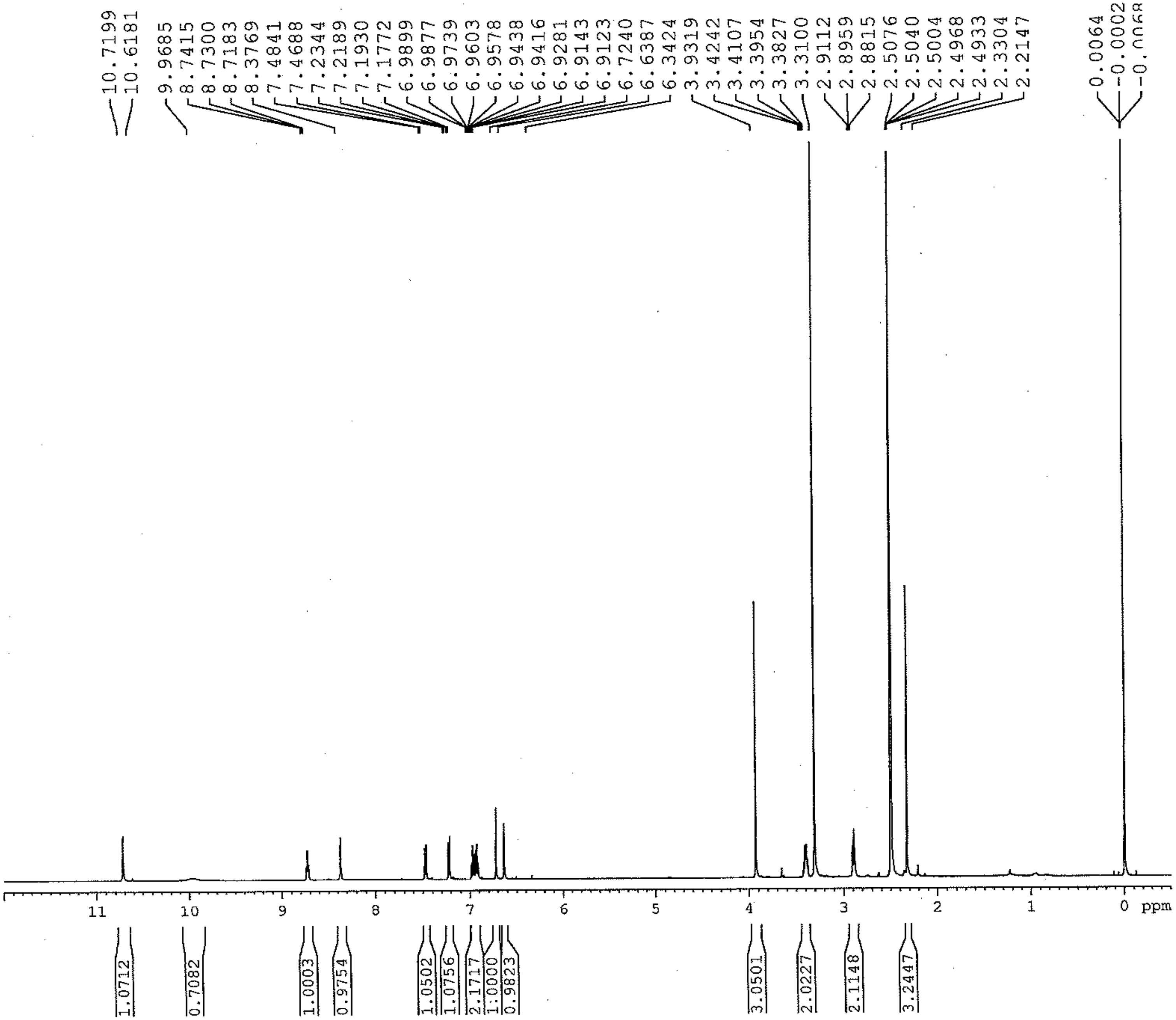

Name [illegible]
Date Sept. 28, 2023
NB # JIA-AS-28

Current Data Parameters
NAME jia-as-28
EXPNO 10
PROCNO 1

F2 - Acquisition Parameters
Date_ 20230928
Time 9.58 h
INSTRUM Avance Neo
PROBHD Z167419_0029 (
PULPROG zg30
TD 65536
SOLVENT DMSO
NS 80
DS 2
SWH 10000.000 Hz
FIDRES 0.305176 Hz
AQ 3.2767999 sec
RG 101
DW 50.000 usec
DE 11.14 usec
TE 300.0 K
D1 1.00000000 sec
TDO 1
SFO1 500.1330883 MHz
NUC1 1H
P0 2.67 usec
P1 8.00 usec
PLW1 24.22400093 W

F2 - Processing parameters
SI 65536
SF 500.1300041 MHz
WDW EM
SSB 0
LB 0.30 Hz
GB 0
PC 1.00

Openlynx Report
Vial:1:46
Date:27-Sep-2023
Name: [signature]

ID:
Time:12:55:56
Date: Sept. 28, 2023

File:jia-as-28
Notebook: JIA-AS-28

Printed: Wed Sep 27 12:58:09 2023

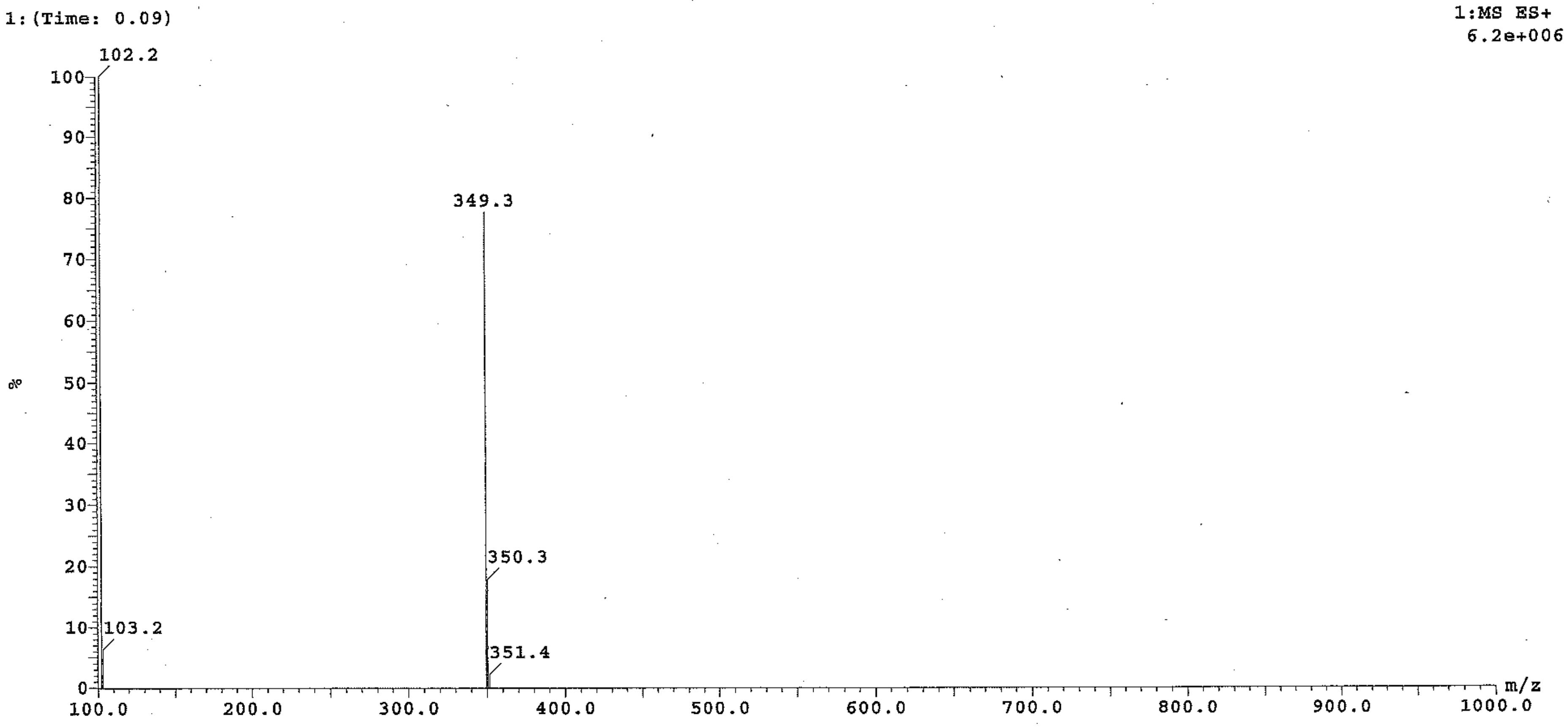

SAMPLE INFORMATION

Sample Name:	jia-as-28
Injection Volume:	3.00 ul
Run Time:	9.0 Minutes
Date Acquired:	9/27/2023 11:28:54 AM EDT
Date Processed:	9/27/2023 12:36:05 PM EDT
Sample Set Name:	Template
Acq. Method Set:	BEH_C18_PDA_75mm
Processing Method:	BEH_C18_PDA
Channel Name:	254nm

Method Notes:
Acquity UPLC BEH C18 1.7u (2.1x75mm)
Flow Rate : 0.5 mL/min
Solvent A : 0.1% TFA in Waters
Solvent B : 0.1% TFA in Acetonitrile
Solvent Gradient Program:

Time (min)	%A	% B
0:00	95	5
6:00	0	100
8:00	0	100
9:00	95	5

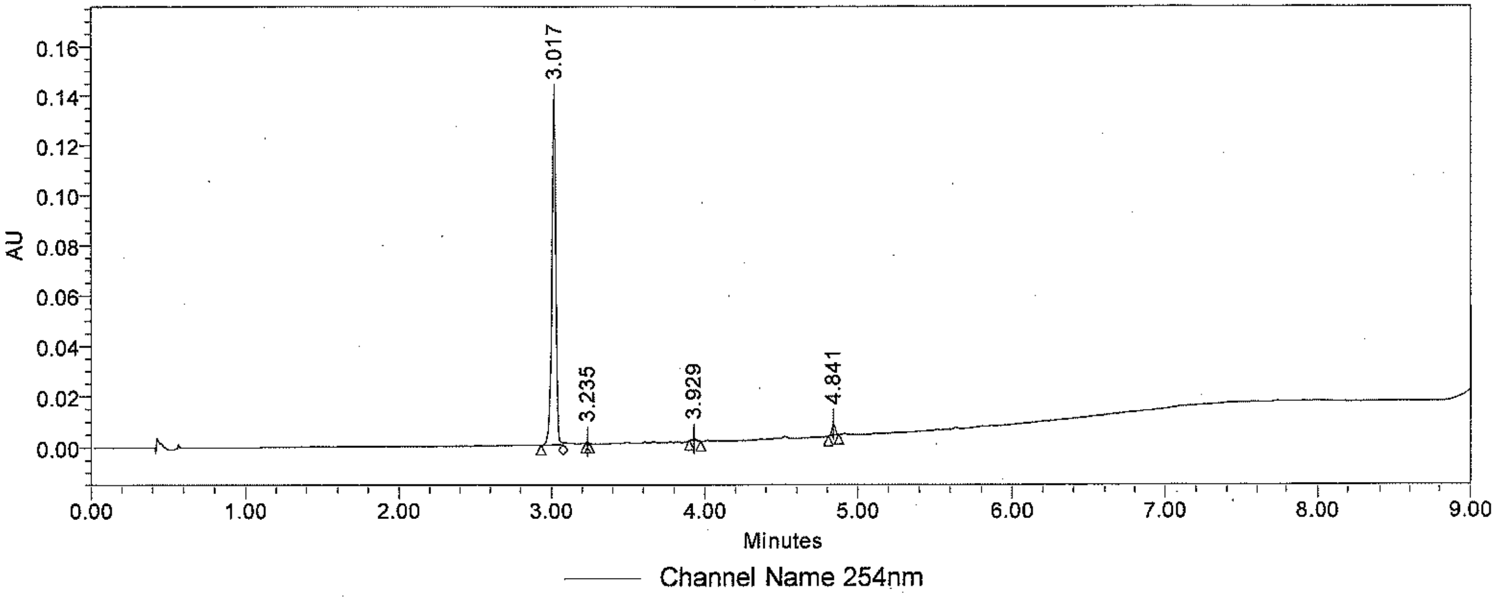

Peak Results

	RT	Area	Int Type	Width (sec)	% Area
1	3.017	240770	BV	8.549	96.34
2	3.235	554	bb	1.550	0.22
3	3.929	1933	bb	4.450	0.77
4	4.841	6653	bb	4.101	2.66

Name: [signature]

Date: Sept. 28, 2023

NB #: JIA-AS-28

CERTIFICATE OF ANALYSIS

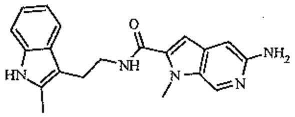

2i.BPN- 37358

Compound Name: BPN-0037358-AA-001
ALB Number: ALB-236973
Batch: 1
Lot Number: QUA-C-95-1
Molecular Formula: $C_{20}H_{21}N_5O$
Molecular Weight: 347.41
Last Solvent: Acetonitrile, Water

TEST	RESULT/REFERENCE
Appearance	Light Yellow Solid
NMR Spectrum	^1H, 500 MHz, Dimethyl Sulfoxide-d_6, Consistent - Attached
Mass Spectrum	ESI, m/z 348 $[M + H]^+$, Attached
UPLC	>99% (area %), ACQUITY UPLC BEH C18 (2.1 *75) mm, 1.7 micron Column, UV 254 nm Detection, Attached

Maria Maychack
Approved By

10-4-2023
Date

For Research Purposes Only. Not Intended for Food or Drug Use.

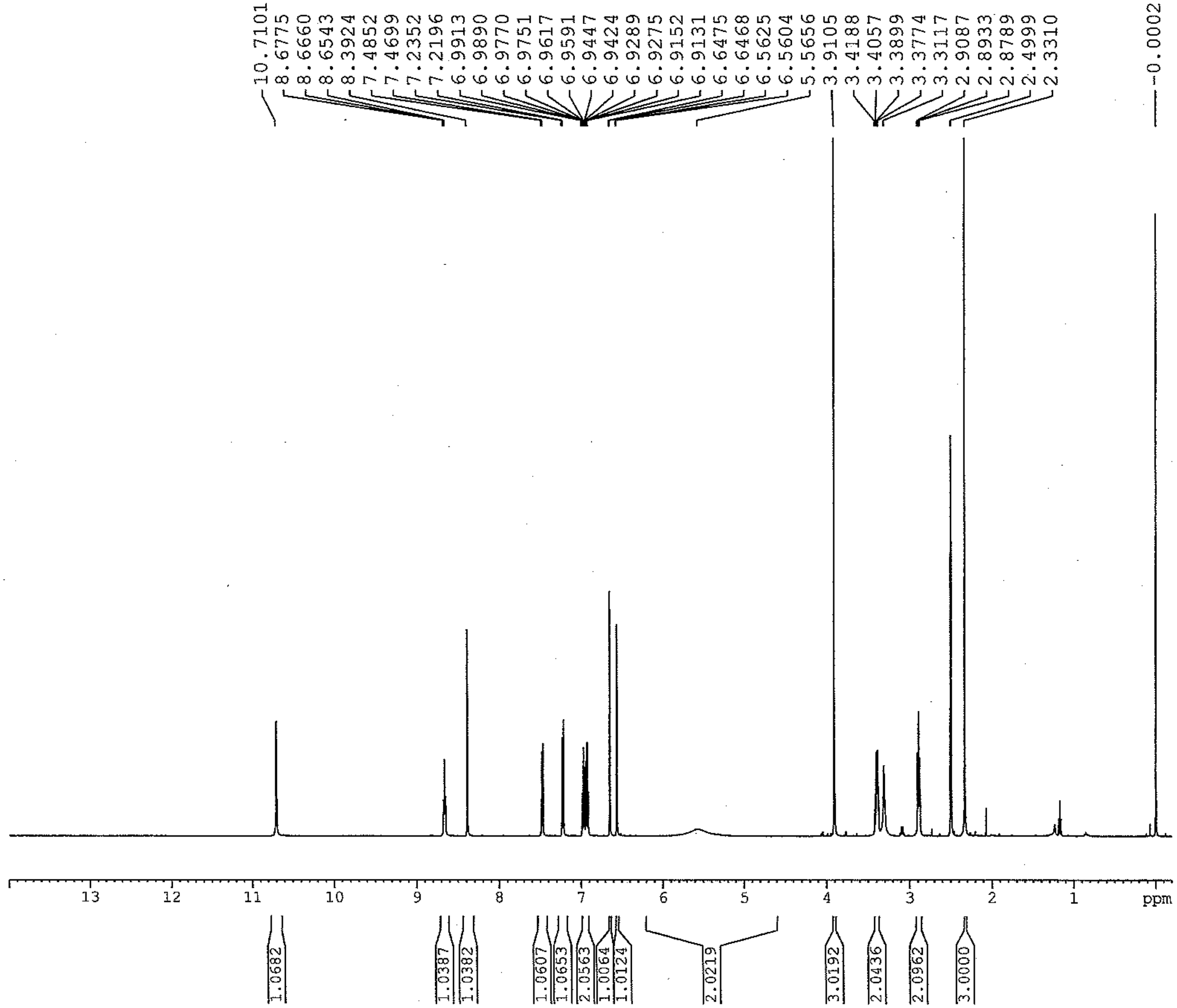

Name Tasdique Quadery
Date 10.02.23
NB # QUA-C-95-1

Current Data Parameters
NAME QUA-C-95-1
EXPNO 10
PROCNO 1

F2 - Acquisition Parameters
Date_ 20231002
Time 11.42 h
INSTRUM Avance Neo
PROBHD Z167419_0029 (
PULPROG zg30
TD 65536
SOLVENT DMSO
NS 32
DS 2
SWH 10000.000 Hz
FIDRES 0.305176 Hz
AQ 3.2767999 sec
RG 101
DW 50.000 usec
DE 11.14 usec
TE 300.0 K
D1 1.00000000 sec
TD0 1
SFO1 500.1330883 MHz
NUC1 1H
P0 2.67 usec
P1 8.00 usec
PLW1 24.22400093 W

F2 - Processing parameters
SI 65536
SF 500.1300043 MHz
WDW EM
SSB 0
LB 0.30 Hz
GB 0
PC 1.00

Openlynx Report Page 1

Vial:1:15
Date:29-Sep-2023

ID:
Time:15:50:22

File:QUA-C-95-1

Name: Tasdique Quadery

Date: 09.29.23

Notebook: QUA-C-95-1

Printed: Fri Sep 29 15:52:12 2023

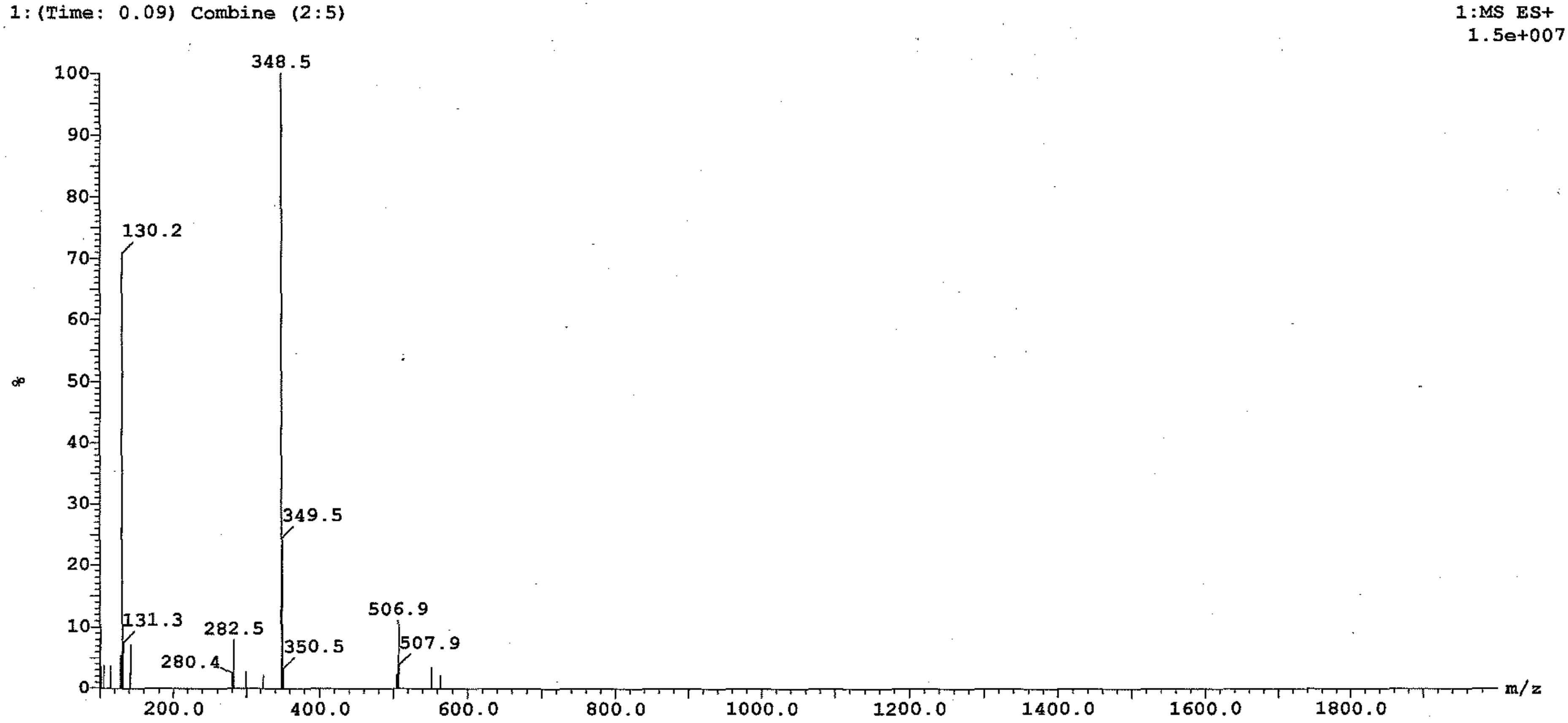

SAMPLE INFORMATION

Sample Name:	QUA-C-95-1
Injection Volume:	3.00 ul
Run Time:	9.0 Minutes
Date Acquired:	9/29/2023 3:48:16 PM EDT
Date Processed:	9/29/2023 4:00:18 PM EDT
Sample Set Name:	Template
Acq. Method Set:	BEH_C18_PDA_75mm
Processing Method:	BEH_C18_PDA
Channel Name:	254nm

Method Notes:
Acquity UPLC BEH C18 1.7u (2.1x75mm)
Flow Rate : 0.5 mL/min
Solvent A : 0.1% TFA in Waters
Solvent B : 0.1% TFA in Acetonitrile
Solvent Gradient Program:

Time (min)	%A	% B
0:00	95	5
6:00	0	100
8:00	0	100
9:00	95	5

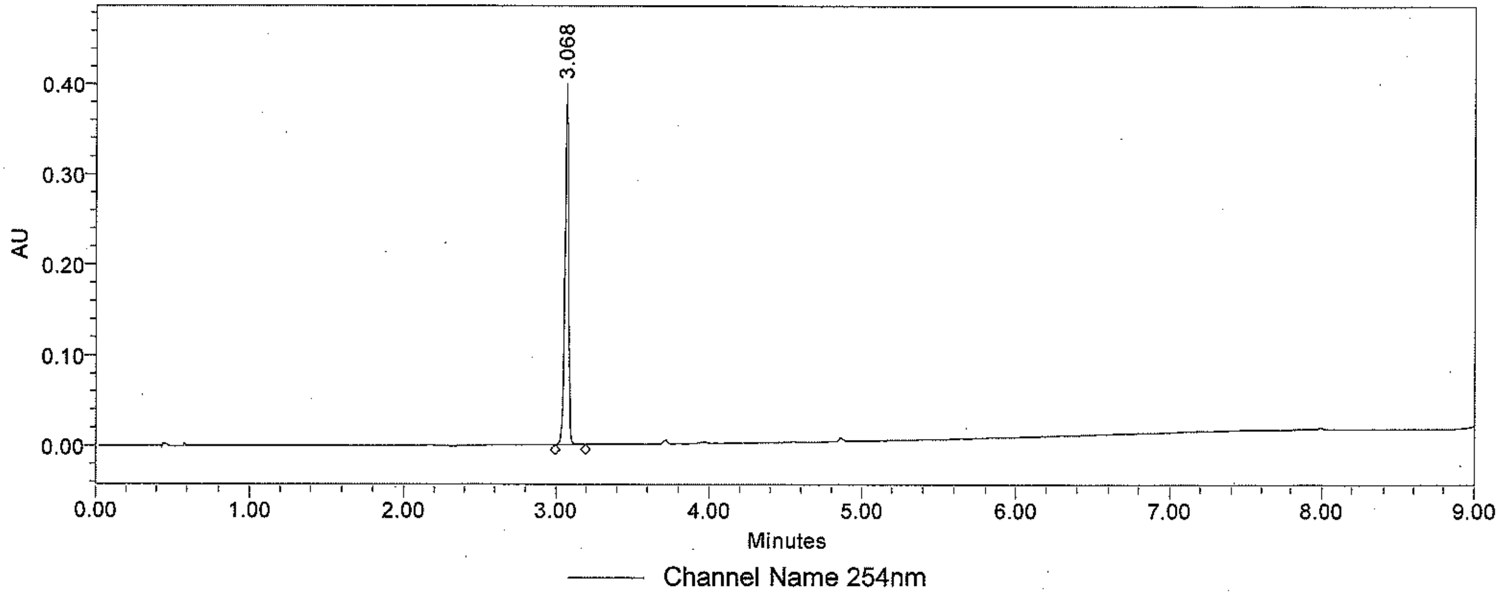

Peak Results

	RT	Area	Int Type	Width (sec)	% Area
1	3.068	661149	VV	11.999	100.00

Name: Tasdique Quadery

Date: 09.29.23

NB #: QUA-C-95-1

CERTIFICATE OF ANALYSIS

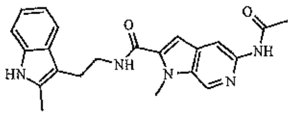

2j.BPN- 37273

Compound Name: BPN-0037273-AA-001
ALB Number: ALB-236742
Batch: 1
Lot Number: QUA-C-80-2
Molecular Formula: $C_{22}H_{23}N_5O_2$
Molecular Weight: 389.45
Last Solvent: Acetonitrile, Water

TEST	RESULT/REFERENCE
Appearance	Yellow Solid
NMR Spectrum	^{1}H, 500 MHz, Dimethyl Sulfoxide-d_6, Consistent - Attached
Mass Spectrum	ESI, m/z 390 $[M + H]^+$, Attached
UPLC	>99% (area %), ACQUITY UPLC BEH C18 (2.1 *75) mm, 1.7 micron Column, UV 254 nm Detection, Attached

MariaDMaychack
Approved By

9-20-2023
Date

For Research Purposes Only. Not Intended for Food or Drug Use.

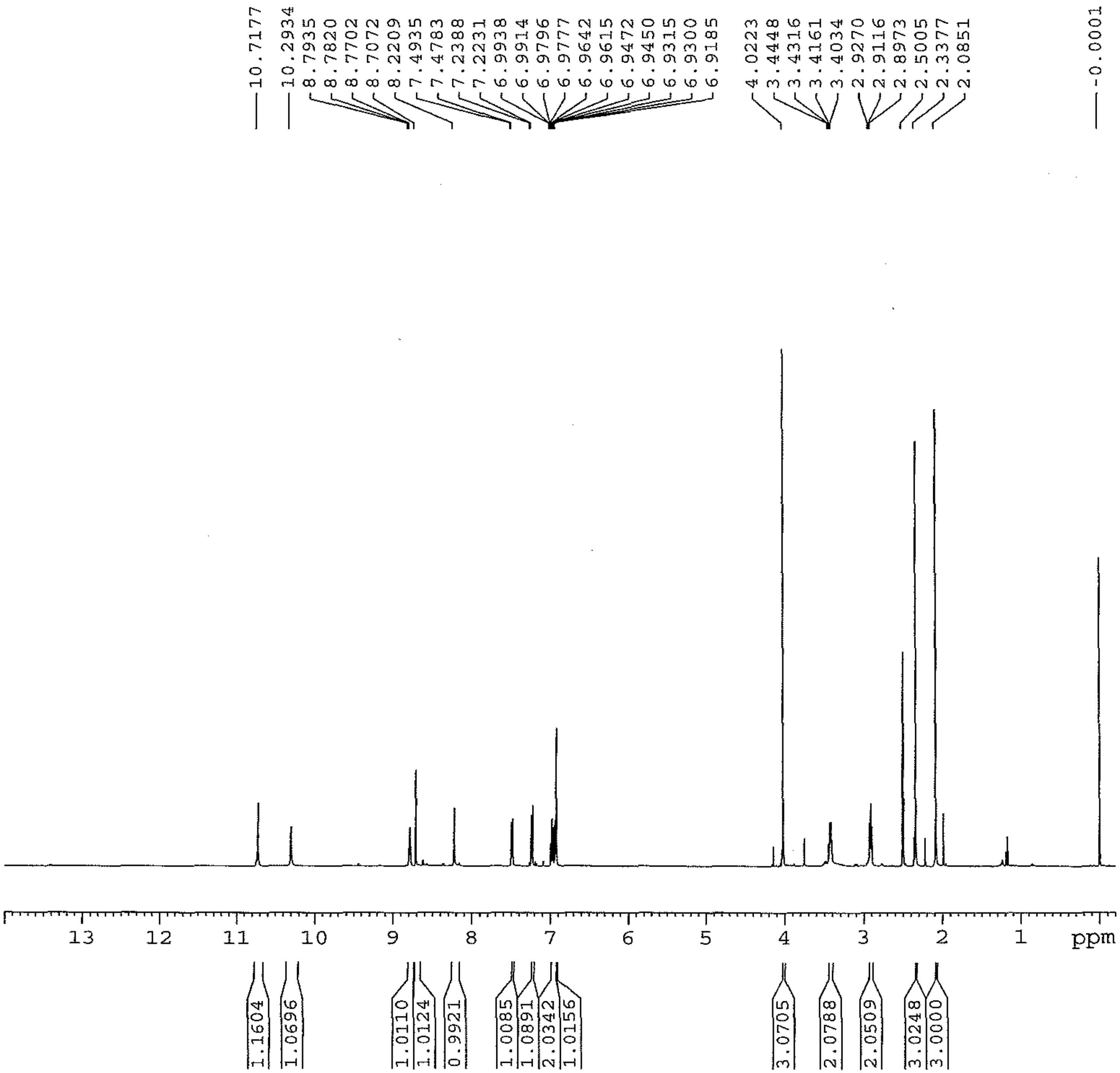

Name Tasdique Quadery
Date 09.14.23
NB # QUA-C-80-2

Current Data Parameters		
NAME	QUA-C-80-2	
EXPNO	10	
PROCNO	1	
F2 - Acquisition Parameters		
Date_	20230914	
Time	14.20	h
INSTRUM	Avance Neo	
PROBHD	Z167419_0029 (	
PULPROG	zg30	
TD	65536	
SOLVENT	DMSO	
NS	32	
DS	2	
SWH	10000.000	Hz
FIDRES	0.305176	Hz
AQ	3.2767999	sec
RG	101	
DW	50.000	usec
DE	11.14	usec
TE	300.0	K
D1	1.00000000	sec
TD0	1	
SFO1	500.1330883	MHz
NUC1	1H	
P0	2.67	usec
P1	8.00	usec
PLW1	24.22400093	W
F2 - Processing parameters		
SI	65536	
SF	500.1300040	MHz
WDW	EM	
SSB	0	
LB	0.30	Hz
GB	0	
PC	1.00	

Openlynx Report
Vial:2:12
Date:14-Sep-2023

ID:
Time:15:57:34

File:QUA-C-80-2

Name: Tasdique Quadery
Date: 09.14.23
Notebook: QUA-C-80-2

Printed: Thu Sep 14 15:59:55 2023

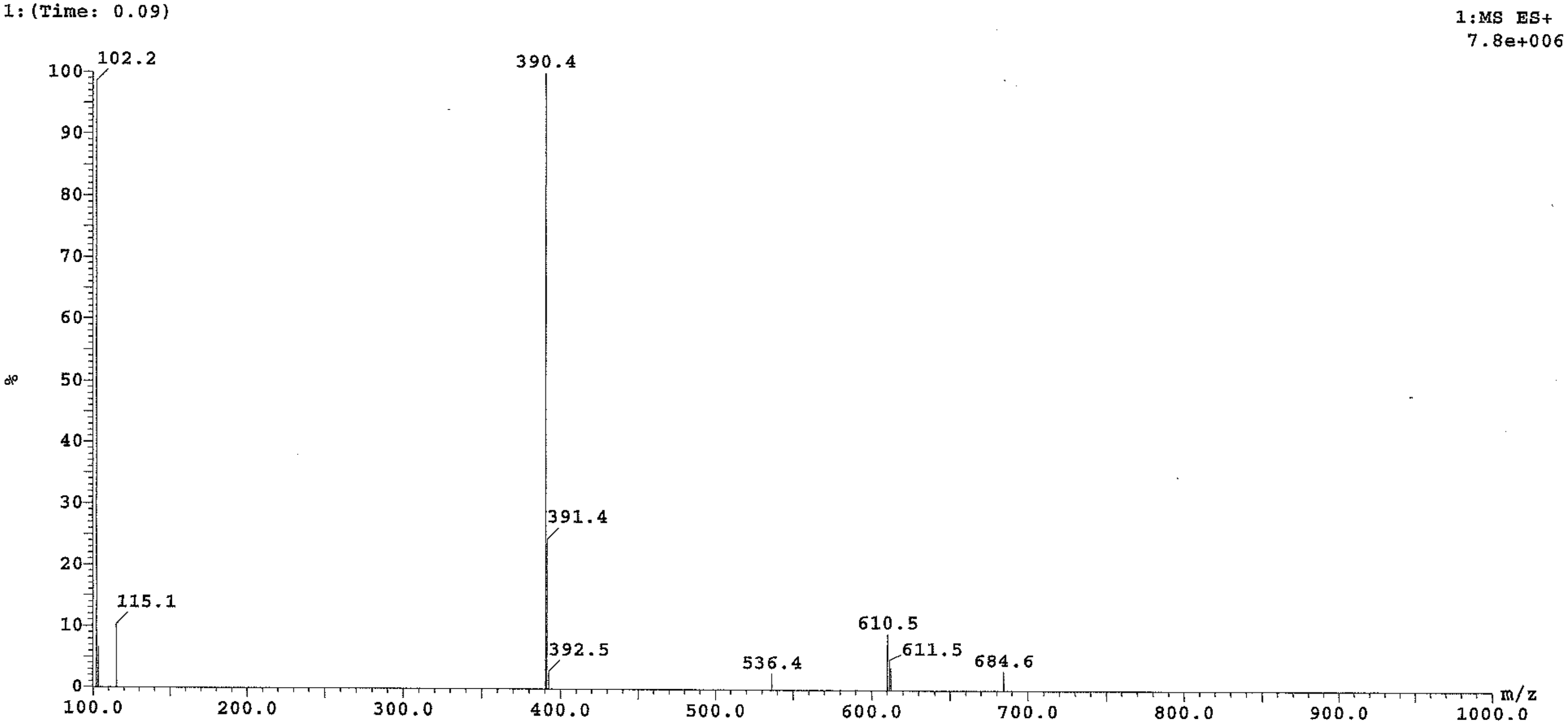

CERTIFICATE OF ANALYSIS

Compound Name: BPN-0037690-AA-001
ALB Number: ALB-238677
Batch: 1
Lot Number: ALK-D-185-2
Molecular Formula: $C_{21}H_{23}N_5O$
Molecular Weight: 361.44
Last Solvent: Water, Acetonitrile

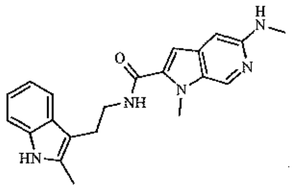

2k.BPN- 37690

TEST	RESULT/REFERENCE
Appearance	Off-white Solid
NMR Spectrum	^{1}H, 500 MHz, Dimethyl Sulfoxide-d_6, Consistent - Attached
Mass Spectrum	ESI, m/z 362 $[M + H]^+$, Attached
UPLC	98.9% (area %), ACQUITY UPLC BEH C18 (2.1 *75) mm, 1.7 micron Column, UV 254 nm Detection, Attached

Mana Maychach

Approved By

2-21-2024

Date

For Research Purposes Only. Not Intended for Food or Drug Use.

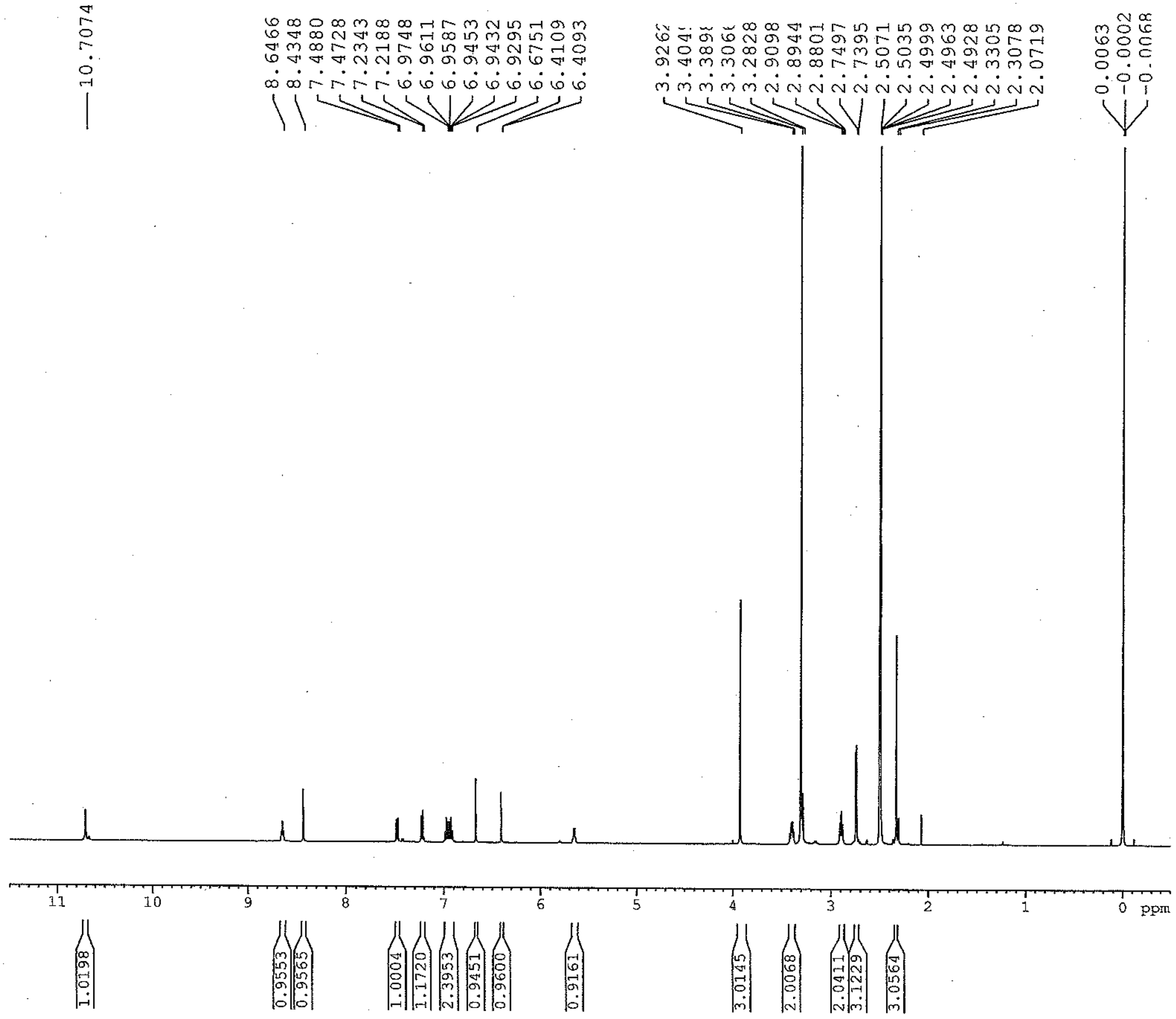

Name Marwah Albaker
Date 13-Feb-2024
NB # ALK-D-185-2

Current Data Parameters

NAME	ALK-D-185-2
EXPNO	10
PROCNO	1

F2 - Acquisition Parameters

Date_	20240213
Time	8.26 h
INSTRUM	Avance Neo
PROBHD	Z167419_0029 (
PULPROG	zg30
TD	65536
SOLVENT	DMSO
NS	64
DS	2
SWH	10000.000 Hz
FIDRES	0.305176 Hz
AQ	3.2767999 sec
RG	101
DW	50.000 usec
DE	11.14 usec
TE	300.0 K
D1	1.00000000 sec
TD0	1
SFO1	500.1330883 MHz
NUC1	1H
P0	2.67 usec
P1	8.00 usec
PLW1	24.22400093 W

F2 - Processing parameters

SI	65536
SF	500.1300043 MHz
WDW	EM
SSB	0
LB	0.30 Hz
GB	0
PC	1.00

ACQ-SQD#F07SQD100W

13-Feb-2024
07:27:28

Name: Marwah Albaker
Date: 13-Feb-2024
Notebook: ALK-D-185-2

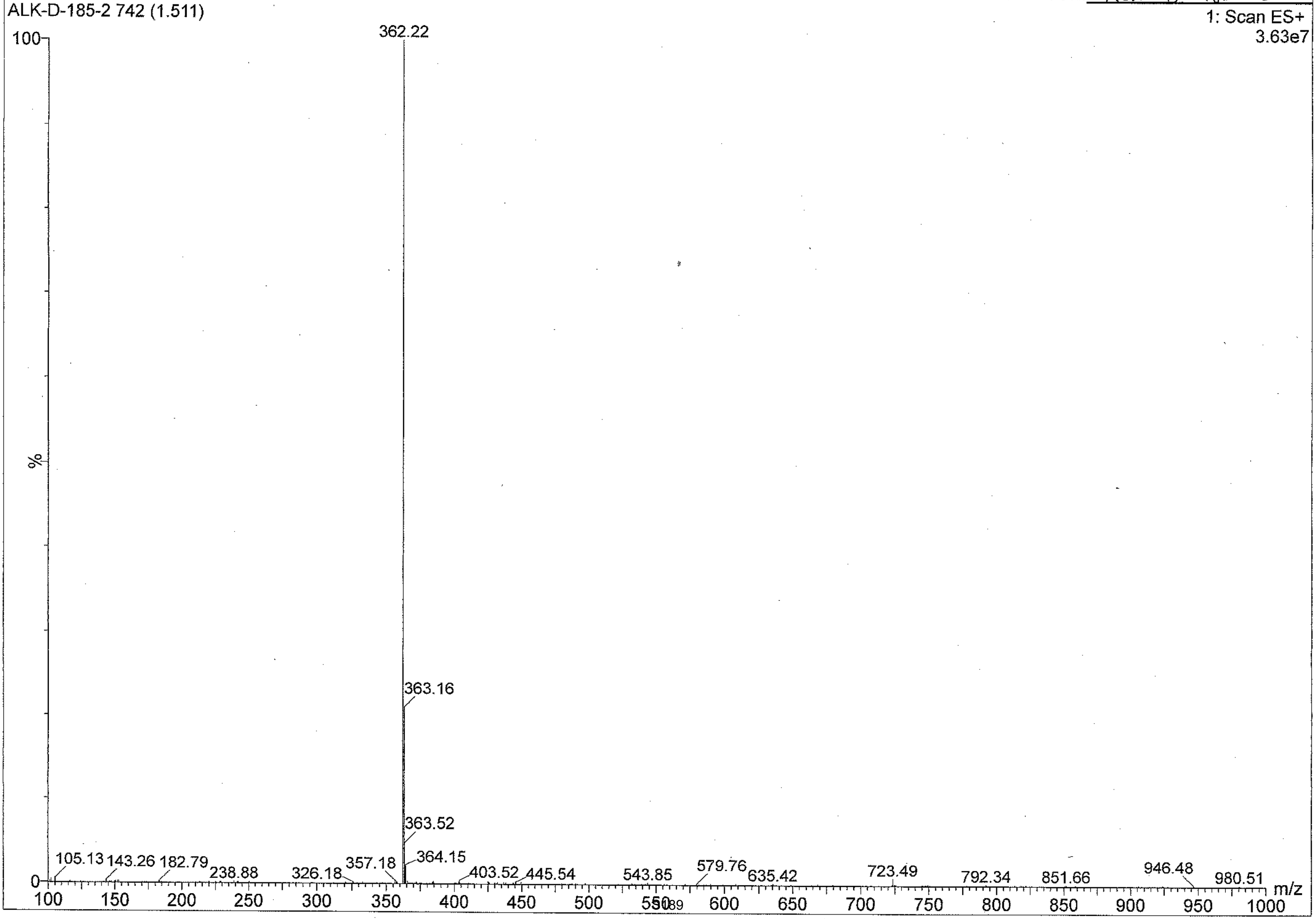

SAMPLE INFORMATION

Sample Name:	ALK-D-185-2
Injection Volume:	2.00 ul
Run Time:	9.0 Minutes
Date Acquired:	2/13/2024 7:33:19 AM EST
Date Processed:	2/13/2024 8:08:13 AM EST
Sample Set Name:	Template
Acq. Method Set:	BEH_C18_PDA_75mm
Processing Method:	BEH_C18_PDA
Channel Name:	254nm

Method Notes:
Acquity UPLC BEH C18 1.7u (2.1x75mm)
Flow Rate : 0.5 mL/min
Solvent A : 0.1% TFA in Waters
Solvent B : 0.1% TFA in Acetonitrile
Solvent Gradient Program:

Time (min)	%A	% B
0:00	95	5
6:00	0	100
8:00	0	100
9:00	95	5

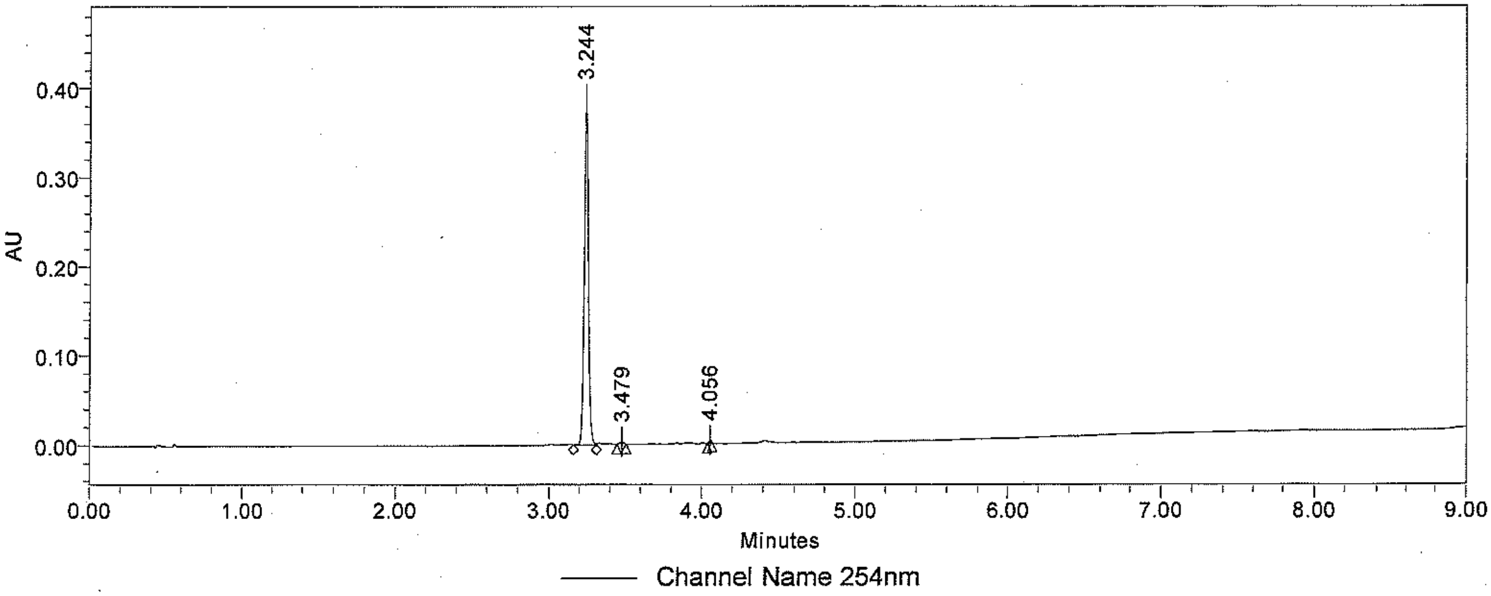

Peak Results

	RT	Area	Int Type	Width (sec)	% Area
1	3.244	701070	VV	8.849	98.94
2	3.479	4499	bb	3.550	0.63
3	4.056	3013	bb	1.800	0.43

Name: Marwah Albaker

Date: 13-Feb-2024

NB #: ALK-D-185-2

CERTIFICATE OF ANALYSIS

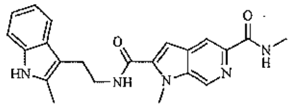

2l. BPN- 37703

Compound Name: BPN-0037703-AA-001
ALB Number: ALB-238846
Batch: 1
Lot Number: ALK-D-199-3
Molecular Formula: $C_{22}H_{23}N_5O_2$
Molecular Weight: 389.45
Last Solvent: Water, Acetonitrile

TEST	RESULT/REFERENCE
Appearance	Off-white Solid
NMR Spectrum	^{1}H, 500 MHz, Dimethyl Sulfoxide-d_6, Consistent - Attached
Mass Spectrum	ESI, *m/z* 390 $[M + H]^+$, Attached
UPLC	97.2% (area %), ACQUITY UPLC BEH C18 (2.1 *75) mm, 1.7 micron Column, UV Detection, Attached

Approved By

2-28-2024

Date

For Research Purposes Only. Not Intended for Food or Drug Use.

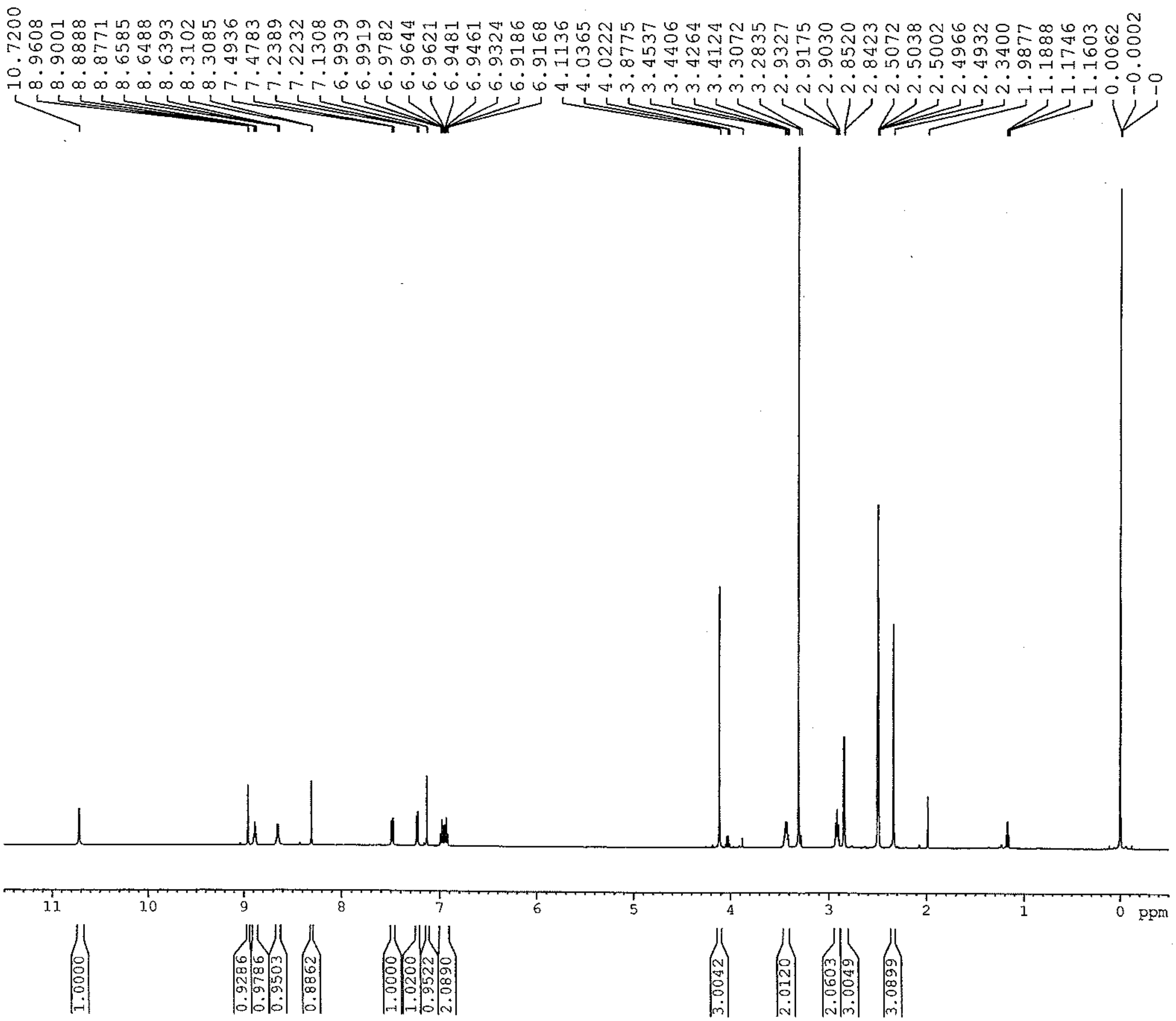

Name Marwah Alhaker

Date 26-Feb-2024

NB # ALK-D-199-3

```
Current Data Parameters
NAME          ALK-D-199-3
EXPNO                  10
PROCNO                  1

F2 - Acquisition Parameters
Date_            20240226
Time                 9.27 h
INSTRUM        Avance Neo
PROBHD   Z167419_0029 (
PULPROG              zg30
TD                  65536
SOLVENT              DMSO
NS                     64
DS                      2
SWH             10000.000 Hz
FIDRES           0.305176 Hz
AQ              3.2767999 sec
RG                    101
DW                 50.000 usec
DE                  11.14 usec
TE                  300.0 K
D1             1.00000000 sec
TD0                     1
SFO1          500.1330883 MHz
NUC1                   1H
P0                   2.67 usec
P1                   8.00 usec
PLW1          24.22400093 W

F2 - Processing parameters
SI                  65536
SF            500.1300041 MHz
WDW                    EM
SSB                     0
LB                   0.30 Hz
GB                      0
PC                   1.00
```

ACQ-SQD#F07SQD100W **23-Feb-2024 11:52:15**

Name: Marwah Albakkar
Date: 23-Feb-2024
Notebook: ALK-D-199-3

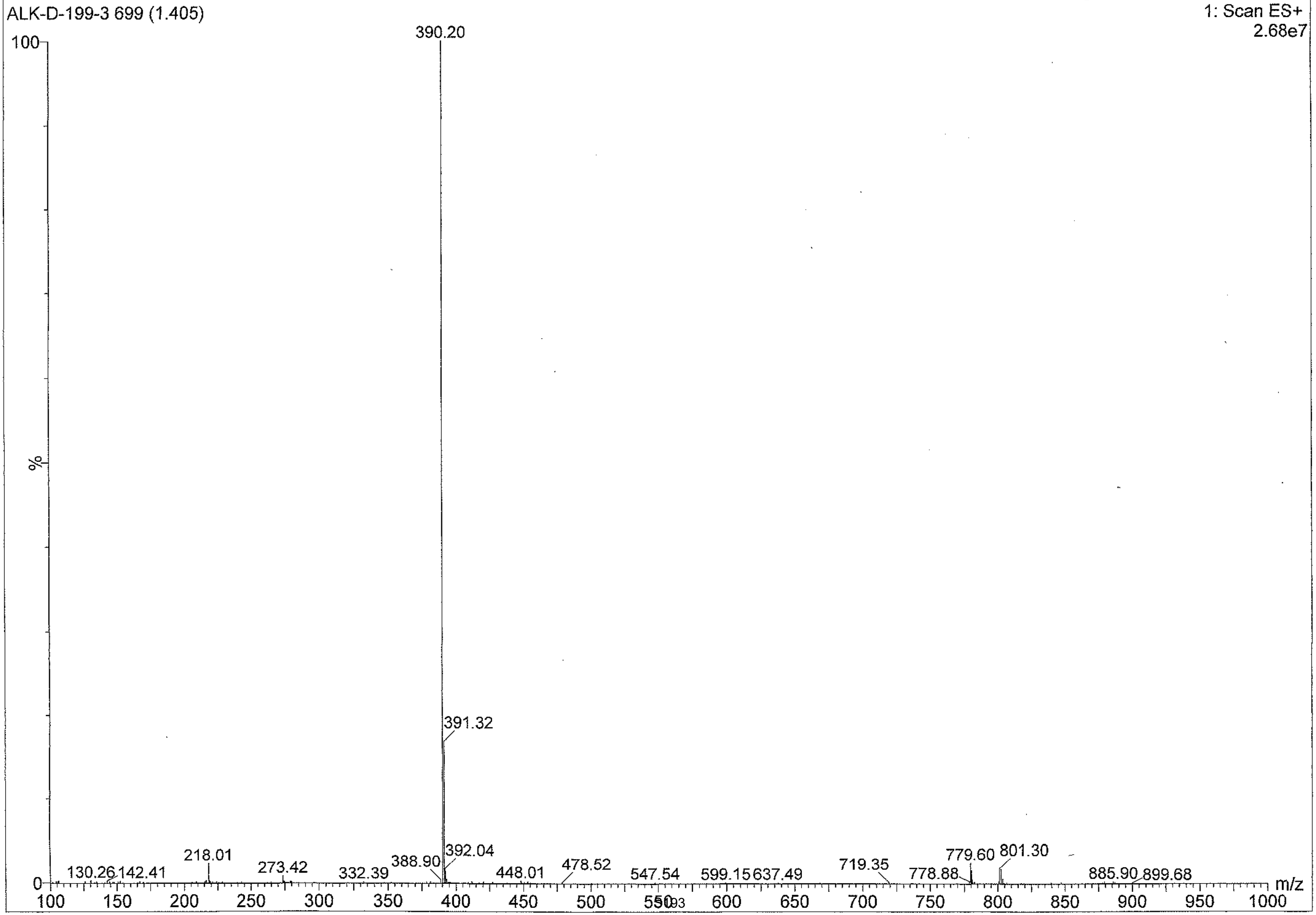

SAMPLE INFORMATION

Sample Name: ALK-D-199-3
Injection Volume: 1.50 ul
Run Time: 9.0 Minutes
Date Acquired: 2/23/2024 12:12:06 PM EST
Date Processed: 2/23/2024 12:26:17 PM EST
Sample Set Name: Template
Acq. Method Set: BEH_C18_PDA_75mm
Processing Method: BEH_C18_PDA
Channel Name: 254nm

Method Notes:
Acquity UPLC BEH C18 1.7u (2.1x75mm)
Flow Rate : 0.5 mL/min
Solvent A : 0.1% TFA in Waters
Solvent B : 0.1% TFA in Acetonitrile
Solvent Gradient Program:

Time (min)	%A	% B
0:00	95	5
6:00	0	100
8:00	0	100
9:00	95	5

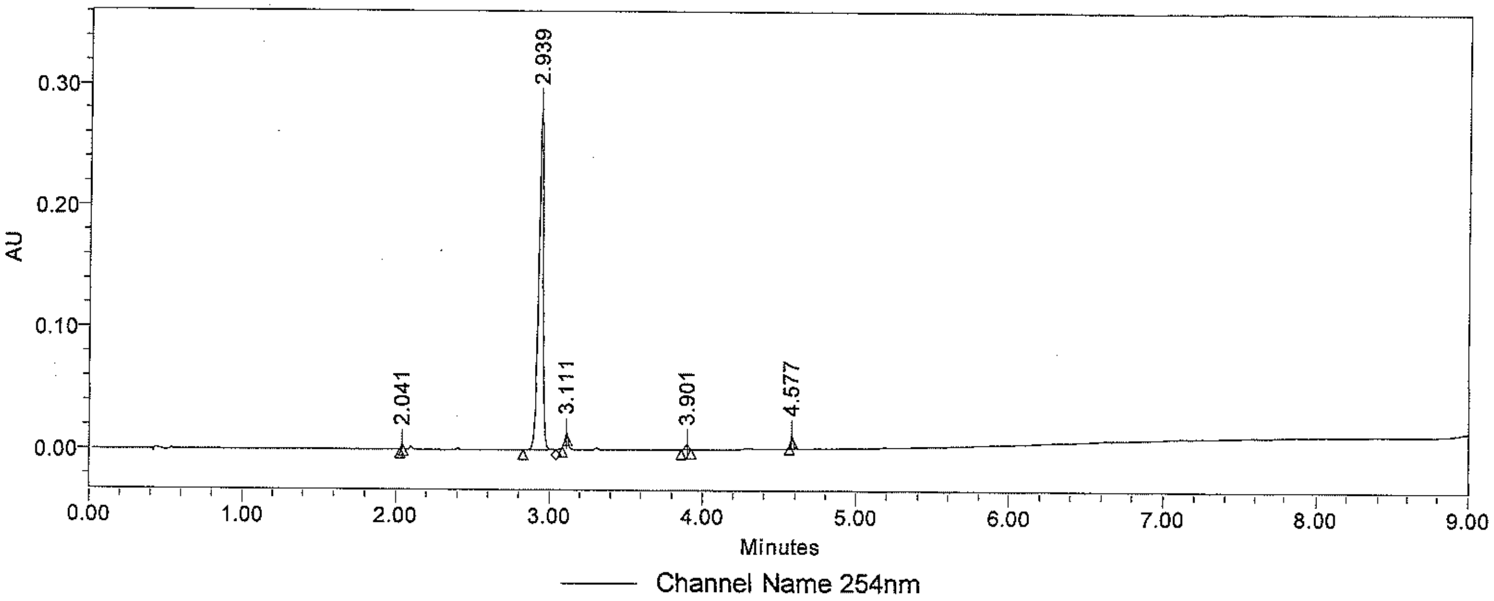

Peak Results

	RT	Area	Int Type	Width (sec)	% Area
1	2.041	1306	bb	1.200	0.23
2	2.939	558308	BV	12.899	97.24
3	3.111	4289	bb	2.050	0.75
4	3.901	6550	bb	3.850	1.14
5	4.577	3731	bb	1.200	0.65

Name: Marwah Alhaker

Date: 23-Feb-2024

NB #: ALK-D-199-3

CERTIFICATE OF ANALYSIS

Compound Name: BPN-0037637-AA-001
ALB Number: ALB-238318
Batch: 1
Lot Number: JIA-AS-80
Molecular Formula: $C_{21}H_{19}N_5O$
Molecular Weight: 357.41
Last Solvent: Water, Acetonitrile

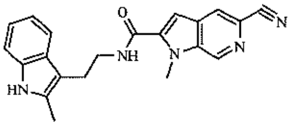

2m. BPN- 37637

TEST	RESULT/REFERENCE
Appearance	Off-white Solid
NMR Spectrum	^{1}H, 500 MHz, Dimethyl Sulfoxide-d_6, Consistent - Attached
Mass Spectrum	ESI, *m/z* 358 $[M + H]^+$, Attached
UPLC	98.6% (area %), ACQUITY UPLC BEH C18 (2.1 *75) mm, 1.7 micron Column, UV 254 nm Detection, Attached

Approved By [signature]

Date 1-24-2024

For Research Purposes Only. Not Intended for Food or Drug Use.

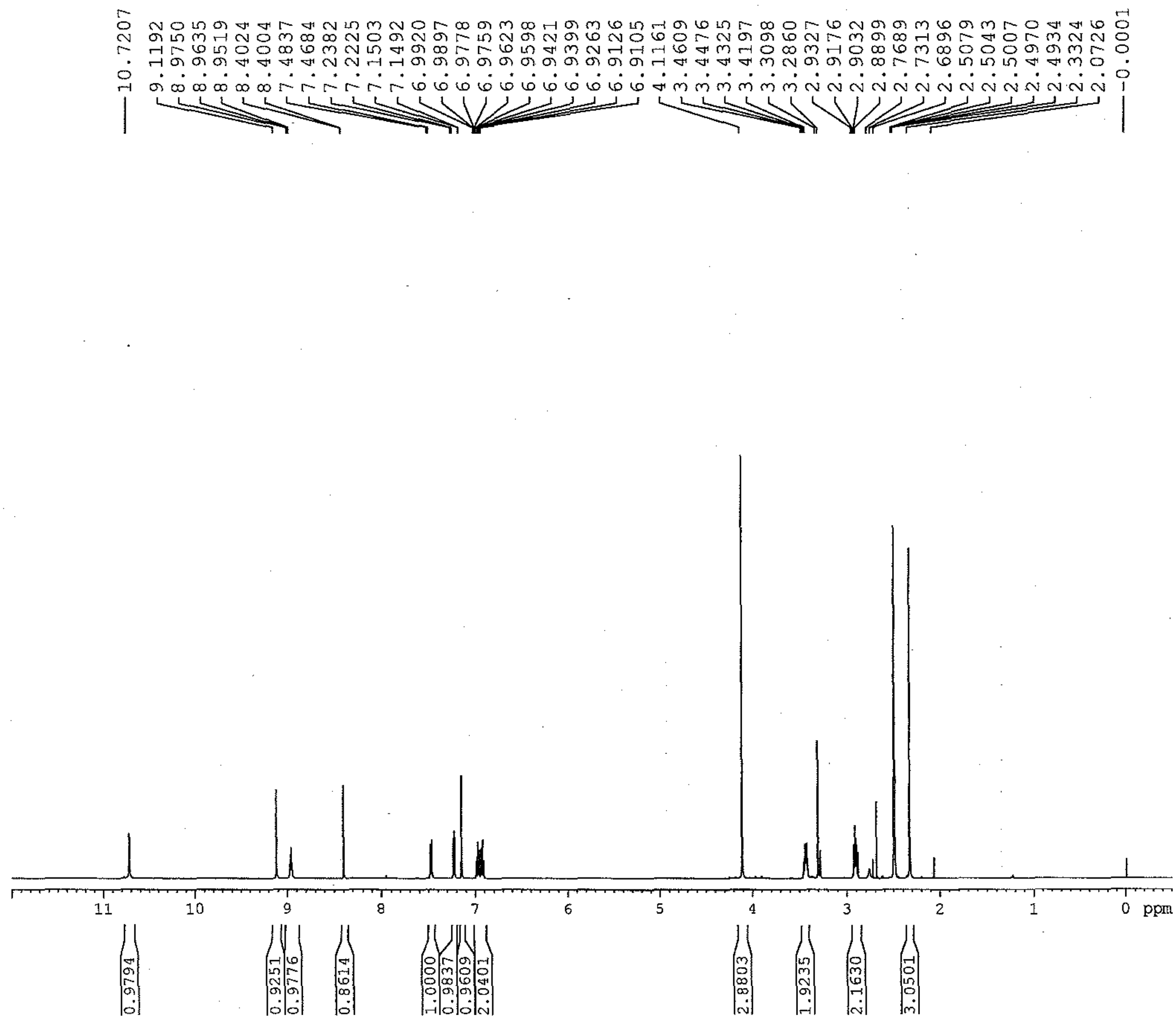

Name ____

Date Jan. 22, 2024

NB # JIA-AS-80

```
Current Data Parameters
NAME            jia-as-80
EXPNO                  10
PROCNO                  1

F2 - Acquisition Parameters
Date_            20240122
Time                10.02 h
INSTRUM         Avance Neo
PROBHD   Z167419_0029 (
PULPROG              zg30
TD                  65536
SOLVENT              DMSO
NS                     40
DS                      2
SWH             10000.000 Hz
FIDRES           0.305176 Hz
AQ              3.2767999 sec
RG                    101
DW                 50.000 usec
DE                  11.14 usec
TE                  300.0 K
D1             1.00000000 sec
TD0                     1
SFO1          500.1330883 MHz
NUC1                   1H
P0                   2.67 usec
P1                   8.00 usec
PLW1          24.22400093 W

F2 - Processing parameters
SI                  65536
SF            500.1300039 MHz
WDW                    EM
SSB                     0
LB                   0.30 Hz
GB                      0
PC                   1.00
```

Openlynx Report

Vial:2:24

Date:18-Jan-2024

Name: [signature]

ID:

Time:15:16:20

Date: Jan. 22, 2024

File:JIA-AS-80

Notebook: JIA-AS-80

Page 1

Printed: Thu Jan 18 15:18:39 2024

1:(Time: 0.09)

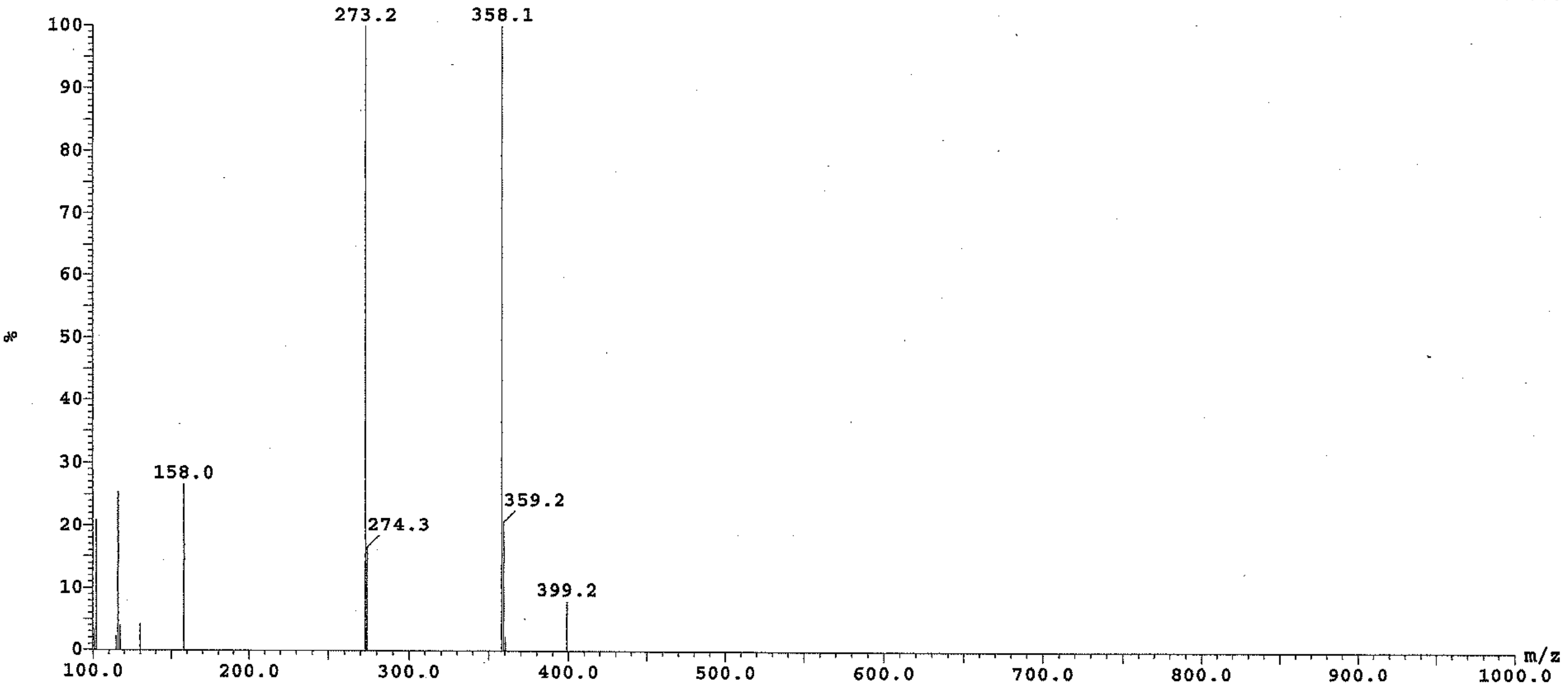

SAMPLE INFORMATION

Sample Name: jia-as-80
Injection Volume: 3.00 ul
Run Time: 9.0 Minutes
Date Acquired: 1/18/2024 3:09:08 PM EST
Date Processed: 1/18/2024 3:21:10 PM EST
Sample Set Name: Template
Acq. Method Set: BEH_C18_PDA_75mm
Processing Method: BEH_C18_PDA
Channel Name: 254nm

Method Notes:
Acquity UPLC BEH C18 1.7u (2.1x75mm)
Flow Rate : 0.5 mL/min
Solvent A : 0.1% TFA in Waters
Solvent B : 0.1% TFA in Acetonitrile
Solvent Gradient Program:

Time (min)	%A	% B
0:00	95	5
6:00	0	100
8:00	0	100
9:00	95	5

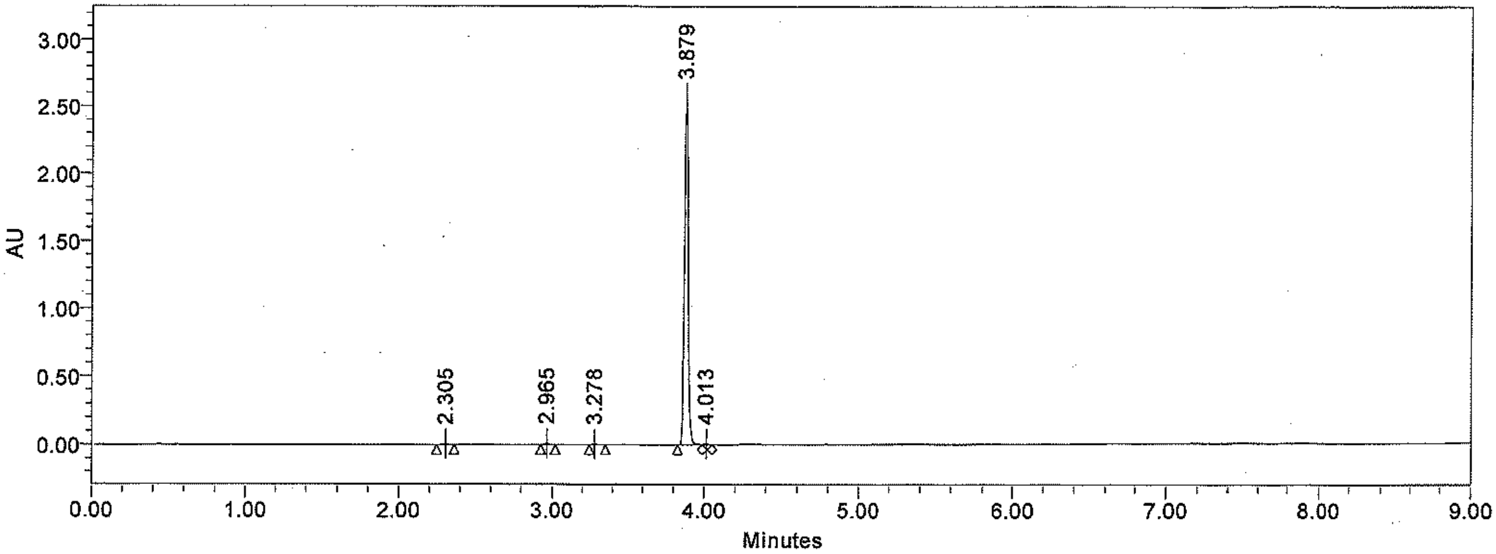

Peak Results

	RT	Area	Int Type	Width (sec)	% Area
1	2.305	17023	BB	6.949	0.39
2	2.965	16435	BB	5.650	0.38
3	3.278	11749	BB	6.400	0.27
4	3.879	4254725	BV	9.649	98.65
5	4.013	13098	VV	3.751	0.30

Name: [signature]

Date: Jan. 22, 2024

NB #: JIA-AS-80

CERTIFICATE OF ANALYSIS

Compound Name: BPN-0037666-AA-001
ALB Number: ALB-238522
Batch: 1
Lot Number: JIA-AS-87
Molecular Formula: $C_{21}H_{22}N_4O_3S$
Molecular Weight: 410.49
Last Solvent: Water, Acetonitrile

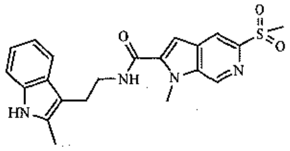

2n. BPN- 37666

TEST	RESULT/REFERENCE
Appearance	Off-white Solid
NMR Spectrum	^1H, 500 MHz, Dimethyl Sulfoxide-d_6, Consistent - Attached
Mass Spectrum	ESI, m/z 411 $[M + H]^+$, Attached
UPLC	97.8% (area %), ACQUITY UPLC BEH C18 (2.1 *75) mm, 1.7 micron Column, UV 254 nm Detection, Attached

Approved By: [signature]

Date: 2-7-2024

For Research Purposes Only. Not Intended for Food or Drug Use.

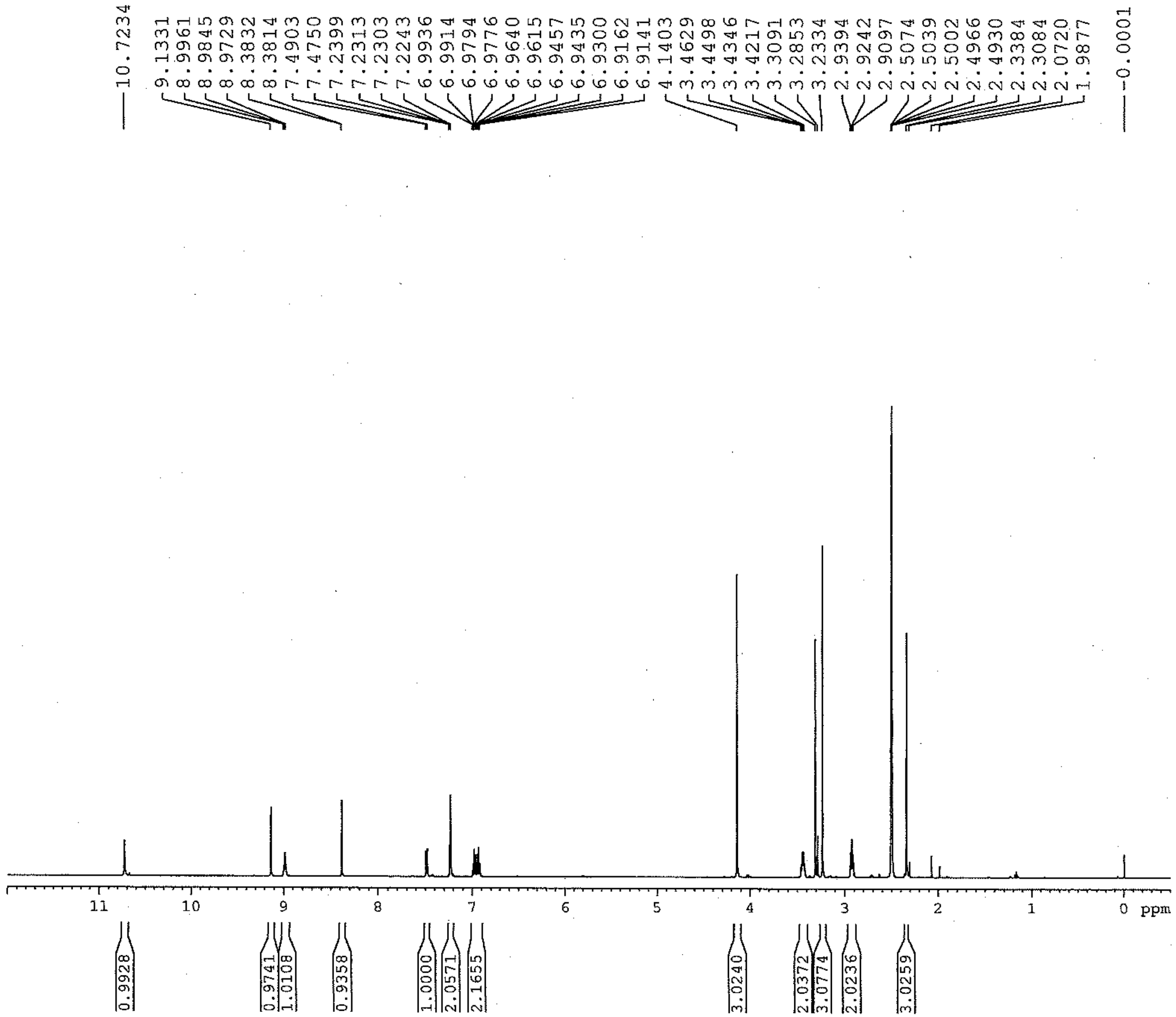

Name

Date Feb. 5, 2024

NB # VIA-AS-87

Current Data Parameters
NAME jia-as-87
EXPNO 10
PROCNO 1

F2 - Acquisition Parameters
Date_ 20240205
Time 9.10 h
INSTRUM Avance Neo
PROBHD Z167419_0029 (
PULPROG zg30
TD 65536
SOLVENT DMSO
NS 40
DS 2
SWH 10000.000 Hz
FIDRES 0.305176 Hz
AQ 3.2767999 sec
RG 101
DW 50.000 usec
DE 11.14 usec
TE 300.0 K
D1 1.00000000 sec
TD0 1
SFO1 500.1330883 MHz
NUC1 1H
P0 2.67 usec
P1 8.00 usec
PLW1 24.22400093 W

F2 - Processing parameters
SI 65536
SF 500.1300041 MHz
WDW EM
SSB 0
LB 0.30 Hz
GB 0
PC 1.00

Openlynx Report

Vial:1:25
Date:02-Feb-2024
Name: [signature]

ID:
Time:10:17:10
Date: Feb. 5, 2024

File:JIA-AS-87
Notebook: JIA-AS-87

Printed: Fri Feb 02 10:19:21 2024

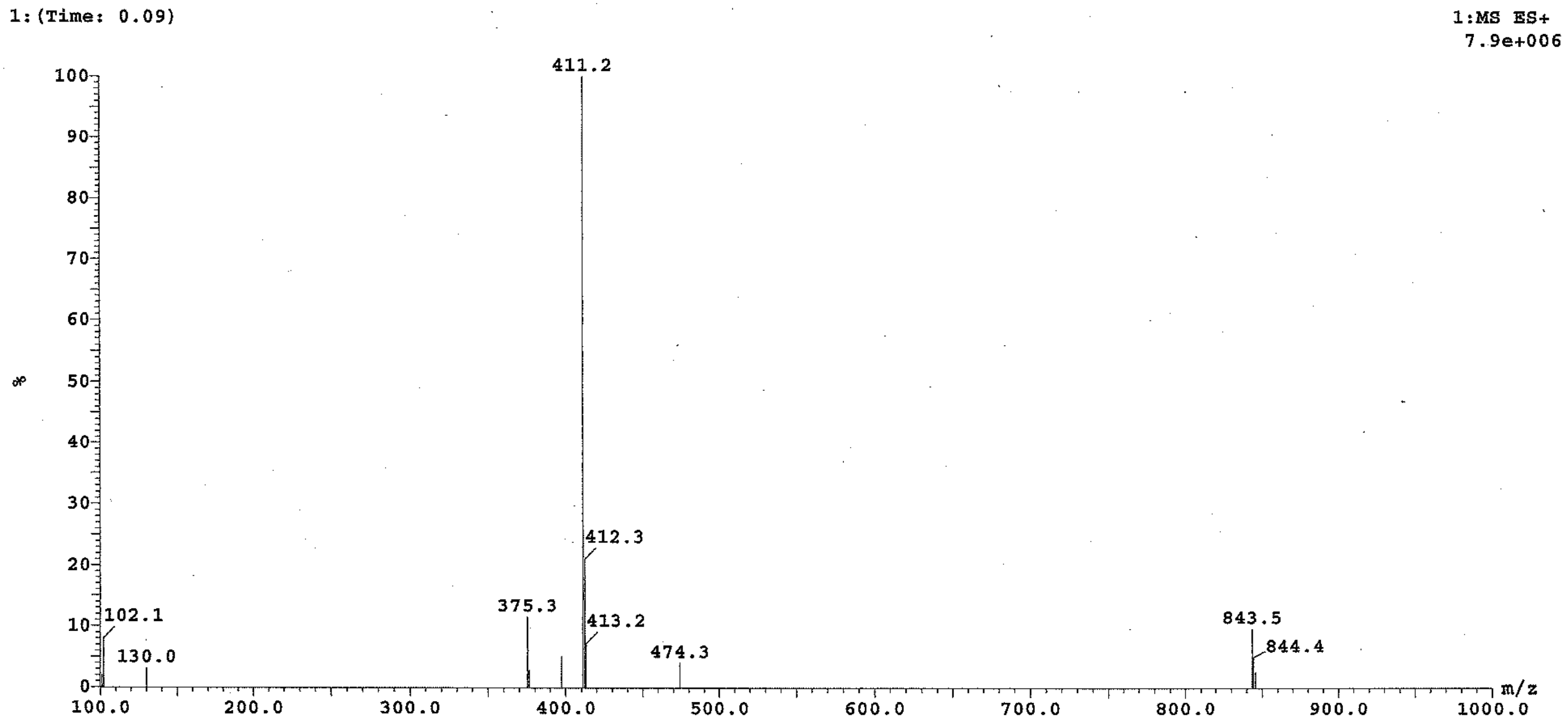

SAMPLE INFORMATION

Sample Name: jia-as-87
Injection Volume: 4.00 ul
Run Time: 9.0 Minutes
Date Acquired: 2/2/2024 9:53:46 AM EST
Date Processed: 2/2/2024 10:10:32 AM EST
Sample Set Name: Template
Acq. Method Set: BEH_C18_PDA_75mm
Processing Method: BEH_C18_PDA
Channel Name: 254nm

Method Notes:
Acquity UPLC BEH C18 1.7u (2.1x75mm)
Flow Rate : 0.5 mL/min
Solvent A : 0.1% TFA in Waters
Solvent B : 0.1% TFA in Acetonitrile
Solvent Gradient Program:

Time (min)	%A	% B
0:00	95	5
6:00	0	100
8:00	0	100
9:00	95	5

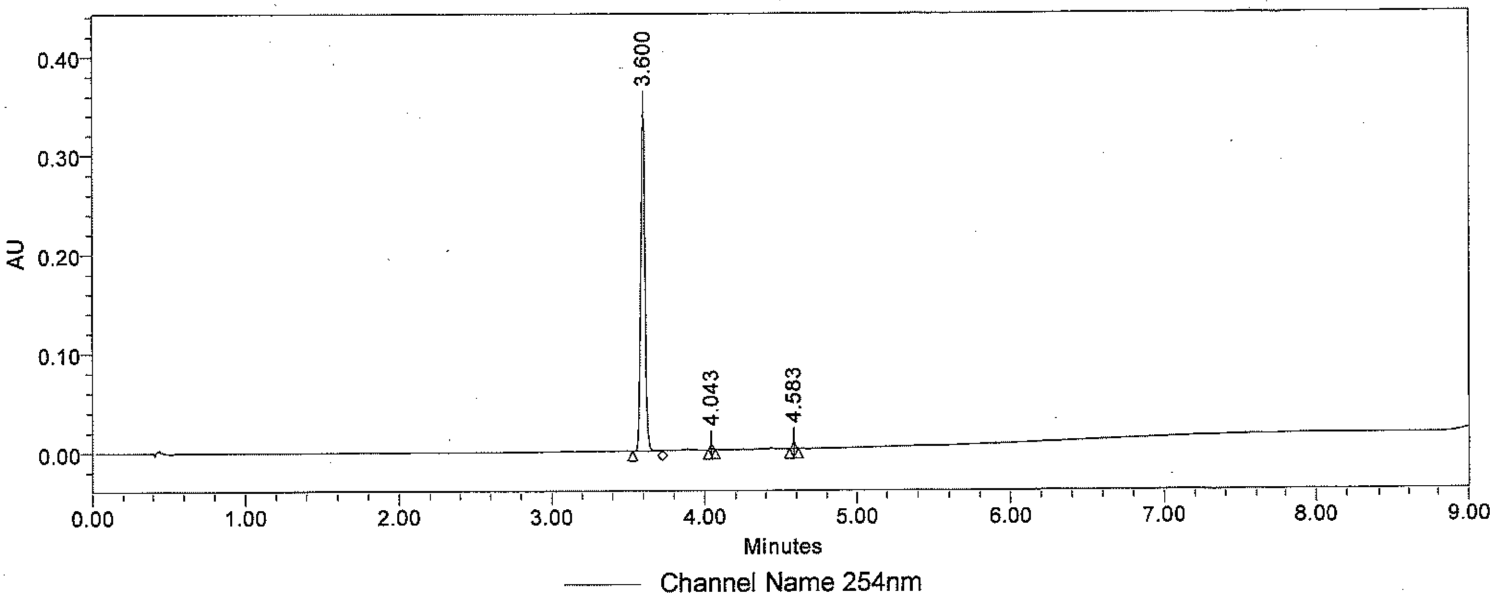

Peak Results

	RT	Area	Int Type	Width (sec)	% Area
1	3.600	650183	BV	11.599	97.84
2	4.043	5498	bb	2.651	0.83
3	4.583	8826	Bb	3.551	1.33

Name: [signature]

Date: Feb. 5, 2024

NB #: JIA-AS-87

CERTIFICATE OF ANALYSIS

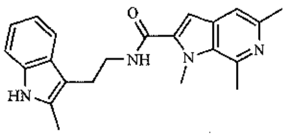

2o.BPN- 37692

Compound Name: BPN-0037692-AA-001
ALB Number: ALB-238678
Batch: 1
Lot Number: JIA-AS-91
Molecular Formula: $C_{22}H_{24}N_4O$
Molecular Weight: 360.45
Last Solvent: Acetonitrile

TEST	RESULT/REFERENCE
Appearance	White Solid
NMR Spectrum	^1H, 500 MHz, Dimethyl Sulfoxide-d_6, Consistent - Attached
Mass Spectrum	ESI, *m/z* 361 $[M + H]^+$, Attached
UPLC	99.0% (area %), ACQUITY UPLC BEH C18 (2.1 *75) mm, 1.7 micron Column, UV 254 nm Detection, Attached

Maria Maychack
Approved By

2-21-2024
Date

For Research Purposes Only. Not Intended for Food or Drug Use.

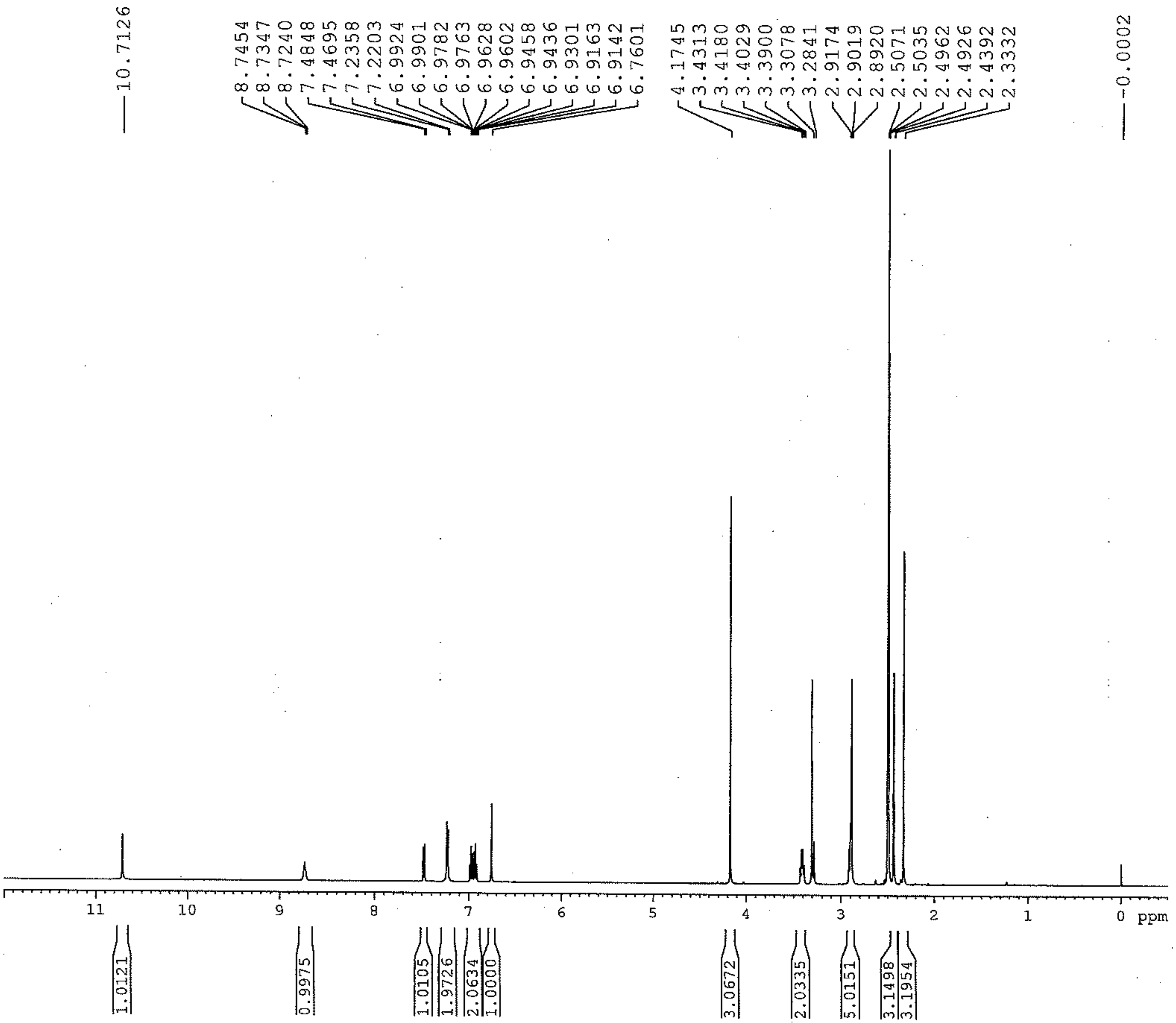

Name [signature]

Date Feb. 15, 2024

NB # JIA-As-91

Current Data Parameters
NAME jia-as-91
EXPNO 10
PROCNO 1

F2 - Acquisition Parameters
Date_ 20240215
Time 12.51 h
INSTRUM Avance Neo
PROBHD Z167419_0029 (
PULPROG zg30
TD 65536
SOLVENT DMSO
NS 40
DS 2
SWH 10000.000 Hz
FIDRES 0.305176 Hz
AQ 3.2767999 sec
RG 101
DW 50.000 usec
DE 11.14 usec
TE 300.0 K
D1 1.00000000 sec
TD0 1
SFO1 500.1330883 MHz
NUC1 1H
P0 2.67 usec
P1 8.00 usec
PLW1 24.22400093 W

F2 - Processing parameters
SI 65536
SF 500.1300043 MHz
WDW EM
SSB 0
LB 0.30 Hz
GB 0
PC 1.00

Openlynx Report
Vial:1:12
Date:13-Feb-2024
Name: [signature]

ID:
Time:09:47:22
Date: Feb. 15, 2024

File:jia-as-91
Notebook: JIA-AS-91

Printed: Tue Feb 13 09:49:45 2024

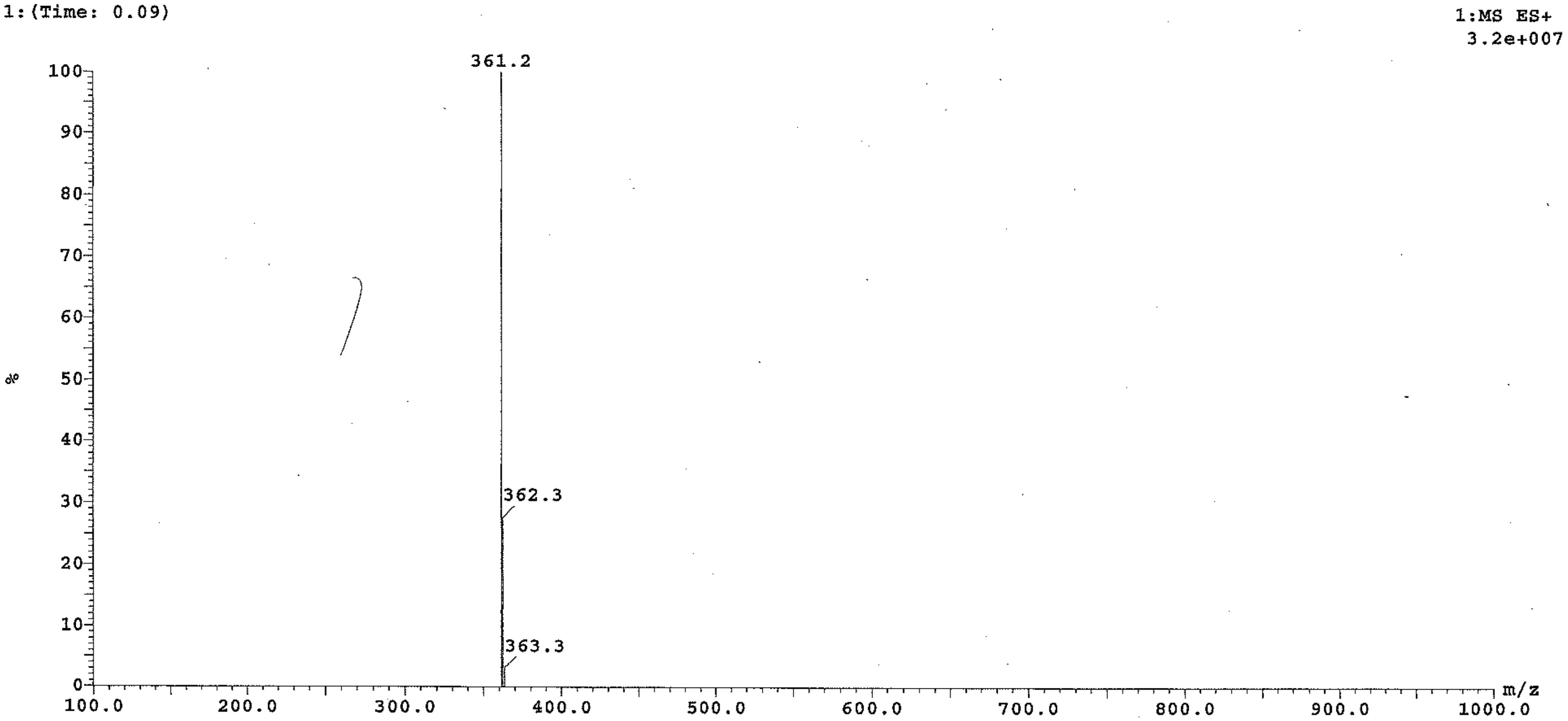

SAMPLE INFORMATION

Sample Name: jia-as-91
Injection Volume: 3.00 ul
Run Time: 9.0 Minutes
Date Acquired: 2/13/2024 1:21:30 PM EST
Date Processed: 2/13/2024 1:54:07 PM EST
Sample Set Name: Template
Acq. Method Set: BEH_C18_PDA_75mm
Processing Method: BEH_C18_PDA
Channel Name: 254nm

Method Notes:
Acquity UPLC BEH C18 1.7u (2.1x75mm)
Flow Rate : 0.5 mL/min
Solvent A : 0.1% TFA in Waters
Solvent B : 0.1% TFA in Acetonitrile
Solvent Gradient Program:

Time (min)	%A	% B
0:00	95	5
6:00	0	100
8:00	0	100
9:00	95	5

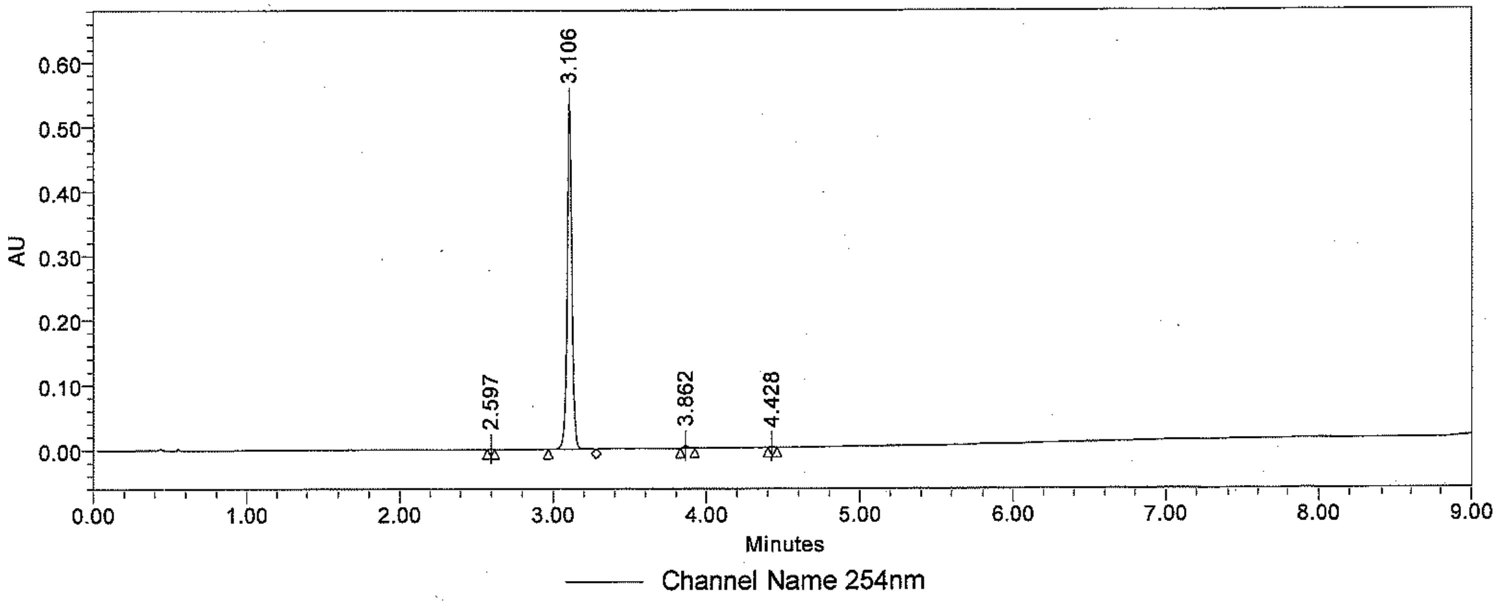

Peak Results

	RT	Area	Int Type	Width (sec)	% Area
1	2.597	996	bb	2.750	0.09
2	3.106	1142288	BV	18.799	99.03
3	3.862	8166	bb	5.500	0.71
4	4.428	1973	bb	3.451	0.17

Name: [signature]

Date: Feb. 15, 2024

NB #: JIA-AS-91

CERTIFICATE OF ANALYSIS

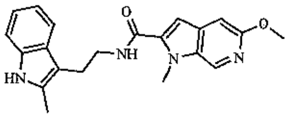

2p. BPN- 37112

Compound Name: BPN-0037112-AA-001
ALB Number: ALB-236036
Batch: 1
Lot Number: ALK-D-28-2
Molecular Formula: $C_{21}H_{22}N_4O_2$
Molecular Weight: 362.42
Last Solvent: Water, Acetonitrile

TEST	RESULT/REFERENCE
Appearance	Off-white Solid
NMR Spectrum	^{1}H, 500 MHz, Dimethyl Sulfoxide-d_6, Consistent - Attached
Mass Spectrum	ESI, *m/z* 363 $[M + H]^+$, Attached
UPLC	>99% (area %), ACQUITY UPLC BEH C18 (2.1 *75) mm, 1.7 micron Column, UV 254 nm Detection, Attached

Maria Maychack
Approved By

8-2-2023
Date

For Research Purposes Only. Not Intended for Food or Drug Use.

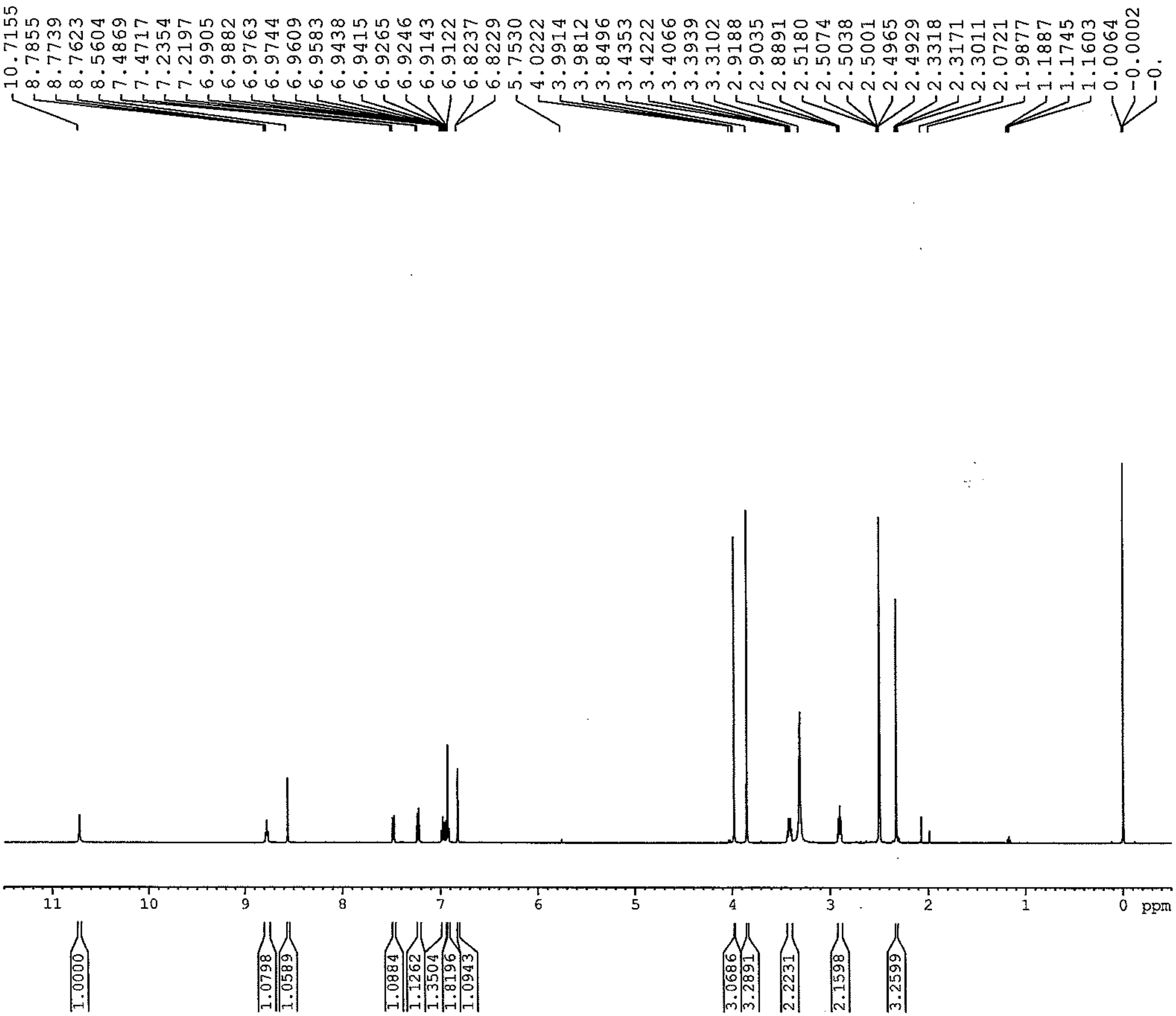

Name Marwah Albaker

Date 25-Jul-2023

NB # ALK-D-28-2

Current Data Parameters
NAME ALK-D-28-2
EXPNO 10
PROCNO 1

F2 - Acquisition Parameters
Date_ 20230725
Time 8.36 h
INSTRUM Avance Neo
PROBHD Z167419_0029 (
PULPROG zg30
TD 65536
SOLVENT DMSO
NS 64
DS 2
SWH 10000.000 Hz
FIDRES 0.305176 Hz
AQ 3.2767999 sec
RG 101
DW 50.000 usec
DE 11.14 usec
TE 300.0 K
D1 1.00000000 sec
TD0 1
SFO1 500.1330883 MHz
NUC1 1H
P0 2.67 usec
P1 8.00 usec
PLW1 24.22400093 W

F2 - Processing parameters
SI 65536
SF 500.1300042 MHz
WDW EM
SSB 0
LB 0.30 Hz
GB 0
PC 1.00

ACQ-SQD#F07SQD100W

24-Jul-2023
07:42:56

Name: Marwah Albaker
Date: 25-Jul-2023
Notebook: ALK-D-28-2

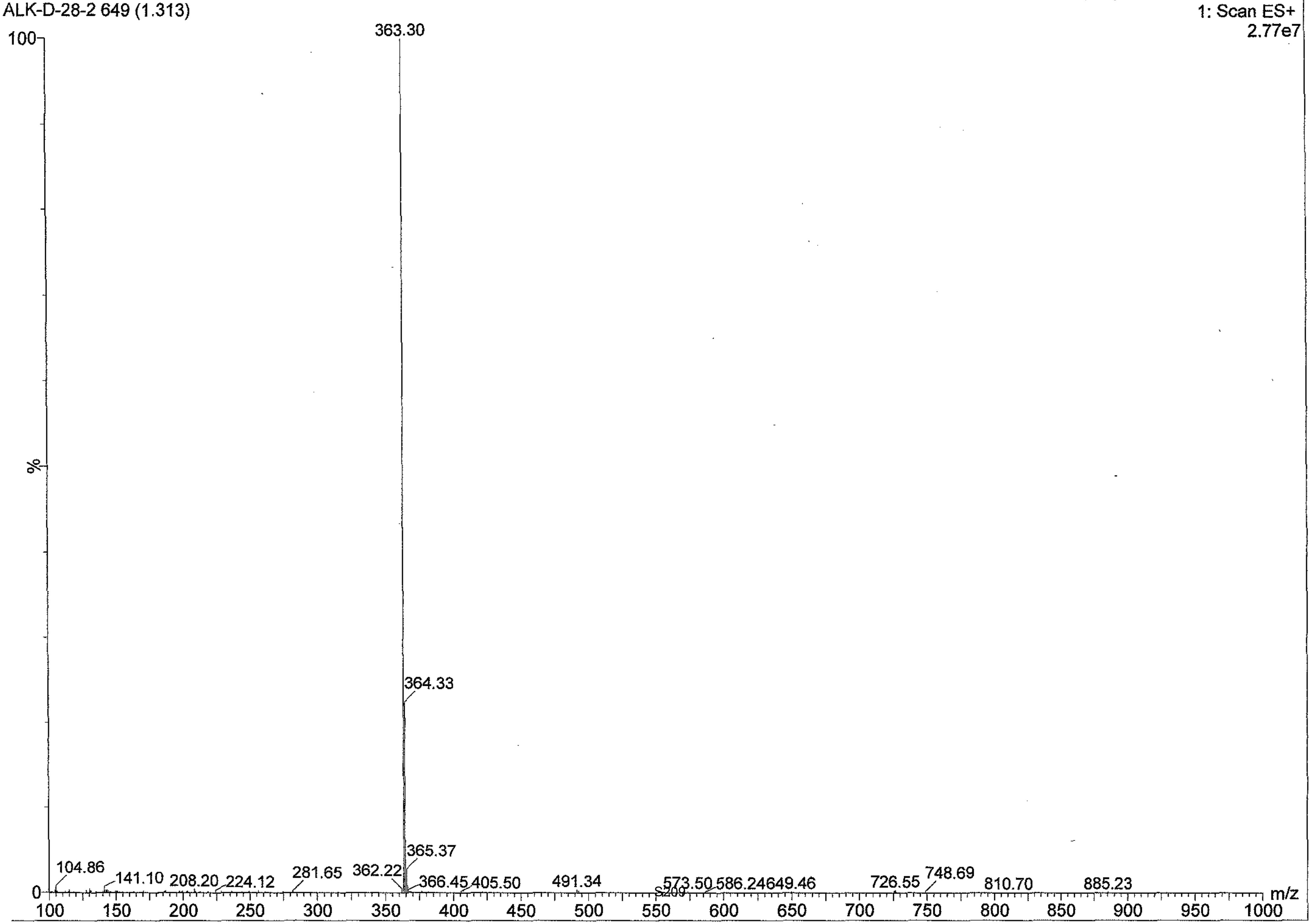

SAMPLE INFORMATION

Sample Name: ALK-D-28-2
Injection Volume: 3.00 ul
Run Time: 9.0 Minutes
Date Acquired: 7/25/2023 7:09:53 AM EDT
Date Processed: 7/25/2023 7:21:55 AM EDT
Sample Set Name: Template
Acq. Method Set: BEH_C18_PDA_75mm
Processing Method: BEH_C18_PDA
Channel Name: 254nm

Method Notes:
Acquity UPLC BEH C18 1.7u (2.1x75mm)
Flow Rate : 0.5 mL/min
Solvent A : 0.1% TFA in Waters
Solvent B : 0.1% TFA in Acetonitrile
Solvent Gradient Program:

Time (min)	%A	% B
0:00	95	5
6:00	0	100
8:00	0	100
9:00	95	5

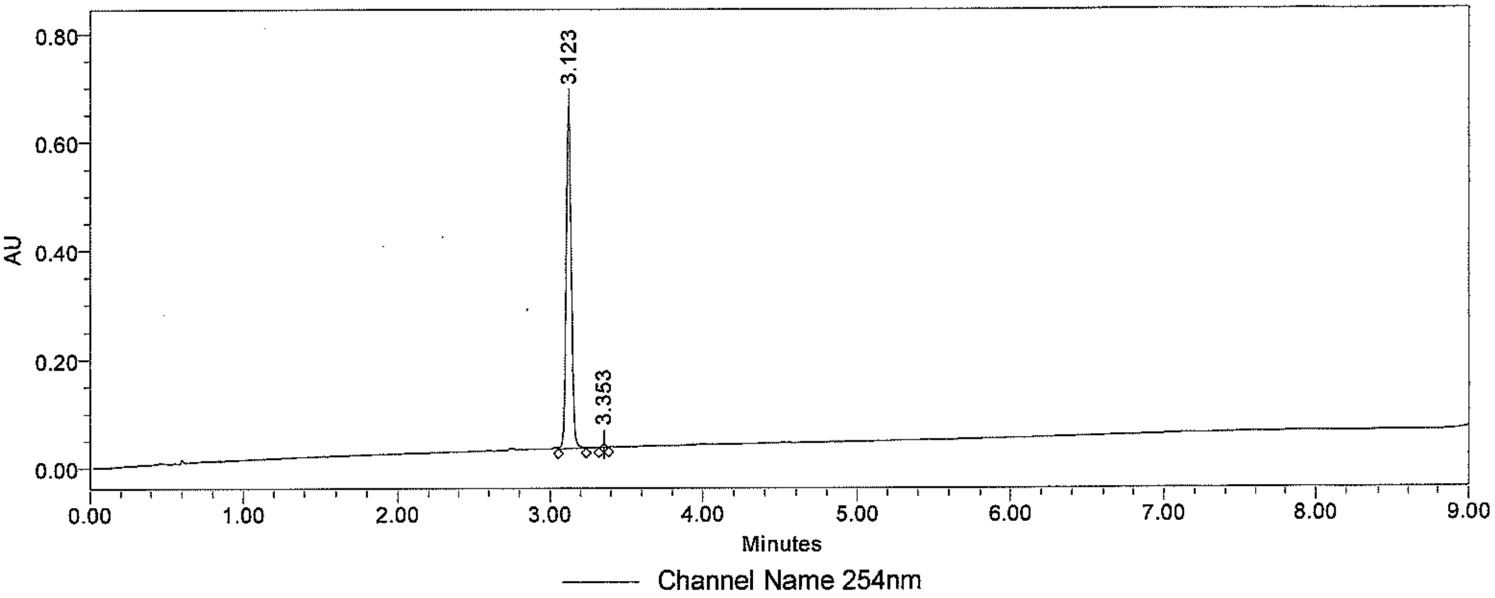

Peak Results

	RT	Area	Int Type	Width (sec)	% Area
1	3.123	1481848	VV	10.999	99.28
2	3.353	10684	VV	3.750	0.72

Name: Marwah Albaker

Date: 25-Jul-2023

NB #: ALK-D-28-2

CERTIFICATE OF ANALYSIS

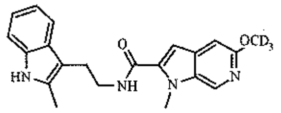

2q. BPN- 37359

Compound Name: BPN-0037359-AA-001
ALB Number: ALB-236972
Batch: 1
Lot Number: CHE-BD-37-2
Molecular Formula: $C_{21}H_{19}D_3N_4O_2$
Molecular Weight: 365.44
Last Solvent: Acetonitrile, Water

TEST	RESULT/REFERENCE
Appearance	White Solid
NMR Spectrum	^{1}H, 500 MHz, Dimethyl Sulfoxide-d_6, Consistent - Attached
Mass Spectrum	ESI, m/z 366 $[M + H]^+$, Attached
UPLC	>99% (area %), ACQUITY UPLC BEH C18 (2.1 *75) mm, 1.7 micron Column, UV 254 nm Detection, Attached

Approved By: [signature]

Date: 10-4-2023

For Research Purposes Only. Not Intended for Food or Drug Use.

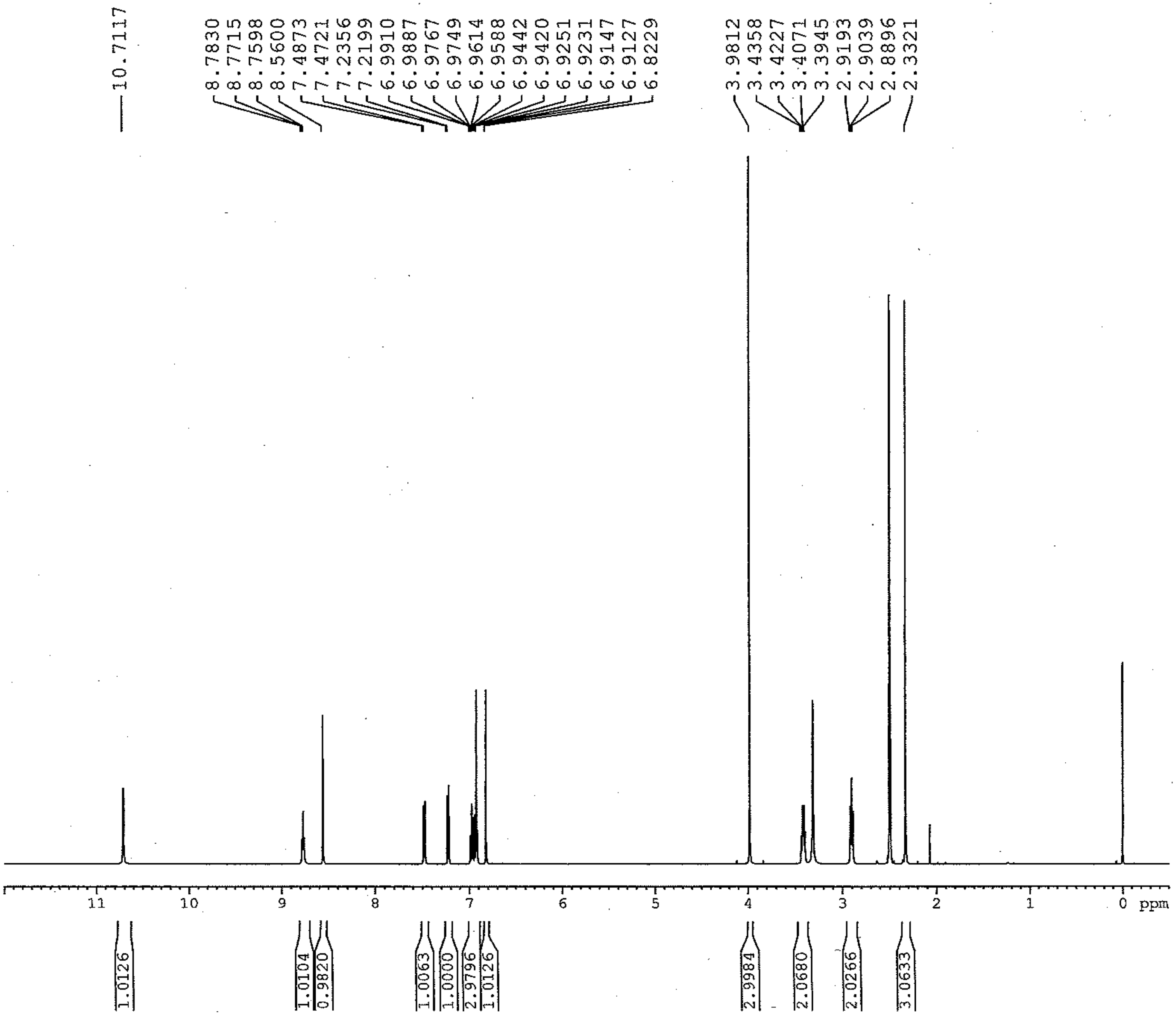

Name Pney Chen
Date Oct 2, 2023
NB # CHE-BD-37-2

Current Data Parameters
NAME che-bd-37-2
EXPNO 10
PROCNO 1

F2 - Acquisition Parameters
Date_ 20231002
Time 9.42 h
INSTRUM Avance Neo
PROBHD Z167419_0029 (
PULPROG zg30
TD 65536
SOLVENT DMSO
NS 64
DS 2
SWH 10000.000 Hz
FIDRES 0.305176 Hz
AQ 3.2767999 sec
RG 101
DW 50.000 usec
DE 11.14 usec
TE 300.0 K
D1 1.00000000 sec
TD0 1
SFO1 500.1330883 MHz
NUC1 1H
P0 2.67 usec
P1 8.00 usec
PLW1 24.22400093 W

F2 - Processing parameters
SI 65536
SF 500.1300041 MHz
WDW EM
SSB 0
LB 0.30 Hz
GB 0
PC 1.00

Openlynx Report

Page 1

Vial:1:46
Date:29-Sep-2023
Name: Ping Chen

ID:
Time:15:33:31
Date: Oct 2, 2023

File:che-bd-37-2
Notebook: CHE-BD-37-2

Printed: Fri Sep 29 15:35:42 2023

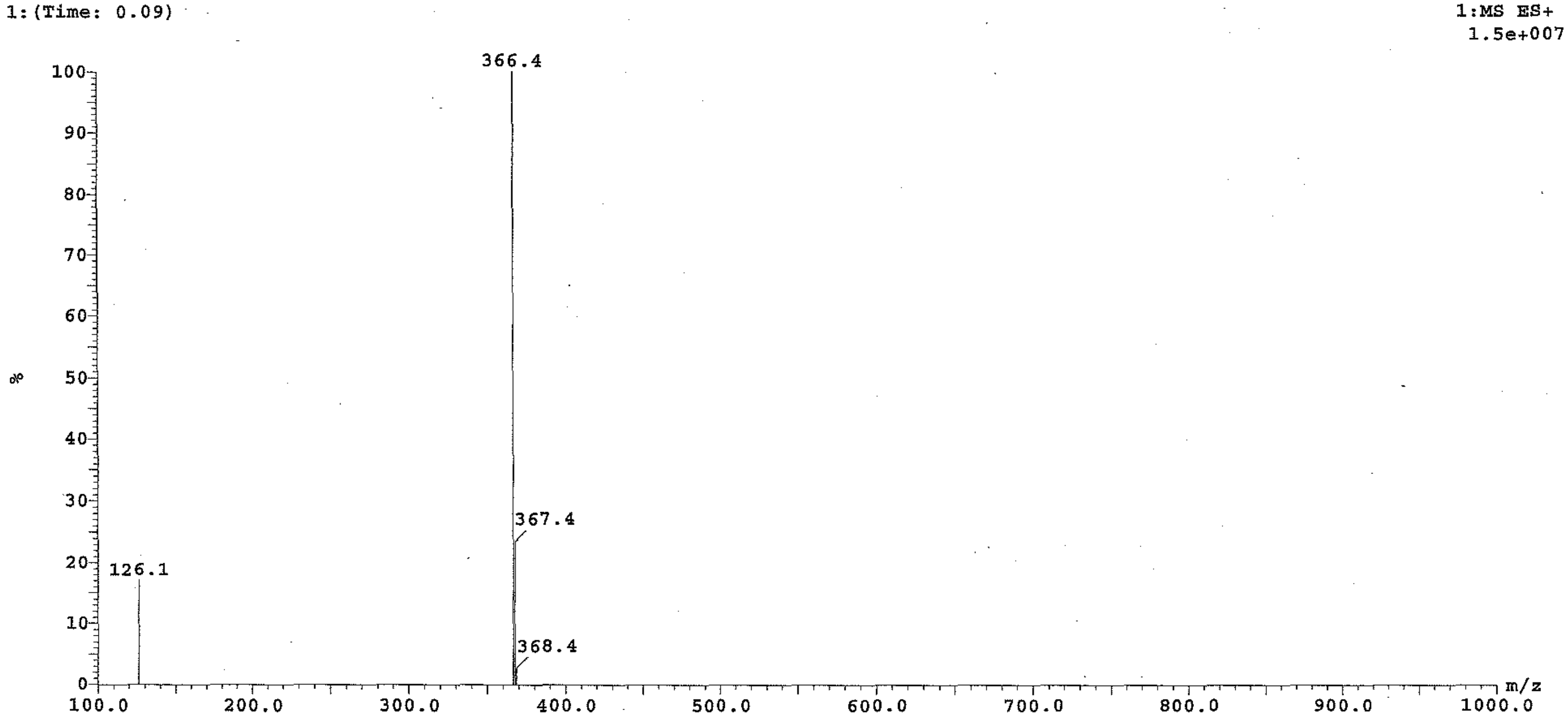

SAMPLE INFORMATION

Sample Name:	che-bd-37-2
Injection Volume:	7.00 ul
Run Time:	9.0 Minutes
Date Acquired:	9/29/2023 3:12:29 PM EDT
Date Processed:	9/29/2023 3:24:31 PM EDT
Sample Set Name:	Template
Acq. Method Set:	BEH_C18_PDA_75mm
Processing Method:	BEH_C18_PDA
Channel Name:	254nm

Method Notes:
Acquity UPLC BEH C18 1.7u (2.1x75mm)
Flow Rate : 0.5 mL/min
Solvent A : 0.1% TFA in Waters
Solvent B : 0.1% TFA in Acetonitrile
Solvent Gradient Program:

Time (min)	%A	% B
0:00	95	5
6:00	0	100
8:00	0	100
9:00	95	5

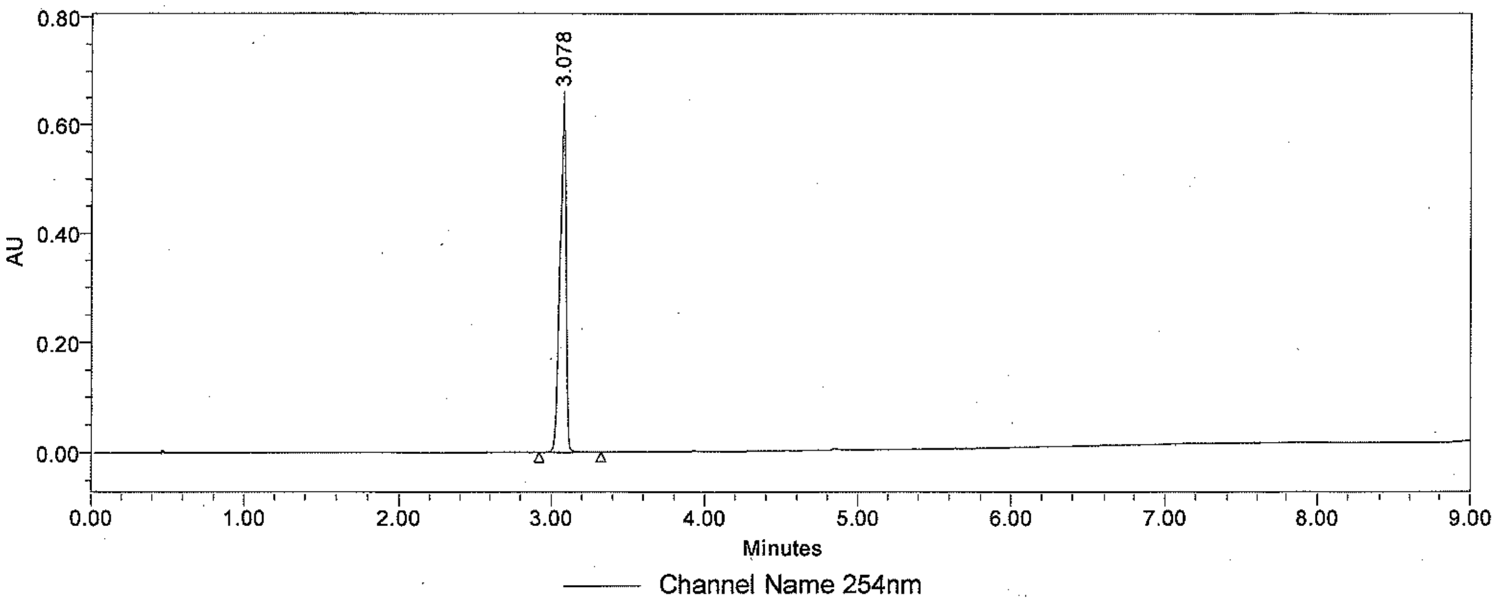

Peak Results

	RT	Area	Int Type	Width (sec)	% Area
1	3.078	1769069	BB	24.048	100.00

Name: Ping Chen

Date: Oct 2, 2023

NB #: CHE-BD-37-2

CERTIFICATE OF ANALYSIS

Compound Name: BPN-0037387-AA-001
ALB Number: ALB-237049
Batch: 1
Lot Number: CHE-BD-41-2
Molecular Formula: $C_{22}H_{24}N_4O_2$
Molecular Weight: 376.45
Last Solvent: Acetonitrile, Water

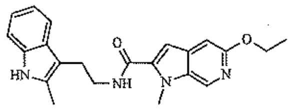

2r. BPN- 37387

TEST	RESULT/REFERENCE
Appearance	White Solid
NMR Spectrum	^1H, 500 MHz, Dimethyl Sulfoxide-d_6, Consistent - Attached
Mass Spectrum	ESI, *m/z* 377 $[M + H]^+$, Attached
UPLC	>99% (area %), ACQUITY UPLC BEH C18 (2.1 *75) mm, 1.7 micron Column, UV 254 nm Detection, Attached

Maria Maychack
Approved By

10 - 11 - 2023
Date

For Research Purposes Only. Not Intended for Food or Drug Use.

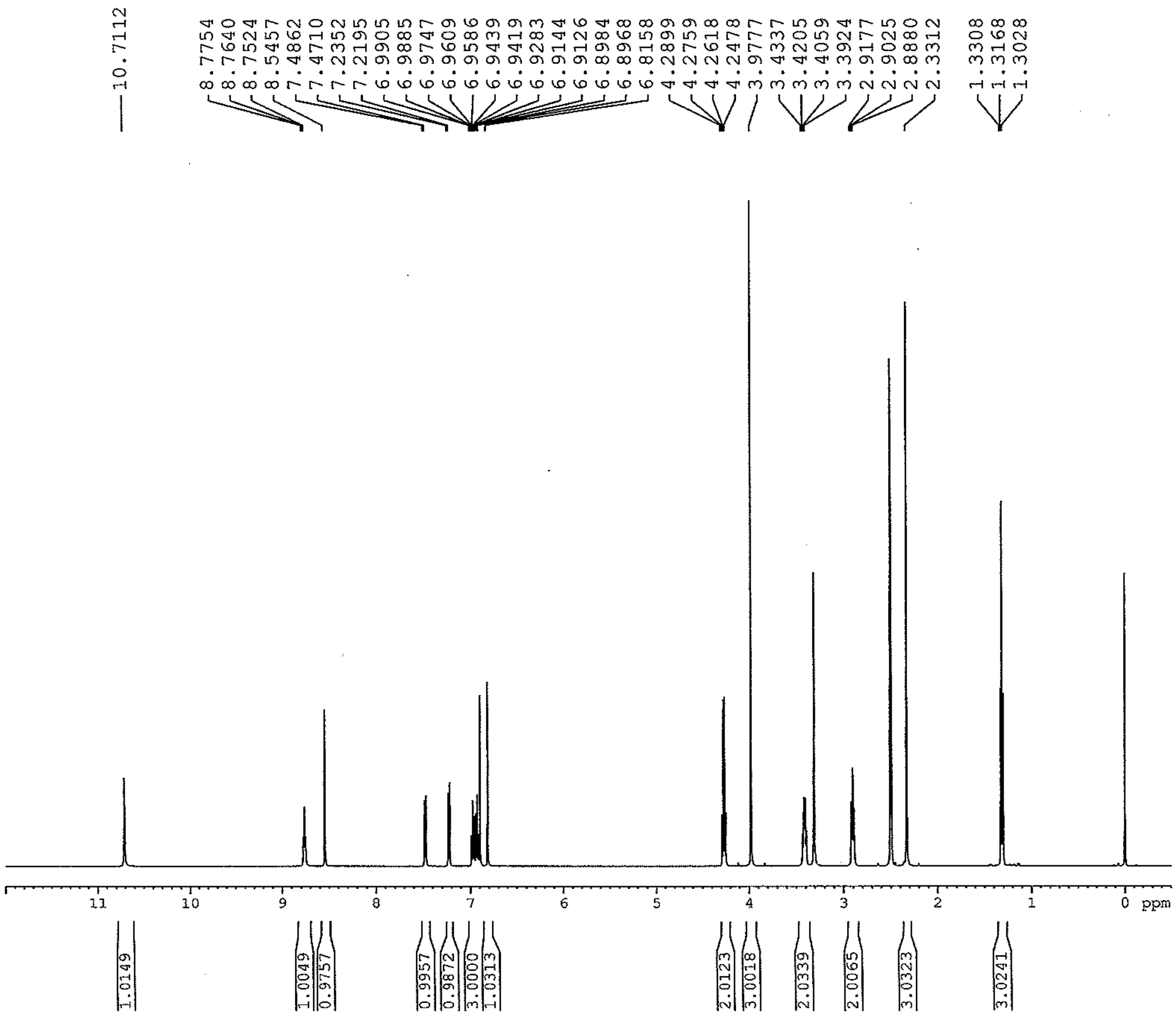

Name Ping Chen

Date Oct 9, 2023

NB # CHE-BD-41-2

Current Data Parameters

NAME	che-bd-41-2
EXPNO	30
PROCNO	1

F2 - Acquisition Parameters

Date_	20231005
Time	10.16 h
INSTRUM	Avance Neo
PROBHD	Z167419_0029 (
PULPROG	zg30
TD	65536
SOLVENT	DMSO
NS	64
DS	2
SWH	10000.000 Hz
FIDRES	0.305176 Hz
AQ	3.2767999 sec
RG	101
DW	50.000 usec
DE	11.14 usec
TE	300.0 K
D1	1.00000000 sec
TD0	1
SFO1	500.1330883 MHz
NUC1	1H
P0	2.67 usec
P1	8.00 usec
PLW1	24.22400093 W

F2 - Processing parameters

SI	65536
SF	500.1300041 MHz
WDW	EM
SSB	0
LB	0.30 Hz
GB	0
PC	1.00

Openlynx Report

Page 1

Vial:1:3

Date:02-Oct-2023

ID:

Time:09:19:00

File:che-bd-41-2

Name: Ping Chen

Date: Oct 9, 2023

Notebook: CHE-BD-41-2

Printed: Mon Oct 02 09:21:22 2023

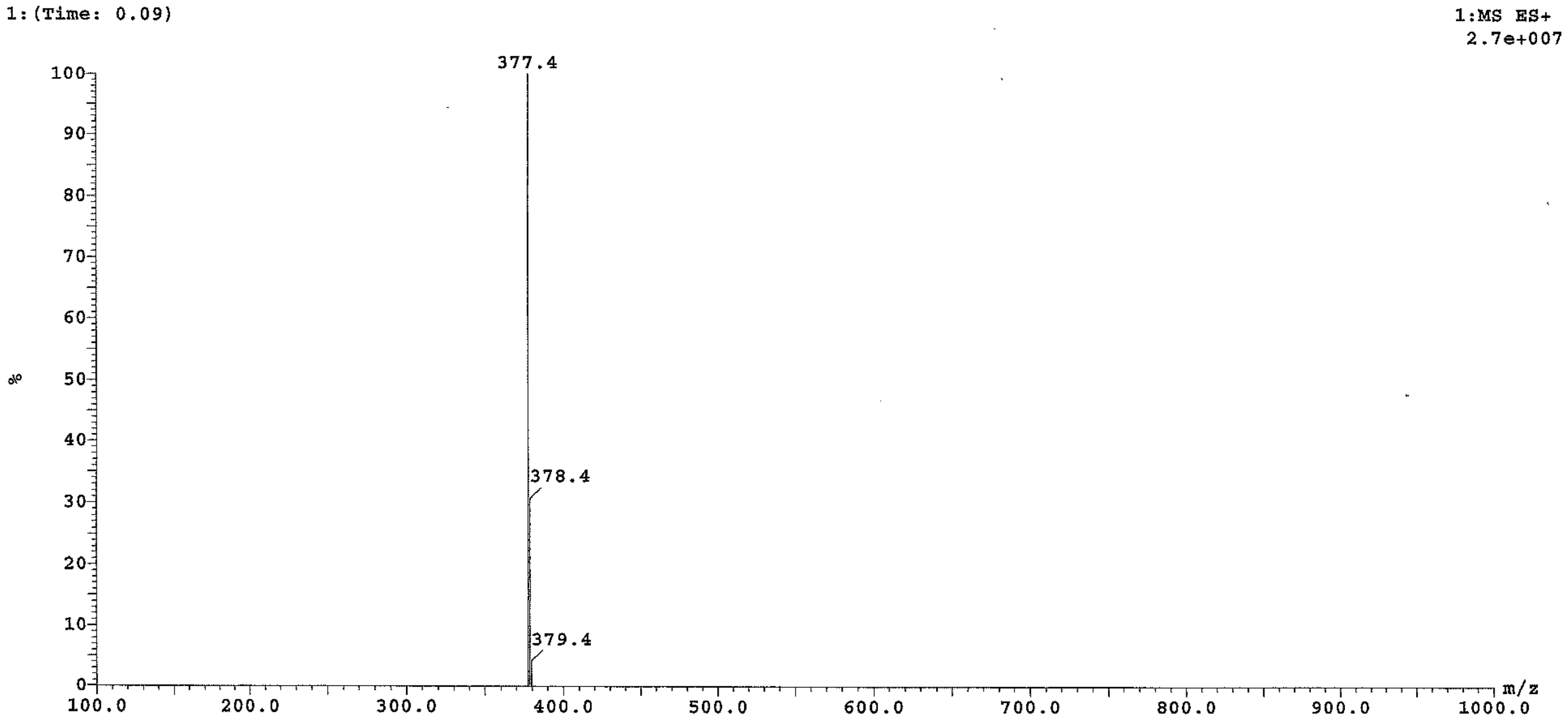

SAMPLE INFORMATION

Sample Name:	che-bd-41-2
Injection Volume:	3.00 ul
Run Time:	9.0 Minutes
Date Acquired:	10/2/2023 9:01:06 AM EDT
Date Processed:	10/2/2023 9:13:08 AM EDT
Sample Set Name:	Template
Acq. Method Set:	BEH_C18_PDA_75mm
Processing Method:	BEH_C18_PDA
Channel Name:	254nm

Method Notes:
Acquity UPLC BEH C18 1.7u (2.1x75mm)
Flow Rate : 0.5 mL/min
Solvent A : 0.1% TFA in Waters
Solvent B : 0.1% TFA in Acetonitrile
Solvent Gradient Program:

Time (min)	%A	% B
0:00	95	5
6:00	0	100
8:00	0	100
9:00	95	5

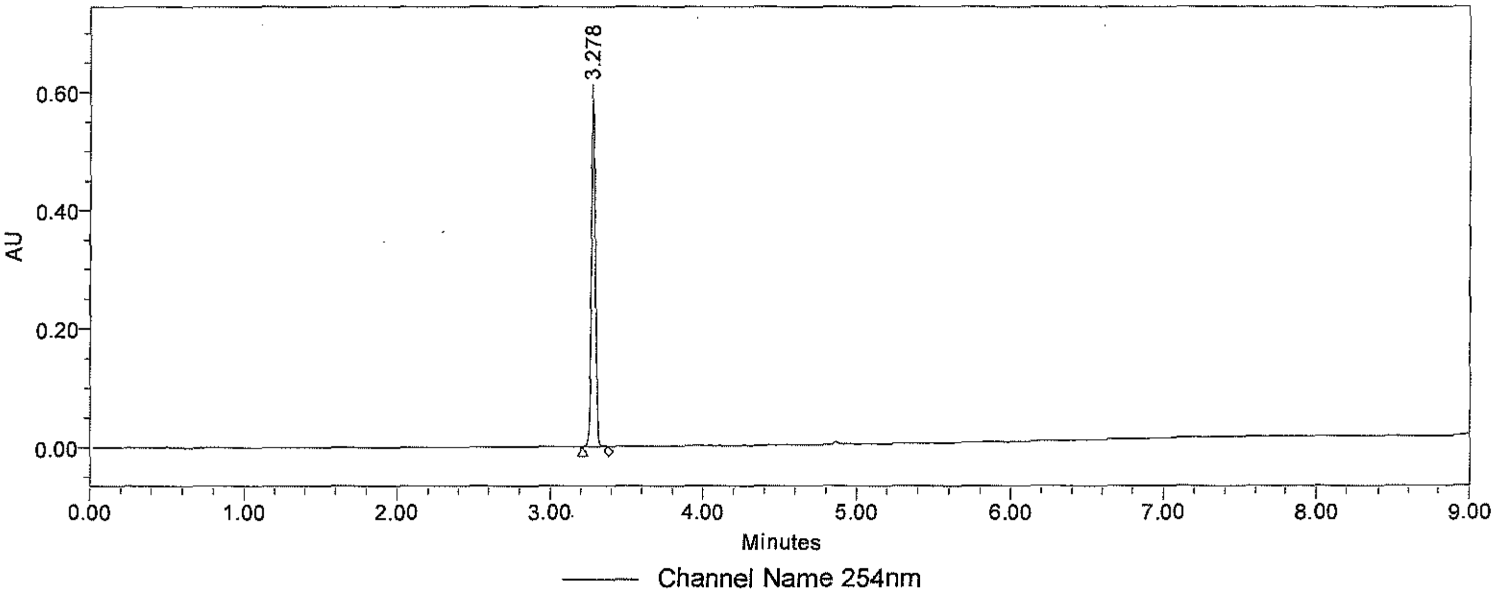

Peak Results

	RT	Area	Int Type	Width (sec)	% Area
1	3.278	1051334	BV	10.099	100.00

Name: Ping Chen

Date: Oct 9, 2023

NB #: CHE-BD-41-2

CERTIFICATE OF ANALYSIS

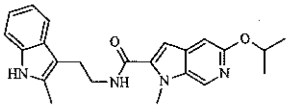

2s. BPN- 37409

Compound Name: BPN-0037409-AA-001
ALB Number: ALB-237201
Batch: 1
Lot Number: CHE-BD-50-2
Molecular Formula: $C_{23}H_{26}N_4O_2$
Molecular Weight: 390.48
Last Solvent: Acetonitrile, Water

**TEST**	**RESULT/REFERENCE**
Appearance	White Solid
NMR Spectrum	^{1}H, 500 MHz, Dimethyl Sulfoxide-d_6, Consistent - Attached
Mass Spectrum	ESI, *m/z* 391 $[M + H]^+$, Attached
UPLC	>99% (area %), ACQUITY UPLC BEH C18 (2.1 *75) mm, 1.7 micron Column, UV 254 nm Detection, Attached

[signature]
Approved By

10-18-2023
Date

For Research Purposes Only. Not Intended for Food or Drug Use.

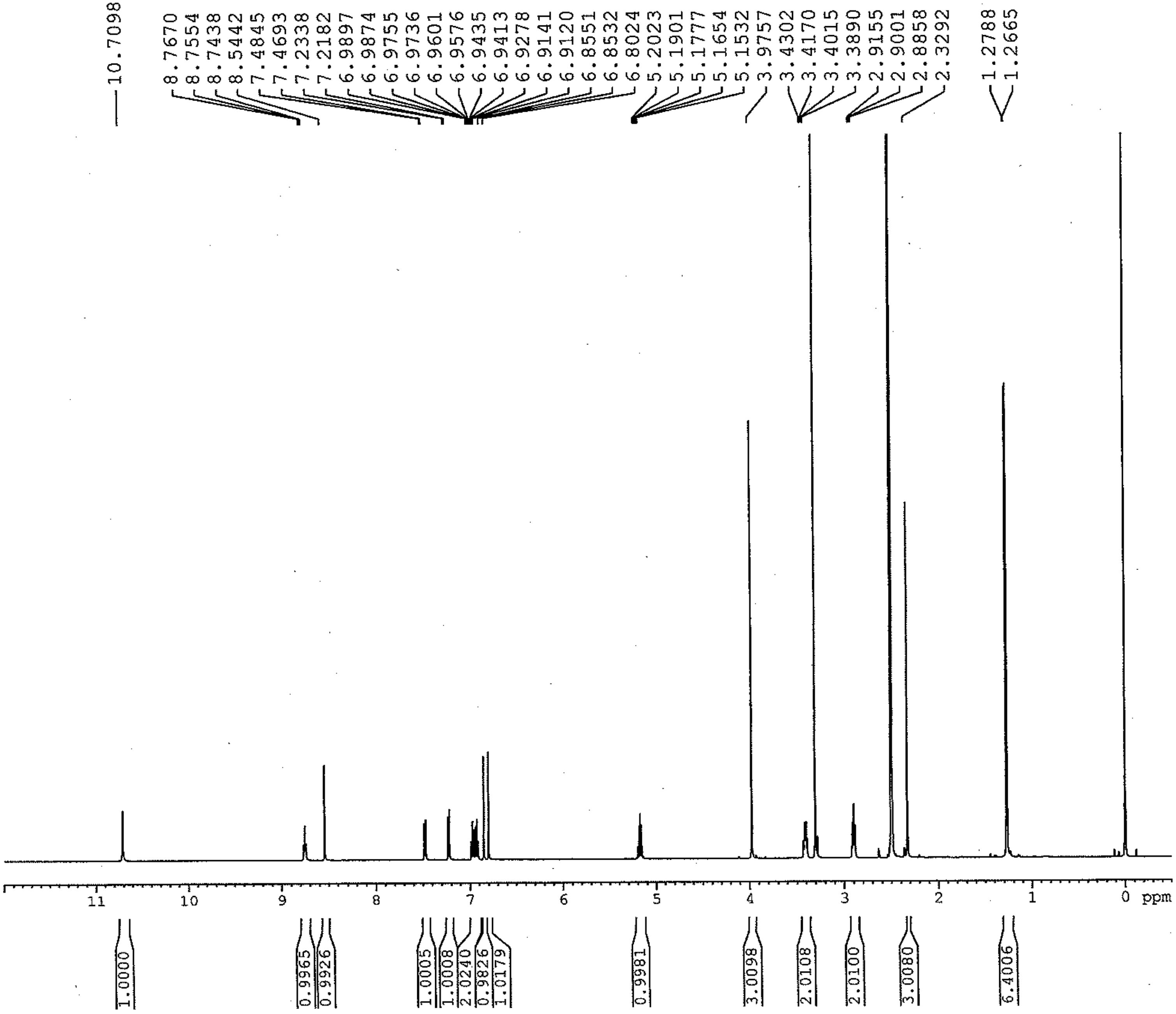

Name Pingzhu
Date Oct 16, 2023
NB # CHE-BD-50-2

Current Data Parameters
NAME che-bd-50-2
EXPNO 10
PROCNO 1

F2 - Acquisition Parameters
Date_ 20231011
Time 15.45 h
INSTRUM Avance Neo
PROBHD Z167419_0029 (
PULPROG zg30
TD 65536
SOLVENT DMSO
NS 128
DS 2
SWH 10000.000 Hz
FIDRES 0.305176 Hz
AQ 3.2767999 sec
RG 101
DW 50.000 usec
DE 11.14 usec
TE 300.0 K
D1 1.00000000 sec
TD0 1
SFO1 500.1330883 MHz
NUC1 1H
P0 2.67 usec
P1 8.00 usec
PLW1 24.22400093 W

F2 - Processing parameters
SI 65536
SF 500.1300042 MHz
WDW EM
SSB 0
LB 0.30 Hz
GB 0
PC 1.00

Openlynx Report Page 1

Vial:1:31 ID: File:CHE-BD-50-2

Date:11-Oct-2023 Time:14:25:06

Name: Pinge hen Date: Oct 16, 2023 Notebook: CHE-BD-50-2

Printed: Wed Oct 11 14:27:32 2023

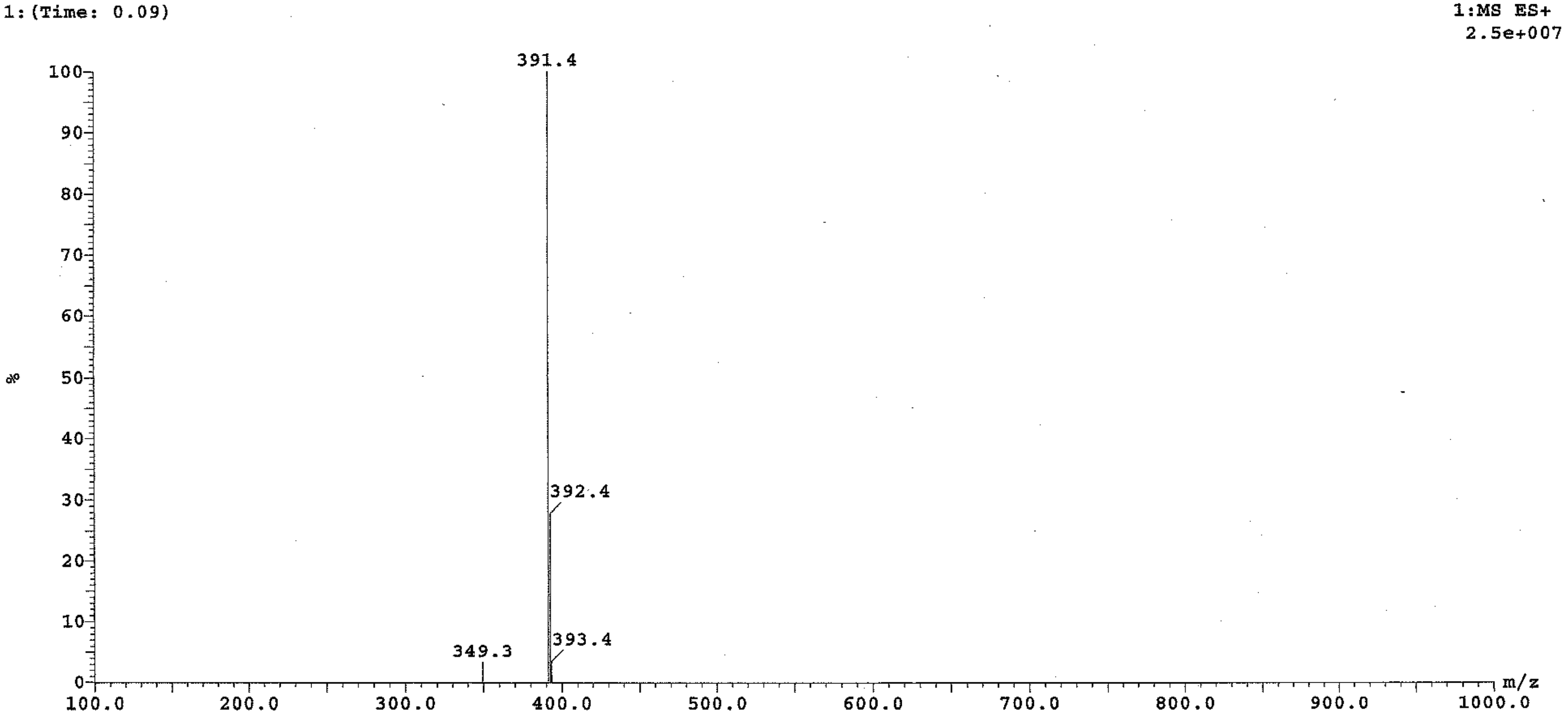

SAMPLE INFORMATION

Sample Name: ~~che-bd-50-1~~ CHE-BD-50-2
Injection Volume: 6.00 ul PC 10/16/23
Run Time: 9.0 Minutes
Date Acquired: 10/11/2023 2:06:56 PM EDT
Date Processed: 10/11/2023 2:18:57 PM EDT
Sample Set Name: Template
Acq. Method Set: BEH_C18_PDA_75mm
Processing Method: BEH_C18_PDA
Channel Name: 254nm

Method Notes:
Acquity UPLC BEH C18 1.7u (2.1x75mm)
Flow Rate : 0.5 mL/min
Solvent A : 0.1% TFA in Waters
Solvent B : 0.1% TFA in Acetonitrile
Solvent Gradient Program:

Time (min)	%A	% B
0:00	95	5
6:00	0	100
8:00	0	100
9:00	95	5

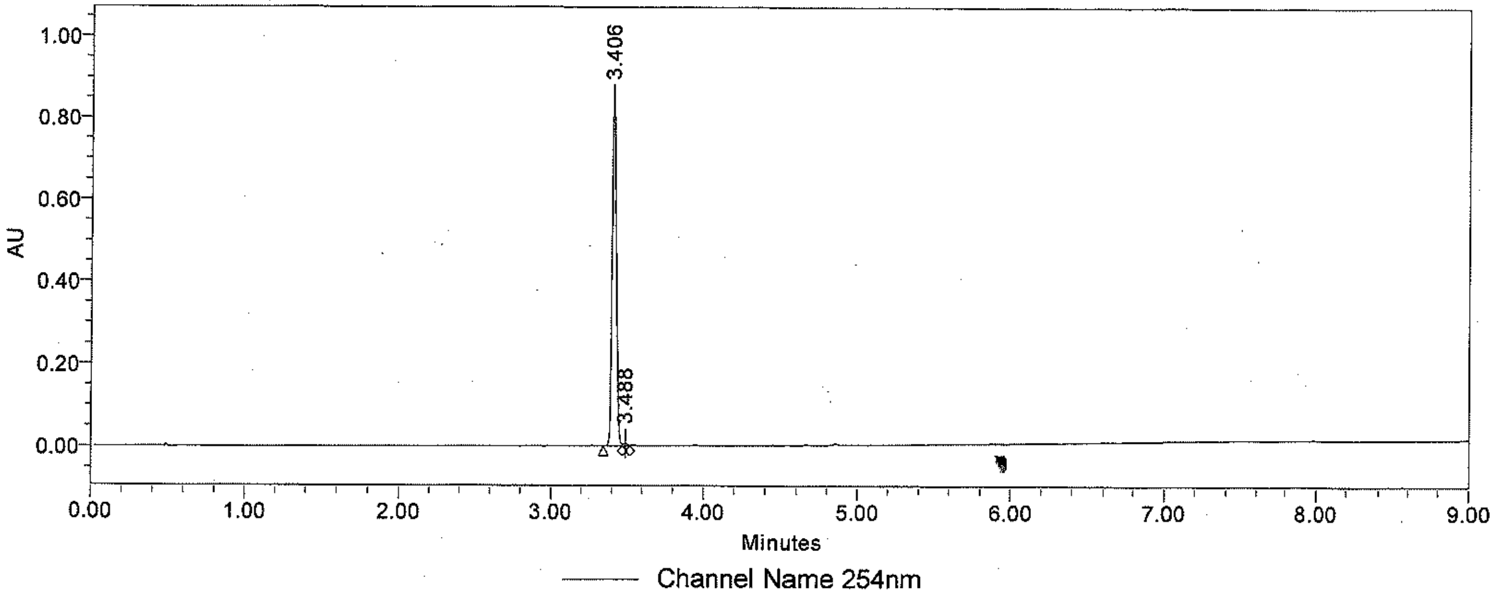

Peak Results

	RT	Area	Int Type	Width (sec)	% Area
1	3.406	1622909	BV	7.549	99.31
2	3.488	11249	VV	2.900	0.69

Name: Pingchen
Date: Oct 16, 2023
NB #: CHE-BD-50-2

CERTIFICATE OF ANALYSIS

Compound Name: BPN-0037360-AA-001
ALB Number: ALB-236969
Batch: 1
Lot Number: CHE-BD-31-2
Molecular Formula: $C_{25}H_{30}N_4O_4$
Molecular Weight: 450.53
Last Solvent: Acetonitrile, Water

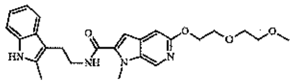

2t. BPN- 37360

TEST	RESULT/REFERENCE
Appearance	White Solid
NMR Spectrum	^{1}H, 500 MHz, Dimethyl Sulfoxide-d_6, Consistent - Attached
Mass Spectrum	ESI, *m/z* 451 $[M + H]^+$, Attached
UPLC	>99% (area %), ACQUITY UPLC BEH C18 (2.1 *75) mm, 1.7 micron Column, UV 254 nm Detection, Attached

Approved By

10-4-2023
Date

For Research Purposes Only. Not Intended for Food or Drug Use.

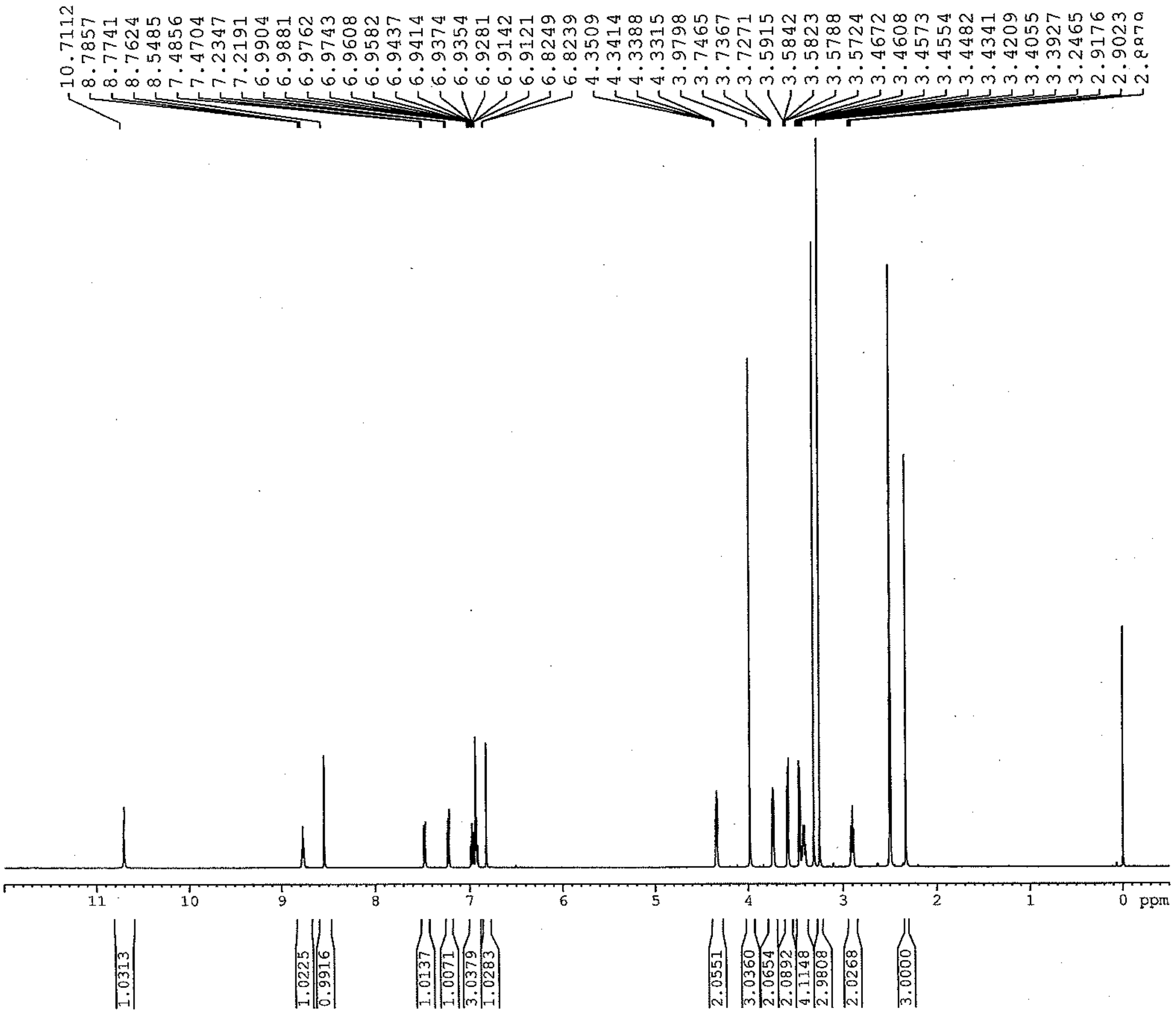

Name Ping Chen

Date Oct 2, 2023

NB # CHE-BD-31-2

Current Data Parameters
NAME che-bd-31-2
EXPNO 10
PROCNO 1

F2 - Acquisition Parameters
Date_ 20230929
Time 8.43 h
INSTRUM Avance Neo
PROBHD Z167419_0029 (
PULPROG zg30
TD 65536
SOLVENT DMSO
NS 64
DS 2
SWH 10000.000 Hz
FIDRES 0.305176 Hz
AQ 3.2767999 sec
RG 101
DW 50.000 usec
DE 11.14 usec
TE 300.0 K
D1 1.00000000 sec
TD0 1
SFO1 500.1330883 MHz
NUC1 1H
P0 2.67 usec
P1 8.00 usec
PLW1 24.22400093 W

F2 - Processing parameters
SI 65536
SF 500.1300043 MHz
WDW EM
SSB 0
LB 0.30 Hz
GB 0
PC 1.00

Openlynx Report
Vial:1:22
Date:28-Sep-2023
Name: Ping Chen

ID:
Time:09:52:28
Date: Oct 2, 2023

File:che-bd-31-2
Notebook: CHE-BD-31-2

Printed: Thu Sep 28 09:54:44 2023

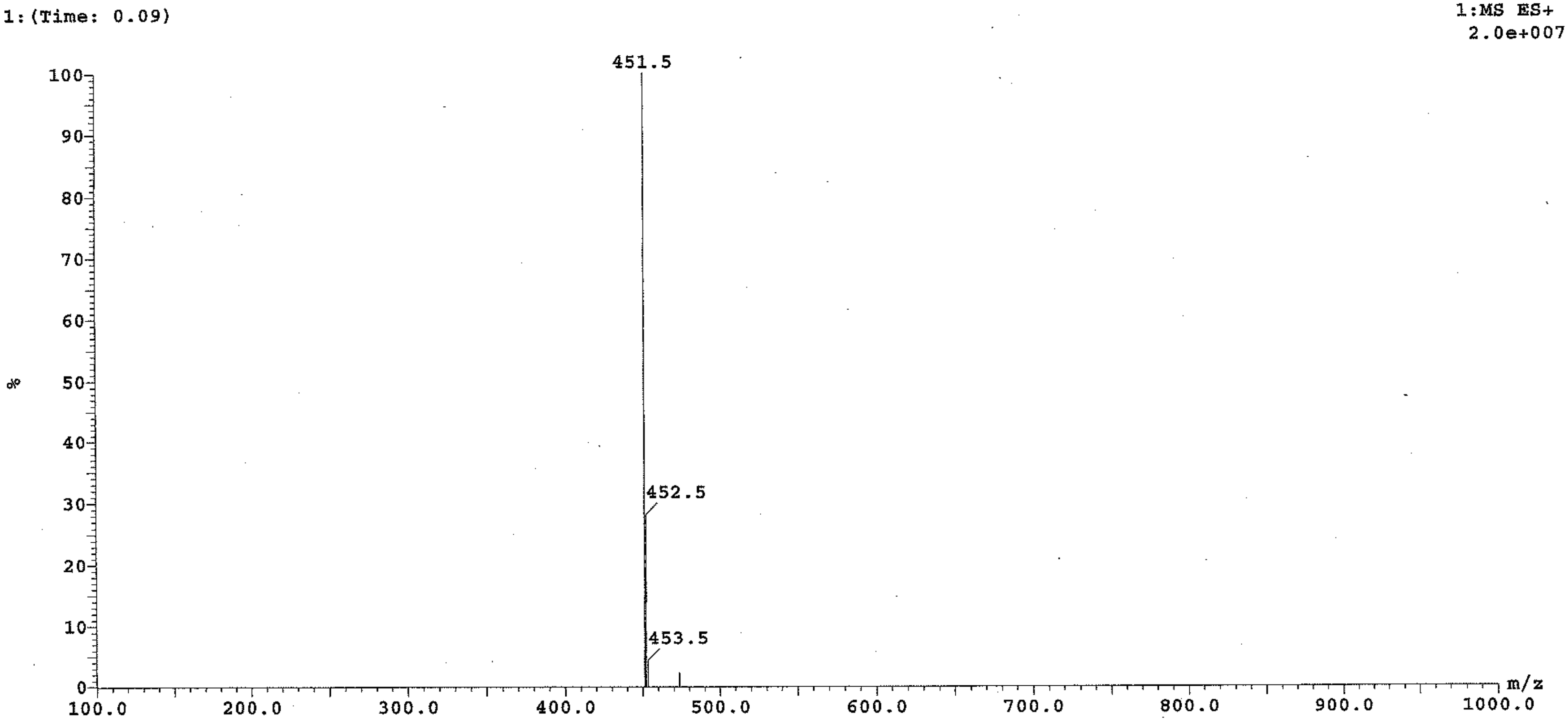

SAMPLE INFORMATION

Sample Name:	che-bd-31-2
Injection Volume:	10.00 ul
Run Time:	9.0 Minutes
Date Acquired:	9/28/2023 9:34:20 AM EDT
Date Processed:	9/28/2023 9:46:22 AM EDT
Sample Set Name:	Template
Acq. Method Set:	BEH_C18_PDA_75mm
Processing Method:	BEH_C18_PDA
Channel Name:	254nm

Method Notes:
Acquity UPLC BEH C18 1.7u (2.1x75mm)
Flow Rate : 0.5 mL/min
Solvent A : 0.1% TFA in Waters
Solvent B : 0.1% TFA in Acetonitrile
Solvent Gradient Program:

Time (min)	%A	% B
0:00	95	5
6:00	0	100
8:00	0	100
9:00	95	5

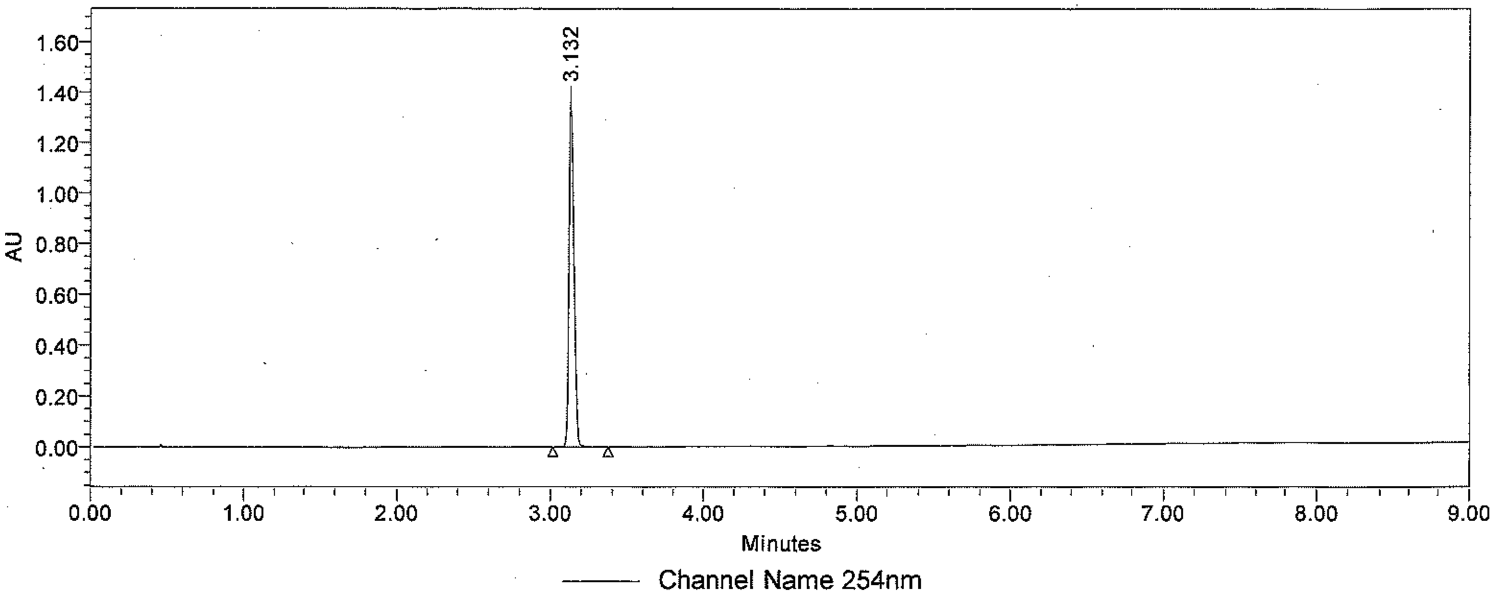

Peak Results

	RT	Area	Int Type	Width (sec)	% Area
1	3.132	3029274	BB	21.498	100.00

Name: Ping Chen

Date: Oct 2, 2023

NB #: CHE-BD-31-2

CERTIFICATE OF ANALYSIS

Compound Name: BPN-0037440-AA-001
ALB Number: ALB-237280
Batch: 1
Lot Number: ALK-D-100-2
Molecular Formula: $C_{21}H_{22}N_4O$
Molecular Weight: 346.43
Last Solvent: Acetonitrile, Water

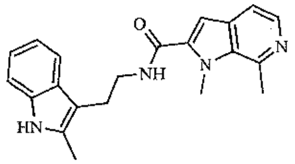

2u. BPN- 37440

TEST	RESULT/REFERENCE
Appearance	Off-white Solid
NMR Spectrum	^{1}H, 500 MHz, Dimethyl Sulfoxide-d_6, Consistent - Attached
Mass Spectrum	ESI, m/z 347 $[M + H]^+$, Attached
UPLC	98.4% (area %), ACQUITY UPLC BEH C18 (2.1 *75) mm, 1.7 micron Column, UV 254 nm Detection, Attached

Approved By

10-25-2023
Date

For Research Purposes Only. Not Intended for Food or Drug Use.

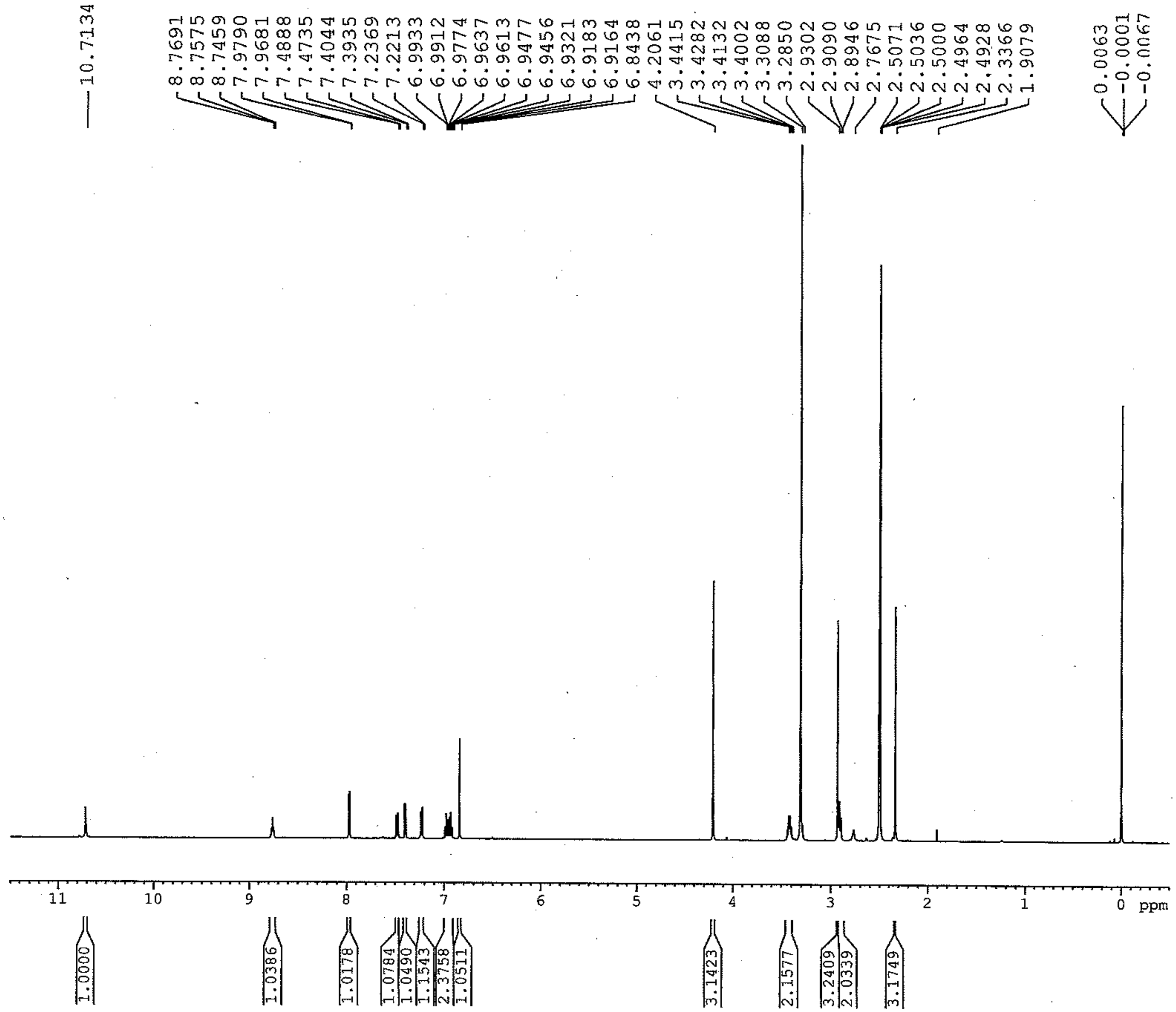

Name Marwah Albaker
Date 17-Oct-2023
NB # ALK-D-100-2

Current Data Parameters
NAME ALK-D-100-2
EXPNO 10
PROCNO 1

F2 - Acquisition Parameters
Date_ 20231017
Time 9.01 h
INSTRUM Avance Neo
PROBHD Z167419_0029 (
PULPROG zg30
TD 65536
SOLVENT DMSO
NS 64
DS 2
SWH 10000.000 Hz
FIDRES 0.305176 Hz
AQ 3.2767999 sec
RG 101
DW 50.000 usec
DE 11.14 usec
TE 300.0 K
D1 1.00000000 sec
TD0 1
SFO1 500.1330883 MHz
NUC1 1H
P0 2.67 usec
P1 8.00 usec
PLW1 24.22400093 W

F2 - Processing parameters
SI 65536
SF 500.1300042 MHz
WDW EM
SSB 0
LB 0.30 Hz
GB 0
PC 1.00

ACQ-SQD#F07SQD100W

17-Oct-2023
08:03:14

Name: Marwah Albaker
Date: 17-Oct-2023
Notebook: ALK-D-100-2

ALK-D-100-2 565 (1.134)

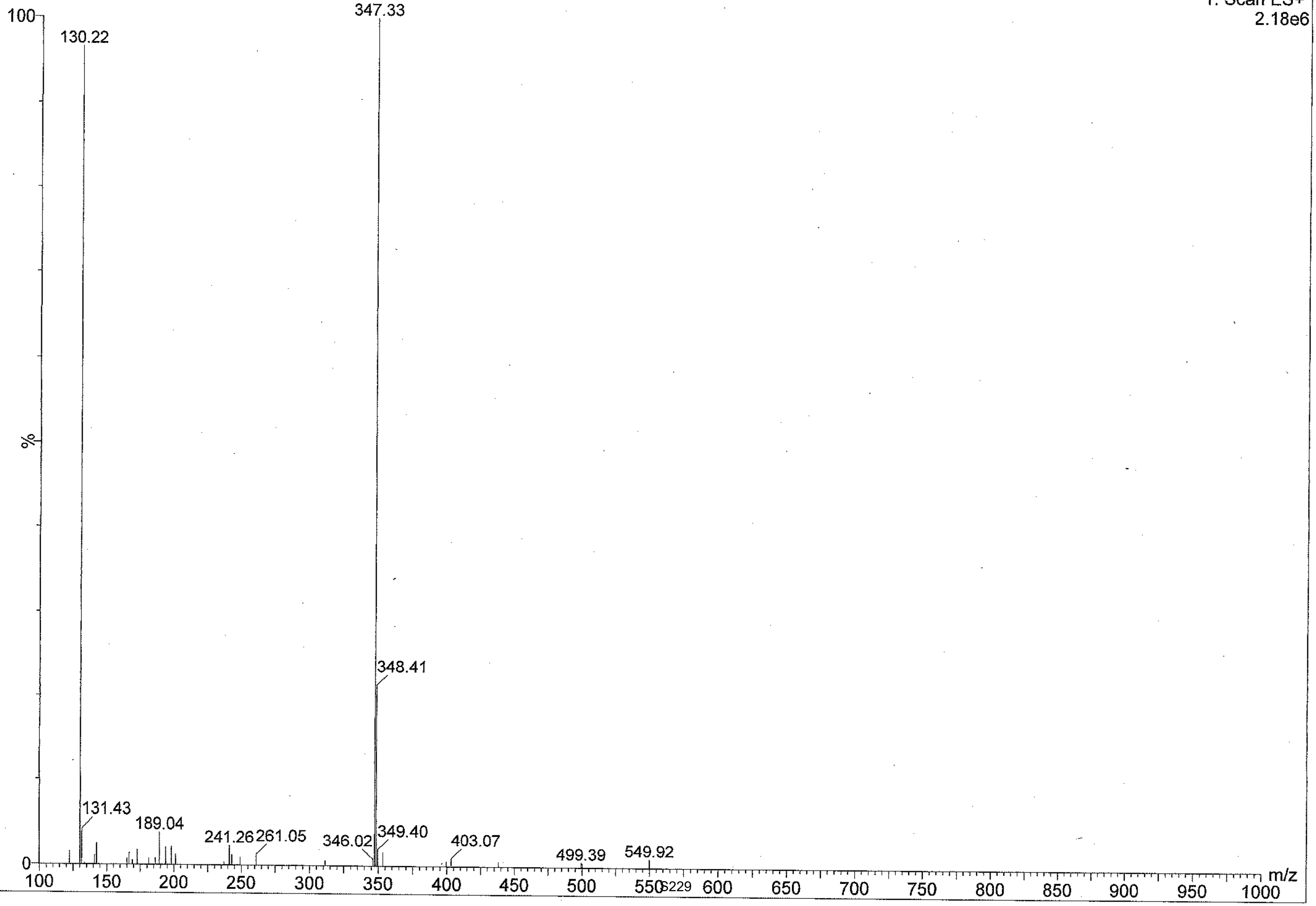

SAMPLE INFORMATION

Sample Name: ALK-D-100-2
Injection Volume: 1.50 ul
Run Time: 9.0 Minutes
Date Acquired: 10/17/2023 7:55:24 AM EDT
Date Processed: 10/17/2023 8:09:52 AM EDT
Sample Set Name: Template
Acq. Method Set: BEH_C18_PDA_75mm
Processing Method: BEH_C18_PDA
Channel Name: 254nm

Method Notes:
Acquity UPLC BEH C18 1.7u (2.1x75mm)
Flow Rate : 0.5 mL/min
Solvent A : 0.1% TFA in Waters
Solvent B : 0.1% TFA in Acetonitrile
Solvent Gradient Program:

Time (min)	%A	% B
0:00	95	5
6:00	0	100
8:00	0	100
9:00	95	5

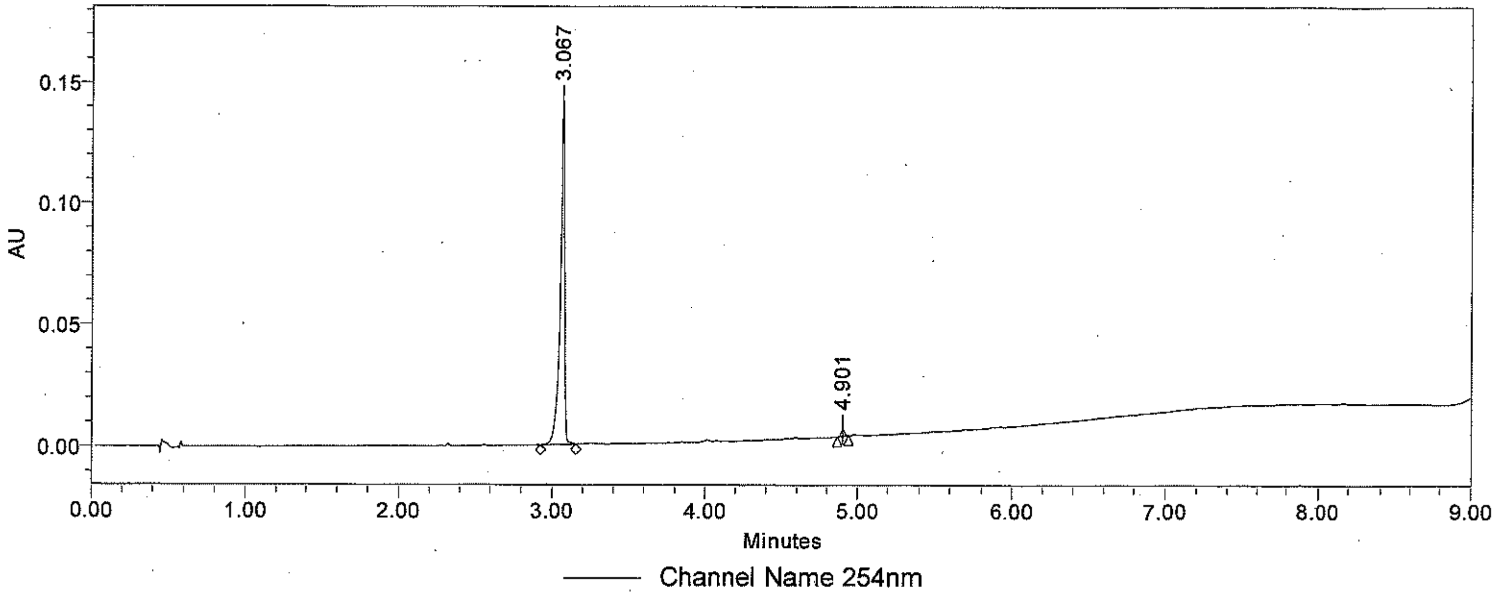

Peak Results

	RT	Area	Int Type	Width (sec)	% Area
1	3.067	271104	VV	13.849	98.47
2	4.901	4199	bb	4.401	1.53

Name: Marwah Albaker

Date: 17-Oct-2023

NB #: ALK-D-100-2

CERTIFICATE OF ANALYSIS

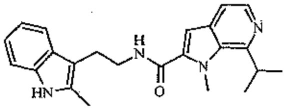

2v. BPN- 37638

Compound Name: BPN-0037638-AA-001
ALB Number: ALB-238319
Batch: 1
Lot Number: CRA-A-196-2
Molecular Formula: $C_{23}H_{26}N_4O$
Molecular Weight: 374.48
Last Solvent: Acetonitrile, Water

TEST	RESULT/REFERENCE
Appearance	White Solid
NMR Spectrum	^{1}H, 500 MHz, Dimethyl Sulfoxide-d_6, Consistent - Attached
Mass Spectrum	ESI, *m/z* 375 $[M + H]^+$, Attached
UPLC	>99% (area %), ACQUITY UPLC BEH C18 (2.1 *75) mm, 1.7 micron Column, UV 254 nm Detection, Attached

Maria Maychack
Approved By

1-24-2024
Date

For Research Purposes Only. Not Intended for Food or Drug Use.

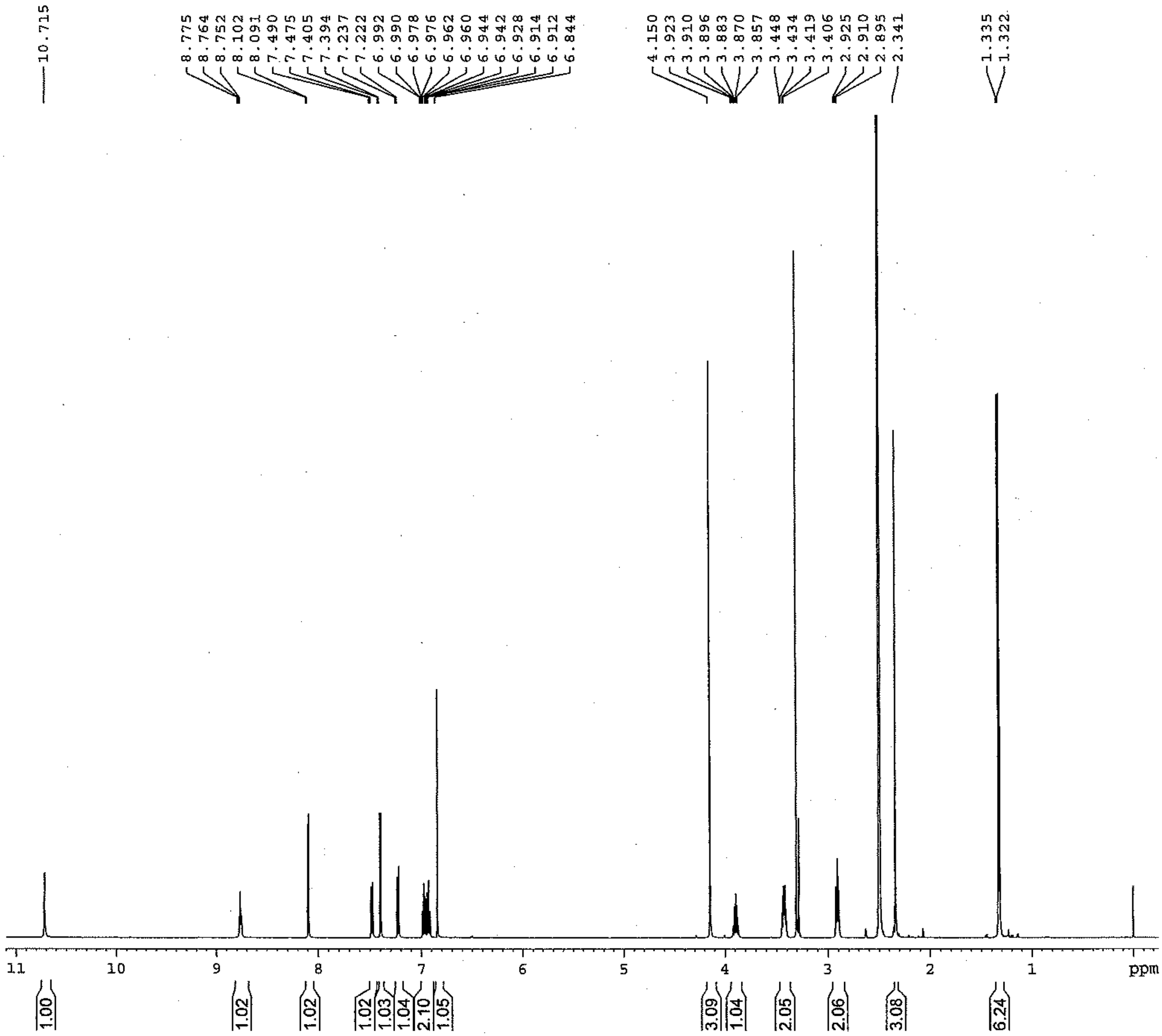

Name Ethan Cramer

Date 22 Jan 2024

NB # CRA-A-196-2

NAME	CRA-A-196-2
EXPNO	10
PROCNO	1
Date_	20240115
Time	9.56 h
INSTRUM	Avance Neo
PROBHD	Z167419_0029 (
PULPROG	zg30
TD	65536
SOLVENT	DMSO
NS	32
DS	2
SWH	10000.000 Hz
FIDRES	0.305176 Hz
AQ	3.2768500 sec
RG	101
DW	50.000 usec
DE	11.14 usec
TE	300.0 K
D1	1.00000000 sec
TD0	1
SFO1	500.1330883 MHz
NUC1	1H
P0	2.67 usec
P1	8.00 usec
SI	65536
SF	500.1300043 MHz
WDW	EM
SSB	0
LB	0.30 Hz
GB	0
PC	1.00

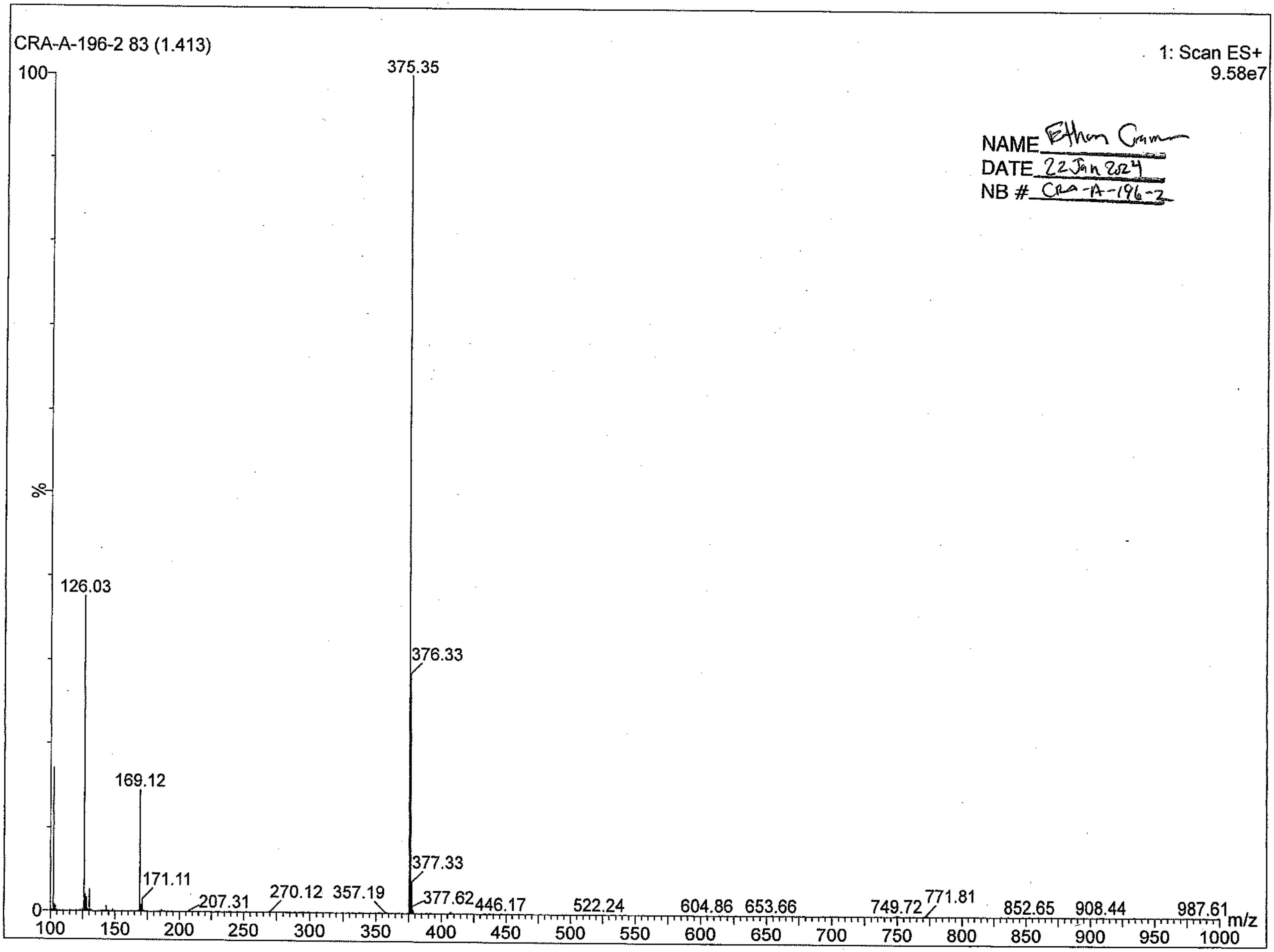

CRA-A-196-2 83 (1.413)
1: Scan ES+
9.58e7
NAME Ethan Cram
DATE 22 Jan 2024
NB # CRA-A-196-2
100
%
0
375.35
126.03
376.33
169.12
377.33
171.11
207.31
270.12
357.19
377.62
446.17
522.24
604.86
653.66
749.72
771.81
852.65
908.44
987.61
m/z
100 150 200 250 300 350 400 450 500 550 600 650 700 750 800 850 900 950 1000

SAMPLE INFORMATION

Sample Name: CRA-A-196-2
Injection Volume: 7.00 ul
Run Time: 9.0 Minutes
Date Acquired: 1/15/2024 9:55:41 AM EST
Date Processed: 1/15/2024 10:07:43 AM EST
Sample Set Name: Template
Acq. Method Set: BEH_C18_PDA_75mm
Processing Method: BEH_C18_PDA
Channel Name: 254nm

Method Notes:
Acquity UPLC BEH C18 1.7u (2.1x75mm)
Flow Rate : 0.5 mL/min
Solvent A : 0.1% TFA in Waters
Solvent B : 0.1% TFA in Acetonitrile
Solvent Gradient Program:

Time (min)	%A	% B
0:00	95	5
6:00	0	100
8:00	0	100
9:00	95	5

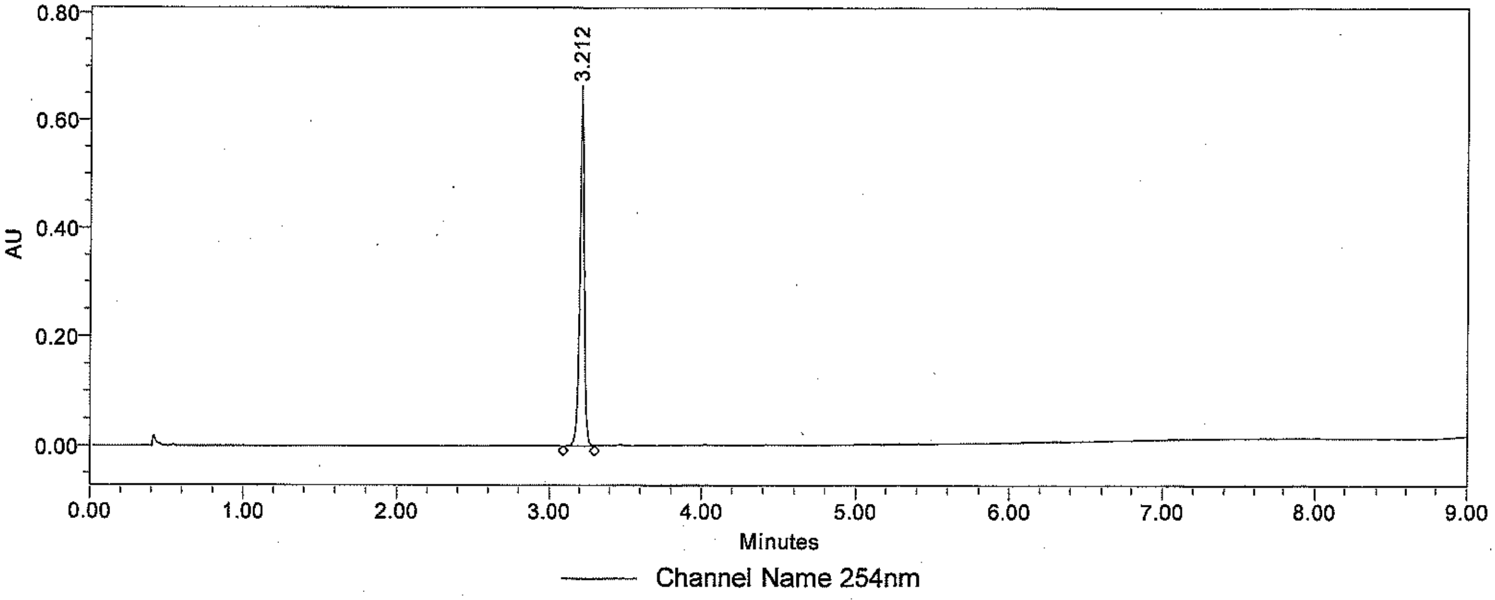

Peak Results

	RT	Area	Int Type	Width (sec)	% Area
1	3.212	1277492	VV	12.449	100.00

Name: Ethan Cramer

Date: 22 Jan 2024

NB #: CRA-A-196-2

CERTIFICATE OF ANALYSIS

Compound Name: BPN-0037601-AA-001
ALB Number: ALB-238169
Batch: 1
Lot Number: CRA-A-182-4
Molecular Formula: $C_{20}H_{20}N_4O_2$
Molecular Weight: 348.40
Last Solvent: Acetonitrile, Water

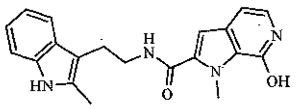

2x. BPN- 37601

TEST	RESULT/REFERENCE
Appearance	White Solid
NMR Spectrum	^1H, 500 MHz, Dimethyl Sulfoxide-d_6, Consistent - Attached
Mass Spectrum	ESI, m/z 349 $[M + H]^+$, Attached
UPLC	>99% (area %), ACQUITY UPLC BEH C18 (2.1 *75) mm, 1.7 micron Column, UV 254 nm Detection, Attached

Approved By

Date 1-10-2024

For Research Purposes Only. Not Intended for Food or Drug Use.

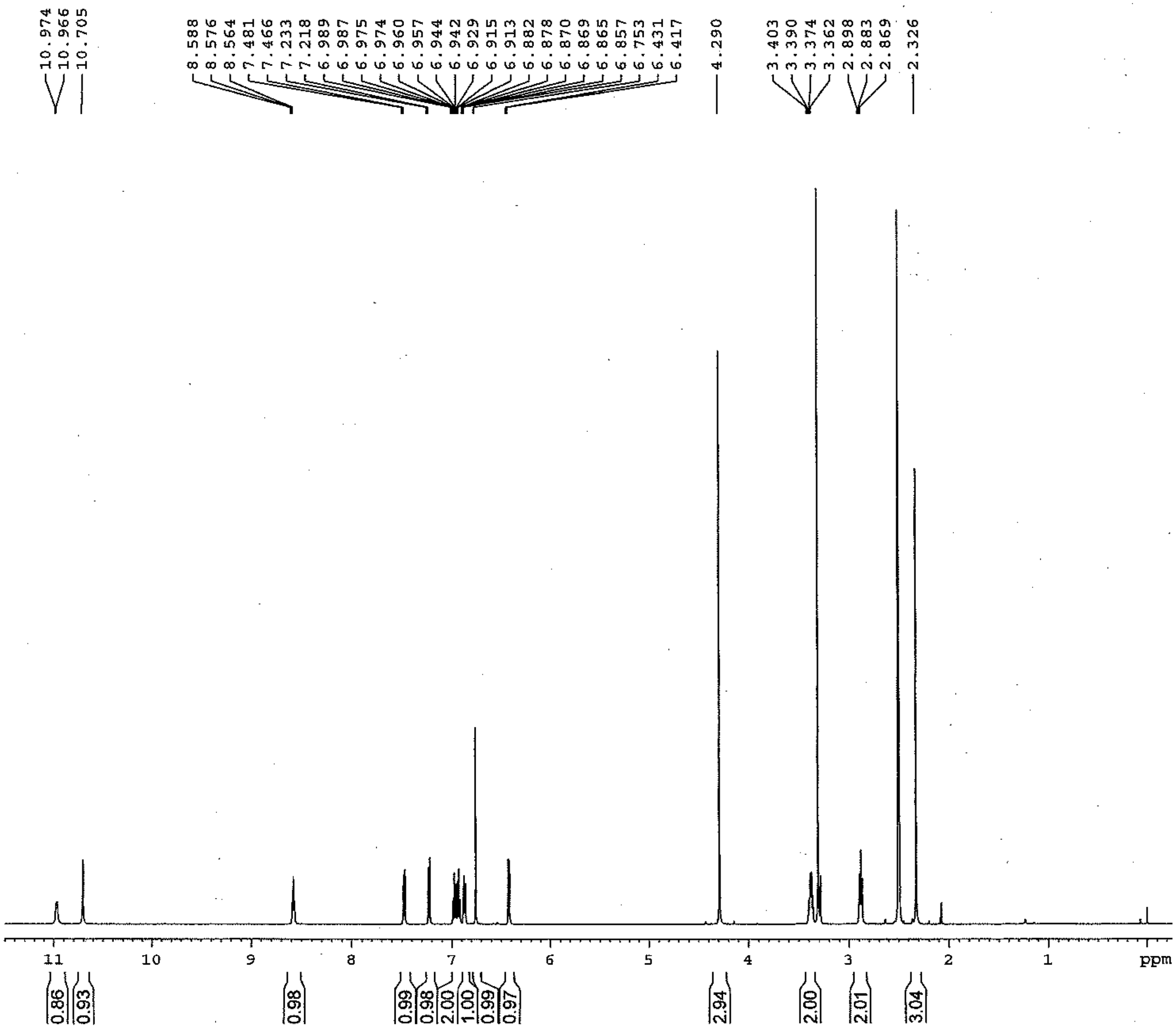

Name Ethan Orame

Date 03 Jan 2024

NB # CRA-A-182-4

NAME	CRA-A-182-4
EXPNO	10
PROCNO	1
Date_	20231221
Time	10.27 h
INSTRUM	Avance Neo
PROBHD	Z167419_0029 (
PULPROG	zg30
TD	65536
SOLVENT	DMSO
NS	32
DS	2
SWH	10000.000 Hz
FIDRES	0.305176 Hz
AQ	3.2768500 sec
RG	101
DW	50.000 usec
DE	11.14 usec
TE	300.0 K
D1	1.00000000 sec
TD0	1
SFO1	500.1330883 MHz
NUC1	1H
P0	2.67 usec
P1	8.00 usec
SI	65536
SF	500.1300041 MHz
WDW	EM
SSB	0
LB	0.30 Hz
GB	0
PC	1.00

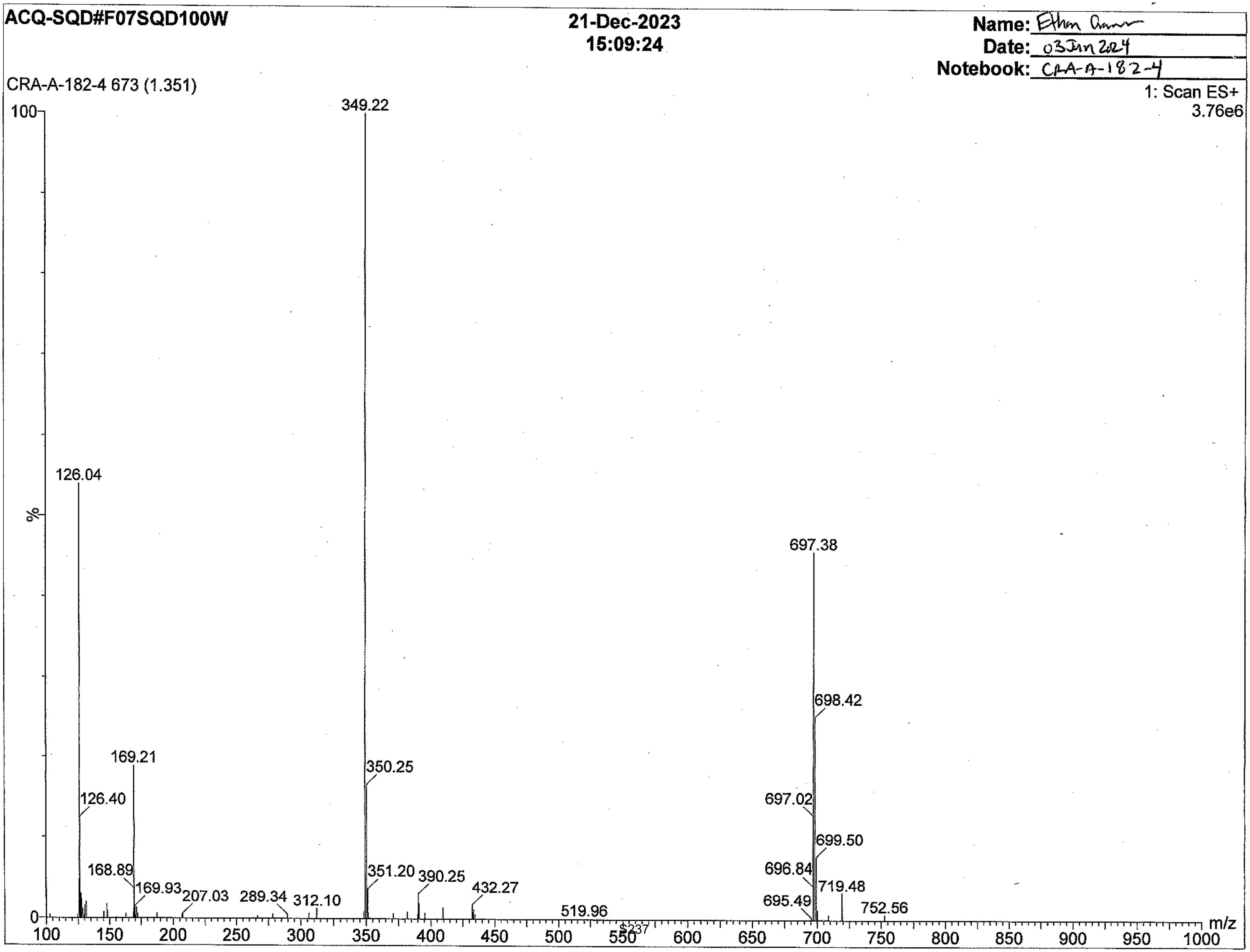

ACQ-SQD#F07SQD100W
21-Dec-2023
15:09:24
Name: Ethan [illegible]
Date: 03Jan2024
Notebook: CRA-A-182-4
CRA-A-182-4 673 (1.351)
1: Scan ES+
3.76e6
100
%
0
349.22
126.04
697.38
698.42
169.21
350.25
126.40
697.02
699.50
168.89
351.20
390.25
696.84
169.93
207.03
289.34
312.10
432.27
719.48
695.49
752.56
519.96
100
150
200
250
300
350
400
450
500
550
600
650
700
750
800
850
900
950
1000
m/z

SAMPLE INFORMATION

Sample Name:	CRA-A-182-4
Injection Volume:	3.00 ul
Run Time:	9.0 Minutes
Date Acquired:	12/21/2023 10:20:49 AM EST
Date Processed:	12/21/2023 10:32:51 AM EST
Sample Set Name:	Template
Acq. Method Set:	BEH_C18_PDA_75mm
Processing Method:	BEH_C18_PDA
Channel Name:	254nm

Method Notes:
Acquity UPLC BEH C18 1.7u (2.1x75mm)
Flow Rate : 0.5 mL/min
Solvent A : 0.1% TFA in Waters
Solvent B : 0.1% TFA in Acetonitrile
Solvent Gradient Program:

Time (min)	%A	% B
0:00	95	5
6:00	0	100
8:00	0	100
9:00	95	5

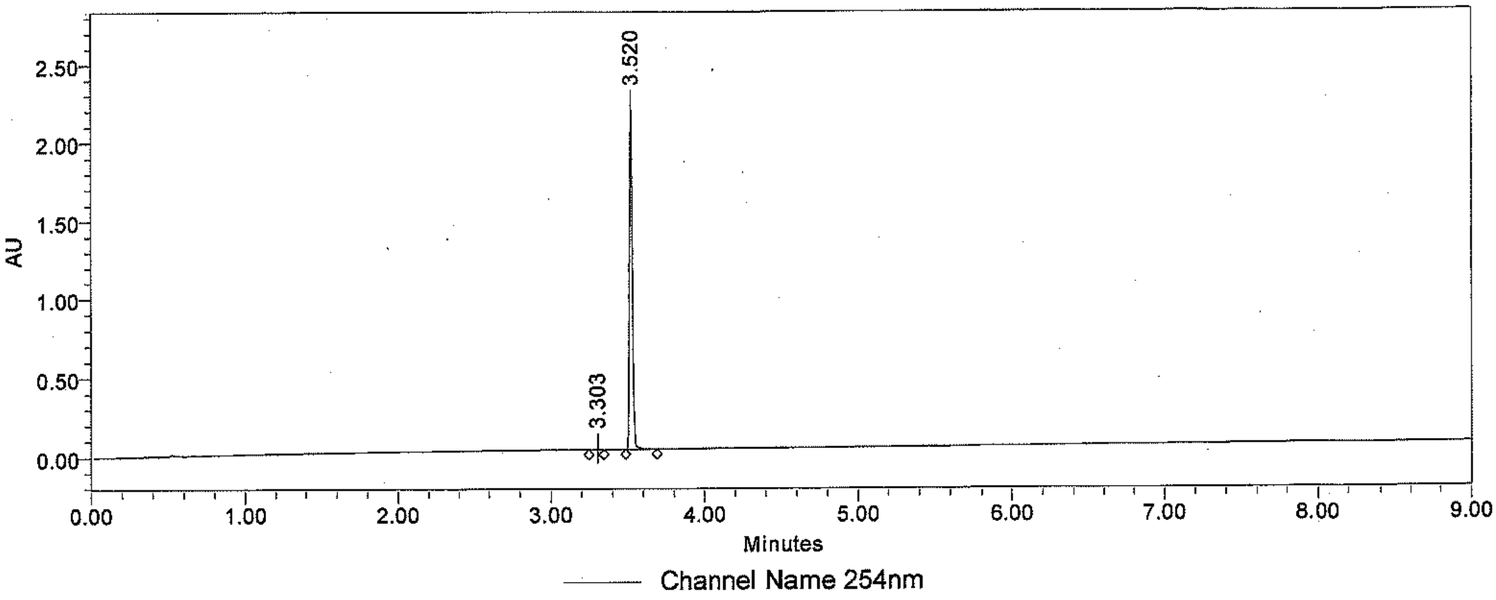

Peak Results

	RT	Area	Int Type	Width (sec)	% Area
1	3.303	15968	VV	5.750	0.55
2	3.520	2907808	VV	12.249	99.45

Name: Ethan Cramer

Date: 03Jan 2024

NB #: CRA-A-182-4

CERTIFICATE OF ANALYSIS

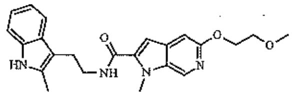

2y. BPN-37590

Compound Name: BPN-0037590-AA-001
ALB Number: ALB-237985
Batch: 1
Lot Number: ALK-D-148-2
Molecular Formula: $C_{23}H_{26}N_4O_3$
Molecular Weight: 406.48
Last Solvent: Water, Acetonitrile

TEST	RESULT/REFERENCE
Appearance	Off-white Solid
NMR Spectrum	^1H, 500 MHz, Dimethyl Sulfoxide-d_6, Consistent - Attached
Mass Spectrum	ESI, *m/z* 407 $[M + H]^+$, Attached
UPLC	97.7% (area %), ACQUITY UPLC BEH C18 (2.1 *75) mm, 1.7 micron Column, UV 254 nm Detection, Attached

Approved By: [signature]

Date: 12-20-2023

For Research Purposes Only. Not Intended for Food or Drug Use.

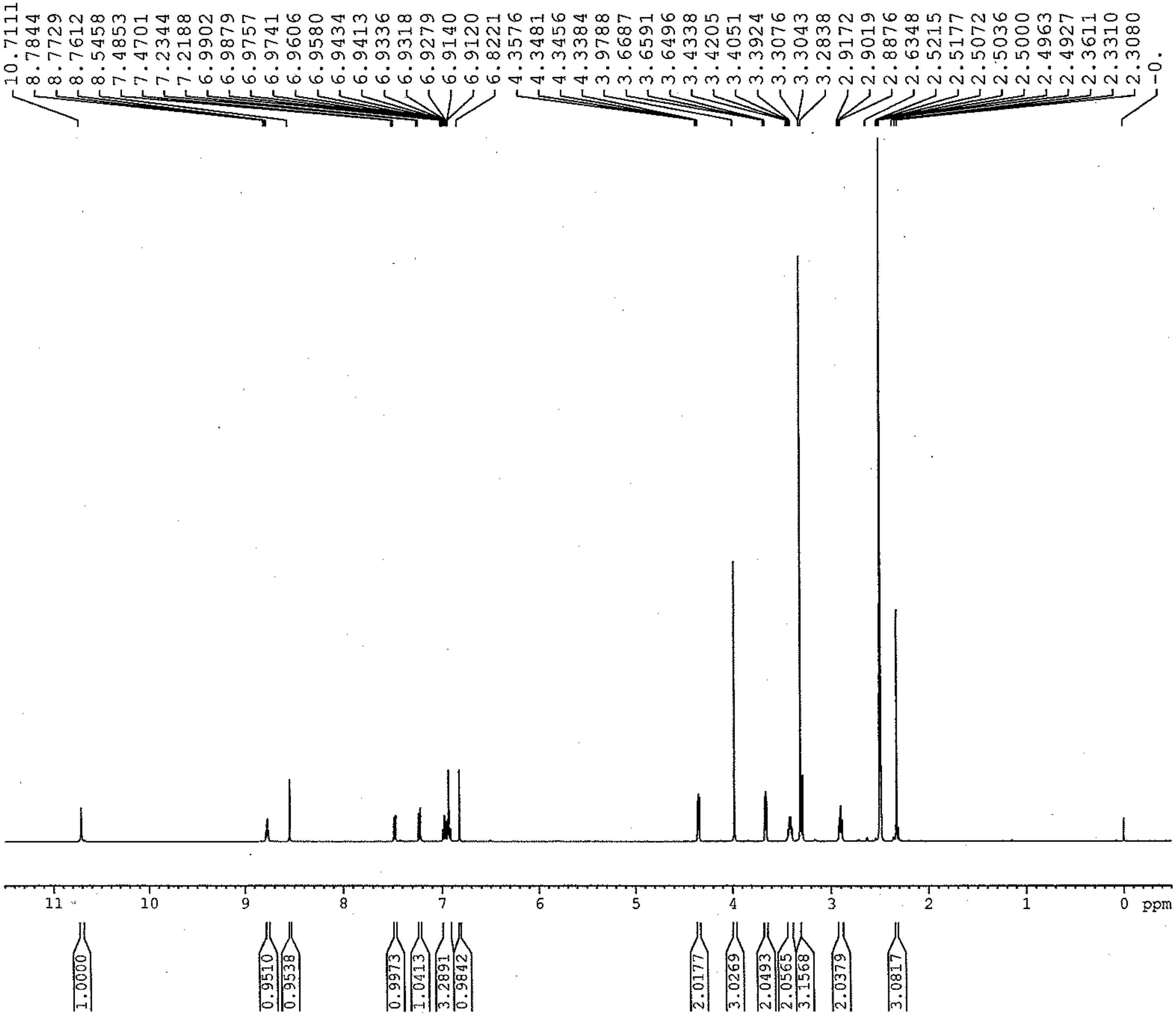

Name Marwah Albaker
Date 14-Dec-2023
NB # ALK-D-148-2

Current Data Parameters

NAME	ALK-D-148-2
EXPNO	10
PROCNO	1

F2 - Acquisition Parameters

Date_	20231214
Time	7.21 h
INSTRUM	Avance Neo
PROBHD	Z167419_0029 (
PULPROG	zg30
TD	65536
SOLVENT	DMSO
NS	64
DS	2
SWH	10000.000 Hz
FIDRES	0.305176 Hz
AQ	3.2767999 sec
RG	101
DW	50.000 usec
DE	11.14 usec
TE	300.0 K
D1	1.00000000 sec
TD0	1
SFO1	500.1330883 MHz
NUC1	1H
P0	2.67 usec
P1	8.00 usec
PLW1	24.22400093 W

F2 - Processing parameters

SI	65536
SF	500.1300043 MHz
WDW	EM
SSB	0
LB	0.30 Hz
GB	0
PC	1.00

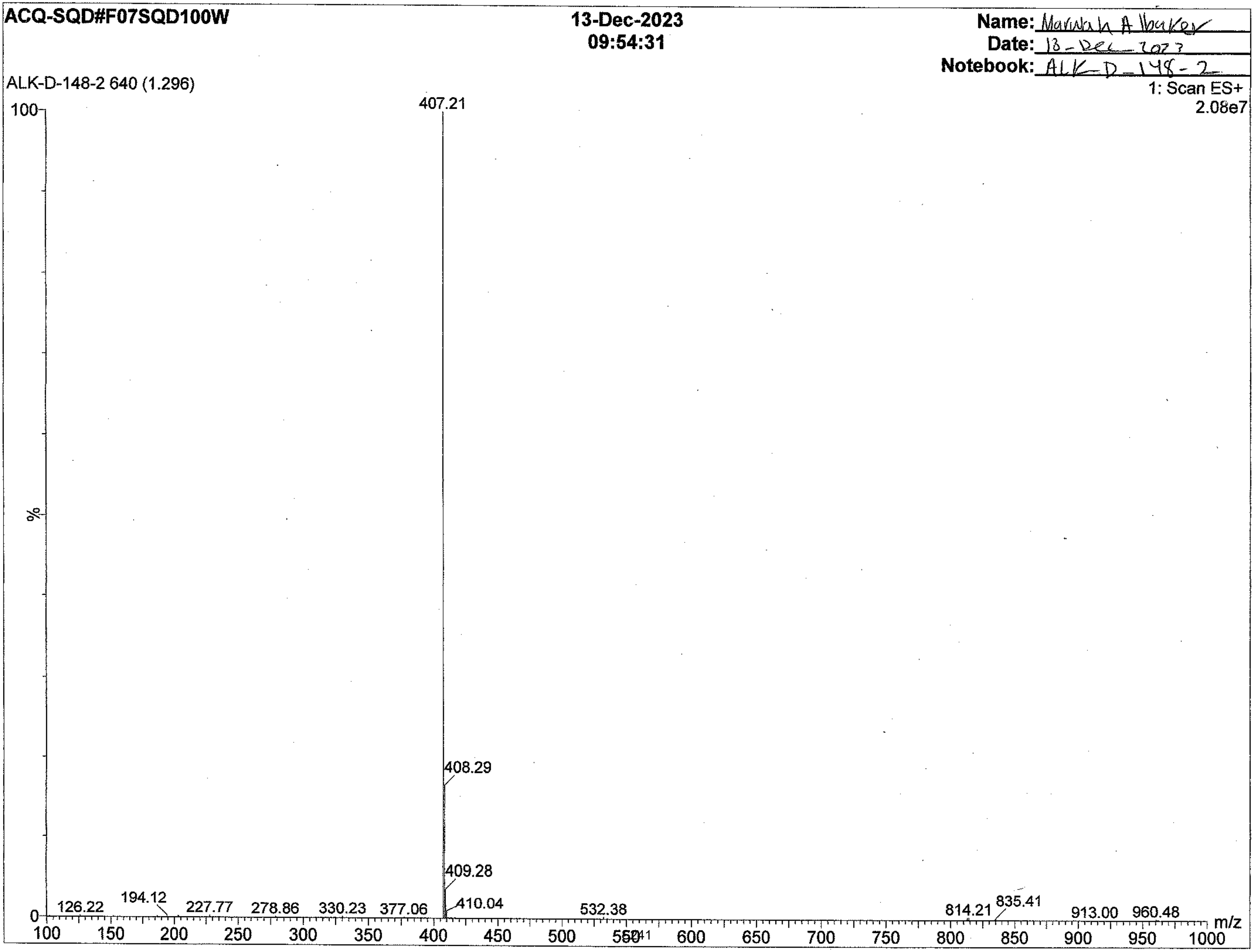
ACQ-SQD#F07SQD100W
13-Dec-2023
09:54:31
Name: Marwah Albaker
Date: 13-Dec-2023
Notebook: ALK-D-148-2
ALK-D-148-2 640 (1.296)
1: Scan ES+
2.08e7
407.21
408.29
409.28
410.04
126.22
194.12
227.77
278.86
330.23
377.06
532.38
814.21
835.41
913.00
960.48
%
m/z

SAMPLE INFORMATION

Sample Name:	ALK-D-148-2
Injection Volume:	3.00 ul
Run Time:	9.0 Minutes
Date Acquired:	12/13/2023 10:01:44 AM EST
Date Processed:	12/13/2023 10:48:13 AM EST
Sample Set Name:	Template
Acq. Method Set:	BEH_C18_PDA_75mm
Processing Method:	BEH_C18_PDA
Channel Name:	254nm

Method Notes:
Acquity UPLC BEH C18 1.7u (2.1x75mm)
Flow Rate : 0.5 mL/min
Solvent A : 0.1% TFA in Waters
Solvent B : 0.1% TFA in Acetonitrile
Solvent Gradient Program:

Time (min)	%A	% B
0:00	95	5
6:00	0	100
8:00	0	100
9:00	95	5

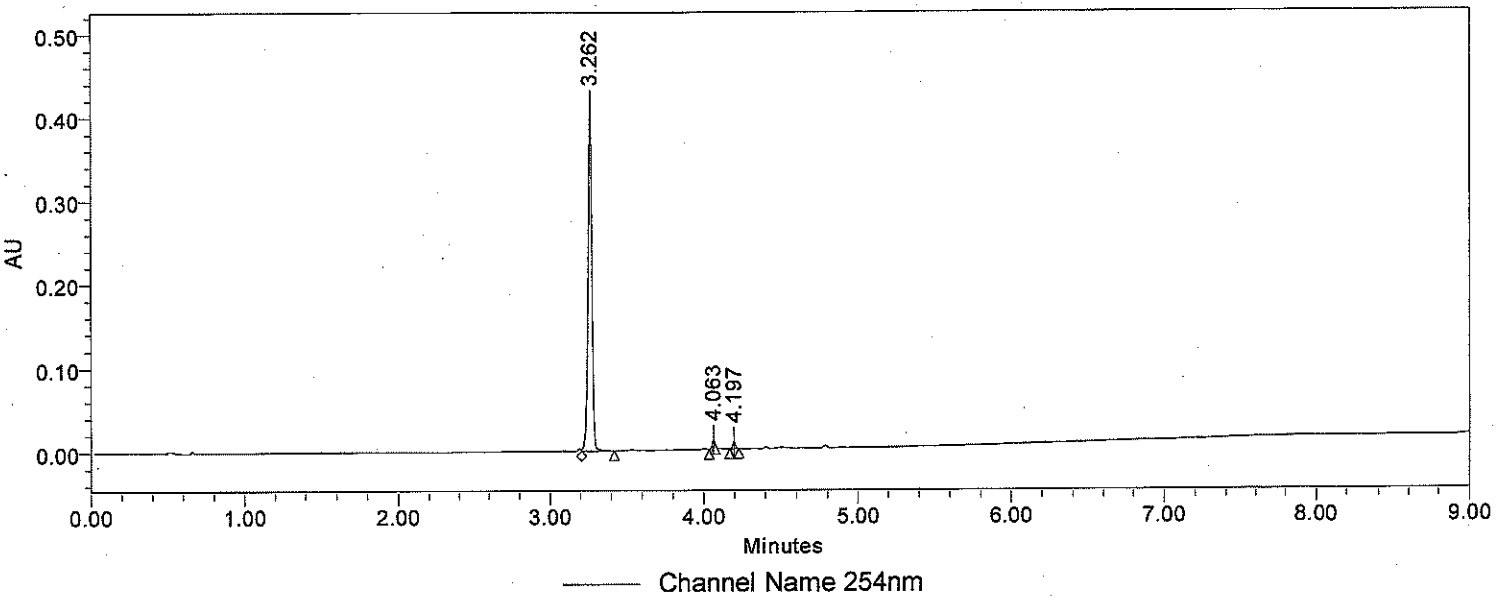

Peak Results

	RT	Area	Int Type	Width (sec)	% Area
1	3.262	725350	VB	12.899	97.70
2	4.063	6950	bb	2.401	0.94
3	4.197	10132	bb	3.551	1.36

Name: Marwah Albaker

Date: 13-Dec-2023

NB #: ALK-D-148-2

CERTIFICATE OF ANALYSIS

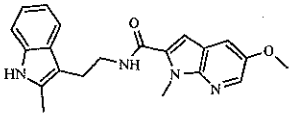

2z. BPN- 37272

Compound Name: BPN-0037272-AA-001
ALB Number: ALB-236739
Batch: 1
Lot Number: QUA-C-81-2
Molecular Formula: $C_{21}H_{22}N_4O_2$
Molecular Weight: 362.42
Last Solvent: Acetonitrile, Water

TEST	RESULT/REFERENCE
Appearance	Off-white Solid
NMR Spectrum	^{1}H, 500 MHz, Dimethyl Sulfoxide-d_6, Consistent - Attached
Mass Spectrum	ESI, *m/z* 363 $[M + H]^+$, Attached
UPLC	>99% (area %), ACQUITY UPLC BEH C18 (2.1 *75) mm, 1.7 micron Column, UV 254 nm Detection, Attached

Maura Maychack

Approved By

9-20-2023

Date

For Research Purposes Only. Not Intended for Food or Drug Use

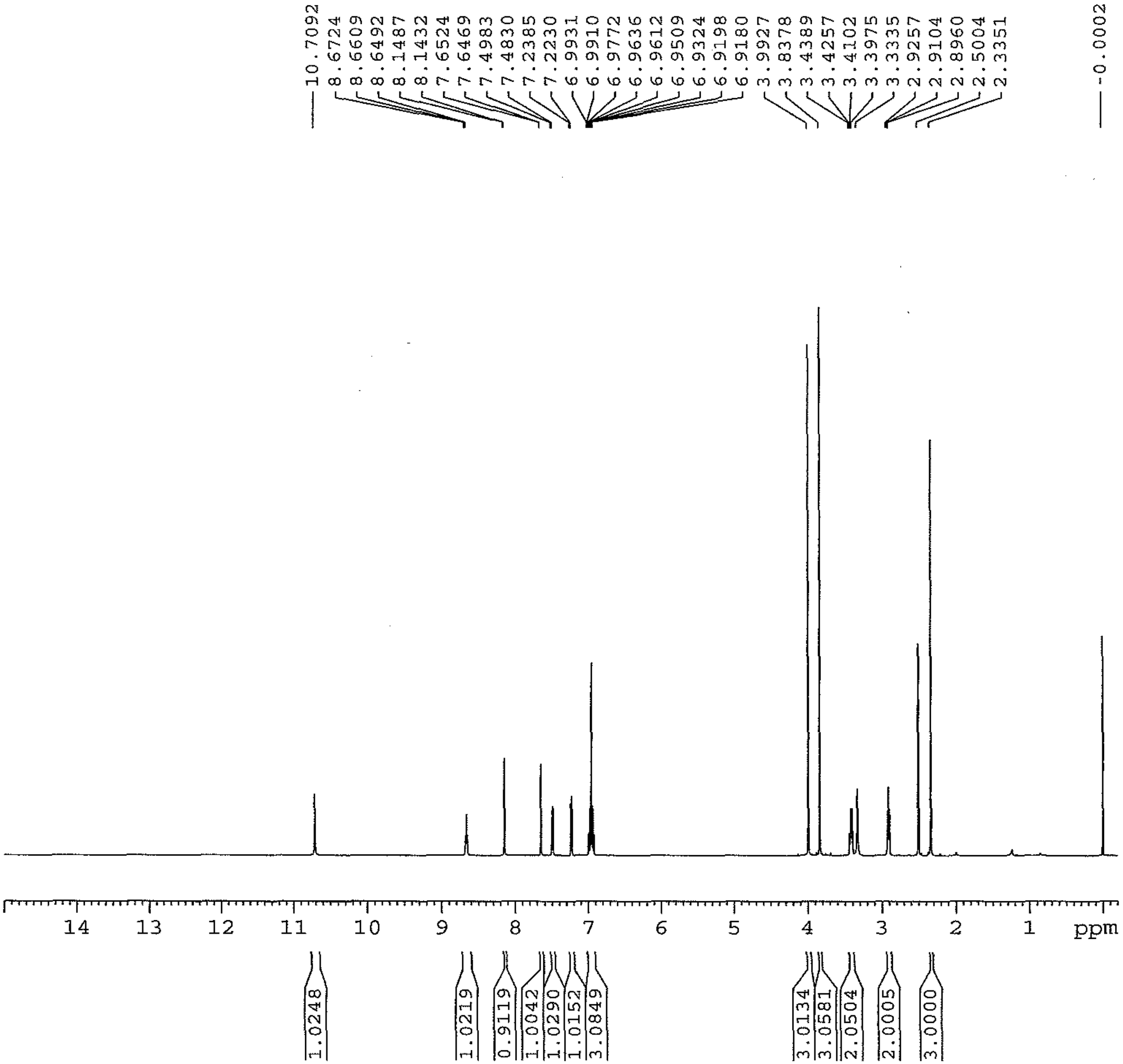

Name Tasdique Quadery
Date 09.13.23
NB # QUA-C-81-2

Current Data Parameters
NAME QUA-C-81-2
EXPNO 10
PROCNO 1

F2 - Acquisition Parameters
Date_ 20230913
Time 15.10 h
INSTRUM Avance Neo
PROBHD Z167419_0029 (
PULPROG zg30
TD 65536
SOLVENT DMSO
NS 32
DS 2
SWH 10000.000 Hz
FIDRES 0.305176 Hz
AQ 3.2767999 sec
RG 101
DW 50.000 usec
DE 11.14 usec
TE 300.0 K
D1 1.00000000 sec
TD0 1
SFO1 500.1330883 MHz
NUC1 1H
P0 2.67 usec
P1 8.00 usec
PLW1 24.22400093 W

F2 - Processing parameters
SI 65536
SF 500.1300041 MHz
WDW EM
SSB 0
LB 0.30 Hz
GB 0
PC 1.00

Openlynx Report

Vial:2:16
Date:12-Sep-2023
ID:
Time:15:46:21
File:QUA-C-81-1

Name: Tasdique Quadery
Date: 09.12.23
Notebook: QUA-C-81-2

Printed: Tue Sep 12 15:48:40 2023

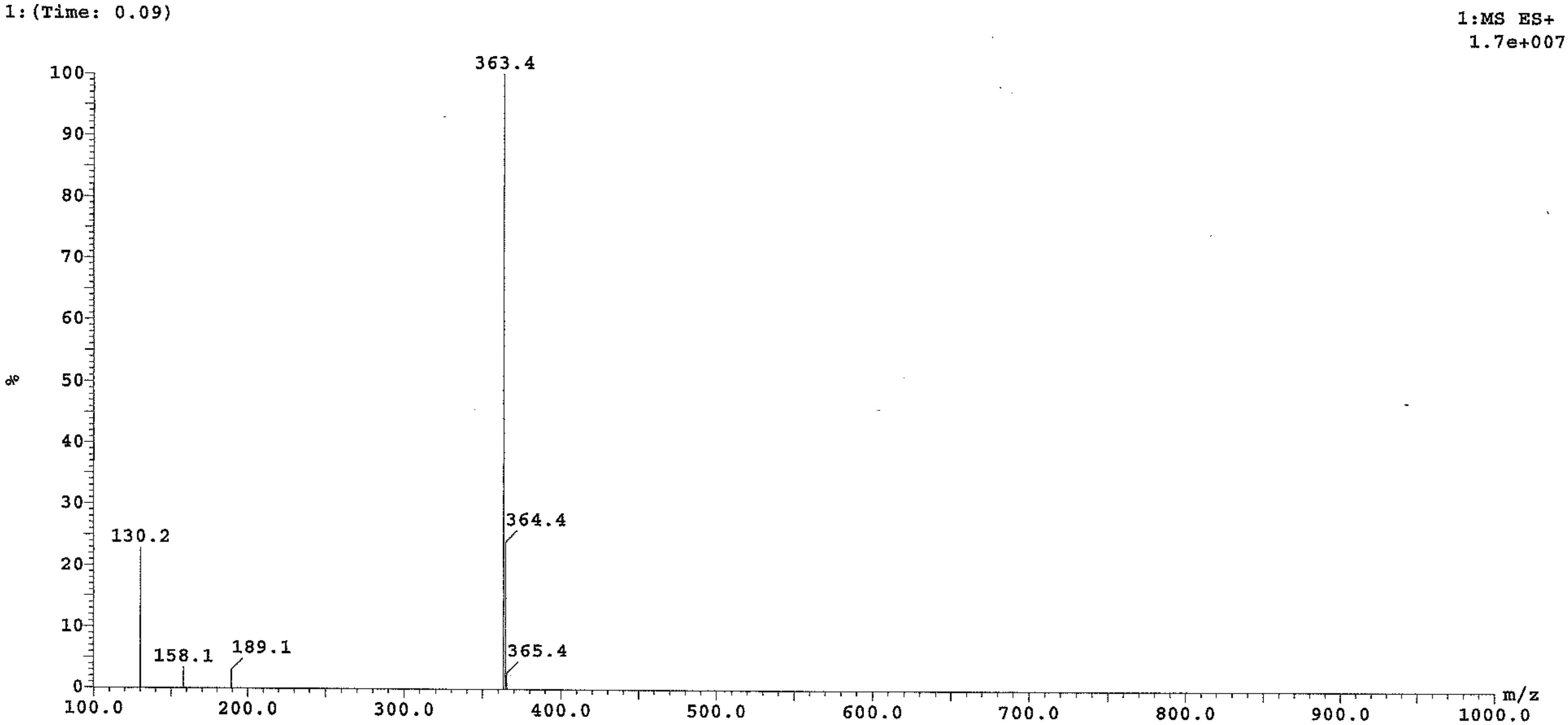

SAMPLE INFORMATION

Sample Name:	QUA-C-81-2
Injection Volume:	3.00 ul
Run Time:	9.0 Minutes
Date Acquired:	9/13/2023 9:40:05 AM EDT
Date Processed:	9/13/2023 9:52:08 AM EDT
Sample Set Name:	Template
Acq. Method Set:	BEH_C18_PDA_75mm
Processing Method:	BEH_C18_PDA
Channel Name:	254nm

Method Notes:
Acquity UPLC BEH C18 1.7u (2.1x75mm)
Flow Rate : 0.5 mL/min
Solvent A : 0.1% TFA in Waters
Solvent B : 0.1% TFA in Acetonitrile
Solvent Gradient Program:

Time (min)	%A	% B
0:00	95	5
6:00	0	100
8:00	0	100
9:00	95	5

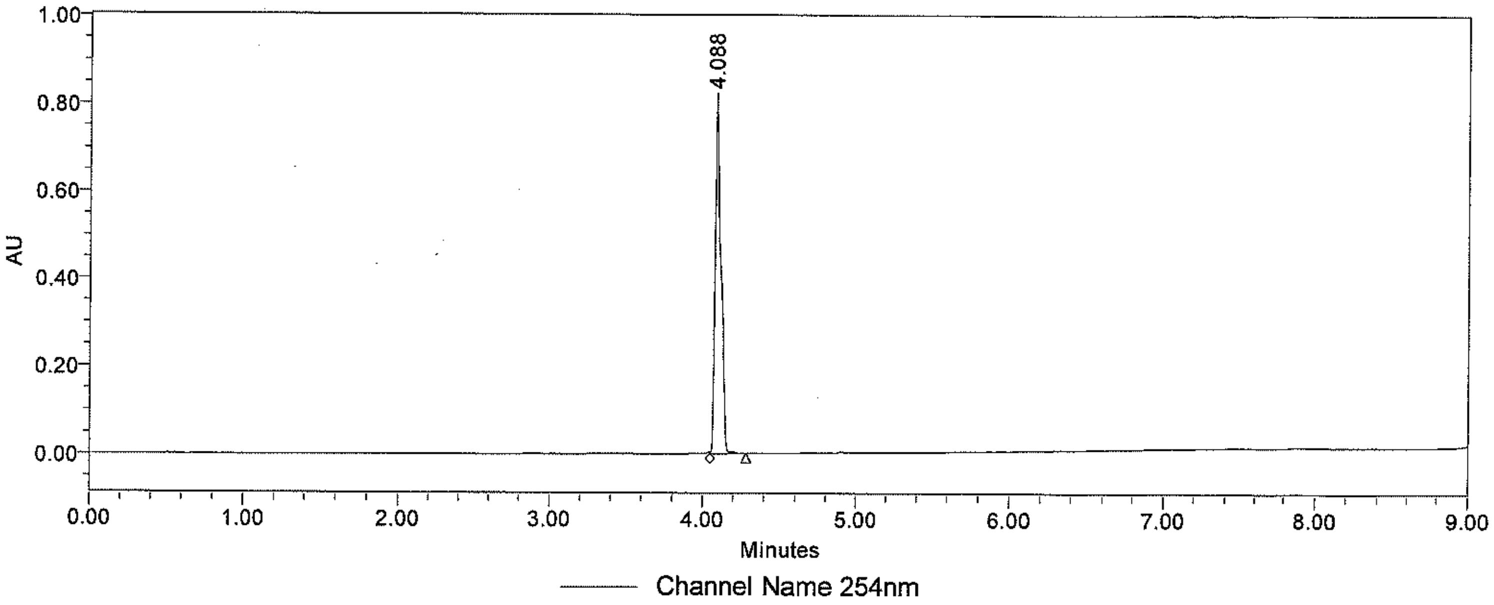

Peak Results

	RT	Area	Int Type	Width (sec)	% Area
1	4.088	2087715	VB	14.203	100.00

Name: Tasdique Quadery

Date: 09.13.23

NB #: QUA-C-81-2

CERTIFICATE OF ANALYSIS

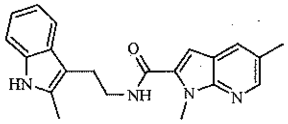

2aa.BPN - 37668

Compound Name: BPN-0037668-AA-001
ALB Number: ALB-238527
Batch: 1
Lot Number: ALK-D-180-2
Molecular Formula: $C_{21}H_{22}N_4O$
Molecular Weight: 346.43
Last Solvent: Water, Acetonitrile

TEST	RESULT/REFERENCE
Appearance	Off-white Solid
NMR Spectrum	^{1}H, 500 MHz, Dimethyl Sulfoxide-d_6, Consistent - Attached
Mass Spectrum	ESI, *m/z* 347 $[M + H]^+$, Attached
UPLC	>99% (area %), ACQUITY UPLC BEH C18 (2.1 *75) mm, 1.7 micron Column, UV 254 nm Detection, Attached

Maria Maychack

Approved By

2-7-2024

Date

For Research Purposes Only. Not Intended for Food or Drug Use.

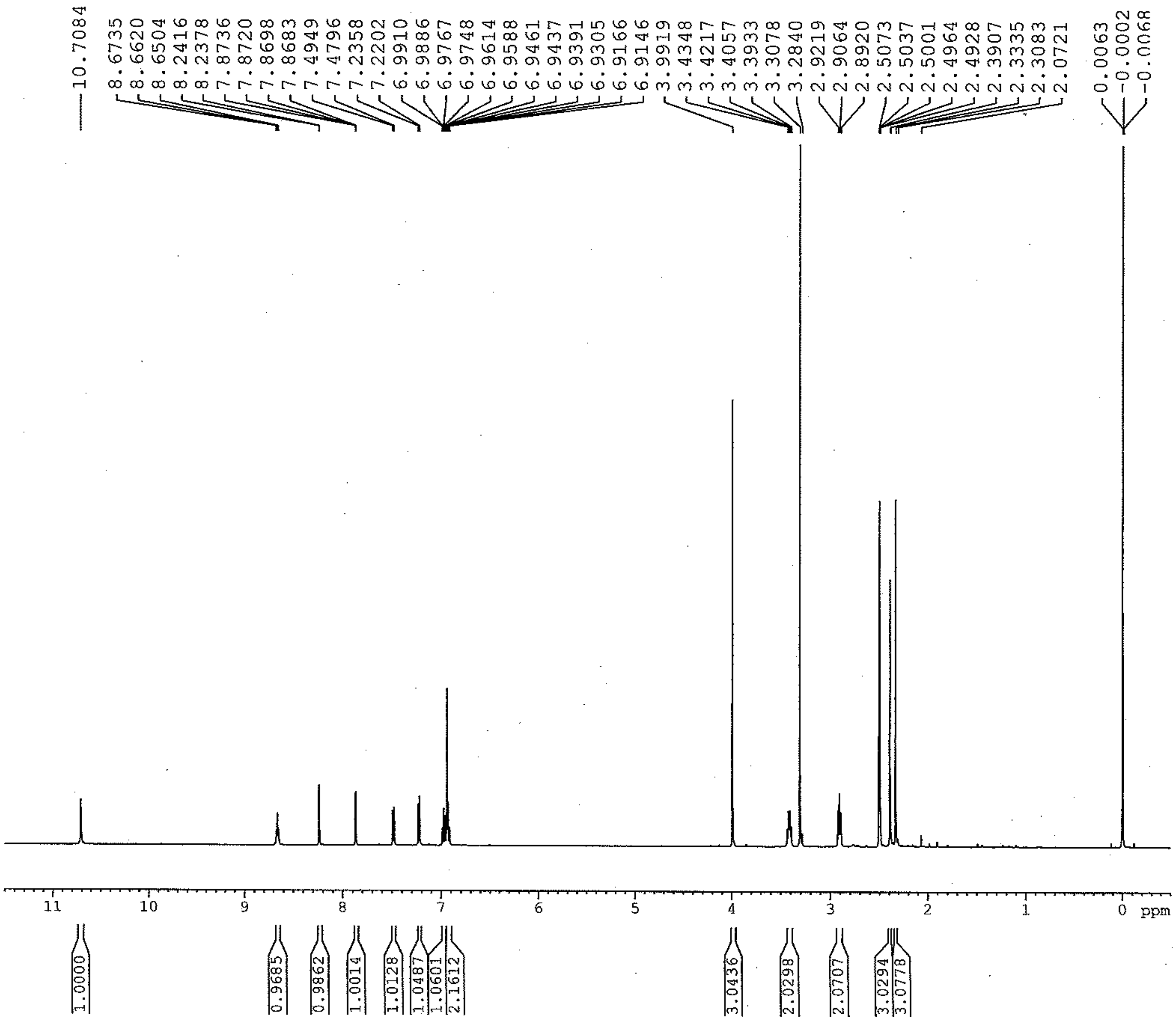

Name Marwah Albaker
Date 2-Feb-2024
NB # ALK-D-180-2

Current Data Parameters
NAME ALK-D-180-2
EXPNO 10
PROCNO 1

F2 - Acquisition Parameters
Date_ 20240202
Time 7.54 h
INSTRUM Avance Neo
PROBHD Z167419_0029 (
PULPROG zg30
TD 65536
SOLVENT DMSO
NS 64
DS 2
SWH 10000.000 Hz
FIDRES 0.305176 Hz
AQ 3.2767999 sec
RG 101
DW 50.000 usec
DE 11.14 usec
TE 300.0 K
D1 1.00000000 sec
TD0 1
SFO1 500.1330883 MHz
NUC1 1H
P0 2.67 usec
P1 8.00 usec
PLW1 24.22400093 W

F2 - Processing parameters
SI 65536
SF 500.1300042 MHz
WDW EM
SSB 0
LB 0.30 Hz
GB 0
PC 1.00

ACQ-SQD#F07SQD100W

01-Feb-2024
12:59:28

Name: Marwah Albaker
Date: 1-Feb-2024
Notebook: ALK-D-180-2

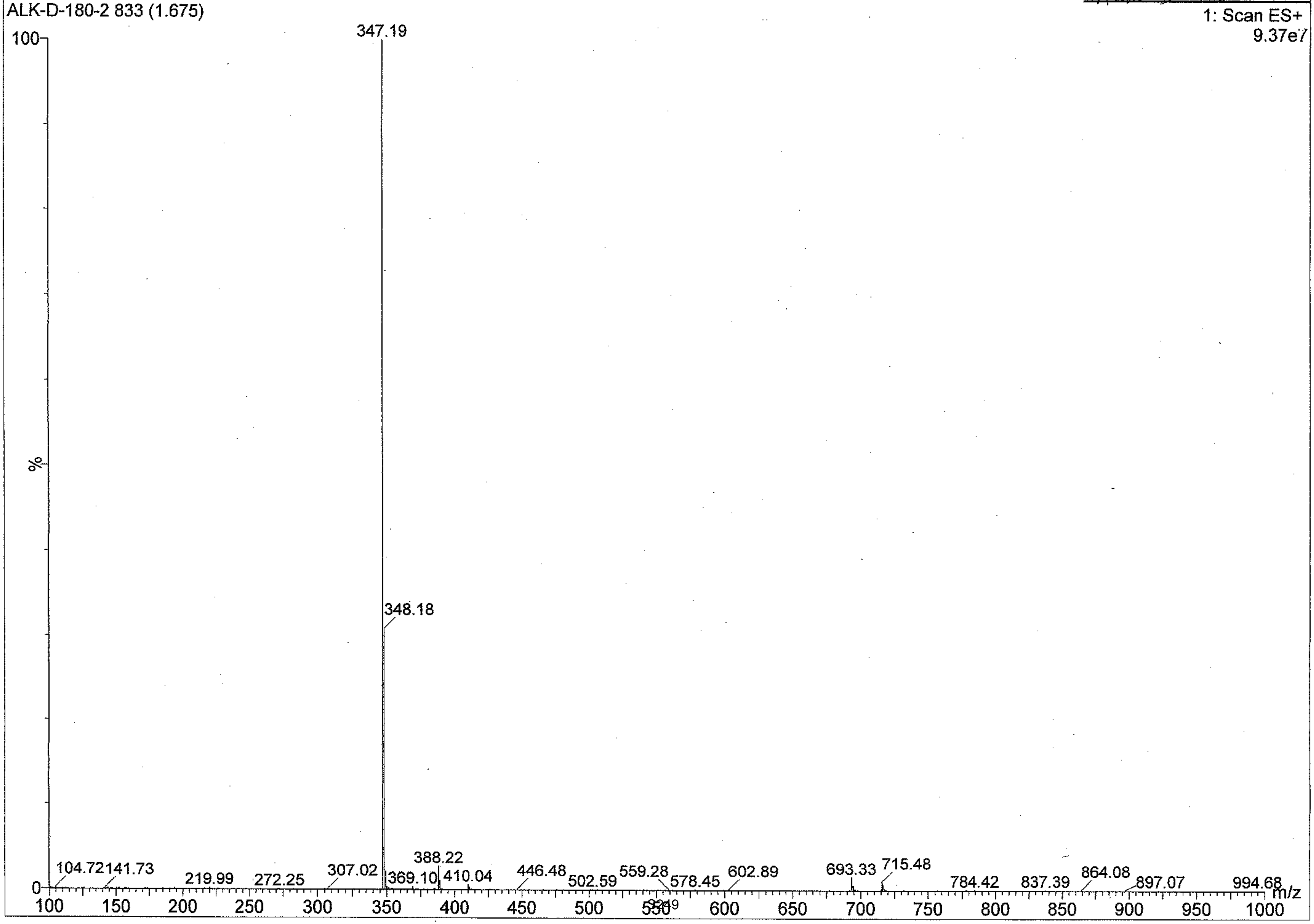

SAMPLE INFORMATION

Sample Name:	ALK-D-180-2
Injection Volume:	2.00 ul
Run Time:	9.0 Minutes
Date Acquired:	2/1/2024 1:07:17 PM EST
Date Processed:	2/1/2024 1:19:20 PM EST
Sample Set Name:	Template
Acq. Method Set:	BEH_C18_PDA_75mm
Processing Method:	BEH_C18_PDA
Channel Name:	254nm

Method Notes:
Acquity UPLC BEH C18 1.7u (2.1x75mm
Flow Rate : 0.5 mL/min
Solvent A : 0.1% TFA in Waters
Solvent B : 0.1% TFA in Acetonitrile
Solvent Gradient Program:

Time (min)	%A	% B
0:00	95	5
6:00	0	100
8:00	0	100
9:00	95	5

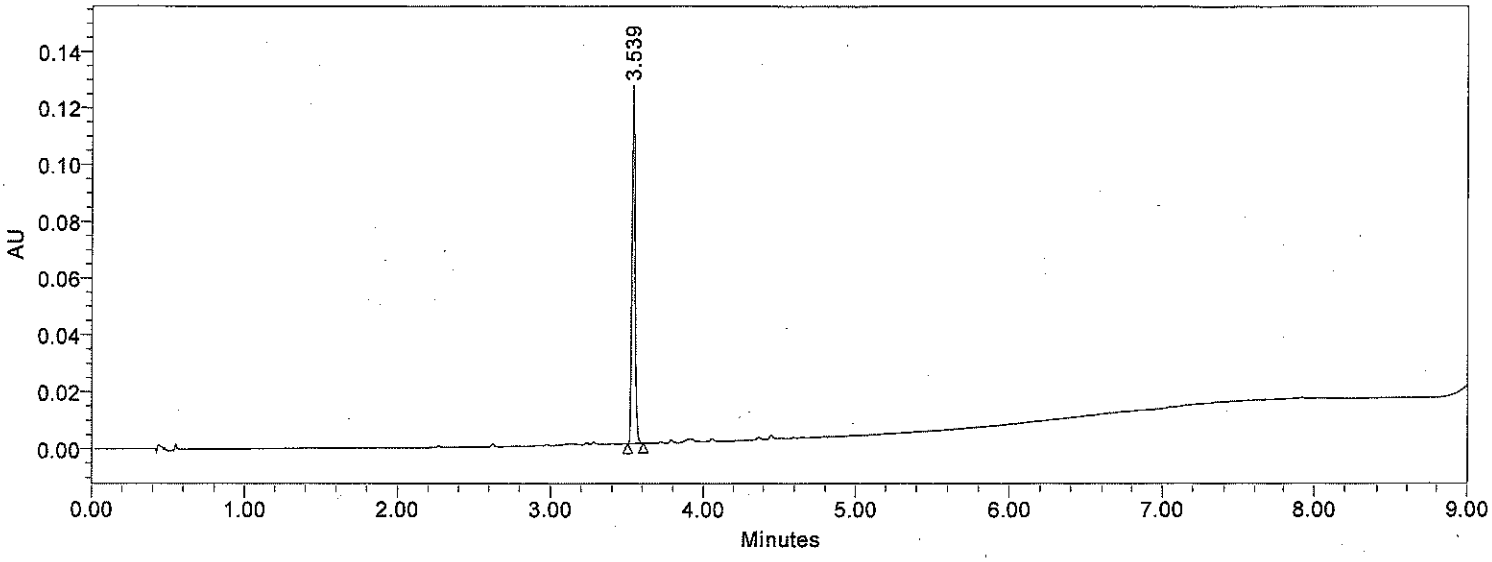

Peak Results

	RT	Area	Int Type	Width (sec)	% Area
1	3.539	170897	BB	6.150	100.00

Name: Marwah Albaker

Date: 1-Feb-2024

NB #: ALK-180-2

CERTIFICATE OF ANALYSIS

Compound Name: BPN-0037669-AA-001
ALB Number: ALB-238524
Batch: 1
Lot Number: ALK-D-177-2
Molecular Formula: $C_{20}H_{21}N_5O$
Molecular Weight: 347.41
Last Solvent: Water, Acetonitrile

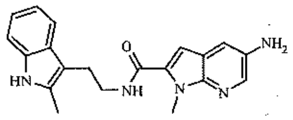

2bb.BPN- 37669

TEST	RESULT/REFERENCE
Appearance	Off-white Solid
NMR Spectrum	^{1}H, 500 MHz, Dimethyl Sulfoxide-d_6, Consistent - Attached
Mass Spectrum	ESI, *m/z* 348 $[M + H]^+$, Attached
UPLC	98.8% (area %), ACQUITY UPLC BEH C18 (2.1 *75) mm, 1.7 micron Column, UV 254 nm Detection, Attached

[illegible signature]

Approved By

2-7-2024

Date

For Research Purposes Only. Not Intended for Food or Drug Use.

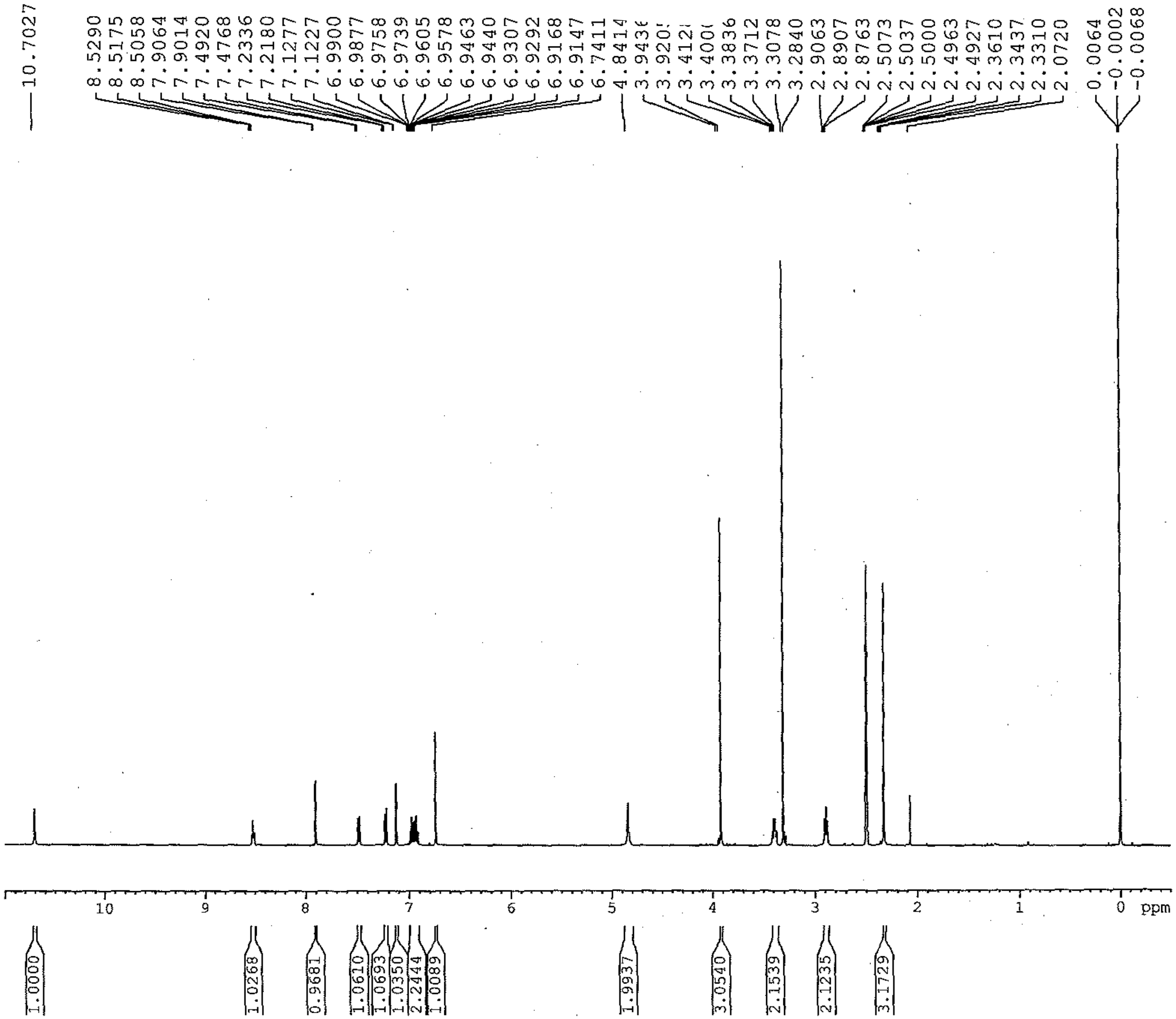

Name Marwah Albaker
Date 1-Feb-2024
NB # ALK-D-177-2

Current Data Parameters
NAME ALK-D-177-2
EXPNO 10
PROCNO 1

F2 - Acquisition Parameters
Date_ 20240201
Time 7.27 h
INSTRUM Avance Neo
PROBHD Z167419_0029 (
PULPROG zg30
TD 65536
SOLVENT DMSO
NS 64
DS 2
SWH 10000.000 Hz
FIDRES 0.305176 Hz
AQ 3.2767999 sec
RG 101
DW 50.000 usec
DE 11.14 usec
TE 300.0 K
D1 1.00000000 sec
TD0 1
SFO1 500.1330883 MHz
NUC1 1H
P0 2.67 usec
P1 8.00 usec
PLW1 24.22400093 W

F2 - Processing parameters
SI 65536
SF 500.1300042 MHz
WDW EM
SSB 0
LB 0.30 Hz
GB 0
PC 1.00

ACQ-SQD#F07SQD100W

31-Jan-2024
09:16:55

Name: Marwah Albaker
Date: 31-Jan-2024
Notebook: ALK-D-177-2

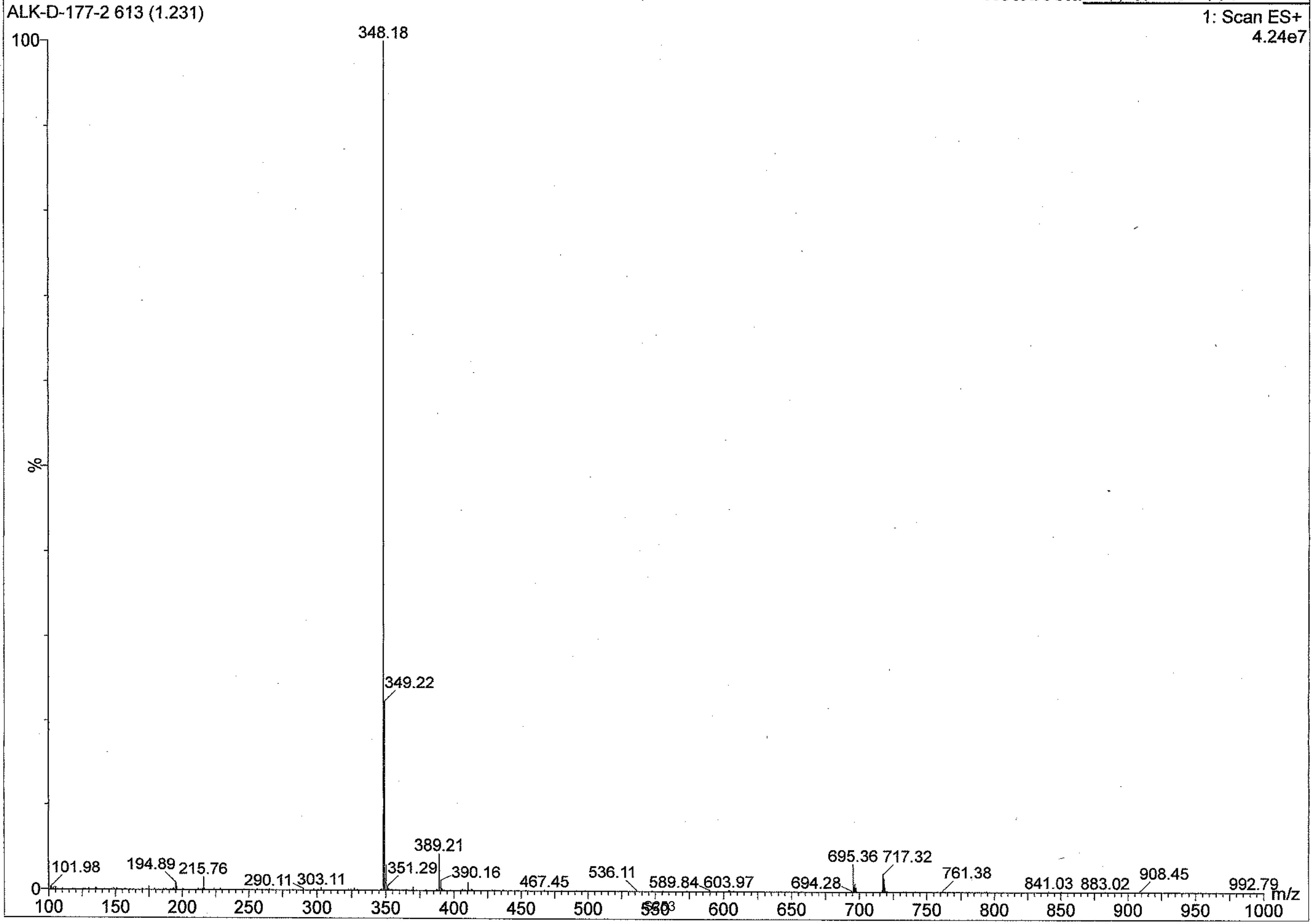

SAMPLE INFORMATION

Sample Name: ALK-D-177-2
Injection Volume: 2.00 ul
Run Time: 9.0 Minutes
Date Acquired: 1/31/2024 10:01:06 AM EST
Date Processed: 1/31/2024 10:20:59 AM EST
Sample Set Name: Template
Acq. Method Set: BEH_C18_PDA_75mm
Processing Method: BEH_C18_PDA
Channel Name: 254nm

Method Notes:
Acquity UPLC BEH C18 1.7u (2.1x75mm)
Flow Rate : 0.5 mL/min
Solvent A : 0.1% TFA in Waters
Solvent B : 0.1% TFA in Acetonitrile
Solvent Gradient Program:

Time (min)	%A	% B
0:00	95	5
6:00	0	100
8:00	0	100
9:00	95	5

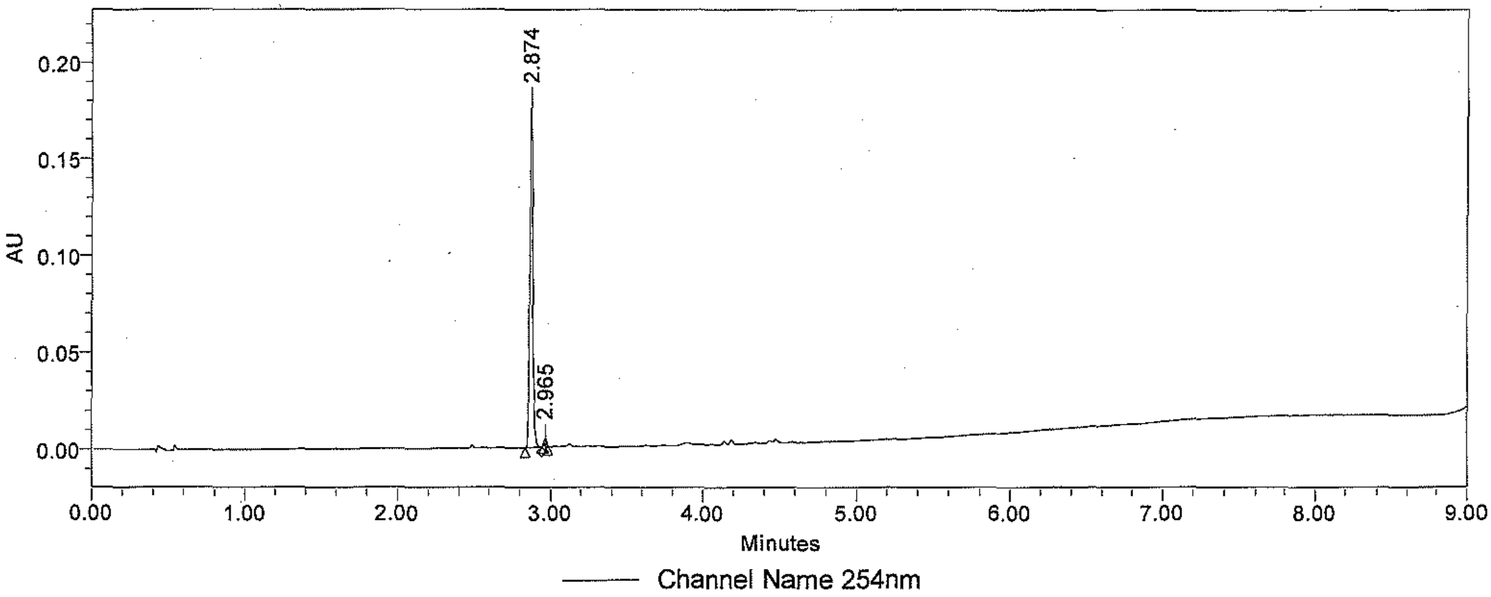

Peak Results

	RT	Area	Int Type	Width (sec)	% Area
1	2.874	245489	BV	6.100	98.83
2	2.965	2894	bb	1.750	1.17

Name: Marwah Albaker

Date: 31 - Jan - 2024

NB #: ALK-D-177-2
